# The brown fiber phenotype in cotton line SA-40 is linked to a missing Ty3-like retrotransposon upstream of the *GhTT2_A07*

**DOI:** 10.3389/fpls.2025.1668965

**Published:** 2025-09-03

**Authors:** Ganesh Pinnika, Gregory N. Thyssen, Crista A. Madison, Ping Li, David D. Fang, Marina Naoumkina, Doug J. Hinchliffe

**Affiliations:** ^1^ Cotton Fiber Bioscience and Utilization Research Unit, United States Department of Agriculture Agricultural Research Service (USDA-ARS), Southern Regional Research Center (SRRC), New Orleans, LA, United States; ^2^ Cotton Quality and Innovation Research Unit, United States Department of Agriculture Agricultural Research Service (USDA-ARS), SRRC, New Orleans, LA, United States

**Keywords:** cotton, Lc1 locus, rust-brown fiber, flame-retardant, flavonoid, proanthocyanidin

## Abstract

**Introduction:**

The naturally brown colored fibers of some cottons exist in varying shades of brown. Linkage analyses have revealed up to six individual loci (*Lc1–Lc6*) for brown color and suggested a separate genetic basis for each color value of fibers. It was previously reported that the brown color and flame-retardant (FR) properties of *Lc1* fibers resulted from an upstream inversion mutation that highly upregulates the *GhTT2_A07* gene and, consequently, the entire phenylpropanoid pathway. However, the genes responsible for the coloration and FR of other brown cotton fibers remained undetermined.

**Methods:**

In the current study, we used a previously uncharacterized SA-40 germplasm that produces rust-brown cotton. An F_2_ mapping population derived from the cross of SA-40 and TM-1 was used in an association mapping study to elucidate the genetics of SA-40 rust-brown fiber color and FR.

**Results and discussion:**

The rust-brown locus was mapped to *GhTT2* on chromosome A07 (*GhTT2_A07*). Comparison of the upstream sequence of *GhTT2_A07* between TM-1 and SA-40 revealed the absence of a Ty3-like LTR retrotransposon in the SA-40 line. No remnants of the LTR retrotransposon in the SA-40 indicated that the retrotransposon insertion did not happen in that line. However, all investigated white cotton lines in this study displayed the presence of the LTR retrotransposon upstream of *GhTT2_A07*. Transcript analysis showed that the absence of retrotransposon upstream of *GhTT2_A07* was associated with higher expression of this gene, but at lower levels than previously reported for the upstream inversion mutation in the MC-BL line, where the retrotransposon has been removed by structural rearrangement. Our finding suggests that the value of brown coloration in *Lc1* fibers is directly controlled by the expression level of *GhTT2_A07*, with higher expression levels resulting in darker fibers.

## 1 Introduction

Cotton is a major crop grown mainly for raw fiber. It has been domesticated and cultivated for thousands of years (Lee and Fang, 2015). Early civilizations used naturally-colored cotton (NCC) to create intricate patterns in their textiles, with brown, red, rust, and green hues, rather than just white cotton (Crawford, 1924). With technological advancement, white cotton replaced NCC since its fiber has better qualities, making it better for spinning yarn. However, public interest in NCC has recently increased because it is considered environmentally friendly, as it eliminates pollution steps associated with the bleaching and dyeing of white cotton. The fiber lumen of naturally brown cotton (NBC) is filled with tannin pigments, which make the color stronger and more intense after laundering (Günaydin et al., 2019). NBC is naturally resistant to pests and can survive on depleted dry soils (Din et al., 2016; Madhu, 2024). The fiber of some NBC varieties shows flame-retardant (FR) properties (Hinchliffe et al., 2015, 2016; Nam et al., 2017, 2016).

Interest in the genetic regulation of NCC began in the early 20^th^ century, when it was determined that plants producing fibers in various shades of brown and green were allelomorphic to white fibers (Ware, 1932). Later, mapping studies have identified at least six genetic loci associated with different shades of brown fiber coloration (*Lc1*-*Lc6*), which were incompletely dominant relative to white cotton (Endrizzi and Kohel, 1966; Kohel, 1985). The *Lc1* locus was mapped on chromosome (Chr.) A07, while the *Lc2* on Chr. A06 of *G. hirsutum* (Wang et al., 2014). A genome-wide association study (GWAS) detected two quantitative trait loci (QTLs), including *qBF-A07-1* (*Gh_A07G2341*–*TT2*) and *qBF-A07-2* (*Gh_A07G0100*–*TTG1*), that regulate the shades of brown color in *G. hirsutum* (Wen et al., 2018).

Advancements in the molecular understanding of NBC traits have revealed that the flavonoid branch of phenylpropanoid pathways largely governs brown pigmentation. Proanthocyanidins (PAs) or condensed tannins are mostly responsible for the brown coloration of fibers. The ternary MBW complex of transcription factors, including MYB (*TT2*)–bHLH (*TT8*)–WD40 (*TTG1*), plays a vital role in the regulation of different branches of the flavonoid biosynthesis pathway and epidermal cell fate determination in higher plants (Yu et al., 2023). The MBW complex functions differently depending on participating MYB and bHLH transcription factors (Wang et al., 2022a). For example, an R2R3-MYB–*Lc1* or *GhTT2* inhibits expression of *ANS* directing metabolic flux toward PA biosynthesis, while cooperation between another R2R3-MYB–*Re* or *GhPAP1* and *GhTT8* promotes expression of *ANS* and anthocyanin biosynthesis in cotton (Hinchliffe et al., 2016; Li et al., 2019; Wang et al., 2022b; Wen et al., 2018; Yan et al., 2018). Moreover, it has been demonstrated that *GhTT16* negatively modulates the MBW complex and the PAs pathway (Li et al., 2024).

Recent studies have shown that the regulation of expression of PA biosynthetic genes influences fiber color intensity and shades. Ectopic expression of *GhTT2-3A* in fibers during the secondary cell wall (SCW) thickening stage resulted in a rise in PA content and brown mature fibers (Yan et al., 2018). Two R2R3-MYBs, *Lc* and *GhPAP1D*, were fused and expressed in fibers during the SCW deposition stage, increasing PAs and anthocyanins and producing reddish-brown fiber color at maturation (Ran et al., 2025).

Furthermore, changes in upstream regulatory regions, such as actions of transposable elements, structural rearrangements, insertions/deletions (Indels), or single nucleotide polymorphisms (SNPs) in promoter regions, can significantly influence the activity of key genes that control the phenylpropanoid pathway (Hinchliffe et al., 2016; Ke et al., 2022; Li et al., 2024). In particular, Hinchliffe et al. (2016) described a 1.4 Mb inversion on chromosome A07 that upregulates the *GhTT2-A07* gene, resulting in brown color and FR properties of fibers. A T-DNA insertion into a retrotransposon positioned in the upstream region of flavonol O-methyltransferase (*GhOMT1*) suppressed the activity of *GhOMT1*, which caused overproduction of anthocyanidins and diversified colors of fiber (Ke et al., 2022). Another study experimentally demonstrated that subtle variations, such as SNPs or Indels, in the promoter of *Gh_TT2* were related to its gain-of-function (Li et al., 2024). This intricate regulation underscores not only the biochemical complexity of PA biosynthesis but also the evolutionary and agronomic significance of naturally colored cotton varieties.

In addition to their distinct pigmentation, brown cotton fibers exhibit noteworthy FR compared with conventional white fibers. Early studies revealed that brown cotton fabrics release heat more slowly and maintain higher char yields, a phenomenon attributed to the interaction of tannin-like compounds with inorganic elements (Hinchliffe et al., 2015). Subsequent investigations demonstrated that elevated sodium and potassium content in brown fibers fosters the formation of metal-phenolic complexes, effectively stabilizing the fiber matrix during combustion (Nam et al., 2016). These complexes behave in a manner akin to intumescent FRs, inhibiting ignition and promoting self-extinguishing behavior under thermal stress (Nam et al., 2017). Consequently, cotton lines that accumulate both proanthocyanidins and key inorganic ions offer an appealing combination of environmental sustainability and improved safety, paving the way for advanced textile applications where natural coloration and FR are desired.

In our previous study, we identified a 1.4 Mb inversion upstream of the *GhTT2_A07* (*Lc1*) in the MC-BL line that resulted in the significant upregulation of this gene, and consequently the whole phenylpropanoid pathway, leading to enhanced pigmentation and FR properties (Hinchliffe et al., 2016). In the current study, we utilized a previously uncharacterized germplasm, SA-40, that has rust-brown fibers. We mapped the rust-brown trait of the SA-40 line to the *GhTT2_A07* locus, which coincides with the previously reported *Lc1* locus (Hinchliffe et al., 2016; Wen et al., 2018). We discovered that the Ty3-like LTR retrotransposon presently located about 7.5 kb upstream of *GhTT2_A07* in white cotton TM-1 is missing in the rust-brown cotton SA-40. The absence of the retrotransposon was associated with higher expression of the *GhTT2_A07* gene, resulting in a rust-brown coloration on the cotton fiber.

## 2 Materials and methods

### 2.1 Plant materials

The cotton accession SA-40 (PI number 528453) was obtained from the USDA Cotton Germplasm Repository in College Station, TX, and is also referred to as Texas Rust Brown. The Texas Marker-1 (TM-1), a white fiber cultivar, was used as the control. An F_2_ population of 508 individuals was developed from a cross between SA-40 and TM-1. Crosses were made from plants grown under controlled greenhouse conditions in 20 L pots approximately 25cm in diameter and 30cm in height containing Metro-Mix 366 potting soil (Sun Gro Horticulture Canada Ltd, Agawam, MA, USA). F_1_ plants were grown in a greenhouse during winter 2017 to produce F_2_ seeds. The F_2_ population was grown in the summer of 2018 in a New Orleans field (30.02° N, 90.09° W). For cotton ovule collection, the cotton lines SA-40, MC-BL (Hinchliffe et al., 2016), and TM-1, about 20 plants each, were grown in the summer of 2019 in a New Orleans field. Organic field practices were applied during the growing season. Previously reported 132 accessions of white cotton were used to obtain the sequence of the LTR retrotransposon upstream of *GhTT2_A07* (Thyssen et al., 2025).

### 2.2 Sample collection and nucleic acid isolation

Fiber samples were collected from developing fibers at 10 days post-anthesis (DPA) according to the previously published protocol (Hinchliffe et al., 2011). Total RNA was extracted using the Sigma Spectrum Plant Total RNA kit (Sigma-Aldrich, St. Louis, MO, USA) with an integrated DNase 1 treatment to eliminate genomic DNA contamination. Genomic DNA from parents and the F_2_ population was extracted from young leaf tissues using a modified CTAB protocol (Fang et al., 2010). RNA and DNA quality and quantity were confirmed by a NanoDrop 2000 spectrometer (Thermo Fisher Scientific, Waltham, MA) as previously described (Hinchliffe et al., 2011).

### 2.3 Bulked segregant analysis and genetic mapping

Bulked segregant analysis (BSA) sequencing was used to identify genetic variants between SA-40 and TM-1. We have prepared two bulks: the first contains 77 samples of brown cotton, and the second includes 77 white cotton F_2_ segregants. Novogene Corporation (Chula Vista, CA, USA) sequenced the samples on the Illumina HiSeq X Ten platform (150 bp paired-end reads). Sequence reads were aligned using bwa-mem2 (Vasimuddin et al., 2019) to the draft *Gossypium hirsutum* TM-1 JGI v1 reference genome (https://jgi.doe.gov/). Variant calling was conducted with bcftools (Li, 2011; Li et al., 2009) and manually verified. Identical variants, including SNPs and Indels, between the two bulks were subtracted across the genome. The subgenome-specific SNP markers were developed from the cluster of different variants on Chr. A07 as described by (Drenkard et al., 2000; Thyssen et al., 2014). Because the rust-brown mutation is dominant, only the mutant-specific genotypes of the SNP markers were analyzed in the F_2_ population. We optimized the annealing temperature for the markers to achieve the largest Δ Ct values between positive and negative samples. **Supplementary Table S1** provides the primer sequences of the SNP markers along with their optimal temperatures.

### 2.4 RNAseq and gene expression analysis

Total RNA isolated from developing fibers of 10 DPA from SA-40, MC-BL, and TM-1 was used for RNAseq analysis. Two biological replicates from each line were processed. Each biological replicate represented a pool of samples collected from 10 plants. Novogene Corporation (Chula Vista, CA, USA) conducted library preparations and sequencing on the Illumina HiSeq 2000 platform to yield 100-bp paired-end reads. Quality control of RNAseq data, including Q20, Q30 and GC, are provided in the **Supplementary Table S2**. Raw reads were quality filtered and trimmed using SICKLE (Joshi and J.N., 2011) and aligned to the *G. hirsutum* TM-1 reference genome (Zhang et al., 2015) with GSNAP software (Wu and Nacu, 2010). We used a different version of the TM-1 reference genome for aligning the RNAseq data compared to the version used for the BSA analysis. This choice was made because we had previously published transcript analysis for the MC-BL aligned to that version, although at different developmental time points (Hinchliffe et al., 2016). Gene-level counts were generated using BEDTools (Quinlan and Hall, 2010). Normalization and ANOVA for differentially expressed genes were performed as described by (Naoumkina et al., 2014). The Illumina raw reads data were deposited into the NCBI-SRA database under BioProject number PRJNA1291244.

### 2.5 Assessment of flame retardancy

Flame retardancy was evaluated via microscale combustion calorimetry (MCC) method on approximately 4 mg of fiber. Samples were accurately weighed into a ceramic specimen cup using a Sartorius CP2P-F microbalance (Sartorius Bohemia, NY, USA) mounted on a Scienceware Vibrasorb system (Bel-Art products, Wayne, NJ, USA) on a marble slab. Pyrolysis was conducted in an MCC-2 instrument (Deatak, Mchenry. IL, USA) at a constant heating rate of 1.2 ° C s ^-1^, with temperature ramped from 90 ° C to 550 ° C. Measured parameters using MCC model MCC-2 (Deatak, McHenry, IL, USA) included heat release capacity (HRC, J g^-1^ K^-1^), peak heat release rate (pHRR, W g^-1^), total heat release (tHR, KJ g^-1^), temperature at pHRR (° C), and percent char yield. Data was processed using MCC Curve Fit v.2 software (Deatak).

### 2.6 Quantitative color space analysis

Objective fiber color space measurements were obtained using a CR-400 Chroma Meter (Konica Minolta, Ramsey, NJ) to capture CIELab (International Commission on Illumination, Vienna, Austria) values (Ibraheem et al., 2012). Measurements in three technical replicates were taken per fiber sample and averaged. Differences in color of mature fibers of the F_2_ population were quantified by calculating ΔE values: $\text{Δ}E=\sqrt{{\left(L_{2}-L_{1}\right)}^{2}+{\left(a_{2}-\;a_{1}\right)}^{2}+\;{\left(b_{2}-\;b_{1}\right)}^{2}}$ . Where L* represents lightness (0 = black, 100 = white), a* represents the green-red axis (-a = green, +a = red) and b* represents the blue-yellow axis (-b = blue, +b = yellow). ΔE less than one is not perceptible to the human eye, while more than two is perceptible upon close observation. Colors are noticeably different for ΔE around 10, and completely different for ΔE over 50.

## 3 Results

### 3.1 Rust-brown fiber phenotype and inheritance

**Figure 1:**
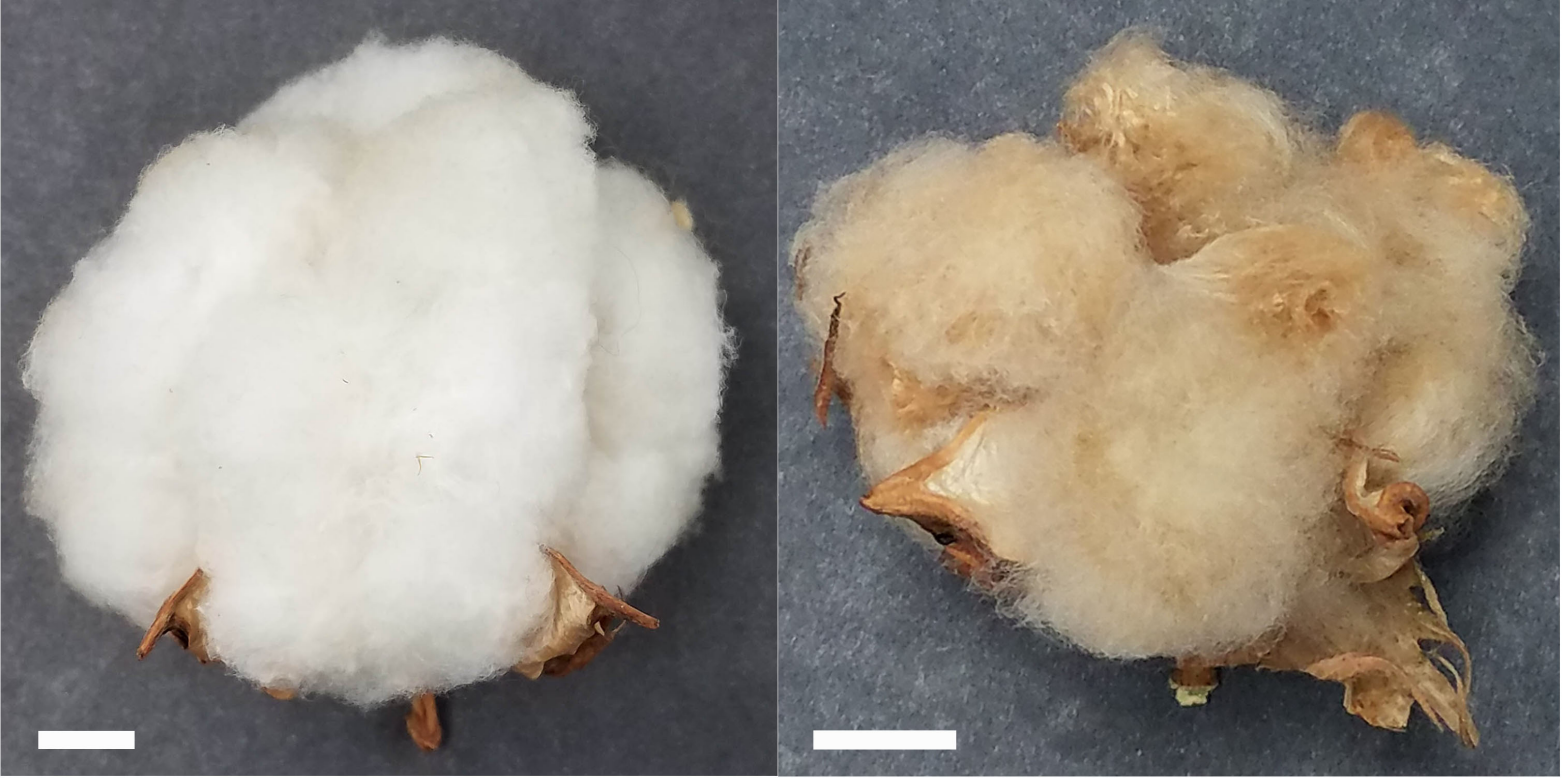
Open bolls of TM-1 (left) and SA-40 (right) cotton lines. Bar scale = 1cm shown in the lower left corner of each image.

SA-40, a previously uncharacterized line, exhibits green, normal-shaped leaves and stems, cream-colored petals on its flowers without spots, and a distinctive rust-brown lint color in mature cotton. We used Texas Marker-1 (TM-1) as a standard reference line (Kohel et al., 1970) for comparison of fiber properties and generating the F_2_ mapping population. **Figure 1** shows images of open bolls from SA-40 and TM-1 cotton lines.

CIELab color space analysis confirmed notable differences in fiber color parameters between these lines (**Table 1**). SA-40 fibers had a markedly lower L* value (indicating lower lightness or brightness) compared to TM-1, consistent with the darker appearance of brown fiber. Conversely, SA-40 showed higher positive a* and b* values, reflecting a stronger red and yellow hue component, respectively, relative to the essentially neutral white fiber of TM-1. These shifts in L*a*b* coordinates quantitatively characterize the rust-brown coloration of SA-40 fibers.

**Table 1:** CIELab color space values and microscale combustion calorimetry of TM-1 and SA-40 mature fibers.

Line	Color	CIE color space			Microscale combustion calorimetry				
		L*	a*	b*	Char yield (%)	HRC (J g^-1^ k^-1^)	pHRR (W g^-1^)	tHR (kJ g^-1^)	Temp (°C)
TM-1	White	96.49 ± 0.85	0.64 ± 0.26	10.25 ± 1.36	18 ± 0.5	148 ± 2	177 ± 3	8.0 ± 0.2	365 ± 6
SA-40	Brown	67.75 ± 0.41	11.58 ± 0.18	30.33 ± 0.53	20 ± 0.2	147 ± 4	177 ± 5	7.5 ± 0.0	370 ± 2

Values represent averages from three technical replicates. L* indicates whiteness (0=black; 100=white); a* indicates colors from greenish (negative values) to reddish (positive values); and b* indicates colors from bluish (negative values) to yellowish (positive values). HRC, heat release capacity; pHRR, peak heat release rate; tHR, total heat release.

In genetic crosses, the brown fiber trait exhibited incomplete dominance. F_1_ hybrids from the cross between SA-40 (brown fiber) and TM-1 (white fiber) produced tan-colored lint intermediate between the two parents, rather than purely brown or white. This intermediate phenotype in F_1_ indicates that the rust-brown allele is only partially dominant over the white allele. In the F_2_ generation, the observed segregation between brown/light brown (395) and white (113) lint phenotype fitted the expected 3:1 ratio with $\chi^{2}=2.06$ (*p*-value 0.15), consistent with a single gene inheritance. Thus, SA-40’s rust-brown fiber phenotype is governed by a single locus displaying partial dominance, as evidenced by the F_1_ fiber color and Mendelian segregation patterns, and this locus produces measurable shifts in standard colorimetric indices relative to white fiber.

### 3.2 Enhanced FR in F_2_ segregating population

**Figure 2:**
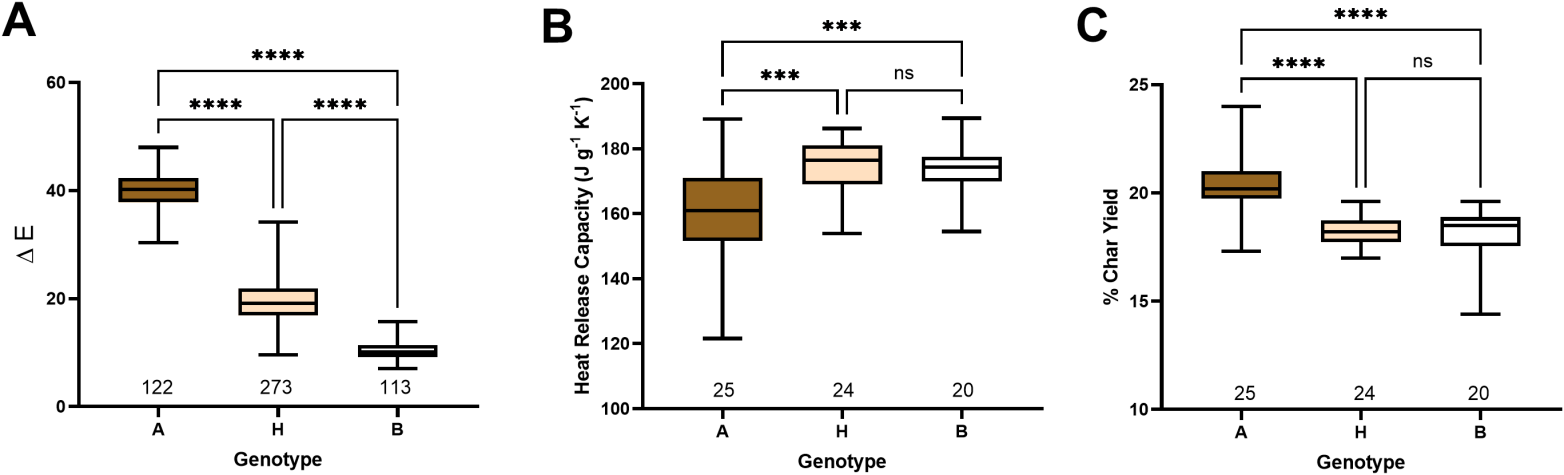
Color and FR segregation in the F_2_ population. **(A)** Box plot of CIELab color space measurements of segregating F_2_ population. Box plots of heat release capacity **(B)** and % of char yield **(C)** measured by microscale combustion calorimetry of selected individuals from the F_2_ population. The X-axis shows the number of samples in each group: A - brown, H – cream, and B – white colored cotton. Asterisks indicate the level of statistical significance, as determined using a one-way ANOVA multiple comparisons test with Tukey’s honest significant difference (Tukey, 1949). ***p-value < 0.001, ****p-value < 0.0001.

Mature fibers of each individual of the F_2_ segregating population were measured in the CIELab color space, and ΔE values were calculated (**Supplementary Data Sheet 1**). The samples were separated into three groups based on significantly (*p*< 0.0001) differentiated colors: brown, cream, and white (**Figure 2A**). FR of fibers from a randomly selected subset of samples (69) from each group and parents were tested with MCC (**Supplementary Data Sheet 2**).

MCC analysis revealed a similar heat release profile during combustion between SA-40 and TM-1 fibers (**Table 1**). However, F_2_ segregating samples showed a significant FR difference between brown and cream and white, but not between cream and white (**Figures 2B, C**). Quantitatively, fiber samples from the brown group yielded a lower heat release capacity (*p*<0.001) than the white or cream groups, indicating a slower, less intense combustion (**Figure 2B**). At the same time, the percentage of the sample not consumed or char yield was significantly (*p*<0.001) elevated in the brown group than in the cream or white, reflecting a higher level of inorganic material in the brown samples (**Figure 2C**).

While neither parent exhibited notable FR properties, the FR trait manifested in the F_2_ progeny only in the brown lint group of samples, indicating transgressive segregation of this trait (**Figure 2**, **Supplementary Data Sheet 1**). The transgressive segregation of the FR trait has also been reported in a multi-parent advanced generation intercross (MAGIC) population of white lint cotton, suggesting that FR is not necessarily linked to the colored lint trait (Thyssen et al., 2023). However, in this study, we observed significantly enhanced FR properties in only the brown-lint samples, indicating that multiple factors contributed to that trait. It has been proposed that the accumulation of proanthocyanidin pigments (condensed tannins) in brown fibers may chelate inorganic salts or otherwise alter the fiber’s chemistry to promote char formation, thereby reducing flammability (Hinchliffe et al., 2015, 2016; Nam et al., 2017, 2016). It is also possible that the unknown FR compound is synthesized in developing fibers and segregated by PA precursors via metal-flavonoid complexes (Hinchliffe et al., 2016). Therefore, the accumulation of brown pigments could enhance FR properties in the brown fiber F_2_ progeny plants.

### 3.3 Genetic mapping of the rust-brown trait locus

**Figure 3:**
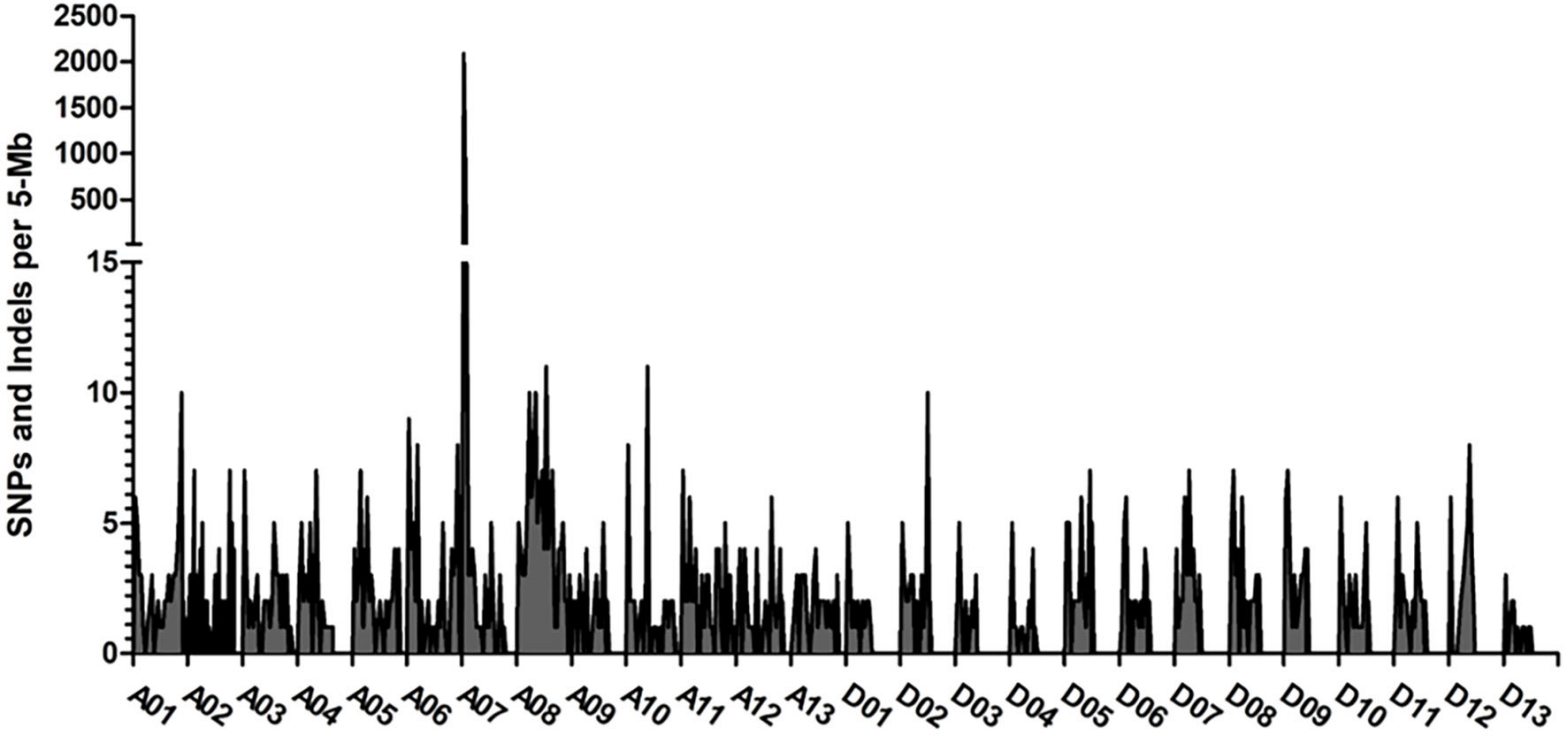
Distribution of sequence polymorphisms between the brown and white fiber bulked samples of the segregated F_2_ population. The frequencies of SNPs/Indels across the genome were obtained by the subtraction of the same variants between the bulks. The largest peak of different SNPs was observed at chromosome A07. Axis Y shows the number of variants per 5 Mb interval, and axis X shows the variants across the *G. hirsutum* genome.

BSA was utilized to develop markers for mapping the genetic locus associated with the rust-brown fiber trait. We created two pools of DNA from F_2_ segregating plants that exhibited contrasting phenotypes, as determined by CIELab color space. The first pool consisted of brown-colored cotton plants, while the second pool included white-colored cotton plants for the BSA. In the brown-colored bulk, each plant carries the mutation locus from the SA-40 genome, whereas the white-colored bulk contains plants with the TM-1 genomic region at the same locus. After performing genome-wide subtraction of the identical SNPs and Indels between the two bulks, we identified a significant peak with approximately 2,000 different variants on chromosome A07 (see **Figure 3**).

**Figure 4:**
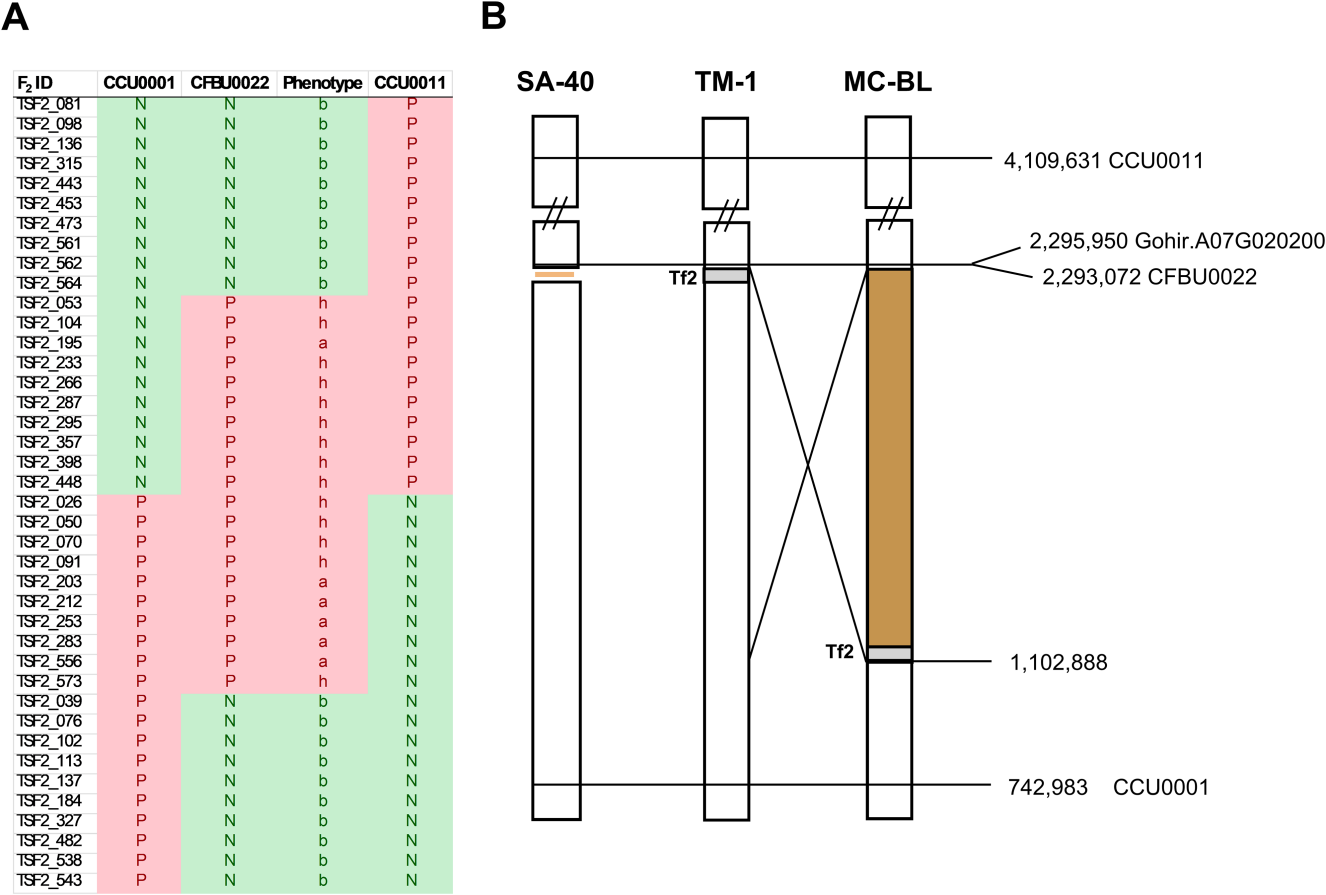
Physical maps of *Lc1* loci in SA-40, MC-BL, and TM-1. **(A)** Recombinant plants from the F_2_ population. PCR was performed with SNP primers specific to the mutant (rust-brown) genotype; therefore, the result is shown as ‘N’ negative or ‘P’ positive. **(B)** The physical distance between markers on chromosome A07, according to the JGIv1 TM-1 reference genome assembly, is shown in base pairs. Positions and names of the markers are displayed on the right side of the diagram. The diagram shows Tf2, an LTR retrotransposon in TM-1, which is absent in SA-40 and has been relocated to 1.4 million base pairs away from *Gh_TT2_A07* (Gohir.A07G02200) gene due to a structural rearrangement in the MC-BL line.

The bin size in the frequency plot shown in **Figure 3** is 5 MB. We hypothesized that the genomic region associated with this peak might harbor a locus for the rust-brown trait; therefore, we developed SNP markers within this interval (see **Supplementary Table 1**). Analysis of the SNP markers in the segregating F_2_ population revealed that the brown-rust locus was flanked by SNP markers CC0001 and CCU0011, spanning a genomic region of approximately 3.37 million base pairs (**Figure 4**). The marker CFBU0022 completely co-segregated with the rust-brown phenotype **Supplementary Data Sheet 1**). **Figure 4A** displays 40 recombinant plants from the mapping population, with the SNP marker CFBU0022 showing positive PCR results only for the samples exhibiting brown (a) or cream (h) fiber phenotypes.

Marker CFBU0022 is positioned 2.9 kb from the start codon of the *GhTT2-A07*, making this gene a leading candidate within the mapped interval. The mapped genetic locus of the SA-40 rust-brown trait coincides with the previously well-characterized *Lc1* genetic locus for natural brown cotton (Hinchliffe et al., 2016; Kohel, 1985; Li et al., 2012; Wen et al., 2018). To investigate allelic differences at *GhTT2_A07*, we compared the genomic sequence of this gene and its adjacent regions from SA-40 and TM-1. Notably, SA-40 misses a ~10.7-kb sequence in the upstream regulatory region of *GhTT2_A07* compared to TM-1. This absent sequence harbors a retrotransposon, which is present in the reference TM-1 genome approximately at 7.5 kb upstream of the *GhTT2_A07* coding sequence.

Blast search of the retrotransposon sequence showed the highest similarity (93% nucleotide identity) to predicted *Gossypium raimondii* transposon Tf2–1 polyprotein (XM_052634828.1). The structure of this retrotransposon in the *G. hirsutum* TM-1 consists of two mostly identical long terminal repeats (LTRs) with 5’LTR 2877 bp and 3’LTR 2879 bp, and three predicted ORFs in the polyprotein, all together 4687 bp (**Supplementary Figure 1**). The Tf2 retrotransposon belongs to the class of LTR retrotransposons of the Ty3/Gypsy family of the *Metaviridae* group. It is a well-studied element in the genome of *Schizosaccharomyces pombe* (Cui and Guo, 2022; Jia et al., 2023). Ty3-like LTR retrotransposons proliferate in plants through a “copy-and-paste” mechanism (Kumar and Bennetzen, 1999). They generally leave recognizable signatures when they move, such as target site duplications or residual LTR sequences; however, under certain circumstances, such as homologous recombination or large structural rearrangements, the LTR retrotransposon can be removed without leaving a trace (Kumar and Bennetzen, 1999; Neumann et al., 2019).

**Figure 5:**
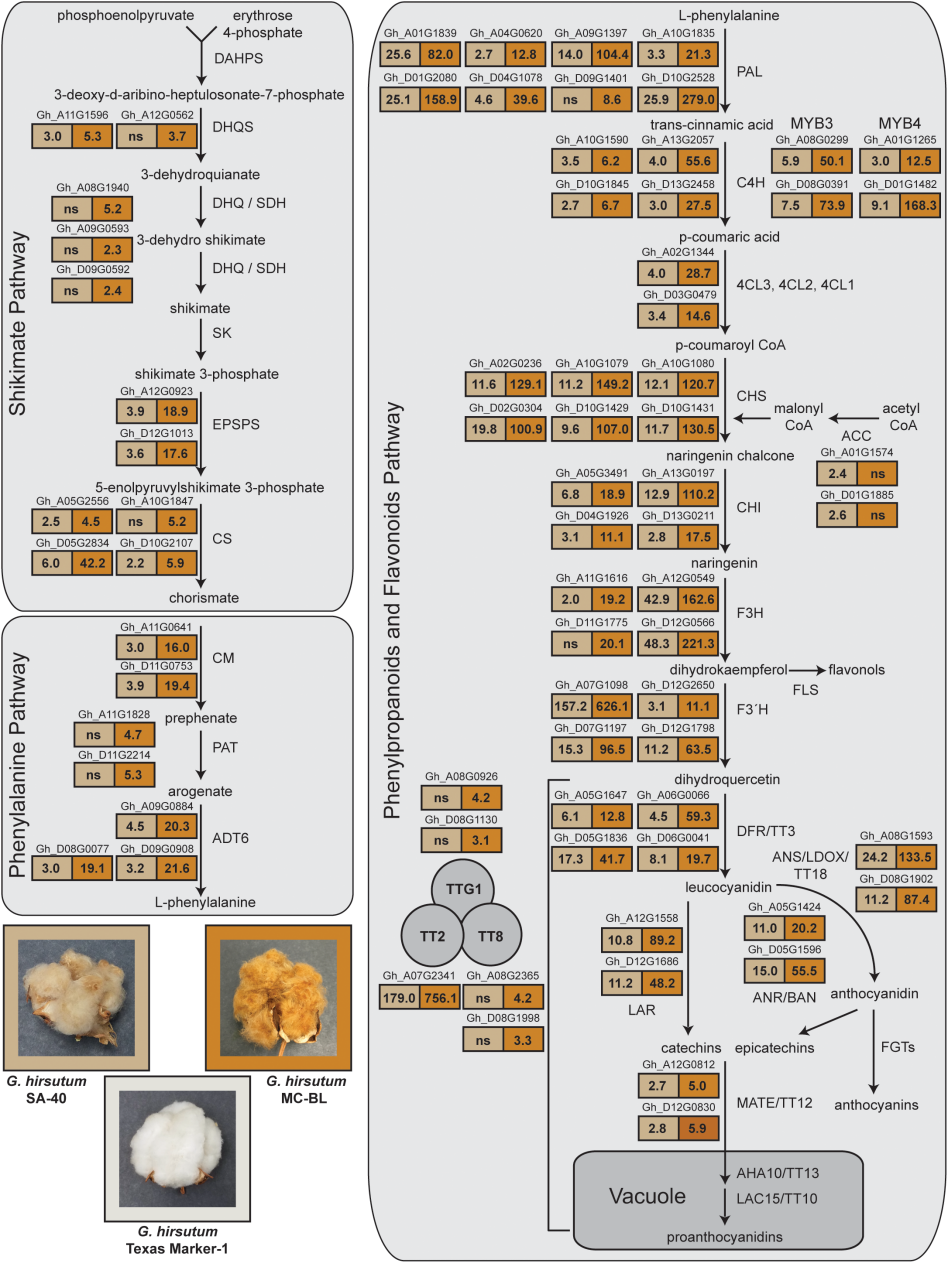
RNAseq analysis of gene expression from shikimate, phenylalanine, phenylpropanoid, and flavonoid pathways. RNA was evaluated from developing cotton fibers at 10 DPA. Images of open bolls from the SA-40, MC-BL, and TM-1 lines are shown in the bottom left corner of the figure. The boxes in the figure display the fold change values of gene expression in comparisons between SA-40 and TM-1, as well as between MC-BL and TM-1. The boxes are filled with two colors: light brown for SA-40 and brown for MC-BL, reflecting the hues of the cotton fibers from these lines. Significantly upregulated genes (more than 2-fold, DRF < 0.05) are indicated above the boxes displaying the fold change; gene names are provided from the *G. hirsutum* TM-1 genome (Zhang et al., 2015). See also <xr rid="sf5">Supplemental Data 3</xr> for details of ANOVA analysis of RNAseq data.

We observed that the structural rearrangement in the MC-BL line relocated the Tf2 retrotransposon 1.4 million base pairs away from the *GhTT2_A07*, resulting in upregulation of this gene (**Figures 4**, **5**). However, careful evaluation of the *GhTT2_A07* promoter region in the SA-40 line revealed that no signatures remain from the Tf2 retrotransposon. Retrotransposons are known to spread epigenetic silencing to neighboring regions (Eichten et al., 2012). We speculate that in the SA-40 line, the absence of the Tf2 retrotransposon upstream of the *GhTT2_A07* gene does not inhibit its transcriptional activity, resulting in the production of rust-brown fiber. In contrast, the presence of the retrotransposon insertion upstream of the *GhTT2_A07* gene in the TM-1 line reduces the activity of *GhTT2_A07*, leading to decreased production of PAs and, consequently, no brown coloration. To support our hypothesis, we tested 132 white cotton accessions (Thyssen et al., 2025) for the presence of the Tf2 retrotransposon in the promoter region of the *GhTT2_A07*. All tested white cotton cultivars contained the Tf2 retrotransposon upstream of the *GhTT2_A07* (**Supplementary Data Sheet 3**).

### 3.4 Absence of retrotransposon upstream of the *GhTT2_A07* gene corresponds with its higher transcriptional activity

TM-1, a white cotton, harbors the Tf2 retrotransposon upstream of the *GhTT2_A07* gene, while SA-40, a rust-brown fiber variety, does not. In MC-BL, a dark brown cotton line, the Tf2 retrotransposon was relocated 1.4 million bp away. To evaluate the transcript activity of genes involved in the proanthocyanidin (PA) pathway, we performed comparative RNAseq analysis of developing fiber samples at 10 DPA between SA-40/TM-1 and MC-BL/TM-1. TM-1 was selected as the baseline for comparative analysis since *GhTT2_A07* expression was lowest among the cotton lines.

Analysis of Variance (ANOVA) in gene expression between SA-40 and TM-1 detected 1,770 genes that were significantly (FDR<0.05, two-fold) differently expressed (DEGs), while between MC-BL and TM-1 identified 14,898 DEGs, which is eight times more (**Supplementary Data Sheet 4**). Among those genes, we found 73 upregulated genes annotated as part of the shikimate, phenylalanine, phenylpropanoid, and flavonoid pathways (**Figure 5**). *GhTT2_A07*, a part of the MBW complex, was upregulated 179-fold in SA-40 and 756-fold in MC-BL compared to TM-1. At the same time, two copies annotated as TTG1 and two copies of the TT8 genes were upregulated 3–4 times only in MC-BL (**Figure 5**).

Upregulation of the *GhTT2_A07* gene resulted in coordinated increases in the expression of genes involved in phenylpropanoid and flavonoid pathways, as well as their precursors of shikimate and phenylalanine biosynthesis. Different intensity upregulation of the *GhTT2_A07* in SA-40 and MC-BL proportionally activated the transcriptional activity in the cohorts of the genes affected by TT2 (**Figure 5**). Genes involved in the final steps of PA biosynthesis, including anthocyanidin synthase (ANS), anthocyanidin reductase (ANR), and leucoanthocyanidin reductase (LAR), as well as vacuole transporters MATE/TT12, were proportionally upregulated in both lines. The expression data agreed with the visual phenotype: the higher *GhTT2_A07* expressed, the darker brown fiber coloration developed in the cotton lines.

## 4 Discussion

### 4.1 Retrotransposon upstream of *GhTT2_A07*

The primary objective of this study was to determine the genetic basis of the rust-brown fiber coloration in the cotton line SA-40. The mapping of the F_2_ segregating population of 508 individuals showed an association of the rust-brown phenotype with the *GhTT2* gene on chromosome A07 (**Figure 4**), which coincides with the *Lc1* locus (Hinchliffe et al., 2016). Comparative analysis of the upstream region of *GhTT2_A07* between TM-1 and SA-40 revealed a missing sequence of the Ty3-like LTR retrotransposon in the SA-40 line. We do not think that the SA-40 line lost the LTR retrotransposon since there were no traces of it left (Kumar and Bennetzen, 1999). We speculate that the white cotton variety obtained a copy of the LTR retrotransposon upstream of *GhTT2_A07* during evolution and was preferentially selected during cotton domestication due to agronomical advantages.

The occurrence of the Ty3-like LTR retrotransposon upstream *GhTT2_A07* in TM-1 corresponds with lower transcriptional activity of this gene and, consequently, colorless (white) cotton fibers. *GhTT2_A07* is an R2R3 MYB transcription factor named for its homology to the Arabidopsis *TRANSPARENT TESTA 2* (*TT2*) gene. *TT2* in Arabidopsis is a well-characterized regulator of PA (condensed tannin) biosynthesis in seed coats (Nesi et al., 2001). The loss or gain of transposable element (TE) insertions can markedly influence nearby gene expression by disrupting cis-regulatory sequences, providing novel regulatory information, or altering chromatin state (Hirsch and Springer, 2017). For example, an LTR retrotransposon insertion upstream of the *MdMYB1* gene, a known activator of the anthocyanin pathway, was linked to the red fruit color of apples (Zhang et al., 2019). In *Capsella rubella*, a TE insertion at the 3’ UTR of *FLOWERING LOCUS C* reduced its expression level, which promoted flowering (Niu et al., 2019). TE-mediated silencing of important metabolic genes has precedent in cotton; for example, a T-DNA inserted into a retrotransposon upstream of a flavonol O-methyltransferase (*GhOMT1*) led to suppressed *GhOMT1* activity and concomitant over-accumulation of anthocyanin pigments in fiber (Ke et al., 2022).

TEs’ influence on adjacent gene expression by changing their chromatin state is well documented (Hirsch and Springer, 2017). It is known that the majority of TEs are epigenetically silenced and that this silencing can spread to adjoining sequences. A study in maize surveyed multiple retrotransposon families and found that some families can spread heterochromatin marks to nearby genes, lowering their expression levels (Eichten et al., 2012). In yellow mustard, retrotransposon insertion changed the chromatin state of the adjacent *FAE1* gene and reduced its expression (Zeng and Cheng, 2014).

These examples underscore how TE insertions or excisions can act as cis-regulatory toggles, creating heritable variations that influence biochemical pathways. From an evolutionary standpoint, the LTR retrotransposon upstream of *GhTT2_A07* may have been acquired in certain cotton lineages and preferred during human selection due to longer, stronger fiber and higher yield. NCCs were historically abundant (Crawford, 1924; Ware, 1932), but white fiber became dominant, especially in recent years, likely because it was easier to dye and had better fiber quality (Lee and Fang, 2015). The retrotransposon insertion upstream of *GhTT2_A07* that turned off the PA pathway would confer a selective advantage under domestication by yielding desirable white fiber.

The finding in the current study closely parallels the previously characterized *Lc1* locus, which identified a 1.4 Mb inversion upstream of the *GhTT2_A07* gene in the MC-BL dark brown fiber line (Hinchliffe et al., 2016). The 1.4 Mb inversion removed the retrotransposon away from the *GhTT2_A07* gene; however, in that case, the absence of the retrotransposon not only returned the expression level to the original state as in SA-40 but increased it even higher, probably due to new regulatory elements brought with the new sequence (**Figure 4**). Elevated expression of *GhTT2_A07* triggers up-regulation of downstream flavonoid biosynthetic genes, ultimately driving the accumulation of PAs (condensed tannins) that impart a brown hue to the mature fibers (Yan et al., 2018). Notably, the *GhTT2_A07* up-regulation in SA-40 is quantitatively lower than that reported for the *Lc1* inversion allele, correlating with the lighter “rust-brown” color of SA-40 fibers compared to the dark brown MC-BL phenotype.

The current study found that the gradation in color intensity depends on the dosage of expression of the *GhTT2_A07*. However, other mechanisms of color shade manipulation have also been reported. Two epistatic QTLs found on chromosome A07, one corresponding to *TT2* (*qBF-A07-1*) and another to *TTG1* (*qBF-A07-2*), govern the shades of brown fiber (Wen et al., 2018). Their study is consistent with our observation: *TT2* and *TTG1* are both part of the MBW complex, where *TT2* initiates the PAs production, while *TTG1* affects the processing activity of *TT2*. Together, our results confirmed the *GhTT2_A07* as a key regulator of cotton fiber pigmentation and demonstrated that retrotransposon activity can modulate the PA pathway to produce different shades of brown cotton fibers.

### 4.2 FR phenotype

The SA-40 fibers have no significant difference in FR properties compared to TM-1 fibers (**Table 1**); however, fibers of their F_2_ brown-colored progeny (group A) showed significantly enhanced FR (**Figure 2**). These results suggest that a new combination of alleles of genes occurred in the progeny that resulted in the FR trait being superior to that of its parents; this phenomenon is known as transgressive segregation. A similar observation has also been reported for the FR trait in the MAGIC population of white lint cotton, where four lines exhibited significant FR properties. The flammability test under standard 45˚ incline for fabrics produced from fibers of these four lines showed self-extinguishing properties (Thyssen et al., 2023).

Thyssen et al. (2023) suggested that FR is not a necessary attribute of brown fibers since white fibers can be flame retardant as well. The authors speculated that the colorless flavonoids accumulated in white cotton lines may share the ring structure that has been proposed to contribute to the FR properties of tannins (Nam et al., 2017). In the current study, significantly enhanced FR properties were detected only in the brown colored group samples that accumulated a higher amount of PA pigments. Therefore, other factors can be considered, such as: the accumulation of PAs (condensed tannins) in brown fibers may chelate inorganic salts as well as unknown FR compounds that can alter the fiber’s chemistry to promote char formation, and reduce flammability (Hinchliffe et al., 2015, 2016; Nam et al., 2017, 2016; Naoumkina et al., 2024).

In the previously reported MC-BL line, dark brown fibers accumulated higher levels of sodium, potassium, and other inorganic elements (Hinchliffe et al., 2016). The authors also observed that the FR effect in MC-BL manifests early in fiber development, well before brown pigmentation is visible. In SA-40, a similar biochemical scenario is likely at play. Our RNA-seq data confirmed the activation of the PA pathway in developing SA-40 fibers, presumably leading to the accumulation of flavanol units (e.g., catechin, epicatechin) and their oligomers. Although we did not specifically isolate the responsible metabolite, it is reasonable to infer that partially polymerized PAs or their precursors endow lint of brown-fiber segregants with improved flame retardancy. Supporting this idea, simple water-based processing can diminish the FR advantage of brown cotton by extracting water-soluble tannins (Hinchliffe et al., 2015; Nam et al., 2016), demonstrating that the flame retardant component is leachable and likely composed of lower-molecular-weight polyphenols.

Future metabolomic analyses of SA-40 fibers at progressive developmental stages could help pinpoint the specific compounds responsible for flame retardancy, which has implications for both plant biology and textile science. Identifying the active FR metabolite(s) may facilitate the use of natural fiber chemistry to reduce reliance on added flame-proofing chemicals in cotton products. In summary, our findings illustrate how structural genomic variation at a single regulatory locus can drive complex trait outcomes (color and FR) and how understanding these mechanisms enables targeted breeding for multi-functional crop products.

## 5 Conclusion

In this work, we revealed that the absence of Ty3-like LTR retrotransposon insertion upstream of the *GhTT2_A07* is linked to the rust-brown fiber phenotype in the SA-40 line. All of the tested 132 white fiber accessions harbored the retrotransposon insertion upstream of the *GhTT2_A07*. The SA-40 line, where the retrotransposon insertion did not occur, has a higher transcriptional activity of the *GhTT2_A07* and produces a brown-rust fiber. In the dark brown fiber MC-BL line, where a large structural inversion has removed the retrotransposon, the activity of the *GhTT2_A07* was detected at a much higher degree. The study findings suggest that the expression level of *GhTT2_A07* directly controls the value of brown coloration in *Lc1* fibers; the higher expression produces darker fibers.

Understanding the regulation of cotton fiber pigmentation and its connection to FR has practical implications for cotton improvement. It provides a way to naturally infuse fibers with desirable qualities, supporting initiatives to create sustainable textiles. Future work will focus on deploying this allele in breeding and perhaps isolating the specific metabolites responsible for FR. Ultimately, the knowledge gained from mutations in the *Lc1* locus reinforces the concept that modulating key regulatory nodes in the flavonoid pathway can yield a spectrum of beneficial traits – from aesthetic fiber colors to enhanced safety – thereby pushing the boundaries of what natural plant fibers can achieve.

## Data Availability

The RNAseq data were submitted to the NCBI-SRA under the BioProject number PRJNA1291244.

## Supplementary material

Supplementary Figure 1 Map of Tf2 LTR Retrotransposon.

Supplementary Table 1 SNP genetic markers for mapping.
