# Supplementary material for: The brown fiber phenotype in cotton line SA-40 is linked to a missing Ty3-like retrotransposon upstream of the GhTT2_A07
Source: Front Plant Sci. 2025 Sep 3;16:1668965. doi: 10.3389/fpls.2025.1668965 (PMC12441163; doi:10.3389/fpls.2025.1668965)
Supplement: Supplementary Figure 1 — Map of Tf2 LTR Retrotransposon. [file Supplementaryfile1.docx]

Supplementary Material

# Supplementary Data

Supplemental Data 1. MCC measurements of fiber samples from individuals of the F_2_ mapping population.

Supplemental Data 2. FASTA file of Ty3-like LTR retrotransposon sequences from 132 cotton accessions.

Supplemental Data 3. ANOVA analysis of RNAseq data.

# Supplementary Figures and Tables

## Supplementary Figure


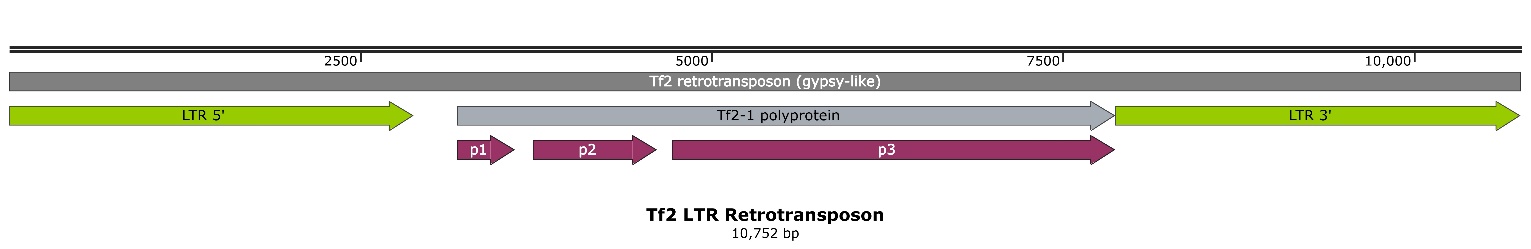


**Supplementary Figure 1.** Map of Tf2 LTR Retrotransposon. The image was created with SnapGene v. 6.2.2 software.

## Supplementary Table

## Table S1. SNP genetic markers for mapping.

|  | | 5’ Primer Sequence | | | | |  |
| --- | --- | --- | --- | --- | --- | --- | --- |
| Primer ID | | Forward |  | | Reverse | | Tm, ^o^C |
| CFBU0001 | TGAAAGGCGGTTCTTTAGT | | |  | | ATGTCCGAGATTGATTTAGATATG | 60.6 |
| CFBU0011 | CTTGAATATCAAATGGGTTTGCA | | |  | | CTGAATTTTCTTTGTTTTGTCATT | 61.0 |
| CFBU0022 | TTTGTATAATGAAAATATAACAGCTTATC | | |  | | GGACCAATGAAATTAGGAAACA | 63.3 |

Note: Primer pairs are specific to the brown-rust mutant genotype.

## Table S2. Quality control data of RNAseq samples.
