# Supplementary material for: The brown fiber phenotype in cotton line SA-40 is linked to a missing Ty3-like retrotransposon upstream of the GhTT2_A07
Source: Front Plant Sci. 2025 Sep 3;16:1668965. doi: 10.3389/fpls.2025.1668965 (PMC12441163; doi:10.3389/fpls.2025.1668965)
Supplement: Supplementary file 4 [file Table3.docx]

**Supplemental Data 3.** LTR retrotransposon sequences from 132 cotton accessions.

>Acala1517-80

AGGTAATGACCCAAAATTCATGGGCATCGGAAAAGTATAATATCGGGCCTCCGTCCTAGT

AAATTGAGTCCGAAAATAATTATTAGAAATATTTACGAGACTAGTAGTGTGTTTAATTAG

GTTTTAATTAAGTAAATTTAGCTTAATTTAGAGTAATTAGTAAAAAGGATTAAATTGAAT

AAGAGTAAAAGTTTAATTATAGATTAAAGGAAAATAATAGGGACCAAATGGGCAATTAAG

CCACATTTGGAAGTTGAGGCGGCATAACATTGTAAAAATCTTAGATTTTTATATTATTAT

TTATATAAATATATAAATTAATTATAAAGTATATTATTAAATTAATTATATTATAAATAT

TATATTATTATATATAAAAGAAACAAAACAGAAAAGAAACAGAATAGAAAGAACAAAGAA

ACAGAATAGAAGAGACGAAACAGGGGAGAAGCAGGGGAGAAAGAAGAAAAAGAAGAAAAA

AGGGGAAATAGGGTTTTTGAAGCTTGAAATTTAAATTGGTAAGTCAAATTAGCCATTTTC

TCTTAATTCTAATGTTTTAAAAGCTTTAAAACAAAGTTTTGATGGAATTAAGTTGATATT

TTGTAAGTTCATAGGTTTTCAAGTATAGTTTATGTTGAACAAAAGAGATGAATTAGGGAT

TAACTTGAAGGAATTTTAAGTTAGAATTGAAAAAGGGATTAAATTGTAAAAGAAACTATA

AGTTTTTTTTGTTTTAGGGACTAGATTGAGGAAAATTCGGAATTAAGAAAATATGTTAAA

AATTTAATAGTTAAATTTGAGTTTAAATGAAATTTGAATAGGAATAAGGTGTGAATTGGT

GTTATAAATTTGGTTATTAACATTTTTAATCAAAACAGTTTTGGGAAGTAGCAATGGTCT

GACTTTGAAAATTCACTAAAAATTTTATAAATTGAACTAGAGGATGAACAAAATATGGAA

TTAAAGCTTATTGAGTCTAGTTTCTTATAGTAGAAACAATGTAAGCAATTAATTGATGAA

TCAAGAGATATTTGAAATTTTGTAATACTGGTTCGGGGTGATTTCGAGATGCCCTGTTTT

AACTTTGGAAAATCATTAAAAATTGTACAAAAATTATTATGGAGTGTAATTTATATATGT

GAACTCCTTAATGAATCTAGTTTCAAAATAAATAAACAAGAACCTTATTCGAGTTCTGTA

CAATGAGATAATTTAGTTTTAGTGGAGAGAGGTCAGAACTGTCAAATGAAATAACAGGGG

AGTATTTAACGAATAAACTGTATTAAATGGCTAGACCAAAAATTCTGGAAATTTTATGAT

TAGAAGATATATGAGTCTAGTTTTAAGGAAAATTTACGGATATTAATTTGGAGTTTCGTA

GCTCAAGATATAAATAATTTAGTAACAATGACCCAAGTAGACAGCTTAATGGTGAAATTA

TATAAATACATTAAAAATGGTTAAATTTGCATGTTTAGGCTCATGAATTAAATTGAATCA

TGTTGTATTGATTATTATAAATTATTATTTTCGTAGCCAACAAAGAACCTAAAGCATCAG

CATCGAAAGGAAAGGAGAAAGTCATCGAGGAGTAAACTCGAGAAAATTACGGTTTGTATT

ACTATAATTCAAGTTATTTATTATTAAATGTTAAATTTTAATTTATGTGTCTAGTAAATG

AAATGTGAGGTAAGTATTATTATTATTATTATTATTATTATTATTATTATTATTATTATT

ATGAGTGGGAATTAAATTGAATAGTTGATATGAAATAATATTTGAATTGTTTGTTGATTG

AAAGCGGGAAATGAATTTAAATCGAATAGTGACCGATATTAAATTGAATGGAAATGTATT

GAGTTGTGAAAATATGTTAATTGCGGATTAATTATTGATTGAAAGGTGGAAAAATGATTG

AATTGAAAGTGTGAGAAAGTGTGATTGAATTGGGATTATATGTGATTTAAATACCCTATT

AACTAGTCGGGCTGAGTCGGATATAGTTGGCATGCCATAGGATTGGAAGAGTTCAGGGAT

ACTTCGACCTCGAGTCGATGAGACACTGGGTGATTTCTTCGGATAGATTGGATGAGGTAC

TGGGTACCAACTTTCTTCGGCTTTGCCGATGAGACACTGGGTGTCAACTATTGCTTCGAA

CTATCCGATGAGGCACTGGGTGCCATTCTGGTGTGTTTGGTTGGATCCGTGTATCCGCCA

AAGTCCGAGTTTTGTTAATAGGGTAAATGATGAAATGATAAACCGAACGAGTTGGTCAAA

CGAGCTATTGAAATGATATGAAAAAGTTGAATTGTGAATTGAAATGTGAAATGAGATTGA

GAAATGAACCTAAGGTTCGTGAATTATTCAAACTCAAATTGTGGATATACGATATTGGTT

GATGAATTGCTATTGTTGAAATATTTAATTTAAATTGTATATACGATTTATGCTTTACAT

GTACATTATTGTTATAATTTGAATTATGGTAATACCACTGAGTATGAATTACTCAGCGTA

CGGTTGTTTCCGTGCGCAGGTCAATAGAAGTCAAAGGTCTCGGTTCAGCATCCAGATTAA

TCCCGGCTTCGGCAAAACTTGGTGATGTATTTTTCCTTTGGTAAAGGTGGCATGTACATA

GATTGTGTATAAAGGTTATTATGTTTTATTATATAATGGTTAAAAATGTTAGTATTAAAA

GTTTATGGATTTTAATGAAAGAAGTCTATCTATTTTATCTAATTAGTACATTGTTAAATT

TTAAATTGGTATTGTGTAGATTGAGTTTGATTAGAAGTATTTAGAATAGAAAATGTGAAT

GTGAAATGAATTGGTTGAATTGATGATATTTGGGAACTATATGGTTTTAATTTGCAGGGG

GTTTTATGTAAAAATAAGCAGAAATGCTGCCGAAATTTTTATAAAAAAAAATGAAGTCAT

TTGGTAAACAAATTAATAAATTTTATGAATTATTTTAATATATTGGTTATTTATTTAAGA

ATTGTTGTAAATCGTTCGATACGTCCGGTAGTGCCTCGTAATTCTGTTCCGGCGACGGTT

CGGGGTTAAGGGGTGTTACATTTTATGGTATCAGAGCTATCAGGTTTAGCCGATTCTCGG

CCTAAATCGAGCTCGGAATTGAGTCTAGATGTACATGCCACTGTCGAGTTAAACTGAGTC

GGGATTTTTGGATGCTGACCTATTTGTTTGTTTTGTTTTATAGATTAAAGATGTCTGAAG

AAAGAATAAATGATACTGATGAAAGAATGTATAGTGAAGATAGAGAATTAGATGAAACAG

AATCTGTTGCACCGAGTGTGAATCCGTTAGGCAACCAACCTTCTAATGTAGAACGAGAAA

ATGTCAGAGATAGAGATGAATCCCAATTACTGAGAATTATAGCTGATGCATTACAAAGAG

TAGCAGGAACTACTCCTGTTACGACTTCAGTACCTACTGTTAGACGGGCTCCGATAAAGG

AACTGAGGAAATATGGTGCCACTGAATTTATGGGTCTAAAAGGAGTTGATCCATCCATAG

CTGAAAATTGGATGGAGTCGACTAAAAGAATTTTGCAGCAATTGGATTGTACCCCCCGAG

AGTGTTTAATCTGTGCCGTATCGTTATTACAAGGGGAGGCTTATCTATGGTGGGAATCAG

TGGTTCGACATTTACCAGAGAGTCAGATAACGTGGGATCTATTTCAGAAGGAGTTTCAAA

AGAAATATATCGGAGAGATGTATATTGAAGACAAGAAACAAGAGTTTTTGTTGCTACAAC

AGGGTGATATGTCAGTAATAGATTATGAGAGGGAATTCTCGAGACTCAGTAGATATGCCT

CCGAGTTTATTCCGACAGAAGCCGATAGTTGTAAAAGATTTTTACGGGGTTTACGAGACG

AGATCAAAGTGCAGCTAGTATCCCATCGGATCACTGAGTTAGTAGATTTGATTGAACGAG

CTAAAATGGTGGAACAAGTTCTGGGCCTCGACAAAAAGACTGAAGTTGTTAGACCAACCG

GGAAGCGTACAGGAACTACCAGTTCGAATCCTCAGCCGAAAAGACCAAAGGAATTCCAAA

GTGGTTGGAGATCCAGTTTCAGGTCAGACAGAGGTGGTAGAAATAGGGGAAAACAGACGA

TGACATCTACTGGCAGTGTGAAAGGTCCTTCCCGAGAAATAGATATTCCAGACTGCCAAC

ACTGCGGAAAGAAACACAGAGGGGAATGTTGGAAATTAACTAGAGGCTGTTTTCGATGTG

GTTCTACAGACCATTTCATCAGAGACTGTCCGAAAGTTGATAGTACTGTACCCGTGACAT

CACAGAGATCGGTATCTACAGCTAGAGGCAGAGGGTTAGGAAGAGGTGGTTCGGTTTCAA

GGGGAGGAAGTATTAGGAGAAGCAATGATATTGCTACTCAGCAGTCTGAGGCTAAAGTAC

CTGCCAGAGCTTATGTGGTCAGAACACAGGAAGAAGGTGACGCCCACGATGTAGTAACAG

GTATATTCTTACTATATTCTGAGCCTGTTTATGCTTTAATTGATCCCGGATCTTCACATT

CTTATATAAATTCAAAATTAGTTGAATTGGGAAAATTTAATTCTGAAATATCTAGAGTGA

CTGTAGAAGTGTCGAGTCCGTTGGGGCAAACAGTATTAGTGAATCAGATCTGTCCGAGAT

GCCCGTTAATTATACAAAATAAAACTTTTCCTATTGACCTGTTGATTATGCCATTTGGAG

ATTTTGATATAATACTGGGGATGGATTGGTTGGCTGAGCACGGAGTGGTATTGGATTGTT

ATAAAAAGAAGTTTAGTATTCAGACAGAAGACGGGGACAGAATTGAAGTAAATGGTATCC

GTACTAATGGGCCGACACGTATTATTTCGGCAATAAAGGCTAATAAATTGCTTCAGCGGG

GTTGTACAGCGTATTTAGCCTATGTTATTAATTCTGATTTGGTTGGTAGTCAGTGCAGTA

AGATTAGAACCGTATGTGAGTTTCCAGATGTATTTCCTGAAGAGCTACCGGGTTTACCAC

CTGACAGAGAGGTTGAATTTGCTATAGAAGTGTATCCGGGTACAGCACCAATCTCTATAC

CACCGTATCGAATGTCACCCACTGAGTTGAAAGAGTTGAAAGTGCAGTTACAGGACTTGT

CAGATCGTGGATTTATTAGACCGAGCATCTCACCTTGGGGAGCTCCAGTATTGTTTGTTA

AAAAGAAAGATGGATCGATGCGGCTTTGTATTGATTACCGGCAGTTAAACAAAGTGACGA

TCAAGAACCGGTATCCGTTACCCCGTATAGATGATTTATTTGATCAACTAAAAGGAGCTT

CAGTATTTTCAAAGATTGACTTAAGATCTGGGTATTATCAGCTGAAGGTAAAAGAAAGTG

ATGTTCCGAAGACTGCATTTCGTACTCGATATGGTCATTATGAATTTTTGGTGATGCCGT

TCGGGTTGACTAATGCTCCAGCTGCTTTTATGGATCTGATGAATCGTATTTTTCAGCCGT

ATTTAGATCAGTTTGTGGTGGTTTTTATTGATGACATCTTGGTTTATTCGAAGTCAGAGT

CAGAGCATGATCAGCATCTCAGAACCGTGCTACAAATTCTGCGAGAAAAACAGTTGTACG

GGAAACTAAGTAAATGTGAATTCTGGTTATCAGAGGTAGTATTCTTGGGACATGTTGTAT

CTGCGGATGGGATTAGAGTTGATCCGAAGAAGATCGAGGCAATTGTTCAATGGAAGGCAC

CAAAGAATGTATCAGAGGTACGCAGTTTTCTTGGTTTGGCTGGGTATTACAGAAGATTTG

TAAATGGGTTTTCGAAGATAGCTTTGCCGATGACCAAATTACTACAGAAGAATGTTCCAT

TTATCTGGGATGATCAGTGTCAGAGGAGCTTTGAAACATTGAAACAGATGTTGACAGAGG

CACCAGTTTTAACTTTACCAGAATCAGGGAAAGATTTCATAGTGTACAGTGATGCTTCTT

TGAATGGTTTGGGTTGTGTATTGATGCAAGAAGGAAAAGTAATAGCTTATGCATCTCGAC

AGTTGAAGTCACATGAACGCAACTACCCGACACACGATTTAGAGTTAGCTGCTGTAATCT

TTGCATTGAAGATTTGGATACATTACTTGTATGGTGAGAAATGTTATATTTACACTGATC

ATAAAAGTCTAAAATATCTTCTGTCACAAAAGGAGTTGAATCTGAGACAGAGACGGTGGA

TTGAACTTCTGAAAGATTATGATTGTGTTATAGATTATCATCCAGGGAAGGCAAATGTGG

TAGCAGATGCATTGAGTAGAAAAGCAGCGATTGAATTACGAGCAATGTTCGCTCGACTTA

GTATTAAGGATGATGGAAGTTTGTTAGCTGAGTTAAGAGTCAAGCCGGTGATGTTTGATC

AAATCAGAGCAGCACAGTTAAAAGATGAAAAGTTGATGAGGAAAAGAGAAATGGTACAGT

ATGGTGCGGTAGAAAATTTTAGTATTGACGAGCATGATTGTTTGAGATTTCGAAATCGAA

TTTGTGTTCCATCTACTTCTGAGATTAAAGAATTGATTCTCCGAGAAGCACATAATAGTA

TTTTTGCTTTGCACCCAGGAGGAACGAAGATGTATCGTGATCTACGAGAACTGTATTGGT

GGCCAGGAATGAAGAAAGATATAGTTGAATATGTCAGTAAATGCTTGACTTGTCAGCGGG

TAAAAGCAGAACATCAGGTACCAACAGGCCTGTTACAGCCTATTACTATTCCCGAGTGGA

AATGGGATCGCATTACCATGGATTTTGTTACGGGGTTGCCATTGTCAGTGAGTAAAAAGA

ATGCTATTTGGGTGATTGTTGATCGACTCACAAAATCAGCTCATTTTATAGCAGTTAGAA

CCGACTGGTCATTACAGAAGCTTGCCGAGGTTTATATTCGAGAAATTGTTAGATTACATG

GTATTCCGGTATCAATAATTTCAGACAGAGATCCTCGATTCACTTCGAGATTTTGGAAGC

AGCTGCATGAATCATTGGGTACTCGACTTAGTTTCAGTACAGCTTTTCATCCTCAAACTG

ATGGACAATCTGAACGAGTAATTCAGATATTAGAAGATATGCTTCGAGCTTGTGTCATTG

ATTTTGAATCAGGTTGGGAACGTTATTTACCATTGGCCGAGTTTGTTTATAATAATAGTT

TCCAATCTAGTATTCAAATGGCTCCATATGAAGCACTTTATGGTCGAAGGTGTCGATCAC

CAATATGTTGGACAAAATTAAGAGAAAGAAAAGTGATTGGGCCGGAATTGATTCAAGAGA

CAGAAGAAACAGTTAAAAAGATTAAAGATAGACTGAAAGCCGCTTTCGACAGACAGAAAT

CTTACGCAGACTTGAAACGACGAGACATTGAATATTCCGTTGGTGATAAGGTATTCCTCA

AAGTATCGCCGTGGAAGAAAATTTTGAGATTTGGTCGGAAGGGAAAATTAAGTCCGCGCT

TTATTGGGCCGTATGAGATAGTGGAAAGAATTGGGCCTGTTGCTTATCGATTATCCTTAC

CTCCAGAGTTACAGAAAATTCATGATGTTTTTCATGTTTCGATGCTTCGGAGATATAGAT

CGGATCCTTCTCATGTTATTCCCACTGAAGACATTGAACTTCGATCTGATTTAACTTATG

AAGAAGAACCAGTTCAAATATTAGCACGAGAAGTGAAAGAATTAAGAAATAAACGGGTTC

CTTTAGTACAAGTTTTATGGAGAAGCCATAGTGTGGAAGAAGCAACTTGGGAACCGGAAG

AGACAATGAGAGCACAATATCCTCATCTCTTCTCAGGTAAATTTCGAGGACGAAATTTAT

TAAGAGGGGGAGAAATGTAATGACCTAAAATTCATGGGCATCGGAAAAGTATAATATTGG

GCCTCCGTCCTAGTAAATTGAGTCCGAAAATAATTATTAGAAATATTTACGAGACTAGTA

GTGTGTTTAATTAGGTTTTAATTAAGTAAATTTAGCTTAATTTAGAGTAATTAGTAAAAA

GAATTAAATTGAATAAGAGTAAAAGTTTAATTATAGATTAAAGGAAAATAATAGGGACCA

AATGGGCAATTAAGCCACATTTGGAAGTTGAGGCGGCATAACATTGTAAAAATCTTAGAT

TTTTATATTATTATTTATATAAATATATAAATTAATTATAAAGTATATTATTAAATTAAT

TATATTATAAATATTATATTATTATATATAAAAGAAACAAAACAGAAAAGAAACAGAATA

GAAAGAACAAAGAAACAGAATAGAAGAGACGAAACAGGGGAGAAGCAGGGGAGAAAGAAG

AAAAAGAAGAAAAAAGGGGAAATAGGGTTTTTGAAGCTTGAAATTTAAATTGGTAAGTCA

AATTAGCCATTTTCTCTTAATTCTAATGTTTTAAAAGCTTTAAAACAAAGTTTTGATGGA

ATTAAGTTGATATTTTGTAAGTTCATAGGTTTTCAAGTATAGTTTATGTTGAACAAAAGA

GATGAATTAGGGATTAACTTGAAGGAATTTTAAGTTAGAATTGAAAAAGGGATTAAATTG

TAAAAGAAACTATAAGTTTTTTTTGTTTTAGGGACTAGATTGAGGAAAATTCGGAATTAA

GAAAATATGTTAAAAATTTAATAGTTAAATTTGAGTTTAAATGAAATTTGAATAGGAATA

AGGTGTGAATTGGTGTTATAAATTTGGTTATTAACATTTTTAATCAAAACAGTTTTGGGA

AGTAGCAATGGTCTGACTTTGAAAATTCACTAAAAATTTTATAAATTGAACTAGAGGATG

AACAAAATATGGAATTAAAGCTTATTGAGTCTAGTTTCTTATAGTAGAAACAATGTAAGC

AATTAATTGATGAATCAAGAGATATTTGAAATTTTGTAATACTGGTTCGGGGTGATTTCG

AGATGCCCTGTTTTAACTTTGGAAAATCATTAAAAATTGTACAAAAATTATTATGGAGTG

TAATTTATATATGTAAACTCCTTAATGAATCTAGTTTCAAAATAAATAAACAAGAACCTT

ATTCGAGTTCTGTACAATGAGATAATTTAGTTTTAGTGGAGAGAGGTCAGAACTGTCAAA

TGAAATAACAGGGGAGTATTTAACGAATAAACTGTATTAAATGGCTAGACCAAAAATTCT

GGAAATTTTATGATTAGAAGATATATGAGTCTAGTTTTAAGGAAAATTTACGGATATTAA

TTTGGAGTTTCGTAGCTCAAGATATAAATAATTTAGTAACAATGACCCAAGTAGACAGCT

TAATGGTGAAATTATATAAATACATTAAAAATGGTTAAATTTGCATGTTTAGGCTCATGA

ATTAAATTGAATCATGTTGTATTGATTATTATAAATTATTATTTTCGTAGCCAACAAAGA

ACCTAAAGCATCAGCATCGAAAGGAAAGGAGAAAGTCATCGAGGAGTAAACTCGAGAAAA

TTACGGTTTGTATTACTATAATTCAAGTTATTTATTATTAAATGTTAAATTTTAATTTAT

GTGTCTAGTAAATGAAATGTGAGGTAAGTATTATTATTATTATTATTATTATTATTATTA

TTATTATTATTATTATTATGAGTGGGAATTAAATTGAATAGTTGATATGAAATAATATTT

GAATTGTTTGTTGATTGAAAGCGGGAAATGAATTTAAATCGAATAGTGACCGATATTAAA

TTGAATGGAAATGTATTGAGTTGTGAAAATATGTTAATTGCGGATTAATTATTGATTGAA

AGGTGGAAAAATGATTGAATTGAAAGTGTGAGAAAGTGTGATTGAATTGGGATTATATGT

GATTTAAATACCCTATTAACTAGTCGGGCTGAGTCGGATATAGTTGGCATGCCATAGGAT

TGGAAGAGTTCAGGGATACTTCGACCTCGAGTCGATGAGACACTGGGTGTCACTATATTT

CTTCGGATAGATTCGATGAGGTACTGGGTACCAACTTTCTTCGGCTTTGCCGATGAGACA

CTGGGTGTCAACTATTGCTTCGAACTATCCGATGAGGCACTGGGTGCCATTCTGGTGTGT

TTGGTTGGATCCGTGTATTCGCCAAAGTCCGAGTTTTGTTAATAGGGTAAATGATGAAAT

GATAAACCGAACGAGTTGGTCAAACGAGCTATTGAAATGATATGAAAAAGTTGAATTGTG

AATTGAAATGTGAAATGAGATTGAGAAATGAACCTAAGGTTCGTGAATTATTCAAACTCA

AATTGTGGATATACGATATTGGTTGATGAATTGCTATTGTTGAAATATTTAATTTAAATT

GTATATACGATTTATGCTTTACATGTACATTATTGTTATAATTTGAATTATGGTAATACC

ACTGAGTATGAATTACTCAGCGTACGGTTGTTTCCGTGCGCAGGTCAATAGAAGTCAAAG

GTCTCGGTTCAGCATCCAGATTAATCCCGGCTTCGGCAAAACTTGGTGATGTATTTTTCC

TTTGGTAAAGGTGGCATGTACATAGATTGTGTATAAAGGTTATTATGTTTTATTATATAA

TGGTTAAAAATGTTAGTATTAAAAGTTTATGGATTTTAATGAAAGAAGTCTATCTATTTT

ATCTAATTAGTACATTGTTAAATTTTAAATTGGTATTAGATTGAGTTTGATTAGAAGTAT

TTAGAATAGAAAATGTGAATGTGAAATGAATTGGTTGAATTGATGATATTTGGGAACTAT

ATGGTTTTAATTTGC

>Acala1517-99

AGGTAATGACCCAAAATTCATGGGCATCGGAAAAGTATAATATCGGGCCTCCGTCCTAGT

AAATTGAGTCCGAAAATAATTATTAGAAATATTTACGAGACTAGTAGTGTGTTTAATTAG

GTTTTAATTAAGTAAATTTAGCTTAATTTAGAGTAATTAGTAAAAAGGATTAAATTGAAT

AAGAGTAAAAGTTTAATTATAGATTAAAGGAAAATAATAGGGACCAAATGGGCAATTAAG

CCACATTTGGAAGTTGAGGCGGCATAACATTGTAAAAATCTTAGATTTTTATATTATTAT

TTATATAAATATATAAATTAATTATAAAGTATATTATTAAATTAATTATATTATAAATAT

TATATTATTATATATAAAAGAAACAAAACAGAAAAGAAACAGAATAGAAAGAACAAAGAA

ACAGAATAGAAGAGACGAAACAGGGGAGAAGCAGGGGAGAAAGAAGAAAAAGAAGAAAAA

AGGGGAAATAGGGTTTTTGAAGCTTGAAATTTAAATTGGTAAGTCAAATTAGCCATTTTC

TCTTAATTCTAATGTTTTAAAAGCTTTAAAACAAAGTTTTGATGGAATTAAGTTGATATT

TTGTAAGTTCATAGGTTTTCAAGTATAGTTTATGTTGAACAAAAGAGATGAATTAGGGAT

TAACTTGAAGGAATTTTAAGTTAGAATTGAAAAAGGGATTAAATTGTAAAAGAAACTATA

AGTTTTTTTTGTTTTAGGGACTAGATTGAGGAAAATTCGGAATTAAGAAAATATGTTAAA

AATTTAATAGTTAAATTTGAGTTTAAATGAAATTTGAATAGGAATAAGGTGTGAATTGGT

GTTATAAATTTGGTTATTAACATTTTTAATCAAAACAGTTTTGGGAAGTAGCAATGGTCT

GACTTTGAAAATTCACTAAAAATTTTATAAATTGAACTAGAGGATGAACAAAATATGGAA

TTAAAGCTTATTGAGTCTAGTTTCTTATAGTAGAAACAATGTAAGCAATTAATTGATGAA

TCAAGAGATATTTGAAATTTTGTAATACTGGTTCGGGGTGATTTCGAGATGCCCTGTTTT

AACTTTGGAAAATCATTAAAAATTGTACAAAAATTATTATGGAGTGTAATTTATATATGT

GAACTCCTTAATGAATCTAGTTTCAAAATAAATAAACAAGAACCTTATTCGAGTTCTGTA

CAATGAGATAATTTAGTTTTAGTGGAGAGAGGTCAGAACTGTCAAATGAAATAACAGGGG

AGTATTTAACGAATAAACTGTATTAAATGGCTAGACCAAAAATTCTGGAAATTTTATGAT

TAGAAGATATATGAGTCTAGTTTTAAGGAAAATTTACGGATATTAATTTGGAGTTTCGTA

GCTCAAGATATAAATAATTTAGTAACAATGACCCAAGTAGACAGCTTAATGGTGAAATTA

TATAAATACATTAAAAATGGTTAAATTTGCATGTTTAGGCTCATGAATTAAATTGAATCA

TGTTGTATTGATTATTATAAATTATTATTTTCGTAGCCAACAAAGAACCTAAAGCATCAG

CATCGAAAGGAAAGGAGAAAGTCATCGAGGAGTAAACTCGAGAAAATTACGGTTTGTATT

ACTATAATTCAAGTTATTTATTATTAAATGTTAAATTTTAATTTATGTGTCTAGTAAATG

AAATGTGAGGTAAGTATTATTATTATTATTATTATTATTATTATTATTATTATTATTATT

ATGAGTGGGAATTAAATTGAATAGTTGATATGAAATAATATTTGAATTGTTTGTTGATTG

AAAGCGGGAAATGAATTTAAATCGAATAGTGACCGATATTAAATTGAATGGAAATGTATT

GAGTTGTGAAAATATGTTAATTGCGGATTAATTATTGATTGAAAGGTGGAAAAATGATTG

AATTGAAAGTGTGAGAAAGTGTGATTGAATTGGGATTATATGTGATTTAAATACCCTATT

AACTAGTCGGGCTGAGTCGGATATAGTTGGCATGCCATAGGATTGGAAGAGTTCAGGGAT

ACTTCGACCTCGAGTCGATGAGACACTGGGTGATTTCTTCGGATAGATTGGATGAGGTAC

TGGGTACCAACTTTCTTCGGCTTTGCCGATGAGACACTGGGTGTCAACTATTGCTTCGAA

CTATCCGATGAGGCACTGGGTGCCATTCTGGTGTGTTTGGTTGGATCCGTGTATCCGCCA

AAGTCCGAGTTTTGTTAATAGGGTAAATGATGAAATGATAAACCGAACGAGTTGGTCAAA

CGAGCTATTGAAATGATATGAAAAAGTTGAATTGTGAATTGAAATGTGAAATGAGATTGA

GAAATGAACCTAAGGTTCGTGAATTATTCAAACTCAAATTGTGGATATACGATATTGGTT

GATGAATTGCTATTGTTGAAATATTTAATTTAAATTGTATATACGATTTATGCTTTACAT

GTACATTATTGTTATAATTTGAATTATGGTAATACCACTGAGTATGAATTACTCAGCGTA

CGGTTGTTTCCGTGCGCAGGTCAATAGAAGTCAAAGGTCTCGGTTCAGCATCCAGATTAA

TCCCGGCTTCGGCAAAACTTGGTGATGTATTTTTCCTTTGGTAAAGGTGGCATGTACATA

GATTGTGTATAAAGGTTATTATGTTTTATTATATAATGGTTAAAAATGTTAGTATTAAAA

GTTTATGGATTTTAATGAAAGAAGTCTATCTATTTTATCTAATTAGTACATTGTTAAATT

TTAAATTGGTATTGTGTAGATTGAGTTTGATTAGAAGTATTTAGAATAGAAAATGTGAAT

GTGAAATGAATTGGTTGAATTGATGATATTTGGGAACTATATGGTTTTAATTTGCAGGGG

GTTTTATGTAAAAATAAGCAGAAATGCTGCCGAAATTTTTATAAAAAAAAATGAAGTCAT

TTGGTAAACAAATTAATAAATTTTATGAATTATTTTAATATATTGGTTATTTATTTAAGA

ATTGTTGTAAATCGTTCGATACGTCCGGTAGTGCCTCGTAATTCTGTTCCGGCGACGGTT

CGGGGTTAAGGGGTGTTACATTTTATGGTATCAGAGCTATCAGGTTTAGCCGATTCTCGG

CCTAAATCGAGCTCGGAATTGAGTCTAGATGTACATGCCACTGTCGAGTTAAACTGAGTC

GGGATTTTTGGATGCTGACCTATTTGTTTGTTTTGTTTTATAGATTAAAGATGTCTGAAG

AAAGAATAAATGATACTGATGAAAGAATGTATAGTGAAGATAGAGAATTAGATGAAACAG

AATCTGTTGCACCGAGTGTGAATCCGTTAGGCAACCAACCTTCTAATGTAGAACGAGAAA

ATGTCAGAGATAGAGATGAATCCCAATTACTGAGAATTATAGCTGATGCATTACAAAGAG

TAGCAGGAACTACTCCTGTTACGACTTCAGTACCTACTGTTAGACGGGCTCCGATAAAGG

AACTGAGGAAATATGGTGCCACTGAATTTATGGGTCTAAAAGGAGTTGATCCATCCATAG

CTGAAAATTGGATGGAGTCGACTAAAAGAATTTTGCAGCAATTGGATTGTACCCCCCGAG

AGTGTTTAATCTGTGCCGTATCGTTATTACAAGGGGAGGCTTATCTATGGTGGGAATCAG

TGGTTCGACATTTACCAGAGAGTCAGATAACGTGGGATCTATTTCAGAAGGAGTTTCAAA

AGAAATATATCGGAGAGATGTATATTGAAGACAAGAAACAAGAGTTTTTGTTGCTACAAC

AGGGTGATATGTCAGTAATAGATTATGAGAGGGAATTCTCGAGACTCAGTAGATATGCCT

CCGAGTTTATTCCGACAGAAGCCGATAGTTGTAAAAGATTTTTACGGGGTTTACGAGACG

AGATCAAAGTGCAGCTAGTATCCCATCGGATCACTGAGTTAGTAGATTTGATTGAACGAG

CTAAAATGGTGGAACAAGTTCTGGGCCTCGACAAAAAGACTGAAGTTGTTAGACCAACCG

GGAAGCGTACAGGAACTACCAGTTCGAATCCTCAGCCGAAAAGACCAAAGGAATTCCAAA

GTGGTTGGAGATCCAGTTTCAGGTCAGACAGAGGTGGTAGAAATAGGGGAAAACAGACGA

TGACATCTACTGGCAGTGTGAAAGGTCCTTCCCGAGAAATAGATATTCCAGACTGCCAAC

ACTGCGGAAAGAAACACAGAGGGGAATGTTGGAAATTAACTAGAGGCTGTTTTCGATGTG

GTTCTACAGACCATTTCATCAGAGACTGTCCGAAAGTTGATAGTACTGTACCCGTGACAT

CACAGAGATCGGTATCTACAGCTAGAGGCAGAGGGTTAGGAAGAGGTGGTTCGGTTTCAA

GGGGAGGAAGTATTAGGAGAAGCAATGATATTGCTACTCAGCAGTCTGAGGCTAAAGTAC

CTGCCAGAGCTTATGTGGTCAGAACACAGGAAGAAGGTGACGCCCACGATGTAGTAACAG

GTATATTCTTACTATATTCTGAGCCTGTTTATGCTTTAATTGATCCCGGATCTTCACATT

CTTATATAAATTCAAAATTAGTTGAATTGGGAAAATTTAATTCTGAAATATCTAGAGTGA

CTGTAGAAGTGTCGAGTCCGTTGGGGCAAACAGTATTAGTGAATCAGATCTGTCCGAGAT

GCCCGTTAATTATACAAAATAAAACTTTTCCTATTGACCTGTTGATTATGCCATTTGGAG

ATTTTGATATAATACTGGGGATGGATTGGTTGGCTGAGCACGGAGTGGTATTGGATTGTT

ATAAAAAGAAGTTTAGTATTCAGACAGAAGACGGGGACAGAATTGAAGTAAATGGTATCC

GTACTAATGGGCCGACACGTATTATTTCGGCAATAAAGGCTAATAAATTGCTTCAGCGGG

GTTGTACAGCGTATTTAGCCTATGTTATTAATTCTGATTTGGTTGGTAGTCAGTGCAGTA

AGATTAGAACCGTATGTGAGTTTCCAGATGTATTTCCTGAAGAGCTACCGGGTTTACCAC

CTGACAGAGAGGTTGAATTTGCTATAGAAGTGTATCCGGGTACAGCACCAATCTCTATAC

CACCGTATCGAATGTCACCCACTGAGTTGAAAGAGTTGAAAGTGCAGTTACAGGACTTGT

CAGATCGTGGATTTATTAGACCGAGCATCTCACCTTGGGGAGCTCCAGTATTGTTTGTTA

AAAAGAAAGATGGATCGATGCGGCTTTGTATTGATTACCGGCAGTTAAACAAAGTGACGA

TCAAGAACCGGTATCCGTTACCCCGTATAGATGATTTATTTGATCAACTAAAAGGAGCTT

CAGTATTTTCAAAGATTGACTTAAGATCTGGGTATTATCAGCTGAAGGTAAAAGAAAGTG

ATGTTCCGAAGACTGCATTTCGTACTCGATATGGTCATTATGAATTTTTGGTGATGCCGT

TCGGGTTGACTAATGCTCCAGCTGCTTTTATGGATCTGATGAATCGTATTTTTCAGCCGT

ATTTAGATCAGTTTGTGGTGGTTTTTATTGATGACATCTTGGTTTATTCGAAGTCAGAGT

CAGAGCATGATCAGCATCTCAGAACCGTGCTACAAATTCTGCGAGAAAAACAGTTGTACG

GGAAACTAAGTAAATGTGAATTCTGGTTATCAGAGGTAGTATTCTTGGGACATGTTGTAT

CTGCGGATGGGATTAGAGTTGATCCGAAGAAGATCGAGGCAATTGTTCAATGGAAGGCAC

CAAAGAATGTATCAGAGGTACGCAGTTTTCTTGGTTTGGCTGGGTATTACAGAAGATTTG

TAAATGGGTTTTCGAAGATAGCTTTGCCGATGACCAAATTACTACAGAAGAATGTTCCAT

TTATCTGGGATGATCAGTGTCAGAGGAGCTTTGAAACATTGAAACAGATGTTGACAGAGG

CACCAGTTTTAACTTTACCAGAATCAGGGAAAGATTTCATAGTGTACAGTGATGCTTCTT

TGAATGGTTTGGGTTGTGTATTGATGCAAGAAGGAAAAGTAATAGCTTATGCATCTCGAC

AGTTGAAGTCACATGAACGCAACTACCCGACACACGATTTAGAGTTAGCTGCTGTAATCT

TTGCATTGAAGATTTGGATACATTACTTGTATGGTGAGAAATGTTATATTTACACTGATC

ATAAAAGTCTAAAATATCTTCTGTCACAAAAGGAGTTGAATCTGAGACAGAGACGGTGGA

TTGAACTTCTGAAAGATTATGATTGTGTTATAGATTATCATCCAGGGAAGGCAAATGTGG

TAGCAGATGCATTGAGTAGAAAAGCAGCGATTGAATTACGAGCAATGTTCGCTCGACTTA

GTATTAAGGATGATGGAAGTTTGTTAGCTGAGTTAAGAGTCAAGCCGGTGATGTTTGATC

AAATCAGAGCAGCACAGTTAAAAGATGAAAAGTTGATGAGGAAAAGAGAAATGGTACAGT

ATGGTGCGGTAGAAAATTTTAGTATTGACGAGCATGATTGTTTGAGATTTCGAAATCGAA

TTTGTGTTCCATCTACTTCTGAGATTAAAGAATTGATTCTCCGAGAAGCACATAATAGTA

TTTTTGCTTTGCACCCAGGAGGAACGAAGATGTATCGTGATCTACGAGAACTGTATTGGT

GGCCAGGAATGAAGAAAGATATAGTTGAATATGTCAGTAAATGCTTGACTTGTCAGCGGG

TAAAAGCAGAACATCAGGTACCAACAGGCCTGTTACAGCCTATTACTATTCCCGAGTGGA

AATGGGATCGCATTACCATGGATTTTGTTACGGGGTTGCCATTGTCAGTGAGTAAAAAGA

ATGCTATTTGGGTGATTGTTGATCGACTCACAAAATCAGCTCATTTTATAGCAGTTAGAA

CCGACTGGTCATTACAGAAGCTTGCCGAGGTTTATATTCGAGAAATTGTTAGATTACATG

GTATTCCGGTATCAATAATTTCAGACAGAGATCCTCGATTCACTTCGAGATTTTGGAAGC

AGCTGCATGAATCATTGGGTACTCGACTTAGTTTCAGTACAGCTTTTCATCCTCAAACTG

ATGGACAATCTGAACGAGTAATTCAGATATTAGAAGATATGCTTCGAGCTTGTGTCATTG

ATTTTGAATCAGGTTGGGAACGTTATTTACCATTGGCCGAGTTTGTTTATAATAATAGTT

TCCAATCTAGTATTCAAATGGCTCCATATGAAGCACTTTATGGTCGAAGGTGTCGATCAC

CAATATGTTGGACAAAATTAAGAGAAAGAAAAGTGATTGGGCCGGAATTGATTCAAGAGA

CAGAAGAAACAGTTAAAAAGATTAAAGATAGACTGAAAGCCGCTTTCGACAGACAGAAAT

CTTACGCAGACTTGAAACGACGAGACATTGAATATTCCGTTGGTGATAAGGTATTCCTCA

AAGTATCGCCGTGGAAGAAAATTTTGAGATTTGGTCGGAAGGGAAAATTAAGTCCGCGCT

TTATTGGGCCGTATGAGATAGTGGAAAGAATTGGGCCTGTTGCTTATCGATTATCCTTAC

CTCCAGAGTTACAGAAAATTCATGATGTTTTTCATGTTTCGATGCTTCGGAGATATAGAT

CGGATCCTTCTCATGTTATTCCCACTGAAGACATTGAACTTCGATCTGATTTAACTTATG

AAGAAGAACCAGTTCAAATATTAGCACGAGAAGTGAAAGAATTAAGAAATAAACGGGTTC

CTTTAGTACAAGTTTTATGGAGAAGCCATAGTGTGGAAGAAGCAACTTGGGAACCGGAAG

AGACAATGAGAGCACAATATCCTCATCTCTTCTCAGGTAAATTTCGAGGACGAAATTTAT

TAAGAGGGGGAGAAATGTAATGACCTAAAATTCATGGGCATCGGAAAAGTATAATATTGG

GCCTCCGTCCTAGTAAATTGAGTCCGAAAATAATTATTAGAAATATTTACGAGACTAGTA

GTGTGTTTAATTAGGTTTTAATTAAGTAAATTTAGCTTAATTTAGAGTAATTAGTAAAAA

GGATTAAATTGAATAAGAGTAAAAGTTTAATTATAGATTAAAGGAAAATAATAGGGACCA

AATGGGCAATTAAGCCACATTTGGAAGTTGAGGCGGCATAACATTGTAAAAATCTTAGAT

TTTTATATTATTATTTATATAAATATATAAATTAATTATAAAGTATATTATTAAATTAAT

TATATTATAAATATTATATTATTATATATAAAAGAAACAAAACAGAAAAGAAACAGAATA

GAAAGAACAAAGAAACAGAATAGAAGAGACGAAACAGGGGAGAAGCAGGGGAGAAAGAAG

AAAAAGAAGAAAAAAGGGGAAATAGGGTTTTTGAAGCTTGAAATTTAAATTGGTAAGTCA

AATTAGCCATTTTCTCTTAATTCTAATGTTTTAAAAGCTTTAAAACAAAGTTTTGATGGA

ATTAAGTTGATATTTTGTAAGTTCATAGGTTTTCAAGTATAGTTTATGTTGAACAAAAGA

GATGAATTAGGGATTAACTTGAAGGAATTTTAAGTTAGAATTGAAAAAGGGATTAAATTG

TAAAAGAAACTATAAGTTTTTTTTGTTTTAGGGACTAGATTGAGGAAAATTCGGAATTAA

GAAAATATGTTAAAAATTTAATAGTTAAATTTGAGTTTAAATGAAATTTGAATAGGAATA

AGGTGTGAATTGGTGTTATAAATTTGGTTATTAACATTTTTAATCAAAACAGTTTTGGGA

AGTAGCAATGGTCTGACTTTGAAAATTCACTAAAAATTTTATAAATTGAACTAGAGGATG

AACAAAATATGGAATTAAAGCTTATTGAGTCTAGTTTCTTATAGTAGAAACAATGTAAGC

AATTAATTGATGAATCAAGAGATATTTGAAATTTTGTAATACTGGTTCGGGGTGATTTCG

AGATGCCCTGTTTTAACTTTGGAAAATCATTAAAAATTGTACAAAAATTATTATGGAGTG

TAATTTATATATGTAAACTCCTTAATGAATCTAGTTTCAAAATAAATAAACAAGAACCTT

ATTCGAGTTCTGTACAATGAGATAATTTAGTTTTAGTGGAGAGAGGTCAGAACTGTCAAA

TGAAATAACAGGGGAGTATTTAACGAATAAACTGTATTAAATGGCTAGACCAAAAATTCT

GGAAATTTTATGATTAGAAGATATATGAGTCTAGTTTTAAGGAAAATTTACGGATATTAA

TTTGGAGTTTCGTAGCTCAAGATATAAATAATTTAGTAACAATGACCCAAGTAGACAGCT

TAATGGTGAAATTATATAAATACATTAAAAATGGTTAAATTTGCATGTTTAGGCTCATGA

ATTAAATTGAATCATGTTGTATTGATTATTATAAATTATTATTTTCGTAGCCAACAAAGA

ACCTAAAGCATCAGCATCGAAAGGAAAGGAGAAAGTCATCGAGGAGTAAACTCGAGAAAA

TTACGGTTTGTATTACTATAATTCAAGTTATTTATTATTAAATGTTAAATTTTAATTTAT

GTGTCTAGTAAATGAAATGTGAGGTAAGTATTATTATTATTATTATTATTATTATTATTA

TTATTATTATTATTATTATGAGTGGGAATTAAATTGAATAGTTGATATGAAATAATATTT

GAATTGTTTGTTGATTGAAAGCGGGAAATGAATTTAAATCGAATAGTGACCGATATTAAA

TTGAATGGAAATGTATTGAGTTGTGAAAATATGTTAATTGCGGATTAATTATTGATTGAA

AGGTGGAAAAATGATTGAATTGAAAGTGTGAGAAAGTGTGATTGAATTGGGATTATATGT

GATTTAAATACCCTATTAACTAGTCGGGCTGAGTCGGATATAGTTGGCATGCCATAGGAT

TGGAAGAGTTCAGGGATACTTCGACCTCGAGTCGATGAGACACTGGGTGTCACTATATTT

CTTCGGATAGATTCGATGAGGTACTGGGTACCAACTTTCTTCGGCTTTGCCGATGAGACA

CTGGGTGTCAACTATTGCTTCGAACTATCCGATGAGGCACTGGGTGCCATTCTGGTGTGT

TTGGTTGGATCCGTGTATTCGCCAAAGTCCGAGTTTTGTTAATAGGGTAAATGATGAAAT

GATAAACCGAACGAGTTGGTCAAACGAGCTATTGAAATGATATGAAAAAGTTGAATTGTG

AATTGAAATGTGAAATGAGATTGAGAAATGAACCTAAGGTTCGTGAATTATTCAAACTCA

AATTGTGGATATACGATATTGGTTGATGAATTGCTATTGTTGAAATATTTAATTTAAATT

GTATATACGATTTATGCTTTACATGTACATTATTGTTATAATTTGAATTATGGTAATACC

ACTGAGTATGAATTACTCAGCGTACGGTTGTTTCCGTGCGCAGGTCAATAGAAGTCAAAG

GTCTCGGTTCAGCATCCAGATTAATCCCGGCTTCGGCAAAACTTGGTGATGTATTTTTCC

TTTGGTAAAGGTGGCATGTACATAGATTGTGTATAAAGGTTATTATGTTTTATTATATAA

TGGTTAAAAATGTTAGTATTAAAAGTTTATGGATTTTAATGAAAGAAGTCTATCTATTTT

ATCTAATTAGTACATTGTTAAATTTTAAATTGGTATTAGATTGAGTTTGATTAGAAGTAT

TTAGAATAGAAAATGTGAATGTGAAATGAATTGGTTGAATTGATGATATTTGGGAACTAT

ATGGTTTTAATTTGC

>Acala_Altima

AGGTAATGACCCAAAATTCATGGGCATCGGAAAAGTATAATATCGGGCCTCCGTCCTAGT

AAATTGAGTCCGAAAATAATTATTAGAAATATTTACGAGACTAGTAGTGTGTTTAATTAG

GTTTTAATTAAGTAAATTTAGCTTAATTTAGAGTAATTAGTAAAAAGGATTAAATTGAAT

AAGAGTAAAAGTTTAATTATAGATTAAAGGAAAATAATAGGGACCAAATGGGCAATTAAG

CCACATTTGGAAGTTGAGGCGGCATAACATTGTAAAAATCTTAGATTTTTATATTATTAT

TTATATAAATATATAAATTAATTATAAAGTATATTATTAAATTAATTATATTATAAATAT

TATATTATTATATATAAAAGAAACAAAACAGAAAAGAAACAGAATAGAAAGAACAAAGAA

ACAGAATAGAAGAGACGAAACAGGGGAGAAGCAGGGGAGAAAGAAGAAAAAGAAGAAAAA

AGGGGAAATAGGGTTTTTGAAGCTTGAAATTTAAATTGGTAAGTCAAATTAGCCATTTTC

TCTTAATTCTAATGTTTTAAAAGCTTTAAAACAAAGTTTTGATGGAATTAAGTTGATATT

TTGTAAGTTCATAGGTTTTCAAGTATAGTTTATGTTGAACAAAAGAGATGAATTAGGGAT

TAACTTGAAGGAATTTTAAGTTAGAATTGAAAAAGGGATTAAATTGTAAAAGAAACTATA

AGTTTTTTTTGTTTTAGGGACTAGATTGAGGAAAATTCGGAATTAAGAAAATATGTTAAA

AATTTAATAGTTAAATTTGAGTTTAAATGAAATTTGAATAGGAATAAGGTGTGAATTGGT

GTTATAAATTTGGTTATTAACATTTTTAATCAAAACAGTTTTGGGAAGTAGCAATGGTCT

GACTTTGAAAATTCACTAAAAATTTTATAAATTGAACTAGAGGATGAACAAAATATGGAA

TTAAAGCTTATTGAGTCTAGTTTCTTATAGTAGAAACAATGTAAGCAATTAATTGATGAA

TCAAGAGATATTTGAAATTTTGTAATACTGGTTCGGGGTGATTTCGAGATGCCCTGTTTT

AACTTTGGAAAATCATTAAAAATTGTACAAAAATTATTATGGAGTGTAATTTATATATGT

GAACTCCTTAATGAATCTAGTTTCAAAATAAATAAACAAGAACCTTATTCGAGTTCTGTA

CAATGAGATAATTTAGTTTTAGTGGAGAGAGGTCAGAACTGTCAAATGAAATAACAGGGG

AGTATTTAACGAATAAACTGTATTAAATGGCTAGACCAAAAATTCTGGAAATTTTATGAT

TAGAAGATATATGAGTCTAGTTTTAAGGAAAATTTACGGATATTAATTTGGAGTTTCGTA

GCTCAAGATATAAATAATTTAGTAACAATGACCCAAGTAGACAGCTTAATGGTGAAATTA

TATAAATACATTAAAAATGGTTAAATTTGCATGTTTAGGCTCATGAATTAAATTGAATCA

TGTTGTATTGATTATTATAAATTATTATTTTCGTAGCCAACAAAGAACCTAAAGCATCAG

CATCGAAAGGAAAGGAGAAAGTCATCGAGGAGTAAACTCGAGAAAATTACGGTTTGTATT

ACTATAATTCAAGTTATTTATTATTAAATGTTAAATTTTAATTTATGTGTCTAGTAAATG

AAATGTGAGGTAAGTATTATTATTATTATTATTATTATTATTATTATTATTATTATTATT

ATGAGTGGGAATTAAATTGAATAGTTGATATGAAATAATATTTGAATTGTTTGTTGATTG

AAAGCGGGAAATGAATTTAAATCGAATAGTGACCGATATTAAATTGAATGGAAATGTATT

GAGTTGTGAAAATATGTTAATTGCGGATTAATTATTGATTGAAAGGTGGAAAAATGATTG

AATTGAAAGTGTGAGAAAGTGTGATTGAATTGGGATTATATGTGATTTAAATACCCTATT

AACTAGTCGGGCTGAGTCGGATATAGTTGGCATGCCATAGGATTGGAAGAGTTCAGGGAT

ACTTCGACCTCGAGTCGATGAGACACTGGGTGATTTCTTCGGATAGATTGGATGAGGTAC

TGGGTACCAACTTTCTTCGGCTTTGCCGATGAGACACTGGGTGTCAACTATTGCTTCGAA

CTATCCGATGAGGCACTGGGTGCCATTCTGGTGTGTTTGGTTGGATCCGTGTATCCGCCA

AAGTCCGAGTTTTGTTAATAGGGTAAATGATGAAATGATAAACCGAACGAGTTGGTCAAA

CGAGCTATTGAAATGATATGAAAAAGTTGAATTGTGAATTGAAATGTGAAATGAGATTGA

GAAATGAACCTAAGGTTCGTGAATTATTCAAACTCAAATTGTGGATATACGATATTGGTT

GATGAATTGCTATTGTTGAAATATTTAATTTAAATTGTATATACGATTTATGCTTTACAT

GTACATTATTGTTATAATTTGAATTATGGTAATACCACTGAGTATGAATTACTCAGCGTA

CGGTTGTTTCCGTGCGCAGGTCAATAGAAGTCAAAGGTCTCGGTTCAGCATCCAGATTAA

TCCCGGCTTCGGCAAAACTTGGTGATGTATTTTTCCTTTGGTAAAGGTGGCATGTACATA

GATTGTGTATAAAGGTTATTATGTTTTATTATATAATGGTTAAAAATGTTAGTATTAAAA

GTTTATGGATTTTAATGAAAGAAGTCTATCTATTTTATCTAATTAGTACATTGTTAAATT

TTAAATTGGTATTGTGTAGATTGAGTTTGATTAGAAGTATTTAGAATAGAAAATGTGAAT

GTGAAATGAATTGGTTGAATTGATGATATTTGGGAACTATATGGTTTTAATTTGCAGGGG

GTTTTATGTAAAAATAAGCAGAAATGCTGCCGAAATTTTTATAAAAAAAAATGAAGTCAT

TTGGTAAACAAATTAATAAATTTTATGAATTATTTTAATATATTGGTTATTTATTTAAGA

ATTGTTGTAAATCGTTCGATACGTCCGGTAGTGCCTCGTAATTCTGTTCCGGCGACGGTT

CGGGGTTAAGGGGTGTTACATTTTATGGTATCAGAGCTATCAGGTTTAGCCGATTCTCGG

CCTAAATCGAGCTCGGAATTGAGTCTAGATGTACATGCCACTGTCGAGTTAAACTGAGTC

GGGATTTTTGGATGCTGACCTATTTGTTTGTTTTGTTTTATAGATTAAAGATGTCTGAAG

AAAGAATAAATGATACTGATGAAAGAATGTATAGTGAAGATAGAGAATTAGATGAAACAG

AATCTGTTGCACCGAGTGTGAATCCGTTAGGCAACCAACCTTCTAATGTAGAACGAGAAA

ATGTCAGAGATAGAGATGAATCCCAATTACTGAGAATTATAGCTGATGCATTACAAAGAG

TAGCAGGAACTACTCCTGTTACGACTTCAGTACCTACTGTTAGACGGGCTCCGATAAAGG

AACTGAGGAAATATGGTGCCACTGAATTTATGGGTCTAAAAGGAGTTGATCCATCCATAG

CTGAAAATTGGATGGAGTCGACTAAAAGAATTTTGCAGCAATTGGATTGTACCCCCCGAG

AGTGTTTAATCTGTGCCGTATCGTTATTACAAGGGGAGGCTTATCTATGGTGGGAATCAG

TGGTTCGACATTTACCAGAGAGTCAGATAACGTGGGATCTATTTCAGAAGGAGTTTCAAA

AGAAATATATCGGAGAGATGTATATTGAAGACAAGAAACAAGAGTTTTTGTTGCTACAAC

AGGGTGATATGTCAGTAATAGATTATGAGAGGGAATTCTCGAGACTCAGTAGATATGCCT

CCGAGTTTATTCCGACAGAAGCCGATAGTTGTAAAAGATTTTTACGGGGTTTACGAGACG

AGATCAAAGTGCAGCTAGTATCCCATCGGATCACTGAGTTAGTAGATTTGATTGAACGAG

CTAAAATGGTGGAACAAGTTCTGGGCCTCGACAAAAAGACTGAAGTTGTTAGACCAACCG

GGAAGCGTACAGGAACTACCAGTTCGAATCCTCAGCCGAAAAGACCAAAGGAATTCCAAA

GTGGTTGGAGATCCAGTTTCAGGTCAGACAGAGGTGGTAGAAATAGGGGAAAACAGACGA

TGACATCTACTGGCAGTGTGAAAGGTCCTTCCCGAGAAATAGATATTCCAGACTGCCAAC

ACTGCGGAAAGAAACACAGAGGGGAATGTTGGAAATTAACTAGAGGCTGTTTTCGATGTG

GTTCTACAGACCATTTCATCAGAGACTGTCCGAAAGTTGATAGTACTGTACCCGTGACAT

CACAGAGATCGGTATCTACAGCTAGAGGCAGAGGGTTAGGAAGAGGTGGTTCGGTTTCAA

GGGGAGGAAGTATTAGGAGAAGCAATGATATTGCTACTCAGCAGTCTGAGGCTAAAGTAC

CTGCCAGAGCTTATGTGGTCAGAACACAGGAAGAAGGTGACGCCCACGATGTAGTAACAG

GTATATTCTTACTATATTCTGAGCCTGTTTATGCTTTAATTGATCCCGGATCTTCACATT

CTTATATAAATTCAAAATTAGTTGAATTGGGAAAATTTAATTCTGAAATATCTAGAGTGA

CTGTAGAAGTGTCGAGTCCGTTGGGGCAAACAGTATTAGTGAATCAGATCTGTCCGAGAT

GCCCGTTAATTATACAAAATAAAACTTTTCCTATTGACCTGTTGATTATGCCATTTGGAG

ATTTTGATATAATACTGGGGATGGATTGGTTGGCTGAGCACGGAGTGGTATTGGATTGTT

ATAAAAAGAAGTTTAGTATTCAGACAGAAGACGGGGACAGAATTGAAGTAAATGGTATCC

GTACTAATGGGCCGACACGTATTATTTCGGCAATAAAGGCTAATAAATTGCTTCAGCGGG

GTTGTACAGCGTATTTAGCCTATGTTATTAATTCTGATTTGGTTGGTAGTCAGTGCAGTA

AGATTAGAACCGTATGTGAGTTTCCAGATGTATTTCCTGAAGAGCTACCGGGTTTACCAC

CTGACAGAGAGGTTGAATTTGCTATAGAAGTGTATCCGGGTACAGCACCAATCTCTATAC

CACCGTATCGAATGTCACCCACTGAGTTGAAAGAGTTGAAAGTGCAGTTACAGGACTTGT

CAGATCGTGGATTTATTAGACCGAGCATCTCACCTTGGGGAGCTCCAGTATTGTTTGTTA

AAAAGAAAGATGGATCGATGCGGCTTTGTATTGATTACCGGCAGTTAAACAAAGTGACGA

TCAAGAACCGGTATCCGTTACCCCGTATAGATGATTTATTTGATCAACTAAAAGGAGCTT

CAGTATTTTCAAAGATTGACTTAAGATCTGGGTATTATCAGCTGAAGGTAAAAGAAAGTG

ATGTTCCGAAGACTGCATTTCGTACTCGATATGGTCATTATGAATTTTTGGTGATGCCGT

TCGGGTTGACTAATGCTCCAGCTGCTTTTATGGATCTGATGAATCGTATTTTTCAGCCGT

ATTTAGATCAGTTTGTGGTGGTTTTTATTGATGACATCTTGGTTTATTCGAAGTCAGAGT

CAGAGCATGATCAGCATCTCAGAACCGTGCTACAAATTCTGCGAGAAAAACAGTTGTACG

GGAAACTAAGTAAATGTGAATTCTGGTTATCAGAGGTAGTATTCTTGGGACATGTTGTAT

CTGCGGATGGGATTAGAGTTGATCCGAAGAAGATCGAGGCAATTGTTCAATGGAAGGCAC

CAAAGAATGTATCAGAGGTACGCAGTTTTCTTGGTTTGGCTGGGTATTACAGAAGATTTG

TAAATGGGTTTTCGAAGATAGCTTTGCCGATGACCAAATTACTACAGAAGAATGTTCCAT

TTATCTGGGATGATCAGTGTCAGAGGAGCTTTGAAACATTGAAACAGATGTTGACAGAGG

CACCAGTTTTAACTTTACCAGAATCAGGGAAAGATTTCATAGTGTACAGTGATGCTTCTT

TGAATGGTTTGGGTTGTGTATTGATGCAAGAAGGAAAAGTAATAGCTTATGCATCTCGAC

AGTTGAAGTCACATGAACGCAACTACCCGACACACGATTTAGAGTTAGCTGCTGTAATCT

TTGCATTGAAGATTTGGATACATTACTTGTATGGTGAGAAATGTTATATTTACACTGATC

ATAAAAGTCTAAAATATCTTCTGTCACAAAAGGAGTTGAATCTGAGACAGAGACGGTGGA

TTGAACTTCTGAAAGATTATGATTGTGTTATAGATTATCATCCAGGGAAGGCAAATGTGG

TAGCAGATGCATTGAGTAGAAAAGCAGCGATTGAATTACGAGCAATGTTCGCTCGACTTA

GTATTAAGGATGATGGAAGTTTGTTAGCTGAGTTAAGAGTCAAGCCGGTGATGTTTGATC

AAATCAGAGCAGCACAGTTAAAAGATGAAAAGTTGATGAGGAAAAGAGAAATGGTACAGT

ATGGTGCGGTAGAAAATTTTAGTATTGACGAGCATGATTGTTTGAGATTTCGAAATCGAA

TTTGTGTTCCATCTACTTCTGAGATTAAAGAATTGATTCTCCGAGAAGCACATAATAGTA

TTTTTGCTTTGCACCCAGGAGGAACGAAGATGTATCGTGATCTACGAGAACTGTATTGGT

GGCCAGGAATGAAGAAAGATATAGTTGAATATGTCAGTAAATGCTTGACTTGTCAGCGGG

TAAAAGCAGAACATCAGGTACCAACAGGCCTGTTACAGCCTATTACTATTCCCGAGTGGA

AATGGGATCGCATTACCATGGATTTTGTTACGGGGTTGCCATTGTCAGTGAGTAAAAAGA

ATGCTATTTGGGTGATTGTTGATCGACTCACAAAATCAGCTCATTTTATAGCAGTTAGAA

CCGACTGGTCATTACAGAAGCTTGCCGAGGTTTATATTCGAGAAATTGTTAGATTACATG

GTATTCCGGTATCAATAATTTCAGACAGAGATCCTCGATTCACTTCGAGATTTTGGAAGC

AGCTGCATGAATCATTGGGTACTCGACTTAGTTTCAGTACAGCTTTTCATCCTCAAACTG

ATGGACAATCTGAACGAGTAATTCAGATATTAGAAGATATGCTTCGAGCTTGTGTCATTG

ATTTTGAATCAGGTTGGGAACGTTATTTACCATTGGCCGAGTTTGTTTATAATAATAGTT

TCCAATCTAGTATTCAAATGGCTCCATATGAAGCACTTTATGGTCGAAGGTGTCGATCAC

CAATATGTTGGACAAAATTAAGAGAAAGAAAAGTGATTGGGCCGGAATTGATTCAAGAGA

CAGAAGAAACAGTTAAAAAGATTAAAGATAGACTGAAAGCCGCTTTCGACAGACAGAAAT

CTTACGCAGACTTGAAACGACGAGACATTGAATATTCCGTTGGTGATAAGGTATTCCTCA

AAGTATCGCCGTGGAAGAAAATTTTGAGATTTGGTCGGAAGGGAAAATTAAGTCCGCGCT

TTATTGGGCCGTATGAGATAGTGGAAAGAATTGGGCCTGTTGCTTATCGATTATCCTTAC

CTCCAGAGTTACAGAAAATTCATGATGTTTTTCATGTTTCGATGCTTCGGAGATATAGAT

CGGATCCTTCTCATGTTATTCCCACTGAAGACATTGAACTTCGATCTGATTTAACTTATG

AAGAAGAACCAGTTCAAATATTAGCACGAGAAGTGAAAGAATTAAGAAATAAACGGGTTC

CTTTAGTACAAGTTTTATGGAGAAGCCATAGTGTGGAAGAAGCAACTTGGGAACCGGAAG

AGACAATGAGAGCACAATATCCTCATCTCTTCTCAGGTAAATTTCGAGGACGAAATTTAT

TAAGAGGGGGAGAAATGTAATGACCTAAAATTCATGGGCATCGGAAAAGTATAATATTGG

GCCTCCGTCCTAGTAAATTGAGTCCGAAAATAATTATTAGAAATATTTACGAGACTAGTA

GTGTGTTTAATTAGGTTTTAATTAAGTAAATTTAGCTTAATTTAGAGTAATTAGTAAAAA

GGATTAAATTGAATAAGAGTAAAAGTTTAATTATAGATTAAAGGAAAATAATAGGGACCA

AATGGGCAATTAAGCCACATTTGGAAGTTGAGGCGGCATAACATTGTAAAAATCTTAGAT

TTTTATATTATTATTTATATAAATATATAAATTAATTATAAAGTATATTATTAAATTAAT

TATATTATAAATATTATATTATTATATATAAAAGAAACAAAACAGAAAAGAAACAGAATA

GAAAGAACAAAGAAACAGAATAGAAGAGACGAAACAGGGGAGAAGCAGGGGAGAAAGAAG

AAAAAGAAGAAAAAAGGGGAAATAGGGTTTTTGAAGCTTGAAATTTAAATTGGTAAGTCA

AATTAGCCATTTTCTCTTAATTCTAATGTTTTAAAAGCTTTAAAACAAAGTTTTGATGGA

ATTAAGTTGATATTTTGTAAGTTCATAGGTTTTCAAGTATAGTTTATGTTGAACAAAAGA

GATGAATTAGGGATTAACTTGAAGGAATTTTAAGTTAGAATTGAAAAAGGGATTAAATTG

TAAAAGAAACTATAAGTTTTTTTTGTTTTAGGGACTAGATTGAGGAAAATTCGGAATTAA

GAAAATATGTTAAAAATTTAATAGTTAAATTTGAGTTTAAATGAAATTTGAATAGGAATA

AGGTGTGAATTGGTGTTATAAATTTGGTTATTAACATTTTTAATCAAAACAGTTTTGGGA

AGTAGCAATGGTCTGACTTTGAAAATTCACTAAAAATTTTATAAATTGAACTAGAGGATG

AACAAAATATGGAATTAAAGCTTATTGAGTCTAGTTTCTTATAGTAGAAACAATGTAAGC

AATTAATTGATGAATCAAGAGATATTTGAAATTTTGTAATACTGGTTCGGGGTGATTTCG

AGATGCCCTGTTTTAACTTTGGAAAATCATTAAAAATTGTACAAAAATTATTATGGAGTG

TAATTTATATATGTAAACTCCTTAATGAATCTAGTTTCAAAATAAATAAACAAGAACCTT

ATTCGAGTTCTGTACAATGAGATAATTTAGTTTTAGTGGAGAGAGGTCAGAACTGTCAAA

TGAAATAACAGGGGAGTATTTAACGAATAAACTGTATTAAATGGCTAGACCAAAAATTCT

GGAAATTTTATGATTAGAAGATATATGAGTCTAGTTTTAAGGAAAATTTACGGATATTAA

TTTGGAGTTTCGTAGCTCAAGATATAAATAATTTAGTAACAATGACCCAAGTAGACAGCT

TAATGGTGAAATTATATAAATACATTAAAAATGGTTAAATTTGCATGTTTAGGCTCATGA

ATTAAATTGAATCATGTTGTATTGATTATTATAAATTATTATTTTCGTAGCCAACAAAGA

ACCTAAAGCATCAGCATCGAAAGGAAAGGAGAAAGTCATCGAGGAGTAAACTCGAGAAAA

TTACGGTTTGTATTACTATAATTCAAGTTATTTATTATTAAATGTTAAATTTTAATTTAT

GTGTCTAGTAAATGAAATGTGAGGTAAGTATTATTATTATTATTATTATTATTATTATTA

TTATTATTATTATTATTATGAGTGGGAATTAAATTGAATAGTTGATATGAAATAATATTT

GAATTGTTTGTTGATTGAAAGCGGGAAATGAATTTAAATCGAATAGTGACCGATATTAAA

TTGAATGGAAATGTATTGAGTTGTGAAAATATGTTAATTGCGGATTAATTATTGATTGAA

AGGTGGAAAAATGATTGAATTGAAAGTGTGAGAAAGTGTGATTGAATTGGGATTATATGT

GATTTAAATACCCTATTAACTAGTCGGGCTGAGTCGGATATAGTTGGCATGCCATAGGAT

TGGAAGAGTTCAGGGATACTTCGACCTCGAGTCGATGAGACACTGGGTGTCACTATATTT

CTTCGGATAGATTCGATGAGGTACTGGGTACCAACTTTCTTCGGCTTTGCCGATGAGACA

CTGGGTGTCAACTATTGCTTCGAACTATCCGATGAGGCACTGGGTGCCATTCTGGTGTGT

TTGGTTGGATCCGTGTATTCGCCAAAGTCCGAGTTTTGTTAATAGGGTAAATGATGAAAT

GATAAACCGAACGAGTTGGTCAAACGAGCTATTGAAATGATATGAAAAAGTTGAATTGTG

AATTGAAATGTGAAATGAGATTGAGAAATGAACCTAAGGTTCGTGAATTATTCAAACTCA

AATTGTGGATATACGATATTGGTTGATGAATTGCTATTGTTGAAATATTTAATTTAAATT

GTATATACGATTTATGCTTTACATGTACATTATTGTTATAATTTGAATTATGGTAATACC

ACTGAGTATGAATTACTCAGCGTACGGTTGTTTCCGTGCGCAGGTCAATAGAAGTCAAAG

GTCTCGGTTCAGCATCCAGATTAATCCCGGCTTCGGCAAAACTTGGTGATGTATTTTTCC

TTTGGTAAAGGTGGCATGTACATAGATTGTGTATAAAGGTTATTATGTTTTATTATATAA

TGGTTAAAAATGTTAGTATTAAAAGTTTATGGATTTTAATGAAAGAAGTCTATCTATTTT

ATCTAATTAGTACATTGTTAAATTTTAAATTGGTATTAGATTGAGTTTGATTAGAAGTAT

TTAGAATAGAAAATGTGAATGTGAAATGAATTGGTTGAATTGATGATATTTGGGAACTAT

ATGGTTTTAATTTGC

>Acala_Maxxa

AGGTAATGACCCAAAATTCATGGGCATCGGAAAAGTATAATATCGGGCCTCCGTCCTAGT

AAATTGAGTCCGAAAATAATTATTAGAAATATTTACGAGACTAGTAGTGTGTTTAATTAG

GTTTTAATTAAGTAAATTTAGCTTAATTTAGAGTAATTAGTAAAAAGGATTAAATTGAAT

AAGAGTAAAAGTTTAATTATAGATTAAAGGAAAATAATAGGGACCAAATGGGCAATTAAG

CCACATTTGGAAGTTGAGGCGGCATAACATTGTAAAAATCTTAGATTTTTATATTATTAT

TTATATAAATATATAAATTAATTATAAAGTATATTATTAAATTAATTATATTATAAATAT

TATATTATTATATATAAAAGAAACAAAACAGAAAAGAAACAGAATAGAAAGAACAAAGAA

ACAGAATAGAAGAGACGAAACAGGGGAGAAGCAGGGGAGAAAGAAGAAAAAGAAGAAAAA

AGGGGAAATAGGGTTTTTGAAGCTTGAAATTTAAATTGGTAAGTCAAATTAGCCATTTTC

TCTTAATTCTAATGTTTTAAAAGCTTTAAAACAAAGTTTTGATGGAATTAAGTTGATATT

TTGTAAGTTCATAGGTTTTCAAGTATAGTTTATGTTGAACAAAAGAGATGAATTAGGGAT

TAACTTGAAGGAATTTTAAGTTAGAATTGAAAAAGGGATTAAATTGTAAAAGAAACTATA

AGTTTTTTTTGTTTTAGGGACTAGATTGAGGAAAATTCGGAATTAAGAAAATATGTTAAA

AATTTAATAGTTAAATTTGAGTTTAAATGAAATTTGAATAGGAATAAGGTGTGAATTGGT

GTTATAAATTTGGTTATTAACATTTTTAATCAAAACAGTTTTGGGAAGTAGCAATGGTCT

GACTTTGAAAATTCACTAAAAATTTTATAAATTGAACTAGAGGATGAACAAAATATGGAA

TTAAAGCTTATTGAGTCTAGTTTCTTATAGTAGAAACAATGTAAGCAATTAATTGATGAA

TCAAGAGATATTTGAAATTTTGTAATACTGGTTCGGGGTGATTTCGAGATGCCCTGTTTT

AACTTTGGAAAATCATTAAAAATTGTACAAAAACTATTATGGAGTGTAATTTATATATGT

GAACTCCTTAATGAATCTAGTTTCAAAATAAATAAACAAGAACCTTATTCGAGTTCTGTA

CAATGAGATAATTTAGTTTTAGTGGAGAGAGGTCAGAACTGTCAAATGAAATAACAGGGG

AGTATTTAACGAATAAACTGTATTAAATGGCTAGACCAAAAATTCTGGAAATTTTATGAT

TAGAAGATATATGAGTCTAGTTTTAAGGAAAATTTACGGATATTAATTTGGAGTTTCGTA

GCTCAAGATATAAATAATTTAGTAACAATGACCCAAGTAGACAGCTTAATGGTGAAATTA

TATAAATACATTAAAAATGGTTAAATTTGCATGTTTAGGCTCATGAATTAAATTGAATCA

TGTTGTATTGATTATTATAAATTATTATTTTCGTAGCCAACAAAGAACCTAAAGCATCAG

CATCGAAAGGAAAGGAGAAAGTCATCGAGGAGTAAACTCGAGAAAATTACGGTTTGTATT

ACTATAATTCAAGTTATTTATTATTAAATGTTAAATTTTAATTTATGTGTCTAGTAAATG

AAATGTGAGGTAAGTATTATTATTATTATTATTATTATTATTATTATTATTATTATTATT

ATGAGTGGGAATTAAATTGAATAGTTGATATGAAATAATATTTGAATTGTTTGTTGATTG

AAAGCGGGAAATGAATTTAAATCGAATAGTGACCGATATTAAATTGAATGGAAATGTATT

GAGTTGTGAAAATATGTTAATTGCGGATTAATTATTGATTGAAAGGTGGAAAAATGATTG

AATTGAAAGTGTGAGAAAGTGTGATTGAATTGGGATTATATGTGATTTAAATACCCTATT

AACTAGTCGGGCTGAGTCGGATATAGTTGGCATGCCATAGGATTGGAAGAGTTCAGGGAT

ACTTCGACCTCGAGTCGATGAGACACTGGGTGATTTCTTCGGATAGATTGGATGAGGTAC

TGGGTACCAACTTTCTTCGGCTTTGCCGATGAGACACTGGGTGTCAACTATTGCTTCGAA

CTATCCGATGAGGCACTGGGTGCCATTCTGGTGTGTTTGGTTGGATCCGTGTATCCGCCA

AAGTCCGAGTTTTGTTAATAGGGTAAATGATGAAATGATAAACCGAACGAGTTGGTCAAA

CGAGCTATTGAAATGATATGAAAAAGTTGAATTGTGAATTGAAATGTGAAATGAGATTGA

GAAATGAACCTAAGGTTCGTGAATTATTCAAACTCAAATTGTGGATATACGATATTGGTT

GATGAATTGCTATTGTTGAAATATTTAATTTAAATTGTATATACGATTTATGCTTTACAT

GTACATTATTGTTATAATTTGAATTATGGTAATACCACTGAGTATGAATTACTCAGCGTA

CGGTTGTTTCCGTGCGCAGGTCAATAGAAGTCAAAGGTCTCGGTTCAGCATCCAGATTAA

TCCCGGCTTCGGCAAAACTTGGTGATGTATTTTTCCTTTGGTAAAGGTGGCATGTACATA

GATTGTGTATAAAGGTTATTATGTTTTATTATATAATGGTTAAAAATGTTAGTATTAAAA

GTTTATGGATTTTAATGAAAGAAGTCTATCTATTTTATCTAATTAGTACATTGTTAAATT

TTAAATTGGTATTGTGTAGATTGAGTTTGATTAGAAGTATTTAGAATAGAAAATGTGAAT

GTGAAATGAATTGGTTGAATTGATGATATTTGGGAACTATATGGTTTTAATTTGCAGGGG

GTTTTATGTAAAAATAAGCAGAAATGCTGCCGAAATTTTTATAAAAAAAAATGAAGTCAT

TTGGTAAACAAATTAATAAATTTTATGAATTATTTTAATATATTGGTTATTTATTTAAGA

ATTGTTGTAAATCGTTCGATACGTCCGGTAGTGCCTCGTAATTCTGTTCCGGCGACGGTT

CGGGGTTAAGGGGTGTTACATTTTATGGTATCAGAGCTATCAGGTTTAGCCGATTCTCGG

CCTAAATCGAGCTCGGAATTGAGTCTAGATGTACATGCCACTGTCGAGTTAAACTGAGTC

GGGATTTTTGGATGCTGACCTATTTGTTTGTTTTGTTTTATAGATTAAAGATGTCTGAAG

AAAGAATAAATGATACTGATGAAAGAATGTATAGTGAAGATAGAGAATTAGATGAAACAG

AATCTGTTGCACCGAGTGTGAATCCGTTAGGCAACCAACCTTCTAATGTAGAACGAGAAA

ATGTCAGAGATAGAGATGAATCCCAATTACTGAGAATTATAGCTGATGCATTACAAAGAG

TAGCAGGAACTACTCCTGTTACGACTTCAGTACCTACTGTTAGACGGGCTCCGATAAAGG

AACTGAGGAAATATGGTGCCACTGAATTTATGGGTCTAAAAGGAGTTGATCCATCCATAG

CTGAAAATTGGATGGAGTCGACTAAAAGAATTTTGCAGCAATTGGATTGTACCCCCCGAG

AGTGTTTAATCTGTGCCGTATCGTTATTACAAGGGGAGGCTTATCTATGGTGGGAATCAG

TGGTTCGACATTTACCAGAGAGTCAGATAACGTGGGATCTATTTCAGAAGGAGTTTCAAA

AGAAATATATCGGAGAGATGTATATTGAAGACAAGAAACAAGAGTTTTTGTTGCTACAAC

AGGGTGATATGTCAGTAATAGATTATGAGAGGGAATTCTCGAGACTCAGTAGATATGCCT

CCGAGTTTATTCCGACAGAAGCCGATAGTTGTAAAAGATTTTTACGGGGTTTACGAGACG

AGATCAAAGTGCAGCTAGTATCCCATCGGATCACTGAGTTAGTAGATTTGATTGAACGAG

CTAAAATGGTGGAACAAGTTCTGGGCCTCGACAAAAAGACTGAAGTTGTTAGACCAACCG

GGAAGCGTACAGGAACTACCAGTTCGAATCCTCAGCCGAAAAGACCAAAGGAATTCCAAA

GTGGTTGGAGATCCAGTTTCAGGTCAGACAGAGGTGGTAGAAATAGGGGAAAACAGACGA

TGACATCTACTGGCAGTGTGAAAGGTCCTTCCCGAGAAATAGATATTCCAGACTGCCAAC

ACTGCGGAAAGAAACACAGAGGGGAATGTTGGAAATTAACTAGAGGCTGTTTTCGATGTG

GTTCTACAGACCATTTCATCAGAGACTGTCCGAAAGTTGATAGTACTGTACCCGTGACAT

CACAGAGATCGGTATCTACAGCTAGAGGCAGAGGGTTAGGAAGAGGTGGTTCGGTTTCAA

GGGGAGGAAGTATTAGGAGAAGCAATGATATTGCTACTCAGCAGTCTGAGGCTAAAGTAC

CTGCCAGAGCTTATGTGGTCAGAACACAGGAAGAAGGTGACGCCCACGATGTAGTAACAG

GTATATTCTTACTATATTCTGAGCCTGTTTATGCTTTAATTGATCCCGGATCTTCACATT

CTTATATAAATTCAAAATTAGTTGAATTGGGAAAATTTAATTCTGAAATATCTAGAGTGA

CTGTAGAAGTGTCGAGTCCGTTGGGGCAAACAGTATTAGTGAATCAGATCTGTCCGAGAT

GCCCGTTAATTATACAAAATAAAACTTTTCCTATTGACCTGTTGATTATGCCATTTGGAG

ATTTTGATATAATACTGGGGATGGATTGGTTGGCTGAGCACGGAGTGGTATTGGATTGTT

ATAAAAAGAAGTTTAGTATTCAGACAGAAGACGGGGACAGAATTGAAGTAAATGGTATCC

GTACTAATGGGCCGACACGTATTATTTCGGCAATAAAGGCTAATAAATTGCTTCAGCGGG

GTTGTACAGCGTATTTAGCCTATGTTATTAATTCTGATTTGGTTGGTAGTCAGTGCAGTA

AGATTAGAACCGTATGTGAGTTTCCAGATGTATTTCCTGAAGAGCTACCGGGTTTACCAC

CTGACAGAGAGGTTGAATTTGCTATAGAAGTGTATCCGGGTACAGCACCAATCTCTATAC

CACCGTATCGAATGTCACCCACTGAGTTGAAAGAGTTGAAAGTGCAGTTACAGGACTTGT

CAGATCGTGGATTTATTAGACCGAGCATCTCACCTTGGGGAGCTCCAGTATTGTTTGTTA

AAAAGAAAGATGGATCGATGCGGCTTTGTATTGATTACCGGCAGTTAAACAAAGTGACGA

TCAAGAACCGGTATCCGTTACCCCGTATAGATGATTTATTTGATCAACTAAAAGGAGCTT

CAGTATTTTCAAAGATTGACTTAAGATCTGGGTATTATCAGCTGAAGGTAAAAGAAAGTG

ATGTTCCGAAGACTGCATTTCGTACTCGATATGGTCATTATGAATTTTTGGTGATGCCGT

TCGGGTTGACTAATGCTCCAGCTGCTTTTATGGATCTGATGAATCGTATTTTTCAGCCGT

ATTTAGATCAGTTTGTGGTGGTTTTTATTGATGACATCTTGGTTTATTCGAAGTCAGAGT

CAGAGCATGATCAGCATCTCAGAACCGTGCTACAAATTCTGCGAGAAAAACAGTTGTACG

GGAAACTAAGTAAATGTGAATTCTGGTTATCAGAGGTAGTATTCTTGGGACATGTTGTAT

CTGCGGATGGGATTAGAGTTGATCCGAAGAAGATCGAGGCAATTGTTCAATGGAAGGCAC

CAAAGAATGTATCAGAGGTACGCAGTTTTCTTGGTTTGGCTGGGTATTACAGAAGATTTG

TAAATGGGTTTTCGAAGATAGCTTTGCCGATGACCAAATTACTACAGAAGAATGTTCCAT

TTATCTGGGATGATCAGTGTCAGAGGAGCTTTGAAACATTGAAACAGATGTTGACAGAGG

CACCAGTTTTAACTTTACCAGAATCAGGGAAAGATTTCATAGTGTACAGTGATGCTTCTT

TGAATGGTTTGGGTTGTGTATTGATGCAAGAAGGAAAAGTAATAGCTTATGCATCTCGAC

AGTTGAAGTCACATGAACGCAACTACCCGACACACGATTTAGAGTTAGCTGCTGTAATCT

TTGCATTGAAGATTTGGATACATTACTTGTATGGTGAGAAATGTTATATTTACACTGATC

ATAAAAGTCTAAAATATCTTCTGTCACAAAAGGAGTTGAATCTGAGACAGAGACGGTGGA

TTGAACTTCTGAAAGATTATGATTGTGTTATAGATTATCATCCAGGGAAGGCAAATGTGG

TAGCAGATGCATTGAGTAGAAAAGCAGCGATTGAATTACGAGCAATGTTCGCTCGACTTA

GTATTAAGGATGATGGAAGTTTGTTAGCTGAGTTAAGAGTCAAGCCGGTGATGTTTGATC

AAATCAGAGCAGCACAGTTAAAAGATGAAAAGTTGATGAGGAAAAGAGAAATGGTACAGT

ATGGTGCGGTAGAAAATTTTAGTATTGACGAGCATGATTGTTTGAGATTTCGAAATCGAA

TTTGTGTTCCATCTACTTCTGAGATTAAAGAATTGATTCTCCGAGAAGCACATAATAGTA

TTTTTGCTTTGCACCCAGGAGGAACGAAGATGTATCGTGATCTACGAGAACTGTATTGGT

GGCCAGGAATGAAGAAAGATATAGTTGAATATGTCAGTAAATGCTTGACTTGTCAGCGGG

TAAAAGCAGAACATCAGGTACCAACAGGCCTGTTACAGCCTATTACTATTCCCGAGTGGA

AATGGGATCGCATTACCATGGATTTTGTTACGGGGTTGCCATTGTCAGTGAGTAAAAAGA

ATGCTATTTGGGTGATTGTTGATCGACTCACAAAATCAGCTCATTTTATAGCAGTTAGAA

CCGACTGGTCATTACAGAAGCTTGCCGAGGTTTATATTCGAGAAATTGTTAGATTACATG

GTATTCCGGTATCAATAATTTCAGACAGAGATCCTCGATTCACTTCGAGATTTTGGAAGC

AGCTGCATGAATCATTGGGTACTCGACTTAGTTTCAGTACAGCTTTTCATCCTCAAACTG

ATGGACAATCTGAACGAGTAATTCAGATATTAGAAGATATGCTTCGAGCTTGTGTCATTG

ATTTTGAATCAGGTTGGGAACGTTATTTACCATTGGCCGAGTTTGTTTATAATAATAGTT

TCCAATCTAGTATTCAAATGGCTCCATATGAAGCACTTTATGGTCGAAGGTGTCGATCAC

CAATATGTTGGACAAAATTAAGAGAAAGAAAAGTGATTGGGCCGGAATTGATTCAAGAGA

CAGAAGAAACAGTTAAAAAGATTAAAGATAGACTGAAAGCCGCTTTCGACAGACAGAAAT

CTTACGCAGACTTGAAACGACGAGACATTGAATATTCCGTTGGTGATAAGGTATTCCTCA

AAGTATCGCCGTGGAAGAAAATTTTGAGATTTGGTCGGAAGGGAAAATTAAGTCCGCGCT

TTATTGGGCCGTATGAGATAGTGGAAAGAATTGGGCCTGTTGCTTATCGATTATCCTTAC

CTCCAGAGTTACAGAAAATTCATGATGTTTTTCATGTTTCGATGCTTCGGAGATATAGAT

CGGATCCTTCTCATGTTATTCCCACTGAAGACATTGAACTTCGATCTGATTTAACTTATG

AAGAAGAACCAGTTCAAATATTAGCACGAGAAGTGAAAGAATTAAGAAATAAACGGGTTC

CTTTAGTACAAGTTTTATGGAGAAGCCATAGTGTGGAAGAAGCAACTTGGGAACCGGAAG

AGACAATGAGAGCACAATATCCTCATCTCTTCTCAGGTAAATTTCGAGGACGAAATTTAT

TAAGAGGGGGAGAAATGTAATGACCTAAAATTCATGGGCATCGGAAAAGTATAATATTGG

GCCTCCGTCCTAGTAAATTGAGTCCGAAAATAATTATTAGAAATATTTACGAGACTAGTA

GTGTGTTTAATTAGGTTTTAATTAAGTAAATTTAGCTTAATTTAGAGTAATTAGTAAAAA

GGATTAAATTGAATAAGAGTAAAAGTTTAATTATAGATTAAAGGAAAATAATAGGGACCA

AATGGGCAATTAAGCCACATTTGGAAGTTGAGGCGGCATAACATTGTAAAAATCTTAGAT

TTTTATATTATTATTTATATAAATATATAAATTAATTATAAAGTATATTATTAAATTAAT

TATATTATAAATATTATATTATTATATATAAAAGAAACAAAACAGAAAAGAAACAGAATA

GAAAGAACAAAGAAACAGAATAGAAGAGACGAAACAGGGGAGAAGCAGGGGAGAAAGAAG

AAAAAGAAGAAAAAAGGGGAAATAGGGTTTTTGAAGCTTGAAATTTAAATTGGTAAGTCA

AATTAGCCATTTTCTCTTAATTCTAATGTTTTAAAAGCTTTAAAACAAAGTTTTGATGGA

ATTAAGTTGATATTTTGTAAGTTCATAGGTTTTCAAGTATAGTTTATGTTGAACAAAAGA

GATGAATTAGGGATTAACTTGAAGGAATTTTAAGTTAGAATTGAAAAAGGGATTAAATTG

TAAAAGAAACTATAAGTTTTTTTTGTTTTAGGGACTAGATTGAGGAAAATTCGGAATTAA

GAAAATATGTTAAAAATTTAATAGTTAAATTTGAGTTTAAATGAAATTTGAATAGGAATA

AGGTGTGAATTGGTGTTATAAATTTGGTTATTAACATTTTTAATCAAAACAGTTTTGGGA

AGTAGCAATGGTCTGACTTTGAAAATTCACTAAAAATTTTATAAATTGAACTAGAGGATG

AACAAAATATGGAATTAAAGCTTATTGAGTCTAGTTTCTTATAGTAGAAACAATGTAAGC

AATTAATTGATGAATCAAGAGATATTTGAAATTTTGTAATACTGGTTCGGGGTGATTTCG

AGATGCCCTGTTTTAACTTTGGAAAATCATTAAAAATTGTACAAAAATTATTATGGAGTG

TAATTTATATATGTAAACTCCTTAATGAATCTAGTTTCAAAATAAATAAACAAGAACCTT

ATTCGAGTTCTGTACAATGAGATAATTTAGTTTTAGTGGAGAGAGGTCAGAACTGTCAAA

TGAAATAACAGGGGAGTATTTAACGAATAAACTGTATTAAATGGCTAGACCAAAAATTCT

GGAAATTTTATGATTAGAAGATATATGAGTCTAGTTTTAAGGAAAATTTACGGATATTAA

TTTGGAGTTTCGTAGCTCAAGATATAAATAATTTAGTAACAATGACCCAAGTAGACAGCT

TAATGGTGAAATTATATAAATACATTAAAAATGGTTAAATTTGCATGTTTAGGCTCATGA

ATTAAATTGAATCATGTTGTATTGATTATTATAAATTATTATTTTCGTAGCCAACAAAGA

ACCTAAAGCATCAGCATCGAAAGGAAAGGAGAAAGTCATCGAGGAGTAAACTCGAGAAAA

TTACGGTTTGTATTACTATAATTCAAGTTATTTATTATTAAATGTTAAATTTTAATTTAT

GTGTCTAGTAAATGAAATGTGAGGTAAGTATTATTATTATTATTATTATTATTATTATTA

TTATTATTATTATTATTATGAGTGGGAATTAAATTGAATAGTTGATATGAAATAATATTT

GAATTGTTTGTTGATTGAAAGCGGGAAATGAATTTAAATCGAATAGTGACCGATATTAAA

TTGAATGGAAATGTATTGAGTTGTGAAAATATGTTAATTGCGGATTAATTATTGATTGAA

AGGTGGAAAAATGATTGAATTGAAAGTGTGAGAAAGTGTGATTGAATTGGGATTATATGT

GATTTAAATACCCTATTAACTAGTCGGGCTGAGTCGGATATAGTTGGCATGCCATAGGAT

TGGAAGAGTTCAGGGATACTTCGACCTCGAGTCGATGAGACACTGGGTGTCACTATATTT

CTTCGGATAGATTCGATGAGGTACTGGGTACCAACTTTCTTCGGCTTTGCCGATGAGACA

CTGGGTGTCAACTATTGCTTCGAACTATCCGATGAGGCACTGGGTGCCATTCTGGTGTGT

TTGGTTGGATCCGTGTATTCGCCAAAGTCCGAGTTTTGTTAATAGGGTAAATGATGAAAT

GATAAACCGAACGAGTTGGTCAAACGAGCTATTGAAATGATATGAAAAAGTTGAATTGTG

AATTGAAATGTGAAATGAGATTGAGAAATGAACCTAAGGTTCGTGAATTATTCAAACTCA

AATTGTGGATATACGATATTGGTTGATGAATTGCTATTGTTGAAATATTTAATTTAAATT

GTATATACGATTTATGCTTTACATGTACATTATTGTTATAATTTGAATTATGGTAATACC

ACTGAGTATGAATTACTCAGCGTACGGTTGTTTCCGTGCGCAGGTCAATAGAAGTCAAAG

GTCTCGGTTCAGCATCCAGATTAATCCCGGCTTCGGCAAAACTTGGTGATGTATTTTTCC

TTTGGTAAAGGTGGCATGTACATAGATTGTGTATAAAGGTTATTATGTTTTATTATATAA

TGGTTAAAAATGTTAGTATTAAAAGTTTATGGATTTTAATGAAAGAAGTCTATCTATTTT

ATCTAATTAGTACATTGTTAAATTTTAAATTGGTATTAGATTGAGTTTGATTAGAAGTAT

TTAGAATAGAAAATGTGAATGTGAAATGAATTGGTTGAATTGATGATATTTGGGAACTAT

ATGGTTTTAATTTGC

>Acala_Ultima_145

AGGTAATGACCCAAAATTCATGGGCATCGGAAAAGTATAATATCGGGCCTCCGTCCTAGT

AAATTGAGTCCGAAAATAATTATTAGAAATATTTACGAGACTAGTAGTGTGTTTAATTAG

GTTTTAATTAAGTAAATTTAGCTTAATTTAGAGTAATTAGTAAAAAGGATTAAATTGAAT

AAGAGTAAAAGTTTAATTATAGATTAAAGGAAAATAATAGGGACCAAATGGGCAATTAAG

CCACATTTGGAAGTTGAGGCGGCATAACATTGTAAAAATCTTAGATTTTTATATTATTAT

TTATATAAATATATAAATTAATTATAAAGTATATTATTAAATTAATTATATTATAAATAT

TATATTATTATATATAAAAGAAACAAAACAGAAAAGAAACAGAATAGAAAGAACAAAGAA

ACAGAATAGAAGAGACGAAACAGGGGAGAAGCAGGGGAGAAAGAAGAAAAAGAAGAAAAA

AGGGGAAATAGGGTTTTTGAAGCTTGAAATTTAAATTGGTAAGTCAAATTAGCCATTTTC

TCTTAATTCTAATGTTTTAAAAGCTTTAAAACAAAGTTTTGATGGAATTAAGTTGATATT

TTGTAAGTTCATAGGTTTTCAAGTATAGTTTATGTTGAACAAAAGAGATGAATTAGGGAT

TAACTTGAAGGAATTTTAAGTTAGAATTGAAAAAGGGATTAAATTGTAAAAGAAACTATA

AGTTTTTTTTGTTTTAGGGACTAGATTGAGGAAAATTCGGAATTAAGAAAATATGTTAAA

AATTTAATAGTTAAATTTGAGTTTAAATGAAATTTGAATAGGAATAAGGTGTGAATTGGT

GTTATAAATTTGGTTATTAACATTTTTAATCAAAACAGTTTTGGGAAGTAGCAATGGTCT

GACTTTGAAAATTCACTAAAAATTTTATAAATTGAACTAGAGGATGAACAAAATATGGAA

TTAAAGCTTATTGAGTCTAGTTTCTTATAGTAGAAACAATGTAAGCAATTAATTGATGAA

TCAAGAGATATTTGAAATTTTGTAATACTGGTTCGGGGTGATTTCGAGATGCCCTGTTTT

AACTTTGGAAAATCATTAAAAATTGTACAAAAATTATTATGGAGTGTAATTTATATATGT

GAACTCCTTAATGAATCTAGTTTCAAAATAAATAAACAAGAACCTTATTCGAGTTCTGTA

CAATGAGATAATTTAGTTTTAGTGGAGAGAGGTCAGAACTGTCAAATGAAATAACAGGGG

AGTATTTAACGAATAAACTGTATTAAATGGCTAGACCAAAAATTCTGGAAATTTTATGAT

TAGAAGATATATGAGTCTAGTTTTAAGGAAAATTTACGGATATTAATTTGGAGTTTCGTA

GCTCAAGATATAAATAATTTAGTAACAATGACCCAAGTAGACAGCTTAATGGTGAAATTA

TATAAATACATTAAAAATGGTTAAATTTGCATGTTTAGGCTCATGAATTAAATTGAATCA

TGTTGTATTGATTATTATAAATTATTATTTTCGTAGCCAACAAAGAACCTAAAGCATCAG

CATCGAAAGGAAAGGAGAAAGTCATCGAGGAGTAAACTCGAGAAAATTACGGTTTGTATT

ACTATAATTCAAGTTATTTATTATTAAATGTTAAATTTTAATTTATGTGTCTAGTAAATG

AAATGTGAGGTAAGTATTATTATTATTATTATTATTATTATTATTATTATTATTATTATT

ATGAGTGGGAATTAAATTGAATAGTTGATATGAAATAATATTTGAATTGTTTGTTGATTG

AAAGCGGGAAATGAATTTAAATCGAATAGTGACCGATATTAAATTGAATGGAAATGTATT

GAGTTGTGAAAATATGTTAATTGCGGATTAATTATTGATTGAAAGGTGGAAAAATGATTG

AATTGAAAGTGTGAGAAAGTGTGATTGAATTGGGATTATATGTGATTTAAATACCCTATT

AACTAGTCGGGCTGAGTCGGATATAGTTGGCATGCCATAGGATTGGAAGAGTTCAGGGAT

ACTTCGACCTCGAGTCGATGAGACACTGGGTGATTTCTTCGGATAGATTGGATGAGGTAC

TGGGTACCAACTTTCTTCGGCTTTGCCGATGAGACACTGGGTGTCAACTATTGCTTCGAA

CTATCCGATGAGGCACTGGGTGCCATTCTGGTGTGTTTGGTTGGATCCGTGTATCCGCCA

AAGTCCGAGTTTTGTTAATAGGGTAAATGATGAAATGATAAACCGAACGAGTTGGTCAAA

CGAGCTATTGAAATGATATGAAAAAGTTGAATTGTGAATTGAAATGTGAAATGAGATTGA

GAAATGAACCTAAGGTTCGTGAATTATTCAAACTCAAATTGTGGATATACGATATTGGTT

GATGAATTGCTATTGTTGAAATATTTAATTTAAATTGTATATACGATTTATGCTTTACAT

GTACATTATTGTTATAATTTGAATTATGGTAATACCACTGAGTATGAATTACTCAGCGTA

CGGTTGTTTCCGTGCGCAGGTCAATAGAAGTCAAAGGTCTCGGTTCAGCATCCAGATTAA

TCCCGGCTTCGGCAAAACTTGGTGATGTATTTTTCCTTTGGTAAAGGTGGCATGTACATA

GATTGTGTATAAAGGTTATTATGTTTTATTATATAATGGTTAAAAATGTTAGTATTAAAA

GTTTATGGATTTTAATGAAAGAAGTCTATCTATTTTATCTAATTAGTACATTGTTAAATT

TTAAATTGGTATTGTGTAGATTGAGTTTGATTAGAAGTATTTAGAATAGAAAATGTGAAT

GTGAAATGAATTGGTTGAATTGATGATATTTGGGAACTATATGGTTTTAATTTGCAGGGG

GTTTTATGTAAAAATAAGCAGAAATGCTGCCGAAATTTTTACAAAAAAAAATGAAGTCAT

TTGGTAAACAAATTAATAAATTTTATGAATTATTTTAATATATTGGTTATTTATTTAAGA

ATTGTTGTAAATCGTTCGATACGTCCGGTAGTGCCTCGTAATTCTGTTCCGGCGACGGTT

CGGGGTTAAGGGGTGTTACATTTTATGGTATCAGAGCTATCAGGTTTAGCCGATTCTCGG

CCTAAATCGAGCTCGGAATTGAGTCTAGATGTACATGCCACTGTCGAGTTAAACTGAGTC

GGGATTTTTGGATGCTGACCTATTTGTTTGTTTTGTTTTATAGATTAAAGATGTCTGAAG

AAAGAATAAATGATACTGATGAAAGAATGTATAGTGAAGATAGAGAATTAGATGAAACAG

AATCTGTTGCACCGAGTGTGAATCCGTTAGGCAACCAACCTTCTAATGTAGAACGAGAAA

ATGTCAGAGATAGAGATGAATCCCAATTACTGAGAATTATAGCTGATGCATTACAAAGAG

TAGCAGGAACTACTCCTGTTACGACTTCAGTACCTACTGTTAGACGGGCTCCGATAAAGG

AACTGAGGAAATATGGTGCCACTGAATTTATGGGTCTAAAAGGAGTTGATCCATCCATAG

CTGAAAATTGGATGGAGTCGACTAAAAGAATTTTGCAGCAATTGGATTGTACCCCCCGAG

AGTGTTTAATCTGTGCCGTATCGTTATTACAAGGGGAGGCTTATCTATGGTGGGAATCAG

TGGTTCGACATTTACCAGAGAGTCAGATAACGTGGGATCTATTTCAGAAGGAGTTTCAAA

AGAAATATATCGGAGAGATGTATATTGAAGACAAGAAACAAGAGTTTTTGTTGCTACAAC

AGGGTGATATGTCAGTAATAGATTATGAGAGGGAATTCTCGAGACTCAGTAGATATGCCT

CCGAGTTTATTCCGACAGAAGCCGATAGTTGTAAAAGATTTTTACGGGGTTTACGAGACG

AGATCAAAGTGCAGCTAGTATCCCATCGGATCACTGAGTTAGTAGATTTGATTGAACGAG

CTAAAATGGTGGAACAAGTTCTGGGCCTCGACAAAAAGACTGAAGTTGTTAGACCAACCG

GGAAGCGTACAGGAACTACCAGTTCGAATCCTCAGCCGAAAAGACCAAAGGAATTCCAAA

GTGGTTGGAGATCCAGTTTCAGGTCAGACAGAGGTGGTAGAAATAGGGGAAAACAGACGA

TGACATCTACTGGCAGTGTGAAAGGTCCTTCCCGAGAAATAGATATTCCAGACTGCCAAC

ACTGCGGAAAGAAACACAGAGGGGAATGTTGGAAATTAACTAGAGGCTGTTTTCGATGTG

GTTCTACAGACCATTTCATCAGAGACTGTCCGAAAGTTGATAGTACTGTACCCGTGACAT

CACAGAGATCGGTATCTACAGCTAGAGGCAGAGGGTTAGGAAGAGGTGGTTCGGTTTCAA

GGGGAGGAAGTATTAGGAGAAGCAATGATATTGCTACTCAGCAGTCTGAGGCTAAAGTAC

CTGCCAGAGCTTATGTGGTCAGAACACAGGAAGAAGGTGACGCCCACGATGTAGTAACAG

GTATATTCTTACTATATTCTGAGCCTGTTTATGCTTTAATTGATCCCGGATCTTCACATT

CTTATATAAATTCAAAATTAGTTGAATTGGGAAAATTTAATTCTGAAATATCTAGAGTGA

CTGTAGAAGTGTCGAGTCCGTTGGGGCAAACAGTATTAGTGAATCAGATCTGTCCGAGAT

GCCCGTTAATTATACAAAATAAAACTTTTCCTATTGACCTGTTGATTATGCCATTTGGAG

ATTTTGATATAATACTGGGGATGGATTGGTTGGCTGAGCACGGAGTGGTATTGGATTGTT

ATAAAAAGAAGTTTAGTATTCAGACAGAAGACGGGGACAGAATTGAAGTAAATGGTATCC

GTACTAATGGGCCGACACGTATTATTTCGGCAATAAAGGCTAATAAATTGCTTCAGCGGG

GTTGTACAGCGTATTTAGCCTATGTTATTAATTCTGATTTGGTTGGTAGTCAGTGCAGTA

AGATTAGAACCGTATGTGAGTTTCCAGATGTATTTCCTGAAGAGCTACCGGGTTTACCAC

CTGACAGAGAGGTTGAATTTGCTATAGAAGTGTATCCGGGTACAGCACCAATCTCTATAC

CACCGTATCGAATGTCACCCACTGAGTTGAAAGAGTTGAAAGTGCAGTTACAGGACTTGT

CAGATCGTGGATTTATTAGACCGAGCATCTCACCTTGGGGAGCTCCAGTATTGTTTGTTA

AAAAGAAAGATGGATCGATGCGGCTTTGTATTGATTACCGGCAGTTAAACAAAGTGACGA

TCAAGAACCGGTATCCGTTACCCCGTATAGATGATTTATTTGATCAACTAAAAGGAGCTT

CAGTATTTTCAAAGATTGACTTAAGATCTGGGTATTATCAGCTGAAGGTAAAAGAAAGTG

ATGTTCCGAAGACTGCATTTCGTACTCGATATGGTCATTATGAATTTTTGGTGATGCCGT

TCGGGTTGACTAATGCTCCAGCTGCTTTTATGGATCTGATGAATCGTATTTTTCAGCCGT

ATTTAGATCAGTTTGTGGTGGTTTTTATTGATGACATCTTGGTTTATTCGAAGTCAGAGT

CAGAGCATGATCAGCATCTCAGAACCGTGCTACAAATTCTGCGAGAAAAACAGTTGTACG

GGAAACTAAGTAAATGTGAATTCTGGTTATCAGAGGTAGTATTCTTGGGACATGTTGTAT

CTGCGGATGGGATTAGAGTTGATCCGAAGAAGATCGAGGCAATTGTTCAATGGAAGGCAC

CAAAGAATGTATCAGAGGTACGCAGTTTTCTTGGTTTGGCTGGGTATTACAGAAGATTTG

TAAATGGGTTTTCGAAGATAGCTTTGCCGATGACCAAATTACTACAGAAGAATGTTCCAT

TTATCTGGGATGATCAGTGTCAGAGGAGCTTTGAAACATTGAAACAGATGTTGACAGAGG

CACCAGTTTTAACTTTACCAGAATCAGGGAAAGATTTCATAGTGTACAGTGATGCTTCTT

TGAATGGTTTGGGTTGTGTATTGATGCAAGAAGGAAAAGTAATAGCTTATGCATCTCGAC

AGTTGAAGTCACATGAACGCAACTACCCGACACACGATTTAGAGTTAGCTGCTGTAATCT

TTGCATTGAAGATTTGGATACATTACTTGTATGGTGAGAAATGTTATATTTACACTGATC

ATAAAAGTCTAAAATATCTTCTGTCACAAAAGGAGTTGAATCTGAGACAGAGACGGTGGA

TTGAACTTCTGAAAGATTATGATTGTGTTATAGATTATCATCCAGGGAAGGCAAATGTGG

TAGCAGATGCATTGAGTAGAAAAGCAGCGATTGAATTACGAGCAATGTTCGCTCGACTTA

GTATTAAGGATGATGGAAGTTTGTTAGCTGAGTTAAGAGTCAAGCCGGTGATGTTTGATC

AAATCAGAGCAGCACAGTTAAAAGATGAAAAGTTGATGAGGAAAAGAGAAATGGTACAGT

ATGGTGCGGTAGAAAATTTTAGTATTGACGAGCATGATTGTTTGAGATTTCGAAATCGAA

TTTGTGTTCCATCTACTTCTGAGATTAAAGAATTGATTCTCCGAGAAGCACATAATAGTA

TTTTTGCTTTGCACCCAGGAGGAACGAAGATGTATCGTGATCTACGAGAACTGTATTGGT

GGCCAGGAATGAAGAAAGATATAGTTGAATATGTCAGTAAATGCTTGACTTGTCAGCGGG

TAAAAGCAGAACATCAGGTACCAACAGGCCTGTTACAGCCTATTACTATTCCCGAGTGGA

AATGGGATCGCATTACCATGGATTTTGTTACGGGGTTGCCATTGTCAGTGAGTAAAAAGA

ATGCTATTTGGGTGATTGTTGATCGACTCACAAAATCAGCTCATTTTATAGCAGTTAGAA

CCGACTGGTCATTACAGAAGCTTGCCGAGGTTTATATTCGAGAAATTGTTAGATTACATG

GTATTCCGGTATCAATAATTTCAGACAGAGATCCTCGATTCACTTCGAGATTTTGGAAGC

AGCTGCATGAATCATTGGGTACTCGACTTAGTTTCAGTACAGCTTTTCATCCTCAAACTG

ATGGACAATCTGAACGAGTAATTCAGATATTAGAAGATATGCTTCGAGCTTGTGTCATTG

ATTTTGAATCAGGTTGGGAACGTTATTTACCATTGGCCGAGTTTGTTTATAATAATAGTT

TCCAATCTAGTATTCAAATGGCTCCATATGAAGCACTTTATGGTCGAAGGTGTCGATCAC

CAATATGTTGGACAAAATTAAGAGAAAGAAAAGTGATTGGGCCGGAATTGATTCAAGAGA

CAGAAGAAACAGTTAAAAAGATTAAAGATAGACTGAAAGCCGCTTTCGACAGACAGAAAT

CTTACGCAGACTTGAAACGACGAGACATTGAATATTCCGTTGGTGATAAGGTATTCCTCA

AAGTATCGCCGTGGAAGAAAATTTTGAGATTTGGTCGGAAGGGAAAATTAAGTCCGCGCT

TTATTGGGCCGTATGAGATAGTGGAAAGAATTGGGCCTGTTGCTTATCGATTATCCTTAC

CTCCAGAGTTACAGAAAATTCATGATGTTTTTCATGTTTCGATGCTTCGGAGATATAGAT

CGGATCCTTCTCATGTTATTCCCACTGAAGACATTGAACTTCGATCTGATTTAACTTATG

AAGAAGAACCAGTTCAAATATTAGCACGAGAAGTGAAAGAATTAAGAAATAAACGGGTTC

CTTTAGTACAAGTTTTATGGAGAAGCCATAGTGTGGAAGAAGCAACTTGGGAACCGGAAG

AGACAATGAGAGCACAATATCCTCATCTCTTCTCAGGTAAATTTCGAGGACGAAATTTAT

TAAGAGGGGGAGAAATGTAATGACCTAAAATTCATGGGCATCGGAAAAGTATAATATTGG

GCCTCCGTCCTAGTAAATTGAGTCCGAAAATAATTATTAGAAATATTTACGAGACTAGTA

GTGTGTTTAATTAGGTTTTAATTAAGTAAATTTAGCTTAATTTAGAGTAATTAGTAAAAA

GGATTAAATTGAATAAGAGTAAAAGTTTAATTATAGATTAAAGGAAAATAATAGGGACCA

AATGGGCAATTAAGCCACATTTGGAAGTTGAGGCGGCATAACATTGTAAAAATCTTAGAT

TTTTATATTATTATTTATATAAATATATAAATTAATTATAAAGTATATTATTAAATTAAT

TATATTATAAATATTATATTATTATATATAAAAGAAACAAAACAGAAAAGAAACAGAATA

GAAAGAACAAAGAAACAGAATAGAAGAGACGAAACAGGGGAGAAGCAGGGGAGAAAGAAG

AAAAAGAAGAAAAAAGGGGAAATAGGGTTTTTGAAGCTTGAAATTTAAATTGGTAAGTCA

AATTAGCCATTTTCTCTTAATTCTAATGTTTTAAAAGCTTTAAAACAAAGTTTTGATGGA

ATTAAGTTGATATTTTGTAAGTTCATAGGTTTTCAAGTATAGTTTATGTTGAACAAAAGA

GATGAATTAGGGATTAACTTGAAGGAATTTTAAGTTAGAATTGAAAAAGGGATTAAATTG

TAAAAGAAACTATAAGTTTTTTTTGTTTTAGGGACTAGATTGAGGAAAATTCGGAATTAA

GAAAATATGTTAAAAATTTAATAGTTAAATTTGAGTTTAAATGAAATTTGAATAGGAATA

AGGTGTGAATTGGTGTTATAAATTTGGTTATTAACATTTTTAATCAAAACAGTTTTGGGA

AGTAGCAATGGTCTGACTTTGAAAATTCACTAAAAATTTTATAAATTGAACTAGAGGATG

AACAAAATATGGAATTAAAGCTTATTGAGTCTAGTTTCTTATAGTAGAAACAATGTAAGC

AATTAATTGATGAATCAAGAGATATTTGAAATTTTGTAATACTGGTTCGGGGTGATTTCG

AGATGCCCTGTTTTAACTTTGGAAAATCATTAAAAATTGTACAAAAATTATTATGGAGTG

TAATTTATATATGTAAACTCCTTAATGAATCTAGTTTCAAAATAAATAAACAAGAACCTT

ATTCGAGTTCTGTACAATGAGATAATTTAGTTTTAGTGGAGAGAGGTCAGAACTGTCAAA

TGAAATAACAGGGGAGTATTTAACGAATAAACTGTATTAAATGGCTAGACCAAAAATTCT

GGAAATTTTATGATTAGAAGATATATGAGTCTAGTTTTAAGGAAAATTTACGGATATTAA

TTTGGAGTTTCGTAGCTCAAGATATAAATAATTTAGTAACAATGACCCAAGTAGACAGCT

TAATGGTGAAATTATATAAATACATTAAAAATGGTTAAATTTGCATGTTTAGGCTCATGA

ATTAAATTGAATCATGTTGTATTGATTATTATAAATTATTATTTTCGTAGCCAACAAAGA

ACCTAAAGCATCAGCATCGAAAGGAAAGGAGAAAGTCATCGAGGAGTAAACTCGAGAAAA

TTACGGTTTGTATTACTATAATTCAAGTTATTTATTATTAAATGTTAAATTTTAATTTAT

GTGTCTAGTAAATGAAATGTGAGGTAAGTATTATTATTATTATTATTATTATTATTATTA

TTATTATTATTATTATTATGAGTGGGAATTAAATTGAATAGTTGATATGAAATAATATTT

GAATTGTTTGTTGATTGAAAGCGGGAAATGAATTTAAATCGAATAGTGACCGATATTAAA

TTGAATGGAAATGTATTGAGTTGTGAAAATATGTTAATTGCGGATTAATTATTGATTGAA

AGGTGGAAAAATGATTGAATTGAAAGTGTGAGAAAGTGTGATTGAATTGGGATTATATGT

GATTTAAATACCCTATTAACTAGTCGGGCTGAGTCGGATATAGTTGGCATGCCATAGGAT

TGGAAGAGTTCAGGGATACTTCGACCTCGAGTCGATGAGACACTGGGTGTCACTATATTT

CTTCGGATAGATTCGATGAGGTACTGGGTACCAACTTTCTTCGGCTTTGCCGATGAGACA

CTGGGTGTCAACTATTGCTTCGAACTATCCGATGAGGCACTGGGTGCCATTCTGGTGTGT

TTGGTTGGATCCGTGTATTCGCCAAAGTCCGAGTTTTGTTAATAGGGTAAATGATGAAAT

GATAAACCGAACGAGTTGGTCAAACGAGCTATTGAAATGATATGAAAAAGTTGAATTGTG

AATTGAAATGTGAAATGAGATTGAGAAATGAACCTAAGGTTCGTGAATTATTCAAACTCA

AATTGTGGATATACGATATTGGTTGATGAATTGCTATTGTTGAAATATTTAATTTAAATT

GTATATACGATTTATGCTTTACATGTACATTATTGTTATAATTTGAATTATGGTAATACC

ACTGAGTATGAATTACTCAGCGTACGGTTGTTTCCGTGCGCAGGTCAATAGAAGTCAAAG

GTCTCGGTTCAGCATCCAGATTAATCCCGGCTTCGGCAAAACTTGGTGATGTATTTTTCC

TTTGGTAAAGGTGGCATGTACATAGATTGTGTATAAAGGTTATTATGTTTTATTATATAA

TGGTTAAAAATGTTAGTATTAAAAGTTTATGGATTTTAATGAAAGAAGTCTATCTATTTT

ATCTAATTAGTACATTGTTAAATTTTAAATTGGTATTAGATTGAGTTTGATTAGAAGTAT

TTAGAATAGAAAATGTGAATGTGAAATGAATTGGTTGAATTGATGATATTTGGGAACTAT

ATGGTTTTAATTTGC

>AHA6-1-4

ATGTAATGACCCAAAATTCATGGGCATCGGAAAAGTATAATATCGGGCCTCCGTCCTAGT

AAATTGAGTCCGAAAATAATTATTAGAAATATTTACGAGACTAGTAGTGTGTTTAATTAG

GTTTTAATTAAGTAAATTTAGCTTAATTTAGAGTAATTAGTAAAAAGGATTAAATTGAAT

AAGAGTAAAAGTTTAATTATAGATTAAAGGAAAATAATAGGGACCAAATGGGCAATTAAG

CCACATTTGGAAGTTGAGGCGGCATAACATTGTAAAAATCTTAGATTTTTATATTATTAT

TTATATAAATATATAAATTAATTATAAAGTATATTATTAAATTAATTATATTATAAATAT

TATATTATTATATATAAAAGAAACAAAACAGAAAAGAAACAGAATAGAAAGAACAAAGAA

ACAGAATAGAAGAGACGAAACAGGGGAGAAGCAGGGGAGAAAGAAGAAAAAGAAGAAAAA

AGGGGAAATAGGGTTTTTGAAGCTTGAAATTTAAATTGGTAAGTCAAATTAGCCATTTTC

TCTTAATTCTAATGTTTTAAAAGCTTTAAAACAAAGTTTTGATGGAATTAAGTTGATATT

TTGTAAGTTCATAGGTTTTCAAGTATAGTTTATGTTGAACAAAAGAGATGAATTAGGGAT

TAACTTGAAGGAATTTTAAGTTAGAATTGAAAAAGGGATTAAATTGTAAAAGAAACTATA

AGTTTTTTTTGTTTTAGGGACTAGATTGAGGAAAATTCGGAATTAAGAAAATATGTTAAA

AATTTAATAGTTAAATTTGAGTTTAAATGAAATTTGAATAGGAATAAGGTGTGAATTGGT

GTTATAAATTTGGTTATTAACATTTTTAATCAAAACAGTTTTGGGAAGTAGCAATGGTCT

GACTTTGAAAATTCACTAAAAATTTTATAAATTGAACTAGAGGATGAACAAAATATGGAA

TTAAAGCTTATTGAGTCTAGTTTCTTATAGTAGAAACAATGTAAGCAATTAATTGATGAA

TCAAGAGATATTTGAAATTTTGTAATACTGGTTCGGGGTGATTTCGAGATGCCCTGTTTT

AACTTTGGAAAATCATTAAAAATTGTACAAAAATTATTATGGAGTGTAATTTATATATGT

GAACTCCTTAATGAATCTAGTTTCAAAATAAATAAACAAGAACCTTATTCGAGTTCTGTA

CAATGAGATAATTTAGTTTTAGTGGAGAGAGGTCAGAACTGTCAAATGAAATAACAGGGG

AGTATTTAACGAATAAACTGTATTAAATGGCTAGACCAAAAATTCTGGAAATTTTATGAT

TAGAAGATATATGAGTCTAGTTTTAAGGAAAATTTACGGATATTAATTTGGAGTTTCGTA

GCTCAAGATATAAATAATTTAGTAACAATGACCCAAGTAGACAGCTTAATGGTGAAATTA

TATAAATACATTAAAAATGGTTAAATTTGCATGTTTAGGCTCATGAATTAAATTGAATCA

TGTTGTATTGATTATTATAAATTATTATTTTCGTAGCCAACAAAGAACCTAAAGCATCAG

CATCGAAAGGAAAGGAGAAAGTCATCGAGGAGTAAACTCGAGAAAATTACGGTTTGTATT

ACTATAATTCAAGTTATTTATTATTAAATGTTAAATTTTAATTTATGTGTCTAGTAAATG

AAATGTGAGGTAAGTATTATTATTATTATTATTATTATTATTATTATTATTATTATTATT

ATGAGTGGGAATTAAATTGAATAGTTGATATGAAATAATATTTGAATTGTTTGTTGATTG

AAAGCGGGAAATGAATTTAAATCGAATAGTGACCGATATTAAATTGAATGGAAATGTATT

GAGTTGTGAAAATATGTTAATTGCGGATTAATTATTGATTGAAAGGTGGAAAAATGATTG

AATTGAAAGTGTGAGAAAGTGTGATTGAATTGGGATTATATGTGATTTAAATACCCTATT

AACTAGTCGGGCTGAGTCGGATATAGTTGGCATGCCATAGGATTGGAAGAGTTCAGGGAT

ACTTCGACCTCGAGTCGATGAGACACTGGGTGATTTCTTCGGATAGATTGGATGAGGTAC

TGGGTACCAACTTTCTTCGGCTTTGCCGATGAGACACTGGGTGTCAACTATTGCTTCGAA

CTATCCGATGAGGCACTGGGTGCCATTCTGGTGTGTTTGGTTGGATCCGTGTATCCGCCA

AAGTCCGAGTTTTGTTAATAGGGTAAATGATGAAATGATAAACCGAACGAGTTGGTCAAA

CGAGCTATTGAAATGATATGAAAAAGTTGAATTGTGAATTGAAATGTGAAATGAGATTGA

GAAATGAACCTAAGGTTCGTGAATTATTCAAACTCAAATTGTGGATATACGATATTGGTT

GATGAATTGCTATTGTTGAAATATTTAATTTAAATTGTATATACGATTTATGCTTTACAT

GTACATTATTGTTATAATTTGAATTATGGTAATACCACTGAGTATGAATTACTCAGCGTA

CGGTTGTTTCCGTGCGCAGGTCAATAGAAGTCAAAGGTCTCGGTTCAGCATCCAGATTAA

TCCCGGCTTCGGCAAAACTTGGTGATGTATTTTTCCTTTGGTAAAGGTGGCATGTACATA

GATTGTGTATAAAGGTTATTATGTTTTATTATATAATGGTTAAAAATGTTAGTATTAAAA

GTTTATGGATTTTAATGAAAGAAGTCTATCTATTTTATCTAATTAGTACATTGTTAAATT

TTAAATTGGTATTGTGTAGATTGAGTTTGATTAGAAGTATTTAGAATAGAAAATGTGAAT

GTGAAATGAATTGGTTGAATTGATGATATTTGGGAACTATATGGTTTTAATTTGCAGGGG

GTTTTATGTAAAAATAAGCAGAAATGCTGCCGAAATTTTTATAAAAAAAAATGAAGTCAT

TTGGTAAACAAATTAATAAATTTTATGAATTATTTTAATATATTGGTTATTTATTTAAGA

ATTGTTGTAAATCGTTCGATACGTCCGGTAGTGCCTCGTAATTCTGTTCCGGCGACGGTT

CGGGGTTAAGGGGTGTTACATTTTATGGTATCAGAGCTATCAGGTTTAGCCGATTCTCGG

CCTAAATCGAGCTCGGAATTGAGTCTAGATGTACATGCCACTGTCGAGTTAAACTGAGTC

GGGATTTTTGGATGCTGACCTATTTGTTTGTTTTGTTTTATAGATTAAAGATGTCTGAAG

AAAGAATAAATGATACTGATGAAAGAATGTATAGTGAAGATAGAGAATTAGATGAAACAG

AATCTGTTGCACCGAGTGTGAATCCGTTAGGCAACCAACCTTCTAATGTAGAACGAGAAA

ATGTCAGAGATAGAGATGAATCCCAATTACTGAGAATTATAGCTGATGCATTACAAAGAG

TAGCAGGAACTACTCCTGTTACGACTTCAGTACCTACTGTTAGACGGGCTCCGATAAAGG

AACTGAGGAAATATGGTGCCACTGAATTTATGGGTCTAAAAGGAGTTGATCCATCCATAG

CTGAAAATTGGATGGAGTCGACTAAAAGAATTTTGCAGCAATTGGATTGTACCCCCCGAG

AGTGTTTAATCTGTGCCGTATCGTTATTACAAGGGGAGGCTTATCTATGGTGGGAATCAG

TGGTTCGACATTTACCAGAGAGTCAGATAACGTGGGATCTATTTCAGAAGGAGTTTCAAA

AGAAATATATCGGAGAGATGTATATTGAAGACAAGAAACAAGAGTTTTTGTTGCTACAAC

AGGGTGATATGTCAGTAATAGATTATGAGAGGGAATTCTCGAGACTCAGTAGATATGCCT

CCGAGTTTATTCCGACAGAAGCCGATAGTTGTAAAAGATTTTTACGGGGTTTACGAGACG

AGATCAAAGTGCAGCTAGTATCCCATCGGATCACTGAGTTAGTAGATTTGATTGAACGAG

CTAAAATGGTGGAACAAGTTCTGGGCCTCGACAAAAAGACTGAAGTTGTTAGACCAACCG

GGAAGCGTACAGGAACTACCAGTTCGAATCCTCAGCCGAAAAGACCAAAGGAATTCCAAA

GTGGTTGGAGATCCAGTTTCAGGTCAGACAGAGGTGGTAGAAATAGGGGAAAACAGACGA

TGACATCTACTGGCAGTGTGAAAGGTCCTTCCCGAGAAATAGATATTCCAGACTGCCAAC

ACTGCGGAAAGAAACACAGAGGGGAATGTTGGAAATTAACTAGAGGCTGTTTTCGATGTG

GTTCTACAGACCATTTCATCAGAGACTGTCCGAAAGTTGATAGTACTGTACCCGTGACAT

CACAGAGATCGGTATCTACAGCTAGAGGCAGAGGGTTAGGAAGAGGTGGTTCGGTTTCAA

GGGGAGGAAGTATTAGGAGAAGCAATGATATTGCTACTCAGCAGTCTGAGGCTAAAGTAC

CTGCCAGAGCTTATGTGGTCAGAACACAGGAAGAAGGTGACGCCCACGATGTAGTAACAG

GTATATTCTTACTATATTCTGAGCCTGTTTATGCTTTAATTGATCCCGGATCTTCACATT

CTTATATAAATTCAAAATTAGTTGAATTGGGAAAATTTAATTCTGAAATATCTAGAGTGA

CTGTAGAAGTGTCGAGTCCGTTGGGGCAAACAGTATTAGTGAATCAGATCTGTCCGAGAT

GCCCGTTAATTATACAAAATAAAACTTTTCCTATTGACCTGTTGATTATGCCATTTGGAG

ATTTTGATATAATACTGGGGATGGATTGGTTGGCTGAGCACGGAGTGGTATTGGATTGTT

ATAAAAAGAAGTTTAGTATTCAGACAGAAGACGGGGACAGAATTGAAGTAAATGGTATCC

GTACTAATGGGCCGACACGTATTATTTCGGCAATAAAGGCTAATAAATTGCTTCAGCGGG

GTTGTACAGCGTATTTAGCCTATGTTATTAATTCTGATTTGGTTGGTAGTCAGTGCAGTA

AGATTAGAACCGTATGTGAGTTTCCAGATGTATTTCCTGAAGAGCTACCGGGTTTACCAC

CTGACAGAGAGGTTGAATTTGCTATAGAAGTGTATCCGGGTACAGCACCAATCTCTATAC

CACCGTATCGAATGTCACCCACTGAGTTGAAAGAGTTGAAAGTGCAGTTACAGGACTTGT

CAGATCGTGGATTTATTAGACCGAGCATCTCACCTTGGGGAGCTCCAGTATTGTTTGTTA

AAAAGAAAGATGGATCGATGCGGCTTTGTATTGATTACCGGCAGTTAAACAAAGTGACGA

TCAAGAACCGGTATCCGTTACCCCGTATAGATGATTTATTTGATCAACTAAAAGGAGCTT

CAGTATTTTCAAAGATTGACTTAAGATCTGGGTATTATCAGCTGAAGGTAAAAGAAAGTG

ATGTTCCGAAGACTGCATTTCGTACTCGATATGGTCATTATGAATTTTTGGTGATGCCGT

TCGGGTTGACTAATGCTCCAGCTGCTTTTATGGATCTGATGAATCGTATTTTTCAGCCGT

ATTTAGATCAGTTTGTGGTGGTTTTTATTGATGACATCTTGGTTTATTCGAAGTCAGAGT

CAGAGCATGATCAGCATCTCAGAACCGTGCTACAAATTCTGCGAGAAAAACAGTTGTACG

GGAAACTAAGTAAATGTGAATTCTGGTTATCAGAGGTAGTATTCTTGGGACATGTTGTAT

CTGCGGATGGGATTAGAGTTGATCCGAAGAAGATCGAGGCAATTGTTCAATGGAAGGCAC

CAAAGAATGTATCAGAGGTACGCAGTTTTCTTGGTTTGGCTGGGTATTACAGAAGATTTG

TAAATGGGTTTTCGAAGATAGCTTTGCCGATGACCAAATTACTACAGAAGAATGTTCCAT

TTATCTGGGATGATCAGTGTCAGAGGAGCTTTGAAACATTGAAACAGATGTTGACAGAGG

CACCAGTTTTAACTTTACCAGAATCAGGGAAAGATTTCATAGTGTACAGTGATGCTTCTT

TGAATGGTTTGGGTTGTGTATTGATGCAAGAAGGAAAAGTAATAGCTTATGCATCTCGAC

AGTTGAAGTCACATGAACGCAACTACCCGACACACGATTTAGAGTTAGCTGCTGTAATCT

TTGCATTGAAGATTTGGATACATTACTTGTATGGTGAGAAATGTTATATTTACACTGATC

ATAAAAGTCTAAAATATCTTCTGTCACAAAAGGAGTTGAATCTGAGACAGAGACGGTGGA

TTGAACTTCTGAAAGATTATGATTGTGTTATAGATTATCATCCAGGGAAGGCAAATGTGG

TAGCAGATGCATTGAGTAGAAAAGCAGCGATTGAATTACGAGCAATGTTCGCTCGACTTA

GTATTAAGGATGATGGAAGTTTGTTAGCTGAGTTAAGAGTCAAGCCGGTGATGTTTGATC

AAATCAGAGCAGCACAGTTAAAAGATGAAAAGTTGATGAGGAAAAGAGAAATGGTACAGT

ATGGTGCGGTAGAAAATTTTAGTATTGACGAGCATGATTGTTTGAGATTTCGAAATCGAA

TTTGTGTTCCATCTACTTCTGAGATTAAAGAATTGATTCTCCGAGAAGCACATAATAGTA

TTTTTGCTTTGCACCCAGGAGGAACGAAGATGTATCGTGATCTACGAGAACTGTATTGGT

GGCCAGGAATGAAGAAAGATATAGTTGAATATGTCAGTAAATGCTTGACTTGTCAGCGGG

TAAAAGCAGAACATCAGGTACCAACAGGCCTGTTACAGCCTATTACTATTCCCGAGTGGA

AATGGGATCGCATTACCATGGATTTTGTTACGGGGTTGCCATTGTCAGTGAGTAAAAAGA

ATGCTATTTGGGTGATTGTTGATCGACTCACAAAATCAGCTCATTTTATAGCAGTTAGAA

CCGACTGGTCATTACAGAAGCTTGCCGAGGTTTATATTCGAGAAATTGTTAGATTACATG

GTATTCCGGTATCAATAATTTCAGACAGAGATCCTCGATTCACTTCGAGATTTTGGAAGC

AGCTGCATGAATCATTGGGTACTCGACTTAGTTTCAGTACAGCTTTTCATCCTCAAACTG

ATGGACAATCTGAACGAGTAATTCAGATATTAGAAGATATGCTTCGAGCTTGTGTCATTG

ATTTTGAATCAGGTTGGGAACGTTATTTACCATTGGCCGAGTTTGTTTATAATAATAGTT

TCCAATCTAGTATTCAAATGGCTCCATATGAAGCACTTTATGGTCGAAGGTGTCGATCAC

CAATATGTTGGACAAAATTAAGAGAAAGAAAAGTGATTGGGCCGGAATTGATTCAAGAGA

CAGAAGAAACAGTTAAAAAGATTAAAGATAGACTGAAAGCCGCTTTCGACAGACAGAAAT

CTTACGCAGACTTGAAACGACGAGACATTGAATATTCCGTTGGTGATAAGGTATTCCTCA

AAGTATCGCCGTGGAAGAAAATTTTGAGATTTGGTCGGAAGGGAAAATTAAGTCCGCGCT

TTATTGGGCCGTATGAGATAGTGGAAAGAATTGGGCCTGTTGCTTATCGATTATCCTTAC

CTCCAGAGTTACAGAAAATTCATGATGTTTTTCATGTTTCGATGCTTCGGAGATATAGAT

CGGATCCTTCTCATGTTATTCCCACTGAAGACATTGAACTTCGATCTGATTTAACTTATG

AAGAAGAACCAGTTCAAATATTAGCACGAGAAGTGAAAGAATTAAGAAATAAACGGGTTC

CTTTAGTACAAGTTTTATGGAGAAGCCATAGTGTGGAAGAAGCAACTTGGGAACCGGAAG

AGACAATGAGAGCACAATATCCTCATCTCTTCTCAGGTAAATTTCGAGGACGAAATTTAT

TAAGAGGGGGAGAAATGTAATGACCTAAAATTCATGGGCATCGGAAAAGTATAATATTGG

GCCTCCGTCCTAGTAAATTGAGTCCGAAAATAATTATTAGAAATATTTACGAGACTAGTA

GTGTGTTTAATTAGGTTTTAATTAAGTAAATTTAGCTTAATTTAGAGTAATTAGTAAAAA

GGATTAAATTGAATAAGAGTAAAAGTTTAATTATAGATTAAAGGAAAATAATAGGGACCA

AATGGGCAATTAAGCCACATTTGGAAGTTGAGGCGGCATAACATTGTAAAAATCTTAGAT

TTTTATATTATTATTTATATAAATATATAAATTAATTATAAAGTATATTATTAAATTAAT

TATATTATAAATATTATATTATTATATATAAAAGAAACAAAACAGAAAAGAAACAGAATA

GAAAGAACAAAGAAACAGAATAGAAGAGACGAAACAGGGGAGAAGCAGGGGAGAAAGAAG

AAAAAGAAGAAAAAAGGGGAAATAGGGTTTTTGAAGCTTGAAATTTAAATTGGTAAGTCA

AATTAGCCATTTTCTCTTAATTCTAATGTTTTAAAAGCTTTAAAACAAAGTTTTGATGGA

ATTAAGTTGATATTTTGTAAGTTCATAGGTTTTCAAGTATAGTTTATGTTGAACAAAAGA

GATGAATTAGGGATTAACTTGAAGGAATTTTAAGTTAGAATTGAAAAAGGGATTAAATTG

TAAAAGAAACTATAAGTTTTTTTTGTTTTAGGGACTAGATTGAGGAAAATTCGGAATTAA

GAAAATATGTTAAAAATTTAATAGTTAAATTTGAGTTTAAATGAAATTTGAATAGGAATA

AGGTGTGAATTGGTGTTATAAATTTGGTTATTAACATTTTTAATCAAAACAGTTTTGGGA

AGTAGCAATGGTCTGACTTTGAAAATTCACTAAAAATTTTATAAATTGAACTAGAGGATG

AACAAAATATGGAATTAAAGCTTATTGAGTCTAGTTTCTTATAGTAGAAACAATGTAAGC

AATTAATTGATGAATCAAGAGATATTTGAAATTTTGTAATACTGGTTCGGGGTGATTTCG

AGATGCCCTGTTTTAACTTTGGAAAATCATTAAAAATTGTACAAAAATTATTATGGAGTG

TAATTTATATATGTAAACTCCTTAATGAATCTAGTTTCAAAATAAATAAACAAGAACCTT

ATTCGAGTTCTGTACAATGAGATAATTTAGTTTTAGTGGAGAGAGGTCAGAACTGTCAAA

TGAAATAACAGGGGAGTATTTAACGAATAAACTGTATTAAATGGCTAGACCAAAAATTCT

GGAAATTTTATGATTAGAAGATATATGAGTCTAGTTTTAAGGAAAATTTACGGATATTAA

TTTGGAGTTTCGTAGCTCAAGATATAAATAATTTAGTAACAATGACCCAAGTAGACAGCT

TAATGGTGAAATTATATAAATACATTAAAAATGGTTAAATTTGCATGTTTAGGCTCATGA

ATTAAATTGAATCATGTTGTATTGATTATTATAAATTATTATTTTCGTAGCCAACAAAGA

ACCTAAAGCATCAGCATCGAAAGGAAAGGAGAAAGTCATCGAGGAGTAAACTCGAGAAAA

TTACGGTTTGTATTACTATAATTCAAGTTATTTATTATTAAATGTTAAATTTTAATTTAT

GTGTCTAGTAAATGAAATGTGAGGTAAGTATTATTATTATTATTATTATTATTATTATTA

TTATTATTATTATTATTATGAGTGGGAATTAAATTGAATAGTTGATATGAAATAATATTT

GAATTGTTTGTTGATTGAAAGCGGGAAATGAATTTAAATCGAATAGTGACCGATATTAAA

TTGAATGGAAATGTATTGAGTTGTGAAAATATGTTAATTGCGGATTAATTATTGATTGAA

AGGTGGAAAAATGATTGAATTGAAAGTGTGAGAAAGTGTGATTGAATTGGGATTATATGT

GATTTAAATACCCTATTAACTAGTCGGGCTGAGTCGGATATAGTTGGCATGCCATAGGAT

TGGAAGAGTTCAGGGATACTTCGACCTCGAGTCGATGAGACACTGGGTGTCACTATATTT

CTTCGGATAGATTCGATGAGGTACTGGGTACCAACTTTCTTCGGCTTTGCCGATGAGACA

CTGGGTGTCAACTATTGCTTCGAACTATCCGATGAGGCACTGGGTGCCATTCTGGTGTGT

TTGGTTGGATCCGTGTATTCGCCAAAGTCCGAGTTTTGTTAATAGGGTAAATGATGAAAT

GATAAACCGAACGAGTTGGTCAAACGAGCTATTGAAATGATATGAAAAAGTTGAATTGTG

AATTGAAATGTGAAATGAGATTGAGAAATGAACCTAAGGTTCGTGAATTATTCAAACTCA

AATTGTGGATATACGATATTGGTTGATGAATTGCTATTGTTGAAATATTTAATTTAAATT

GTATATACGATTTATGCTTTACATGTACATTATTGTTATAATTTGAATTATGGTAATACC

ACTGAGTATGAATTACTCAGCGTACGGTTGTTTCCGTGCGCAGGTCAATAGAAGTCAAAG

GTCTCGGTTCAGCATCCAGATTAATCCCGGCTTCGGCAAAACTTGGTGATGTATTTTTCC

TTTGGTAAAGGTGGCATGTACATAGATTGTGTATAAAGGTTATTATGTTTTATTATATAA

TGGTTAAAAATGTTAGTATTAAAAGTTTATGGATTTTAATGAAAGAAGTCTATCTATTTT

ATCTAATTAGTACATTGTTAAATTTTAAATTGGTATTAGATTGAGTTTGATTAGAAGTAT

TTAGAATAGAAAATGTGAATGTGAAATGAATTGGTTGAATTGATGATATTTGGGAACTAT

ATGGTTTTAATTTGC

>AII_Tex7A21

AGGTAATGACCCAAAATTCATGGGCATCGGAAAAGTATAATATCGGGCCTCCGTCCTAGT

AAATTGAGTCCGAAAATAATTATTAGAAATATTTACGAGACTAGTAGTGTGTTTAATTAG

GTTTTAATTAAGTAAATTTAGCTTAATTTAGAGTAATTAGTAAAAAGGATTAAATTGAAT

AAGAGTAAAAGTTTAATTATAGATTAAAGGAAAATAATAGGGACCAAATGGGCAATTAAG

CCACATTTGGAAGTTGAGGCGGCATAACATTGTAAAAATCTTAGATTTTTATATTATTAT

TTATATAAATATATAAATTAATTATAAAGTATATTATTAAATTAATTATATTATAAATAT

TATATTATTATATATAAAAGAAACAAAACAGAAAAGAAACAGAATAGAAAGAACAAAGAA

ACAGAATAGAAGAGACGAAACAGGGGAGAAGCAGGGGAGAAAGAAGAAAAAGAAGAAAAA

AGGGGAAATAGGGTTTTTGAAGCTTGAAATTTAAATTGGTAAGTCAAATTAGCCATTTTC

TCTTAATTCTAATGTTTTAAAAGCTTTAAAACAAAGTTTTGATGGAATTAAGTTGATATT

TTGTAAGTTCATAGGTTTTCAAGTATAGTTTATGTTGAACAAAAGAGATGAATTAGGGAT

TAACTTGAAGGAATTTTAAGTTAGAATTGAAAAAGGGATTAAATTGTAAAAGAAACTATA

AGTTTTTTTTGTTTTAGGGACTAGATTGAGGAAAATTCGGAATTAAGAAAATATGTTAAA

AATTTAATAGTTAAATTTGAGTTTAAATGAAATTTGAATAGGAATAAGGTGTGAATTGGT

GTTATAAATTTGGTTATTAACATTTTTAATCAAAACAGTTTTGGGAAGTAGCAATGGTCT

GACTTTGAAAATTCACTAAAAATTTTATAAATTGAACTAGAGGATGAACAAAATATGGAA

TTAAAGCTTATTGAGTCTAGTTTCTTATAGTAGAAACAATGTAAGCAATTAATTGATGAA

TCAAGAGATATTTGAAATTTTGTAATACTGGTTCGGGGTGATTTCGAGATGCCCTGTTTT

AACTTTGGAAAATCATTAAAAATTGTACAAAAATTATTATGGAGTGTAATTTATATATGT

GAACTCCTTAATGAATCTAGTTTCAAAATAAATAAACAAGAACCTTATTCGAGTTCTGTA

CAATGAGATAATTTAGTTTTAGTGGAGAGAGGTCAGAACTGTCAAATGAAATAACAGGGG

AGTATTTAACGAATAAACTGTATTAAATGGCTAGACCAAAAATTCTGGAAATTTTATGAT

TAGAAGATATATGAGTCTAGTTTTAAGGAAAATTTACGGATATTAATTTGGAGTTTCGTA

GCTCAAGATATAAATAATTTAGTAACAATGACCCAAGTAGACAGCTTAATGGTGAAATTA

TATAAATACATTAAAAATGGTTAAATTTGCATGTTTAGGCTCATGAATTAAATTGAATCA

TGTTGTATTGATTATTATAAATTATTATTTTCGTAGCCAACAAAGAACCTAAAGCATCAG

CATCGAAAGGAAAGGAGAAAGTCATCGAGGAGTAAACTCGAGAAAATTACGGTTTGTATT

ACTATAATTCAAGTTATTTATTATTAAATGTTAAATTTTAATTTATGTGTCTAGTAAATG

AAATGTGAGGTAAGTATTATTATTATTATTATTATTATTATTATTATTATTATTATTATT

ATGAGTGGGAATTAAATTGAATAGTTGATATGAAATAATATTTGAATTGTTTGTTGATTG

AAAGCGGGAAATGAATTTAAATCGAATAGTGACCGATATTAAATTGAATGGAAATGTATT

GAGTTGTGAAAATATGTTAATTGCGGATTAATTATTGATTGAAAGGTGGAAAAATGATTG

AATTGAAAGTGTGAGAAAGTGTGATTGAATTGGGATTATATGTGATTTAAATACCCTATT

AACTAGTCGGGCTGAGTCGGATATAGTTGGCATGCCATAGGATTGGAAGAGTTCAGGGAT

ACTTCGACCTCGAGTCGATGAGACACTGGGTGATTTCTTCGGATAGATTGGATGAGGTAC

TGGGTACCAACTTTCTTCGGCTTTGCCGATGAGACACTGGGTGTCAACTATTGCTTCGAA

CTATCCGATGAGGCACTGGGTGCCATTCTGGTGTGTTTGGTTGGATCCGTGTATCCGCCA

AAGTCCGAGTTTTGTTAATAGGGTAAATGATGAAATGATAAACCGAACGAGTTGGTCAAA

CGAGCTATTGAAATGATATGAAAAAGTTGAATTGTGAATTGAAATGTGAAATGAGATTGA

GAAATGAACCTAAGGTTCGTGAATTATTCAAACTCAAATTGTGGATATACGATATTGGTT

GATGAATTGCTATTGTTGAAATATTTAATTTAAATTGTATATACGATTTATGCTTTACAT

GTACATTATTGTTATAATTTGAATTATGGTAATACCACTGAGTATGAATTACTCAGCGTA

CGGTTGTTTCCGTGCGCAGGTCAATAGAAGTCAAAGGTCTCGGTTCAGCATCCAGATTAA

TCCCGGCTTCGGCAAAACTTGGTGATGTATTTTTCCTTTGGTAAAGGTGGCATGTACATA

GATTGTGTATAAAGGTTATTATGTTTTATTATATAATGGTTAAAAATGTTAGTATTAAAA

GTTTATGGATTTTAATGAAAGAAGTCTATCTATTTTATCTAATTAGTACATTGTTAAATT

TTAAATTGGTATTGTGTAGATTGAGTTTGATTAGAAGTATTTAGAATAGAAAATGTGAAT

GTGAAATGAATTGGTTGAATTGATGATATTTGGGAACTATATGGTTTTAATTTGCAGGGG

GTTTTATGTAAAAATAAGCAGAAATGCTGCCGAAATTTTTATAAAAAAAAATGAAGTCAT

TTGGTAAACAAATTAATAAATTTTATGAATTATTTTAATATATTGGTTATTTATTTAAGA

ATTGTTGTAAATCGTTCGATACGTCCGGTAGTGCCTCGTAATTCTGTTCCGGCGACGGTT

CGGGGTTAAGGGGTGTTACATTTTATGGTATCAGAGCTATCAGGTTTAGCCGATTCTCGG

CCTAAATCGAGCTCGGAATTGAGTCTAGATGTACATGCCACTGTCGAGTTAAACTGAGTC

GGGATTTTTGGATGCTGACCTATTTGTTTGTTTTGTTTTATAGATTAAAGATGTCTGAAG

AAAGAATAAATGATACTGATGAAAGAATGTATAGTGAAGATAGAGAATTAGATGAAACAG

AATCTGTTGCACCGAGTGTGAATCCGTTAGGCAACCAACCTTCTAATGTAGAACGAGAAA

ATGTCAGAGATAGAGATGAATCCCAATTACTGAGAATTATAGCTGATGCATTACAAAGAG

TAGCAGGAACTACTCCTGTTACGACTTCAGTACCTACTGTTAGACGGGCTCCGATAAAGG

AACTGAGGAAATATGGTGCCACTGAATTTATGGGTCTAAAAGGAGTTGATCCATCCATAG

CTGAAAATTGGATGGAGTCGACTAAAAGAATTTTGCAGCAATTGGATTGTACCCCCCGAG

AGTGTTTAATCTGTGCCGTATCGTTATTACAAGGGGAGGCTTATCTATGGTGGGAATCAG

TGGTTCGACATTTACCAGAGAGTCAGATAACGTGGGATCTATTTCAGAAGGAGTTTCAAA

AGAAATATATCGGAGAGATGTATATTGAAGACAAGAAACAAGAGTTTTTGTTGCTACAAC

AGGGTGATATGTCAGTAATAGATTATGAGAGGGAATTCTCGAGACTCAGTAGATATGCCT

CCGAGTTTATTCCGACAGAAGCCGATAGTTGTAAAAGATTTTTACGGGGTTTACGAGACG

AGATCAAAGTGCAGCTAGTATCCCATCGGATCACTGAGTTAGTAGATTTGATTGAACGAG

CTAAAATGGTGGAACAAGTTCTGGGCCTCGACAAAAAGACTGAAGTTGTTAGACCAACCG

GGAAGCGTACAGGAACTACCAGTTCGAATCCTCAGCCGAAAAGACCAAAGGAATTCCAAA

GTGGTTGGAGATCCAGTTTCAGGTCAGACAGAGGTGGTAGAAATAGGGGAAAACAGACGA

TGACATCTACTGGCAGTGTGAAAGGTCCTTCCCGAGAAATAGATATTCCAGACTGCCAAC

ACTGCGGAAAGAAACACAGAGGGGAATGTTGGAAATTAACTAGAGGCTGTTTTCGATGTG

GTTCTACAGACCATTTCATCAGAGACTGTCCGAAAGTTGATAGTACTGTACCCGTGACAT

CACAGAGATCGGTATCTACAGCTAGAGGCAGAGGGTTAGGAAGAGGTGGTTCGGTTTCAA

GGGGAGGAAGTATTAGGAGAAGCAATGATATTGCTACTCAGCAGTCTGAGGCTAAAGTAC

CTGCCAGAGCTTATGTGGTCAGAACACAGGAAGAAGGTGACGCCCACGATGTAGTAACAG

GTATATTCTTACTATATTCTGAGCCTGTTTATGCTTTAATTGATCCCGGATCTTCACATT

CTTATATAAATTCAAAATTAGTTGAATTGGGAAAATTTAATTCTGAAATATCTAGAGTGA

CTGTAGAAGTGTCGAGTCCGTTGGGGCAAACAGTATTAGTGAATCAGATCTGTCCGAGAT

GCCCGTTAATTATACAAAATAAAACTTTTCCTATTGACCTGTTGATTATGCCATTTGGAG

ATTTTGATATAATACTGGGGATGGATTGGTTGGCTGAGCACGGAGTGGTATTGGATTGTT

ATAAAAAGAAGTTTAGTATTCAGACAGAAGACGGGGACAGAATTGAAGTAAATGGTATCC

GTACTAATGGGCCGACACGTATTATTTCGGCAATAAAGGCTAATAAATTGCTTCAGCGGG

GTTGTACAGCGTATTTAGCCTATGTTATTAATTCTGATTTGGTTGGTAGTCAGTGCAGTA

AGATTAGAACCGTATGTGAGTTTCCAGATGTATTTCCTGAAGAGCTACCGGGTTTACCAC

CTGACAGAGAGGTTGAATTTGCTATAGAAGTGTATCCGGGTACAGCACCAATCTCTATAC

CACCGTATCGAATGTCACCCACTGAGTTGAAAGAGTTGAAAGTGCAGTTACAGGACTTGT

CAGATCGTGGATTTATTAGACCGAGCATCTCACCTTGGGGAGCTCCAGTATTGTTTGTTA

AAAAGAAAGATGGATCGATGCGGCTTTGTATTGATTACCGGCAGTTAAACAAAGTGACGA

TCAAGAACCGGTATCCGTTACCCCGTATAGATGATTTATTTGATCAACTAAAAGGAGCTT

CAGTATTTTCAAAGATTGACTTAAGATCTGGGTATTATCAGCTGAAGGTAAAAGAAAGTG

ATGTTCCGAAGACTGCATTTCGTACTCGATATGGTCATTATGAATTTTTGGTGATGCCGT

TCGGGTTGACTAATGCTCCAGCTGCTTTTATGGATCTGATGAATCGTATTTTTCAGCCGT

ATTTAGATCAGTTTGTGGTGGTTTTTATTGATGACATCTTGGTTTATTCGAAGTCAGAGT

CAGAGCATGATCAGCATCTCAGAACCGTGCTACAAATTCTGCGAGAAAAACAGTTGTACG

GGAAACTAAGTAAATGTGAATTCTGGTTATCAGAGGTAGTATTCTTGGGACATGTTGTAT

CTGCGGATGGGATTAGAGTTGATCCGAAGAAGATCGAGGCAATTGTTCAATGGAAGGCAC

CAAAGAATGTATCAGAGGTACGCAGTTTTCTTGGTTTGGCTGGGTATTACAGAAGATTTG

TAAATGGGTTTTCGAAGATAGCTTTGCCGATGACCAAATTACTACAGAAGAATGTTCCAT

TTATCTGGGATGATCAGTGTCAGAGGAGCTTTGAAACATTGAAACAGATGTTGACAGAGG

CACCAGTTTTAACTTTACCAGAATCAGGGAAAGATTTCATAGTGTACAGTGATGCTTCTT

TGAATGGTTTGGGTTGTGTATTGATGCAAGAAGGAAAAGTAATAGCTTATGCATCTCGAC

AGTTGAAGTCACATGAACGCAACTACCCGACACACGATTTAGAGTTAGCTGCTGTAATCT

TTGCATTGAAGATTTGGATACATTACTTGTATGGTGAGAAATGTTATATTTACACTGATC

ATAAAAGTCTAAAATATCTTCTGTCACAAAAGGAGTTGAATCTGAGACAGAGACGGTGGA

TTGAACTTCTGAAAGATTATGATTGTGTTATAGATTATCATCCAGGGAAGGCAAATGTGG

TAGCAGATGCATTGAGTAGAAAAGCAGCGATTGAATTACGAGCAATGTTCGCTCGACTTA

GTATTAAGGATGATGGAAGTTTGTTAGCTGAGTTAAGAGTCAAGCCGGTGATGTTTGATC

AAATCAGAGCAGCACAGTTAAAAGATGAAAAGTTGATGAGGAAAAGAGAAATGGTACAGT

ATGGTGCGGTAGAAAATTTTAGTATTGACGAGCATGATTGTTTGAGATTTCGAAATCGAA

TTTGTGTTCCATCTACTTCTGAGATTAAAGAATTGATTCTCCGAGAAGCACATAATAGTA

TTTTTGCTTTGCACCCAGGAGGAACGAAGATGTATCGTGATCTACGAGAACTGTATTGGT

GGCCAGGAATGAAGAAAGATATAGTTGAATATGTCAGTAAATGCTTGACTTGTCAGCGGG

TAAAAGCAGAACATCAGGTACCAACAGGCCTGTTACAGCCTATTACTATTCCCGAGTGGA

AATGGGATCGCATTACCATGGATTTTGTTACGGGGTTGCCATTGTCAGTGAGTAAAAAGA

ATGCTATTTGGGTGATTGTTGATCGACTCACAAAATCAGCTCATTTTATAGCAGTTAGAA

CCGACTGGTCATTACAGAAGCTTGCCGAGGTTTATATTCGAGAAATTGTTAGATTACATG

GTATTCCGGTATCAATAATTTCAGACAGAGATCCTCGATTCACTTCGAGATTTTGGAAGC

AGCTGCATGAATCATTGGGTACTCGACTTAGTTTCAGTACAGCTTTTCATCCTCAAACTG

ATGGACAATCTGAACGAGTAATTCAGATATTAGAAGATATGCTTCGAGCTTGTGTCATTG

ATTTTGAATCAGGTTGGGAACGTTATTTACCATTGGCCGAGTTTGTTTATAATAATAGTT

TCCAATCTAGTATTCAAATGGCTCCATATGAAGCACTTTATGGTCGAAGGTGTCGATCAC

CAATATGTTGGACAAAATTAAGAGAAAGAAAAGTGATTGGGCCGGAATTGATTCAAGAGA

CAGAAGAAACAGTTAAAAAGATTAAAGATAGACTGAAAGCCGCTTTCGACAGACAGAAAT

CTTACGCAGACTTGAAACGACGAGACATTGAATATTCCGTTGGTGATAAGGTATTCCTCA

AAGTATCGCCGTGGAAGAAAATTTTGAGATTTGGTCGGAAGGGAAAATTAAGTCCGCGCT

TTATTGGGCCGTATGAGATAGTGGAAAGAATTGGGCCTGTTGCTTATCGATTATCCTTAC

CTCCAGAGTTACAGAAAATTCATGATGTTTTTCATGTTTCGATGCTTCGGAGATATAGAT

CGGATCCTTCTCATGTTATTCCCACTGAAGACATTGAACTTCGATCTGATTTAACTTATG

AAGAAGAACCAGTTCAAATATTAGCACGAGAAGTGAAAGAATTAAGAAATAAACGGGTTC

CTTTAGTACAAGTTTTATGGAGAAGCCATAGTGTGGAAGAAGCAACTTGGGAACCGGAAG

AGACAATGAGAGCACAATATCCTCATCTCTTCTCAGGTAAATTTCGAGGACGAAATTTAT

TAAGAGGGGGAGAAATGTAATGACCTAAAATTCATGGGCATCGGAAAAGTATAATATTGG

GCCTCCGTCCTAGTAAATTGAGTCCGAAAATAATTATTAGAAATATTTACGAGACTAGTA

GTGTGTTTAATTAGGTTTTAATTAAGTAAATTTAGCTTAATTTAGAGTAATTAGTAAAAA

GGATTAAATTGAATAAGAGTAAAAGTTTAATTATAGATTAAAGGAAAATAATAGGGACCA

AATGGGCAATTAAGCCACATTTGGAAGTTGAGGCGGCATAACATTGTAAAAATCTTAGAT

TTTTATATTATTATTTATATAAATATATAAATTAATTATAAAGTATATTATTAAATTAAT

TATATTATAAATATTATATTATTATATATAAAAGAAACAAAACAGAAAAGAAACAGAATA

GAAAGAACAAAGAAACAGAATAGAAGAGACGAAACAGGGGAGAAGCAGGGGAGAAAGAAG

AAAAAGAAGAAAAAAGGGGAAATAGGGTTTTTGAAGCTTGAAATTTAAATTGGTAAGTCA

AATTAGCCATTTTCTCTTAATTCTAATGTTTTAAAAGCTTTAAAACAAAGTTTTGATGGA

ATTAAGTTGATATTTTGTAAGTTCATAGGTTTTCAAGTATAGTTTATGTTGAACAAAAGA

GATGAATTAGGGATTAACTTGAAGGAATTTTAAGTTAGAATTGAAAAAGGGATTAAATTG

TAAAAGAAACTATAAGTTTTTTTTGTTTTAGGGACTAGATTGAGGAAAATTCGGAATTAA

GAAAATATGTTAAAAATTTAATAGTTAAATTTGAGTTTAAATGAAATTTGAATAGGAATA

AGGTGTGAATTGGTGTTATAAATTTGGTTATTAACATTTTTAATCAAAACAGTTTTGGGA

AGTAGCAATGGTCTGACTTTGAAAATTCACTAAAAATTTTATAAATTGAACTAGAGGATG

AACAAAATATGGAATTAAAGCTTATTGAGTCTAGTTTCTTATAGTAGAAACAATGTAAGC

AATTAATTGATGAATCAAGAGATATTTGAAATTTTGTAATACTGGTTCGGGGTGATTTCG

AGATGCCCTGTTTTAACTTTGGAAAATCATTAAAAATTGTACAAAAATTATTATGGAGTG

TAATTTATATATGTAAACTCCTTAATGAATCTAGTTTCAAAATAAATAAACAAGAACCTT

ATTCGAGTTCTGTACAATGAGATAATTTAGTTTTAGTGGAGAGAGGTCAGAACTGTCAAA

TGAAATAACAGGGGAGTATTTAACGAATAAACTGTATTAAATGGCTAGACCAAAAATTCT

GGAAATTTTATGATTAGAAGATATATGAGTCTAGTTTTAAGGAAAATTTACGGATATTAA

TTTGGAGTTTCGTAGCTCAAGATATAAATAATTTAGTAACAATGACCCAAGTAGACAGCT

TAATGGTGAAATTATATAAATACATTAAAAATGGTTAAATTTGCATGTTTAGGCTCATGA

ATTAAATTGAATCATGTTGTATTGATTATTATAAATTATTATTTTCGTAGCCAACAAAGA

ACCTAAAGCATCAGCATCGAAAGGAAAGGAGAAAGTCATCGAGGAGTAAACTCGAGAAAA

TTACGGTTTGTATTACTATAATTCAAGTTATTTATTATTAAATGTTAAATTTTAATTTAT

GTGTCTAGTAAATGAAATGTGAGGTAAGTATTATTATTATTATTATTATTATTATTATTA

TTATTATTATTATTATTATGAGTGGGAATTAAATTGAATAGTTGATATGAAATAATATTT

GAATTGTTTGTTGATTGAAAGCGGGAAATGAATTTAAATCGAATAGTGACCGATATTAAA

TTGAATGGAAATGTATTGAGTTGTGAAAATATGTTAATTGCGGATTAATTATTGATTGAA

AGGTGGAAAAATGATTGAATTGAAAGTGTGAGAAAGTGTGATTGAATTGGGATTATATGT

GATTTAAATACCCTATTAACTAGTCGGGCTGAGTCGGATATAGTTGGCATGCCATAGGAT

TGGAAGAGTTCAGGGATACTTCGACCTCGAGTCGATGAGACACTGGGTGTCACTATATTT

CTTCGGATAGATTCGATGAGGTACTGGGTACCAACTTTCTTCGGCTTTGCCGATGAGACA

CTGGGTGTCAACTATTGCTTCGAACTATCCGATGAGGCACTGGGTGCCATTCTGGTGTGT

TTGGTTGGATCCGTGTATTCGCCAAAGTCCGAGTTTTGTTAATAGGGTAAATGATGAAAT

GATAAACCGAACGAGTTGGTCAAACGAGCTATTGAAATGATATGAAAAAGTTGAATTGTG

AATTGAAATGTGAAATGAGATTGAGAAATGAACCTAAGGTTCGTGAATTATTCAAACTCA

AATTGTGGATATACGATATTGGTTGATGAATTGCTATTGTTGAAATATTTAATTTAAATT

GTATATACGATTTATGCTTTACATGTACATTATTGTTATAATTTGAATTATGGTAATACC

ACTGAGTATGAATTACTCAGCGTACGGTTGTTTCCGTGCGCAGGTCAATAGAAGTCAAAG

GTCTCGGTTCAGCATCCAGATTAATCCCGGCTTCGGCAAAACTTGGTGATGTATTTTTCC

TTTGGTAAAGGTGGCATGTACATAGATTGTGTATAAAGGTTATTATGTTTTATTATATAA

TGGTTAAAAATGTTAGTATTAAAAGTTTATGGATTTTAATGAAAGAAGTCTATCTATTTT

ATCTAATTAGTACATTGTTAAATTTTAAATTGGTATTAGATTGAGTTTGATTAGAAGTAT

TTAGAATAGAAAATGTGAATGTGAAATGAATTGGTTGAATTGATGATATTTGGGAACTAT

ATGGTTTTAATTTGC

>AR9317-26

AGGTAATGACCCAAAATTCATGGGCATCGGAAAAGTATAATATCGGGCCTCCGTCCTAGT

AAATTGAGTCCGAAAATAATTATTAGAAATATTTACGAGACTAGTAGTGTGTTTAATTAG

GTTTTAATTAAGTAAATTTAGCTTAATTTAGAGTAATTAGTAAAAAGGATTAAATTGAAT

AAGAGTAAAAGTTTAATTATAGATTAAAGGAAAATAATAGGGACCAAATGGGCAATTAAG

CCACATTTGGAAGTTGAGGCGGCATAACATTGTAAAAATCTTAGATTTTTATATTATTAT

TTATATAAATATATAAATTAATTATAAAGTATATTATTAAATTAATTATATTATAAATAT

TATATTATTATATATAAAAGAAACAAAACAGAAAAGAAACAGAATAGAAAGAACAAAGAA

ACAGAATAGAAGAGACGAAACAGGGGAGAAGCAGGGGAGAAAGAAGAAAAAGAAGAAAAA

AGGGGAAATAGGGTTTTTGAAGCTTGAAATTTAAATTGGTAAGTCAAATTAGCCATTTTC

TCTTAATTCTAATGTTTTAAAAGCTTTAAAACAAAGTTTTGATGGAATTAAGTTGATATT

TTGTAAGTTCATAGGTTTTCAAGTATAGTTTATGTTGAACAAAAGAGATGAATTAGGGAT

TAACTTGAAGGAATTTTAAGTTAGAATTGAAAAAGGGATTAAATTGTAAAAGAAACTATA

AGTTTTTTTTGTTTTAGGGACTAGATTGAGGAAAATTCGGAATTAAGAAAATATGTTAAA

AATTTAATAGTTAAATTTGAGTTTAAATGAAATTTGAATAGGAATAAGGTGTGAATTGGT

GTTATAAATTTGGTTATTAACATTTTTAATCAAAACAGTTTTGGGAAGTAGCAATGGTCT

GACTTTGAAAATTCACTAAAAATTTTATAAATTGAACTAGAGGATGAACAAAATATGGAA

TTAAAGCTTATTGAGTCTAGTTTCTTATAGTAGAAACAATGTAAGCAATTAATTGATGAA

TCAAGAGATATTTGAAATTTTGTAATACTGGTTCGGGGTGATTTCGAGATGCCCTGTTTT

AACTTTGGAAAATCATTAAAAATTGTACAAAAATTATTATGGAGTGTAATTTATATATGT

GAACTCCTTAATGAATCTAGTTTCAAAATAAATAAACAAGAACCTTATTCGAGTTCTGTA

CAATGAGATAATTTAGTTTTAGTGGAGAGAGGTCAGAACTGTCAAATGAAATAACAGGGG

AGTATTTAACGAATAAACTGTATTAAATGGCTAGACCAAAAATTCTGGAAATTTTATGAT

TAGAAGATATATGAGTCTAGTTTTAAGGAAAATTTACGGATATTAATTTGGAGTTTCGTA

GCTCAAGATATAAATAATTTAGTAACAATGACCCAAGTAGACAGCTTAATGGTGAAATTA

TATAAATACATTAAAAATGGTTAAATTTGCATGTTTAGGCTCATGAATTAAATTGAATCA

TGTTGTATTGATTATTATAAATTATTATTTTCGTAGCCAACAAAGAACCTAAAGCATCAG

CATCGAAAGGAAAGGAGAAAGTCATCGAGGAGTAAACTCGAGAAAATTACGGTTTGTATT

ACTATAATTCAAGTTATTTATTATTAAATGTTAAATTTTAATTTATGTGTCTAGTAAATG

AAATGTGAGGTAAGTATTATTATTATTATTATTATTATTATTATTATTATTATTATTATT

ATGAGTGGGAATTAAATTGAATAGTTGATATGAAATAATATTTGAATTGTTTGTTGATTG

AAAGCGGGAAATGAATTTAAATCGAATAGTGACCGATATTAAATTGAATGGAAATGTATT

GAGTTGTGAAAATATGTTAATTGCGGATTAATTATTGATTGAAAGGTGGAAAAATGATTG

AATTGAAAGTGTGAGAAAGTGTGATTGAATTGGGATTATATGTGATTTAAATACCCTATT

AACTAGTCGGGCTGAGTCGGATATAGTTGGCATGCCATAGGATTGGAAGAGTTCAGGGAT

ACTTCGACCTCGAGTCGATGAGACACTGGGTGATTTCTTCGGATAGATTGGATGAGGTAC

TGGGTACCAACTTTCTTCGGCTTTGCCGATGAGACACTGGGTGTCAACTATTGCTTCGAA

CTATCCGATGAGGCACTGGGTGCCATTCTGGTGTGTTTGGTTGGATCCGTGTATCCGCCA

AAGTCCGAGTTTTGTTAATAGGGTAAATGATGAAATGATAAACCGAACGAGTTGGTCAAA

CGAGCTATTGAAATGATATGAAAAAGTTGAATTGTGAATTGAAATGTGAAATGAGATTGA

GAAATGAACCTAAGGTTCGTGAATTATTCAAACTCAAATTGTGGATATACGATATTGGTT

GATGAATTGCTATTGTTGAAATATTTAATTTAAATTGTATATACGATTTATGCTTTACAT

GTACATTATTGTTATAATTTGAATTATGGTAATACCACTGAGTATGAATTACTCAGCGTA

CGGTTGTTTCCGTGCGCAGGTCAATAGAAGTCAAAGGTCTCGGTTCAGCATCCAGATTAA

TCCCGGCTTCGGCAAAACTTGGTGATGTATTTTTCCTTTGGTAAAGGTGGCATGTACATA

GATTGTGTATAAAGGTTATTATGTTTTATTATATAATGGTTAAAAATGTTAGTATTAAAA

GTTTATGGATTTTAATGAAAGAAGTCTATCTATTTTATCTAATTAGTACATTGTTAAATT

TTAAATTGGTATTGTGTAGATTGAGTTTGATTAGAAGTATTTAGAATAGAAAATGTGAAT

GTGAAATGAATTGGTTGAATTGATGATATTTGGGAACTATATGGTTTTAATTTGCAGGGG

GTTTTATGTAAAAATAAGCAGAAATGCTGCCGAAATTTTTATAAAAAAAAATGAAGTCAT

TTGGTAAACAAATTAATAAATTTTATGAATTATTTTAATATATTGGTTATTTATTTAAGA

ATTGTTGTAAATCGTTCGATACGTCCGGTAGTGCCTCGTAATTCTGTTCCGGCGACGGTT

CGGGGTTAAGGGGTGTTACATTTTATGGTATCAGAGCTATCAGGTTTAGCCGATTCTCGG

CCTAAATCGAGCTCGGAATTGAGTCTAGATGTACATGCCACTGTCGAGTTAAACTGAGTC

GGGATTTTTGGATGCTGACCTATTTGTTTGTTTTGTTTTATAGATTAAAGATGTCTGAAG

AAAGAATAAATGATACTGATGAAAGAATGTATAGTGAAGATAGAGAATTAGATGAAACAG

AATCTGTTGCACCGAGTGTGAATCCGTTAGGCAACCAACCTTCTAATGTAGAACGAGAAA

ATGTCAGAGATAGAGATGAATCCCAATTACTGAGAATTATAGCTGATGCATTACAAAGAG

TAGCAGGAACTACTCCTGTTACGACTTCAGTACCTACTGTTAGACGGGCTCCGATAAAGG

AACTGAGGAAATATGGTGCCACTGAATTTATGGGTCTAAAAGGAGTTGATCCATCCATAG

CTGAAAATTGGATGGAGTCGACTAAAAGAATTTTGCAGCAATTGGATTGTACCCCCCGAG

AGTGTTTAATCTGTGCCGTATCGTTATTACAAGGGGAGGCTTATCTATGGTGGGAATCAG

TGGTTCGACATTTACCAGAGAGTCAGATAACGTGGGATCTATTTCAGAAGGAGTTTCAAA

AGAAATATATCGGAGAGATGTATATTGAAGACAAGAAACAAGAGTTTTTGTTGCTACAAC

AGGGTGATATGTCAGTAATAGATTATGAGAGGGAATTCTCGAGACTCAGTAGATATGCCT

CCGAGTTTATTCCGACAGAAGCCGATAGTTGTAAAAGATTTTTACGGGGTTTACGAGACG

AGATCAAAGTGCAGCTAGTATCCCATCGGATCACTGAGTTAGTAGATTTGATTGAACGAG

CTAAAATGGTGGAACAAGTTCTGGGCCTCGACAAAAAGACTGAAGTTGTTAGACCAACCG

GGAAGCGTACAGGAACTACCAGTTCGAATCCTCAGCCGAAAAGACCAAAGGAATTCCAAA

GTGGTTGGAGATCCAGTTTCAGGTCAGACAGAGGTGGTAGAAATAGGGGAAAACAGACGA

TGACATCTACTGGCAGTGTGAAAGGTCCTTCCCGAGAAATAGATATTCCAGACTGCCAAC

ACTGCGGAAAGAAACACAGAGGGGAATGTTGGAAATTAACTAGAGGCTGTTTTCGATGTG

GTTCTACAGACCATTTCATCAGAGACTGTCCGAAAGTTGATAGTACTGTACCCGTGACAT

CACAGAGATCGGTATCTACAGCTAGAGGCAGAGGGTTAGGAAGAGGTGGTTCGGTTTCAA

GGGGAGGAAGTATTAGGAGAAGCAATGATATTGCTACTCAGCAGTCTGAGGCTAAAGTAC

CTGCCAGAGCTTATGTGGTCAGAACACGGGAAGAAGGTGACGCCCACGATGTAGTAACAG

GTATATTCTTACTATATTCTGAGCCTGTTTATGCTTTAATTGATCCCGGATCTTCACATT

CTTATATAAATTCAAAATTAGTTGAATTGGGAAAATTTAATTCTGAAATATCTAGAGTGA

CTGTAGAAGTGTCGAGTCCGTTGGGGCAAACAGTATTAGTGAATCAGATCTGTCCGAGAT

GCCCGTTAATTATACAAAATAAAACTTTTCCTATTGACCTGTTGATTATGCCATTTGGAG

ATTTTGATATAATACTGGGGATGGATTGGTTGGCTGAGCACGGAGTGGTATTGGATTGTT

ATAAAAAGAAGTTTAGTATTCAGACAGAAGACGGGGACAGAATTGAAGTAAATGGTATCC

GTACTAATGGGCCGACACGTATTATTTCGGCAATAAAGGCTAATAAATTGCTTCAGCGGG

GTTGTACAGCGTATTTAGCCTATGTTATTAATTCTGATTTGGTTGGTAGTCAGTGCAGTA

AGATTAGAACCGTATGTGAGTTTCCAGATGTATTTCCTGAAGAGCTACCGGGTTTACCAC

CTGACAGAGAGGTTGAATTTGCTATAGAAGTGTATCCGGGTACAGCACCAATCTCTATAC

CACCGTATCGAATGTCACCCACTGAGTTGAAAGAGTTGAAAGTGCAGTTACAGGACTTGT

CAGATCGTGGATTTATTAGACCGAGCATCTCACCTTGGGGAGCTCCAGTATTGTTTGTTA

AAAAGAAAGATGGATCGATGCGGCTTTGTATTGATTACCGGCAGTTAAACAAAGTGACGA

TCAAGAACCGGTATCCGTTACCCCGTATAGATGATTTATTTGATCAACTAAAAGGAGCTT

CAGTATTTTCAAAGATTGACTTAAGATCTGGGTATTATCAGCTGAAGGTAAAAGAAAGTG

ATGTTCCGAAGACTGCATTTCGTACTCGATATGGTCATTATGAATTTTTGGTGATGCCGT

TCGGGTTGACTAATGCTCCAGCTGCTTTTATGGATCTGATGAATCGTATTTTTCAGCCGT

ATTTAGATCAGTTTGTGGTGGTTTTTATTGATGACATCTTGGTTTATTCGAAGTCAGAGT

CAGAGCATGATCAGCATCTCAGAACCGTGCTACAAATTCTGCGAGAAAAACAGTTGTACG

GGAAACTAAGTAAATGTGAATTCTGGTTATCAGAGGTAGTATTCTTGGGACATGTTGTAT

CTGCGGATGGGATTAGAGTTGATCCGAAGAAGATCGAGGCAATTGTTCAATGGAAGGCAC

CAAAGAATGTATCAGAGGTACGCAGTTTTCTTGGTTTGGCTGGGTATTACAGAAGATTTG

TAAATGGGTTTTCGAAGATAGCTTTGCCGATGACCAAATTACTACAGAAGAATGTTCCAT

TTATCTGGGATGATCAGTGTCAGAGGAGCTTTGAAACATTGAAACAGATGTTGACAGAGG

CACCAGTTTTAACTTTACCAGAATCAGGGAAAGATTTCATAGTGTACAGTGATGCTTCTT

TGAATGGTTTGGGTTGTGTATTGATGCAAGAAGGAAAAGTAATAGCTTATGCATCTCGAC

AGTTGAAGTCACATGAACGCAACTACCCGACACACGATTTAGAGTTAGCTGCTGTAATCT

TTGCATTGAAGATTTGGATACATTACTTGTATGGTGAGAAATGTTATATTTACACTGATC

ATAAAAGTCTAAAATATCTTCTGTCACAAAAGGAGTTGAATCTGAGACAGAGACGGTGGA

TTGAACTTCTGAAAGATTATGATTGTGTTATAGATTATCATCCAGGGAAGGCAAATGTGG

TAGCAGATGCATTGAGTAGAAAAGCAGCGATTGAATTACGAGCAATGTTCGCTCGACTTA

GTATTAAGGATGATGGAAGTTTGTTAGCTGAGTTAAGAGTCAAGCCGGTGATGTTTGATC

AAATCAGAGCAGCACAGTTAAAAGATGAAAAGTTGATGAGGAAAAGAGAAATGGTACAGT

ATGGTGCGGTAGAAAATTTTAGTATTGACGAGCATGATTGTTTGAGATTTCGAAATCGAA

TTTGTGTTCCATCTACTTCTGAGATTAAAGAATTGATTCTCCGAGAAGCACATAATAGTA

TTTTTGCTTTGCACCCAGGAGGAACGAAGATGTATCGTGATCTACGAGAACTGTATTGGT

GGCCAGGAATGAAGAAAGATATAGTTGAATATGTCAGTAAATGCTTGACTTGTCAGCGGG

TAAAAGCAGAACATCAGGTACCAACAGGCCTGTTACAGCCTATTACTATTCCCGAGTGGA

AATGGGATCGCATTACCATGGATTTTGTTACGGGGTTGCCATTGTCAGTGAGTAAAAAGA

ATGCTATTTGGGTGATTGTTGATCGACTCACAAAATCAGCTCATTTTATAGCAGTTAGAA

CCGACTGGTCATTACAGAAGCTTGCCGAGGTTTATATTCGAGAAATTGTTAGATTACATG

GTATTCCGGTATCAATAATTTCAGACAGAGATCCTCGATTCACTTCGAGATTTTGGAAGC

AGCTGCATGAATCATTGGGTACTCGACTTAGTTTCAGTACAGCTTTTCATCCTCAAACTG

ATGGACAATCTGAACGAGTAATTCAGATATTAGAAGATATGCTTCGAGCTTGTGTCATTG

ATTTTGAATCAGGTTGGGAACGTTATTTACCATTGGCCGAGTTTGTTTATAATAATAGTT

TCCAATCTAGTATTCAAATGGCTCCATATGAAGCACTTTATGGTCGAAGGTGTCGATCAC

CAATATGTTGGACAAAATTAAGAGAAAGAAAAGTGATTGGGCCGGAATTGATTCAAGAGA

CAGAAGAAACAGTTAAAAAGATTAAAGATAGACTGAAAGCCGCTTTCGACAGACAGAAAT

CTTACGCAGACTTGAAACGACGAGACATTGAATATTCCGTTGGTGATAAGGTATTCCTCA

AAGTATCGCCGTGGAAGAAAATTTTGAGATTTGGTCGGAAGGGAAAATTAAGTCCGCGCT

TTATTGGGCCGTATGAGATAGTGGAAAGAATTGGGCCTGTTGCTTATCGATTATCCTTAC

CTCCAGAGTTACAGAAAATTCATGATGTTTTTCATGTTTCGATGCTTCGGAGATATAGAT

CGGATCCTTCTCATGTTATTCCCACTGAAGACATTGAACTTCGATCTGATTTAACTTATG

AAGAAGAACCAGTTCAAATATTAGCACGAGAAGTGAAAGAATTAAGAAATAAACGGGTTC

CTTTAGTACAAGTTTTATGGAGAAGCCATAGTGTGGAAGAAGCAACTTGGGAACCGGAAG

AGACAATGAGAGCACAATATCCTCATCTCTTCTCAGGTAAATTTCGAGGACGAAATTTAT

TAAGAGGGGGAGAAATGTAATGACCTAAAATTCATGGGCATCGGAAAAGTATAATATTGG

GCCTCCGTCCTAGTAAATTGAGTCCGAAAATAATTATTAGAAATATTTACGAGACTAGTA

GTGTGTTTAATTAGGTTTTAATTAAGTAAATTTAGCTTAATTTAGAGTAATTAGTAAAAA

GGATTAAATTGAATAAGAGTAAAAGTTTAATTATAGATTAAAGGAAAATAATAGGGACCA

AATGGGCAATTAAGCCACATTTGGAAGTTGAGGCGGCATAACATTGTAAAAATCTTAGAT

TTTTATATTATTATTTATATAAATATATAAATTAATTATAAAGTATATTATTAAATTAAT

TATATTATAAATATTATATTATTATATATAAAAGAAACAAAACAGAAAAGAAACAGAATA

GAAAGAACAAAGAAACAGAATAGAAGAGACGAAACAGGGGAGAAGCAGGGGAGAAAGAAG

AAAAAGAAGAAAAAAGGGGAAATAGGGTTTTTGAAGCTTGAAATTTAAATTGGTAAGTCA

AATTAGCCATTTTCTCTTAATTCTAATGTTTTAAAAGCTTTAAAACAAAGTTTTGATGGA

ATTAAGTTGATATTTTGTAAGTTCATAGGTTTTCAAGTATAGTTTATGTTGAACAAAAGA

GATGAATTAGGGATTAACTTGAAGGAATTTTAAGTTAGAATTGAAAAAGGGATTAAATTG

TAAAAGAAACTATAAGTTTTTTTTGTTTTAGGGACTAGATTGAGGAAAATTCGGAATTAA

GAAAATATGTTAAAAATTTAATAGTTAAATTTGAGTTTAAATGAAATTTGAATAGGAATA

AGGTGTGAATTGGTGTTATAAATTTGGTTATTAACATTTTTAATCAAAACAGTTTTGGGA

AGTAGCAATGGTCTGACTTTGAAAATTCACTAAAAATTTTATAAATTGAACTAGAGGATG

AACAAAATATGGAATTAAAGCTTATTGAGTCTAGTTTCTTATAGTAGAAACAATGTAAGC

AATTAATTGATGAATCAAGAGATATTTGAAATTTTGTAATACTGGTTCGGGGTGATTTCG

AGATGCCCTGTTTTAACTTTGGAAAATCATTAAAAATTGTACAAAAATTATTATGGAGTG

TAATTTATATATGTAAACTCCTTAATGAATCTAGTTTCAAAATAAATAAACAAGAACCTT

ATTCGAGTTCTGTACAATGAGATAATTTAGTTTTAGTGGAGAGAGGTCAGAACTGTCAAA

TGAAATAACAGGGGAGTATTTAACGAATAAACTGTATTAAATGGCTAGACCAAAAATTCT

GGAAATTTTATGATTAGAAGATATATGAGTCTAGTTTTAAGGAAAATTTACGGATATTAA

TTTGGAGTTTCGTAGCTCAAGATATAAATAATTTAGTAACAATGACCCAAGTAGACAGCT

TAATGGTGAAATTATATAAATACATTAAAAATGGTTAAATTTGCATGTTTAGGCTCATGA

ATTAAATTGAATCATGTTGTATTGATTATTATAAATTATTATTTTCGTAGCCAACAAAGA

ACCTAAAGCATCAGCATCGAAAGGAAAGGAGAAAGTCATCGAGGAGTAAACTCGAGAAAA

TTACGGTTTGTATTACTATAATTCAAGTTATTTATTATTAAATGTTAAATTTTAATTTAT

GTGTCTAGTAAATGAAATGTGAGGTAAGTATTATTATTATTATTATTATTATTATTATTA

TTATTATTATTATTATTATGAGTGGGAATTAAATTGAATAGTTGATATGAAATAATATTT

GAATTGTTTGTTGATTGAAAGCGGGAAATGAATTTAAATCGAATAGTGACCGATATTAAA

TTGAATGGAAATGTATTGAGTTGTGAAAATATGTTAATTGCGGATTAATTATTGATTGAA

AGGTGGAAAAATGATTGAATTGAAAGTGTGAGAAAGTGTGATTGAATTGGGATTATATGT

GATTTAAATACCCTATTAACTAGTCGGGCTGAGTCGGATATAGTTGGCATGCCATAGGAT

TGGAAGAGTTCAGGGATACTTCGACCTCGAGTCGATGAGACACTGGGTGTCACTATATTT

CTTCGGATAGATTCGATGAGGTACTGGGTACCAACTTTCTTCGGCTTTGCCGATGAGACA

CTGGGTGTCAACTATTGCTTCGAACTATCCGATGAGGCACTGGGTGCCATTCTGGTGTGT

TTGGTTGGATCCGTGTATTCGCCAAAGTCCGAGTTTTGTTAATAGGGTAAATGATGAAAT

GATAAACCGAACGAGTTGGTCAAACGAGCTATTGAAATGATATGAAAAAGTTGAATTGTG

AATTGAAATGTGAAATGAGATTGAGAAATGAACCTAAGGTTCGTGAATTATTCAAACTCA

AATTGTGGATATACGATATTGGTTGATGAATTGCTATTGTTGAAATATTTAATTTAAATT

GTATATACGATTTATGCTTTACATGTACATTATTGTTATAATTTGAATTATGGTAATACC

ACTGAGTATGAATTACTCAGCGTACGGTTGTTTCCGTGCGCAGGTCAATAGAAGTCAAAG

GTCTCGGTTCAGCATCCAGATTAATCCCGGCTTCGGCAAAACTTGGTGATGTATTTTTCC

TTTGGTAAAGGTGGCATGTACATAGATTGTGTATAAAGGTTATTATGTTTTATTATATAA

TGGTTAAAAATGTTAGTATTAAAAGTTTATGGATTTTAATGAAAGAAGTCTATCTATTTT

ATCTAATTAGTACATTGTTAAATTTTAAATTGGTATTAGATTGAGTTTGATTAGAAGTAT

TTAGAATAGAAAATGTGAATGTGAAATGAATTGGTTGAATTGATGATATTTGGGAACTAT

ATGGTTTTAATTTGC

>Atlas

ATGTAATGACCCAAAATTCATGGGCATCGGAAAAGTATAATATCGGGCCTCCGTCCTAGT

AAATTGAGTCCGAAAATAATTATTAGAAATATTTACGAGACTAGTAGTGTGTTTAATTAG

GTTTTAATTAAGTAAATTTAGCTTAATTTAGAGTAATTAGTAAAAAGGATTAAATTGAAT

AAGAGTAAAAGTTTAATTATAGATTAAAGGAAAATAATAGGGACCAAATGGGCAATTAAG

CCACATTTGGAAGTTGAGGCGGCATAACATTGTAAAAATCTTAGATTTTTATATTATTAT

TTATATAAATATATAAATTAATTATAAAGTATATTATTAAATTAATTATATTATAAATAT

TATATTATTATATATAAAAGAAACAAAACAGAAAAGAAACAGAATAGAAAGAACAAAGAA

ACAGAATAGAAGAGACGAAACAGGGGAGAAGCAGGGGAGAAAGAAGAAAAAGAAGAAAAA

AGGGGAAATAGGGTTTTTGAAGCTTGAAATTTAAATTGGTAAGTCAAATTAGCCATTTTC

TCTTAATTCTAATGTTTTAAAAGCTTTAAAACAAAGTTTTGATGGAATTAAGTTGATATT

TTGTAAGTTCATAGGTTTTCAAGTATAGTTTATGTTGAACAAAAGAGATGAATTAGGGAT

TAACTTGAAGGAATTTTAAGTTAGAATTGAAAAAGGGATTAAATTGTAAAAGAAACTATA

AGTTTTTTTTGTTTTAGGGACTAGATTGAGGAAAATTCGGAATTAAGAAAATATGTTAAA

AATTTAATAGTTAAATTTGAGTTTAAATGAAATTTGAATAGGAATAAGGTGTGAATTGGT

GTTATAAATTTGGTTATTAACATTTTTAATCAAAACAGTTTTGGGAAGTAGCAATGGTCT

GACTTTGAAAATTCACTAAAAATTTTATAAATTGAACTAGAGGATGAACAAAATATGGAA

TTAAAGCTTATTGAGTCTAGTTTCTTATAGTAGAAACAATGTAAGCAATTAATTGATGAA

TCAAGAGATATTTGAAATTTTGTAATACTGGTTCGGGGTGATTTCGAGATGCCCTGTTTT

AACTTTGGAAAATCATTAAAAATTGTACAAAAATTATTATGGAGTGTAATTTATATATGT

GAACTCCTTAATGAATCTAGTTTCAAAATAAATAAACAAGAACCTTATTCGAGTTCTGTA

CAATGAGATAATTTAGTTTTAGTGGAGAGAGGTCAGAACTGTCAAATGAAATAACAGGGG

AGTATTTAACGAATAAACTGTATTAAATGGCTAGACCAAAAATTCTGGAAATTTTATGAT

TAGAAGATATATGAGTCTAGTTTTAAGGAAAATTTACGGATATTAATTTGGAGTTTCGTA

GCTCAAGATATAAATAATTTAGTAACAATGACCCAAGTAGACAGCTTAATGGTGAAATTA

TATAAATACATTAAAAATGGTTAAATTTGCATGTTTAGGCTCATGAATTAAATTGAATCA

TGTTGTATTGATTATTATAAATTATTATTTTCGTAGCCAACAAAGAACCTAAAGCATCAG

CATCGAAAGGAAAGGAGAAAGTCATCGAGGAGTAAACTCGAGAAAATTACGGTTTGTATT

ACTATAATTCAAGTTATTTATTATTAAATGTTAAATTTTAATTTATGTGTCTAGTAAATG

AAATGTGAGGTAAGTATTATTATTATTATTATTATTATTATTATTATTATTATTATTATT

ATGAGTGGGAATTAAATTGAATAGTTGATATGAAATAATATTTGAATTGTTTGTTGATTG

AAAGCGGGAAATGAATTTAAATCGAATAGTGACCGATATTAAATTGAATGGAAATGTATT

GAGTTGTGAAAATATGTTAATTGCGGATTAATTATTGATTGAAAGGTGGAAAAATGATTG

AATTGAAAGTGTGAGAAAGTGTGATTGAATTGGGATTATATGTGATTTAAATACCCTATT

AACTAGTCGGGCTGAGTCGGATATAGTTGGCATGCCATAGGATTGGAAGAGTTCAGGGAT

ACTTCGACCTCGAGTCGATGAGACACTGGGTGATTTCTTCGGATAGATTGGATGAGGTAC

TGGGTACCAACTTTCTTCGGCTTTGCCGATGAGACACTGGGTGTCAACTATTGCTTCGAA

CTATCCGATGAGGCACTGGGTGCCATTCTGGTGTGTTTGGTTGGATCCGTGTATCCGCCA

AAGTCCGAGTTTTGTTAATAGGGTAAATGATGAAATGATAAACCGAACGAGTTGGTCAAA

CGAGCTATTGAAATGATATGAAAAAGTTGAATTGTGAATTGAAATGTGAAATGAGATTGA

GAAATGAACCTAAGGTTCGTGAATTATTCAAACTCAAATTGTGGATATACGATATTGGTT

GATGAATTGCTATTGTTGAAATATTTAATTTAAATTGTATATACGATTTATGCTTTACAT

GTACATTATTGTTATAATTTGAATTATGGTAATACCACTGAGTATGAATTACTCAGCGTA

CGGTTGTTTCCGTGCGCAGGTCAATAGAAGTCAAAGGTCTCGGTTCAGCATCCAGATTAA

TCCCGGCTTCGGCAAAACTTGGTGATGTATTTTTCCTTTGGTAAAGGTGGCATGTACATA

GATTGTGTATAAAGGTTATTATGTTTTATTATATAATGGTTAAAAATGTTAGTATTAAAA

GTTTATGGATTTTAATGAAAGAAGTCTATCTATTTTATCTAATTAGTACATTGTTAAATT

TTAAATTGGTATTGTGTAGATTGAGTTTGATTAGAAGTATTTAGAATAGAAAATGTGAAT

GTGAAATGAATTGGTTGAATTGATGATATTTGGGAACTATATGGTTTTAATTTGCAGGGG

GTTTTATGTAAAAATAAGCAGAAATGCTGCCGAAATTTTTATAAAAAAAAATGAAGTCAT

TTGGTAAACAAATTAATAAATTTTATGAATTATTTTAATATATTGGTTATTTATTTAAGA

ATTGTTGTAAATCGTTCGATACGTCCGGTAGTGCCTCGTAATTCTGTTCCGGCGACGGTT

CGGGGTTAAGGGGTGTTACATTTTATGGTATCAGAGCTATCAGGTTTAGCCGATTCTCGG

CCTAAATCGAGCTCGGAATTGAGTCTAGATGTACATGCCACTGTCGAGTTAAACTGAGTC

GGGATTTTTGGATGCTGACCTATTTGTTTGTTTTGTTTTATAGATTAAAGATGTCTGAAG

AAAGAATAAATGATACTGATGAAAGAATGTATAGTGAAGATAGAGAATTAGATGAAACAG

AATCTGTTGCACCGAGTGTGAATCCGTTAGGCAACCAACCTTCTAATGTAGAACGAGAAA

ATGTCAGAGATAGAGATGAATCCCAATTACTGAGAATTATAGCTGATGCATTACAAAGAG

TAGCAGGAACTACTCCTGTTACGACTTCAGTACCTACTGTTAGACGGGCTCCGATAAAGG

AACTGAGGAAATATGGTGCCACTGAATTTATGGGTCTAAAAGGAGTTGATCCATCCATAG

CTGAAAATTGGATGGAGTCGACTAAAAGAATTTTGCAGCAATTGGATTGTACCCCCCGAG

AGTGTTTAATCTGTGCCGTATCGTTATTACAAGGGGAGGCTTATCTATGGTGGGAATCAG

TGGTTCGACATTTACCAGAGAGTCAGATAACGTGGGATCTATTTCAGAAGGAGTTTCAAA

AGAAATATATCGGAGAGATGTATATTGAAGACAAGAAACAAGAGTTTTTGTTGCTACAAC

AGGGTGATATGTCAGTAATAGATTATGAGAGGGAATTCTCGAGACTCAGTAGATATGCCT

CCGAGTTTATTCCGACAGAAGCCGATAGTTGTAAAAGATTTTTACGGGGTTTACGAGACG

AGATCAAAGTGCAGCTAGTATCCCATCGGATCACTGAGTTAGTAGATTTGATTGAACGAG

CTAAAATGGTGGAACAAGTTCTGGGCCTCGACAAAAAGACTGAAGTTGTTAGACCAACCG

GGAAGCGTACAGGAACTACCAGTTCGAATCCTCAGCCGAAAAGACCAAAGGAATTCCAAA

GTGGTTGGAGATCCAGTTTCAGGTCAGACAGAGGTGGTAGAAATAGGGGAAAACAGACGA

TGACATCTACTGGCAGTGTGAAAGGTCCTTCCCGAGAAATAGATATTCCAGACTGCCAAC

ACTGCGGAAAGAAACACAGAGGGGAATGTTGGAAATTAACTAGAGGCTGTTTTCGATGTG

GTTCTACAGACCATTTCATCAGAGACTGTCCGAAAGTTGATAGTACTGTACCCGTGACAT

CACAGAGATCGGTATCTACAGCTAGAGGCAGAGGGTTAGGAAGAGGTGGTTCGGTTTCAA

GGGGAGGAAGTATTAGGAGAAGCAATGATATTGCTACTCAGCAGTCTGAGGCTAAAGTAC

CTGCCAGAGCTTATGTGGTCAGAACACAGGAAGAAGGTGACGCCCACGATGTAGTAACAG

GTATATTCTTACTATATTCTGAGCCTGTTTATGCTTTAATTGATCCCGGATCTTCACATT

CTTATATAAATTCAAAATTAGTTGAATTGGGAAAATTTAATTCTGAAATATCTAGAGTGA

CTGTAGAAGTGTCGAGTCCGTTGGGGCAAACAGTATTAGTGAATCAGATCTGTCCGAGAT

GCCCGTTAATTATACAAAATAAAACTTTTCCTATTGACCTGTTGATTATGCCATTTGGAG

ATTTTGATATAATACTGGGGATGGATTGGTTGGCTGAGCACGGAGTGGTATTGGATTGTT

ATAAAAAGAAGTTTAGTATTCAGACAGAAGACGGGGACAGAATTGAAGTAAATGGTATCC

GTACTAATGGGCCGACACGTATTATTTCGGCAATAAAGGCTAATAAATTGCTTCAGCGGG

GTTGTACAGCGTATTTAGCCTATGTTATTAATTCTGATTTGGTTGGTAGTCAGTGCAGTA

AGATTAGAACCGTATGTGAGTTTCCAGATGTATTTCCTGAAGAGCTACCGGGTTTACCAC

CTGACAGAGAGGTTGAATTTGCTATAGAAGTGTATCCGGGTACAGCACCAATCTCTATAC

CACCGTATCGAATGTCACCCACTGAGTTGAAAGAGTTGAAAGTGCAGTTACAGGACTTGT

CAGATCGTGGATTTATTAGACCGAGCATCTCACCTTGGGGAGCTCCAGTATTGTTTGTTA

AAAAGAAAGATGGATCGATGCGGCTTTGTATTGATTACCGGCAGTTAAACAAAGTGACGA

TCAAGAACCGGTATCCGTTACCCCGTATAGATGATTTATTTGATCAACTAAAAGGAGCTT

CAGTATTTTCAAAGATTGACTTAAGATCTGGGTATTATCAGCTGAAGGTAAAAGAAAGTG

ATGTTCCGAAGACTGCATTTCGTACTCGATATGGTCATTATGAATTTTTGGTGATGCCGT

TCGGGTTGACTAATGCTCCAGCTGCTTTTATGGATCTGATGAATCGTATTTTTCAGCCGT

ATTTAGATCAGTTTGTGGTGGTTTTTATTGATGACATCTTGGTTTATTCGAAGTCAGAGT

CAGAGCATGATCAGCATCTCAGAACCGTGCTACAAATTCTGCGAGAAAAACAGTTGTACG

GGAAACTAAGTAAATGTGAATTCTGGTTATCAGAGGTAGTATTCTTGGGACATGTTGTAT

CTGCGGATGGGATTAGAGTTGATCCGAAGAAGATCGAGGCAATTGTTCAATGGAAGGCAC

CAAAGAATGTATCAGAGGTACGCAGTTTTCTTGGTTTGGCTGGGTATTACAGAAGATTTG

TAAATGGGTTTTCGAAGATAGCTTTGCCGATGACCAAATTACTACAGAAGAATGTTCCAT

TTATCTGGGATGATCAGTGTCAGAGGAGCTTTGAAACATTGAAACAGATGTTGACAGAGG

CACCAGTTTTAACTTTACCAGAATCAGGGAAAGATTTCATAGTGTACAGTGATGCTTCTT

TGAATGGTTTGGGTTGTGTATTGATGCAAGAAGGAAAAGTAATAGCTTATGCATCTCGAC

AGTTGAAGTCACATGAACGCAACTACCCGACACACGATTTAGAGTTAGCTGCTGTAATCT

TTGCATTGAAGATTTGGATACATTACTTGTATGGTGAGAAATGTTATATTTACACTGATC

ATAAAAGTCTAAAATATCTTCTGTCACAAAAGGAGTTGAATCTGAGACAGAGACGGTGGA

TTGAACTTCTGAAAGATTATGATTGTGTTATAGATTATCATCCAGGGAAGGCAAATGTGG

TAGCAGATGCATTGAGTAGAAAAGCAGCGATTGAATTACGAGCAATGTTCGCTCGACTTA

GTATTAAGGATGATGGAAGTTTGTTAGCTGAGTTAAGAGTCAAGCCGGTGATGTTTGATC

AAATCAGAGCAGCACAGTTAAAAGATGAAAAGTTGATGAGGAAAAGAGAAATGGTACAGT

ATGGTGCGGTAGAAAATTTTAGTATTGACGAGCATGATTGTTTGAGATTTCGAAATCGAA

TTTGTGTTCCATCTACTTCTGAGATTAAAGAATTGATTCTCCGAGAAGCACATAATAGTA

TTTTTGCTTTGCACCCAGGAGGAACGAAGATGTATCGTGATCTACGAGAACTGTATTGGT

GGCCAGGAATGAAGAAAGATATAGTTGAATATGTCAGTAAATGCTTGACTTGTCAGCGGG

TAAAAGCAGAACATCAGGTACCAACAGGCCTGTTACAGCCTATTACTATTCCCGAGTGGA

AATGGGATCGCATTACCATGGATTTTGTTACGGGGTTGCCATTGTCAGTGAGTAAAAAGA

ATGCTATTTGGGTGATTGTTGATCGACTCACAAAATCAGCTCATTTTATAGCAGTTAGAA

CCGACTGGTCATTACAGAAGCTTGCCGAGGTTTATATTCGAGAAATTGTTAGATTACATG

GTATTCCGGTATCAATAATTTCAGACAGAGATCCTCGATTCACTTCGAGATTTTGGAAGC

AGCTGCATGAATCATTGGGTACTCGACTTAGTTTCAGTACAGCTTTTCATCCTCAAACTG

ATGGACAATCTGAACGAGTAATTCAGATATTAGAAGATATGCTTCGAGCTTGTGTCATTG

ATTTTGAATCAGGTTGGGAACGTTATTTACCATTGGCCGAGTTTGTTTATAATAATAGTT

TCCAATCTAGTATTCAAATGGCTCCATATGAAGCACTTTATGGTCGAAGGTGTCGATCAC

CAATATGTTGGACAAAATTAAGAGAAAGAAAAGTGATTGGGCCGGAATTGATTCAAGAGA

CAGAAGAAACAGTTAAAAAGATTAAAGATAGACTGAAAGCCGCTTTCGACAGACAGAAAT

CTTACGCAGACTTGAAACGACGAGACATTGAATATTCCGTTGGTGATAAGGTATTCCTCA

AAGTATCGCCGTGGAAGAAAATTTTGAGATTTGGTCGGAAGGGAAAATTAAGTCCGCGCT

TTATTGGGCCGTATGAGATAGTGGAAAGAATTGGGCCTGTTGCTTATCGATTATCCTTAC

CTCCAGAGTTACAGAAAATTCATGATGTTTTTCATGTTTCGATGCTTCGGAGATATAGAT

CGGATCCTTCTCATGTTATTCCCACTGAAGACATTGAACTTCGATCTGATTTAACTTATG

AAGAAGAACCAGTTCAAATATTAGCACGAGAAGTGAAAGAATTAAGAAATAAACGGGTTC

CTTTAGTACAAGTTTTATGGAGAAGCCATAGTGTGGAAGAAGCAACTTGGGAACCGGAAG

AGACAATGAGAGCACAATATCCTCATCTCTTCTCAGGTAAATTTCGAGGACGAAATTTAT

TAAGAGGGGGAGAAATGTAATGACCTAAAATTCATGGGCATCGGAAAAGTATAATATTGG

GCCTCCGTCCTAGTAAATTGAGTCCGAAAATAATTATTAGAAATATTTACGAGACTAGTA

GTGTGTTTAATTAGGTTTTAATTAAGTAAATTTAGCTTAATTTAGAGTAATTAGTAAAAA

GGATTAAATTGAATAAGAGTAAAAGTTTAATTATAGATTAAAGGAAAATAATAGGGACCA

AATGGGCAATTAAGCCACATTTGGAAGTTGAGGCGGCATAACATTGTAAAAATCTTAGAT

TTTTATATTATTATTTATATAAATATATAAATTAATTATAAAGTATATTATTAAATTAAT

TATATTATAAATATTATATTATTATATATAAAAGAAACAAAACAGAAAAGAAACAGAATA

GAAAGAACAAAGAAACAGAATAGAAGAGACGAAACAGGGGAGAAGCAGGGGAGAAAGAAG

AAAAAGAAGAAAAAAGGGGAAATAGGGTTTTTGAAGCTTGAAATTTAAATTGGTAAGTCA

AATTAGCCATTTTCTCTTAATTCTAATGTTTTAAAAGCTTTAAAACAAAGTTTTGATGGA

ATTAAGTTGATATTTTGTAAGTTCATAGGTTTTCAAGTATAGTTTATGTTGAACAAAAGA

GATGAATTAGGGATTAACTTGAAGGAATTTTAAGTTAGAATTGAAAAAGGGATTAAATTG

TAAAAGAAACTATAAGTTTTTTTTGTTTTAGGGACTAGATTGAGGAAAATTCGGAATTAA

GAAAATATGTTAAAAATTTAATAGTTAAATTTGAGTTTAAATGAAATTTGAATAGGAATA

AGGTGTGAATTGGTGTTATAAATTTGGTTATTAACATTTTTAATCAAAACAGTTTTGGGA

AGTAGCAATGGTCTGACTTTGAAAATTCACTAAAAATTTTATAAATTGAACTAGAGGATG

AACAAAATATGGAATTAAAGCTTATTGAGTCTAGTTTCTTATAGTAGAAACAATGTAAGC

AATTAATTGATGAATCAAGAGATATTTGAAATTTTGTAATACTGGTTCGGGGTGATTTCG

AGATGCCCTGTTTTAACTTTGGAAAATCATTAAAAATTGTACAAAAATTATTATGGAGTG

TAATTTATATATGTAAACTCCTTAATGAATCTAGTTTCAAAATAAATAAACAAGAACCTT

ATTCGAGTTCTGTACAATGAGATAATTTAGTTTTAGTGGAGAGAGGTCAGAACTGTCAAA

TGAAATAACAGGGGAGTATTTAACGAATAAACTGTATTAAATGGCTAGACCAAAAATTCT

GGAAATTTTATGATTAGAAGATATATGAGTCTAGTTTTAAGGAAAATTTACGGATATTAA

TTTGGAGTTTCGTAGCTCAAGATATAAATAATTTAGTAACAATGACCCAAGTAGACAGCT

TAATGGTGAAATTATATAAATACATTAAAAATGGTTAAATTTGCATGTTTAGGCTCATGA

ATTAAATTGAATCATGTTGTATTGATTATTATAAATTATTATTTTCGTAGCCAACAAAGA

ACCTAAAGCATCAGCATCGAAAGGAAAGGAGAAAGTCATCGAGGAGTAAACTCGAGAAAA

TTACGGTTTGTATTACTATAATTCAAGTTATTTATTATTAAATGTTAAATTTTAATTTAT

GTGTCTAGTAAATGAAATGTGAGGTAAGTATTATTATTATTATTATTATTATTATTATTA

TTATTATTATTATTATTATGAGTGGGAATTAAATTGAATAGTTGATATGAAATAATATTT

GAATTGTTTGTTGATTGAAAGCGGGAAATGAATTTAAATCGAATAGTGACCGATATTAAA

TTGAATGGAAATGTATTGAGTTGTGAAAATATGTTAATTGCGGATTAATTATTGATTGAA

AGGTGGAAAAATGATTGAATTGAAAGTGTGAGAAAGTGTGATTGAATTGGGATTATATGT

GATTTAAATACCCTATTAACTAGTCGGGCTGAGTCGGATATAGTTGGCATGCCATAGGAT

TGGAAGAGTTCAGGGATACTTCGACCTCGAGTCGATGAGACACTGGGTGTCACTATATTT

CTTCGGATAGATTCGATGAGGTACTGGGTACCAACTTTCTTCGGCTTTGCCGATGAGACA

CTGGGTGTCAACTATTGCTTCGAACTATCCGATGAGGCACTGGGTGCCATTCTGGTGTGT

TTGGTTGGATCCGTGTATTCGCCAAAGTCCGAGTTTTGTTAATAGGGTAAATGATGAAAT

GATAAACCGAACGAGTTGGTCAAACGAGCTATTGAAATGATATGAAAAAGTTGAATTGTG

AATTGAAATGTGAAATGAGATTGAGAAATGAACCTAAGGTTCGTGAATTATTCAAACTCA

AATTGTGGATATACGATATTGGTTGATGAATTGCTATTGTTGAAATATTTAATTTAAATT

GTATATACGATTTATGCTTTACATGTACATTATTGTTATAATTTGAATTATGGTAATACC

ACTGAGTATGAATTACTCAGCGTACGGTTGTTTCCGTGCGCAGGTCAATAGAAGTCAAAG

GTCTCGGTTCAGCATCCAGATTAATCCCGGCTTCGGCAAAACTTGGTGATGTATTTTTCC

TTTGGTAAAGGTGGCATGTACATAGATTGTGTATAAAGGTTATTATGTTTTATTATATAA

TGGTTAAAAATGTTAGTATTAAAAGTTTATGGATTTTAATGAAAGAAGTCTATCTATTTT

ATCTAATTAGTACATTGTTAAATTTTAAATTGGTATTAGATTGAGTTTGATTAGAAGTAT

TTAGAATAGAAAATGTGAATGTGAAATGAATTGGTTGAATTGATGATATTTGGGAACTAT

ATGGTTTTAATTTGC

>Auburn56

ATGTAATGACCCAAAATTCATGGGCATCGGAAAAGTATAATATCGGGCCTCCGTCCTAGT

AAATTGAGTCCGAAAATAATTATTAGAAATATTTACGAGACTAGTAGTGTGTTTAATTAG

GTTTTAATTAAGTAAATTTAGCTTAATTTAGAGTAATTAGTAAAAAGGATTAAATTGAAT

AAGAGTAAAAGTTTAATTATAGATTAAAGGAAAATAATAGGGACCAAATGGGCAATTAAG

CCACATTTGGAAGTTGAGGCGGCATAACATTGTAAAAATCTTAGATTTTTATATTATTAT

TTATATAAATATATAAATTAATTATAAAGTATATTATTAAATTAATTATATTATAAATAT

TATATTATTATATATAAAAGAAACAAAACAGAAAAGAAACAGAATAGAAAGAACAAAGAA

ACAGAATAGAAGAGACGAAACAGGGGAGAAGCAGGGGAGAAAGAAGAAAAAGAAGAAAAA

AGGGGAAATAGGGTTTTTGAAGCTTGAAATTTAAATTGGTAAGTCAAATTAGCCATTTTC

TCTTAATTCTAATGTTTTAAAAGCTTTAAAACAAAGTTTTGATGGAATTAAGTTGATATT

TTGTAAGTTCATAGGTTTTCAAGTATAGTTTATGTTGAACAAAAGAGATGAATTAGGGAT

TAACTTGAAGGAATTTTAAGTTAGAATTGAAAAAGGGATTAAATTGTAAAAGAAACTATA

AGTTTTTTTTGTTTTAGGGACTAGATTGAGGAAAATTCGGAATTAAGAAAATATGTTAAA

AATTTAATAGTTAAATTTGAGTTTAAATGAAATTTGAATAGGAATAAGGTGTGAATTGGT

GTTATAAATTTGGTTATTAACATTTTTAATCAAAACAGTTTTGGGAAGTAGCAATGGTCT

GACTTTGAAAATTCACTAAAAATTTTATAAATTGAACTAGAGGATGAACAAAATATGGAA

TTAAAGCTTATTGAGTCTAGTTTCTTATAGTAGAAACAATGTAAGCAATTAATTGATGAA

TCAAGAGATATTTGAAATTTTGTAATACTGGTTCGGGGTGATTTCGAGATGCCCTGTTTT

AACTTTGGAAAATCATTAAAAATTGTACAAAAATTATTATGGAGTGTAATTTATATATGT

GAACTCCTTAATGAATCTAGTTTCAAAATAAATAAACAAGAACCTTATTCGAGTTCTGTA

CAATGAGATAATTTAGTTTTAGTGGAGAGAGGTCAGAACTGTCAAATGAAATAACAGGGG

AGTATTTAACGAATAAACTGTATTAAATGGCTAGACCAAAAATTCTGGAAATTTTATGAT

TAGAAGATATATGAGTCTAGTTTTAAGGAAAATTTACGGATATTAATTTGGAGTTTCGTA

GCTCAAGATATAAATAATTTAGTAACAATGACCCAAGTAGACAGCTTAATGGTGAAATTA

TATAAATACATTAAAAATGGTTAAATTTGCATGTTTAGGCTCATGAATTAAATTGAATCA

TGTTGTATTGATTATTATAAATTATTATTTTCGTAGCCAACAAAGAACCTAAAGCATCAG

CATCGAAAGGAAAGGAGAAAGTCATCGAGGAGTAAACTCGAGAAAATTACGGTTTGTATT

ACTATAATTCAAGTTATTTATTATTAAATGTTAAATTTTAATTTATGTGTCTAGTAAATG

AAATGTGAGGTAAGTATTATTATTATTATTATTATTATTATTATTATTATTATTATTATT

ATGAGTGGGAATTAAATTGAATAGTTGATATGAAATAATATTTGAATTGTTTGTTGATTG

AAAGCGGGAAATGAATTTAAATCGAATAGTGACCGATATTAAATTGAATGGAAATGTATT

GAGTTGTGAAAATATGTTAATTGCGGATTAATTATTGATTGAAAGGTGGAAAAATGATTG

AATTGAAAGTGTGAGAAAGTGTGATTGAATTGGGATTATATGTGATTTAAATACCCTATT

AACTAGTCGGGCTGAGTCGGATATAGTTGGCATGCCATAGGATTGGAAGAGTTCAGGGAT

ACTTCGACCTCGAGTCGATGAGACACTGGGTGATTTCTTCGGATAGATTGGATGAGGTAC

TGGGTACCAACTTTCTTCGGCTTTGCCGATGAGACACTGGGTGTCAACTATTGCTTCGAA

CTATCCGATGAGGCACTGGGTGCCATTCTGGTGTGTTTGGTTGGATCCGTGTATCCGCCA

AAGTCCGAGTTTTGTTAATAGGGTAAATGATGAAATGATAAACCGAACGAGTTGGTCAAA

CGAGCTATTGAAATGATATGAAAAAGTTGAATTGTGAATTGAAATGTGAAATGAGATTGA

GAAATGAACCTAAGGTTCGTGAATTATTCAAACTCAAATTGTGGATATACGATATTGGTT

GATGAATTGCTATTGTTGAAATATTTAATTTAAATTGTATATACGATTTATGCTTTACAT

GTACATTATTGTTATAATTTGAATTATGGTAATACCACTGAGTATGAATTACTCAGCGTA

CGGTTGTTTCCGTGCGCAGGTCAATAGAAGTCAAAGGTCTCGGTTCAGCATCCAGATTAA

TCCCGGCTTCGGCAAAACTTGGTGATGTATTTTTCCTTTGGTAAAGGTGGCATGTACATA

GATTGTGTATAAAGGTTATTATGTTTTATTATATAATGGTTAAAAATGTTAGTATTAAAA

GTTTATGGATTTTAATGAAAGAAGTCTATCTATTTTATCTAATTAGTACATTGTTAAATT

TTAAATTGGTATTGTGTAGATTGAGTTTGATTAGAAGTATTTAGAATAGAAAATGTGAAT

GTGAAATGAATTGGTTGAATTGATGATATTTGGGAACTATATGGTTTTAATTTGCAGGGG

GTTTTATGTAAAAATAAGCAGAAATGCTGCCGAAATTTTTATAAAAAAAAATGAAGTCAT

TTGGTAAACAAATTAATAAATTTTATGAATTATTTTAATATATTGGTTATTTATTTAAGA

ATTGTTGTAAATCGTTCGATACGTCCGGTAGTGCCTCGTAATTCTGTTCCGGCGACGGTT

CGGGGTTAAGGGGTGTTACATTTTATGGTATCAGAGCTATCAGGTTTAGCCGATTCTCGG

CCTAAATCGAGCTCGGAATTGAGTCTAGATGTACATGCCACTGTCGAGTTAAACTGAGTC

GGGATTTTTGGATGCTGACCTATTTGTTTGTTTTGTTTTATAGATTAAAGATGTCTGAAG

AAAGAATAAATGATACTGATGAAAGAATGTATAGTGAAGATAGAGAATTAGATGAAACAG

AATCTGTTGCACCGAGTGTGAATCCGTTAGGCAACCAACCTTCTAATGTAGAACGAGAAA

ATGTCAGAGATAGAGATGAATCCCAATTACTGAGAATTATAGCTGATGCATTACAAAGAG

TAGCAGGAACTACTCCTGTTACGACTTCAGTACCTACTGTTAGACGGGCTCCGATAAAGG

AACTGAGGAAATATGGTGCCACTGAATTTATGGGTCTAAAAGGAGTTGATCCATCCATAG

CTGAAAATTGGATGGAGTCGACTAAAAGAATTTTGCAGCAATTGGATTGTACCCCCCGAG

AGTGTTTAATCTGTGCCGTATCGTTATTACAAGGGGAGGCTTATCTATGGTGGGAATCAG

TGGTTCGACATTTACCAGAGAGTCAGATAACGTGGGATCTATTTCAGAAGGAGTTTCAAA

AGAAATATATCGGAGAGATGTATATTGAAGACAAGAAACAAGAGTTTTTGTTGCTACAAC

AGGGTGATATGTCAGTAATAGATTATGAGAGGGAATTCTCGAGACTCAGTAGATATGCCT

CCGAGTTTATTCCGACAGAAGCCGATAGTTGTAAAAGATTTTTACGGGGTTTACGAGACG

AGATCAAAGTGCAGCTAGTATCCCATCGGATCACTGAGTTAGTAGATTTGATTGAACGAG

CTAAAATGGTGGAACAAGTTCTGGGCCTCGACAAAAAGACTGAAGTTGTTAGACCAACCG

GGAAGCGTACAGGAACTACCAGTTCGAATCCTCAGCCGAAAAGACCAAAGGAATTCCAAA

GTGGTTGGAGATCCAGTTTCAGGTCAGACAGAGGTGGTAGAAATAGGGGAAAACAGACGA

TGACATCTACTGGCAGTGTGAAAGGTCCTTCCCGAGAAATAGATATTCCAGACTGCCAAC

ACTGCGGAAAGAAACACAGAGGGGAATGTTGGAAATTAACTAGAGGCTGTTTTCGATGTG

GTTCTACAGACCATTTCATCAGAGACTGTCCGAAAGTTGATAGTACTGTACCCGTGACAT

CACAGAGATCGGTATCTACAGCTAGAGGCAGAGGGTTAGGAAGAGGTGGTTCGGTTTCAA

GGGGAGGAAGTATTAGGAGAAGCAATGATATTGCTACTCAGCAGTCTGAGGCTAAAGTAC

CTGCCAGAGCTTATGTGGTCAGAACACAGGAAGAAGGTGACGCCCACGATGTAGTAACAG

GTATATTCTTACTATATTCTGAGCCTGTTTATGCTTTAATTGATCCCGGATCTTCACATT

CTTATATAAATTCAAAATTAGTTGAATTGGGAAAATTTAATTCTGAAATATCTAGAGTGA

CTGTAGAAGTGTCGAGTCCGTTGGGGCAAACAGTATTAGTGAATCAGATCTGTCCGAGAT

GCCCGTTAATTATACAAAATAAAACTTTTCCTATTGACCTGTTGATTATGCCATTTGGAG

ATTTTGATATAATACTGGGGATGGATTGGTTGGCTGAGCACGGAGTGGTATTGGATTGTT

ATAAAAAGAAGTTTAGTATTCAGACAGAAGACGGGGACAGAATTGAAGTAAATGGTATCC

GTACTAATGGGCCGACACGTATTATTTCGGCAATAAAGGCTAATAAATTGCTTCAGCGGG

GTTGTACAGCGTATTTAGCCTATGTTATTAATTCTGATTTGGTTGGTAGTCAGTGCAGTA

AGATTAGAACCGTATGTGAGTTTCCAGATGTATTTCCTGAAGAGCTACCGGGTTTACCAC

CTGACAGAGAGGTTGAATTTGCTATAGAAGTGTATCCGGGTACAGCACCAATCTCTATAC

CACCGTATCGAATGTCACCCACTGAGTTGAAAGAGTTGAAAGTGCAGTTACAGGACTTGT

CAGATCGTGGATTTATTAGACCGAGCATCTCACCTTGGGGAGCTCCAGTATTGTTTGTTA

AAAAGAAAGATGGATCGATGCGGCTTTGTATTGATTACCGGCAGTTAAACAAAGTGACGA

TCAAGAACCGGTATCCGTTACCCCGTATAGATGATTTATTTGATCAACTAAAAGGAGCTT

CAGTATTTTCAAAGATTGACTTAAGATCTGGGTATTATCAGCTGAAGGTAAAAGAAAGTG

ATGTTCCGAAGACTGCATTTCGTACTCGATATGGTCATTATGAATTTTTGGTGATGCCGT

TCGGGTTGACTAATGCTCCAGCTGCTTTTATGGATCTGATGAATCGTATTTTTCAGCCGT

ATTTAGATCAGTTTGTGGTGGTTTTTATTGATGACATCTTGGTTTATTCGAAGTCAGAGT

CAGAGCATGATCAGCATCTCAGAACCGTGCTACAAATTCTGCGAGAAAAACAGTTGTACG

GGAAACTAAGTAAATGTGAATTCTGGTTATCAGAGGTAGTATTCTTGGGACATGTTGTAT

CTGCGGATGGGATTAGAGTTGATCCGAAGAAGATCGAGGCAATTGTTCAATGGAAGGCAC

CAAAGAATGTATCAGAGGTACGCAGTTTTCTTGGTTTGGCTGGGTATTACAGAAGATTTG

TAAATGGGTTTTCGAAGATAGCTTTGCCGATGACCAAATTACTACAGAAGAATGTTCCAT

TTATCTGGGATGATCAGTGTCAGAGGAGCTTTGAAACATTGAAACAGATGTTGACAGAGG

CACCAGTTTTAACTTTACCAGAATCAGGGAAAGATTTCATAGTGTACAGTGATGCTTCTT

TGAATGGTTTGGGTTGTGTATTGATGCAAGAAGGAAAAGTAATAGCTTATGCATCTCGAC

AGTTGAAGTCACATGAACGCAACTACCCGACACACGATTTAGAGTTAGCTGCTGTAATCT

TTGCATTGAAGATTTGGATACATTACTTGTATGGTGAGAAATGTTATATTTACACTGATC

ATAAAAGTCTAAAATATCTTCTGTCACAAAAGGAGTTGAATCTGAGACAGAGACGGTGGA

TTGAACTTCTGAAAGATTATGATTGTGTTATAGATTATCATCCAGGGAAGGCAAATGTGG

TAGCAGATGCATTGAGTAGAAAAGCAGCGATTGAATTACGAGCAATGTTCGCTCGACTTA

GTATTAAGGATGATGGAAGTTTGTTAGCTGAGTTAAGAGTCAAGCCGGTGATGTTTGATC

AAATCAGAGCAGCACAGTTAAAAGATGAAAAGTTGATGAGGAAAAGAGAAATGGTACAGT

ATGGTGCGGTAGAAAATTTTAGTATTGACGAGCATGATTGTTTGAGATTTCGAAATCGAA

TTTGTGTTCCATCTACTTCTGAGATTAAAGAATTGATTCTCCGAGAAGCACATAATAGTA

TTTTTGCTTTGCACCCAGGAGGAACGAAGATGTATCGTGATCTACGAGAACTGTATTGGT

GGCCAGGAATGAAGAAAGATATAGTTGAATATGTCAGTAAATGCTTGACTTGTCAGCGGG

TAAAAGCAGAACATCAGGTACCAACAGGCCTGTTACAGCCTATTACTATTCCCGAGTGGA

AATGGGATCGCATTACCATGGATTTTGTTACGGGGTTGCCATTGTCAGTGAGTAAAAAGA

ATGCTATTTGGGTGATTGTTGATCGACTCACAAAATCAGCTCATTTTATAGCAGTTAGAA

CCGACTGGTCATTACAGAAGCTTGCCGAGGTTTATATTCGAGAAATTGTTAGATTACATG

GTATTCCGGTATCAATAATTTCAGACAGAGATCCTCGATTCACTTCGAGATTTTGGAAGC

AGCTGCATGAATCATTGGGTACTCGACTTAGTTTCAGTACAGCTTTTCATCCTCAAACTG

ATGGACAATCTGAACGAGTAATTCAGATATTAGAAGATATGCTTCGAGCTTGTGTCATTG

ATTTTGAATCAGGTTGGGAACGTTATTTACCATTGGCCGAGTTTGTTTATAATAATAGTT

TCCAATCTAGTATTCAAATGGCTCCATATGAAGCACTTTATGGTCGAAGGTGTCGATCAC

CAATATGTTGGACAAAATTAAGAGAAAGAAAAGTGATTGGGCCGGAATTGATTCAAGAGA

CAGAAGAAACAGTTAAAAAGATTAAAGATAGACTGAAAGCCGCTTTCGACAGACAGAAAT

CTTACGCAGACTTGAAACGACGAGACATTGAATATTCCGTTGGTGATAAGGTATTCCTCA

AAGTATCGCCGTGGAAGAAAATTTTGAGATTTGGTCGGAAGGGAAAATTAAGTCCGCGCT

TTATTGGGCCGTATGAGATAGTGGAAAGAATTGGGCCTGTTGCTTATCGATTATCCTTAC

CTCCAGAGTTACAGAAAATTCATGATGTTTTTCATGTTTCGATGCTTCGGAGATATAGAT

CGGATCCTTCTCATGTTATTCCCACTGAAGACATTGAACTTCGATCTGATTTAACTTATG

AAGAAGAACCAGTTCAAATATTAGCACGAGAAGTGAAAGAATTAAGAAATAAACGGGTTC

CTTTAGTACAAGTTTTATGGAGAAGCCATAGTGTGGAAGAAGCAACTTGGGAACCGGAAG

AGACAATGAGAGCACAATATCCTCATCTCTTCTCAGGTAAATTTCGAGGACGAAATTTAT

TAAGAGGGGGAGAAATGTAATGACCTAAAATTCATGGGCATCGGAAAAGTATAATATTGG

GCCTCCGTCCTAGTAAATTGAGTCCGAAAATAATTATTAGAAATATTTACGAGACTAGTA

GTGTGTTTAATTAGGTTTTAATTAAGTAAATTTAGCTTAATTTAGAGTAATTAGTAAAAA

GGATTAAATTGAATAAGAGTAAAAGTTTAATTATAGATTAAAGGAAAATAATAGGGACCA

AATGGGCAATTAAGCCACATTTGGAAGTTGAGGCGGCATAACATTGTAAAAATCTTAGAT

TTTTATATTATTATTTATATAAATATATAAATTAATTATAAAGTATATTATTAAATTAAT

TATATTATAAATATTATATTATTATATATAAAAGAAACAAAACAGAAAAGAAACAGAATA

GAAAGAACAAAGAAACAGAATAGAAGAGACGAAACAGGGGAGAAGCAGGGGAGAAAGAAG

AAAAAGAAGAAAAAAGGGGAAATAGGGTTTTTGAAGCTTGAAATTTAAATTGGTAAGTCA

AATTAGCCATTTTCTCTTAATTCTAATGTTTTAAAAGCTTTAAAACAAAGTTTTGATGGA

ATTAAGTTGATATTTTGTAAGTTCATAGGTTTTCAAGTATAGTTTATGTTGAACAAAAGA

GATGAATTAGGGATTAACTTGAAGGAATTTTAAGTTAGAATTGAAAAAGGGATTAAATTG

TAAAAGAAACTATAAGTTTTTTTTGTTTTAGGGACTAGATTGAGGAAAATTCGGAATTAA

GAAAATATGTTAAAAATTTAATAGTTAAATTTGAGTTTAAATGAAATTTGAATAGGAATA

AGGTGTGAATTGGTGTTATAAATTTGGTTATTAACATTTTTAATCAAAACAGTTTTGGGA

AGTAGCAATGGTCTGACTTTGAAAATTCACTAAAAATTTTATAAATTGAACTAGAGGATG

AACAAAATATGGAATTAAAGCTTATTGAGTCTAGTTTCTTATAGTAGAAACAATGTAAGC

AATTAATTGATGAATCAAGAGATATTTGAAATTTTGTAATACTGGTTCGGGGTGATTTCG

AGATGCCCTGTTTTAACTTTGGAAAATCATTAAAAATTGTACAAAAATTATTATGGAGTG

TAATTTATATATGTAAACTCCTTAATGAATCTAGTTTCAAAATAAATAAACAAGAACCTT

ATTCGAGTTCTGTACAATGAGATAATTTAGTTTTAGTGGAGAGAGGTCAGAACTGTCAAA

TGAAATAACAGGGGAGTATTTAACGAATAAACTGTATTAAATGGCTAGACCAAAAATTCT

GGAAATTTTATGATTAGAAGATATATGAGTCTAGTTTTAAGGAAAATTTACGGATATTAA

TTTGGAGTTTCGTAGCTCAAGATATAAATAATTTAGTAACAATGACCCAAGTAGACAGCT

TAATGGTGAAATTATATAAATACATTAAAAATGGTTAAATTTGCATGTTTAGGCTCATGA

ATTAAATTGAATCATGTTGTATTGATTATTATAAATTATTATTTTCGTAGCCAACAAAGA

ACCTAAAGCATCAGCATCGAAAGGAAAGGAGAAAGTCATCGAGGAGTAAACTCGAGAAAA

TTACGGTTTGTATTACTATAATTCAAGTTATTTATTATTAAATGTTAAATTTTAATTTAT

GTGTCTAGTAAATGAAATGTGAGGTAAGTATTATTATTATTATTATTATTATTATTATTA

TTATTATTATTATTATTATGAGTGGGAATTAAATTGAATAGTTGATATGAAATAATATTT

GAATTGTTTGTTGATTGAAAGCGGGAAATGAATTTAAATCGAATAGTGACCGATATTAAA

TTGAATGGAAATGTATTGAGTTGTGAAAATATGTTAATTGCGGATTAATTATTGATTGAA

AGGTGGAAAAATGATTGAATTGAAAGTGTGAGAAAGTGTGATTGAATTGGGATTATATGT

GATTTAAATACCCTATTAACTAGTCGGGCTGAGTCGGATATAGTTGGCATGCCATAGGAT

TGGAAGAGTTCAGGGATACTTCGACCTCGAGTCGATGAGACACTGGGTGTCACTATATTT

CTTCGGATAGATTCGATGAGGTACTGGGTACCAACTTTCTTCGGCTTTGCCGATGAGACA

CTGGGTGTCAACTATTGCTTCGAACTATCCGATGAGGCACTGGGTGCCATTCTGGTGTGT

TTGGTTGGATCCGTGTATTCGCCAAAGTCCGAGTTTTGTTAATAGGGTAAATGATGAAAT

GATAAACCGAACGAGTTGGTCAAACGAGCTATTGAAATGATATGAAAAAGTTGAATTGTG

AATTGAAATGTGAAATGAGATTGAGAAATGAACCTAAGGTTCGTGAATTATTCAAACTCA

AATTGTGGATATACGATATTGGTTGATGAATTGCTATTGTTGAAATATTTAATTTAAATT

GTATATACGATTTATGCTTTACATGTACATTATTGTTATAATTTGAATTATGGTAATACC

ACTGAGTATGAATTACTCAGCGTACGGTTGTTTCCGTGCGCAGGTCAATAGAAGTCAAAG

GTCTCGGTTCAGCATCCAGATTAATCCCGGCTTCGGCAAAACTTGGTGATGTATTTTTCC

TTTGGTAAAGGTGGCATGTACATAGATTGTGTATAAAGGTTATTATGTTTTATTATATAA

TGGTTAAAAATGTTAGTATTAAAAGTTTATGGATTTTAATGAAAGAAGTCTATCTATTTT

ATCTAATTAGTACATTGTTAAATTTTAAATTGGTATTAGATTGAGTTTGATTAGAAGTAT

TTAGAATAGAAAATGTGAATGTGAAATGAATTGGTTGAATTGATGATATTTGGGAACTAT

ATGGTTTTAATTTGC

>Austin

ATGTAATGACCCAAAATTCATGGGCATCGGAAAAGTATAATATCGGGCCTCCGTCCTAGT

AAATTGAGTCCGAAAATAATTATTAGAAATATTTACGAGACTAGTAGTGTGTTTAATTAG

GTTTTAATTAAGTAAATTTAGCTTAATTTAGAGTAATTAGTAAAAAGGATTAAATTGAAT

AAGAGTAAAAGTTTAATTATAGATTAAAGGAAAATAATAGGGACCAAATGGGCAATTAAG

CCACATTTGGAAGTTGAGGCGGCATAACATTGTAAAAATCTTAGATTTTTATATTATTAT

TTATATAAATATATAAATTAATTATAAAGTATATTATTAAATTAATTATATTATAAATAT

TATATTATTATATATAAAAGAAACAAAACAGAAAAGAAACAGAATAGAAAGAACAAAGAA

ACAGAATAGAAGAGACGAAACAGGGGAGAAGCAGGGGAGAAAGAAGAAAAAGAAGAAAAA

AGGGGAAATAGGGTTTTTGAAGCTTGAAATTTAAATTGGTAAGTCAAATTAGCCATTTTC

TCTTAATTCTAATGTTTTAAAAGCTTTAAAACAAAGTTTTGATGGAATTAAGTTGATATT

TTGTAAGTTCATAGGTTTTCAAGTATAGTTTATGTTGAACAAAAGAGATGAATTAGGGAT

TAACTTGAAGGAATTTTAAGTTAGAATTGAAAAAGGGATTAAATTGTAAAAGAAACTATA

AGTTTTTTTTGTTTTAGGGACTAGATTGAGGAAAATTCGGAATTAAGAAAATATGTTAAA

AATTTAATAGTTAAATTTGAGTTTAAATGAAATTTGAATAGGAATAAGGTGTGAATTGGT

GTTATAAATTTGGTTATTAACATTTTTAATCAAAACAGTTTTGGGAAGTAGCAATGGTCT

GACTTTGAAAATTCACTAAAAATTTTATAAATTGAACTAGAGGATGAACAAAATATGGAA

TTAAAGCTTATTGAGTCTAGTTTCTTATAGTAGAAACAATGTAAGCAATTAATTGATGAA

TCAAGAGATATTTGAAATTTTGTAATACTGGTTCGGGGTGATTTCGAGATGCCCTGTTTT

AACTTTGGAAAATCATTAAAAATTGTACAAAAATTATTATGGAGTGTAATTTATATATGT

GAACTCCTTAATGAATCTAGTTTCAAAATAAATAAACAAGAACCTTATTCGAGTTCTGTA

CAATGAGATAATTTAGTTTTAGTGGAGAGAGGTCAGAACTGTCAAATGAAATAACAGGGG

AGTATTTAACGAATAAACTGTATTAAATGGCTAGACCAAAAATTCTGGAAATTTTATGAT

TAGAAGATATATGAGTCTAGTTTTAAGGAAAATTTACGGATATTAATTTGGAGTTTCGTA

GCTCAAGATATAAATAATTTAGTAACAATGACCCAAGTAGACAGCTTAATGGTGAAATTA

TATAAATACATTAAAAATGGTTAAATTTGCATGTTTAGGCTCATGAATTAAATTGAATCA

TGTTGTATTGATTATTATAAATTATTATTTTCGTAGCCAACAAAGAACCTAAAGCATCAG

CATCGAAAGGAAAGGAGAAAGTCATCGAGGAGTAAACTCGAGAAAATTACGGTTTGTATT

ACTATAATTCAAGTTATTTATTATTAAATGTTAAATTTTAATTTATGTGTCTAGTAAATG

AAATGTGAGGTAAGTATTATTATTATTATTATTATTATTATTATTATTATTATTATTATT

ATGAGTGGGAATTAAATTGAATAGTTGATATGAAATAATATTTGAATTGTTTGTTGATTG

AAAGCGGGAAATGAATTTAAATCGAATAGTGACCGATATTAAATTGAATGGAAATGTATT

GAGTTGTGAAAATATGTTAATTGCGGATTAATTATTGATTGAAAGGTGGAAAAATGATTG

AATTGAAAGTGTGAGAAAGTGTGATTGAATTGGGATTATATGTGATTTAAATACCCTATT

AACTAGTCGGGCTGAGTCGGATATAGTTGGCATGCCATAGGATTGGAAGAGTTCAGGGAT

ACTTCGACCTCGAGTCGATGAGACACTGGGTGATTTCTTCGGATAGATTGGATGAGGTAC

TGGGTACCAACTTTCTTCGGCTTTGCCGATGAGACACTGGGTGTCAACTATTGCTTCGAA

CTATCCGATGAGGCACTGGGTGCCATTCTGGTGTGTTTGGTTGGATCCGTGTATCCGCCA

AAGTCCGAGTTTTGTTAATAGGGTAAATGATGAAATGATAAACCGAACGAGTTGGTCAAA

CGAGCTATTGAAATGATATGAAAAAGTTGAATTGTGAATTGAAATGTGAAATGAGATTGA

GAAATGAACCTAAGGTTCGTGAATTATTCAAACTCAAATTGTGGATATACGATATTGGTT

GATGAATTGCTATTGTTGAAATATTTAATTTAAATTGTATATACGATTTATGCTTTACAT

GTACATTATTGTTATAATTTGAATTATGGTAATACCACTGAGTATGAATTACTCAGCGTA

CGGTTGTTTCCGTGCGCAGGTCAATAGAAGTCAAAGGTCTCGGTTCAGCATCCAGATTAA

TCCCGGCTTCGGCAAAACTTGGTGATGTATTTTTCCTTTGGTAAAGGTGGCATGTACATA

GATTGTGTATAAAGGTTATTATGTTTTATTATATAATGGTTAAAAATGTTAGTATTAAAA

GTTTATGGATTTTAATGAAAGAAGTCTATCTATTTTATCTAATTAGTACATTGTTAAATT

TTAAATTGGTATTGTGTAGATTGAGTTTGATTAGAAGTATTTAGAATAGAAAATGTGAAT

GTGAAATGAATTGGTTGAATTGATGATATTTGGGAACTATATGGTTTTAATTTGCAGGGG

GTTTTATGTAAAAATAAGCAGAAATGCTGCCGAAATTTTTATAAAAAAAAATGAAGTCAT

TTGGTAAACAAATTAATAAATTTTATGAATTATTTTAATATATTGGTTATTTATTTAAGA

ATTGTTGTAAATCGTTCGATACGTCCGGTAGTGCCTCGTAATTCTGTTCCGGCGACGGTT

CGGGGTTAAGGGGTGTTACATTTTATGGTATCAGAGCTATCAGGTTTAGCCGATTCTCGG

CCTAAATCGAGCTCGGAATTGAGTCTAGATGTACATGCCACTGTCGAGTTAAACTGAGTC

GGGATTTTTGGATGCTGACCTATTTGTTTGTTTTGTTTTATAGATTAAAGATGTCTGAAG

AAAGAATAAATGATACTGATGAAAGAATGTATAGTGAAGATAGAGAATTAGATGAAACAG

AATCTGTTGCACCGAGTGTGAATCCGTTAGGCAACCAACCTTCTAATGTAGAACGAGAAA

ATGTCAGAGATAGAGATGAATCCCAATTACTGAGAATTATAGCTGATGCATTACAAAGAG

TAGCAGGAACTACTCCTGTTACGACTTCAGTACCTACTGTTAGACGGGCTCCGATAAAGG

AACTGAGGAAATATGGTGCCACTGAATTTATGGGTCTAAAAGGAGTTGATCCATCCATAG

CTGAAAATTGGATGGAGTCGACTAAAAGAATTTTGCAGCAATTGGATTGTACCCCCCGAG

AGTGTTTAATCTGTGCCGTATCGTTATTACAAGGGGAGGCTTATCTATGGTGGGAATCAG

TGGTTCGACATTTACCAGAGAGTCAGATAACGTGGGATCTATTTCAGAAGGAGTTTCAAA

AGAAATATATCGGAGAGATGTATATTGAAGACAAGAAACAAGAGTTTTTGTTGCTACAAC

AGGGTGATATGTCAGTAATAGATTATGAGAGGGAATTCTCGAGACTCAGTAGATATGCCT

CCGAGTTTATTCCGACAGAAGCCGATAGTTGTAAAAGATTTTTACGGGGTTTACGAGACG

AGATCAAAGTGCAGCTAGTATCCCATCGGATCACTGAGTTAGTAGATTTGATTGAACGAG

CTAAAATGGTGGAACAAGTTCTGGGCCTCGACAAAAAGACTGAAGTTGTTAGACCAACCG

GGAAGCGTACAGGAACTACCAGTTCGAATCCTCAGCCGAAAAGACCAAAGGAATTCCAAA

GTGGTTGGAGATCCAGTTTCAGGTCAGACAGAGGTGGTAGAAATAGGGGAAAACAGACGA

TGACATCTACTGGCAGTGTGAAAGGTCCTTCCCGAGAAATAGATATTCCAGACTGCCAAC

ACTGCGGAAAGAAACACAGAGGGGAATGTTGGAAATTAACTAGAGGCTGTTTTCGATGTG

GTTCTACAGACCATTTCATCAGAGACTGTCCGAAAGTTGATAGTACTGTACCCGTGACAT

CACAGAGATCGGTATCTACAGCTAGAGGCAGAGGGTTAGGAAGAGGTGGTTCGGTTTCAA

GGGGAGGAAGTATTAGGAGAAGCAATGATATTGCTACTCAGCAGTCTGAGGCTAAAGTAC

CTGCCAGAGCTTATGTGGTCAGAACACAGGAAGAAGGTGACGCCCACGATGTAGTAACAG

GTATATTCTTACTATATTCTGAGCCTGTTTATGCTTTAATTGATCCCGGATCTTCACATT

CTTATATAAATTCAAAATTAGTTGAATTGGGAAAATTTAATTCTGAAATATCTAGAGTGA

CTGTAGAAGTGTCGAGTCCGTTGGGGCAAACAGTATTAGTGAATCAGATCTGTCCGAGAT

GCCCGTTAATTATACAAAATAAAACTTTTCCTATTGACCTGTTGATTATGCCATTTGGAG

ATTTTGATATAATACTGGGGATGGATTGGTTGGCTGAGCACGGAGTGGTATTGGATTGTT

ATAAAAAGAAGTTTAGTATTCAGACAGAAGACGGGGACAGAATTGAAGTAAATGGTATCC

GTACTAATGGGCCGACACGTATTATTTCGGCAATAAAGGCTAATAAATTGCTTCAGCGGG

GTTGTACAGCGTATTTAGCCTATGTTATTAATTCTGATTTGGTTGGTAGTCAGTGCAGTA

AGATTAGAACCGTATGTGAGTTTCCAGATGTATTTCCTGAAGAGCTACCGGGTTTACCAC

CTGACAGAGAGGTTGAATTTGCTATAGAAGTGTATCCGGGTACAGCACCAATCTCTATAC

CACCGTATCGAATGTCACCCACTGAGTTGAAAGAGTTGAAAGTGCAGTTACAGGACTTGT

CAGATCGTGGATTTATTAGACCGAGCATCTCACCTTGGGGAGCTCCAGTATTGTTTGTTA

AAAAGAAAGATGGATCGATGCGGCTTTGTATTGATTACCGGCAGTTAAACAAAGTGACGA

TCAAGAACCGGTATCCGTTACCCCGTATAGATGATTTATTTGATCAACTAAAAGGAGCTT

CAGTATTTTCAAAGATTGACTTAAGATCTGGGTATTATCAGCTGAAGGTAAAAGAAAGTG

ATGTTCCGAAGACTGCATTTCGTACTCGATATGGTCATTATGAATTTTTGGTGATGCCGT

TCGGGTTGACTAATGCTCCAGCTGCTTTTATGGATCTGATGAATCGTATTTTTCAGCCGT

ATTTAGATCAGTTTGTGGTGGTTTTTATTGATGACATCTTGGTTTATTCGAAGTCAGAGT

CAGAGCATGATCAGCATCTCAGAACCGTGCTACAAATTCTGCGAGAAAAACAGTTGTACG

GGAAACTAAGTAAATGTGAATTCTGGTTATCAGAGGTAGTATTCTTGGGACATGTTGTAT

CTGCGGATGGGATTAGAGTTGATCCGAAGAAGATCGAGGCAATTGTTCAATGGAAGGCAC

CAAAGAATGTATCAGAGGTACGCAGTTTTCTTGGTTTGGCTGGGTATTACAGAAGATTTG

TAAATGGGTTTTCGAAGATAGCTTTGCCGATGACCAAATTACTACAGAAGAATGTTCCAT

TTATCTGGGATGATCAGTGTCAGAGGAGCTTTGAAACATTGAAACAGATGTTGACAGAGG

CACCAGTTTTAACTTTACCAGAATCAGGGAAAGATTTCATAGTGTACAGTGATGCTTCTT

TGAATGGTTTGGGTTGTGTATTGATGCAAGAAGGAAAAGTAATAGCTTATGCATCTCGAC

AGTTGAAGTCACATGAACGCAACTACCCGACACACGATTTAGAGTTAGCTGCTGTAATCT

TTGCATTGAAGATTTGGATACATTACTTGTATGGTGAGAAATGTTATATTTACACTGATC

ATAAAAGTCTAAAATATCTTCTGTCACAAAAGGAGTTGAATCTGAGACAGAGACGGTGGA

TTGAACTTCTGAAAGATTATGATTGTGTTATAGATTATCATCCAGGGAAGGCAAATGTGG

TAGCAGATGCATTGAGTAGAAAAGCAGCGATTGAATTACGAGCAATGTTCGCTCGACTTA

GTATTAAGGATGATGGAAGTTTGTTAGCTGAGTTAAGAGTCAAGCCGGTGATGTTTGATC

AAATCAGAGCAGCACAGTTAAAAGATGAAAAGTTGATGAGGAAAAGAGAAATGGTACAGT

ATGGTGCGGTAGAAAATTTTAGTATTGACGAGCATGATTGTTTGAGATTTCGAAATCGAA

TTTGTGTTCCATCTACTTCTGAGATTAAAGAATTGATTCTCCGAGAAGCACATAATAGTA

TTTTTGCTTTGCACCCAGGAGGAACGAAGATGTATCGTGATCTACGAGAACTGTATTGGT

GGCCAGGAATGAAGAAAGATATAGTTGAATATGTCAGTAAATGCTTGACTTGTCAGCGGG

TAAAAGCAGAACATCAGGTACCAACAGGCCTGTTACAGCCTATTACTATTCCCGAGTGGA

AATGGGATCGCATTACCATGGATTTTGTTACGGGGTTGCCATTGTCAGTGAGTAAAAAGA

ATGCTATTTGGGTGATTGTTGATCGACTCACAAAATCAGCTCATTTTATAGCAGTTAGAA

CCGACTGGTCATTACAGAAGCTTGCCGAGGTTTATATTCGAGAAATTGTTAGATTACATG

GTATTCCGGTATCAATAATTTCAGACAGAGATCCTCGATTCACTTCGAGATTTTGGAAGC

AGCTGCATGAATCATTGGGTACTCGACTTAGTTTCAGTACAGCTTTTCATCCTCAAACTG

ATGGACAATCTGAACGAGTAATTCAGATATTAGAAGATATGCTTCGAGCTTGTGTCATTG

ATTTTGAATCAGGTTGGGAACGTTATTTACCATTGGCCGAGTTTGTTTATAATAATAGTT

TCCAATCTAGTATTCAAATGGCTCCATATGAAGCACTTTATGGTCGAAGGTGTCGATCAC

CAATATGTTGGACAAAATTAAGAGAAAGAAAAGTGATTGGGCCGGAATTGATTCAAGAGA

CAGAAGAAACAGTTAAAAAGATTAAAGATAGACTGAAAGCCGCTTTCGACAGACAGAAAT

CTTACGCAGACTTGAAACGACGAGACATTGAATATTCCGTTGGTGATAAGGTATTCCTCA

AAGTATCGCCGTGGAAGAAAATTTTGAGATTTGGTCGGAAGGGAAAATTAAGTCCGCGCT

TTATTGGGCCGTATGAGATAGTGGAAAGAATTGGGCCTGTTGCTTATCGATTATCCTTAC

CTCCAGAGTTACAGAAAATTCATGATGTTTTTCATGTTTCGATGCTTCGGAGATATAGAT

CGGATCCTTCTCATGTTATTCCCACTGAAGACATTGAACTTCGATCTGATTTAACTTATG

AAGAAGAACCAGTTCAAATATTAGCACGAGAAGTGAAAGAATTAAGAAATAAACGGGTTC

CTTTAGTACAAGTTTTATGGAGAAGCCATAGTGTGGAAGAAGCAACTTGGGAACCGGAAG

AGACAATGAGAGCACAATATCCTCATCTCTTCTCAGGTAAATTTCGAGGACGAAATTTAT

TAAGAGGGGGAGAAATGTAATGACCTAAAATTCATGGGCATCGGAAAAGTATAATATTGG

GCCTCCGTCCTAGTAAATTGAGTCCGAAAATAATTATTAGAAATATTTACGAGACTAGTA

GTGTGTTTAATTAGGTTTTAATTAAGTAAATTTAGCTTAATTTAGAGTAATTAGTAAAAA

GGATTAAATTGAATAAGAGTAAAAGTTTAATTATAGATTAAAGGAAAATAATAGGGACCA

AATGGGCAATTAAGCCACATTTGGAAGTTGAGGCGGCATAACATTGTAAAAATCTTAGAT

TTTTATATTATTATTTATATAAATATATAAATTAATTATAAAGTATATTATTAAATTAAT

TATATTATAAATATTATATTATTATATATAAAAGAAACAAAACAGAAAAGAAACAGAATA

GAAAGAACAAAGAAACAGAATAGAAGAGACGAAACAGGGGAGAAGCAGGGGAGAAAGAAG

AAAAAGAAGAAAAAAGGGGAAATAGGGTTTTTGAAGCTTGAAATTTAAATTGGTAAGTCA

AATTAGCCATTTTCTCTTAATTCTAATGTTTTAAAAGCTTTAAAACAAAGTTTTGATGGA

ATTAAGTTGATATTTTGTAAGTTCATAGGTTTTCAAGTATAGTTTATGTTGAACAAAAGA

GATGAATTAGGGATTAACTTGAAGGAATTTTAAGTTAGAATTGAAAAAGGGATTAAATTG

TAAAAGAAACTATAAGTTTTTTTTGTTTTAGGGACTAGATTGAGGAAAATTCGGAATTAA

GAAAATATGTTAAAAATTTAATAGTTAAATTTGAGTTTAAATGAAATTTGAATAGGAATA

AGGTGTGAATTGGTGTTATAAATTTGGTTATTAACATTTTTAATCAAAACAGTTTTGGGA

AGTAGCAATGGTCTGACTTTGAAAATTCACTAAAAATTTTATAAATTGAACTAGAGGATG

AACAAAATATGGAATTAAAGCTTATTGAGTCTAGTTTCTTATAGTAGAAACAATGTAAGC

AATTAATTGATGAATCAAGAGATATTTGAAATTTTGTAATACTGGTTCGGGGTGATTTCG

AGATGCCCTGTTTTAACTTTGGAAAATCATTAAAAATTGTACAAAAATTATTATGGAGTG

TAATTTATATATGTAAACTCCTTAATGAATCTAGTTTCAAAATAAATAAACAAGAACCTT

ATTCGAGTTCTGTACAATGAGATAATTTAGTTTTAGTGGAGAGAGGTCAGAACTGTCAAA

TGAAATAACAGGGGAGTATTTAACGAATAAACTGTATTAAATGGCTAGACCAAAAATTCT

GGAAATTTTATGATTAGAAGATATATGAGTCTAGTTTTAAGGAAAATTTACGGATATTAA

TTTGGAGTTTCGTAGCTCAAGATATAAATAATTTAGTAACAATGACCCAAGTAGACAGCT

TAATGGTGAAATTATATAAATACATTAAAAATGGTTAAATTTGCATGTTTAGGCTCATGA

ATTAAATTGAATCATGTTGTATTGATTATTATAAATTATTATTTTCGTAGCCAACAAAGA

ACCTAAAGCATCAGCATCGAAAGGAAAGGAGAAAGTCATCGAGGAGTAAACTCGAGAAAA

TTACGGTTTGTATTACTATAATTCAAGTTATTTATTATTAAATGTTAAATTTTAATTTAT

GTGTCTAGTAAATGAAATGTGAGGTAAGTATTATTATTATTATTATTATTATTATTATTA

TTATTATTATTATTATTATGAGTGGGAATTAAATTGAATAGTTGATATGAAATAATATTT

GAATTGTTTGTTGATTGAAAGCGGGAAATGAATTTAAATCGAATAGTGACCGATATTAAA

TTGAATGGAAATGTATTGAGTTGTGAAAATATGTTAATTGCGGATTAATTATTGATTGAA

AGGTGGAAAAATGATTGAATTGAAAGTGTGAGAAAGTGTGATTGAATTGGGATTATATGT

GATTTAAATACCCTATTAACTAGTCGGGCTGAGTCGGATATAGTTGGCATGCCATAGGAT

TGGAAGAGTTCAGGGATACTTCGACCTCGAGTCGATGAGACACTGGGTGTCACTATATTT

CTTCGGATAGATTCGATGAGGTACTGGGTACCAACTTTCTTCGGCTTTGCCGATGAGACA

CTGGGTGTCAACTATTGCTTCGAACTATCCGATGAGGCACTGGGTGCCATTCTGGTGTGT

TTGGTTGGATCCGTGTATTCGCCAAAGTCCGAGTTTTGTTAATAGGGTAAATGATGAAAT

GATAAACCGAACGAGTTGGTCAAACGAGCTATTGAAATGATATGAAAAAGTTGAATTGTG

AATTGAAATGTGAAATGAGATTGAGAAATGAACCTAAGGTTCGTGAATTATTCAAACTCA

AATTGTGGATATACGATATTGGTTGATGAATTGCTATTGTTGAAATATTTAATTTAAATT

GTATATACGATTTATGCTTTACATGTACATTATTGTTATAATTTGAATTATGGTAATACC

ACTGAGTATGAATTACTCAGCGTACGGTTGTTTCCGTGCGCAGGTCAATAGAAGTCAAAG

GTCTCGGTTCAGCATCCAGATTAATCCCGGCTTCGGCAAAACTTGGTGATGTATTTTTCC

TTTGGTAAAGGTGGCATGTACATAGATTGTGTATAAAGGTTATTATGTTTTATTATATAA

TGGTTAAAAATGTTAGTATTAAAAGTTTATGGATTTTAATGAAAGAAGTCTATCTATTTT

ATCTAATTAGTACATTGTTAAATTTTAAATTGGTATTAGATTGAGTTTGATTAGAAGTAT

TTAGAATAGAAAATGTGAATGTGAAATGAATTGGTTGAATTGATGATATTTGGGAACTAT

ATGGTTTTAATTTGC

>Bobshaw1

AGGTAATGACCCAAAATTCATGGGCATCGGAAAAGTATAATATCGGGCCTCCGTCCTAGT

AAATTGAGTCCGAAAATAATTATTAGAAATATTTACGAGACTAGTAGTGTGTTTAATTAG

GTTTTAATTAAGTAAATTTAGCTTAATTTAGAGTAATTAGTAAAAAGGATTAAATTGAAT

AAGAGTAAAAGTTTAATTATAGATTAAAGGAAAATAATAGGGACCAAATGGGCAATTAAG

CCACATTTGGAAGTTGAGGCGGCATAACATTGTAAAAATCTTAGATTTTTATATTATTAT

TTATATAAATATATAAATTAATTATAAAGTATATTATTAAATTAATTATATTATAAATAT

TATATTATTATATATAAAAGAAACAAAACAGAAAAGAAACAGAATAGAAAGAACAAAGAA

ACAGAATAGAAGAGACGAAACAGGGGAGAAGCAGGGGAGAAAGAAGAAAAAGAAGAAAAA

AGGGGAAATAGGGTTTTTGAAGCTTGAAATTTAAATTGGTAAGTCAAATTAGCCATTTTC

TCTTAATTCTAATGTTTTAAAAGCTTTAAAACAAAGTTTTGATGGAATTAAGTTGATATT

TTGTAAGTTCATAGGTTTTCAAGTATAGTTTATGTTGAACAAAAGAGATGAATTAGGGAT

TAACTTGAAGGAATTTTAAGTTAGAATTGAAAAAGGGATTAAATTGTAAAAGAAACTATA

AGTTTTTTTTGTTTTAGGGACTAGATTGAGGAAAATTCGGAATTAAGAAAATATGTTAAA

AATTTAATAGTTAAATTTGAGTTTAAATGAAATTTGAATAGGAATAAGGTGTGAATTGGT

GTTATAAATTTGGTTATTAACATTTTTAATCAAAACAGTTTTGGGAAGTAGCAATGGTCT

GACTTTGAAAATTCACTAAAAATTTTATAAATTGAACTAGAGGATGAACAAAATATGGAA

TTAAAGCTTATTGAGTCTAGTTTCTTATAGTAGAAACAATGTAAGCAATTAATTGATGAA

TCAAGAGATATTTGAAATTTTGTAATACTGGTTCGGGGTGATTTCGAGATGCCCTGTTTT

AACTTTGGAAAATCATTAAAAATTGTACAAAAATTATTATGGAGTGTAATTTATATATGT

GAACTCCTTAATGAATCTAGTTTCAAAATAAATAAACAAGAACCTTATTCGAGTTCTGTA

CAATGAGATAATTTAGTTTTAGTGGAGAGAGGTCAGAACTGTCAAATGAAATAACAGGGG

AGTATTTAACGAATAAACTGTATTAAATGGCTAGACCAAAAATTCTGGAAATTTTATGAT

TAGAAGATATATGAGTCTAGTTTTAAGGAAAATTTACGGATATTAATTTGGAGTTTCGTA

GCTCAAGATATAAATAATTTAGTAACAATGACCCAAGTAGACAGCTTAATGGTGAAATTA

TATAAATACATTAAAAATGGTTAAATTTGCATGTTTAGGCTCATGAATTAAATTGAATCA

TGTTGTATTGATTATTATAAATTATTATTTTCGTAGCCAACAAAGAACCTAAAGCATCAG

CATCGAAAGGAAAGGAGAAAGTCATCGAGGAGTAAACTCGAGAAAATTACGGTTTGTATT

ACTATAATTCAAGTTATTTATTATTAAATGTTAAATTTTAATTTATGTGTCTAGTAAATG

AAATGTGAGGTAAGTATTATTATTATTATTATTATTATTATTATTATTATTATTATTATT

ATGAGTGGGAATTAAATTGAATAGTTGATATGAAATAATATTTGAATTGTTTGTTGATTG

AAAGCGGGAAATGAATTTAAATCGAATAGTGACCGATATTAAATTGAATGGAAATGTATT

GAGTTGTGAAAATATGTTAATTGCGGATTAATTATTGATTGAAAGGTGGAAAAATGATTG

AATTGAAAGTGTGAGAAAGTGTGATTGAATTGGGATTATATGTGATTTAAATACCCTATT

AACTAGTCGGGCTGAGTCGGATATAGTTGGCATGCCATAGGATTGGAAGAGTTCAGGGAT

ACTTCGACCTCGAGTCGATGAGACACTGGGTGATTTCTTCGGATAGATTGGATGAGGTAC

TGGGTACCAACTTTCTTCGGCTTTGCCGATGAGACACTGGGTGTCAACTATTGCTTCGAA

CTATCCGATGAGGCACTGGGTGCCATTCTGGTGTGTTTGGTTGGATCCGTGTATCCGCCA

AAGTCCGAGTTTTGTTAATAGGGTAAATGATGAAATGATAAACCGAACGAGTTGGTCAAA

CGAGCTATTGAAATGATATGAAAAAGTTGAATTGTGAATTGAAATGTGAAATGAGATTGA

GAAATGAACCTAAGGTTCGTGAATTATTCAAACTCAAATTGTGGATATACGATATTGGTT

GATGAATTGCTATTGTTGAAATATTTAATTTAAATTGTATATACGATTTATGCTTTACAT

GTACATTATTGTTATAATTTGAATTATGGTAATACCACTGAGTATGAATTACTCAGCGTA

CGGTTGTTTCCGTGCGCAGGTCAATAGAAGTCAAAGGTCTCGGTTCAGCATCCAGATTAA

TCCCGGCTTCGGCAAAACTTGGTGATGTATTTTTCCTTTGGTAAAGGTGGCATGTACATA

GATTGTGTATAAAGGTTATTATGTTTTATTATATAATGGTTAAAAATGTTAGTATTAAAA

GTTTATGGATTTTAATGAAAGAAGTCTATCTATTTTATCTAATTAGTACATTGTTAAATT

TTAAATTGGTATTGTGTAGATTGAGTTTGATTAGAAGTATTTAGAATAGAAAATGTGAAT

GTGAAATGAATTGGTTGAATTGATGATATTTGGGAACTATATGGTTTTAATTTGCAGGGG

GTTTTATGTAAAAATAAGCAGAAATGCTGCCGAAATTTTTATAAAAAAAAATGAAGTCAT

TTGGTAAACAAATTAATAAATTTTATGAATTATTTTAATATATTGGTTATTTATTTAAGA

ATTGTTGTAAATCGTTCGATACGTCCGGTAGTGCCTCGTAATTCTGTTCCGGCGACGGTT

CGGGGTTAAGGGGTGTTACATTTTATGGTATCAGAGCTATCAGGTTTAGCCGATTCTCGG

CCTAAATCGAGCTCGGAATTGAGTCTAGATGTACATGCCACTGTCGAGTTAAACTGAGTC

GGGATTTTTGGATGCTGACCTATTTGTTTGTTTTGTTTTATAGATTAAAGATGTCTGAAG

AAAGAATAAATGATACTGATGAAAGAATGTATAGTGAAGATAGAGAATTAGATGAAACAG

AATCTGTTGCACCGAGTGTGAATCCGTTAGGCAACCAACCTTCTAATGTAGAACGAGAAA

ATGTCAGAGATAGAGATGAATCCCAATTACTGAGAATTATAGCTGATGCATTACAAAGAG

TAGCAGGAACTACTCCTGTTACGACTTCAGTACCTACTGTTAGACGGGCTCCGATAAAGG

AACTGAGGAAATATGGTGCCACTGAATTTATGGGTCTAAAAGGAGTTGATCCATCCATAG

CTGAAAATTGGATGGAGTCGACTAAAAGAATTTTGCAGCAATTGGATTGTACCCCCCGAG

AGTGTTTAATCTGTGCCGTATCGTTATTACAAGGGGAGGCTTATCTATGGTGGGAATCAG

TGGTTCGACATTTACCAGAGAGTCAGATAACGTGGGATCTATTTCAGAAGGAGTTTCAAA

AGAAATATATCGGAGAGATGTATATTGAAGACAAGAAACAAGAGTTTTTGTTGCTACAAC

AGGGTGATATGTCAGTAATAGATTATGAGAGGGAATTCTCGAGACTCAGTAGATATGCCT

CCGAGTTTATTCCGACAGAAGCCGATAGTTGTAAAAGATTTTTACGGGGTTTACGAGACG

AGATCAAAGTGCAGCTAGTATCCCATCGGATCACTGAGTTAGTAGATTTGATTGAACGAG

CTAAAATGGTGGAACAAGTTCTGGGCCTCGACAAAAAGACTGAAGTTGTTAGACCAACCG

GGAAGCGTACAGGAACTACCAGTTCGAATCCTCAGCCGAAAAGACCAAAGGAATTCCAAA

GTGGTTGGAGATCCAGTTTCAGGTCAGACAGAGGTGGTAGAAATAGGGGAAAACAGACGA

TGACATCTACTGGCAGTGTGAAAGGTCCTTCCCGAGAAATAGATATTCCAGACTGCCAAC

ACTGCGGAAAGAAACACAGAGGGGAATGTTGGAAATTAACTAGAGGCTGTTTTCGATGTG

GTTCTACAGACCATTTCATCAGAGACTGTCCGAAAGTTGATAGTACTGTACCCGTGACAT

CACAGAGATCGGTATCTACAGCTAGAGGCAGAGGGTTAGGAAGAGGTGGTTCGGTTTCAA

GGGGAGGAAGTATTAGGAGAAGCAATGATATTGCTACTCAGCAGTCTGAGGCTAAAGTAC

CTGCCAGAGCTTATGTGGTCAGAACACAGGAAGAAGGTGACGCCCACGATGTAGTAACAG

GTATATTCTTACTATATTCTGAGCCTGTTTATGCTTTAATTGATCCCGGATCTTCACATT

CTTATATAAATTCAAAATTAGTTGAATTGGGAAAATTTAATTCTGAAATATCTAGAGTGA

CTGTAGAAGTGTCGAGTCCGTTGGGGCAAACAGTATTAGTGAATCAGATCTGTCCGAGAT

GCCCGTTAATTATACAAAATAAAACTTTTCCTATTGACCTGTTGATTATGCCATTTGGAG

ATTTTGATATAATACTGGGGATGGATTGGTTGGCTGAGCACGGAGTGGTATTGGATTGTT

ATAAAAAGAAGTTTAGTATTCAGACAGAAGACGGGGACAGAATTGAAGTAAATGGTATCC

GTACTAATGGGCCGACACGTATTATTTCGGCAATAAAGGCTAATAAATTGCTTCAGCGGG

GTTGTACAGCGTATTTAGCCTATGTTATTAATTCTGATTTGGTTGGTAGTCAGTGCAGTA

AGATTAGAACCGTATGTGAGTTTCCAGATGTATTTCCTGAAGAGCTACCGGGTTTACCAC

CTGACAGAGAGGTTGAATTTGCTATAGAAGTGTATCCGGGTACAGCACCAATCTCTATAC

CACCGTATCGAATGTCACCCACTGAGTTGAAAGAGTTGAAAGTGCAGTTACAGGACTTGT

CAGATCGTGGATTTATTAGACCGAGCATCTCACCTTGGGGAGCTCCAGTATTGTTTGTTA

AAAAGAAAGATGGATCGATGCGGCTTTGTATTGATTACCGGCAGTTAAACAAAGTGACGA

TCAAGAACCGGTATCCGTTACCCCGTATAGATGATTTATTTGATCAACTAAAAGGAGCTT

CAGTATTTTCAAAGATTGACTTAAGATCTGGGTATTATCAGCTGAAGGTAAAAGAAAGTG

ATGTTCCGAAGACTGCATTTCGTACTCGATATGGTCATTATGAATTTTTGGTGATGCCGT

TCGGGTTGACTAATGCTCCAGCTGCTTTTATGGATCTGATGAATCGTATTTTTCAGCCGT

ATTTAGATCAGTTTGTGGTGGTTTTTATTGATGACATCTTGGTTTATTCGAAGTCAGAGT

CAGAGCATGATCAGCATCTCAGAACCGTGCTACAAATTCTGCGAGAAAAACAGTTGTACG

GGAAACTAAGTAAATGTGAATTCTGGTTATCAGAGGTAGTATTCTTGGGACATGTTGTAT

CTGCGGATGGGATTAGAGTTGATCCGAAGAAGATCGAGGCAATTGTTCAATGGAAGGCAC

CAAAGAATGTATCAGAGGTACGCAGTTTTCTTGGTTTGGCTGGGTATTACAGAAGATTTG

TAAATGGGTTTTCGAAGATAGCTTTGCCGATGACCAAATTACTACAGAAGAATGTTCCAT

TTATCTGGGATGATCAGTGTCAGAGGAGCTTTGAAACATTGAAACAGATGTTGACAGAGG

CACCAGTTTTAACTTTACCAGAATCAGGGAAAGATTTCATAGTGTACAGTGATGCTTCTT

TGAATGGTTTGGGTTGTGTATTGATGCAAGAAGGAAAAGTAATAGCTTATGCATCTCGAC

AGTTGAAGTCACATGAACGCAACTACCCGACACACGATTTAGAGTTAGCTGCTGTAATCT

TTGCATTGAAGATTTGGATACATTACTTGTATGGTGAGAAATGTTATATTTACACTGATC

ATAAAAGTCTAAAATATCTTCTGTCACAAAAGGAGTTGAATCTGAGACAGAGACGGTGGA

TTGAACTTCTGAAAGATTATGATTGTGTTATAGATTATCATCCAGGGAAGGCAAATGTGG

TAGCAGATGCATTGAGTAGAAAAGCAGCGATTGAATTACGAGCAATGTTCGCTCGACTTA

GTATTAAGGATGATGGAAGTTTGTTAGCTGAGTTAAGAGTCAAGCCGGTGATGTTTGATC

AAATCAGAGCAGCACAGTTAAAAGATGAAAAGTTGATGAGGAAAAGAGAAATGGTACAGT

ATGGTGCGGTAGAAAATTTTAGTATTGACGAGCATGATTGTTTGAGATTTCGAAATCGAA

TTTGTGTTCCATCTACTTCTGAGATTAAAGAATTGATTCTCCGAGAAGCACATAATAGTA

TTTTTGCTTTGCACCCAGGAGGAACGAAGATGTATCGTGATCTACGAGAACTGTATTGGT

GGCCAGGAATGAAGAAAGATATAGTTGAATATGTCAGTAAATGCTTGACTTGTCAGCGGG

TAAAAGCAGAACATCAGGTACCAACAGGCCTGTTACAGCCTATTACTATTCCCGAGTGGA

AATGGGATCGCATTACCATGGATTTTGTTACGGGGTTGCCATTGTCAGTGAGTAAAAAGA

ATGCTATTTGGGTGATTGTTGATCGACTCACAAAATCAGCTCATTTTATAGCAGTTAGAA

CCGACTGGTCATTACAGAAGCTTGCCGAGGTTTATATTCGAGAAATTGTTAGATTACATG

GTATTCCGGTATCAATAATTTCAGACAGAGATCCTCGATTCACTTCGAGATTTTGGAAGC

AGCTGCATGAATCATTGGGTACTCGACTTAGTTTCAGTACAGCTTTTCATCCTCAAACTG

ATGGACAATCTGAACGAGTAATTCAGATATTAGAAGATATGCTTCGAGCTTGTGTCATTG

ATTTTGAATCAGGTTGGGAACGTTATTTACCATTGGCCGAGTTTGTTTATAATAATAGTT

TCCAATCTAGTATTCAAATGGCTCCATATGAAGCACTTTATGGTCGAAGGTGTCGATCAC

CAATATGTTGGACAAAATTAAGAGAAAGAAAAGTGATTGGGCCGGAATTGATTCAAGAGA

CAGAAGAAACAGTTAAAAAGATTAAAGATAGACTGAAAGCCGCTTTCGACAGACAGAAAT

CTTACGCAGACTTGAAACGACGAGACATTGAATATTCCGTTGGTGATAAGGTATTCCTCA

AAGTATCGCCGTGGAAGAAAATTTTGAGATTTGGTCGGAAGGGAAAATTAAGTCCGCGCT

TTATTGGGCCGTATGAGATAGTGGAAAGAATTGGGCCTGTTGCTTATCGATTATCCTTAC

CTCCAGAGTTACAGAAAATTCATGATGTTTTTCATGTTTCGATGCTTCGGAGATATAGAT

CGGATCCTTCTCATGTTATTCCCACTGAAGACATTGAACTTCGATCTGATTTAACTTATG

AAGAAGAACCAGTTCAAATATTAGCACGAGAAGTGAAAGAATTAAGAAATAAACGGGTTC

CTTTAGTACAAGTTTTATGGAGAAGCCATAGTGTGGAAGAAGCAACTTGGGAACCGGAAG

AGACAATGAGAGCACAATATCCTCATCTCTTCTCAGGTAAATTTCGAGGACGAAATTTAT

TAAGAGGGGGAGAAATGTAATGACCTAAAATTCATGGGCATCGGAAAAGTATAATATTGG

GCCTCCGTCCTAGTAAATTGAGTCCGAAAATAATTATTAGAAATATTTACGAGACTAGTA

GTGTGTTTAATTAGGTTTTAATTAAGTAAATTTAGCTTAATTTAGAGTAATTAGTAAAAA

GGATTAAATTGAATAAGAGTAAAAGTTTAATTATAGATTAAAGGAAAATAATAGGGACCA

AATGGGCAATTAAGCCACATTTGGAAGTTGAGGCGGCATAACATTGTAAAAATCTTAGAT

TTTTATATTATTATTTATATAAATATATAAATTAATTATAAAGTATATTATTAAATTAAT

TATATTATAAATATTATATTATTATATATAAAAGAAACAAAACAGAAAAGAAACAGAATA

GAAAGAACAAAGAAACAGAATAGAAGAGACGAAACAGGGGAGAAGCAGGGGAGAAAGAAG

AAAAAGAAGAAAAAAGGGGAAATAGGGTTTTTGAAGCTTGAAATTTAAATTGGTAAGTCA

AATTAGCCATTTTCTCTTAATTCTAATGTTTTAAAAGCTTTAAAACAAAGTTTTGATGGA

ATTAAGTTGATATTTTGTAAGTTCATAGGTTTTCAAGTATAGTTTATGTTGAACAAAAGA

GATGAATTAGGGATTAACTTGAAGGAATTTTAAGTTAGAATTGAAAAAGGGATTAAATTG

TAAAAGAAACTATAAGTTTTTTTTGTTTTAGGGACTAGATTGAGGAAAATTCGGAATTAA

GAAAATATGTTAAAAATTTAATAGTTAAATTTGAGTTTAAATGAAATTTGAATAGGAATA

AGGTGTGAATTGGTGTTATAAATTTGGTTATTAACATTTTTAATCAAAACAGTTTTGGGA

AGTAGCAATGGTCTGACTTTGAAAATTCACTAAAAATTTTATAAATTGAACTAGAGGATG

AACAAAATATGGAATTAAAGCTTATTGAGTCTAGTTTCTTATAGTAGAAACAATGTAAGC

AATTAATTGATGAATCAAGAGATATTTGAAATTTTGTAATACTGGTTCGGGGTGATTTCG

AGATGCCCTGTTTTAACTTTGGAAAATCATTAAAAATTGTACAAAAATTATTATGGAGTG

TAATTTATATATGTAAACTCCTTAATGAATCTAGTTTCAAAATAAATAAACAAGAACCTT

ATTCGAGTTCTGTACAATGAGATAATTTAGTTTTAGTGGAGAGAGGTCAGAACTGTCAAA

TGAAATAACAGGGGAGTATTTAACGAATAAACTGTATTAAATGGCTAGACCAAAAATTCT

GGAAATTTTATGATTAGAAGATATATGAGTCTAGTTTTAAGGAAAATTTACGGATATTAA

TTTGGAGTTTCGTAGCTCAAGATATAAATAATTTAGTAACAATGACCCAAGTAGACAGCT

TAATGGTGAAATTATATAAATACATTAAAAATGGTTAAATTTGCATGTTTAGGCTCATGA

ATTAAATTGAATCATGTTGTATTGATTATTATAAATTATTATTTTCGTAGCCAACAAAGA

ACCTAAAGCATCAGCATCGAAAGGAAAGGAGAAAGTCATCGAGGAGTAAACTCGAGAAAA

TTACGGTTTGTATTACTATAATTCAAGTTATTTATTATTAAATGTTAAATTTTAATTTAT

GTGTCTAGTAAATGAAATGTGAGGTAAGTATTATTATTATTATTATTATTATTATTATTA

TTATTATTATTATTATTATGAGTGGGAATTAAATTGAATAGTTGATATGAAATAATATTT

GAATTGTTTGTTGATTGAAAGCGGGAAATGAATTTAAATCGAATAGTGACCGATATTAAA

TTGAATGGAAATGTATTGAGTTGTGAAAATATGTTAATTGCGGATTAATTATTGATTGAA

AGGTGGAAAAATGATTGAATTGAAAGTGTGAGAAAGTGTGATTGAATTGGGATTATATGT

GATTTAAATACCCTATTAACTAGTCGGGCTGAGTCGGATATAGTTGGCATGCCATAGGAT

TGGAAGAGTTCAGGGATACTTCGACCTCGAGTCGATGAGACACTGGGTGTCACTATATTT

CTTCGGATAGATTCGATGAGGTACTGGGTACCAACTTTCTTCGGCTTTGCCGATGAGACA

CTGGGTGTCAACTATTGCTTCGAACTATCCGATGAGGCACTGGGTGCCATTCTGGTGTGT

TTGGTTGGATCCGTGTATTCGCCAAAGTCCGAGTTTTGTTAATAGGGTAAATGATGAAAT

GATAAACCGAACGAGTTGGTCAAACGAGCTATTGAAATGATATGAAAAAGTTGAATTGTG

AATTGAAATGTGAAATGAGATTGAGAAATGAACCTAAGGTTCGTGAATTATTCAAACTCA

AATTGTGGATATACGATATTGGTTGATGAATTGCTATTGTTGAAATATTTAATTTAAATT

GTATATACGATTTATGCTTTACATGTACATTATTGTTATAATTTGAATTATGGTAATACC

ACTGAGTATGAATTACTCAGCGTACGGTTGTTTCCGTGCGCAGGTCAATAGAAGTCAAAG

GTCTCGGTTCAGCATCCAGATTAATCCCGGCTTCGGCAAAACTTGGTGATGTATTTTTCC

TTTGGTAAAGGTGGCATGTACATAGATTGTGTATAAAGGTTATTATGTTTTATTATATAA

TGGTTAAAAATGTTAGTATTAAAAGTTTATGGATTTTAATGAAAGAAGTCTATCTATTTT

ATCTAATTAGTACATTGTTAAATTTTAAATTGGTATTAGATTGAGTTTGATTAGAAGTAT

TTAGAATAGAAAATGTGAATGTGAAATGAATTGGTTGAATTGATGATATTTGGGAACTAT

ATGGTTTTAATTTGC

>BR636

ATGTAATGACCCAAAATTCATGGGCATCGGAAAAGTATAATATCGGGCCTCCGTCCTAGT

AAATTGAGTCCGAAAATAATTATTAGAAATATTTACGAGACTAGTAGTGTGTTTAATTAG

GTTTTAATTAAGTAAATTTAGCTTAATTTAGAGTAATTAGTAAAAAGGATTAAATTGAAT

AAGAGTAAAAGTTTAATTATAGATTAAAGGAAAATAATAGGGACCAAATGGGCAATTAAG

CCACATTTGGAAGTTGAGGCGGCATAACATTGTAAAAATCTTAGATTTTTATATTATTAT

TTATATAAATATATAAATTAATTATAAAGTATATTATTAAATTAATTATATTATAAATAT

TATATTATTATATATAAAAGAAACAAAACAGAAAAGAAACAGAATAGAAAGAACAAAGAA

ACAGAATAGAAGAGACGAAACAGGGGAGAAGCAGGGGAGAAAGAAGAAAAAGAAGAAAAA

AGGGGAAATAGGGTTTTTGAAGCTTGAAATTTAAATTGGTAAGTCAAATTAGCCATTTTC

TCTTAATTCTAATGTTTTAAAAGCTTTAAAACAAAGTTTTGATGGAATTAAGTTGATATT

TTGTAAGTTCATAGGTTTTCAAGTATAGTTTATGTTGAACAAAAGAGATGAATTAGGGAT

TAACTTGAAGGAATTTTAAGTTAGAATTGAAAAAGGGATTAAATTGTAAAAGAAACTATA

AGTTTTTTTTGTTTTAGGGACTAGATTGAGGAAAATTCGGAATTAAGAAAATATGTTAAA

AATTTAATAGTTAAATTTGAGTTTAAATGAAATTTGAATAGGAATAAGGTGTGAATTGGT

GTTATAAATTTGGTTATTAACATTTTTAATCAAAACAGTTTTGGGAAGTAGCAATGGTCT

GACTTTGAAAATTCACTAAAAATTTTATAAATTGAACTAGAGGATGAACAAAATATGGAA

TTAAAGCTTATTGAGTCTAGTTTCTTATAGTAGAAACAATGTAAGCAATTAATTGATGAA

TCAAGAGATATTTGAAATTTTGTAATACTGGTTCGGGGTGATTTCGAGATGCCCTGTTTT

AACTTTGGAAAATCATTAAAAATTGTACAAAAATTATTATGGAGTGTAATTTATATATGT

GAACTCCTTAATGAATCTAGTTTCAAAATAAATAAACAAGAACCTTATTCGAGTTCTGTA

CAATGAGATAATTTAGTTTTAGTGGAGAGAGGTCAGAACTGTCAAATGAAATAACAGGGG

AGTATTTAACGAATAAACTGTATTAAATGGCTAGACCAAAAATTCTGGAAATTTTATGAT

TAGAAGATATATGAGTCTAGTTTTAAGGAAAATTTACGGATATTAATTTGGAGTTTCGTA

GCTCAAGATATAAATAATTTAGTAACAATGACCCAAGTAGACAGCTTAATGGTGAAATTA

TATAAATACATTAAAAATGGTTAAATTTGCATGTTTAGGCTCATGAATTAAATTGAATCA

TGTTGTATTGATTATTATAAATTATTATTTTCGTAGCCAACAAAGAACCTAAAGCATCAG

CATCGAAAGGAAAGGAGAAAGTCATCGAGGAGTAAACTCGAGAAAATTACGGTTTGTATT

ACTATAATTCAAGTTATTTATTATTAAATGTTAAATTTTAATTTATGTGTCTAGTAAATG

AAATGTGAGGTAAGTATTATTATTATTATTATTATTATTATTATTATTATTATTATTATT

ATGAGTGGGAATTAAATTGAATAGTTGATATGAAATAATATTTGAATTGTTTGTTGATTG

AAAGCGGGAAATGAATTTAAATCGAATAGTGACCGATATTAAATTGAATGGAAATGTATT

GAGTTGTGAAAATATGTTAATTGCGGATTAATTATTGATTGAAAGGTGGAAAAATGATTG

AATTGAAAGTGTGAGAAAGTGTGATTGAATTGGGATTATATGTGATTTAAATACCCTATT

AACTAGTCGGGCTGAGTCGGATATAGTTGGCATGCCATAGGATTGGAAGAGTTCAGGGAT

ACTTCGACCTCGAGTCGATGAGACACTGGGTGATTTCTTCGGATAGATTGGATGAGGTAC

TGGGTACCAACTTTCTTCGGCTTTGCCGATGAGACACTGGGTGTCAACTATTGCTTCGAA

CTATCCGATGAGGCACTGGGTGCCATTCTGGTGTGTTTGGTTGGATCCGTGTATCCGCCA

AAGTCCGAGTTTTGTTAATAGGGTAAATGATGAAATGATAAACCGAACGAGTTGGTCAAA

CGAGCTATTGAAATGATATGAAAAAGTTGAATTGTGAATTGAAATGTGAAATGAGATTGA

GAAATGAACCTAAGGTTCGTGAATTATTCAAACTCAAATTGTGGATATACGATATTGGTT

GATGAATTGCTATTGTTGAAATATTTAATTTAAATTGTATATACGATTTATGCTTTACAT

GTACATTATTGTTATAATTTGAATTATGGTAATACCACTGAGTATGAATTACTCAGCGTA

CGGTTGTTTCCGTGCGCAGGTCAATAGAAGTCAAAGGTCTCGGTTCAGCATCCAGATTAA

TCCCGGCTTCGGCAAAACTTGGTGATGTATTTTTCCTTTGGTAAAGGTGGCATGTACATA

GATTGTGTATAAAGGTTATTATGTTTTATTATATAATGGTTAAAAATGTTAGTATTAAAA

GTTTATGGATTTTAATGAAAGAAGTCTATCTATTTTATCTAATTAGTACATTGTTAAATT

TTAAATTGGTATTGTGTAGATTGAGTTTGATTAGAAGTATTTAGAATAGAAAATGTGAAT

GTGAAATGAATTGGTTGAATTGATGATATTTGGGAACTATATGGTTTTAATTTGCAGGGG

GTTTTATGTAAAAATAAGCAGAAATGCTGCCGAAATTTTTATAAAAAAAAATGAAGTCAT

TTGGTAAACAAATTAATAAATTTTATGAATTATTTTAATATATTGGTTATTTATTTAAGA

ATTGTTGTAAATCGTTCGATACGTCCGGTAGTGCCTCGTAATTCTGTTCCGGCGACGGTT

CGGGGTTAAGGGGTGTTACATTTTATGGTATCAGAGCTATCAGGTTTAGCCGATTCTCGG

CCTAAATCGAGCTCGGAATTGAGTCTAGATGTACATGCCACTGTCGAGTTAAACTGAGTC

GGGATTTTTGGATGCTGACCTATTTGTTTGTTTTGTTTTATAGATTAAAGATGTCTGAAG

AAAGAATAAATGATACTGATGAAAGAATGTATAGTGAAGATAGAGAATTAGATGAAACAG

AATCTGTTGCACCGAGTGTGAATCCGTTAGGCAACCAACCTTCTAATGTAGAACGAGAAA

ATGTCAGAGATAGAGATGAATCCCAATTACTGAGAATTATAGCTGATGCATTACAAAGAG

TAGCAGGAACTACTCCTGTTACGACTTCAGTACCTACTGTTAGACGGGCTCCGATAAAGG

AACTGAGGAAATATGGTGCCACTGAATTTATGGGTCTAAAAGGAGTTGATCCATCCATAG

CTGAAAATTGGATGGAGTCGACTAAAAGAATTTTGCAGCAATTGGATTGTACCCCCCGAG

AGTGTTTAATCTGTGCCGTATCGTTATTACAAGAGGAGGCTTATCTATGGTGGGAATCAG

TGGTTCGACATTTACCAGAGAGTCAGATAACGTGGGATCTATTTCAGAAGGAGTTTCAAA

AGAAATATATCGGAGAGATGTATATTGAAGACAAGAAACAAGAGTTTTTGTTGCTACAAC

AGGGTGATATGTCAGTAATAGATTATGAGAGGGAATTCTCGAGACTCAGTAGATATGCCT

CCGAGTTTATTCCGACAGAAGCCGATAGTTGTAAAAGATTTTTACGGGGTTTACGAGACG

AGATCAAAGTGCAGCTAGTATCCCATCGGATCACTGAGTTAGTAGATTTGATTGAACGAG

CTAAAATGGTGGAACAAGTTCTGGGCCTCGACAAAAAGACTGAAGTTGTTAGACCAACCG

GGAAGCGTACAGGAACTACCAGTTCGAATCCTCAGCCGAAAAGACCAAAGGAATTCCAAA

GTGGTTGGAGATCCAGTTTCAGGTCAGACAGAGGTGGTAGAAATAGGGGAAAACAGACGA

TGACATCTACTGGCAGTGTGAAAGGTCCTTCCCGAGAAATAGATATTCCAGACTGCCAAC

ACTGCGGAAAGAAACACAGAGGGGAATGTTGGAAATTAACTAGAGGCTGTTTTCGATGTG

GTTCTACAGACCATTTCATCAGAGACTGTCCGAAAGTTGATAGTACTGTACCCGTGACAT

CACAGAGATCGGTATCTACAGCTAGAGGCAGAGGGTTAGGAAGAGGTGGTTCGGTTTCAA

GGGGAGGAAGTATTAGGAGAAGCAATGATATTGCTACTCAGCAGTCTGAGGCTAAAGTAC

CTGCCAGAGCTTATGTGGTCAGAACACAGGAAGAAGGTGACGCCCACGATGTAGTAACAG

GTATATTCTTACTATATTCTGAGCCTGTTTATGCTTTAATTGATCCCGGATCTTCACATT

CTTATATAAATTCAAAATTAGTTGAATTGGGAAAATTTAATTCTGAAATATCTAGAGTGA

CTGTAGAAGTGTCGAGTCCGTTGGGGCAAACAGTATTAGTGAATCAGATCTGTCCGAGAT

GCCCGTTAATTATACAAAATAAAACTTTTCCTATTGACCTGTTGATTATGCCATTTGGAG

ATTTTGATATAATACTGGGGATGGATTGGTTGGCTGAGCACGGAGTGGTATTGGATTGTT

ATAAAAAGAAGTTTAGTATTCAGACAGAAGACGGGGACAGAATTGAAGTAAATGGTATCC

GTACTAATGGGCCGACACGTATTATTTCGGCAATAAAGGCTAATAAATTGCTTCAGCGGG

GTTGTACAGCGTATTTAGCCTATGTTATTAATTCTGATTTGGTTGGTAGTCAGTGCAGTA

AGATTAGAACCGTATGTGAGTTTCCAGATGTATTTCCTGAAGAGCTACCGGGTTTACCAC

CTGACAGAGAGGTTGAATTTGCTATAGAAGTGTATCCGGGTACAGCACCAATCTCTATAC

CACCGTATCGAATGTCACCCACTGAGTTGAAAGAGTTGAAAGTGCAGTTACAGGACTTGT

CAGATCGTGGATTTATTAGACCGAGCATCTCACCTTGGGGAGCTCCAGTATTGTTTGTTA

AAAAGAAAGATGGATCGATGCGGCTTTGTATTGATTACCGGCAGTTAAACAAAGTGACGA

TCAAGAACCGGTATCCGTTACCCCGTATAGATGATTTATTTGATCAACTAAAAGGAGCTT

CAGTATTTTCAAAGATTGACTTAAGATCTGGGTATTATCAGCTGAAGGTAAAAGAAAGTG

ATGTTCCGAAGACTGCATTTCGTACTCGATATGGTCATTATGAATTTTTGGTGATGCCGT

TCGGGTTGACTAATGCTCCAGCTGCTTTTATGGATCTGATGAATCGTATTTTTCAGCCGT

ATTTAGATCAGTTTGTGGTGGTTTTTATTGATGACATCTTGGTTTATTCGAAGTCAGAGT

CAGAGCATGATCAGCATCTCAGAACCGTGCTACAAATTCTGCGAGAAAAACAGTTGTACG

GGAAACTAAGTAAATGTGAATTCTGGTTATCAGAGGTAGTATTCTTGGGACATGTTGTAT

CTGCGGATGGGATTAGAGTTGATCCGAAGAAGATCGAGGCAATTGTTCAATGGAAGGCAC

CAAAGAATGTATCAGAGGTACGCAGTTTTCTTGGTTTGGCTGGGTATTACAGAAGATTTG

TAAATGGGTTTTCGAAGATAGCTTTGCCGATGACCAAATTACTACAGAAGAATGTTCCAT

TTATCTGGGATGATCAGTGTCAGAGGAGCTTTGAAACATTGAAACAGATGTTGACAGAGG

CACCAGTTTTAACTTTACCAGAATCAGGGAAAGATTTCATAGTGTACAGTGATGCTTCTT

TGAATGGTTTGGGTTGTGTATTGATGCAAGAAGGAAAAGTAATAGCTTATGCATCTCGAC

AGTTGAAGTCACATGAACGCAACTACCCGACACACGATTTAGAGTTAGCTGCTGTAATCT

TTGCATTGAAGATTTGGATACATTACTTGTATGGTGAGAAATGTTATATTTACACTGATC

ATAAAAGTCTAAAATATCTTCTGTCACAAAAGGAGTTGAATCTGAGACAGAGACGGTGGA

TTGAACTTCTGAAAGATTATGATTGTGTTATAGATTATCATCCAGGGAAGGCAAATGTGG

TAGCAGATGCATTGAGTAGAAAAGCAGCGATTGAATTACGAGCAATGTTCGCTCGACTTA

GTATTAAGGATGATGGAAGTTTGTTAGCTGAGTTAAGAGTCAAGCCGGTGATGTTTGATC

AAATCAGAGCAGCACAGTTAAAAGATGAAAAGTTGATGAGGAAAAGAGAAATGGTACAGT

ATGGTGCGGTAGAAAATTTTAGTATTGACGAGCATGATTGTTTGAGATTTCGAAATCGAA

TTTGTGTTCCATCTACTTCTGAGATTAAAGAATTGATTCTCCGAGAAGCACATAATAGTA

TTTTTGCTTTGCACCCAGGAGGAACGAAGATGTATCGTGATCTACGAGAACTGTATTGGT

GGCCAGGAATGAAGAAAGATATAGTTGAATATGTCAGTAAATGCTTGACTTGTCAGCAGG

TAAAAGCAGAACATCAGGTACCAACAGGCCTGTTACAGCCTATTACTATTCCCGAGTGGA

AATGGGATCGCATTACCATGGATTTTGTTACGGGGTTGCCATTGTCAGTGAGTAAAAAGA

ATGCTATTTGGGTGATTGTTGATCGACTCACAAAATCAGCTCATTTTATAGCAGTTAGAA

CCGACTGGTCATTACAGAAGCTTGCCGAGGTTTATATTCGAGAAATTGTTAGATTACATG

GTATTCCGGTATCAATAATTTCAGACAGAGATCCTCGATTCACTTCGAGATTTTGGAAGC

AGCTGCATGAATCATTGGGTACTCGACTTAGTTTCAGTACAGCTTTTCATCCTCAAACTG

ATGGACAATCTGAACGAGTAATTCAGATATTAGAAGATATGCTTCGAGCTTGTGTCATTG

ATTTTGAATCAGGTTGGGAACGTTATTTACCATTGGCCGAGTTTGTTTATAATAATAGTT

TCCAATCTAGTATTCAAATGGCTCCATATGAAGCACTTTATGGTCGAAGGTGTCGATCAC

CAATATGTTGGACAAAATTAAGAGAAAGAAAAGTGATTGGGCCGGAATTGATTCAAGAGA

CAGAAGAAACAGTTAAAAAGATTAAAGATAGACTGAAAGCCGCTTTCGACAGACAGAAAT

CTTACGCAGACTTGAAACGACGAGACATTGAATATTCCGTTGGTGATAAGGTATTCCTCA

AAGTATCGCCGTGGAAGAAAATTTTGAGATTTGGTCGGAAGGGAAAATTAAGTCCGCGCT

TTATTGGGCCGTATGAGATAGTGGAAAGAATTGGGCCTGTTGCTTATCGATTATCCTTAC

CTCCAGAGTTACAGAAAATTCATGATGTTTTTCATGTTTCGATGCTTCGGAGATATAGAT

CGGATCCTTCTCATGTTATTCCCACTGAAGACATTGAACTTCGATCTGATTTAACTTATG

AAGAAGAACCAGTTCAAATATTAGCACGAGAAGTGAAAGAATTAAGAAATAAACGGGTTC

CTTTAGTACAAGTTTTATGGAGAAGCCATAGTGTGGAAGAAGCAACTTGGGAACCGGAAG

AGACAATGAGAGCACAATATCCTCATCTCTTCTCAGGTAAATTTCGAGGACGAAATTTAT

TAAGAGGGGGAGAAATGTAATGACCTAAAATTCATGGGCATCGGAAAAGTATAATATTGG

GCCTCCGTCCTAGTAAATTGAGTCCGAAAATAATTATTAGAAATATTTACGAGACTAGTA

GTGTGTTTAATTAGGTTTTAATTAAGTAAATTTAGCTTAATTTAGAGTAATTAGTAAAAA

GGATTAAATTGAATAAGAGTAAAAGTTTAATTATAGATTAAAGGAAAATAATAGGGACCA

AATGGGCAATTAAGCCACATTTGGAAGTTGAGGCGGCATAACATTGTAAAAATCTTAGAT

TTTTATATTATTATTTATATAAATATATAAATTAATTATAAAGTATATTATTAAATTAAT

TATATTATAAATATTATATTATTATATATAAAAGAAACAAAACAGAAAAGAAACAGAATA

GAAAGAACAAAGAAACAGAATAGAAGAGACGAAACAGGGGAGAAGCAGGGGAGAAAGAAG

AAAAAGAAGAAAAAAGGGGAAATAGGGTTTTTGAAGCTTGAAATTTAAATTGGTAAGTCA

AATTAGCCATTTTCTCTTAATTCTAATGTTTTAAAAGCTTTAAAACAAAGTTTTGATGGA

ATTAAGTTGATATTTTGTAAGTTCATAGGTTTTCAAGTATAGTTTATGTTGAACAAAAGA

GATGAATTAGGGATTAACTTGAAGGAATTTTAAGTTAGAATTGAAAAAGGGATTAAATTG

TAAAAGAAACTATAAGTTTTTTTTGTTTTAGGGACTAGATTGAGGAAAATTCGGAATTAA

GAAAATATGTTAAAAATTTAATAGTTAAATTTGAGTTTAAATGAAATTTGAATAGGAATA

AGGTGTGAATTGGTGTTATAAATTTGGTTATTAACATTTTTAATCAAAACAGTTTTGGGA

AGTAGCAATGGTCTGACTTTGAAAATTCACTAAAAATTTTATAAATTGAACTAGAGGATG

AACAAAATATGGAATTAAAGCTTATTGAGTCTAGTTTCTTATAGTAGAAACAATGTAAGC

AATTAATTGATGAATCAAGAGATATTTGAAATTTTGTAATACTGGTTCGGGGTGATTTCG

AGATGCCCTGTTTTAACTTTGGAAAATCATTAAAAATTGTACAAAAATTATTATGGAGTG

TAATTTATATATGTAAACTCCTTAATGAATCTAGTTTCAAAATAAATAAACAAGAACCTT

ATTCGAGTTCTGTACAATGAGATAATTTAGTTTTAGTGGAGAGAGGTCAGAACTGTCAAA

TGAAATAACAGGGGAGTATTTAACGAATAAACTGTATTAAATGGCTAGACCAAAAATTCT

GGAAATTTTATGATTAGAAGATATATGAGTCTAGTTTTAAGGAAAATTTACGGATATTAA

TTTGGAGTTTCGTAGCTCAAGATATAAATAATTTAGTAACAATGACCCAAGTAGACAGCT

TAATGGTGAAATTATATAAATACATTAAAAATGGTTAAATTTGCATGTTTAGGCTCATGA

ATTAAATTGAATCATGTTGTATTGATTATTATAAATTATTATTTTCGTAGCCAACAAAGA

ACCTAAAGCATCAGCATCGAAAGGAAAGGAGAAAGTCATCGAGGAGTAAACTCGAGAAAA

TTACGGTTTGTATTACTATAATTCAAGTTATTTATTATTAAATGTTAAATTTTAATTTAT

GTGTCTAGTAAATGAAATGTGAGGTAAGTATTATTATTATTATTATTATTATTATTATTA

TTATTATTATTATTATTATGAGTGGGAATTAAATTGAATAGTTGATATGAAATAATATTT

GAATTGTTTGTTGATTGAAAGCGGGAAATGAATTTAAATCGAATAGTGACCGATATTAAA

TTGAATGGAAATGTATTGAGTTGTGAAAATATGTTAATTGCGGATTAATTATTGATTGAA

AGGTGGAAAAATGATTGAATTGAAAGTGTGAGAAAGTGTGATTGAATTGGGATTATATGT

GATTTAAATACCCTATTAACTAGTCGGGCTGAGTCGGATATAGTTGGCATGCCATAGGAT

TGGAAGAGTTCAGGGATACTTCGACCTCGAGTCGATGAGACACTGGGTGTCACTATATTT

CTTCGGATAGATTCGATGAGGTACTGGGTACCAACTTTCTTCGGCTTTGCCGATGAGACA

CTGGGTGTCAACTATTGCTTCGAACTATCCGATGAGGCACTGGGTGCCATTCTGGTGTGT

TTGGTTGGATCCGTGTATTCGCCAAAGTCCGAGTTTTGTTAATAGGGTAAATGATGAAAT

GATAAACCGAACGAGTTGGTCAAACGAGCTATTGAAATGATATGAAAAAGTTGAATTGTG

AATTGAAATGTGAAATGAGATTGAGAAATGAACCTAAGGTTCGTGAATTATTCAAACTCA

AATTGTGGATATACGATATTGGTTGATGAATTGCTATTGTTGAAATATTTAATTTAAATT

GTATATACGATTTATGCTTTACATGTACATTATTGTTATAATTTGAATTATGGTAATACC

ACTGAGTATGAATTACTCAGCGTACGGTTGTTTCCGTGCGCAGGTCAATAGAAGTCAAAG

GTCTCGGTTCAGCATCCAGATTAATCCCGGCTTCGGCAAAACTTGGTGATGTATTTTTCC

TTTGGTAAAGGTGGCATGTACATAGATTGTGTATAAAGGTTATTATGTTTTATTATATAA

TGGTTAAAAATGTTAGTATTAAAAGTTTATGGATTTTAATGAAAGAAGTCTATCTATTTT

ATCTAATTAGTACATTGTTAAATTTTAAATTGGTATTAGATTGAGTTTGATTAGAAGTAT

TTAGAATAGAAAATGTGAATGTGAAATGAATTGGTTGAATTGATGATATTTGGGAACTAT

ATGGTTTTAATTTGC

>Cleveland_WRWanr

AGGTAATGACCCAAAATTCATGGGCATCGGAAAAGTATAATATCGGGCCTCCGTCCTAGT

AAATTGAGTCCGAAAATAATTATTAGAAATATTTACGAGACTAGTAGTGTGTTTAATTAG

GTTTTAATTAAGTAAATTTAGCTTAATTTAGAGTAATTAGTAAAAAGGATTAAATTGAAT

AAGAGTAAAAGTTTAATTATAGATTAAAGGAAAATAATAGGGACCAAATGGGCAATTAAG

CCACATTTGGAAGTTGAGGCGGCATAACATTGTAAAAATCTTAGATTTTTATATTATTAT

TTATATAAATATATAAATTAATTATAAAGTATATTATTAAATTAATTATATTATAAATAT

TATATTATTATATATAAAAGAAACAAAACAGAAAAGAAACAGAATAGAAAGAACAAAGAA

ACAGAATAGAAGAGACGAAACAGGGGAGAAGCAGGGGAGAAAGAAGAAAAAGAAGAAAAA

AGGGGAAATAGGGTTTTTGAAGCTTGAAATTTAAATTGGTAAGTCAAATTAGCCATTTTC

TCTTAATTCTAATGTTTTAAAAGCTTTAAAACAAAGTTTTGATGGAATTAAGTTGATATT

TTGTAAGTTCATAGGTTTTCAAGTATAGTTTATGTTGAACAAAAGAGATGAATTAGGGAT

TAACTTGAAGGAATTTTAAGTTAGAATTGAAAAAGGGATTAAATTGTAAAAGAAACTATA

AGTTTTTTTTGTTTTAGGGACTAGATTGAGGAAAATTCGGAATTAAGAAAATATGTTAAA

AATTTAATAGTTAAATTTGAGTTTAAATGAAATTTGAATAGGAATAAGGTGTGAATTGGT

GTTATAAATTTGGTTATTAACATTTTTAATCAAAACAGTTTTGGGAAGTAGCAATGGTCT

GACTTTGAAAATTCACTAAAAATTTTATAAATTGAACTAGAGGATGAACAAAATATGGAA

TTAAAGCTTATTGAGTCTAGTTTCTTATAGTAGAAACAATGTAAGCAATTAATTGATGAA

TCAAGAGATATTTGAAATTTTGTAATACTGGTTCGGGGTGATTTCGAGATGCCCTGTTTT

AACTTTGGAAAATCATTAAAAATTGTACAAAAATTATTATGGAGTGTAATTTATATATGT

GAACTCCTTAATGAATCTAGTTTCAAAATAAATAAACAAGAACCTTATTCGAGTTCTGTA

CAATGAGATAATTTAGTTTTAGTGGAGAGAGGTCAGAACTGTCAAATGAAATAACAGGGG

AGTATTTAACGAATAAACTGTATTAAATGGCTAGACCAAAAATTCTGGAAATTTTATGAT

TAGAAGATATATGAGTCTAGTTTTAAGGAAAATTTACGGATATTAATTTGGAGTTTCGTA

GCTCAAGATATAAATAATTTAGTAACAATGACCCAAGTAGACAGCTTAATGGTGAAATTA

TATAAATACATTAAAAATGGTTAAATTTGCATGTTTAGGCTCATGAATTAAATTGAATCA

TGTTGTATTGATTATTATAAATTATTATTTTCGTAGCCAACAAAGAACCTAAAGCATCAG

CATCGAAAGGAAAGGAGAAAGTCATCGAGGAGTAAACTCGAGAAAATTACGGTTTGTATT

ACTATAATTCAAGTTATTTATTATTAAATGTTAAATTTTAATTTATGTGTCTAGTAAATG

AAATGTGAGGTAAGTATTATTATTATTATTATTATTATTATTATTATTATTATTATTATT

ATGAGTGGGAATTAAATTGAATAGTTGATATGAAATAATATTTGAATTGTTTGTTGATTG

AAAGCGGGAAATGAATTTAAATCGAATAGTGACCGATATTAAATTGAATGGAAATGTATT

GAGTTGTGAAAATATGTTAATTGCGGATTAATTATTGATTGAAAGGTGGAAAAATGATTG

AATTGAAAGTGTGAGAAAGTGTGATTGAATTGGGATTATATGTGATTTAAATACCCTATT

AACTAGTCGGGCTGAGTCGGATATAGTTGGCATGCCATAGGATTGGAAGAGTTCAGGGAT

ACTTCGACCTCGAGTCGATGAGACACTGGGTGATTTCTTCGGATAGATTGGATGAGGTAC

TGGGTACCAACTTTCTTCGGCTTTGCCGATGAGACACTGGGTGTCAACTATTGCTTCGAA

CTATCCGATGAGGCACTGGGTGCCATTCTGGTGTGTTTGGTTGGATCCGTGTATCCGCCA

AAGTCCGAGTTTTGTTAATAGGGTAAATGATGAAATGATAAACCGAACGAGTTGGTCAAA

CGAGCTATTGAAATGATATGAAAAAGTTGAATTGTGAATTGAAATGTGAAATGAGATTGA

GAAATGAACCTAAGGTTCGTGAATTATTCAAACTCAAATTGTGGATATACGATATTGGTT

GATGAATTGCTATTGTTGAAATATTTAATTTAAATTGTATATACGATTTATGCTTTACAT

GTACATTATTGTTATAATTTGAATTATGGTAATACCACTGAGTATGAATTACTCAGCGTA

CGGTTGTTTCCGTGCGCAGGTCAATAGAAGTCAAAGGTCTCGGTTCAGCATCCAGATTAA

TCCCGGCTTCGGCAAAACTTGGTGATGTATTTTTCCTTTGGTAAAGGTGGCATGTACATA

GATTGTGTATAAAGGTTATTATGTTTTATTATATAATGGTTAAAAATGTTAGTATTAAAA

GTTTATGGATTTTAATGAAAGAAGTCTATCTATTTTATCTAATTAGTACATTGTTAAATT

TTAAATTGGTATTGTGTAGATTGAGTTTGATTAGAAGTATTTAGAATAGAAAATGTGAAT

GTGAAATGAATTGGTTGAATTGATGATATTTGGGAACTATATGGTTTTAATTTGCAGGGG

GTTTTATGTAAAAATAAGCAGAAATGCTGCCGAAATTTTTATAAAAAAAAATGAAGTCAT

TTGGTAAACAAATTAATAAATTTTATGAATTATTTTAATATATTGGTTATTTATTTAAGA

ATTGTTGTAAATCGTTCGATACGTCCGGTAGTGCCTCGTAATTCTGTTCCGGCGACGGTT

CGGGGTTAAGGGGTGTTACATTTTATGGTATCAGAGCTATCAGGTTTAGCCGATTCTCGG

CCTAAATCGAGCTCGGAATTGAGTCTAGATGTACATGCCACTGTCGAGTTAAACTGAGTC

GGGATTTTTGGATGCTGACCTATTTGTTTGTTTTGTTTTATAGATTAAAGATGTCTGAAG

AAAGAATAAATGATACTGATGAAAGAATGTATAGTGAAGATAGAGAATTAGATGAAACAG

AATCTGTTGCACCGAGTGTGAATCCGTTAGGCAACCAACCTTCTAATGTAGAACGAGAAA

ATGTCAGAGATAGAGATGAATCCCAATTACTGAGAATTATAGCTGATGCATTACAAAGAG

TAGCAGGAACTACTCCTGTTACGACTTCAGTACCTACTGTTAGACGGGCTCCGATAAAGG

AACTGAGGAAATATGGTGCCACTGAATTTATGGGTCTAAAAGGAGTTGATCCATCCATAG

CTGAAAATTGGATGGAGTCGACTAAAAGAATTTTGCAGCAATTGGATTGTACCCCCCGAG

AGTGTTTAATCTGTGCCGTATCGTTATTACAAGGGGAGGCTTATCTATGGTGGGAATCAG

TGGTTCGACATTTACCAGAGAGTCAGATAACGTGGGATCTATTTCAGAAGGAGTTTCAAA

AGAAATATATCGGAGAGATGTATATTGAAGACAAGAAACAAGAGTTTTTGTTGCTACAAC

AGGGTGATATGTCAGTAATAGATTATGAGAGGGAATTCTCGAGACTCAGTAGATATGCCT

CCGAGTTTATTCCGACAGAAGCCGATAGTTGTAAAAGATTTTTACGGGGTTTACGAGACG

AGATCAAAGTGCAGCTAGTATCCCATCGGATCACTGAGTTAGTAGATTTGATTGAACGAG

CTAAAATGGTGGAACAAGTTCTGGGCCTCGACAAAAAGACTGAAGTTGTTAGACCAACCG

GGAAGCGTACAGGAACTACCAGTTCGAATCCTCAGCCGAAAAGACCAAAGGAATTCCAAA

GTGGTTGGAGATCCAGTTTCAGGTCAGACAGAGGTGGTAGAAATAGGGGAAAACAGACGA

TGACATCTACTGGCAGTGTGAAAGGTCCTTCCCGAGAAATAGATATTCCAGACTGCCAAC

ACTGCGGAAAGAAACACAGAGGGGAATGTTGGAAATTAACTAGAGGCTGTTTTCGATGTG

GTTCTACAGACCATTTCATCAGAGACTGTCCGAAAGTTGATAGTACTGTACCCGTGACAT

CACAGAGATCGGTATCTACAGCTAGAGGCAGAGGGTTAGGAAGAGGTGGTTCGGTTTCAA

GGGGAGGAAGTATTAGGAGAAGCAATGATATTGCTACTCAGCAGTCTGAGGCTAAAGTAC

CTGCCAGAGCTTATGTGGTCAGAACACAGGAAGAAGGTGACGCCCACGATGTAGTAACAG

GTATATTCTTACTATATTCTGAGCCTGTTTATGCTTTAATTGATCCCGGATCTTCACATT

CTTATATAAATTCAAAATTAGTTGAATTGGGAAAATTTAATTCTGAAATATCTAGAGTGA

CTGTAGAAGTGTCGAGTCCGTTGGGGCAAACAGTATTAGTGAATCAGATCTGTCCGAGAT

GCCCGTTAATTATACAAAATAAAACTTTTCCTATTGACCTGTTGATTATGCCATTTGGAG

ATTTTGATATAATACTGGGGATGGATTGGTTGGCTGAGCACGGAGTGGTATTGGATTGTT

ATAAAAAGAAGTTTAGTATTCAGACAGAAGACGGGGACAGAATTGAAGTAAATGGTATCC

GTACTAATGGGCCGACACGTATTATTTCGGCAATAAAGGCTAATAAATTGCTTCAGCGGG

GTTGTACAGCGTATTTAGCCTATGTTATTAATTCTGATTTGGTTGGTAGTCAGTGCAGTA

AGATTAGAACCGTATGTGAGTTTCCAGATGTATTTCCTGAAGAGCTACCGGGTTTACCAC

CTGACAGAGAGGTTGAATTTGCTATAGAAGTGTATCCGGGTACAGCACCAATCTCTATAC

CACCGTATCGAATGTCACCCACTGAGTTGAAAGAGTTGAAAGTGCAGTTACAGGACTTGT

CAGATCGTGGATTTATTAGACCGAGCATCTCACCTTGGGGAGCTCCAGTATTGTTTGTTA

AAAAGAAAGATGGATCGATGCGGCTTTGTATTGATTACCGGCAGTTAAACAAAGTGACGA

TCAAGAACCGGTATCCGTTACCCCGTATAGATGATTTATTTGATCAACTAAAAGGAGCTT

CAGTATTTTCAAAGATTGACTTAAGATCTGGGTATTATCAGCTGAAGGTAAAAGAAAGTG

ATGTTCCGAAGACTGCATTTCGTACTCGATATGGTCATTATGAATTTTTGGTGATGCCGT

TCGGGTTGACTAATGCTCCAGCTGCTTTTATGGATCTGATGAATCGTATTTTTCAGCCGT

ATTTAGATCAGTTTGTGGTGGTTTTTATTGATGACATCTTGGTTTATTCGAAGTCAGAGT

CAGAGCATGATCAGCATCTCAGAACCGTGCTACAAATTCTGCGAGAAAAACAGTTGTACG

GGAAACTAAGTAAATGTGAATTCTGGTTATCAGAGGTAGTATTCTTGGGACATGTTGTAT

CTGCGGATGGGATTAGAGTTGATCCGAAGAAGATCGAGGCAATTGTTCAATGGAAGGCAC

CAAAGAATGTATCAGAGGTACGCAGTTTTCTTGGTTTGGCTGGGTATTACAGAAGATTTG

TAAATGGGTTTTCGAAGATAGCTTTGCCGATGACCAAATTACTACAGAAGAATGTTCCAT

TTATCTGGGATGATCAGTGTCAGAGGAGCTTTGAAACATTGAAACAGATGTTGACAGAGG

CACCAGTTTTAACTTTACCAGAATCAGGGAAAGATTTCATAGTGTACAGTGATGCTTCTT

TGAATGGTTTGGGTTGTGTATTGATGCAAGAAGGAAAAGTAATAGCTTATGCATCTCGAC

AGTTGAAGTCACATGAACGCAACTACCCGACACACGATTTAGAGTTAGCTGCTGTAATCT

TTGCATTGAAGATTTGGATACATTACTTGTATGGTGAGAAATGTTATATTTACACTGATC

ATAAAAGTCTAAAATATCTTCTGTCACAAAAGGAGTTGAATCTGAGACAGAGACGGTGGA

TTGAACTTCTGAAAGATTATGATTGTGTTATAGATTATCATCCAGGGAAGGCAAATGTGG

TAGCAGATGCATTGAGTAGAAAAGCAGCGATTGAATTACGAGCAATGTTCGCTCGACTTA

GTATTAAGGATGATGGAAGTTTGTTAGCTGAGTTAAGAGTCAAGCCGGTGATGTTTGATC

AAATCAGAGCAGCACAGTTAAAAGATGAAAAGTTGATGAGGAAAAGAGAAATGGTACAGT

ATGGTGCGGTAGAAAATTTTAGTATTGACGAGCATGATTGTTTGAGATTTCGAAATCGAA

TTTGTGTTCCATCTACTTCTGAGATTAAAGAATTGATTCTCCGAGAAGCACATAATAGTA

TTTTTGCTTTGCACCCAGGAGGAACGAAGATGTATCGTGATCTACGAGAACTGTATTGGT

GGCCAGGAATGAAGAAAGATATAGTTGAATATGTCAGTAAATGCTTGACTTGTCAGCGGG

TAAAAGCAGAACATCAGGTACCAACAGGCCTGTTACAGCCTATTACTATTCCCGAGTGGA

AATGGGATCGCATTACCATGGATTTTGTTACGGGGTTGCCATTGTCAGTGAGTAAAAAGA

ATGCTATTTGGGTGATTGTTGATCGACTCACAAAATCAGCTCATTTTATAGCAGTTAGAA

CCGACTGGTCATTACAGAAGCTTGCCGAGGTTTATATTCGAGAAATTGTTAGATTACATG

GTATTCCGGTATCAATAATTTCAGACAGAGATCCTCGATTCACTTCGAGATTTTGGAAGC

AGCTGCATGAATCATTGGGTACTCGACTTAGTTTCAGTACAGCTTTTCATCCTCAAACTG

ATGGACAATCTGAACGAGTAATTCAGATATTAGAAGATATGCTTCGAGCTTGTGTCATTG

ATTTTGAATCAGGTTGGGAACGTTATTTACCATTGGCCGAGTTTGTTTATAATAATAGTT

TCCAATCTAGTATTCAAATGGCTCCATATGAAGCACTTTATGGTCGAAGGTGTCGATCAC

CAATATGTTGGACAAAATTAAGAGAAAGAAAAGTGATTGGGCCGGAATTGATTCAAGAGA

CAGAAGAAACAGTTAAAAAGATTAAAGATAGACTGAAAGCCGCTTTCGACAGACAGAAAT

CTTACGCAGACTTGAAACGACGAGACATTGAATATTCCGTTGGTGATAAGGTATTCCTCA

AAGTATCGCCGTGGAAGAAAATTTTGAGATTTGGTCGGAAGGGAAAATTAAGTCCGCGCT

TTATTGGGCCGTATGAGATAGTGGAAAGAATTGGGCCTGTTGCTTATCGATTATCCTTAC

CTCCAGAGTTACAGAAAATTCATGATGTTTTTCATGTTTCGATGCTTCGGAGATATAGAT

CGGATCCTTCTCATGTTATTCCCACTGAAGACATTGAACTTCGATCTGATTTAACTTATG

AAGAAGAACCAGTTCAAATATTAGCACGAGAAGTGAAAGAATTAAGAAATAAACGGGTTC

CTTTAGTACAAGTTTTATGGAGAAGCCATAGTGTGGAAGAAGCAACTTGGGAACCGGAAG

AGACAATGAGAGCACAATATCCTCATCTCTTCTCAGGTAAATTTCGAGGACGAAATTTAT

TAAGAGGGGGAGAAATGTAATGACCTAAAATTCATGGGCATCGGAAAAGTATAATATTGG

GCCTCCGTCCTAGTAAATTGAGTCCGAAAATAATTATTAGAAATATTTACGAGACTAGTA

GTGTGTTTAATTAGGTTTTAATTAAGTAAATTTAGCTTAATTTAGAGTAATTAGTAAAAA

GGATTAAATTGAATAAGAGTAAAAGTTTAATTATAGATTAAAGGAAAATAATAGGGACCA

AATGGGCAATTAAGCCACATTTGGAAGTTGAGGCGGCATAACATTGTAAAAATCTTAGAT

TTTTATATTATTATTTATATAAATATATAAATTAATTATAAAGTATATTATTAAATTAAT

TATATTATAAATATTATATTATTATATATAAAAGAAACAAAACAGAAAAGAAACAGAATA

GAAAGAACAAAGAAACAGAATAGAAGAGACGAAACAGGGGAGAAGCAGGGGAGAAAGAAG

AAAAAGAAGAAAAAAGGGGAAATAGGGTTTTTGAAGCTTGAAATTTAAATTGGTAAGTCA

AATTAGCCATTTTCTCTTAATTCTAATGTTTTAAAAGCTTTAAAACAAAGTTTTGATGGA

ATTAAGTTGATATTTTGTAAGTTCATAGGTTTTCAAGTATAGTTTATGTTGAACAAAAGA

GATGAATTAGGGATTAACTTGAAGGAATTTTAAGTTAGAATTGAAAAAGGGATTAAATTG

TAAAAGAAACTATAAGTTTTTTTTGTTTTAGGGACTAGATTGAGGAAAATTCGGAATTAA

GAAAATATGTTAAAAATTTAATAGTTAAATTTGAGTTTAAATGAAATTTGAATAGGAATA

AGGTGTGAATTGGTGTTATAAATTTGGTTATTAACATTTTTAATCAAAACAGTTTTGGGA

AGTAGCAATGGTCTGACTTTGAAAATTCACTAAAAATTTTATAAATTGAACTAGAGGATG

AACAAAATATGGAATTAAAGCTTATTGAGTCTAGTTTCTTATAGTAGAAACAATGTAAGC

AATTAATTGATGAATCAAGAGATATTTGAAATTTTGTAATACTGGTTCGGGGTGATTTCG

AGATGCCCTGTTTTAACTTTGGAAAATCATTAAAAATTGTACAAAAATTATTATGGAGTG

TAATTTATATATGTAAACTCCTTAATGAATCTAGTTTCAAAATAAATAAACAAGAACCTT

ATTCGAGTTCTGTACAATGAGATAATTTAGTTTTAGTGGAGAGAGGTCAGAACTGTCAAA

TGAAATAACAGGGGAGTATTTAACGAATAAACTGTATTAAATGGCTAGACCAAAAATTCT

GGAAATTTTATGATTAGAAGATATATGAGTCTAGTTTTAAGGAAAATTTACGGATATTAA

TTTGGAGTTTCGTAGCTCAAGATATAAATAATTTAGTAACAATGACCCAAGTAGACAGCT

TAATGGTGAAATTATATAAATACATTAAAAATGGTTAAATTTGCATGTTTAGGCTCATGA

ATTAAATTGAATCATGTTGTATTGATTATTATAAATTATTATTTTCGTAGCCAACAAAGA

ACCTAAAGCATCAGCATCGAAAGGAAAGGAGAAAGTCATCGAGGAGTAAACTCGAGAAAA

TTACGGTTTGTATTACTATAATTCAAGTTATTTATTATTAAATGTTAAATTTTAATTTAT

GTGTCTAGTAAATGAAATGTGAGGTAAGTATTATTATTATTATTATTATTATTATTATTA

TTATTATTATTATTATTATGAGTGGGAATTAAATTGAATAGTTGATATGAAATAATATTT

GAATTGTTTGTTGATTGAAAGCGGGAAATGAATTTAAATCGAATAGTGACCGATATTAAA

TTGAATGGAAATGTATTGAGTTGTGAAAATATGTTAATTGCGGATTAATTATTGATTGAA

AGGTGGAAAAATGATTGAATTGAAAGTGTGAGAAAGTGTGATTGAATTGGGATTATATGT

GATTTAAATACCCTATTAACTAGTCGGGCTGAGTCGGATATAGTTGGCATGCCATAGGAT

TGGAAGAGTTCAGGGATACTTCGACCTCGAGTCGATGAGACACTGGGTGTCACTATATTT

CTTCGGATAGATTCGATGAGGTACTGGGTACCAACTTTCTTCGGCTTTGCCGATGAGACA

CTGGGTGTCAACTATTGCTTCGAACTATCCGATGAGGCACTGGGTGCCATTCTGGTGTGT

TTGGTTGGATCCGTGTATTCGCCAAAGTCCGAGTTTTGTTAATAGGGTAAATGATGAAAT

GATAAACCGAACGAGTTGGTCAAACGAGCTATTGAAATGATATGAAAAAGTTGAATTGTG

AATTGAAATGTGAAATGAGATTGAGAAATGAACCTAAGGTTCGTGAATTATTCAAACTCA

AATTGTGGATATACGATATTGGTTGATGAATTGCTATTGTTGAAATATTTAATTTAAATT

GTATATACGATTTATGCTTTACATGTACATTATTGTTATAATTTGAATTATGGTAATACC

ACTGAGTATGAATTACTCAGCGTACGGTTGTTTCCGTGCGCAGGTCAATAGAAGTCAAAG

GTCTCGGTTCAGCATCCAGATTAATCCCGGCTTCGGCAAAACTTGGTGATGTATTTTTCC

TTTGGTAAAGGTGGCATGTACATAGATTGTGTATAAAGGTTATTATGTTTTATTATATAA

TGGTTAAAAATGTTAGTATTAAAAGTTTATGGATTTTAATGAAAGAAGTCTATCTATTTT

ATCTAATTAGTACATTGTTAAATTTTAAATTGGTATTAGATTGAGTTTGATTAGAAGTAT

TTAGAATAGAAAATGTGAATGTGAAATGAATTGGTTGAATTGATGATATTTGGGAACTAT

ATGGTTTTAATTTGC

>Coker100wilt

AGGTAATGACCCAAAATTCATGGGCATCGGAAAAGTATAATATCGGGCCTCCGTCCTAGT

AAATTGAGTCCGAAAATAATTATTAGAAATATTTACGAGACTAGTAGTGTGTTTAATTAG

GTTTTAATTAAGTAAATTTAGCTTAATTTAGAGTAATTAGTAAAAAGGATTAAATTGAAT

AAGAGTAAAAGTTTAATTATAGATTAAAGGAAAATAATAGGGACCAAATGGGCAATTAAG

CCACATTTGGAAGTTGAGGCGGCATAACATTGTAAAAATCTTAGATTTTTATATTATTAT

TTATATAAATATATAAATTAATTATAAAGTATATTATTAAATTAATTATATTATAAATAT

TATATTATTATATATAAAAGAAACAAAACAGAAAAGAAACAGAATAGAAAGAACAAAGAA

ACAGAATAGAAGAGACGAAACAGGGGAGAAGCAGGGGAGAAAGAAGAAAAAGAAGAAAAA

AGGGGAAATAGGGTTTTTGAAGCTTGAAATTTAAATTGGTAAGTCAAATTAGCCATTTTC

TCTTAATTCTAATGTTTTAAAAGCTTTAAAACAAAGTTTTGATGGAATTAAGTTGATATT

TTGTAAGTTCATAGGTTTTCAAGTATAGTTTATGTTGAACAAAAGAGATGAATTAGGGAT

TAACTTGAAGGAATTTTAAGTTAGAATTGAAAAAGGGATTAAATTGTAAAAGAAACTATA

AGTTTTTTTTGTTTTAGGGACTAGATTGAGGAAAATTCGGAATTAAGAAAATATGTTAAA

AATTTAATAGTTAAATTTGAGTTTAAATGAAATTTGAATAGGAATAAGGTGTGAATTGGT

GTTATAAATTTGGTTATTAACATTTTTAATCAAAACAGTTTTGGGAAGTAGCAATGGTCT

GACTTTGAAAATTCACTAAAAATTTTATAAATTGAACTAGAGGATGAACAAAATATGGAA

TTAAAGCTTATTGAGTCTAGTTTCTTATAGTAGAAACAATGTAAGCAATTAATTGATGAA

TCAAGAGATATTTGAAATTTTGTAATACTGGTTCGGGGTGATTTCGAGATGCCCTGTTTT

AACTTTGGAAAATCATTAAAAATTGTACAAAAATTATTATGGAGTGTAATTTATATATGT

GAACTCCTTAATGAATCTAGTTTCAAAATAAATAAACAAGAACCTTATTCGAGTTCTGTA

CAATGAGATAATTTAGTTTTAGTGGAGAGAGGTCAGAACTGTCAAATGAAATAACAGGGG

AGTATTTAACGAATAAACTGTATTAAATGGCTAGACCAAAAATTCTGGAAATTTTATGAT

TAGAAGATATATGAGTCTAGTTTTAAGGAAAATTTACGGATATTAATTTGGAGTTTCGTA

GCTCAAGATATAAATAATTTAGTAACAATGACCCAAGTAGACAGCTTAATGGTGAAATTA

TATAAATACATTAAAAATGGTTAAATTTGCATGTTTAGGCTCATGAATTAAATTGAATCA

TGTTGTATTGATTATTATAAATTATTATTTTCGTAGCCAACAAAGAACCTAAAGCATCAG

CATCGAAAGGAAAGGAGAAAGTCATCGAGGAGTAAACTCGAGAAAATTACGGTTTGTATT

ACTATAATTCAAGTTATTTATTATTAAATGTTAAATTTTAATTTATGTGTCTAGTAAATG

AAATGTGAGGTAAGTATTATTATTATTATTATTATTATTATTATTATTATTATTATTATT

ATGAGTGGGAATTAAATTGAATAGTTGATATGAAATAATATTTGAATTGTTTGTTGATTG

AAAGCGGGAAATGAATTTAAATCGAATAGTGACCGATATTAAATTGAATGGAAATGTATT

GAGTTGTGAAAATATGTTAATTGCGGATTAATTATTGATTGAAAGGTGGAAAAATGATTG

AATTGAAAGTGTGAGAAAGTGTGATTGAATTGGGATTATATGTGATTTAAATACCCTATT

AACTAGTCGGGCTGAGTCGGATATAGTTGGCATGCCATAGGATTGGAAGAGTTCAGGGAT

ACTTCGACCTCGAGTCGATGAGACACTGGGTGATTTCTTCGGATAGATTGGATGAGGTAC

TGGGTACCAACTTTCTTCGGCTTTGCCGATGAGACACTGGGTGTCAACTATTGCTTCGAA

CTATCCGATGAGGCACTGGGTGCCATTCTGGTGTGTTTGGTTGGATCCGTGTATCCGCCA

AAGTCCGAGTTTTGTTAATAGGGTAAATGATGAAATGATAAACCGAACGAGTTGGTCAAA

CGAGCTATTGAAATGATATGAAAAAGTTGAATTGTGAATTGAAATGTGAAATGAGATTGA

GAAATGAACCTAAGGTTCGTGAATTATTCAAACTCAAATTGTGGATATACGATATTGGTT

GATGAATTGCTATTGTTGAAATATTTAATTTAAATTGTATATACGATTTATGCTTTACAT

GTACATTATTGTTATAATTTGAATTATGGTAATACCACTGAGTATGAATTACTCAGCGTA

CGGTTGTTTCCGTGCGCAGGTCAATAGAAGTCAAAGGTCTCGGTTCAGCATCCAGATTAA

TCCCGGCTTCGGCAAAACTTGGTGATGTATTTTTCCTTTGGTAAAGGTGGCATGTACATA

GATTGTGTATAAAGGTTATTATGTTTTATTATATAATGGTTAAAAATGTTAGTATTAAAA

GTTTATGGATTTTAATGAAAGAAGTCTATCTATTTTATCTAATTAGTACATTGTTAAATT

TTAAATTGGTATTGTGTAGATTGAGTTTGATTAGAAGTATTTAGAATAGAAAATGTGAAT

GTGAAATGAATTGGTTGAATTGATGATATTTGGGAACTATATGGTTTTAATTTGCAGGGG

GTTTTATGTAAAAATAAGCAGAAATGCTGCCGAAATTTTTATAAAAAAAAATGAAGTCAT

TTGGTAAACAAATTAATAAATTTTATGAATTATTTTAATATATTGGTTATTTATTTAAGA

ATTGTTGTAAATCGTTCGATACGTCCGGTAGTGCCTCGTAATTCTGTTCCGGCGACGGTT

CGGGGTTAAGGGGTGTTACATTTTATGGTATCAGAGCTATCAGGTTTAGCCGATTCTCGG

CCTAAATCGAGCTCGGAATTGAGTCTAGATGTACATGCCACTGTCGAGTTAAACTGAGTC

GGGATTTTTGGATGCTGACCTATTTGTTTGTTTTGTTTTATAGATTAAAGATGTCTGAAG

AAAGAATAAATGATACTGATGAAAGAATGTATAGTGAAGATAGAGAATTAGATGAAACAG

AATCTGTTGCACCGAGTGTGAATCCGTTAGGCAACCAACCTTCTAATGTAGAACGAGAAA

ATGTCAGAGATAGAGATGAATCCCAATTACTGAGAATTATAGCTGATGCATTACAAAGAG

TAGCAGGAACTACTCCTGTTACGACTTCAGTACCTACTGTTAGACGGGCTCCGATAAAGG

AACTGAGGAAATATGGTGCCACTGAATTTATGGGTCTAAAAGGAGTTGATCCATCCATAG

CTGAAAATTGGATGGAGTCGACTAAAAGAATTTTGCAGCAATTGGATTGTACCCCCCGAG

AGTGTTTAATCTGTGCCGTATCGTTATTACAAGGGGAGGCTTATCTATGGTGGGAATCAG

TGGTTCGACATTTACCAGAGAGTCAGATAACGTGGGATCTATTTCAGAAGGAGTTTCAAA

AGAAATATATCGGAGAGATGTATATTGAAGACAAGAAACAAGAGTTTTTGTTGCTACAAC

AGGGTGATATGTCAGTAATAGATTATGAGAGGGAATTCTCGAGACTCAGTAGATATGCCT

CCGAGTTTATTCCGACAGAAGCCGATAGTTGTAAAAGATTTTTACGGGGTTTACGAGACG

AGATCAAAGTGCAGCTAGTATCCCATCGGATCACTGAGTTAGTAGATTTGATTGAACGAG

CTAAAATGGTGGAACAAGTTCTGGGCCTCGACAAAAAGACTGAAGTTGTTAGACCAACCG

GGAAGCGTACAGGAACTACCAGTTCGAATCCTCAGCCGAAAAGACCAAAGGAATTCCAAA

GTGGTTGGAGATCCAGTTTCAGGTCAGACAGAGGTGGTAGAAATAGGGGAAAACAGACGA

TGACATCTACTGGCAGTGTGAAAGGTCCTTCCCGAGAAATAGATATTCCAGACTGCCAAC

ACTGCGGAAAGAAACACAGAGGGGAATGTTGGAAATTAACTAGAGGCTGTTTTCGATGTG

GTTCTACAGACCATTTCATCAGAGACTGTCCGAAAGTTGATAGTACTGTACCCGTGACAT

CACAGAGATCGGTATCTACAGCTAGAGGCAGAGGGTTAGGAAGAGGTGGTTCGGTTTCAA

GGGGAGGAAGTATTAGGAGAAGCAATGATATTGCTACTCAGCAGTCTGAGGCTAAAGTAC

CTGCCAGAGCTTATGTGGTCAGAACACAGGAAGAAGGTGACGCCCACGATGTAGTAACAG

GTATATTCTTACTATATTCTGAGCCTGTTTATGCTTTAATTGATCCCGGATCTTCACATT

CTTATATAAATTCAAAATTAGTTGAATTGGGAAAATTTAATTCTGAAATATCTAGAGTGA

CTGTAGAAGTGTCGAGTCCGTTGGGGCAAACAGTATTAGTGAATCAGATCTGTCCGAGAT

GCCCGTTAATTATACAAAATAAAACTTTTCCTATTGACCTGTTGATTATGCCATTTGGAG

ATTTTGATATAATACTGGGGATGGATTGGTTGGCTGAGCACGGAGTGGTATTGGATTGTT

ATAAAAAGAAGTTTAGTATTCAGACAGAAGACGGGGACAGAATTGAAGTAAATGGTATCC

GTACTAATGGGCCGACACGTATTATTTCGGCAATAAAGGCTAATAAATTGCTTCAGCGGG

GTTGTACAGCGTATTTAGCCTATGTTATTAATTCTGATTTGGTTGGTAGTCAGTGCAGTA

AGATTAGAACCGTATGTGAGTTTCCAGATGTATTTCCTGAAGAGCTACCGGGTTTACCAC

CTGACAGAGAGGTTGAATTTGCTATAGAAGTGTATCCGGGTACAGCACCAATCTCTATAC

CACCGTATCGAATGTCACCCACTGAGTTGAAAGAGTTGAAAGTGCAGTTACAGGACTTGT

CAGATCGTGGATTTATTAGACCGAGCATCTCACCTTGGGGAGCTCCAGTATTGTTTGTTA

AAAAGAAAGATGGATCGATGCGGCTTTGTATTGATTACCGGCAGTTAAACAAAGTGACGA

TCAAGAACCGGTATCCGTTACCCCGTATAGATGATTTATTTGATCAACTAAAAGGAGCTT

CAGTATTTTCAAAGATTGACTTAAGATCTGGGTATTATCAGCTGAAGGTAAAAGAAAGTG

ATGTTCCGAAGACTGCATTTCGTACTCGATATGGTCATTATGAATTTTTGGTGATGCCGT

TCGGGTTGACTAATGCTCCAGCTGCTTTTATGGATCTGATGAATCGTATTTTTCAGCCGT

ATTTAGATCAGTTTGTGGTGGTTTTTATTGATGACATCTTGGTTTATTCGAAGTCAGAGT

CAGAGCATGATCAGCATCTCAGAACCGTGCTACAAATTCTGCGAGAAAAACAGTTGTACG

GGAAACTAAGTAAATGTGAATTCTGGTTATCAGAGGTAGTATTCTTGGGACATGTTGTAT

CTGCGGATGGGATTAGAGTTGATCCGAAGAAGATCGAGGCAATTGTTCAATGGAAGGCAC

CAAAGAATGTATCAGAGGTACGCAGTTTTCTTGGTTTGGCTGGGTATTACAGAAGATTTG

TAAATGGGTTTTCGAAGATAGCTTTGCCGATGACCAAATTACTACAGAAGAATGTTCCAT

TTATCTGGGATGATCAGTGTCAGAGGAGCTTTGAAACATTGAAACAGATGTTGACAGAGG

CACCAGTTTTAACTTTACCAGAATCAGGGAAAGATTTCATAGTGTACAGTGATGCTTCTT

TGAATGGTTTGGGTTGTGTATTGATGCAAGAAGGAAAAGTAATAGCTTATGCATCTCGAC

AGTTGAAGTCACATGAACGCAACTACCCGACACACGATTTAGAGTTAGCTGCTGTAATCT

TTGCATTGAAGATTTGGATACATTACTTGTATGGTGAGAAATGTTATATTTACACTGATC

ATAAAAGTCTAAAATATCTTCTGTCACAAAAGGAGTTGAATCTGAGACAGAGACGGTGGA

TTGAACTTCTGAAAGATTATGATTGTGTTATAGATTATCATCCAGGGAAGGCAAATGTGG

TAGCAGATGCATTGAGTAGAAAAGCAGCGATTGAATTACGAGCAATGTTCGCTCGACTTA

GTATTAAGGATGATGGAAGTTTGTTAGCTGAGTTAAGAGTCAAGCCGGTGATGTTTGATC

AAATCAGAGCAGCACAGTTAAAAGATGAAAAGTTGATGAGGAAAAGAGAAATGGTACAGT

ATGGTGCGGTAGAAAATTTTAGTATTGACGAGCATGATTGTTTGAGATTTCGAAATCGAA

TTTGTGTTCCATCTACTTCTGAGATTAAAGAATTGATTCTCCGAGAAGCACATAATAGTA

TTTTTGCTTTGCACCCAGGAGGAACGAAGATGTATCGTGATCTACGAGAACTGTATTGGT

GGCCAGGAATGAAGAAAGATATAGTTGAATATGTCAGTAAATGCTTGACTTGTCAGCGGG

TAAAAGCAGAACATCAGGTACCAACAGGCCTGTTACAGCCTATTACTATTCCCGAGTGGA

AATGGGATCGCATTACCATGGATTTTGTTACGGGGTTGCCATTGTCAGTGAGTAAAAAGA

ATGCTATTTGGGTGATTGTTGATCGACTCACAAAATCAGCTCATTTTATAGCAGTTAGAA

CCGACTGGTCATTACAGAAGCTTGCCGAGGTTTATATTCGAGAAATTGTTAGATTACATG

GTATTCCGGTATCAATAATTTCAGACAGAGATCCTCGATTCACTTCGAGATTTTGGAAGC

AGCTGCATGAATCATTGGGTACTCGACTTAGTTTCAGTACAGCTTTTCATCCTCAAACTG

ATGGACAATCTGAACGAGTAATTCAGATATTAGAAGATATGCTTCGAGCTTGTGTCATTG

ATTTTGAATCAGGTTGGGAACGTTATTTACCATTGGCCGAGTTTGTTTATAATAATAGTT

TCCAATCTAGTATTCAAATGGCTCCATATGAAGCACTTTATGGTCGAAGGTGTCGATCAC

CAATATGTTGGACAAAATTAAGAGAAAGAAAAGTGATTGGGCCGGAATTGATTCAAGAGA

CAGAAGAAACAGTTAAAAAGATTAAAGATAGACTGAAAGCCGCTTTCGACAGACAGAAAT

CTTACGCAGACTTGAAACGACGAGACATTGAATATTCCGTTGGTGATAAGGTATTCCTCA

AAGTATCGCCGTGGAAGAAAATTTTGAGATTTGGTCGGAAGGGAAAATTAAGTCCGCGCT

TTATTGGGCCGTATGAGATAGTGGAAAGAATTGGGCCTGTTGCTTATCGATTATCCTTAC

CTCCAGAGTTACAGAAAATTCATGATGTTTTTCATGTTTCGATGCTTCGGAGATATAGAT

CGGATCCTTCTCATGTTATTCCCACTGAAGACATTGAACTTCGATCTGATTTAACTTATG

AAGAAGAACCAGTTCAAATATTAGCACGAGAAGTGAAAGAATTAAGAAATAAACGGGTTC

CTTTAGTACAAGTTTTATGGAGAAGCCATAGTGTGGAAGAAGCAACTTGGGAACCGGAAG

AGACAATGAGAGCACAATATCCTCATCTCTTCTCAGGTAAATTTCGAGGACGAAATTTAT

TAAGAGGGGGAGAAATGTAATGACCTAAAATTCATGGGCATCGGAAAAGTATAATATTGG

GCCTCCGTCCTAGTAAATTGAGTCCGAAAATAATTATTAGAAATATTTACGAGACTAGTA

GTGTGTTTAATTAGGTTTTAATTAAGTAAATTTAGCTTAATTTAGAGTAATTAGTAAAAA

GGATTAAATTGAATAAGAGTAAAAGTTTAATTATAGATTAAAGGAAAATAATAGGGACCA

AATGGGCAATTAAGCCACATTTGGAAGTTGAGGCGGCATAACATTGTAAAAATCTTAGAT

TTTTATATTATTATTTATATAAATATATAAATTAATTATAAAGTATATTATTAAATTAAT

TATATTATAAATATTATATTATTATATATAAAAGAAACAAAACAGAAAAGAAACAGAATA

GAAAGAACAAAGAAACAGAATAGAAGAGACGAAACAGGGGAGAAGCAGGGGAGAAAGAAG

AAAAAGAAGAAAAAAGGGGAAATAGGGTTTTTGAAGCTTGAAATTTAAATTGGTAAGTCA

AATTAGCCATTTTCTCTTAATTCTAATGTTTTAAAAGCTTTAAAACAAAGTTTTGATGGA

ATTAAGTTGATATTTTGTAAGTTCATAGGTTTTCAAGTATAGTTTATGTTGAACAAAAGA

GATGAATTAGGGATTAACTTGAAGGAATTTTAAGTTAGAATTGAAAAAGGGATTAAATTG

TAAAAGAAACTATAAGTTTTTTTTGTTTTAGGGACTAGATTGAGGAAAATTCGGAATTAA

GAAAATATGTTAAAAATTTAATAGTTAAATTTGAGTTTAAATGAAATTTGAATAGGAATA

AGGTGTGAATTGGTGTTATAAATTTGGTTATTAACATTTTTAATCAAAACAGTTTTGGGA

AGTAGCAATGGTCTGACTTTGAAAATTCACTAAAAATTTTATAAATTGAACTAGAGGATG

AACAAAATATGGAATTAAAGCTTATTGAGTCTAGTTTCTTATAGTAGAAACAATGTAAGC

AATTAATTGATGAATCAAGAGATATTTGAAATTTTGTAATACTGGTTCGGGGTGATTTCG

AGATGCCCTGTTTTAACTTTGGAAAATCATTAAAAATTGTACAAAAATTATTATGGAGTG

TAATTTATATATGTAAACTCCTTAATGAATCTAGTTTCAAAATAAATAAACAAGAACCTT

ATTCGAGTTCTGTACAATGAGATAATTTAGTTTTAGTGGAGAGAGGTCAGAACTGTCAAA

TGAAATAACAGGGGAGTATTTAACGAATAAACTGTATTAAATGGCTAGACCAAAAATTCT

GGAAATTTTATGATTAGAAGATATATGAGTCTAGTTTTAAGGAAAATTTACGGATATTAA

TTTGGAGTTTCGTAGCTCAAGATATAAATAATTTAGTAACAATGACCCAAGTAGACAGCT

TAATGGTGAAATTATATAAATACATTAAAAATGGTTAAATTTGCATGTTTAGGCTCATGA

ATTAAATTGAATCATGTTGTATTGATTATTATAAATTATTATTTTCGTAGCCAACAAAGA

ACCTAAAGCATCAGCATCGAAAGGAAAGGAGAAAGTCATCGAGGAGTAAACTCGAGAAAA

TTACGGTTTGTATTACTATAATTCAAGTTATTTATTATTAAATGTTAAATTTTAATTTAT

GTGTCTAGTAAATGAAATGTGAGGTAAGTATTATTATTATTATTATTATTATTATTATTA

TTATTATTATTATTATTATGAGTGGGAATTAAATTGAATAGTTGATATGAAATAATATTT

GAATTGTTTGTTGATTGAAAGCGGGAAATGAATTTAAATCGAATAGTGACCGATATTAAA

TTGAATGGAAATGTATTGAGTTGTGAAAATATGTTAATTGCGGATTAATTATTGATTGAA

AGGTGGAAAAATGATTGAATTGAAAGTGTGAGAAAGTGTGATTGAATTGGGATTATATGT

GATTTAAATACCCTATTAACTAGTCGGGCTGAGTCGGATATAGTTGGCATGCCATAGGAT

TGGAAGAGTTCAGGGATACTTCGACCTCGAGTCGATGAGACACTGGGTGTCACTATATTT

CTTCGGATAGATTCGATGAGGTACTGGGTACCAACTTTCTTCGGCTTTGCCGATGAGACA

CTGGGTGTCAACTATTGCTTCGAACTATCCGATGAGGCACTGGGTGCCATTCTGGTGTGT

TTGGTTGGATCCGTGTATTCGCCAAAGTCCGAGTTTTGTTAATAGGGTAAATGATGAAAT

GATAAACCGAACGAGTTGGTCAAACGAGCTATTGAAATGATATGAAAAAGTTGAATTGTG

AATTGAAATGTGAAATGAGATTGAGAAATGAACCTAAGGTTCGTGAATTATTCAAACTCA

AATTGTGGATATACGATATTGGTTGATGAATTGCTATTGTTGAAATATTTAATTTAAATT

GTATATACGATTTATGCTTTACATGTACATTATTGTTATAATTTGAATTATGGTAATACC

ACTGAGTATGAATTACTCAGCGTACGGTTGTTTCCGTGCGCAGGTCAATAGAAGTCAAAG

GTCTCGGTTCAGCATCCAGATTAATCCCGGCTTCGGCAAAACTTGGTGATGTATTTTTCC

TTTGGTAAAGGTGGCATGTACATAGATTGTGTATAAAGGTTATTATGTTTTATTATATAA

TGGTTAAAAATGTTAGTATTAAAAGTTTATGGATTTTAATGAAAGAAGTCTATCTATTTT

ATCTAATTAGTACATTGTTAAATTTTAAATTGGTATTAGATTGAGTTTGATTAGAAGTAT

TTAGAATAGAAAATGTGAATGTGAAATGAATTGGTTGAATTGATGATATTTGGGAACTAT

ATGGTTTTAATTTGC

>Coker312

ATGTAATGACCCAAAATTCATGGGCATCGGAAAAGTATAATATCGGGCCTCCGTCCTAGT

AAATTGAGTCCGAAAATAATTATTAGAAATATTTACGAGACTAGTAGTGTGTTTAATTAG

GTTTTAATTAAGTAAATTTAGCTTAATTTAGAGTAATTAGTAAAAAGGATTAAATTGAAT

AAGAGTAAAAGTTTAATTATAGATTAAAGGAAAATAATAGGGACCAAATGGGCAATTAAG

CCACATTTGGAAGTTGAGGCGGCATAACATTGTAAAAATCTTAGATTTTTATATTATTAT

TTATATAAATATATAAATTAATTATAAAGTATATTATTAAATTAATTATATTATAAATAT

TATATTATTATATATAAAAGAAACAAAACAGAAAAGAAACAGAATAGAAAGAACAAAGAA

ACAGAATAGAAGAGACGAAACAGGGGAGAAGCAGGGGAGAAAGAAGAAAAAGAAGAAAAA

AGGGGAAATAGGGTTTTTGAAGCTTGAAATTTAAATTGGTAAGTCAAATTAGCCATTTTC

TCTTAATTCTAATGTTTTAAAAGCTTTAAAACAAAGTTTTGATGGAATTAAGTTGATATT

TTGTAAGTTCATAGGTTTTCAAGTATAGTTTATGTTGAACAAAAGAGATGAATTAGGGAT

TAACTTGAAGGAATTTTAAGTTAGAATTGAAAAAGGGATTAAATTGTAAAAGAAACTATA

AGTTTTTTTTGTTTTAGGGACTAGATTGAGGAAAATTCGGAATTAAGAAAATATGTTAAA

AATTTAATAGTTAAATTTGAGTTTAAATGAAATTTGAATAGGAATAAGGTGTGAATTGGT

GTTATAAATTTGGTTATTAACATTTTTAATCAAAACAGTTTTGGGAAGTAGCAATGGTCT

GACTTTGAAAATTCACTAAAAATTTTATAAATTGAACTAGAGGATGAACAAAATATGGAA

TTAAAGCTTATTGAGTCTAGTTTCTTATAGTAGAAACAATGTAAGCAATTAATTGATGAA

TCAAGAGATATTTGAAATTTTGTAATACTGGTTCGGGGTGATTTCGAGATGCCCTGTTTT

AACTTTGGAAAATCATTAAAAATTGTACAAAAATTATTATGGAGTGTAATTTATATATGT

GAACTCCTTAATGAATCTAGTTTCAAAATAAATAAACAAGAACCTTATTCGAGTTCTGTA

CAATGAGATAATTTAGTTTTAGTGGAGAGAGGTCAGAACTGTCAAATGAAATAACAGGGG

AGTATTTAACGAATAAACTGTATTAAATGGCTAGACCAAAAATTCTGGAAATTTTATGAT

TAGAAGATATATGAGTCTAGTTTTAAGGAAAATTTACGGATATTAATTTGGAGTTTCGTA

GCTCAAGATATAAATAATTTAGTAACAATGACCCAAGTAGACAGCTTAATGGTGAAATTA

TATAAATACATTAAAAATGGTTAAATTTGCATGTTTAGGCTCATGAATTAAATTGAATCA

TGTTGTATTGATTATTATAAATTATTATTTTCGTAGCCAACAAAGAACCTAAAGCATCAG

CATCGAAAGGAAAGGAGAAAGTCATCGAGGAGTAAACTCGAGAAAATTACGGTTTGTATT

ACTATAATTCAAGTTATTTATTATTAAATGTTAAATTTTAATTTATGTGTCTAGTAAATG

AAATGTGAGGTAAGTATTATTATTATTATTATTATTATTATTATTATTATTATTATTATT

ATGAGTGGGAATTAAATTGAATAGTTGATATGAAATAATATTTGAATTGTTTGTTGATTG

AAAGCGGGAAATGAATTTAAATCGAATAGTGACCGATATTAAATTGAATGGAAATGTATT

GAGTTGTGAAAATATGTTAATTGCGGATTAATTATTGATTGAAAGGTGGAAAAATGATTG

AATTGAAAGTGTGAGAAAGTGTGATTGAATTGGGATTATATGTGATTTAAATACCCTATT

AACTAGTCGGGCTGAGTCGGATATAGTTGGCATGCCATAGGATTGGAAGAGTTCAGGGAT

ACTTCGACCTCGAGTCGATGAGACACTGGGTGATTTCTTCGGATAGATTGGATGAGGTAC

TGGGTACCAACTTTCTTCGGCTTTGCCGATGAGACACTGGGTGTCAACTATTGCTTCGAA

CTATCCGATGAGGCACTGGGTGCCATTCTGGTGTGTTTGGTTGGATCCGTGTATCCGCCA

AAGTCCGAGTTTTGTTAATAGGGTAAATGATGAAATGATAAACCGAACGAGTTGGTCAAA

CGAGCTATTGAAATGATATGAAAAAGTTGAATTGTGAATTGAAATGTGAAATGAGATTGA

GAAATGAACCTAAGGTTCGTGAATTATTCAAACTCAAATTGTGGATATACGATATTGGTT

GATGAATTGCTATTGTTGAAATATTTAATTTAAATTGTATATACGATTTATGCTTTACAT

GTACATTATTGTTATAATTTGAATTATGGTAATACCACTGAGTATGAATTACTCAGCGTA

CGGTTGTTTCCGTGCGCAGGTCAATAGAAGTCAAAGGTCTCGGTTCAGCATCCAGATTAA

TCCCGGCTTCGGCAAAACTTGGTGATGTATTTTTCCTTTGGTAAAGGTGGCATGTACATA

GATTGTGTATAAAGGTTATTATGTTTTATTATATAATGGTTAAAAATGTTAGTATTAAAA

GTTTATGGATTTTAATGAAAGAAGTCTATCTATTTTATCTAATTAGTACATTGTTAAATT

TTAAATTGGTATTGTGTAGATTGAGTTTGATTAGAAGTATTTAGAATAGAAAATGTGAAT

GTGAAATGAATTGGTTGAATTGATGATATTTGGGAACTATATGGTTTTAATTTGCAGGGG

GTTTTATGTAAAAATAAGCAGAAATGCTGCCGAAATTTTTATAAAAAAAAATGAAGTCAT

TTGGTAAACAAATTAATAAATTTTATGAATTATTTTAATATATTGGTTATTTATTTAAGA

ATTGTTGTAAATCGTTCGATACGTCCGGTAGTGCCTCGTAATTCTGTTCCGGCGACGGTT

CGGGGTTAAGGGGTGTTACATTTTATGGTATCAGAGCTATCAGGTTTAGCCGATTCTCGG

CCTAAATCGAGCTCGGAATTGAGTCTAGATGTACATGCCACTGTCGAGTTAAACTGAGTC

GGGATTTTTGGATGCTGACCTATTTGTTTGTTTTGTTTTATAGATTAAAGATGTCTGAAG

AAAGAATAAATGATACTGATGAAAGAATGTATAGTGAAGATAGAGAATTAGATGAAACAG

AATCTGTTGCACCGAGTGTGAATCCGTTAGGCAACCAACCTTCTAATGTAGAACGAGAAA

ATGTCAGAGATAGAGATGAATCCCAATTACTGAGAATTATAGCTGATGCATTACAAAGAG

TAGCAGGAACTACTCCTGTTACGACTTCAGTACCTACTGTTAGACGGGCTCCGATAAAGG

AACTGAGGAAATATGGTGCCACTGAATTTATGGGTCTAAAAGGAGTTGATCCATCCATAG

CTGAAAATTGGATGGAGTCGACTAAAAGAATTTTGCAGCAATTGGATTGTACCCCCCGAG

AGTGTTTAATCTGTGCCGTATCGTTATTACAAGGGGAGGCTTATCTATGGTGGGAATCAG

TGGTTCGACATTTACCAGAGAGTCAGATAACGTGGGATCTATTTCAGAAGGAGTTTCAAA

AGAAATATATCGGAGAGATGTATATTGAAGACAAGAAACAAGAGTTTTTGTTGCTACAAC

AGGGTGATATGTCAGTAATAGATTATGAGAGGGAATTCTCGAGACTCAGTAGATATGCCT

CCGAGTTTATTCCGACAGAAGCCGATAGTTGTAAAAGATTTTTACGGGGTTTACGAGACG

AGATCAAAGTGCAGCTAGTATCCCATCGGATCACTGAGTTAGTAGATTTGATTGAACGAG

CTAAAATGGTGGAACAAGTTCTGGGCCTCGACAAAAAGACTGAAGTTGTTAGACCAACCG

GGAAGCGTACAGGAACTACCAGTTCGAATCCTCAGCCGAAAAGACCAAAGGAATTCCAAA

GTGGTTGGAGATCCAGTTTCAGGTCAGACAGAGGTGGTAGAAATAGGGGAAAACAGACGA

TGACATCTACTGGCAGTGTGAAAGGTCCTTCCCGAGAAATAGATATTCCAGACTGCCAAC

ACTGCGGAAAGAAACACAGAGGGGAATGTTGGAAATTAACTAGAGGCTGTTTTCGATGTG

GTTCTACAGACCATTTCATCAGAGACTGTCCGAAAGTTGATAGTACTGTACCCGTGACAT

CACAGAGATCGGTATCTACAGCTAGAGGCAGAGGGTTAGGAAGAGGTGGTTTGGTTTCAA

GGGGAGGAAGTATTAGGAGAAGCAATGATATTGCTACTCAGCAGTCTGAGGCTAAAGTAC

CTGCCAGAGCTTATGTGGTCAGAACACAGGAAGAAGGTGACGCCCACGATGTAGTAACAG

GTATATTCTTACTATATTCTGAGCCTGTTTATGCTTTAATTGATCCCGGATCTTCACATT

CTTATATAAATTCAAAATTAGTTGAATTGGGAAAATTTAATTCTGAAATATCTAGAGTGA

CTGTAGAAGTGTCGAGTCCGTTGGGGCAAACAGTATTAGTGAATCAGATCTGTCCGAGAT

GCCCGTTAATTATACAAAATAAAACTTTTCCTATTGACCTGTTGATTATGCCATTTGGAG

ATTTTGATATAATACTGGGGATGGATTGGTTGGCTGAGCACGGAGTGGTATTGGATTGTT

ATAAAAAGAAGTTTAGTATTCAGACAGAAGACGGGGACAGAATTGAAGTAAATGGTATCC

GTACTAATGGGCCGACACGTATTATTTCGGCAATAAAGGCTAATAAATTGCTTCAGCGGG

GTTGTACAGCGTATTTAGCCTATGTTATTAATTCTGATTTGGTTGGTAGTCAGTGCAGTA

AGATTAGAACCGTATGTGAGTTTCCAGATGTATTTCCTGAAGAGCTACCGGGTTTACCAC

CTGACAGAGAGGTTGAATTTGCTATAGAAGTGTATCCGGGTACAGCACCAATCTCTATAC

CACCGTATCGAATGTCACCCACTGAGTTGAAAGAGTTGAAAGTGCAGTTACAGGACTTGT

CAGATCGTGGATTTATTAGACCGAGCATCTCACCTTGGGGAGCTCCAGTATTGTTTGTTA

AAAAGAAAGATGGATCGATGCGGCTTTGTATTGATTACCGGCAGTTAAACAAAGTGACGA

TCAAGAACCGGTATCCGTTACCCCGTATAGATGATTTATTTGATCAACTAAAAGGAGCTT

CAGTATTTTCAAAGATTGACTTAAGATCTGGGTATTATCAGCTGAAGGTAAAAGAAAGTG

ATGTTCCGAAGACTGCATTTCGTACTCGATATGGTCATTATGAATTTTTGGTGATGCCGT

TCGGGTTGACTAATGCTCCAGCTGCTTTTATGGATCTGATGAATCGTATTTTTCAGCCGT

ATTTAGATCAGTTTGTGGTGGTTTTTATTGATGACATCTTGGTTTATTCGAAGTCAGAGT

CAGAGCATGATCAGCATCTCAGAACCGTGCTACAAATTCTGCGAGAAAAACAGTTGTACG

GGAAACTAAGTAAATGTGAATTCTGGTTATCAGAGGTAGTATTCTTGGGACATGTTGTAT

CTGCGGATGGGATTAGAGTTGATCCGAAGAAGATCGAGGCAATTGTTCAATGGAAGGCAC

CAAAGAATGTATCAGAGGTACGCAGTTTTCTTGGTTTGGCTGGGTATTACAGAAGATTTG

TAAATGGGTTTTCGAAGATAGCTTTGCCGATGACCAAATTACTACAGAAGAATGTTCCAT

TTATCTGGGATGATCAGTGTCAGAGGAGCTTTGAAACATTGAAACAGATGTTGACAGAGG

CACCAGTTTTAACTTTACCAGAATCAGGGAAAGATTTCATAGTGTACAGTGATGCTTCTT

TGAATGGTTTGGGTTGTGTATTGATGCAAGAAGGAAAAGTAATAGCTTATGCATCTCGAC

AGTTGAAGTCACATGAACGCAACTACCCGACACACGATTTAGAGTTAGCTGCTGTAATCT

TTGCATTGAAGATTTGGATACATTACTTGTATGGTGAGAAATGTTATATTTACACTGATC

ATAAAAGTCTAAAATATCTTCTGTCACAAAAGGAGTTGAATCTGAGACAGAGACGGTGGA

TTGAACTTCTGAAAGATTATGATTGTGTTATAGATTATCATCCAGGGAAGGCAAATGTGG

TAGCAGATGCATTGAGTAGAAAAGCAGCGATTGAATTACGAGCAATGTTCGCTCGACTTA

GTATTAAGGATGATGGAAGTTTGTTAGCTGAGTTAAGAGTCAAGCCGGTGATGTTTGATC

AAATCAGAGCAGCACAGTTAAAAGATGAAAAGTTGATGAGGAAAAGAGAAATGGTACAGT

ATGGTGCGGTAGAAAATTTTAGTATTGACGAGCATGATTGTTTGAGATTTCGAAATCGAA

TTTGTGTTCCATCTACTTCTGAGATTAAAGAATTGATTCTCCGAGAAGCACATAATAGTA

TTTTTGCTTTGCACCCAGGAGGAACGAAGATGTATCGTGATCTACGAGAACTGTATTGGT

GGCCAGGAATGAAGAAAGATATAGTTGAATATGTCAGTAAATGCTTGACTTGTCAGCGGG

TAAAAGCAGAACATCAGGTACCAACAGGCCTGTTACAGCCTATTACTATTCCCGAGTGGA

AATGGGATCGCATTACCATGGATTTTGTTACGGGGTTGCCATTGTCAGTGAGTAAAAAGA

ATGCTATTTGGGTGATTGTTGATCGACTCACAAAATCAGCTCATTTTATAGCAGTTAGAA

CCGACTGGTCATTACAGAAGCTTGCCGAGGTTTATATTCGAGAAATTGTTAGATTACATG

GTATTCCGGTATCAATAATTTCAGACAGAGATCCTCGATTCACTTCGAGATTTTGGAAGC

AGCTGCATGAATCATTGGGTACTCGACTTAGTTTCAGTACAGCTTTTCATCCTCAAACTG

ATGGACAATCTGAACGAGTAATTCAGATATTAGAAGATATGCTTCGAGCTTGTGTCATTG

ATTTTGAATCAGGTTGGGAACGTTATTTACCATTGGCCGAGTTTGTTTATAATAATAGTT

TCCAATCTAGTATTCAAATGGCTCCATATGAAGCACTTTATGGTCGAAGGTGTCGATCAC

CAATATGTTGGACAAAATTAAGAGAAAGAAAAGTGATTGGGCCGGAATTGATTCAAGAGA

CAGAAGAAACAGTTAAAAAGATTAAAGATAGACTGAAAGCCGCTTTCGACAGACAGAAAT

CTTACGCAGACTTGAAACGACGAGACATTGAATATTCCGTTGGTGATAAGGTATTCCTCA

AAGTATCGCCGTGGAAGAAAATTTTGAGATTTGGTCGGAAGGGAAAATTAAGTCCGCGCT

TTATTGGGCCGTATGAGATAGTGGAAAGAATTGGGCCTGTTGCTTATCGATTATCCTTAC

CTCCAGAGTTACAGAAAATTCATGATGTTTTTCATGTTTCGATGCTTCGGAGATATAGAT

CGGATCCTTCTCATGTTATTCCCACTGAAGACATTGAACTTCGATCTGATTTAACTTATG

AAGAAGAACCAGTTCAAATATTAGCACGAGAAGTGAAAGAATTAAGAAATAAACGGGTTC

CTTTAGTACAAGTTTTATGGAGAAGCCATAGTGTGGAAGAAGCAACTTGGGAACCGGAAG

AGACAATGAGAGCACAATATCCTCATCTCTTCTCAGGTAAATTTCGAGGACGAAATTTAT

TAAGAGGGGGAGAAATGTAATGACCTAAAATTCATGGGCATCGGAAAAGTATAATATTGG

GCCTCCGTCCTAGTAAATTGAGTCCGAAAATAATTATTAGAAATATTTACGAGACTAGTA

GTGTGTTTAATTAGGTTTTAATTAAGTAAATTTAGCTTAATTTAGAGTAATTAGTAAAAA

GGATTAAATTGAATAAGAGTAAAAGTTTAATTATAGATTAAAGGAAAATAATAGGGACCA

AATGGGCAATTAAGCCACATTTGGAAGTTGAGGCGGCATAACATTGTAAAAATCTTAGAT

TTTTATATTATTATTTATATAAATATATAAATTAATTATAAAGTATATTATTAAATTAAT

TATATTATAAATATTATATTATTATATATAAAAGAAACAAAACAGAAAAGAAACAGAATA

GAAAGAACAAAGAAACAGAATAGAAGAGACGAAACAGGGGAGAAGCAGGGGAGAAAGAAG

AAAAAGAAGAAAAAAGGGGAAATAGGGTTTTTGAAGCTTGAAATTTAAATTGGTAAGTCA

AATTAGCCATTTTCTCTTAATTCTAATGTTTTAAAAGCTTTAAAACAAAGTTTTGATGGA

ATTAAGTTGATATTTTGTAAGTTCATAGGTTTTCAAGTATAGTTTATGTTGAACAAAAGA

GATGAATTAGGGATTAACTTGAAGGAATTTTAAGTTAGAATTGAAAAAGGGATTAAATTG

TAAAAGAAACTATAAGTTTTTTTTGTTTTAGGGACTAGATTGAGGAAAATTCGGAATTAA

GAAAATATGTTAAAAATTTAATAGTTAAATTTGAGTTTAAATGAAATTTGAATAGGAATA

AGGTGTGAATTGGTGTTATAAATTTGGTTATTAACATTTTTAATCAAAACAGTTTTGGGA

AGTAGCAATGGTCTGACTTTGAAAATTCACTAAAAATTTTATAAATTGAACTAGAGGATG

AACAAAATATGGAATTAAAGCTTATTGAGTCTAGTTTCTTATAGTAGAAACAATGTAAGC

AATTAATTGATGAATCAAGAGATATTTGAAATTTTGTAATACTGGTTCGGGGTGATTTCG

AGATGCCCTGTTTTAACTTTGGAAAATCATTAAAAATTGTACAAAAATTATTATGGAGTG

TAATTTATATATGTAAACTCCTTAATGAATCTAGTTTCAAAATAAATAAACAAGAACCTT

ATTCGAGTTCTGTACAATGAGATAATTTAGTTTTAGTGGAGAGAGGTCAGAACTGTCAAA

TGAAATAACAGGGGAGTATTTAACGAATAAACTGTATTAAATGGCTAGACCAAAAATTCT

GGAAATTTTATGATTAGAAGATATATGAGTCTAGTTTTAAGGAAAATTTACGGATATTAA

TTTGGAGTTTCGTAGCTCAAGATATAAATAATTTAGTAACAATGACCCAAGTAGACAGCT

TAATGGTGAAATTATATAAATACATTAAAAATGGTTAAATTTGCATGTTTAGGCTCATGA

ATTAAATTGAATCATGTTGTATTGATTATTATAAATTATTATTTTCGTAGCCAACAAAGA

ACCTAAAGCATCAGCATCGAAAGGAAAGGAGAAAGTCATCGAGGAGTAAACTCGAGAAAA

TTACGGTTTGTATTACTATAATTCAAGTTATTTATTATTAAATGTTAAATTTTAATTTAT

GTGTCTAGTAAATGAAATGTGAGGTAAGTATTATTATTATTATTATTATTATTATTATTA

TTATTATTATTATTATTATGAGTGGGAATTAAATTGAATAGTTGATATGAAATAATATTT

GAATTGTTTGTTGATTGAAAGCGGGAAATGAATTTAAATCGAATAGTGACCGATATTAAA

TTGAATGGAAATGTATTGAGTTGTGAAAATATGTTAATTGCGGATTAATTATTGATTGAA

AGGTGGAAAAATGATTGAATTGAAAGTGTGAGAAAGTGTGATTGAATTGGGATTATATGT

GATTTAAATACCCTATTAACTAGTCGGGCTGAGTCGGATATAGTTGGCATGCCATAGGAT

TGGAAGAGTTCAGGGATACTTCGACCTCGAGTCGATGAGACACTGGGTGTCACTATATTT

CTTCGGATAGATTCGATGAGGTACTGGGTACCAACTTTCTTCGGCTTTGCCGATGAGACA

CTGGGTGTCAACTATTGCTTCGAACTATCCGATGAGGCACTGGGTGCCATTCTGGTGTGT

TTGGTTGGATCCGTGTATTCGCCAAAGTCCGAGTTTTGTTAATAGGGTAAATGATGAAAT

GATAAACCGAACGAGTTGGTCAAACGAGCTATTGAAATGATATGAAAAAGTTGAATTGTG

AATTGAAATGTGAAATGAGATTGAGAAATGAACCTAAGGTTCGTGAATTATTCAAACTCA

AATTGTGGATATACGATATTGGTTGATGAATTGCTATTGTTGAAATATTTAATTTAAATT

GTATATACGATTTATGCTTTACATGTACATTATTGTTATAATTTGAATTATGGTAATACC

ACTGAGTATGAATTACTCAGCGTACGGTTGTTTCCGTGCGCAGGTCAATAGAAGTCAAAG

GTCTCGGTTCAGCATCCAGATTAATCCCGGCTTCGGCAAAACTTGGTGATGTATTTTTCC

TTTGGTAAAGGTGGCATGTACATAGATTGTGTATAAAGGTTATTATGTTTTATTATATAA

TGGTTAAAAATGTTAGTATTAAAAGTTTATGGATTTTAATGAAAGAAGTCTATCTATTTT

ATCTAATTAGTACATTGTTAAATTTTAAATTGGTATTAGATTGAGTTTGATTAGAAGTAT

TTAGAATAGAAAATGTGAATGTGAAATGAATTGGTTGAATTGATGATATTTGGGAACTAT

ATGGTTTTAATTTGC

>Coker315

ATGTAATGACCCAAAATTCATGGGCATCGGAAAAGTATAATATCGGGCCTCCGTCCTAGT

AAATTGAGTCCGAAAATAATTATTAGAAATATTTACGAGACTAGTAGTGTGTTTAATTAG

GTTTTAATTAAGTAAATTTAGCTTAATTTAGAGTAATTAGTAAAAAGGATTAAATTGAAT

AAGAGTAAAAGTTTAATTATAGATTAAAGGAAAATAATAGGGACCAAATGGGCAATTAAG

CCACATTTGGAAGTTGAGGCGGCATAACATTGTAAAAATCTTAGATTTTTATATTATTAT

TTATATAAATATATAAATTAATTATAAAGTATATTATTAAATTAATTATATTATAAATAT

TATATTATTATATATAAAAGAAACAAAACAGAAAAGAAACAGAATAGAAAGAACAAAGAA

ACAGAATAGAAGAGACGAAACAGGGGAGAAGCAGGGGAGAAAGAAGAAAAAGAAGAAAAA

AGGGGAAATAGGGTTTTTGAAGCTTGAAATTTAAATTGGTAAGTCAAATTAGCCATTTTC

TCTTAATTCTAATGTTTTAAAAGCTTTAAAACAAAGTTTTGATGGAATTAAGTTGATATT

TTGTAAGTTCATAGGTTTTCAAGTATAGTTTATGTTGAACAAAAGAGATGAATTAGGGAT

TAACTTGAAGGAATTTTAAGTTAGAATTGAAAAAGGGATTAAATTGTAAAAGAAACTATA

AGTTTTTTTTGTTTTAGGGACTAGATTGAGGAAAATTCGGAATTAAGAAAATATGTTAAA

AATTTAATAGTTAAATTTGAGTTTAAATGAAATTTGAATAGGAATAAGGTGTGAATTGGT

GTTATAAATTTGGTTATTAACATTTTTAATCAAAACAGTTTTGGGAAGTAGCAATGGTCT

GACTTTGAAAATTCACTAAAAATTTTATAAATTGAACTAGAGGATGAACAAAATATGGAA

TTAAAGCTTATTGAGTCTAGTTTCTTATAGTAGAAACAATGTAAGCAATTAATTGATGAA

TCAAGAGATATTTGAAATTTTGTAATACTGGTTCGGGGTGATTTCGAGATGCCCTGTTTT

AACTTTGGAAAATCATTAAAAATTGTACAAAAATTATTATGGAGTGTAATTTATATATGT

GAACTCCTTAATGAATCTAGTTTCAAAATAAATAAACAAGAACCTTATTCGAGTTCTGTA

CAATGAGATAATTTAGTTTTAGTGGAGAGAGGTCAGAACTGTCAAATGAAATAACAGGGG

AGTATTTAACGAATAAACTGTATTAAATGGCTAGACCAAAAATTCTGGAAATTTTATGAT

TAGAAGATATATGAGTCTAGTTTTAAGGAAAATTTACGGATATTAATTTGGAGTTTCGTA

GCTCAAGATATAAATAATTTAGTAACAATGACCCAAGTAGACAGCTTAATGGTGAAATTA

TATAAATACATTAAAAATGGTTAAATTTGCATGTTTAGGCTCATGAATTAAATTGAATCA

TGTTGTATTGATTATTATAAATTATTATTTTCGTAGCCAACAAAGAACCTAAAGCATCAG

CATCGAAAGGAAAGGAGAAAGTCATCGAGGAGTAAACTCGAGAAAATTACGGTTTGTATT

ACTATAATTCAAGTTATTTATTATTAAATGTTAAATTTTAATTTATGTGTCTAGTAAATG

AAATGTGAGGTAAGTATTATTATTATTATTATTATTATTATTATTATTATTATTATTATT

ATGAGTGGGAATTAAATTGAATAGTTGATATGAAATAATATTTGAATTGTTTGTTGATTG

AAAGCGGGAAATGAATTTAAATCGAATAGTGACCGATATTAAATTGAATGGAAATGTATT

GAGTTGTGAAAATATGTTAATTGCGGATTAATTATTGATTGAAAGGTGGAAAAATGATTG

AATTGAAAGTGTGAGAAAGTGTGATTGAATTGGGATTATATGTGATTTAAATACCCTATT

AACTAGTCGGGCTGAGTCGGATATAGTTGGCATGCCATAGGATTGGAAGAGTTCAGGGAT

ACTTCGACCTCGAGTCGATGAGACACTGGGTGATTTCTTCGGATAGATTGGATGAGGTAC

TGGGTACCAACTTTCTTCGGCTTTGCCGATGAGACACTGGGTGTCAACTATTGCTTCGAA

CTATCCGATGAGGCACTGGGTGCCATTCTGGTGTGTTTGGTTGGATCCGTGTATCCGCCA

AAGTCCGAGTTTTGTTAATAGGGTAAATGATGAAATGATAAACCGAACGAGTTGGTCAAA

CGAGCTATTGAAATGATATGAAAAAGTTGAATTGTGAATTGAAATGTGAAATGAGATTGA

GAAATGAACCTAAGGTTCGTGAATTATTCAAACTCAAATTGTGGATATACGATATTGGTT

GATGAATTGCTATTGTTGAAATATTTAATTTAAATTGTATATACGATTTATGCTTTACAT

GTACATTATTGTTATAATTTGAATTATGGTAATACCACTGAGTATGAATTACTCAGCGTA

CGGTTGTTTCCGTGCGCAGGTCAATAGAAGTCAAAGGTCTCGGTTCAGCATCCAGATTAA

TCCCGGCTTCGGCAAAACTTGGTGATGTATTTTTCCTTTGGTAAAGGTGGCATGTACATA

GATTGTGTATAAAGGTTATTATGTTTTATTATATAATGGTTAAAAATGTTAGTATTAAAA

GTTTATGGATTTTAATGAAAGAAGTCTATCTATTTTATCTAATTAGTACATTGTTAAATT

TTAAATTGGTATTGTGTAGATTGAGTTTGATTAGAAGTATTTAGAATAGAAAATGTGAAT

GTGAAATGAATTGGTTGAATTGATGATATTTGGGAACTATATGGTTTTAATTTGCAGGGG

GTTTTATGTAAAAATAAGCAGAAATGCTGCCGAAATTTTTATAAAAAAAAATGAAGTCAT

TTGGTAAACAAATTAATAAATTTTATGAATTATTTTAATATATTGGTTATTTATTTAAGA

ATTGTTGTAAATCGTTCGATACGTCCGGTAGTGCCTCGTAATTCTGTTCCGGCGACGGTT

CGGGGTTAAGGGGTGTTACATTTTATGGTATCAGAGCTATCAGGTTTAGCCGATTCTCGG

CCTAAATCGAGCTCGGAATTGAGTCTAGATGTACATGCCACTGTCGAGTTAAACTGAGTC

GGGATTTTTGGATGCTGACCTATTTGTTTGTTTTGTTTTATAGATTAAAGATGTCTGAAG

AAAGAATAAATGATACTGATGAAAGAATGTATAGTGAAGATAGAGAATTAGATGAAACAG

AATCTGTTGCACCGAGTGTGAATCCGTTAGGCAACCAACCTTCTAATGTAGAACGAGAAA

ATGTCAGAGATAGAGATGAATCCCAATTACTGAGAATTATAGCTGATGCATTACAAAGAG

TAGCAGGAACTACTCCTGTTACGACTTCAGTACCTACTGTTAGACGGGCTCCGATAAAGG

AACTGAGGAAATATGGTGCCACTGAATTTATGGGTCTAAAAGGAGTTGATCCATCCATAG

CTGAAAATTGGATGGAGTCGACTAAAAGAATTTTGCAGCAATTGGATTGTACCCCCCGAG

AGTGTTTAATCTGTGCCGTATCGTTATTACAAGGGGAGGCTTATCTATGGTGGGAATCAG

TGGTTCGACATTTACCAGAGAGTCAGATAACGTGGGATCTATTTCAGAAGGAGTTTCAAA

AGAAATATATCGGAGAGATGTATATTGAAGACAAGAAACAAGAGTTTTTGTTGCTACAAC

AGGGTGATATGTCAGTAATAGATTATGAGAGGGAATTCTCGAGACTCAGTAGATATGCCT

CCGAGTTTATTCCGACAGAAGCCGATAGTTGTAAAAGATTTTTACGGGGTTTACGAGACG

AGATCAAAGTGCAGCTAGTATCCCATCGGATCACTGAGTTAGTAGATTTGATTGAACGAG

CTAAAATGGTGGAACAAGTTCTGGGCCTCGACAAAAAGACTGAAGTTGTTAGACCAACCG

GGAAGCGTACAGGAACTACCAGTTCGAATCCTCAGCCGAAAAGACCAAAGGAATTCCAAA

GTGGTTGGAGATCCAGTTTCAGGTCAGACAGAGGTGGTAGAAATAGGGGAAAACAGACGA

TGACATCTACTGGCAGTGTGAAAGGTCCTTCCCGAGAAATAGATATTCCAGACTGCCAAC

ACTGCGGAAAGAAACACAGAGGGGAATGTTGGAAATTAACTAGAGGCTGTTTTCGATGTG

GTTCTACAGACCATTTCATCAGAGACTGTCCGAAAGTTGATAGTACTGTACCCGTGACAT

CACAGAGATCGGTATCTACAGCTAGAGGCAGAGGGTTAGGAAGAGGTGGTTCGGTTTCAA

GGGGAGGAAGTATTAGGAGAAGCAATGATATTGCTACTCAGCAGTCTGAGGCTAAAGTAC

CTGCCAGAGCTTATGTGGTCAGAACACAGGAAGAAGGTGACGCCCACGATGTAGTAACAG

GTATATTCTTACTATATTCTGAGCCTGTTTATGCTTTAATTGATCCCGGATCTTCACATT

CTTATATAAATTCAAAATTAGTTGAATTGGGAAAATTTAATTCTGAAATATCTAGAGTGA

CTGTAGAAGTGTCGAGTCCGTTGGGGCAAACAGTATTAGTGAATCAGATCTGTCCGAGAT

GCCCGTTAATTATACAAAATAAAACTTTTCCTATTGACCTGTTGATTATGCCATTTGGAG

ATTTTGATATAATACTGGGGATGGATTGGTTGGCTGAGCACGGAGTGGTATTGGATTGTT

ATAAAAAGAAGTTTAGTATTCAGACAGAAGACGGGGACAGAATTGAAGTAAATGGTATCC

GTACTAATGGGCCGACACGTATTATTTCGGCAATAAAGGCTAATAAATTGCTTCAGCGGG

GTTGTACAGCGTATTTAGCCTATGTTATTAATTCTGATTTGGTTGGTAGTCAGTGCAGTA

AGATTAGAACCGTATGTGAGTTTCCAGATGTATTTCCTGAAGAGCTACCGGGTTTACCAC

CTGACAGAGAGGTTGAATTTGCTATAGAAGTGTATCCGGGTACAGCACCAATCTCTATAC

CACCGTATCGAATGTCACCCACTGAGTTGAAAGAGTTGAAAGTGCAGTTACAGGACTTGT

CAGATCGTGGATTTATTAGACCGAGCATCTCACCTTGGGGAGCTCCAGTATTGTTTGTTA

AAAAGAAAGATGGATCGATGCGGCTTTGTATTGATTACCGGCAGTTAAACAAAGTGACGA

TCAAGAACCGGTATCCGTTACCCCGTATAGATGATTTATTTGATCAACTAAAAGGAGCTT

CAGTATTTTCAAAGATTGACTTAAGATCTGGGTATTATCAGCTGAAGGTAAAAGAAAGTG

ATGTTCCGAAGACTGCATTTCGTACTCGATATGGTCATTATGAATTTTTGGTGATGCCGT

TCGGGTTGACTAATGCTCCAGCTGCTTTTATGGATCTGATGAATCGTATTTTTCAGCCGT

ATTTAGATCAGTTTGTGGTGGTTTTTATTGATGACATCTTGGTTTATTCGAAGTCAGAGT

CAGAGCATGATCAGCATCTCAGAACCGTGCTACAAATTCTGCGAGAAAAACAGTTGTACG

GGAAACTAAGTAAATGTGAATTCTGGTTATCAGAGGTAGTATTCTTGGGACATGTTGTAT

CTGCGGATGGGATTAGAGTTGATCCGAAGAAGATCGAGGCAATTGTTCAATGGAAGGCAC

CAAAGAATGTATCAGAGGTACGCAGTTTTCTTGGTTTGGCTGGGTATTACAGAAGATTTG

TAAATGGGTTTTCGAAGATAGCTTTGCCGATGACCAAATTACTACAGAAGAATGTTCCAT

TTATCTGGGATGATCAGTGTCAGAGGAGCTTTGAAACATTGAAACAGATGTTGACAGAGG

CACCAGTTTTAACTTTACCAGAATCAGGGAAAGATTTCATAGTGTACAGTGATGCTTCTT

TGAATGGTTTGGGTTGTGTATTGATGCAAGAAGGAAAAGTAATAGCTTATGCATCTCGAC

AGTTGAAGTCACATGAACGCAACTACCCGACACACGATTTAGAGTTAGCTGCTGTAATCT

TTGCATTGAAGATTTGGATACATTACTTGTATGGTGAGAAATGTTATATTTACACTGATC

ATAAAAGTCTAAAATATCTTCTGTCACAAAAGGAGTTGAATCTGAGACAGAGACGGTGGA

TTGAACTTCTGAAAGATTATGATTGTGTTATAGATTATCATCCAGGGAAGGCAAATGTGG

TAGCAGATGCATTGAGTAGAAAAGCAGCGATTGAATTACGAGCAATGTTCGCTCGACTTA

GTATTAAGGATGATGGAAGTTTGTTAGCTGAGTTAAGAGTCAAGCCGGTGATGTTTGATC

AAATCAGAGCAGCACAGTTAAAAGATGAAAAGTTGATGAGGAAAAGAGAAATGGTACAGT

ATGGTGCGGTAGAAAATTTTAGTATTGACGAGCATGATTGTTTGAGATTTCGAAATCGAA

TTTGTGTTCCATCTACTTCTGAGATTAAAGAATTGATTCTCCGAGAAGCACATAATAGTA

TTTTTGCTTTGCACCCAGGAGGAACGAAGATGTATCGTGATCTACGAGAACTGTATTGGT

GGCCAGGAATGAAGAAAGATATAGTTGAATATGTCAGTAAATGCTTGACTTGTCAGCGGG

TAAAAGCAGAACATCAGGTACCAACAGGCCTGTTACAGCCTATTACTATTCCCGAGTGGA

AATGGGATCGCATTACCATGGATTTTGTTACGGGGTTGCCATTGTCAGTGAGTAAAAAGA

ATGCTATTTGGGTGATTGTTGATCGACTCACAAAATCAGCTCATTTTATAGCAGTTAGAA

CCGACTGGTCATTACAGAAGCTTGCCGAGGTTTATATTCGAGAAATTGTTAGATTACATG

GTATTCCGGTATCAATAATTTCAGACAGAGATCCTCGATTCACTTCGAGATTTTGGAAGC

AGCTGCATGAATCATTGGGTACTCGACTTAGTTTCAGTACAGCTTTTCATCCTCAAACTG

ATGGACAATCTGAACGAGTAATTCAGATATTAGAAGATATGCTTCGAGCTTGTGTCATTG

ATTTTGAATCAGGTTGGGAACGTTATTTACCATTGGCCGAGTTTGTTTATAATAATAGTT

TCCAATCTAGTATTCAAATGGCTCCATATGAAGCACTTTATGGTCGAAGGTGTCGATCAC

CAATATGTTGGACAAAATTAAGAGAAAGAAAAGTGATTGGGCCGGAATTGATTCAAGAGA

CAGAAGAAACAGTTAAAAAGATTAAAGATAGACTGAAAGCCGCTTTCGACAGACAGAAAT

CTTACGCAGACTTGAAACGACGAGACATTGAATATTCCGTTGGTGATAAGGTATTCCTCA

AAGTATCGCCGTGGAAGAAAATTTTGAGATTTGGTCGGAAGGGAAAATTAAGTCCGCGCT

TTATTGGGCCGTATGAGATAGTGGAAAGAATTGGGCCTGTTGCTTATCGATTATCCTTAC

CTCCAGAGTTACAGAAAATTCATGATGTTTTTCATGTTTCGATGCTTCGGAGATATAGAT

CGGATCCTTCTCATGTTATTCCCACTGAAGACATTGAACTTCGATCTGATTTAACTTATG

AAGAAGAACCAGTTCAAATATTAGCACGAGAAGTGAAAGAATTAAGAAATAAACGGGTTC

CTTTAGTACAAGTTTTATGGAGAAGCCATAGTGTGGAAGAAGCAACTTGGGAACCGGAAG

AGACAATGAGAGCACAATATCCTCATCTCTTCTCAGGTAAATTTCGAGGACGAAATTTAT

TAAGAGGGGGAGAAATGTAATGACCTAAAATTCATGGGCATCGGAAAAGTATAATATTGG

GCCTCCGTCCTAGTAAATTGAGTCCGAAAATAATTATTAGAAATATTTACGAGACTAGTA

GTGTGTTTAATTAGGTTTTAATTAAGTAAATTTAGCTTAATTTAGAGTAATTAGTAAAAA

GGATTAAATTGAATAAGAGTAAAAGTTTAATTATAGATTAAAGGAAAATAATAGGGACCA

AATGGGCAATTAAGCCACATTTGGAAGTTGAGGCGGCATAACATTGTAAAAATCTTAGAT

TTTTATATTATTATTTATATAAATATATAAATTAATTATAAAGTATATTATTAAATTAAT

TATATTATAAATATTATATTATTATATATAAAAGAAACAAAACAGAAAAGAAACAGAATA

GAAAGAACAAAGAAACAGAATAGAAGAGACGAAACAGGGGAGAAGCAGGGGAGAAAGAAG

AAAAAGAAGAAAAAAGGGGAAATAGGGTTTTTGAAGCTTGAAATTTAAATTGGTAAGTCA

AATTAGCCATTTTCTCTTAATTCTAATGTTTTAAAAGCTTTAAAACAAAGTTTTGATGGA

ATTAAGTTGATATTTTGTAAGTTCATAGGTTTTCAAGTATAGTTTATGTTGAACAAAAGA

GATGAATTAGGGATTAACTTGAAGGAATTTTAAGTTAGAATTGAAAAAGGGATTAAATTG

TAAAAGAAACTATAAGTTTTTTTTGTTTTAGGGACTAGATTGAGGAAAATTCGGAATTAA

GAAAATATGTTAAAAATTTAATAGTTAAATTTGAGTTTAAATGAAATTTGAATAGGAATA

AGGTGTGAATTGGTGTTATAAATTTGGTTATTAACATTTTTAATCAAAACAGTTTTGGGA

AGTAGCAATGGTCTGACTTTGAAAATTCACTAAAAATTTTATAAATTGAACTAGAGGATG

AACAAAATATGGAATTAAAGCTTATTGAGTCTAGTTTCTTATAGTAGAAACAATGTAAGC

AATTAATTGATGAATCAAGAGATATTTGAAATTTTGTAATACTGGTTCGGGGTGATTTCG

AGATGCCCTGTTTTAACTTTGGAAAATCATTAAAAATTGTACAAAAATTATTATGGAGTG

TAATTTATATATGTAAACTCCTTAATGAATCTAGTTTCAAAATAAATAAACAAGAACCTT

ATTCGAGTTCTGTACAATGAGATAATTTAGTTTTAGTGGAGAGAGGTCAGAACTGTCAAA

TGAAATAACAGGGGAGTATTTAACGAATAAACTGTATTAAATGGCTAGACCAAAAATTCT

GGAAATTTTATGATTAGAAGATATATGAGTCTAGTTTTAAGGAAAATTTACGGATATTAA

TTTGGAGTTTCGTAGCTCAAGATATAAATAATTTAGTAACAATGACCCAAGTAGACAGCT

TAATGGTGAAATTATATAAATACATTAAAAATGGTTAAATTTGCATGTTTAGGCTCATGA

ATTAAATTGAATCATGTTGTATTGATTATTATAAATTATTATTTTCGTAGCCAACAAAGA

ACCTAAAGCATCAGCATCGAAAGGAAAGGAGAAAGTCATCGAGGAGTAAACTCGAGAAAA

TTACGGTTTGTATTACTATAATTCAAGTTATTTATTATTAAATGTTAAATTTTAATTTAT

GTGTCTAGTAAATGAAATGTGAGGTAAGTATTATTATTATTATTATTATTATTATTATTA

TTATTATTATTATTATTATGAGTGGGAATTAAATTGAATAGTTGATATGAAATAATATTT

GAATTGTTTGTTGATTGAAAGCGGGAAATGAATTTAAATCGAATAGTGACCGATATTAAA

TTGAATGGAAATGTATTGAGTTGTGAAAATATGTTAATTGCGGATTAATTATTGATTGAA

AGGTGGAAAAATGATTGAATTGAAAGTGTGAGAAAGTGTGATTGAATTGGGATTATATGT

GATTTAAATACCCTATTAACTAGTCGGGCTGAGTCGGATATAGTTGGCATGCCATAGGAT

TGGAAGAGTTCAGGGATACTTCGACCTCGAGTCGATGAGACACTGGGTGTCACTATATTT

CTTCGGATAGATTCGATGAGGTACTGGGTACCAACTTTCTTCGGCTTTGCCGATGAGACA

CTGGGTGTCAACTATTGCTTCGAACTATCCGATGAGGCACTGGGTGCCATTCTGGTGTGT

TTGGTTGGATCCGTGTATTCGCCAAAGTCCGAGTTTTGTTAATAGGGTAAATGATGAAAT

GATAAACCGAACGAGTTGGTCAAACGAGCTATTGAAATGATATGAAAAAGTTGAATTGTG

AATTGAAATGTGAAATGAGATTGAGAAATGAACCTAAGGTTCGTGAATTATTCAAACTCA

AATTGTGGATATACGATATTGGTTGATGAATTGCTATTGTTGAAATATTTAATTTAAATT

GTATATACGATTTATGCTTTACATGTACATTATTGTTATAATTTGAATTATGGTAATACC

ACTGAGTATGAATTACTCAGCGTACGGTTGTTTCCGTGCGCAGGTCAATAGAAGTCAAAG

GTCTCGGTTCAGCATCCAGATTAATCCCGGCTTCGGCAAAACTTGGTGATGTATTTTTCC

TTTGGTAAAGGTGGCATGTACATAGATTGTGTATAAAGGTTATTATGTTTTATTATATAA

TGGTTAAAAATGTTAGTATTAAAAGTTTATGGATTTTAATGAAAGAAGTCTATCTATTTT

ATCTAATTAGTACATTGTTAAATTTTAAATTGGTATTAGATTGAGTTTGATTAGAAGTAT

TTAGAATAGAAAATGTGAATGTGAAATGAATTGGTTGAATTGATGATATTTGGGAACTAT

ATGGTTTTAATTTGC

>Cokers_Clevewilt3

AGGTAATGACCCAAAATTCATGGGCATCGGAAAAGTATAATATCGGGCCTCCGTCCTAGT

AAATTGAGTCCGAAAATAATTATTAGAAATATTTACGAGACTAGTAGTGTGTTTAATTAG

GTTTTAATTAAGTAAATTTAGCTTAATTTAGAGTAATTAGTAAAAAGGATTAAATTGAAT

AAGAGTAAAAGTTTAATTATAGATTAAAGGAAAATAATAGGGACCAAATGGGCAATTAAG

CCACATTTGGAAGTTGAGGCGGCATAACATTGTAAAAATCTTAGATTTTTATATTATTAT

TTATATAAATATATAAATTAATTATAAAGTATATTATTAAATTAATTATATTATAAATAT

TATATTATTATATATAAAAGAAACAAAACAGAAAAGAAACAGAATAGAAAGAACAAAGAA

ACAGAATAGAAGAGACGAAACAGGGGAGAAGCAGGGGAGAAAGAAGAAAAAGAAGAAAAA

AGGGGAAATAGGGTTTTTGAAGCTTGAAATTTAAATTGGTAAGTCAAATTAGCCATTTTC

TCTTAATTCTAATGTTTTAAAAGCTTTAAAACAAAGTTTTGATGGAATTAAGTTGATATT

TTGTAAGTTCATAGGTTTTCAAGTATAGTTTATGTTGAACAAAAGAGATGAATTAGGGAT

TAACTTGAAGGAATTTTAAGTTAGAATTGAAAAAGGGATTAAATTGTAAAAGAAACTATA

AGTTTTTTTTGTTTTAGGGACTAGATTGAGGAAAATTCGGAATTAAGAAAATATGTTAAA

AATTTAATAGTTAAATTTGAGTTTAAATGAAATTTGAATAGGAATAAGGTGTGAATTGGT

GTTATAAATTTGGTTATTAACATTTTTAATCAAAACAGTTTTGGGAAGTAGCAATGGTCT

GACTTTGAAAATTCACTAAAAATTTTATAAATTGAACTAGAGGATGAACAAAATATGGAA

TTAAAGCTTATTGAGTCTAGTTTCTTATAGTAGAAACAATGTAAGCAATTAATTGATGAA

TCAAGAGATATTTGAAATTTTGTAATACTGGTTCGGGGTGATTTCGAGATGCCCTGTTTT

AACTTTGGAAAATCATTAAAAATTGTACAAAAATTATTATGGAGTGTAATTTATATATGT

GAACTCCTTAATGAATCTAGTTTCAAAATAAATAAACAAGAACCTTATTCGAGTTCTGTA

CAATGAGATAATTTAGTTTTAGTGGAGAGAGGTCAGAACTGTCAAATGAAATAACAGGGG

AGTATTTAACGAATAAACTGTATTAAATGGCTAGACCAAAAATTCTGGAAATTTTATGAT

TAGAAGATATATGAGTCTAGTTTTAAGGAAAATTTACGGATATTAATTTGGAGTTTCGTA

GCTCAAGATATAAATAATTTAGTAACAATGACCCAAGTAGACAGCTTAATGGTGAAATTA

TATAAATACATTAAAAATGGTTAAATTTGCATGTTTAGGCTCATGAATTAAATTGAATCA

TGTTGTATTGATTATTATAAATTATTATTTTCGTAGCCAACAAAGAACCTAAAGCATCAG

CATCGAAAGGAAAGGAGAAAGTCATCGAGGAGTAAACTCGAGAAAATTACGGTTTGTATT

ACTATAATTCAAGTTATTTATTATTAAATGTTAAATTTTAATTTATGTGTCTAGTAAATG

AAATGTGAGGTAAGTATTATTATTATTATTATTATTATTATTATTATTATTATTATTATT

ATGAGTGGGAATTAAATTGAATAGTTGATATGAAATAATATTTGAATTGTTTGTTGATTG

AAAGCGGGAAATGAATTTAAATCGAATAGTGACCGATATTAAATTGAATGGAAATGTATT

GAGTTGTGAAAATATGTTAATTGCGGATTAATTATTGATTGAAAGGTGGAAAAATGATTG

AATTGAAAGTGTGAGAAAGTGTGATTGAATTGGGATTATATGTGATTTAAATACCCTATT

AACTAGTCGGGCTGAGTCGGATATAGTTGGCATGCCATAGGATTGGAAGAGTTCAGGGAT

ACTTCGACCTCGAGTCGATGAGACACTGGGTGATTTCTTCGGATAGATTGGATGAGGTAC

TGGGTACCAACTTTCTTCGGCTTTGCCGATGAGACACTGGGTGTCAACTATTGCTTCGAA

CTATCCGATGAGGCACTGGGTGCCATTCTGGTGTGTTTGGTTGGATCCGTGTATCCGCCA

AAGTCCGAGTTTTGTTAATAGGGTAAATGATGAAATGATAAACCGAACGAGTTGGTCAAA

CGAGCTATTGAAATGATATGAAAAAGTTGAATTGTGAATTGAAATGTGAAATGAGATTGA

GAAATGAACCTAAGGTTCGTGAATTATTCAAACTCAAATTGTGGATATACGATATTGGTT

GATGAATTGCTATTGTTGAAATATTTAATTTAAATTGTATATACGATTTATGCTTTACAT

GTACATTATTGTTATAATTTGAATTATGGTAATACCACTGAGTATGAATTACTCAGCGTA

CGGTTGTTTCCGTGCGCAGGTCAATAGAAGTCAAAGGTCTCGGTTCAGCATCCAGATTAA

TCCCGGCTTCGGCAAAACTTGGTGATGTATTTTTCCTTTGGTAAAGGTGGCATGTACATA

GATTGTGTATAAAGGTTATTATGTTTTATTATATAATGGTTAAAAATGTTAGTATTAAAA

GTTTATGGATTTTAATGAAAGAAGTCTATCTATTTTATCTAATTAGTACATTGTTAAATT

TTAAATTGGTATTGTGTAGATTGAGTTTGATTAGAAGTATTTAGAATAGAAAATGTGAAT

GTGAAATGAATTGGTTGAATTGATGATATTTGGGAACTATATGGTTTTAATTTGCAGGGG

GTTTTATGTAAAAATAAGCAGAAATGCTGCCGAAATTTTTATAAAAAAAAATGAAGTCAT

TTGGTAAACAAATTAATAAATTTTATGAATTATTTTAATATATTGGTTATTTATTTAAGA

ATTGTTGTAAATCGTTCGATACGTCCGGTAGTGCCTCGTAATTCTGTTCCGGCGACGGTT

CGGGGTTAAGGGGTGTTACATTTTATGGTATCAGAGCTATCAGGTTTAGCCGATTCTCGG

CCTAAATCGAGCTCGGAATTGAGTCTAGATGTACATGCCACTGTCGAGTTAAACTGAGTC

GGGATTTTTGGATGCTGACCTATTTGTTTGTTTTGTTTTATAGATTAAAGATGTCTGAAG

AAAGAATAAATGATACTGATGAAAGAATGTATAGTGAAGATAGAGAATTAGATGAAACAG

AATCTGTTGCACCGAGTGTGAATCCGTTAGGCAACCAACCTTCTAATGTAGAACGAGAAA

ATGTCAGAGATAGAGATGAATCCCAATTACTGAGAATTATAGCTGATGCATTACAAAGAG

TAGCAGGAACTACTCCTGTTACGACTTCAGTACCTACTGTTAGACGGGCTCCGATAAAGG

AACTGAGGAAATATGGTGCCACTGAATTTATGGGTCTAAAAGGAGTTGATCCATCCATAG

CTGAAAATTGGATGGAGTCGACTAAAAGAATTTTGCAGCAATTGGATTGTACCCCCCGAG

AGTGTTTAATCTGTGCCGTATCGTTATTACAAGGGGAGGCTTATCTATGGTGGGAATCAG

TGGTTCGACATTTACCAGAGAGTCAGATAACGTGGGATCTATTTCAGAAGGAGTTTCAAA

AGAAATATATCGGAGAGATGTATATTGAAGACAAGAAACAAGAGTTTTTGTTGCTACAAC

AGGGTGATATGTCAGTAATAGATTATGAGAGGGAATTCTCGAGACTCAGTAGATATGCCT

CCGAGTTTATTCCGACAGAAGCCGATAGTTGTAAAAGATTTTTACGGGGTTTACGAGACG

AGATCAAAGTGCAGCTAGTATCCCATCGGATCACTGAGTTAGTAGATTTGATTGAACGAG

CTAAAATGGTGGAACAAGTTCTGGGCCTCGACAAAAAGACTGAAGTTGTTAGACCAACCG

GGAAGCGTACAGGAACTACCAGTTCGAATCCTCAGCCGAAAAGACCAAAGGAATTCCAAA

GTGGTTGGAGATCCAGTTTCAGGTCAGACAGAGGTGGTAGAAATAGGGGAAAACAGACGA

TGACATCTACTGGCAGTGTGAAAGGTCCTTCCCGAGAAATAGATATTCCAGACTGCCAAC

ACTGCGGAAAGAAACACAGAGGGGAATGTTGGAAATTAACTAGAGGCTGTTTTCGATGTG

GTTCTACAGACCATTTCATCAGAGACTGTCCGAAAGTTGATAGTACTGTACCCGTGACAT

CACAGAGATCGGTATCTACAGCTAGAGGCAGAGGGTTAGGAAGAGGTGGTTCGGTTTCAA

GGGGAGGAAGTATTAGGAGAAGCAATGATATTGCTACTCAGCAGTCTGAGGCTAAAGTAC

CTGCCAGAGCTTATGTGGTCAGAACACAGGAAGAAGGTGACGCCCACGATGTAGTAACAG

GTATATTCTTACTATATTCTGAGCCTGTTTATGCTTTAATTGATCCCGGATCTTCACATT

CTTATATAAATTCAAAATTAGTTGAATTGGGAAAATTTAATTCTGAAATATCTAGAGTGA

CTGTAGAAGTGTCGAGTCCGTTGGGGCAAACAGTATTAGTGAATCAGATCTGTCCGAGAT

GCCCGTTAATTATACAAAATAAAACTTTTCCTATTGACCTGTTGATTATGCCATTTGGAG

ATTTTGATATAATACTGGGGATGGATTGGTTGGCTGAGCACGGAGTGGTATTGGATTGTT

ATAAAAAGAAGTTTAGTATTCAGACAGAAGACGGGGACAGAATTGAAGTAAATGGTATCC

GTACTAATGGGCCGACACGTATTATTTCGGCAATAAAGGCTAATAAATTGCTTCAGCGGG

GTTGTACAGCGTATTTAGCCTATGTTATTAATTCTGATTTGGTTGGTAGTCAGTGCAGTA

AGATTAGAACCGTATGTGAGTTTCCAGATGTATTTCCTGAAGAGCTACCGGGTTTACCAC

CTGACAGAGAGGTTGAATTTGCTATAGAAGTGTATCCGGGTACAGCACCAATCTCTATAC

CACCGTATCGAATGTCACCCACTGAGTTGAAAGAGTTGAAAGTGCAGTTACAGGACTTGT

CAGATCGTGGATTTATTAGACCGAGCATCTCACCTTGGGGAGCTCCAGTATTGTTTGTTA

AAAAGAAAGATGGATCGATGCGGCTTTGTATTGATTACCGGCAGTTAAACAAAGTGACGA

TCAAGAACCGGTATCCGTTACCCCGTATAGATGATTTATTTGATCAACTAAAAGGAGCTT

CAGTATTTTCAAAGATTGACTTAAGATCTGGGTATTATCAGCTGAAGGTAAAAGAAAGTG

ATGTTCCGAAGACTGCATTTCGTACTCGATATGGTCATTATGAATTTTTGGTGATGCCGT

TCGGGTTGACTAATGCTCCAGCTGCTTTTATGGATCTGATGAATCGTATTTTTCAGCCGT

ATTTAGATCAGTTTGTGGTGGTTTTTATTGATGACATCTTGGTTTATTCGAAGTCAGAGT

CAGAGCATGATCAGCATCTCAGAACCGTGCTACAAATTCTGCGAGAAAAACAGTTGTACG

GGAAACTAAGTAAATGTGAATTCTGGTTATCAGAGGTAGTATTCTTGGGACATGTTGTAT

CTGCGGATGGGATTAGAGTTGATCCGAAGAAGATCGAGGCAATTGTTCAATGGAAGGCAC

CAAAGAATGTATCAGAGGTACGCAGTTTTCTTGGTTTGGCTGGGTATTACAGAAGATTTG

TAAATGGGTTTTCGAAGATAGCTTTGCCGATGACCAAATTACTACAGAAGAATGTTCCAT

TTATCTGGGATGATCAGTGTCAGAGGAGCTTTGAAACATTGAAACAGATGTTGACAGAGG

CACCAGTTTTAACTTTACCAGAATCAGGGAAAGATTTCATAGTGTACAGTGATGCTTCTT

TGAATGGTTTGGGTTGTGTATTGATGCAAGAAGGAAAAGTAATAGCTTATGCATCTCGAC

AGTTGAAGTCACATGAACGCAACTACCCGACACACGATTTAGAGTTAGCTGCTGTAATCT

TTGCATTGAAGATTTGGATACATTACTTGTATGGTGAGAAATGTTATATTTACACTGATC

ATAAAAGTCTAAAATATCTTCTGTCACAAAAGGAGTTGAATCTGAGACAGAGACGGTGGA

TTGAACTTCTGAAAGATTATGATTGTGTTATAGATTATCATCCAGGGAAGGCAAATGTGG

TAGCAGATGCATTGAGTAGAAAAGCAGCGATTGAATTACGAGCAATGTTCGCTCGACTTA

GTATTAAGGATGATGGAAGTTTGTTAGCTGAGTTAAGAGTCAAGCCGGTGATGTTTGATC

AAATCAGAGCAGCACAGTTAAAAGATGAAAAGTTGATGAGGAAAAGAGAAATGGTACAGT

ATGGTGCGGTAGAAAATTTTAGTATTGACGAGCATGATTGTTTGAGATTTCGAAATCGAA

TTTGTGTTCCATCTACTTCTGAGATTAAAGAATTGATTCTCCGAGAAGCACATAATAGTA

TTTTTGCTTTGCACCCAGGAGGAACGAAGATGTATCGTGATCTACGAGAACTGTATTGGT

GGCCAGGAATGAAGAAAGATATAGTTGAATATGTCAGTAAATGCTTGACTTGTCAGCGGG

TAAAAGCAGAACATCAGGTACCAACAGGCCTGTTACAGCCTATTACTATTCCCGAGTGGA

AATGGGATCGCATTACCATGGATTTTGTTACGGGGTTGCCATTGTCAGTGAGTAAAAAGA

ATGCTATTTGGGTGATTGTTGATCGACTCACAAAATCAGCTCATTTTATAGCAGTTAGAA

CCGACTGGTCATTACAGAAGCTTGCCGAGGTTTATATTCGAGAAATTGTTAGATTACATG

GTATTCCGGTATCAATAATTTCAGACAGAGATCCTCGATTCACTTCGAGATTTTGGAAGC

AGCTGCATGAATCATTGGGTACTCGACTTAGTTTCAGTACAGCTTTTCATCCTCAAACTG

ATGGACAATCTGAACGAGTAATTCAGATATTAGAAGATATGCTTCGAGCTTGTGTCATTG

ATTTTGAATCAGGTTGGGAACGTTATTTACCATTGGCCGAGTTTGTTTATAATAATAGTT

TCCAATCTAGTATTCAAATGGCTCCATATGAAGCACTTTATGGTCGAAGGTGTCGATCAC

CAATATGTTGGACAAAATTAAGAGAAAGAAAAGTGATTGGGCCGGAATTGATTCAAGAGA

CAGAAGAAACAGTTAAAAAGATTAAAGATAGACTGAAAGCCGCTTTCGACAGACAGAAAT

CTTACGCAGACTTGAAACGACGAGACATTGAATATTCCGTTGGTGATAAGGTATTCCTCA

AAGTATCGCCGTGGAAGAAAATTTTGAGATTTGGTCGGAAGGGAAAATTAAGTCCGCGCT

TTATTGGGCCGTATGAGATAGTGGAAAGAATTGGGCCTGTTGCTTATCGATTATCCTTAC

CTCCAGAGTTACAGAAAATTCATGATGTTTTTCATGTTTCGATGCTTCGGAGATATAGAT

CGGATCCTTCTCATGTTATTCCCACTGAAGACATTGAACTTCGATCTGATTTAACTTATG

AAGAAGAACCAGTTCAAATATTAGCACGAGAAGTGAAAGAATTAAGAAATAAACGGGTTC

CTTTAGTACAAGTTTTATGGAGAAGCCATAGTGTGGAAGAAGCAACTTGGGAACCGGAAG

AGACAATGAGAGCACAATATCCTCATCTCTTCTCAGGTAAATTTCGAGGACGAAATTTAT

TAAGAGGGGGAGAAATGTAATGACCTAAAATTCATGGGCATCGGAAAAGTATAATATTGG

GCCTCCGTCCTAGTAAATTGAGTCCGAAAATAATTATTAGAAATATTTACGAGACTAGTA

GTGTGTTTAATTAGGTTTTAATTAAGTAAATTTAGCTTAATTTAGAGTAATTAGTAAAAA

GGATTAAATTGAATAAGAGTAAAAGTTTAATTATAGATTAAAGGAAAATAATAGGGACCA

AATGGGCAATTAAGCCACATTTGGAAGTTGAGGCGGCATAACATTGTAAAAATCTTAGAT

TTTTATATTATTATTTATATAAATATATAAATTAATTATAAAGTATATTATTAAATTAAT

TATATTATAAATATTATATTATTATATATAAAAGAAACAAAACAGAAAAGAAACAGAATA

GAAAGAACAAAGAAACAGAATAGAAGAGACGAAACAGGGGAGAAGCAGGGGAGAAAGAAG

AAAAAGAAGAAAAAAGGGGAAATAGGGTTTTTGAAGCTTGAAATTTAAATTGGTAAGTCA

AATTAGCCATTTTCTCTTAATTCTAATGTTTTAAAAGCTTTAAAACAAAGTTTTGATGGA

ATTAAGTTGATATTTTGTAAGTTCATAGGTTTTCAAGTATAGTTTATGTTGAACAAAAGA

GATGAATTAGGGATTAACTTGAAGGAATTTTAAGTTAGAATTGAAAAAGGGATTAAATTG

TAAAAGAAACTATAAGTTTTTTTTGTTTTAGGGACTAGATTGAGGAAAATTCGGAATTAA

GAAAATATGTTAAAAATTTAATAGTTAAATTTGAGTTTAAATGAAATTTGAATAGGAATA

AGGTGTGAATTGGTGTTATAAATTTGGTTATTAACATTTTTAATCAAAACAGTTTTGGGA

AGTAGCAATGGTCTGACTTTGAAAATTCACTAAAAATTTTATAAATTGAACTAGAGGATG

AACAAAATATGGAATTAAAGCTTATTGAGTCTAGTTTCTTATAGTAGAAACAATGTAAGC

AATTAATTGATGAATCAAGAGATATTTGAAATTTTGTAATACTGGTTCGGGGTGATTTCG

AGATGCCCTGTTTTAACTTTGGAAAATCATTAAAAATTGTACAAAAATTATTATGGAGTG

TAATTTATATATGTAAACTCCTTAATGAATCTAGTTTCAAAATAAATAAACAAGAACCTT

ATTCGAGTTCTGTACAATGAGATAATTTAGTTTTAGTGGAGAGAGGTCAGAACTGTCAAA

TGAAATAACAGGGGAGTATTTAACGAATAAACTGTATTAAATGGCTAGACCAAAAATTCT

GGAAATTTTATGATTAGAAGATATATGAGTCTAGTTTTAAGGAAAATTTACGGATATTAA

TTTGGAGTTTCGTAGCTCAAGATATAAATAATTTAGTAACAATGACCCAAGTAGACAGCT

TAATGGTGAAATTATATAAATACATTAAAAATGGTTAAATTTGCATGTTTAGGCTCATGA

ATTAAATTGAATCATGTTGTATTGATTATTATAAATTATTATTTTCGTAGCCAACAAAGA

ACCTAAAGCATCAGCATCGAAAGGAAAGGAGAAAGTCATCGAGGAGTAAACTCGAGAAAA

TTACGGTTTGTATTACTATAATTCAAGTTATTTATTATTAAATGTTAAATTTTAATTTAT

GTGTCTAGTAAATGAAATGTGAGGTAAGTATTATTATTATTATTATTATTATTATTATTA

TTATTATTATTATTATTATGAGTGGGAATTAAATTGAATAGTTGATATGAAATAATATTT

GAATTGTTTGTTGATTGAAAGCGGGAAATGAATTTAAATCGAATAGTGACCGATATTAAA

TTGAATGGAAATGTATTGAGTTGTGAAAATATGTTAATTGCGGATTAATTATTGATTGAA

AGGTGGAAAAATGATTGAATTGAAAGTGTGAGAAAGTGTGATTGAATTGGGATTATATGT

GATTTAAATACCCTATTAACTAGTCGGGCTGAGTCGGATATAGTTGGCATGCCATAGGAT

TGGAAGAGTTCAGGGATACTTCGACCTCGAGTCGATGAGACACTGGGTGTCACTATATTT

CTTCGGATAGATTCGATGAGGTACTGGGTACCAACTTTCTTCGGCTTTGCCGATGAGACA

CTGGGTGTCAACTATTGCTTCGAACTATCCGATGAGGCACTGGGTGCCATTCTGGTGTGT

TTGGTTGGATCCGTGTATTCGCCAAAGTCCGAGTTTTGTTAATAGGGTAAATGATGAAAT

GATAAACCGAACGAGTTGGTCAAACGAGCTATTGAAATGATATGAAAAAGTTGAATTGTG

AATTGAAATGTGAAATGAGATTGAGAAATGAACCTAAGGTTCGTGAATTATTCAAACTCA

AATTGTGGATATACGATATTGGTTGATGAATTGCTATTGTTGAAATATTTAATTTAAATT

GTATATACGATTTATGCTTTACATGTACATTATTGTTATAATTTGAATTATGGTAATACC

ACTGAGTATGAATTACTCAGCGTACGGTTGTTTCCGTGCGCAGGTCAATAGAAGTCAAAG

GTCTCGGTTCAGCATCCAGATTAATCCCGGCTTCGGCAAAACTTGGTGATGTATTTTTCC

TTTGGTAAAGGTGGCATGTACATAGATTGTGTATAAAGGTTATTATGTTTTATTATATAA

TGGTTAAAAATGTTAGTATTAAAAGTTTATGGATTTTAATGAAAGAAGTCTATCTATTTT

ATCTAATTAGTACATTGTTAAATTTTAAATTGGTATTAGATTGAGTTTGATTAGAAGTAT

TTAGAATAGAAAATGTGAATGTGAAATGAATTGGTTGAATTGATGATATTTGGGAACTAT

ATGGTTTTAATTTGC

>Del_Cerro

AGGTAATGACCCAAAATTCATGGGCATCGGAAAAGTATAATATCGGGCCTCCGTCCTAGT

AAATTGAGTCCGAAAATAATTATTAGAAATATTTACGAGACTAGTAGTGTGTTTAATTAG

GTTTTAATTAAGTAAATTTAGCTTAATTTAGAGTAATTAGTAAAAAGGATTAAATTGAAT

AAGAGTAAAAGTTTAATTATAGATTAAAGGAAAATAATAGGGACCAAATGGGCAATTAAG

CCACATTTGGAAGTTGAGGCGGCATAACATTGTAAAAATCTTAGATTTTTATATTATTAT

TTATATAAATATATAAATTAATTATAAAGTATATTATTAAATTAATTATATTATAAATAT

TATATTATTATATATAAAAGAAACAAAACAGAAAAGAAACAGAATAGAAAGAACAAAGAA

ACAGAATAGAAGAGACGAAACAGGGGAGAAGCAGGGGAGAAAGAAGAAAAAGAAGAAAAA

AGGGGAAATAGGGTTTTTGAAGCTTGAAATTTAAATTGGTAAGTCAAATTAGCCATTTTC

TCTTAATTCTAATGTTTTAAAAGCTTTAAAACAAAGTTTTGATGGAATTAAGTTGATATT

TTGTAAGTTCATAGGTTTTCAAGTATAGTTTATGTTGAACAAAAGAGATGAATTAGGGAT

TAACTTGAAGGAATTTTAAGTTAGAATTGAAAAAGGGATTAAATTGTAAAAGAAACTATA

AGTTTTTTTTGTTTTAGGGACTAGATTGAGGAAAATTCGGAATTAAGAAAATATGTTAAA

AATTTAATAGTTAAATTTGAGTTTAAATGAAATTTGAATAGGAATAAGGTGTGAATTGGT

GTTATAAATTTGGTTATTAACATTTTTAATCAAAACAGTTTTGGGAAGTAGCAATGGTCT

GACTTTGAAAATTCACTAAAAATTTTATAAATTGAACTAGAGGATGAACAAAATATGGAA

TTAAAGCTTATTGAGTCTAGTTTCTTATAGTAGAAACAATGTAAGCAATTAATTGATGAA

TCAAGAGATATTTGAAATTTTGTAATACTGGTTCGGGGTGATTTCGAGATGCCCTGTTTT

AACTTTGGAAAATCATTAAAAATTGTACAAAAATTATTATGGAGTGTAATTTATATATGT

GAACTCCTTAATGAATCTAGTTTCAAAATAAATAAACAAGAACCTTATTCGAGTTCTGTA

CAATGAGATAATTTAGTTTTAGTGGAGAGAGGTCAGAACTGTCAAATGAAATAACAGGGG

AGTATTTAACGAATAAACTGTATTAAATGGCTAGACCAAAAATTCTGGAAATTTTATGAT

TAGAAGATATATGAGTCTAGTTTTAAGGAAAATTTACGGATATTAATTTGGAGTTTCGTA

GCTCAAGATATAAATAATTTAGTAACAATGACCCAAGTAGACAGCTTAATGGTGAAATTA

TATAAATACATTAAAAATGGTTAAATTTGCATGTTTAGGCTCATGAATTAAATTGAATCA

TGTTGTATTGATTATTATAAATTATTATTTTCGTAGCCAACAAAGAACCTAAAGCATCAG

CATCGAAAGGAAAGGAGAAAGTCATCGAGGAGTAAACTCGAGAAAATTACGGTTTGTATT

ACTATAATTCAAGTTATTTATTATTAAATGTTAAATTTTAATTTATGTGTCTAGTAAATG

AAATGTGAGGTAAGTATTATTATTATTATTATTATTATTATTATTATTATTATTATTATT

ATGAGTGGGAATTAAATTGAATAGTTGATATGAAATAATATTTGAATTGTTTGTTGATTG

AAAGCGGGAAATGAATTTAAATCGAATAGTGACCGATATTAAATTGAATGGAAATGTATT

GAGTTGTGAAAATATGTTAATTGCGGATTAATTATTGATTGAAAGGTGGAAAAATGATTG

AATTGAAAGTGTGAGAAAGTGTGATTGAATTGGGATTATATGTGATTTAAATACCCTATT

AACTAGTCGGGCTGAGTCGGATATAGTTGGCATGCCATAGGATTGGAAGAGTTCAGGGAT

ACTTCGACCTCGAGTCGATGAGACACTGGGTGATTTCTTCGGATAGATTGGATGAGGTAC

TGGGTACCAACTTTCTTCGGCTTTGCCGATGAGACACTGGGTGTCAACTATTGCTTCGAA

CTATCCGATGAGGCACTGGGTGCCATTCTGGTGTGTTTGGTTGGATCCGTGTATCCGCCA

AAGTCCGAGTTTTGTTAATAGGGTAAATGATGAAATGATAAACCGAACGAGTTGGTCAAA

CGAGCTATTGAAATGATATGAAAAAGTTGAATTGTGAATTGAAATGTGAAATGAGATTGA

GAAATGAACCTAAGGTTCGTGAATTATTCAAACTCAAATTGTGGATATACGATATTGGTT

GATGAATTGCTATTGTTGAAATATTTAATTTAAATTGTATATACGATTTATGCTTTACAT

GTACATTATTGTTATAATTTGAATTATGGTAATACCACTGAGTATGAATTACTCAGCGTA

CGGTTGTTTCCGTGCGCAGGTCAATAGAAGTCAAAGGTCTCGGTTCAGCATCCAGATTAA

TCCCGGCTTCGGCAAAACTTGGTGATGTATTTTTCCTTTGGTAAAGGTGGCATGTACATA

GATTGTGTATAAAGGTTATTATGTTTTATTATATAATGGTTAAAAATGTTAGTATTAAAA

GTTTATGGATTTTAATGAAAGAAGTCTATCTATTTTATCTAATTAGTACATTGTTAAATT

TTAAATTGGTATTGTGTAGATTGAGTTTGATTAGAAGTATTTAGAATAGAAAATGTGAAT

GTGAAATGAATTGGTTGAATTGATGATATTTGGGAACTATATGGTTTTAATTTGCAGGGG

GTTTTATGTAAAAATAAGCAGAAATGCTGCCGAAATTTTTATAAAAAAAAATGAAGTCAT

TTGGTAAACAAATTAATAAATTTTATGAATTATTTTAATATATTGGTTATTTATTTAAGA

ATTGTTGTAAATCGTTCGATACGTCCGGTAGTGCCTCGTAATTCTGTTCCGGCGACGGTT

CGGGGTTAAGGGGTGTTACATTTTATGGTATCAGAGCTATCAGGTTTAGCCGATTCTCGG

CCTAAATCGAGCTCGGAATTGAGTCTAGATGTACATGCCACTGTCGAGTTAAACTGAGTC

GGGATTTTTGGATGCTGACCTATTTGTTTGTTTTGTTTTATAGATTAAAGATGTCTGAAG

AAAGAATAAATGATACTGATGAAAGAATGTATAGTGAAGATAGAGAATTAGATGAAACAG

AATCTGTTGCACCGAGTGTGAATCCGTTAGGCAACCAACCTTCTAATGTAGAACGAGAAA

ATGTCAGAGATAGAGATGAATCCCAATTACTGAGAATTATAGCTGATGCATTACAAAGAG

TAGCAGGAACTACTCCTGTTACGACTTCAGTACCTACTGTTAGACGGGCTCCGATAAAGG

AACTGAGGAAATATGGTGCCACTGAATTTATGGGTCTAAAAGGAGTTGATCCATCCATAG

CTGAAAATTGGATGGAGTCGACTAAAAGAATTTTGCAGCAATTGGATTGTACCCCCCGAG

AGTGTTTAATCTGTGCCGTATCGTTATTACAAGGGGAGGCTTATCTATGGTGGGAATCAG

TGGTTCGACATTTACCAGAGAGTCAGATAACGTGGGATCTATTTCAGAAGGAGTTTCAAA

AGAAATATATCGGAGAGATGTATATTGAAGACAAGAAACAAGAGTTTTTGTTGCTACAAC

AGGGTGATATGTCAGTAATAGATTATGAGAGGGAATTCTCGAGACTCAGTAGATATGCCT

CCGAGTTTATTCCGACAGAAGCCGATAGTTGTAAAAGATTTTTACGGGGTTTACGAGACG

AGATCAAAGTGCAGCTAGTATCCCATCGGATCACTGAGTTAGTAGATTTGATTGAACGAG

CTAAAATGGTGGAACAAGTTCTGGGCCTCGACAAAAAGACTGAAGTTGTTAGACCAACCG

GGAAGCGTACAGGAACTACCAGTTCGAATCCTCAGCCGAAAAGACCAAAGGAATTCCAAA

GTGGTTGGAGATCCAGTTTCAGGTCAGACAGAGGTGGTAGAAATAGGGGAAAACAGACGA

TGACATCTACTGGCAGTGTGAAAGGTCCTTCCCGAGAAATAGATATTCCAGACTGCCAAC

ACTGCGGAAAGAAACACAGAGGGGAATGTTGGAAATTAACTAGAGGCTGTTTTCGATGTG

GTTCTACAGACCATTTCATCAGAGACTGTCCGAAAGTTGATAGTACTGTACCCGTGACAT

CACAGAGATCGGTATCTACAGCTAGAGGCAGAGGGTTAGGAAGAGGTGGTTCGGTTTCAA

GGGGAGGAAGTATTAGGAGAAGCAATGATATTGCTACTCAGCAGTCTGAGGCTAAAGTAC

CTGCCAGAGCTTATGTGGTCAGAACACAGGAAGAAGGTGACGCCCACGATGTAGTAACAG

GTATATTCTTACTATATTCTGAGCCTGTTTATGCTTTAATTGATCCCGGATCTTCACATT

CTTATATAAATTCAAAATTAGTTGAATTGGGAAAATTTAATTCTGAAATATCTAGAGTGA

CTGTAGAAGTGTCGAGTCCGTTGGGGCAAACAGTATTAGTGAATCAGATCTGTCCGAGAT

GCCCGTTAATTATACAAAATAAAACTTTTCCTATTGACCTGTTGATTATGCCATTTGGAG

ATTTTGATATAATACTGGGGATGGATTGGTTGGCTGAGCACGGAGTGGTATTGGATTGTT

ATAAAAAGAAGTTTAGTATTCAGACAGAAGACGGGGACAGAATTGAAGTAAATGGTATCC

GTACTAATGGGCCGACACGTATTATTTCGGCAATAAAGGCTAATAAATTGCTTCAGCGGG

GTTGTACAGCGTATTTAGCCTATGTTATTAATTCTGATTTGGTTGGTAGTCAGTGCAGTA

AGATTAGAACCGTATGTGAGTTTCCAGATGTATTTCCTGAAGAGCTACCGGGTTTACCAC

CTGACAGAGAGGTTGAATTTGCTATAGAAGTGTATCCGGGTACAGCACCAATCTCTATAC

CACCGTATCGAATGTCACCCACTGAGTTGAAAGAGTTGAAAGTGCAGTTACAGGACTTGT

CAGATCGTGGATTTATTAGACCGAGCATCTCACCTTGGGGAGCTCCAGTATTGTTTGTTA

AAAAGAAAGATGGATCGATGCGGCTTTGTATTGATTACCGGCAGTTAAACAAAGTGACGA

TCAAGAACCGGTATCCGTTACCCCGTATAGATGATTTATTTGATCAACTAAAAGGAGCTT

CAGTATTTTCAAAGATTGACTTAAGATCTGGGTATTATCAGCTGAAGGTAAAAGAAAGTG

ATGTTCCGAAGACTGCATTTCGTACTCGATATGGTCATTATGAATTTTTGGTGATGCCGT

TCGGGTTGACTAATGCTCCAGCTGCTTTTATGGATCTGATGAATCGTATTTTTCAGCCGT

ATTTAGATCAGTTTGTGGTGGTTTTTATTGATGACATCTTGGTTTATTCGAAGTCAGAGT

CAGAGCATGATCAGCATCTCAGAACCGTGCTACAAATTCTGCGAGAAAAACAGTTGTACG

GGAAACTAAGTAAATGTGAATTCTGGTTATCAGAGGTAGTATTCTTGGGACATGTTGTAT

CTGCGGATGGGATTAGAGTTGATCCGAAGAAGATCGAGGCAATTGTTCAATGGAAGGCAC

CAAAGAATGTATCAGAGGTACGCAGTTTTCTTGGTTTGGCTGGGTATTACAGAAGATTTG

TAAATGGGTTTTCGAAGATAGCTTTGCCGATGACCAAATTACTACAGAAGAATGTTCCAT

TTATCTGGGATGATCAGTGTCAGAGGAGCTTTGAAACATTGAAACAGATGTTGACAGAGG

CACCAGTTTTAACTTTACCAGAATCAGGGAAAGATTTCATAGTGTACAGTGATGCTTCTT

TGAATGGTTTGGGTTGTGTATTGATGCAAGAAGGAAAAGTAATAGCTTATGCATCTCGAC

AGTTGAAGTCACATGAACGCAACTACCCGACACACGATTTAGAGTTAGCTGCTGTAATCT

TTGCATTGAAGATTTGGATACATTACTTGTATGGTGAGAAATGTTATATTTACACTGATC

ATAAAAGTCTAAAATATCTTCTGTCACAAAAGGAGTTGAATCTGAGACAGAGACGGTGGA

TTGAACTTCTGAAAGATTATGATTGTGTTATAGATTATCATCCAGGGAAGGCAAATGTGG

TAGCAGATGCATTGAGTAGAAAAGCAGCGATTGAATTACGAGCAATGTTCGCTCGACTTA

GTATTAAGGATGATGGAAGTTTGTTAGCTGAGTTAAGAGTCAAGCCGGTGATGTTTGATC

AAATCAGAGCAGCACAGTTAAAAGATGAAAAGTTGATGAGGAAAAGAGAAATGGTACAGT

ATGGTGCGGTAGAAAATTTTAGTATTGACGAGCATGATTGTTTGAGATTTCGAAATCGAA

TTTGTGTTCCATCTACTTCTGAGATTAAAGAATTGATTCTCCGAGAAGCACATAATAGTA

TTTTTGCTTTGCACCCAGGAGGAACGAAGATGTATCGTGATCTACGAGAACTGTATTGGT

GGCCAGGAATGAAGAAAGATATAGTTGAATATGTCAGTAAATGCTTGACTTGTCAGCGGG

TAAAAGCAGAACATCAGGTACCAACAGGCCTGTTACAGCCTATTACTATTCCCGAGTGGA

AATGGGATCGCATTACCATGGATTTTGTTACGGGGTTGCCATTGTCAGTGAGTAAAAAGA

ATGCTATTTGGGTGATTGTTGATCGACTCACAAAATCAGCTCATTTTATAGCAGTTAGAA

CCGACTGGTCATTACAGAAGCTTGCCGAGGTTTATATTCGAGAAATTGTTAGATTACATG

GTATTCCGGTATCAATAATTTCAGACAGAGATCCTCGATTCACTTCGAGATTTTGGAAGC

AGCTGCATGAATCATTGGGTACTCGACTTAGTTTCAGTACAGCTTTTCATCCTCAAACTG

ATGGACAATCTGAACGAGTAATTCAGATATTAGAAGATATGCTTCGAGCTTGTGTCATTG

ATTTTGAATCAGGTTGGGAACGTTATTTACCATTGGCCGAGTTTGTTTATAATAATAGTT

TCCAATCTAGTATTCAAATGGCTCCATATGAAGCACTTTATGGTCGAAGGTGTCGATCAC

CAATATGTTGGACAAAATTAAGAGAAAGAAAAGTGATTGGGCCGGAATTGATTCAAGAGA

CAGAAGAAACAGTTAAAAAGATTAAAGATAGACTGAAAGCCGCTTTCGACAGACAGAAAT

CTTACGCAGACTTGAAACGACGAGACATTGAATATTCCGTTGGTGATAAGGTATTCCTCA

AAGTATCGCCGTGGAAGAAAATTTTGAGATTTGGTCGGAAGGGAAAATTAAGTCCGCGCT

TTATTGGGCCGTATGAGATAGTGGAAAGAATTGGGCCTGTTGCTTATCGATTATCCTTAC

CTCCAGAGTTACAGAAAATTCATGATGTTTTTCATGTTTCGATGCTTCGGAGATATAGAT

CGGATCCTTCTCATGTTATTCCCACTGAAGACATTGAACTTCGATCTGATTTAACTTATG

AAGAAGAACCAGTTCAAATATTAGCACGAGAAGTGAAAGAATTAAGAAATAAACGGGTTC

CTTTAGTACAAGTTTTATGGAGAAGCCATAGTGTGGAAGAAGCAACTTGGGAACCGGAAG

AGACAATGAGAGCACAATATCCTCATCTCTTCTCAGGTAAATTTCGAGGACGAAATTTAT

TAAGAGGGGGAGAAATGTAATGACCTAAAATTCATGGGCATCGGAAAAGTATAATATTGG

GCCTCCGTCCTAGTAAATTGAGTCCGAAAATAATTATTAGAAATATTTACGAGACTAGTA

GTGTGTTTAATTAGGTTTTAATTAAGTAAATTTAGCTTAATTTAGAGTAATTAGTAAAAA

GGATTAAATTGAATAAGAGTAAAAGTTTAATTATAGATTAAAGGAAAATAATAGGGACCA

AATGGGCAATTAAGCCACATTTGGAAGTTGAGGCGGCATAACATTGTAAAAATCTTAGAT

TTTTATATTATTATTTATATAAATATATAAATTAATTATAAAGTATATTATTAAATTAAT

TATATTATAAATATTATATTATTATATATAAAAGAAACAAAACAGAAAAGAAACAGAATA

GAAAGAACAAAGAAACAGAATAGAAGAGACGAAACAGGGGAGAAGCAGGGGAGAAAGAAG

AAAAAGAAGAAAAAAGGGGAAATAGGGTTTTTGAAGCTTGAAATTTAAATTGGTAAGTCA

AATTAGCCATTTTCTCTTAATTCTAATGTTTTAAAAGCTTTAAAACAAAGTTTTGATGGA

ATTAAGTTGATATTTTGTAAGTTCATAGGTTTTCAAGTATAGTTTATGTTGAACAAAAGA

GATGAATTAGGGATTAACTTGAAGGAATTTTAAGTTAGAATTGAAAAAGGGATTAAATTG

TAAAAGAAACTATAAGTTTTTTTTGTTTTAGGGACTAGATTGAGGAAAATTCGGAATTAA

GAAAATATGTTAAAAATTTAATAGTTAAATTTGAGTTTAAATGAAATTTGAATAGGAATA

AGGTGTGAATTGGTGTTATAAATTTGGTTATTAACATTTTTAATCAAAACAGTTTTGGGA

AGTAGCAATGGTCTGACTTTGAAAATTCACTAAAAATTTTATAAATTGAACTAGAGGATG

AACAAAATATGGAATTAAAGCTTATTGAGTCTAGTTTCTTATAGTAGAAACAATGTAAGC

AATTAATTGATGAATCAAGAGATATTTGAAATTTTGTAATACTGGTTCGGGGTGATTTCG

AGATGCCCTGTTTTAACTTTGGAAAATCATTAAAAATTGTACAAAAATTATTATGGAGTG

TAATTTATATATGTAAACTCCTTAATGAATCTAGTTTCAAAATAAATAAACAAGAACCTT

ATTCGAGTTCTGTACAATGAGATAATTTAGTTTTAGTGGAGAGAGGTCAGAACTGTCAAA

TGAAATAACAGGGGAGTATTTAACGAATAAACTGTATTAAATGGCTAGACCAAAAATTCT

GGAAATTTTATGATTAGAAGATATATGAGTCTAGTTTTAAGGAAAATTTACGGATATTAA

TTTGGAGTTTCGTAGCTCAAGATATAAATAATTTAGTAACAATGACCCAAGTAGACAGCT

TAATGGTGAAATTATATAAATACATTAAAAATGGTTAAATTTGCATGTTTAGGCTCATGA

ATTAAATTGAATCATGTTGTATTGATTATTATAAATTATTATTTTCGTAGCCAACAAAGA

ACCTAAAGCATCAGCATCGAAAGGAAAGGAGAAAGTCATCGAGGAGTAAACTCGAGAAAA

TTACGGTTTGTATTACTATAATTCAAGTTATTTATTATTAAATGTTAAATTTTAATTTAT

GTGTCTAGTAAATGAAATGTGAGGTAAGTATTATTATTATTATTATTATTATTATTATTA

TTATTATTATTATTATTATGAGTGGGAATTAAATTGAATAGTTGATATGAAATAATATTT

GAATTGTTTGTTGATTGAAAGCGGGAAATGAATTTAAATCGAATAGTGACCGATATTAAA

TTGAATGGAAATGTATTGAGTTGTGAAAATATGTTAATTGCGGATTAATTATTGATTGAA

AGGTGGAAAAATGATTGAATTGAAAGTGTGAGAAAGTGTGATTGAATTGGGATTATATGT

GATTTAAATACCCTATTAACTAGTCGGGCTGAGTCGGATATAGTTGGCATGCCATAGGAT

TGGAAGAGTTCAGGGATACTTCGACCTCGAGTCGATGAGACACTGGGTGTCACTATATTT

CTTCGGATAGATTCGATGAGGTACTGGGTACCAACTTTCTTCGGCTTTGCCGATGAGACA

CTGGGTGTCAACTATTGCTTCGAACTATCCGATGAGGCACTGGGTGCCATTCTGGTGTGT

TTGGTTGGATCCGTGTATTCGCCAAAGTCCGAGTTTTGTTAATAGGGTAAATGATGAAAT

GATAAACCGAACGAGTTGGTCAAACGAGCTATTGAAATGATATGAAAAAGTTGAATTGTG

AATTGAAATGTGAAATGAGATTGAGAAATGAACCTAAGGTTCGTGAATTATTCAAACTCA

AATTGTGGATATACGATATTGGTTGATGAATTGCTATTGTTGAAATATTTAATTTAAATT

GTATATACGATTTATGCTTTACATGTACATTATTGTTATAATTTGAATTATGGTAATACC

ACTGAGTATGAATTACTCAGCGTACGGTTGTTTCCGTGCGCAGGTCAATAGAAGTCAAAG

GTCTCGGTTCAGCATCCAGATTAATCCCGGCTTCGGCAAAACTTGGTGATGTATTTTTCC

TTTGGTAAAGGTGGCATGTACATAGATTGTGTATAAAGGTTATTATGTTTTATTATATAA

TGGTTAAAAATGTTAGTATTAAAAGTTTATGGATTTTAATGAAAGAAGTCTATCTATTTT

ATCTAATTAGTACATTGTTAAATTTTAAATTGGTATTAGATTGAGTTTGATTAGAAGTAT

TTAGAATAGAAAATGTGAATGTGAAATGAATTGGTTGAATTGATGATATTTGGGAACTAT

ATGGTTTTAATTTGC

>Delta_Pearl

ATGTAATGACCCAAAATTCATGGGCATCGGAAAAGTATAATATCGGGCCTCCGTCCTAGT

AAATTGAGTCCGAAAATAATTATTAGAAATATTTACGAGACTAGTAGTGTGTTTAATTAG

GTTTTAATTAAGTAAATTTAGCTTAATTTAGAGTAATTAGTAAAAAGGATTAAATTGAAT

AAGAGTAAAAGTTTAATTATAGATTAAAGGAAAATAATAGGGACCAAATGGGCAATTAAG

CCACATTTGGAAGTTGAGGCGGCATAACATTGTAAAAATCTTAGATTTTTATATTATTAT

TTATATAAATATATAAATTAATTATAAAGTATATTATTAAATTAATTATATTATAAATAT

TATATTATTATATATAAAAGAAACAAAACAGAAAAGAAACAGAATAGAAAGAACAAAGAA

ACAGAATAGAAGAGACGAAACAGGGGAGAAGCAGGGGAGAAAGAAGAAAAAGAAGAAAAA

AGGGGAAATAGGGTTTTTGAAGCTTGAAATTTAAATTGGTAAGTCAAATTAGCCATTTTC

TCTTAATTCTAATGTTTTAAAAGCTTTAAAACAAAGTTTTGATGGAATTAAGTTGATATT

TTGTAAGTTCATAGGTTTTCAAGTATAGTTTATGTTGAACAAAAGAGATGAATTAGGGAT

TAACTTGAAGGAATTTTAAGTTAGAATTGAAAAAGGGATTAAATTGTAAAAGAAACTATA

AGTTTTTTTTGTTTTAGGGACTAGATTGAGGAAAATTCGGAATTAAGAAAATATGTTAAA

AATTTAATAGTTAAATTTGAGTTTAAATGAAATTTGAATAGGAATAAGGTGTGAATTGGT

GTTATAAATTTGGTTATTAACATTTTTAATCAAAACAGTTTTGGGAAGTAGCAATGGTCT

GACTTTGAAAATTCACTAAAAATTTTATAAATTGAACTAGAGGATGAACAAAATATGGAA

TTAAAGCTTATTGAGTCTAGTTTCTTATAGTAGAAACAATGTAAGCAATTAATTGATGAA

TCAAGAGATATTTGAAATTTTGTAATACTGGTTCGGGGTGATTTCGAGATGCCCTGTTTT

AACTTTGGAAAATCATTAAAAATTGTACAAAAATTATTATGGAGTGTAATTTATATATGT

GAACTCCTTAATGAATCTAGTTTCAAAATAAATAAACAAGAACCTTATTCGAGTTCTGTA

CAATGAGATAATTTAGTTTTAGTGGAGAGAGGTCAGAACTGTCAAATGAAATAACAGGGG

AGTATTTAACGAATAAACTGTATTAAATGGCTAGACCAAAAATTCTGGAAATTTTATGAT

TAGAAGATATATGAGTCTAGTTTTAAGGAAAATTTACGGATATTAATTTGGAGTTTCGTA

GCTCAAGATATAAATAATTTAGTAACAATGACCCAAGTAGACAGCTTAATGGTGAAATTA

TATAAATACATTAAAAATGGTTAAATTTGCATGTTTAGGCTCATGAATTAAATTGAATCA

TGTTGTATTGATTATTATAAATTATTATTTTCGTAGCCAACAAAGAACCTAAAGCATCAG

CATCGAAAGGAAAGGAGAAAGTCATCGAGGAGTAAACTCGAGAAAATTACGGTTTGTATT

ACTATAATTCAAGTTATTTATTATTAAATGTTAAATTTTAATTTATGTGTCTAGTAAATG

AAATGTGAGGTAAGTATTATTATTATTATTATTATTATTATTATTATTATTATTATTATT

ATGAGTGGGAATTAAATTGAATAGTTGATATGAAATAATATTTGAATTGTTTGTTGATTG

AAAGCGGGAAATGAATTTAAATCGAATAGTGACCGATATTAAATTGAATGGAAATGTATT

GAGTTGTGAAAATATGTTAATTGCGGATTAATTATTGATTGAAAGGTGGAAAAATGATTG

AATTGAAAGTGTGAGAAAGTGTGATTGAATTGGGATTATATGTGATTTAAATACCCTATT

AACTAGTCGGGCTGAGTCGGATATAGTTGGCATGCCATAGGATTGGAAGAGTTCAGGGAT

ACTTCGACCTCGAGTCGATGAGACACTGGGTGATTTCTTCGGATAGATTGGATGAGGTAC

TGGGTACCAACTTTCTTCGGCTTTGCCGATGAGACACTGGGTGTCAACTATTGCTTCGAA

CTATCCGATGAGGCACTGGGTGCCATTCTGGTGTGTTTGGTTGGATCCGTGTATCCGCCA

AAGTCCGAGTTTTGTTAATAGGGTAAATGATGAAATGATAAACCGAACGAGTTGGTCAAA

CGAGCTATTGAAATGATATGAAAAAGTTGAATTGTGAATTGAAATGTGAAATGAGATTGA

GAAATGAACCTAAGGTTCGTGAATTATTCAAACTCAAATTGTGGATATACGATATTGGTT

GATGAATTGCTATTGTTGAAATATTTAATTTAAATTGTATATACGATTTATGCTTTACAT

GTACATTATTGTTATAATTTGAATTATGGTAATACCACTGAGTATGAATTACTCAGCGTA

CGGTTGTTTCCGTGCGCAGGTCAATAGAAGTCAAAGGTCTCGGTTCAGCATCCAGATTAA

TCCCGGCTTCGGCAAAACTTGGTGATGTATTTTTCCTTTGGTAAAGGTGGCATGTACATA

GATTGTGTATAAAGGTTATTATGTTTTATTATATAATGGTTAAAAATGTTAGTATTAAAA

GTTTATGGATTTTAATGAAAGAAGTCTATCTATTTTATCTAATTAGTACATTGTTAAATT

TTAAATTGGTATTGTGTAGATTGAGTTTGATTAGAAGTATTTAGAATAGAAAATGTGAAT

GTGAAATGAATTGGTTGAATTGATGATATTTGGGAACTATATGGTTTTAATTTGCAGGGG

GTTTTATGTAAAAATAAGCAGAAATGCTGCCGAAATTTTTATAAAAAAAAATGAAGTCAT

TTGGTAAACAAATTAATAAATTTTATGAATTATTTTAATATATTGGTTATTTATTTAAGA

ATTGTTGTAAATCGTTCGATACGTCCGGTAGTGCCTCGTAATTCTGTTCCGGCGACGGTT

CGGGGTTAAGGGGTGTTACATTTTATGGTATCAGAGCTATCAGGTTTAGCCGATTCTCGG

CCTAAATCGAGCTCGGAATTGAGTCTAGATGTACATGCCACTGTCGAGTTAAACTGAGTC

GGGATTTTTGGATGCTGACCTATTTGTTTGTTTTGTTTTATAGATTAAAGATGTCTGAAG

AAAGAATAAATGATACTGATGAAAGAATGTATAGTGAAGATAGAGAATTAGATGAAACAG

AATCTGTTGCACCGAGTGTGAATCCGTTAGGCAACCAACCTTCTAATGTAGAACGAGAAA

ATGTCAGAGATAGAGATGAATCCCAATTACTGAGAATTATAGCTGATGCATTACAAAGAG

TAGCAGGAACTACTCCTGTTACGACTTCAGTACCTACTGTTAGACGGGCTCCGATAAAGG

AACTGAGGAAATATGGTGCCACTGAATTTATGGGTCTAAAAGGAGTTGATCCATCCATAG

CTGAAAATTGGATGGAGTCGACTAAAAGAATTTTGCAGCAATTGGATTGTACCCCCCGAG

AGTGTTTAATCTGTGCCGTATCGTTATTACAAGGGGAGGCTTATCTATGGTGGGAATCAG

TGGTTCGACATTTACCAGAGAGTCAGATAACGTGGGATCTATTTCAGAAGGAGTTTCAAA

AGAAATATATCGGAGAGATGTATATTGAAGACAAGAAACAAGAGTTTTTGTTGCTACAAC

AGGGTGATATGTCAGTAATAGATTATGAGAGGGAATTCTCGAGACTCAGTAGATATGCCT

CCGAGTTTATTCCGACAGAAGCCGATAGTTGTAAAAGATTTTTACGGGGTTTACGAGACG

AGATCAAAGTGCAGCTAGTATCCCATCGGATCACTGAGTTAGTAGATTTGATTGAACGAG

CTAAAATGGTGGAACAAGTTCTGGGCCTCGACAAAAAGACTGAAGTTGTTAGACCAACCG

GGAAGCGTACAGGAACTACCAGTTCGAATCCTCAGCCGAAAAGACCAAAGGAATTCCAAA

GTGGTTGGAGATCCAGTTTCAGGTCAGACAGAGGTGGTAGAAATAGGGGAAAACAGACGA

TGACATCTACTGGCAGTGTGAAAGGTCCTTCCCGAGAAATAGATATTCCAGACTGCCAAC

ACTGCGGAAAGAAACACAGAGGGGAATGTTGGAAATTAACTAGAGGCTGTTTTCGATGTG

GTTCTACAGACCATTTCATCAGAGACTGTCCGAAAGTTGATAGTACTGTACCCGTGACAT

CACAGAGATCGGTATCTACAGCTAGAGGCAGAGGGTTAGGAAGAGGTGGTTCGGTTTCAA

GGGGAGGAAGTATTAGGAGAAGCAATGATATTGCTACTCAGCAGTCTGAGGCTAAAGTAC

CTGCCAGAGCTTATGTGGTCAGAACACAGGAAGAAGGTGACGCCCACGATGTAGTAACAG

GTATATTCTTACTATATTCTGAGCCTGTTTATGCTTTAATTGATCCCGGATCTTCACATT

CTTATATAAATTCAAAATTAGTTGAATTGGGAAAATTTAATTCTGAAATATCTAGAGTGA

CTGTAGAAGTGTCGAGTCCGTTGGGGCAAACAGTATTAGTGAATCAGATCTGTCCGAGAT

GCCCGTTAATTATACAAAATAAAACTTTTCCTATTGACCTGTTGATTATGCCATTTGGAG

ATTTTGATATAATACTGGGGATGGATTGGTTGGCTGAGCACGGAGTGGTATTGGATTGTT

ATAAAAAGAAGTTTAGTATTCAGACAGAAGACGGGGACAGAATTGAAGTAAATGGTATCC

GTACTAATGGGCCGACACGTATTATTTCGGCAATAAAGGCTAATAAATTGCTTCAGCGGG

GTTGTACAGCGTATTTAGCCTATGTTATTAATTCTGATTTGGTTGGTAGTCAGTGCAGTA

AGATTAGAACCGTATGTGAGTTTCCAGATGTATTTCCTGAAGAGCTACCGGGTTTACCAC

CTGACAGAGAGGTTGAATTTGCTATAGAAGTGTATCCGGGTACAGCACCAATCTCTATAC

CACCGTATCGAATGTCACCCACTGAGTTGAAAGAGTTGAAAGTGCAGTTACAGGACTTGT

CAGATCGTGGATTTATTAGACCGAGCATCTCACCTTGGGGAGCTCCAGTATTGTTTGTTA

AAAAGAAAGATGGATCGATGCGGCTTTGTATTGATTACCGGCAGTTAAACAAAGTGACGA

TCAAGAACCGGTATCCGTTACCCCGTATAGATGATTTATTTGATCAACTAAAAGGAGCTT

CAGTATTTTCAAAGATTGACTTAAGATCTGGGTATTATCAGCTGAAGGTAAAAGAAAGTG

ATGTTCCGAAGACTGCATTTCGTACTCGATATGGTCATTATGAATTTTTGGTGATGCCGT

TCGGGTTGACTAATGCTCCAGCTGCTTTTATGGATCTGATGAATCGTATTTTTCAGCCGT

ATTTAGATCAGTTTGTGGTGGTTTTTATTGATGACATCTTGGTTTATTCGAAGTCAGAGT

CAGAGCATGATCAGCATCTCAGAACCGTGCTACAAATTCTGCGAGAAAAACAGTTGTACG

GGAAACTAAGTAAATGTGAATTCTGGTTATCAGAGGTAGTATTCTTGGGACATGTTGTAT

CTGCGGATGGGATTAGAGTTGATCCGAAGAAGATCGAGGCAATTGTTCAATGGAAGGCAC

CAAAGAATGTATCAGAGGTACGCAGTTTTCTTGGTTTGGCTGGGTATTACAGAAGATTTG

TAAATGGGTTTTCGAAGATAGCTTTGCCGATGACCAAATTACTACAGAAGAATGTTCCAT

TTATCTGGGATGATCAGTGTCAGAGGAGCTTTGAAACATTGAAACAGATGTTGACAGAGG

CACCAGTTTTAACTTTACCAGAATCAGGGAAAGATTTCATAGTGTACAGTGATGCTTCTT

TGAATGGTTTGGGTTGTGTATTGATGCAAGAAGGAAAAGTAATAGCTTATGCATCTCGAC

AGTTGAAGTCACATGAACGCAACTACCCGACACACGATTTAGAGTTAGCTGCTGTAATCT

TTGCATTGAAGATTTGGATACATTACTTGTATGGTGAGAAATGTTATATTTACACTGATC

ATAAAAGTCTAAAATATCTTCTGTCACAAAAGGAGTTGAATCTGAGACAGAGACGGTGGA

TTGAACTTCTGAAAGATTATGATTGTGTTATAGATTATCATCCAGGGAAGGCAAATGTGG

TAGCAGATGCATTGAGTAGAAAAGCAGCGATTGAATTACGAGCAATGTTCGCTCGACTTA

GTATTAAGGATGATGGAAGTTTGTTAGCTGAGTTAAGAGTCAAGCCGGTGAGGTTTGATC

AAATCAGAGCAGCACAGTTAAAAGATGAAAAGTTGATGAGGAAAAGAGAAATGGTACAGT

ATGGTGCGGTAGAAAATTTTAGTATTGACGAGCATGATTGTTTGAGATTTCGAAATCGAA

TTTGTGTTCCATCTACTTCTGAGATTAAAGAATTGATTCTCCGAGAAGCACATAATAGTA

TTTTTGCTTTGCACCCAGGAGGAACGAAGATGTATCGTGATCTACGAGAACTGTATTGGT

GGCCAGGAATGAAGAAAGATATAGTTGAATATGTCAGTAAATGCTTGACTTGTCAGCGGG

TAAAAGCAGAACATCAGGTACCAACAGGCCTGTTACAGCCTATTACTATTCCCGAGTGGA

AATGGGATCGCATTACCATGGATTTTGTTACGGGGTTGCCATTGTCAGTGAGTAAAAAGA

ATGCTATTTGGGTGATTGTTGATCGACTCACAAAATCAGCTCATTTTATAGCAGTTAGAA

CCGACTGGTCATTACAGAAGCTTGCCGAGGTTTATATTCGAGAAATTGTTAGATTACATG

GTATTCCGGTATCAATAATTTCAGACAGAGATCCTCGATTCACTTCGAGATTTTGGAAGC

AGCTGCATGAATCATTGGGTACTCGACTTAGTTTCAGTACAGCTTTTCATCCTCAAACTG

ATGGACAATCTGAACGAGTAATTCAGATATTAGAAGATATGCTTCGAGCTTGTGTCATTG

ATTTTGAATCAGGTTGGGAACGTTATTTACCATTGGCCGAGTTTGTTTATAATAATAGTT

TCCAATCTAGTATTCAAATGGCTCCATATGAAGCACTTTATGGTCGAAGGTGTCGATCAC

CAATATGTTGGACAAAATTAAGAGAAAGAAAAGTGATTGGGCCGGAATTGATTCAAGAGA

CAGAAGAAACAGTTAAAAAGATTAAAGATAGACTGAAAGCCGCTTTCGACAGACAGAAAT

CTTACGCAGACTTGAAACGACGAGACATTGAATATTCCGTTGGTGATAAGGTATTCCTCA

AAGTATCGCCGTGGAAGAAAATTTTGAGATTTGGTCGGAAGGGAAAATTAAGTCCGCGCT

TTATTGGGCCGTATGAGATAGTGGAAAGAATTGGGCCTGTTGCTTATCGATTATCCTTAC

CTCCAGAGTTACAGAAAATTCATGATGTTTTTCATGTTTCGATGCTTCGGAGATATAGAT

CGGATCCTTCTCATGTTATTCCCACTGAAGACATTGAACTTCGATCTGATTTAACTTATG

AAGAAGAACCAGTTCAAATATTAGCACGAGAAGTGAAAGAATTAAGAAATAAACGGGTTC

CTTTAGTACAAGTTTTATGGAGAAGCCATAGTGTGGAAGAAGCAACTTGGGAACCGGAAG

AGACAATGAGAGCACAATATCCTCATCTCTTCTCAGGTAAATTTCGAGGACGAAATTTAT

TAAGAGGGGGAGAAATGTAATGACCTAAAATTCATGGGCATCGGAAAAGTATAATATTGG

GCCTCCGTCCTAGTAAATTGAGTCCGAAAATAATTATTAGAAATATTTACGAGACTAGTA

GTGTGTTTAATTAGGTTTTAATTAAGTAAATTTAGCTTAATTTAGAGTAATTAGTAAAAA

GGATTAAATTGAATAAGAGTAAAAGTTTAATTATAGATTAAAGGAAAATAATAGGGACCA

AATGGGCAATTAAGCCACATTTGGAAGTTGAGGCGGCATAACATTGTAAAAATCTTAGAT

TTTTATATTATTATTTATATAAATATATAAATTAATTATAAAGTATATTATTAAATTAAT

TATATTATAAATATTATATTATTATATATAAAAGAAACAAAACAGAAAAGAAACAGAATA

GAAAGAACAAAGAAACAGAATAGAAGAGACGAAACAGGGGAGAAGCAGGGGAGAAAGAAG

AAAAAGAAGAAAAAAGGGGAAATAGGGTTTTTGAAGCTTGAAATTTAAATTGGTAAGTCA

AATTAGCCATTTTCTCTTAATTCTAATGTTTTAAAAGCTTTAAAACAAAGTTTTGATGGA

ATTAAGTTGATATTTTGTAAGTTCATAGGTTTTCAAGTATAGTTTATGTTGAACAAAAGA

GATGAATTAGGGATTAACTTGAAGGAATTTTAAGTTAGAATTGAAAAAGGGATTAAATTG

TAAAAGAAACTATAAGTTTTTTTTGTTTTAGGGACTAGATTGAGGAAAATTCGGAATTAA

GAAAATATGTTAAAAATTTAATAGTTAAATTTGAGTTTAAATGAAATTTGAATAGGAATA

AGGTGTGAATTGGTGTTATAAATTTGGTTATTAACATTTTTAATCAAAACAGTTTTGGGA

AGTAGCAATGGTCTGACTTTGAAAATTCACTAAAAATTTTATAAATTGAACTAGAGGATG

AACAAAATATGGAATTAAAGCTTATTGAGTCTAGTTTCTTATAGTAGAAACAATGTAAGC

AATTAATTGATGAATCAAGAGATATTTGAAATTTTGTAATACTGGTTCGGGGTGATTTCG

AGATGCCCTGTTTTAACTTTGGAAAATCATTAAAAATTGTACAAAAATTATTATGGAGTG

TAATTTATATATGTAAACTCCTTAATGAATCTAGTTTCAAAATAAATAAACAAGAACCTT

ATTCGAGTTCTGTACAATGAGATAATTTAGTTTTAGTGGAGAGAGGTCAGAACTGTCAAA

TGAAATAACAGGGGAGTATTTAACGAATAAACTGTATTAAATGGCTAGACCAAAAATTCT

GGAAATTTTATGATTAGAAGATATATGAGTCTAGTTTTAAGGAAAATTTACGGATATTAA

TTTGGAGTTTCGTAGCTCAAGATATAAATAATTTAGTAACAATGACCCAAGTAGACAGCT

TAATGGTGAAATTATATAAATACATTAAAAATGGTTAAATTTGCATGTTTAGGCTCATGA

ATTAAATTGAATCATGTTGTATTGATTATTATAAATTATTATTTTCGTAGCCAACAAAGA

ACCTAAAGCATCAGCATCGAAAGGAAAGGAGAAAGTCATCGAGGAGTAAACTCGAGAAAA

TTACGGTTTGTATTACTATAATTCAAGTTATTTATTATTAAATGTTAAATTTTAATTTAT

GTGTCTAGTAAATGAAATGTGAGGTAAGTATTATTATTATTATTATTATTATTATTATTA

TTATTATTATTATTATTATGAGTGGGAATTAAATTGAATAGTTGATATGAAATAATATTT

GAATTGTTTGTTGATTGAAAGCGGGAAATGAATTTAAATCGAATAGTGACCGATATTAAA

TTGAATGGAAATGTATTGAGTTGTGAAAATATGTTAATTGCGGATTAATTATTGATTGAA

AGGTGGAAAAATGATTGAATTGAAAGTGTGAGAAAGTGTGATTGAATTGGGATTATATGT

GATTTAAATACCCTATTAACTAGTCGGGCTGAGTCGGATATAGTTGGCATGCCATAGGAT

TGGAAGAGTTCAGGGATACTTCGACCTCGAGTCGATGAGACACTGGGTGTCACTATATTT

CTTCGGATAGATTCGATGAGGTACTGGGTACCAACTTTCTTCGGCTTTGCCGATGAGACA

CTGGGTGTCAACTATTGCTTCGAACTATCCGATGAGGCACTGGGTGCCATTCTGGTGTGT

TTGGTTGGATCCGTGTATTCGCCAAAGTCCGAGTTTTGTTAATAGGGTAAATGATGAAAT

GATAAACCGAACGAGTTGGTCAAACGAGCTATTGAAATGATATGAAAAAGTTGAATTGTG

AATTGAAATGTGAAATGAGATTGAGAAATGAACCTAAGGTTCGTGAATTATTCAAACTCA

AATTGTGGATATACGATATTGGTTGATGAATTGCTATTGTTGAAATATTTAATTTAAATT

GTATATACGATTTATGCTTTACATGTACATTATTGTTATAATTTGAATTATGGTAATACC

ACTGAGTATGAATTACTCAGCGTACGGTTGTTTCCGTGCGCAGGTCAATAGAAGTCAAAG

GTCTCGGTTCAGCATCCAGATTAATCCCGGCTTCGGCAAAACTTGGTGATGTATTTTTCC

TTTGGTAAAGGTGGCATGTACATAGATTGTGTATAAAGGTTATTATGTTTTATTATATAA

TGGTTAAAAATGTTAGTATTAAAAGTTTATGGATTTTAATGAAAGAAGTCTATCTATTTT

ATCTAATTAGTACATTGTTAAATTTTAAATTGGTATTAGATTGAGTTTGATTAGAAGTAT

TTAGAATAGAAAATGTGAATGTGAAATGAATTGGTTGAATTGATGATATTTGGGAACTAT

ATGGTTTTAATTTGC

>Deltapine10

ATGTAATGACCCAAAATTCATGGGCATCGGAAAAGTATAATATCGGGCCTCCGTCCTAGT

AAATTGAGTCCGAAAATAATTATTAGAAATATTTACGAGACTAGTAGTGTGTTTAATTAG

GTTTTAATTAAGTAAATTTAGCTTAATTTAGAGTAATTAGTAAAAAGGATTAAATTGAAT

AAGAGTAAAAGTTTAATTATAGATTAAAGGAAAATAATAGGGACCAAATGGGCAATTAAG

CCACATTTGGAAGTTGAGGCGGCATAACATTGTAAAAATCTTAGATTTTTATATTATTAT

TTATATAAATATATAAATTAATTATAAAGTATATTATTAAATTAATTATATTATAAATAT

TATATTATTATATATAAAAGAAACAAAACAGAAAAGAAACAGAATAGAAAGAACAAAGAA

ACAGAATAGAAGAGACGAAACAGGGGAGAAGCAGGGGAGAAAGAAGAAAAAGAAGAAAAA

AGGGGAAATAGGGTTTTTGAAGCTTGAAATTTAAATTGGTAAGTCAAATTAGCCATTTTC

TCTTAATTCTAATGTTTTAAAAGCTTTAAAACAAAGTTTTGATGGAATTAAGTTGATATT

TTGTAAGTTCATAGGTTTTCAAGTATAGTTTATGTTGAACAAAAGAGATGAATTAGGGAT

TAACTTGAAGGAATTTTAAGTTAGAATTGAAAAAGGGATTAAATTGTAAAAGAAACTATA

AGTTTTTTTTGTTTTAGGGACTAGATTGAGGAAAATTCGGAATTAAGAAAATATGTTAAA

AATTTAATAGTTAAATTTGAGTTTAAATGAAATTTGAATAGGAATAAGGTGTGAATTGGT

GTTATAAATTTGGTTATTAACATTTTTAATCAAAACAGTTTTGGGAAGTAGCAATGGTCT

GACTTTGAAAATTCACTAAAAATTTTATAAATTGAACTAGAGGATGAACAAAATATGGAA

TTAAAGCTTATTGAGTCTAGTTTCTTATAGTAGAAACAATGTAAGCAATTAATTGATGAA

TCAAGAGATATTTGAAATTTTGTAATACTGGTTCGGGGTGATTTCGAGATGCCCTGTTTT

AACTTTGGAAAATCATTAAAAATTGTACAAAAATTATTATGGAGTGTAATTTATATATGT

GAACTCCTTAATGAATCTAGTTTCAAAATAAATAAACAAGAACCTTATTCGAGTTCTGTA

CAATGAGATAATTTAGTTTTAGTGGAGAGAGGTCAGAACTGTCAAATGAAATAACAGGGG

AGTATTTAACGAATAAACTGTATTAAATGGCTAGACCAAAAATTCTGGAAATTTTATGAT

TAGAAGATATATGAGTCTAGTTTTAAGGAAAATTTACGGATATTAATTTGGAGTTTCGTA

GCTCAAGATATAAATAATTTAGTAACAATGACCCAAGTAGACAGCTTAATGGTGAAATTA

TATAAATACATTAAAAATGGTTAAATTTGCATGTTTAGGCTCATGAATTAAATTGAATCA

TGTTGTATTGATTATTATAAATTATTATTTTCGTAGCCAACAAAGAACCTAAAGCATCAG

CATCGAAAGGAAAGGAGAAAGTCATCGAGGAGTAAACTCGAGAAAATTACGGTTTGTATT

ACTATAATTCAAGTTATTTATTATTAAATGTTAAATTTTAATTTATGTGTCTAGTAAATG

AAATGTGAGGTAAGTATTATTATTATTATTATTATTATTATTATTATTATTATTATTATT

ATGAGTGGGAATTAAATTGAATAGTTGATATGAAATAATATTTGAATTGTTTGTTGATTG

AAAGCGGGAAATGAATTTAAATCGAATAGTGACCGATATTAAATTGAATGGAAATGTATT

GAGTTGTGAAAATATGTTAATTGCGGATTAATTATTGATTGAAAGGTGGAAAAATGATTG

AATTGAAAGTGTGAGAAAGTGTGATTGAATTGGGATTATATGTGATTTAAATACCCTATT

AACTAGTCGGGCTGAGTCGGATATAGTTGGCATGCCATAGGATTGGAAGAGTTCAGGGAT

ACTTCGACCTCGAGTCGATGAGACACTGGGTGATTTCTTCGGATAGATTGGATGAGGTAC

TGGGTACCAACTTTCTTCGGCTTTGCCGATGAGACACTGGGTGTCAACTATTGCTTCGAA

CTATCCGATGAGGCACTGGGTGCCATTCTGGTGTGTTTGGTTGGATCCGTGTATCCGCCA

AAGTCCGAGTTTTGTTAATAGGGTAAATGATGAAATGATAAACCGAACGAGTTGGTCAAA

CGAGCTATTGAAATGATATGAAAAAGTTGAATTGTGAATTGAAATGTGAAATGAGATTGA

GAAATGAACCTAAGGTTCGTGAATTATTCAAACTCAAATTGTGGATATACGATATTGGTT

GATGAATTGCTATTGTTGAAATATTTAATTTAAATTGTATATACGATTTATGCTTTACAT

GTACATTATTGTTATAATTTGAATTATGGTAATACCACTGAGTATGAATTACTCAGCGTA

CGGTTGTTTCCGTGCGCAGGTCAATAGAAGTCAAAGGTCTCGGTTCAGCATCCAGATTAA

TCCCGGCTTCGGCAAAACTTGGTGATGTATTTTTCCTTTGGTAAAGGTGGCATGTACATA

GATTGTGTATAAAGGTTATTATGTTTTATTATATAATGGTTAAAAATGTTAGTATTAAAA

GTTTATGGATTTTAATGAAAGAAGTCTATCTATTTTATCTAATTAGTACATTGTTAAATT

TTAAATTGGTATTGTGTAGATTGAGTTTGATTAGAAGTATTTAGAATAGAAAATGTGAAT

GTGAAATGAATTGGTTGAATTGATGATATTTGGGAACTATATGGTTTTAATTTGCAGGGG

GTTTTATGTAAAAATAAGCAGAAATGCTGCCGAAATTTTTATAAAAAAAAATGAAGTCAT

TTGGTAAACAAATTAATAAATTTTATGAATTATTTTAATATATTGGTTATTTATTTAAGA

ATTGTTGTAAATCGTTCGATACGTCCGGTAGTGCCTCGTAATTCTGTTCCGGCGACGGTT

CGGGGTTAAGGGGTGTTACATTTTATGGTATCAGAGCTATCAGGTTTAGCCGATTCTCGG

CCTAAATCGAGCTCGGAATTGAGTCTAGATGTACATGCCACTGTCGAGTTAAACTGAGTC

GGGATTTTTGGATGCTGACCTATTTGTTTGTTTTGTTTTATAGATTAAAGATGTCTGAAG

AAAGAATAAATGATACTGATGAAAGAATGTATAGTGAAGATAGAGAATTAGATGAAACAG

AATCTGTTGCACCGAGTGTGAATCCGTTAGGCAACCAACCTTCTAATGTAGAACGAGAAA

ATGTCAGAGATAGAGATGAATCCCAATTACTGAGAATTATAGCTGATGCATTACAAAGAG

TAGCAGGAACTACTCCTGTTACGACTTCAGTACCTACTGTTAGACGGGCTCCGATAAAGG

AACTGAGGAAATATGGTGCCACTGAATTTATGGGTCTAAAAGGAGTTGATCCATCCATAG

CTGAAAATTGGATGGAGTCGACTAAAAGAATTTTGCAGCAATTGGATTGTACCCCCCGAG

AGTGTTTAATCTGTGCCGTATCGTTATTACAAGGGGAGGCTTATCTATGGTGGGAATCAG

TGGTTCGACATTTACCAGAGAGTCAGATAACGTGGGATCTATTTCAGAAGGAGTTTCAAA

AGAAATATATCGGAGAGATGTATATTGAAGACAAGAAACAAGAGTTTTTGTTGCTACAAC

AGGGTGATATGTCAGTAATAGATTATGAGAGGGAATTCTCGAGACTCAGTAGATATGCCT

CCGAGTTTATTCCGACAGAAGCCGATAGTTGTAAAAGATTTTTACGGGGTTTACGAGACG

AGATCAAAGTGCAGCTAGTATCCCATCGGATCACTGAGTTAGTAGATTTGATTGAACGAG

CTAAAATGGTGGAACAAGTTCTGGGCCTCGACAAAAAGACTGAAGTTGTTAGACCAACCG

GGAAGCGTACAGGAACTACCAGTTCGAATCCTCAGCCGAAAAGACCAAAGGAATTCCAAA

GTGGTTGGAGATCCAGTTTCAGGTCAGACAGAGGTGGTAGAAATAGGGGAAAACAGACGA

TGACATCTACTGGCAGTGTGAAAGGTCCTTCCCGAGAAATAGATATTCCAGACTGCCAAC

ACTGCGGAAAGAAACACAGAGGGGAATGTTGGAAATTAACTAGAGGCTGTTTTCGATGTG

GTTCTACAGACCATTTCATCAGAGACTGTCCGAAAGTTGATAGTACTGTACCCGTGACAT

CACAGAGATCGGTATCTACAGCTAGAGGCAGAGGGTTAGGAAGAGGTGGTTCGGTTTCAA

GGGGAGGAAGTATTAGGAGAAGCAATGATATTGCTACTCAGCAGTCTGAGGCTAAAGTAC

CTGCCAGAGCTTATGTGGTCAGAACACAGGAAGAAGGTGACGCCCACGATGTAGTAACAG

GTATATTCTTACTATATTCTGAGCCTGTTTATGCTTTAATTGATCCCGGATCTTCACATT

CTTATATAAATTCAAAATTAGTTGAATTGGGAAAATTTAATTCTGAAATATCTAGAGTGA

CTGTAGAAGTGTCGAGTCCGTTGGGGCAAACAGTATTAGTGAATCAGATCTGTCCGAGAT

GCCCGTTAATTATACAAAATAAAACTTTTCCTATTGACCTGTTGATTATGCCATTTGGAG

ATTTTGATATAATACTGGGGATGGATTGGTTGGCTGAGCACGGAGTGGTATTGGATTGTT

ATAAAAAGAAGTTTAGTATTCAGACAGAAGACGGGGACAGAATTGAAGTAAATGGTATCC

GTACTAATGGGCCGACACGTATTATTTCGGCAATAAAGGCTAATAAATTGCTTCAGCGGG

GTTGTACAGCGTATTTAGCCTATGTTATTAATTCTGATTTGGTTGGTAGTCAGTGCAGTA

AGATTAGAACCGTATGTGAGTTTCCAGATGTATTTCCTGAAGAGCTACCGGGTTTACCAC

CTGACAGAGAGGTTGAATTTGCTATAGAAGTGTATCCGGGTACAGCACCAATCTCTATAC

CACCGTATCGAATGTCACCCACTGAGTTGAAAGAGTTGAAAGTGCAGTTACAGGACTTGT

CAGATCGTGGATTTATTAGACCGAGCATCTCACCTTGGGGAGCTCCAGTATTGTTTGTTA

AAAAGAAAGATGGATCGATGCGGCTTTGTATTGATTACCGGCAGTTAAACAAAGTGACGA

TCAAGAACCGGTATCCGTTACCCCGTATAGATGATTTATTTGATCAACTAAAAGGAGCTT

CAGTATTTTCAAAGATTGACTTAAGATCTGGGTATTATCAGCTGAAGGTAAAAGAAAGTG

ATGTTCCGAAGACTGCATTTCGTACTCGATATGGTCATTATGAATTTTTGGTGATGCCGT

TCGGGTTGACTAATGCTCCAGCTGCTTTTATGGATCTGATGAATCGTATTTTTCAGCCGT

ATTTAGATCAGTTTGTGGTGGTTTTTATTGATGACATCTTGGTTTATTCGAAGTCAGAGT

CAGAGCATGATCAGCATCTCAGAACCGTGCTACAAATTCTGCGAGAAAAACAGTTGTACG

GGAAACTAAGTAAATGTGAATTCTGGTTATCAGAGGTAGTATTCTTGGGACATGTTGTAT

CTGCGGATGGGATTAGAGTTGATCCGAAGAAGATCGAGGCAATTGTTCAATGGAAGGCAC

CAAAGAATGTATCAGAGGTACGCAGTTTTCTTGGTTTGGCTGGGTATTACAGAAGATTTG

TAAATGGGTTTTCGAAGATAGCTTTGCCGATGACCAAATTACTACAGAAGAATGTTCCAT

TTATCTGGGATGATCAGTGTCAGAGGAGCTTTGAAACATTGAAACAGATGTTGACAGAGG

CACCAGTTTTAACTTTACCAGAATCAGGGAAAGATTTCATAGTGTACAGTGATGCTTCTT

TGAATGGTTTGGGTTGTGTATTGATGCAAGAAGGAAAAGTAATAGCTTATGCATCTCGAC

AGTTGAAGTCACATGAACGCAACTACCCGACACACGATTTAGAGTTAGCTGCTGTAATCT

TTGCATTGAAGATTTGGATACATTACTTGTATGGTGAGAAATGTTATATTTACACTGATC

ATAAAAGTCTAAAATATCTTCTGTCACAAAAGGAGTTGAATCTGAGACAGAGACGGTGGA

TTGAACTTCTGAAAGATTATGATTGTGTTATAGATTATCATCCAGGGAAGGCAAATGTGG

TAGCAGATGCATTGAGTAGAAAAGCAGCGATTGAATTACGAGCAATGTTCGCTCGACTTA

GTATTAAGGATGATGGAAGTTTGTTAGCTGAGTTAAGAGTCAAGCCGGTGATGTTTGATC

AAATCAGAGCAGCACAGTTAAAAGATGAAAAGTTGATGAGGAAAAGAGAAATGGTACAGT

ATGGTGCGGTAGAAAATTTTAGTATTGACGAGCATGATTGTTTGAGATTTCGAAATCGAA

TTTGTGTTCCATCTACTTCTGAGATTAAAGAATTGATTCTCCGAGAAGCACATAATAGTA

TTTTTGCTTTGCACCCAGGAGGAACGAAGATGTATCGTGATCTACGAGAACTGTATTGGT

GGCCAGGAATGAAGAAAGATATAGTTGAATATGTCAGTAAATGCTTGACTTGTCAGCGGG

TAAAAGCAGAACATCAGGTACCAACAGGCCTGTTACAGCCTATTACTATTCCCGAGTGGA

AATGGGATCGCATTACCATGGATTTTGTTACGGGGTTGCCATTGTCAGTGAGTAAAAAGA

ATGCTATTTGGGTGATTGTTGATCGACTCACAAAATCAGCTCATTTTATAGCAGTTAGAA

CCGACTGGTCATTACAGAAGCTTGCCGAGGTTTATATTCGAGAAATTGTTAGATTACATG

GTATTCCGGTATCAATAATTTCAGACAGAGATCCTCGATTCACTTCGAGATTTTGGAAGC

AGCTGCATGAATCATTGGGTACTCGACTTAGTTTCAGTACAGCTTTTCATCCTCAAACTG

ATGGACAATCTGAACGAGTAATTCAGATATTAGAAGATATGCTTCGAGCTTGTGTCATTG

ATTTTGAATCAGGTTGGGAACGTTATTTACCATTGGCCGAGTTTGTTTATAATAATAGTT

TCCAATCTAGTATTCAAATGGCTCCATATGAAGCACTTTATGGTCGAAGGTGTCGATCAC

CAATATGTTGGACAAAATTAAGAGAAAGAAAAGTGATTGGGCCGGAATTGATTCAAGAGA

CAGAAGAAACAGTTAAAAAGATTAAAGATAGACTGAAAGCCGCTTTCGACAGACAGAAAT

CTTACGCAGACTTGAAACGACGAGACATTGAATATTCCGTTGGTGATAAGGTATTCCTCA

AAGTATCGCCGTGGAAGAAAATTTTGAGATTTGGTCGGAAGGGAAAATTAAGTCCGCGCT

TTATTGGGCCGTATGAGATAGTGGAAAGAATTGGGCCTGTTGCTTATCGATTATCCTTAC

CTCCAGAGTTACAGAAAATTCATGATGTTTTTCATGTTTCGATGCTTCGGAGATATAGAT

CGGATCCTTCTCATGTTATTCCCACTGAAGACATTGAACTTCGATCTGATTTAACTTATG

AAGAAGAACCAGTTCAAATATTAGCACGAGAAGTGAAAGAATTAAGAAATAAACGGGTTC

CTTTAGTACAAGTTTTATGGAGAAGCCATAGTGTGGAAGAAGCAACTTGGGAACCGGAAG

AGACAATGAGAGCACAATATCCTCATCTCTTCTCAGGTAAATTTCGAGGACGAAATTTAT

TAAGAGGGGGAGAAATGTAATGACCTAAAATTCATGGGCATCGGAAAAGTATAATATTGG

GCCTCCGTCCTAGTAAATTGAGTCCGAAAATAATTATTAGAAATATTTACGAGACTAGTA

GTGTGTTTAATTAGGTTTTAATTAAGTAAATTTAGCTTAATTTAGAGTAATTAGTAAAAA

GGATTAAATTGAATAAGAGTAAAAGTTTAATTATAGATTAAAGGAAAATAATAGGGACCA

AATGGGCAATTAAGCCACATTTGGAAGTTGAGGCGGCATAACATTGTAAAAATCTTAGAT

TTTTATATTATTATTTATATAAATATATAAATTAATTATAAAGTATATTATTAAATTAAT

TATATTATAAATATTATATTATTATATATAAAAGAAACAAAACAGAAAAGAAACAGAATA

GAAAGAACAAAGAAACAGAATAGAAGAGACGAAACAGGGGAGAAGCAGGGGAGAAAGAAG

AAAAAGAAGAAAAAAGGGGAAATAGGGTTTTTGAAGCTTGAAATTTAAATTGGTAAGTCA

AATTAGCCATTTTCTCTTAATTCTAATGTTTTAAAAGCTTTAAAACAAAGTTTTGATGGA

ATTAAGTTGATATTTTGTAAGTTCATAGGTTTTCAAGTATAGTTTATGTTGAACAAAAGA

GATGAATTAGGGATTAACTTGAAGGAATTTTAAGTTAGAATTGAAAAAGGGATTAAATTG

TAAAAGAAACTATAAGTTTTTTTTGTTTTAGGGACTAGATTGAGGAAAATTCGGAATTAA

GAAAATATGTTAAAAATTTAATAGTTAAATTTGAGTTTAAATGAAATTTGAATAGGAATA

AGGTGTGAATTGGTGTTATAAATTTGGTTATTAACATTTTTAATCAAAACAGTTTTGGGA

AGTAGCAATGGTCTGACTTTGAAAATTCACTAAAAATTTTATAAATTGAACTAGAGGATG

AACAAAATATGGAATTAAAGCTTATTGAGTCTAGTTTCTTATAGTAGAAACAATGTAAGC

AATTAATTGATGAATCAAGAGATATTTGAAATTTTGTAATACTGGTTCGGGGTGATTTCG

AGATGCCCTGTTTTAACTTTGGAAAATCATTAAAAATTGTACAAAAATTATTATGGAGTG

TAATTTATATATGTAAACTCCTTAATGAATCTAGTTTCAAAATAAATAAACAAGAACCTT

ATTCGAGTTCTGTACAATGAGATAATTTAGTTTTAGTGGAGAGAGGTCAGAACTGTCAAA

TGAAATAACAGGGGAGTATTTAACGAATAAACTGTATTAAATGGCTAGACCAAAAATTCT

GGAAATTTTATGATTAGAAGATATATGAGTCTAGTTTTAAGGAAAATTTACGGATATTAA

TTTGGAGTTTCGTAGCTCAAGATATAAATAATTTAGTAACAATGACCCAAGTAGACAGCT

TAATGGTGAAATTATATAAATACATTAAAAATGGTTAAATTTGCATGTTTAGGCTCATGA

ATTAAATTGAATCATGTTGTATTGATTATTATAAATTATTATTTTCGTAGCCAACAAAGA

ACCTAAAGCATCAGCATCGAAAGGAAAGGAGAAAGTCATCGAGGAGTAAACTCGAGAAAA

TTACGGTTTGTATTACTATAATTCAAGTTATTTATTATTAAATGTTAAATTTTAATTTAT

GTGTCTAGTAAATGAAATGTGAGGTAAGTATTATTATTATTATTATTATTATTATTATTA

TTATTATTATTATTATTATGAGTGGGAATTAAATTGAATAGTTGATATGAAATAATATTT

GAATTGTTTGTTGATTGAAAGCGGGAAATGAATTTAAATCGAATAGTGACCGATATTAAA

TTGAATGGAAATGTATTGAGTTGTGAAAATATGTTAATTGCGGATTAATTATTGATTGAA

AGGTGGAAAAATGATTGAATTGAAAGTGTGAGAAAGTGTGATTGAATTGGGATTATATGT

GATTTAAATACCCTATTAACTAGTCGGGCTGAGTCGGATATAGTTGGCATGCCATAGGAT

TGGAAGAGTTCAGGGATACTTCGACCTCGAGTCGATGAGACACTGGGTGTCACTATATTT

CTTCGGATAGATTCGATGAGGTACTGGGTACCAACTTTCTTCGGCTTTGCCGATGAGACA

CTGGGTGTCAACTATTGCTTCGAACTATCCGATGAGGCACTGGGTGCCATTCTGGTGTGT

TTGGTTGGATCCGTGTATTCGCCAAAGTCCGAGTTTTGTTAATAGGGTAAATGATGAAAT

GATAAACCGAACGAGTTGGTCAAACGAGCTATTGAAATGATATGAAAAAGTTGAATTGTG

AATTGAAATGTGAAATGAGATTGAGAAATGAACCTAAGGTTCGTGAATTATTCAAACTCA

AATTGTGGATATACGATATTGGTTGATGAATTGCTATTGTTGAAATATTTAATTTAAATT

GTATATACGATTTATGCTTTACATGTACATTATTGTTATAATTTGAATTATGGTAATACC

ACTGAGTATGAATTACTCAGCGTACGGTTGTTTCCGTGCGCAGGTCAATAGAAGTCAAAG

GTCTCGGTTCAGCATCCAGATTAATCCCGGCTTCGGCAAAACTTGGTGATGTATTTTTCC

TTTGGTAAAGGTGGCATGTACATAGATTGTGTATAAAGGTTATTATGTTTTATTATATAA

TGGTTAAAAATGTTAGTATTAAAAGTTTATGGATTTTAATGAAAGAAGTCTATCTATTTT

ATCTAATTAGTACATTGTTAAATTTTAAATTGGTATTAGATTGAGTTTGATTAGAAGTAT

TTAGAATAGAAAATGTGAATGTGAAATGAATTGGTTGAATTGATGATATTTGGGAACTAT

ATGGTTTTAATTTGC

>Deltapine11

ATGTAATGACCCAAAATTCATGGGCATCGGAAAAGTATAATATCGGGCCTCCGTCCTAGT

AAATTGAGTCCGAAAATAATTATTAGAAATATTTACGAGACTAGTAGTGTGTTTAATTAG

GTTTTAATTAAGTAAATTTAGCTTAATTTAGAGTAATTAGTAAAAAGGATTAAATTGAAT

AAGAGTAAAAGTTTAATTATAGATTAAAGGAAAATAATAGGGACCAAATGGGCAATTAAG

CCACATTTGGAAGTTGAGGCGGCATAACATTGTAAAAATCTTAGATTTTTATATTATTAT

TTATATAAATATATAAATTAATTATAAAGTATATTATTAAATTAATTATATTATAAATAT

TATATTATTATATATAAAAGAAACAAAACAGAAAAGAAACAGAATAGAAAGAACAAAGAA

ACAGAATAGAAGAGACGAAACAGGGGAGAAGCAGGGGAGAAAGAAGAAAAAGAAGAAAAA

AGGGGAAATAGGGTTTTTGAAGCTTGAAATTTAAATTGGTAAGTCAAATTAGCCATTTTC

TCTTAATTCTAATGTTTTAAAAGCTTTAAAACAAAGTTTTGATGGAATTAAGTTGATATT

TTGTAAGTTCATAGGTTTTCAAGTATAGTTTATGTTGAACAAAAGAGATGAATTAGGGAT

TAACTTGAAGGAATTTTAAGTTAGAATTGAAAAAGGGATTAAATTGTAAAAGAAACTATA

AGTTTTTTTTGTTTTAGGGACTAGATTGAGGAAAATTCGGAATTAAGAAAATATGTTAAA

AATTTAATAGTTAAATTTGAGTTTAAATGAAATTTGAATAGGAATAAGGTGTGAATTGGT

GTTATAAATTTGGTTATTAACATTTTTAATCAAAACAGTTTTGGGAAGTAGCAATGGTCT

GACTTTGAAAATTCACTAAAAATTTTATAAATTGAACTAGAGGATGAACAAAATATGGAA

TTAAAGCTTATTGAGTCTAGTTTCTTATAGTAGAAACAATGTAAGCAATTAATTGATGAA

TCAAGAGATATTTGAAATTTTGTAATACTGGTTCGGGGTGATTTCGAGATGCCCTGTTTT

AACTTTGGAAAATCATTAAAAATTGTACAAAAATTATTATGGAGTGTAATTTATATATGT

GAACTCCTTAATGAATCTAGTTTCAAAATAAATAAACAAGAACCTTATTCGAGTTCTGTA

CAATGAGATAATTTAGTTTTAGTGGAGAGAGGTCAGAACTGTCAAATGAAATAACAGGGG

AGTATTTAACGAATAAACTGTATTAAATGGCTAGACCAAAAATTCTGGAAATTTTATGAT

TAGAAGATATATGAGTCTAGTTTTAAGGAAAATTTACGGATATTAATTTGGAGTTTCGTA

GCTCAAGATATAAATAATTTAGTAACAATGACCCAAGTAGACAGCTTAATGGTGAAATTA

TATAAATACATTAAAAATGGTTAAATTTGCATGTTTAGGCTCATGAATTAAATTGAATCA

TGTTGTATTGATTATTATAAATTATTATTTTCGTAGCCAACAAAGAACCTAAAGCATCAG

CATCGAAAGGAAAGGAGAAAGTCATCGAGGAGTAAACTCGAGAAAATTACGGTTTGTATT

ACTATAATTCAAGTTATTTATTATTAAATGTTAAATTTTAATTTATGTGTCTAGTAAATG

AAATGTGAGGTAAGTATTATTATTATTATTATTATTATTATTATTATTATTATTATTATT

ATGAGTGGGAATTAAATTGAATAGTTGATATGAAATAATATTTGAATTGTTTGTTGATTG

AAAGCGGGAAATGAATTTAAATCGAATAGTGACCGATATTAAATTGAATGGAAATGTATT

GAGTTGTGAAAATATGTTAATTGCGGATTAATTATTGATTGAAAGGTGGAAAAATGATTG

AATTGAAAGTGTGAGAAAGTGTGATTGAATTGGGATTATATGTGATTTAAATACCCTATT

AACTAGTCGGGCTGAGTCGGATATAGTTGGCATGCCATAGGATTGGAAGAGTTCAGGGAT

ACTTCGACCTCGAGTCGATGAGACACTGGGTGATTTCTTCGGATAGATTGGATGAGGTAC

TGGGTACCAACTTTCTTCGGCTTTGCCGATGAGACACTGGGTGTCAACTATTGCTTCGAA

CTATCCGATGAGGCACTGGGTGCCATTCTGGTGTGTTTGGTTGGATCCGTGTATCCGCCA

AAGTCCGAGTTTTGTTAATAGGGTAAATGATGAAATGATAAACCGAACGAGTTGGTCAAA

CGAGCTATTGAAATGATATGAAAAAGTTGAATTGTGAATTGAAATGTGAAATGAGATTGA

GAAATGAACCTAAGGTTCGTGAATTATTCAAACTCAAATTGTGGATATACGATATTGGTT

GATGAATTGCTATTGTTGAAATATTTAATTTAAATTGTATATACGATTTATGCTTTACAT

GTACATTATTGTTATAATTTGAATTATGGTAATACCACTGAGTATGAATTACTCAGCGTA

CGGTTGTTTCCGTGCGCAGGTCAATAGAAGTCAAAGGTCTCGGTTCAGCATCCAGATTAA

TCCCGGCTTCGGCAAAACTTGGTGATGTATTTTTCCTTTGGTAAAGGTGGCATGTACATA

GATTGTGTATAAAGGTTATTATGTTTTATTATATAATGGTTAAAAATGTTAGTATTAAAA

GTTTATGGATTTTAATGAAAGAAGTCTATCTATTTTATCTAATTAGTACATTGTTAAATT

TTAAATTGGTATTGTGTAGATTGAGTTTGATTAGAAGTATTTAGAATAGAAAATGTGAAT

GTGAAATGAATTGGTTGAATTGATGATATTTGGGAACTATATGGTTTTAATTTGCAGGGG

GTTTTATGTAAAAATAAGCAGAAATGCTGCCGAAATTTTTATAAAAAAAAATGAAGTCAT

TTGGTAAACAAATTAATAAATTTTATGAATTATTTTAATATATTGGTTATTTATTTAAGA

ATTGTTGTAAATCGTTCGATACGTCCGGTAGTGCCTCGTAATTCTGTTCCGGCGACGGTT

CGGGGTTAAGGGGTGTTACATTTTATGGTATCAGAGCTATCAGGTTTAGCCGATTCTCGG

CCTAAATCGAGCTCGGAATTGAGTCTAGATGTACATGCCACTGTCGAGTTAAACTGAGTC

GGGATTTTTGGATGCTGACCTATTTGTTTGTTTTGTTTTATAGATTAAAGATGTCTGAAG

AAAGAATAAATGATACTGATGAAAGAATGTATAGTGAAGATAGAGAATTAGATGAAACAG

AATCTGTTGCACCGAGTGTGAATCCGTTAGGCAACCAACCTTCTAATGTAGAACGAGAAA

ATGTCAGAGATAGAGATGAATCCCAATTACTGAGAATTATAGCTGATGCATTACAAAGAG

TAGCAGGAACTACTCCTGTTACGACTTCAGTACCTACTGTTAGACGGGCTCCGATAAAGG

AACTGAGGAAATATGGTGCCACTGAATTTATGGGTCTAAAAGGAGTTGATCCATCCATAG

CTGAAAATTGGATGGAGTCGACTAAAAGAATTTTGCAGCAATTGGATTGTACCCCCCGAG

AGTGTTTAATCTGTGCCGTATCGTTATTACAAGGGGAGGCTTATCTATGGTGGGAATCAG

TGGTTCGACATTTACCAGAGAGTCAGATAACGTGGGATCTATTTCAGAAGGAGTTTCAAA

AGAAATATATCGGAGAGATGTATATTGAAGACAAGAAACAAGAGTTTTTGTTGCTACAAC

AGGGTGATATGTCAGTAATAGATTATGAGAGGGAATTCTCGAGACTCAGTAGATATGCCT

CCGAGTTTATTCCGACAGAAGCCGATAGTTGTAAAAGATTTTTACGGGGTTTACGAGACG

AGATCAAAGTGCAGCTAGTATCCCATCGGATCACTGAGTTAGTAGATTTGATTGAACGAG

CTAAAATGGTGGAACAAGTTCTGGGCCTCGACAAAAAGACTGAAGTTGTTAGACCAACCG

GGAAGCGTACAGGAACTACCAGTTCGAATCCTCAGCCGAAAAGACCAAAGGAATTCCAAA

GTGGTTGGAGATCCAGTTTCAGGTCAGACAGAGGTGGTAGAAATAGGGGAAAACAGACGA

TGACATCTACTGGCAGTGTGAAAGGTCCTTCCCGAGAAATAGATATTCCAGACTGCCAAC

ACTGCGGAAAGAAACACAGAGGGGAATGTTGGAAATTAACTAGAGGCTGTTTTCGATGTG

GTTCTACAGACCATTTCATCAGAGACTGTCCGAAAGTTGATAGTACTGTACCCGTGACAT

CACAGAGATCGGTATCTACAGCTAGAGGCAGAGGGTTAGGAAGAGGTGGTTCGGTTTCAA

GGGGAGGAAGTATTAGGAGAAGCAATGATATTGCTACTCAGCAGTCTGAGGCTAAAGTAC

CTGCCAGAGCTTATGTGGTCAGAACACAGGAAGAAGGTGACGCCCACGATGTAGTAACAG

GTATATTCTTACTATATTCTGAGCCTGTTTATGCTTTAATTGATCCCGGATCTTCACATT

CTTATATAAATTCAAAATTAGTTGAATTGGGAAAATTTAATTCTGAAATATCTAGAGTGA

CTGTAGAAGTGTCGAGTCCGTTGGGGCAAACAGTATTAGTGAATCAGATCTGTCCGAGAT

GCCCGTTAATTATACAAAATAAAACTTTTCCTATTGACCTGTTGATTATGCCATTTGGAG

ATTTTGATATAATACTGGGGATGGATTGGTTGGCTGAGCACGGAGTGGTATTGGATTGTT

ATAAAAAGAAGTTTAGTATTCAGACAGAAGACGGGGACAGAATTGAAGTAAATGGTATCC

GTACTAATGGGCCGACACGTATTATTTCGGCAATAAAGGCTAATAAATTGCTTCAGCGGG

GTTGTACAGCGTATTTAGCCTATGTTATTAATTCTGATTTGGTTGGTAGTCAGTGCAGTA

AGATTAGAACCGTATGTGAGTTTCCAGATGTATTTCCTGAAGAGCTACCGGGTTTACCAC

CTGACAGAGAGGTTGAATTTGCTATAGAAGTGTATCCGGGTACAGCACCAATCTCTATAC

CACCGTATCGAATGTCACCCACTGAGTTGAAAGAGTTGAAAGTGCAGTTACAGGACTTGT

CAGATCGTGGATTTATTAGACCGAGCATCTCACCTTGGGGAGCTCCAGTATTGTTTGTTA

AAAAGAAAGATGGATCGATGCGGCTTTGTATTGATTACCGGCAGTTAAACAAAGTGACGA

TCAAGAACCGGTATCCGTTACCCCGTATAGATGATTTATTTGATCAACTAAAAGGAGCTT

CAGTATTTTCAAAGATTGACTTAAGATCTGGGTATTATCAGCTGAAGGTAAAAGAAAGTG

ATGTTCCGAAGACTGCATTTCGTACTCGATATGGTCATTATGAATTTTTGGTGATGCCGT

TCGGGTTGACTAATGCTCCAGCTGCTTTTATGGATCTGATGAATCGTATTTTTCAGCCGT

ATTTAGATCAGTTTGTGGTGGTTTTTATTGATGACATCTTGGTTTATTCGAAGTCAGAGT

CAGAGCATGATCAGCATCTCAGAACCGTGCTACAAATTCTGCGAGAAAAACAGTTGTACG

GGAAACTAAGTAAATGTGAATTCTGGTTATCAGAGGTAGTATTCTTGGGACATGTTGTAT

CTGCGGATGGGATTAGAGTTGATCCGAAGAAGATCGAGGCAATTGTTCAATGGAAGGCAC

CAAAGAATGTATCAGAGGTACGCAGTTTTCTTGGTTTGGCTGGGTATTACAGAAGATTTG

TAAATGGGTTTTCGAAGATAGCTTTGCCGATGACCAAATTACTACAGAAGAATGTTCCAT

TTATCTGGGATGATCAGTGTCAGAGGAGCTTTGAAACATTGAAACAGATGTTGACAGAGG

CACCAGTTTTAACTTTACCAGAATCAGGGAAAGATTTCATAGTGTACAGTGATGCTTCTT

TGAATGGTTTGGGTTGTGTATTGATGCAAGAAGGAAAAGTAATAGCTTATGCATCTCGAC

AGTTGAAGTCACATGAACGCAACTACCCGACACACGATTTAGAGTTAGCTGCTGTAATCT

TTGCATTGAAGATTTGGATACATTACTTGTATGGTGAGAAATGTTATATTTACACTGATC

ATAAAAGTCTAAAATATCTTCTGTCACAAAAGGAGTTGAATCTGAGACAGAGACGGTGGA

TTGAACTTCTGAAAGATTATGATTGTGTTATAGATTATCATCCAGGGAAGGCAAATGTGG

TAGCAGATGCATTGAGTAGAAAAGCAGCGATTGAATTACGAGCAATGTTCGCTCGACTTA

GTATTAAGGATGATGGAAGTTTGTTAGCTGAGTTAAGAGTCAAGCCGGTGATGTTTGATC

AAATCAGAGCAGCACAGTTAAAAGATGAAAAGTTGATGAGGAAAAGAGAAATGGTACAGT

ATGGTGCGGTAGAAAATTTTAGTATTGACGAGCATGATTGTTTGAGATTTCGAAATCGAA

TTTGTGTTCCATCTACTTCTGAGATTAAAGAATTGATTCTCCGAGAAGCACATAATAGTA

TTTTTGCTTTGCACCCAGGAGGAACGAAGATGTATCGTGATCTACGAGAACTGTATTGGT

GGCCAGGAATGAAGAAAGATATAGTTGAATATGTCAGTAAATGCTTGACTTGTCAGCGGG

TAAAAGCAGAACATCAGGTACCAACAGGCCTGTTACAGCCTATTACTATTCCCGAGTGGA

AATGGGATCGCATTACCATGGATTTTGTTACGGGGTTGCCATTGTCAGTGAGTAAAAAGA

ATGCTATTTGGGTGATTGTTGATCGACTCACAAAATCAGCTCATTTTATAGCAGTTAGAA

CCGACTGGTCATTACAGAAGCTTGCCGAGGTTTATATTCGAGAAATTGTTAGATTACATG

GTATTCCGGTATCAATAATTTCAGACAGAGATCCTCGATTCACTTCGAGATTTTGGAAGC

AGCTGCATGAATCATTGGGTACTCGACTTAGTTTCAGTACAGCTTTTCATCCTCAAACTG

ATGGACAATCTGAACGAGTAATTCAGATATTAGAAGATATGCTTCGAGCTTGTGTCATTG

ATTTTGAATCAGGTTGGGAACGTTATTTACCATTGGCCGAGTTTGTTTATAATAATAGTT

TCCAATCTAGTATTCAAATGGCTCCATATGAAGCACTTTATGGTCGAAGGTGTCGATCAC

CAATATGTTGGACAAAATTAAGAGAAAGAAAAGTGATTGGGCCGGAATTGATTCAAGAGA

CAGAAGAAACAGTTAAAAAGATTAAAGATAGACTGAAAGCCGCTTTCGACAGACAGAAAT

CTTACGCAGACTTGAAACGACGAGACATTGAATATTCCGTTGGTGATAAGGTATTCCTCA

AAGTATCGCCGTGGAAGAAAATTTTGAGATTTGGTCGGAAGGGAAAATTAAGTCCGCGCT

TTATTGGGCCGTATGAGATAGTGGAAAGAATTGGGCCTGTTGCTTATCGATTATCCTTAC

CTCCAGAGTTACAGAAAATTCATGATGTTTTTCATGTTTCGATGCTTCGGAGATATAGAT

CGGATCCTTCTCATGTTATTCCCACTGAAGACATTGAACTTCGATCTGATTTAACTTATG

AAGAAGAACCAGTTCAAATATTAGCACGAGAAGTGAAAGAATTAAGAAATAAACGGGTTC

CTTTAGTACAAGTTTTATGGAGAAGCCATAGTGTGGAAGAAGCAACTTGGGAACCGGAAG

AGACAATGAGAGCACAATATCCTCATCTCTTCTCAGGTAAATTTCGAGGACGAAATTTAT

TAAGAGGGGGAGAAATGTAATGACCTAAAATTCATGGGCATCGGAAAAGTATAATATTGG

GCCTCCGTCCTAGTAAATTGAGTCCGAAAATAATTATTAGAAATATTTACGAGACTAGTA

GTGTGTTTAATTAGGTTTTAATTAAGTAAATTTAGCTTAATTTAGAGTAATTAGTAAAAA

GGATTAAATTGAATAAGAGTAAAAGTTTAATTATAGATTAAAGGAAAATAATAGGGACCA

AATGGGCAATTAAGCCACATTTGGAAGTTGAGGCGGCATAACATTGTAAAAATCTTAGAT

TTTTATATTATTATTTATATAAATATATAAATTAATTATAAAGTATATTATTAAATTAAT

TATATTATAAATATTATATTATTATATATAAAAGAAACAAAACAGAAAAGAAACAGAATA

GAAAGAACAAAGAAACAGAATAGAAGAGACGAAACAGGGGAGAAGCAGGGGAGAAAGAAG

AAAAAGAAGAAAAAAGGGGAAATAGGGTTTTTGAAGCTTGAAATTTAAATTGGTAAGTCA

AATTAGCCATTTTCTCTTAATTCTAATGTTTTAAAAGCTTTAAAACAAAGTTTTGATGGA

ATTAAGTTGATATTTTGTAAGTTCATAGGTTTTCAAGTATAGTTTATGTTGAACAAAAGA

GATGAATTAGGGATTAACTTGAAGGAATTTTAAGTTAGAATTGAAAAAGGGATTAAATTG

TAAAAGAAACTATAAGTTTTTTTTGTTTTAGGGACTAGATTGAGGAAAATTCGGAATTAA

GAAAATATGTTAAAAATTTAATAGTTAAATTTGAGTTTAAATGAAATTTGAATAGGAATA

AGGTGTGAATTGGTGTTATAAATTTGGTTATTAACATTTTTAATCAAAACAGTTTTGGGA

AGTAGCAATGGTCTGACTTTGAAAATTCACTAAAAATTTTATAAATTGAACTAGAGGATG

AACAAAATATGGAATTAAAGCTTATTGAGTCTAGTTTCTTATAGTAGAAACAATGTAAGC

AATTAATTGATGAATCAAGAGATATTTGAAATTTTGTAATACTGGTTCGGGGTGATTTCG

AGATGCCCTGTTTTAACTTTGGAAAATCATTAAAAATTGTACAAAAATTATTATGGAGTG

TAATTTATATATGTAAACTCCTTAATGAATCTAGTTTCAAAATAAATAAACAAGAACCTT

ATTCGAGTTCTGTACAATGAGATAATTTAGTTTTAGTGGAGAGAGGTCAGAACTGTCAAA

TGAAATAACAGGGGAGTATTTAACGAATAAACTGTATTAAATGGCTAGACCAAAAATTCT

GGAAATTTTATGATTAGAAGATATATGAGTCTAGTTTTAAGGAAAATTTACGGATATTAA

TTTGGAGTTTCGTAGCTCAAGATATAAATAATTTAGTAACAATGACCCAAGTAGACAGCT

TAATGGTGAAATTATATAAATACATTAAAAATGGTTAAATTTGCATGTTTAGGCTCATGA

ATTAAATTGAATCATGTTGTATTGATTATTATAAATTATTATTTTCGTAGCCAACAAAGA

ACCTAAAGCATCAGCATCGAAAGGAAAGGAGAAAGTCATCGAGGAGTAAACTCGAGAAAA

TTACGGTTTGTATTACTATAATTCAAGTTATTTATTATTAAATGTTAAATTTTAATTTAT

GTGTCTAGTAAATGAAATGTGAGGTAAGTATTATTATTATTATTATTATTATTATTATTA

TTATTATTATTATTATTATGAGTGGGAATTAAATTGAATAGTTGATATGAAATAATATTT

GAATTGTTTGTTGATTGAAAGCGGGAAATGAATTTAAATCGAATAGTGACCGATATTAAA

TTGAATGGAAATGTATTGAGTTGTGAAAATATGTTAATTGCGGATTAATTATTGATTGAA

AGGTGGAAAAATGATTGAATTGAAAGTGTGAGAAAGTGTGATTGAATTGGGATTATATGT

GATTTAAATACCCTATTAACTAGTCGGGCTGAGTCGGATATAGTTGGCATGCCATAGGAT

TGGAAGAGTTCAGGGATACTTCGACCTCGAGTCGATGAGACACTGGGTGTCACTATATTT

CTTCGGATAGATTCGATGAGGTACTGGGTACCAACTTTCTTCGGCTTTGCCGATGAGACA

CTGGGTGTCAACTATTGCTTCGAACTATCCGATGAGGCACTGGGTGCCATTCTGGTGTGT

TTGGTTGGATCCGTGTATTCGCCAAAGTCCGAGTTTTGTTAATAGGGTAAATGATGAAAT

GATAAACCGAACGAGTTGGTCAAACGAGCTATTGAAATGATATGAAAAAGTTGAATTGTG

AATTGAAATGTGAAATGAGATTGAGAAATGAACCTAAGGTTCGTGAATTATTCAAACTCA

AATTGTGGATATACGATATTGGTTGATGAATTGCTATTGTTGAAATATTTAATTTAAATT

GTATATACGATTTATGCTTTACATGTACATTATTGTTATAATTTGAATTATGGTAATACC

ACTGAGTATGAATTACTCAGCGTACGGTTGTTTCCGTGCGCAGGTCAATAGAAGTCAAAG

GTCTCGGTTCAGCATCCAGATTAATCCCGGCTTCGGCAAAACTTGGTGATGTATTTTTCC

TTTGGTAAAGGTGGCATGTACATAGATTGTGTATAAAGGTTATTATGTTTTATTATATAA

TGGTTAAAAATGTTAGTATTAAAAGTTTATGGATTTTAATGAAAGAAGTCTATCTATTTT

ATCTAATTAGTACATTGTTAAATTTTAAATTGGTATTAGATTGAGTTTGATTAGAAGTAT

TTAGAATAGAAAATGTGAATGTGAAATGAATTGGTTGAATTGATGATATTTGGGAACTAT

ATGGTTTTAATTTGC

>Deltapine12

ATGTAATGACCCAAAATTCATGGGCATCGGAAAAGTATAATATCGGGCCTCCGTCCTAGT

AAATTGAGTCCGAAAATAATTATTAGAAATATTTACGAGACTAGTAGTGTGTTTAATTAG

GTTTTAATTAAGTAAATTTAGCTTAATTTAGAGTAATTAGTAAAAAGGATTAAATTGAAT

AAGAGTAAAAGTTTAATTATAGATTAAAGGAAAATAATAGGGACCAAATGGGCAATTAAG

CCACATTTGGAAGTTGAGGCGGCATAACATTGTAAAAATCTTAGATTTTTATATTATTAT

TTATATAAATATATAAATTAATTATAAAGTATATTATTAAATTAATTATATTATAAATAT

TATATTATTATATATAAAAGAAACAAAACAGAAAAGAAACAGAATAGAAAGAACAAAGAA

ACAGAATAGAAGAGACGAAACAGGGGAGAAGCAGGGGAGAAAGAAGAAAAAGAAGAAAAA

AGGGGAAATAGGGTTTTTGAAGCTTGAAATTTAAATTGGTAAGTCAAATTAGCCATTTTC

TCTTAATTCTAATGTTTTAAAAGCTTTAAAACAAAGTTTTGATGGAATTAAGTTGATATT

TTGTAAGTTCATAGGTTTTCAAGTATAGTTTATGTTGAACAAAAGAGATGAATTAGGGAT

TAACTTGAAGGAATTTTAAGTTAGAATTGAAAAAGGGATTAAATTGTAAAAGAAACTATA

AGTTTTTTTTGTTTTAGGGACTAGATTGAGGAAAATTCGGAATTAAGAAAATATGTTAAA

AATTTAATAGTTAAATTTGAGTTTAAATGAAATTTGAATAGGAATAAGGTGTGAATTGGT

GTTATAAATTTGGTTATTAACATTTTTAATCAAAACAGTTTTGGGAAGTAGCAATGGTCT

GACTTTGAAAATTCACTAAAAATTTTATAAATTGAACTAGAGGATGAACAAAATATGGAA

TTAAAGCTTATTGAGTCTAGTTTCTTATAGTAGAAACAATGTAAGCAATTAATTGATGAA

TCAAGAGATATTTGAAATTTTGTAATACTGGTTCGGGGTGATTTCGAGATGCCCTGTTTT

AACTTTGGAAAATCATTAAAAATTGTACAAAAATTATTATGGAGTGTAATTTATATATGT

GAACTCCTTAATGAATCTAGTTTCAAAATAAATAAACAAGAACCTTATTCGAGTTCTGTA

CAATGAGATAATTTAGTTTTAGTGGAGAGAGGTCAGAACTGTCAAATGAAATAACAGGGG

AGTATTTAACGAATAAACTGTATTAAATGGCTAGACCAAAAATTCTGGAAATTTTATGAT

TAGAAGATATATGAGTCTAGTTTTAAGGAAAATTTACGGATATTAATTTGGAGTTTCGTA

GCTCAAGATATAAATAATTTAGTAACAATGACCCAAGTAGACAGCTTAATGGTGAAATTA

TATAAATACATTAAAAATGGTTAAATTTGCATGTTTAGGCTCATGAATTAAATTGAATCA

TGTTGTATTGATTATTATAAATTATTATTTTCGTAGCCAACAAAGAACCTAAAGCATCAG

CATCGAAAGGAAAGGAGAAAGTCATCGAGGAGTAAACTCGAGAAAATTACGGTTTGTATT

ACTATAATTCAAGTTATTTATTATTAAATGTTAAATTTTAATTTATGTGTCTAGTAAATG

AAATGTGAGGTAAGTATTATTATTATTATTATTATTATTATTATTATTATTATTATTATT

ATGAGTGGGAATTAAATTGAATAGTTGATATGAAATAATATTTGAATTGTTTGTTGATTG

AAAGCGGGAAATGAATTTAAATCGAATAGTGACCGATATTAAATTGAATGGAAATGTATT

GAGTTGTGAAAATATGTTAATTGCGGATTAATTATTGATTGAAAGGTGGAAAAATGATTG

AATTGAAAGTGTGAGAAAGTGTGATTGAATTGGGATTATATGTGATTTAAATACCCTATT

AACTAGTCGGGCTGAGTCGGATATAGTTGGCATGCCATAGGATTGGAAGAGTTCAGGGAT

ACTTCGACCTCGAGTCGATGAGACACTGGGTGATTTCTTCGGATAGATTGGATGAGGTAC

TGGGTACCAACTTTCTTCGGCTTTGCCGATGAGACACTGGGTGTCAACTATTGCTTCGAA

CTATCCGATGAGGCACTGGGTGCCATTCTGGTGTGTTTGGTTGGATCCGTGTATCCGCCA

AAGTCCGAGTTTTGTTAATAGGGTAAATGATGAAATGATAAACCGAACGAGTTGGTCAAA

CGAGCTATTGAAATGATATGAAAAAGTTGAATTGTGAATTGAAATGTGAAATGAGATTGA

GAAATGAACCTAAGGTTCGTGAATTATTCAAACTCAAATTGTGGATATACGATATTGGTT

GATGAATTGCTATTGTTGAAATATTTAATTTAAATTGTATATACGATTTATGCTTTACAT

GTACATTATTGTTATAATTTGAATTATGGTAATACCACTGAGTATGAATTACTCAGCGTA

CGGTTGTTTCCGTGCGCAGGTCAATAGAAGTCAAAGGTCTCGGTTCAGCATCCAGATTAA

TCCCGGCTTCGGCAAAACTTGGTGATGTATTTTTCCTTTGGTAAAGGTGGCATGTACATA

GATTGTGTATAAAGGTTATTATGTTTTATTATATAATGGTTAAAAATGTTAGTATTAAAA

GTTTATGGATTTTAATGAAAGAAGTCTATCTATTTTATCTAATTAGTACATTGTTAAATT

TTAAATTGGTATTGTGTAGATTGAGTTTGATTAGAAGTATTTAGAATAGAAAATGTGAAT

GTGAAATGAATTGGTTGAATTGATGATATTTGGGAACTATATGGTTTTAATTTGCAGGGG

GTTTTATGTAAAAATAAGCAGAAATGCTGCCGAAATTTTTATAAAAAAAAATGAAGTCAT

TTGGTAAACAAATTAATAAATTTTATGAATTATTTTAATATATTGGTTATTTATTTAAGA

ATTGTTGTAAATCGTTCGATACGTCCGGTAGTGCCTCGTAATTCTGTTCCGGCGACGGTT

CGGGGTTAAGGGGTGTTACATTTTATGGTATCAGAGCTATCAGGTTTAGCCGATTCTCGG

CCTAAATCGAGCTCGGAATTGAGTCTAGATGTACATGCCACTGTCGAGTTAAACTGAGTC

GGGATTTTTGGATGCTGACCTATTTGTTTGTTTTGTTTTATAGATTAAAGATGTCTGAAG

AAAGAATAAATGATACTGATGAAAGAATGTATAGTGAAGATAGAGAATTAGATGAAACAG

AATCTGTTGCACCGAGTGTGAATCCGTTAGGCAACCAACCTTCTAATGTAGAACGAGAAA

ATGTCAGAGATAGAGATGAATCCCAATTACTGAGAATTATAGCTGATGCATTACAAAGAG

TAGCAGGAACTACTCCTGTTACGACTTCAGTACCTACTGTTAGACGGGCTCCGATAAAGG

AACTGAGGAAATATGGTGCCACTGAATTTATGGGTCTAAAAGGAGTTGATCCATCCATAG

CTGAAAATTGGATGGAGTCGACTAAAAGAATTTTGCAGCAATTGGATTGTACCCCCCGAG

AGTGTTTAATCTGTGCCGTATCGTTATTACAAGGGGAGGCTTATCTATGGTGGGAATCAG

TGGTTCGACATTTACCAGAGAGTCAGATAACGTGGGATCTATTTCAGAAGGAGTTTCAAA

AGAAATATATCGGAGAGATGTATATTGAAGACAAGAAACAAGAGTTTTTGTTGCTACAAC

AGGGTGATATGTCAGTAATAGATTATGAGAGGGAATTCTCGAGACTCAGTAGATATGCCT

CCGAGTTTATTCCGACAGAAGCCGATAGTTGTAAAAGATTTTTACGGGGTTTACGAGACG

AGATCAAAGTGCAGCTAGTATCCCATCGGATCACTGAGTTAGTAGATTTGATTGAACGAG

CTAAAATGGTGGAACAAGTTCTGGGCCTCGACAAAAAGACTGAAGTTGTTAGACCAACCG

GGAAGCGTACAGGAACTACCAGTTCGAATCCTCAGCCGAAAAGACCAAAGGAATTCCAAA

GTGGTTGGAGATCCAGTTTCAGGTCAGACAGAGGTGGTAGAAATAGGGGAAAACAGACGA

TGACATCTACTGGCAGTGTGAAAGGTCCTTCCCGAGAAATAGATATTCCAGACTGCCAAC

ACTGCGGAAAGAAACACAGAGGGGAATGTTGGAAATTAACTAGAGGCTGTTTTCGATGTG

GTTCTACAGACCATTTCATCAGAGACTGTCCGAAAGTTGATAGTACTGTACCCGTGACAT

CACAGAGATCGGTATCTACAGCTAGAGGCAGAGGGTTAGGAAGAGGTGGTTCGGTTTCAA

GGGGAGGAAGTATTAGGAGAAGCAATGATATTGCTACTCAGCAGTCTGAGGCTAAAGTAC

CTGCCAGAGCTTATGTGGTCAGAACACAGGAAGAAGGTGACGCCCACGATGTAGTAACAG

GTATATTCTTACTATATTCTGAGCCTGTTTATGCTTTAATTGATCCCGGATCTTCACATT

CTTATATAAATTCAAAATTAGTTGAATTGGGAAAATTTAATTCTGAAATATCTAGAGTGA

CTGTAGAAGTGTCGAGTCCGTTGGGGCAAACAGTATTAGTGAATCAGATCTGTCCGAGAT

GCCCGTTAATTATACAAAATAAAACTTTTCCTATTGACCTGTTGATTATGCCATTTGGAG

ATTTTGATATAATACTGGGGATGGATTGGTTGGCTGAGCACGGAGTGGTATTGGATTGTT

ATAAAAAGAAGTTTAGTATTCAGACAGAAGACGGGGACAGAATTGAAGTAAATGGTATCC

GTACTAATGGGCCGACACGTATTATTTCGGCAATAAAGGCTAATAAATTGCTTCAGCGGG

GTTGTACAGCGTATTTAGCCTATGTTATTAATTCTGATTTGGTTGGTAGTCAGTGCAGTA

AGATTAGAACCGTATGTGAGTTTCCAGATGTATTTCCTGAAGAGCTACCGGGTTTACCAC

CTGACAGAGAGGTTGAATTTGCTATAGAAGTGTATCCGGGTACAGCACCAATCTCTATAC

CACCGTATCGAATGTCACCCACTGAGTTGAAAGAGTTGAAAGTGCAGTTACAGGACTTGT

CAGATCGTGGATTTATTAGACCGAGCATCTCACCTTGGGGAGCTCCAGTATTGTTTGTTA

AAAAGAAAGATGGATCGATGCGGCTTTGTATTGATTACCGGCAGTTAAACAAAGTGACGA

TCAAGAACCGGTATCCGTTACCCCGTATAGATGATTTATTTGATCAACTAAAAGGAGCTT

CAGTATTTTCAAAGATTGACTTAAGATCTGGGTATTATCAGCTGAAGGTAAAAGAAAGTG

ATGTTCCGAAGACTGCATTTCGTACTCGATATGGTCATTATGAATTTTTGGTGATGCCGT

TCGGGTTGACTAATGCTCCAGCTGCTTTTATGGATCTGATGAATCGTATTTTTCAGCCGT

ATTTAGATCAGTTTGTGGTGGTTTTTATTGATGACATCTTGGTTTATTCGAAGTCAGAGT

CAGAGCATGATCAGCATCTCAGAACCGTGCTACAAATTCTGCGAGAAAAACAGTTGTACG

GGAAACTAAGTAAATGTGAATTCTGGTTATCAGAGGTAGTATTCTTGGGACATGTTGTAT

CTGCGGATGGGATTAGAGTTGATCCGAAGAAGATCGAGGCAATTGTTCAATGGAAGGCAC

CAAAGAATGTATCAGAGGTACGCAGTTTTCTTGGTTTGGCTGGGTATTACAGAAGATTTG

TAAATGGGTTTTCGAAGATAGCTTTGCCGATGACCAAATTACTACAGAAGAATGTTCCAT

TTATCTGGGATGATCAGTGTCAGAGGAGCTTTGAAACATTGAAACAGATGTTGACAGAGG

CACCAGTTTTAACTTTACCAGAATCAGGGAAAGATTTCATAGTGTACAGTGATGCTTCTT

TGAATGGTTTGGGTTGTGTATTGATGCAAGAAGGAAAAGTAATAGCTTATGCATCTCGAC

AGTTGAAGTCACATGAACGCAACTACCCGACACACGATTTAGAGTTAGCTGCTGTAATCT

TTGCATTGAAGATTTGGATACATTACTTGTATGGTGAGAAATGTTATATTTACACTGATC

ATAAAAGTCTAAAATATCTTCTGTCACAAAAGGAGTTGAATCTGAGACAGAGACGGTGGA

TTGAACTTCTGAAAGATTATGATTGTGTTATAGATTATCATCCAGGGAAGGCAAATGTGG

TAGCAGATGCATTGAGTAGAAAAGCAGCGATTGAATTACGAGCAATGTTCGCTCGACTTA

GTATTAAGGATGATGGAAGTTTGTTAGCTGAGTTAAGAGTCAAGCCGGTGATGTTTGATC

AAATCAGAGCAGCACAGTTAAAAGATGAAAAGTTGATGAGGAAAAGAGAAATGGTACAGT

ATGGTGCGGTAGAAAATTTTAGTATTGACGAGCATGATTGTTTGAGATTTCGAAATCGAA

TTTGTGTTCCATCTACTTCTGAGATTAAAGAATTGATTCTCCGAGAAGCACATAATAGTA

TTTTTGCTTTGCACCCAGGAGGAACGAAGATGTATCGTGATCTACGAGAACTGTATTGGT

GGCCAGGAATGAAGAAAGATATAGTTGAATATGTCAGTAAATGCTTGACTTGTCAGCGGG

TAAAAGCAGAACATCAGGTACCAACAGGCCTGTTACAGCCTATTACTATTCCCGAGTGGA

AATGGGATCGCATTACCATGGATTTTGTTACGGGGTTGCCATTGTCAGTGAGTAAAAAGA

ATGCTATTTGGGTGATTGTTGATCGACTCACAAAATCAGCTCATTTTATAGCAGTTAGAA

CCGACTGGTCATTACAGAAGCTTGCCGAGGTTTATATTCGAGAAATTGTTAGATTACATG

GTATTCCGGTATCAATAATTTCAGACAGAGATCCTCGATTCACTTCGAGATTTTGGAAGC

AGCTGCATGAATCATTGGGTACTCGACTTAGTTTCAGTACAGCTTTTCATCCTCAAACTG

ATGGACAATCTGAACGAGTAATTCAGATATTAGAAGATATGCTTCGAGCTTGTGTCATTG

ATTTTGAATCAGGTTGGGAACGTTATTTACCATTGGCCGAGTTTGTTTATAATAATAGTT

TCCAATCTAGTATTCAAATGGCTCCATATGAAGCACTTTATGGTCGAAGGTGTCGATCAC

CAATATGTTGGACAAAATTAAGAGAAAGAAAAGTGATTGGGCCGGAATTGATTCAAGAGA

CAGAAGAAACAGTTAAAAAGATTAAAGATAGACTGAAAGCCGCTTTCGACAGACAGAAAT

CTTACGCAGACTTGAAACGACGAGACATTGAATATTCCGTTGGTGATAAGGTATTCCTCA

AAGTATCGCCGTGGAAGAAAATTTTGAGATTTGGTCGGAAGGGAAAATTAAGTCCGCGCT

TTATTGGGCCGTATGAGATAGTGGAAAGAATTGGGCCTGTTGCTTATCGATTATCCTTAC

CTCCAGAGTTACAGAAAATTCATGATGTTTTTCATGTTTCGATGCTTCGGAGATATAGAT

CGGATCCTTCTCATGTTATTCCCACTGAAGACATTGAACTTCGATCTGATTTAACTTATG

AAGAAGAACCAGTTCAAATATTAGCACGAGAAGTGAAAGAATTAAGAAATAAACGGGTTC

CTTTAGTACAAGTTTTATGGAGAAGCCATAGTGTGGAAGAAGCAACTTGGGAACCGGAAG

AGACAATGAGAGCACAATATCCTCATCTCTTCTCAGGTAAATTTCGAGGACGAAATTTAT

TAAGAGGGGGAGAAATGTAATGACCTAAAATTCATGGGCATCGGAAAAGTATAATATTGG

GCCTCCGTCCTAGTAAATTGAGTCCGAAAATAATTATTAGAAATATTTACGAGACTAGTA

GTGTGTTTAATTAGGTTTTAATTAAGTAAATTTAGCTTAATTTAGAGTAATTAGTAAAAA

GGATTAAATTGAATAAGAGTAAAAGTTTAATTATAGATTAAAGGAAAATAATAGGGACCA

AATGGGCAATTAAGCCACATTTGGAAGTTGAGGCGGCATAACATTGTAAAAATCTTAGAT

TTTTATATTATTATTTATATAAATATATAAATTAATTATAAAGTATATTATTAAATTAAT

TATATTATAAATATTATATTATTATATATAAAAGAAACAAAACAGAAAAGAAACAGAATA

GAAAGAACAAAGAAACAGAATAGAAGAGACGAAACAGGGGAGAAGCAGGGGAGAAAGAAG

AAAAAGAAGAAAAAAGGGGAAATAGGGTTTTTGAAGCTTGAAATTTAAATTGGTAAGTCA

AATTAGCCATTTTCTCTTAATTCTAATGTTTTAAAAGCTTTAAAACAAAGTTTTGATGGA

ATTAAGTTGATATTTTGTAAGTTCATAGGTTTTCAAGTATAGTTTATGTTGAACAAAAGA

GATGAATTAGGGATTAACTTGAAGGAATTTTAAGTTAGAATTGAAAAAGGGATTAAATTG

TAAAAGAAACTATAAGTTTTTTTTGTTTTAGGGACTAGATTGAGGAAAATTCGGAATTAA

GAAAATATGTTAAAAATTTAATAGTTAAATTTGAGTTTAAATGAAATTTGAATAGGAATA

AGGTGTGAATTGGTGTTATAAATTTGGTTATTAACATTTTTAATCAAAACAGTTTTGGGA

AGTAGCAATGGTCTGACTTTGAAAATTCACTAAAAATTTTATAAATTGAACTAGAGGATG

AACAAAATATGGAATTAAAGCTTATTGAGTCTAGTTTCTTATAGTAGAAACAATGTAAGC

AATTAATTGATGAATCAAGAGATATTTGAAATTTTGTAATACTGGTTCGGGGTGATTTCG

AGATGCCCTGTTTTAACTTTGGAAAATCATTAAAAATTGTACAAAAATTATTATGGAGTG

TAATTTATATATGTAAACTCCTTAATGAATCTAGTTTCAAAATAAATAAACAAGAACCTT

ATTCGAGTTCTGTACAATGAGATAATTTAGTTTTAGTGGAGAGAGGTCAGAACTGTCAAA

TGAAATAACAGGGGAGTATTTAACGAATAAACTGTATTAAATGGCTAGACCAAAAATTCT

GGAAATTTTATGATTAGAAGATATATGAGTCTAGTTTTAAGGAAAATTTACGGATATTAA

TTTGGAGTTTCGTAGCTCAAGATATAAATAATTTAGTAACAATGACCCAAGTAGACAGCT

TAATGGTGAAATTATATAAATACATTAAAAATGGTTAAATTTGCATGTTTAGGCTCATGA

ATTAAATTGAATCATGTTGTATTGATTATTATAAATTATTATTTTCGTAGCCAACAAAGA

ACCTAAAGCATCAGCATCGAAAGGAAAGGAGAAAGTCATCGAGGAGTAAACTCGAGAAAA

TTACGGTTTGTATTACTATAATTCAAGTTATTTATTATTAAATGTTAAATTTTAATTTAT

GTGTCTAGTAAATGAAATGTGAGGTAAGTATTATTATTATTATTATTATTATTATTATTA

TTATTATTATTATTATTATGAGTGGGAATTAAATTGAATAGTTGATATGAAATAATATTT

GAATTGTTTGTTGATTGAAAGCGGGAAATGAATTTAAATCGAATAGTGACCGATATTAAA

TTGAATGGAAATGTATTGAGTTGTGAAAATATGTTAATTGCGGATTAATTATTGATTGAA

AGGTGGAAAAATGATTGAATTGAAAGTGTGAGAAAGTGTGATTGAATTGGGATTATATGT

GATTTAAATACCCTATTAACTAGTCGGGCTGAGTCGGATATAGTTGGCATGCCATAGGAT

TGGAAGAGTTCAGGGATACTTCGACCTCGAGTCGATGAGACACTGGGTGTCACTATATTT

CTTCGGATAGATTCGATGAGGTACTGGGTACCAACTTTCTTCGGCTTTGCCGATGAGACA

CTGGGTGTCAACTATTGCTTCGAACTATCCGATGAGGCACTGGGTGCCATTCTGGTGTGT

TTGGTTGGATCCGTGTATTCGCCAAAGTCCGAGTTTTGTTAATAGGGTAAATGATGAAAT

GATAAACCGAACGAGTTGGTCAAACGAGCTATTGAAATGATATGAAAAAGTTGAATTGTG

AATTGAAATGTGAAATGAGATTGAGAAATGAACCTAAGGTTCGTGAATTATTCAAACTCA

AATTGTGGATATACGATATTGGTTGATGAATTGCTATTGTTGAAATATTTAATTTAAATT

GTATATACGATTTATGCTTTACATGTACATTATTGTTATAATTTGAATTATGGTAATACC

ACTGAGTATGAATTACTCAGCGTACGGTTGTTTCCGTGCGCAGGTCAATAGAAGTCAAAG

GTCTCGGTTCAGCATCCAGATTAATCCCGGCTTCGGCAAAACTTGGTGATGTATTTTTCC

TTTGGTAAAGGTGGCATGTACATAGATTGTGTATAAAGGTTATTATGTTTTATTATATAA

TGGTTAAAAATGTTAGTATTAAAAGTTTATGGATTTTAATGAAAGAAGTCTATCTATTTT

ATCTAATTAGTACATTGTTAAATTTTAAATTGGTATTAGATTGAGTTTGATTAGAAGTAT

TTAGAATAGAAAATGTGAATGTGAAATGAATTGGTTGAATTGATGATATTTGGGAACTAT

ATGGTTTTAATTTGC

>Deltapine16

ATGTAATGACCCAAAATTCATGGGCATCGGAAAAGTATAATATCGGGCCTCCGTCCTAGT

AAATTGAGTCCGAAAATAATTATTAGAAATATTTACGAGACTAGTAGTGTGTTTAATTAG

GTTTTAATTAAGTAAATTTAGCTTAATTTAGAGTAATTAGTAAAAAGGATTAAATTGAAT

AAGAGTAAAAGTTTAATTATAGATTAAAGGAAAATAATAGGGACCAAATGGGCAATTAAG

CCACATTTGGAAGTTGAGGCGGCATAACATTGTAAAAATCTTAGATTTTTATATTATTAT

TTATATAAATATATAAATTAATTATAAAGTATATTATTAAATTAATTATATTATAAATAT

TATATTATTATATATAAAAGAAACAAAACAGAAAAGAAACAGAATAGAAAGAACAAAGAA

ACAGAATAGAAGAGACGAAACAGGGGAGAAGCAGGGGAGAAAGAAGAAAAAGAAGAAAAA

AGGGGAAATAGGGTTTTTGAAGCTTGAAATTTAAATTGGTAAGTCAAATTAGCCATTTTC

TCTTAATTCTAATGTTTTAAAAGCTTTAAAACAAAGTTTTGATGGAATTAAGTTGATATT

TTGTAAGTTCATAGGTTTTCAAGTATAGTTTATGTTGAACAAAAGAGATGAATTAGGGAT

TAACTTGAAGGAATTTTAAGTTAGAATTGAAAAAGGGATTAAATTGTAAAAGAAACTATA

AGTTTTTTTTGTTTTAGGGACTAGATTGAGGAAAATTCGGAATTAAGAAAATATGTTAAA

AATTTAATAGTTAAATTTGAGTTTAAATGAAATTTGAATAGGAATAAGGTGTGAATTGGT

GTTATAAATTTGGTTATTAACATTTTTAATCAAAACAGTTTTGGGAAGTAGCAATGGTCT

GACTTTGAAAATTCACTAAAAATTTTATAAATTGAACTAGAGGATGAACAAAATATGGAA

TTAAAGCTTATTGAGTCTAGTTTCTTATAGTAGAAACAATGTAAGCAATTAATTGATGAA

TCAAGAGATATTTGAAATTTTGTAATACTGGTTCGGGGTGATTTCGAGATGCCCTGTTTT

AACTTTGGAAAATCATTAAAAATTGTACAAAAATTATTATGGAGTGTAATTTATATATGT

GAACTCCTTAATGAATCTAGTTTCAAAATAAATAAACAAGAACCTTATTCGAGTTCTGTA

CAATGAGATAATTTAGTTTTAGTGGAGAGAGGTCAGAACTGTCAAATGAAATAACAGGGG

AGTATTTAACGAATAAACTGTATTAAATGGCTAGACCAAAAATTCTGGAAATTTTATGAT

TAGAAGATATATGAGTCTAGTTTTAAGGAAAATTTACGGATATTAATTTGGAGTTTCGTA

GCTCAAGATATAAATAATTTAGTAACAATGACCCAAGTAGACAGCTTAATGGTGAAATTA

TATAAATACATTAAAAATGGTTAAATTTGCATGTTTAGGCTCATGAATTAAATTGAATCA

TGTTGTATTGATTATTATAAATTATTATTTTCGTAGCCAACAAAGAACCTAAAGCATCAG

CATCGAAAGGAAAGGAGAAAGTCATCGAGGAGTAAACTCGAGAAAATTACGGTTTGTATT

ACTATAATTCAAGTTATTTATTATTAAATGTTAAATTTTAATTTATGTGTCTAGTAAATG

AAATGTGAGGTAAGTATTATTATTATTATTATTATTATTATTATTATTATTATTATTATT

ATGAGTGGGAATTAAATTGAATAGTTGATATGAAATAATATTTGAATTGTTTGTTGATTG

AAAGCGGGAAATGAATTTAAATCGAATAGTGACCGATATTAAATTGAATGGAAATGTATT

GAGTTGTGAAAATATGTTAATTGCGGATTAATTATTGATTGAAAGGTGGAAAAATGATTG

AATTGAAAGTGTGAGAAAGTGTGATTGAATTGGGATTATATGTGATTTAAATACCCTATT

AACTAGTCGGGCTGAGTCGGATATAGTTGGCATGCCATAGGATTGGAAGAGTTCAGGGAT

ACTTCGACCTCGAGTCGATGAGACACTGGGTGATTTCTTCGGATAGATTGGATGAGGTAC

TGGGTACCAACTTTCTTCGGCTTTGCCGATGAGACACTGGGTGTCAACTATTGCTTCGAA

CTATCCGATGAGGCACTGGGTGCCATTCTGGTGTGTTTGGTTGGATCCGTGTATCCGCCA

AAGTCCGAGTTTTGTTAATAGGGTAAATGATGAAATGATAAACCGAACGAGTTGGTCAAA

CGAGCTATTGAAATGATATGAAAAAGTTGAATTGTGAATTGAAATGTGAAATGAGATTGA

GAAATGAACCTAAGGTTCGTGAATTATTCAAACTCAAATTGTGGATATACGATATTGGTT

GATGAATTGCTATTGTTGAAATATTTAATTTAAATTGTATATACGATTTATGCTTTACAT

GTACATTATTGTTATAATTTGAATTATGGTAATACCACTGAGTATGAATTACTCAGCGTA

CGGTTGTTTCCGTGCGCAGGTCAATAGAAGTCAAAGGTCTCGGTTCAGCATCCAGATTAA

TCCCGGCTTCGGCAAAACTTGGTGATGTATTTTTCCTTTGGTAAAGGTGGCATGTACATA

GATTGTGTATAAAGGTTATTATGTTTTATTATATAATGGTTAAAAATGTTAGTATTAAAA

GTTTATGGATTTTAATGAAAGAAGTCTATCTATTTTATCTAATTAGTACATTGTTAAATT

TTAAATTGGTATTGTGTAGATTGAGTTTGATTAGAAGTATTTAGAATAGAAAATGTGAAT

GTGAAATGAATTGGTTGAATTGATGATATTTGGGAACTATATGGTTTTAATTTGCAGGGG

GTTTTATGTAAAAATAAGCAGAAATGCTGCCGAAATTTTTATAAAAAAAAATGAAGTCAT

TTGGTAAACAAATTAATAAATTTTATGAATTATTTTAATATATTGGTTATTTATTTAAGA

ATTGTTGTAAATCGTTCGATACGTCCGGTAGTGCCTCGTAATTCTGTTCCGGCGACGGTT

CGGGGTTAAGGGGTGTTACATTTTATGGTATCAGAGCTATCAGGTTTAGCCGATTCTCGG

CCTAAATCGAGCTCGGAATTGAGTCTAGATGTACATGCCACTGTCGAGTTAAACTGAGTC

GGGATTTTTGGATGCTGACCTATTTGTTTGTTTTGTTTTATAGATTAAAGATGTCTGAAG

AAAGAATAAATGATACTGATGAAAGAATGTATAGTGAAGATAGAGAATTAGATGAAACAG

AATCTGTTGCACCGAGTGTGAATCCGTTAGGCAACCAACCTTCTAATGTAGAACGAGAAA

ATGTCAGAGATAGAGATGAATCCCAATTACTGAGAATTATAGCTGATGCATTACAAAGAG

TAGCAGGAACTACTCCTGTTACGACTTCAGTACCTACTGTTAGACGGGCTCCGATAAAGG

AACTGAGGAAATATGGTGCCACTGAATTTATGGGTCTAAAAGGAGTTGATCCATCCATAG

CTGAAAATTGGATGGAGTCGACTAAAAGAATTTTGCAGCAATTGGATTGTACCCCCCGAG

AGTGTTTAATCTGTGCCGTATCGTTATTACAAGGGGAGGCTTATCTATGGTGGGAATCAG

TGGTTCGACATTTACCAGAGAGTCAGATAACGTGGGATCTATTTCAGAAGGAGTTTCAAA

AGAAATATATCGGAGAGATGTATATTGAAGACAAGAAACAAGAGTTTTTGTTGCTACAAC

AGGGTGATATGTCAGTAATAGATTATGAGAGGGAATTCTCGAGACTCAGTAGATATGCCT

CCGAGTTTATTCCGACAGAAGCCGATAGTTGTAAAAGATTTTTACGGGGTTTACGAGACG

AGATCAAAGTGCAGCTAGTATCCCATCGGATCACTGAGTTAGTAGATTTGATTGAACGAG

CTAAAATGGTGGAACAAGTTCTGGGCCTCGACAAAAAGACTGAAGTTGTTAGACCAACCG

GGAAGCGTACAGGAACTACCAGTTCGAATCCTCAGCCGAAAAGACCAAAGGAATTCCAAA

GTGGTTGGAGATCCAGTTTCAGGTCAGACAGAGGTGGTAGAAATAGGGGAAAACAGACGA

TGACATCTACTGGCAGTGTGAAAGGTCCTTCCCGAGAAATAGATATTCCAGACTGCCAAC

ACTGCGGAAAGAAACACAGAGGGGAATGTTGGAAATTAACTAGAGGCTGTTTTCGATGTG

GTTCTACAGACCATTTCATCAGAGACTGTCCGAAAGTTGATAGTACTGTACCCGTGACAT

CACAGAGATCGGTATCTACAGCTAGAGGCAGAGGGTTAGGAAGAGGTGGTTCGGTTTCAA

GGGGAGGAAGTATTAGGAGAAGCAATGATATTGCTACTCAGCAGTCTGAGGCTAAAGTAC

CTGCCAGAGCTTATGTGGTCAGAACACAGGAAGAAGGTGACGCCCACGATGTAGTAACAG

GTATATTCTTACTATATTCTGAGCCTGTTTATGCTTTAATTGATCCCGGATCTTCACATT

CTTATATAAATTCAAAATTAGTTGAATTGGGAAAATTTAATTCTGAAATATCTAGAGTGA

CTGTAGAAGTGTCGAGTCCGTTGGGGCAAACAGTATTAGTGAATCAGATCTGTCCGAGAT

GCCCGTTAATTATACAAAATAAAACTTTTCCTATTGACCTGTTGATTATGCCATTTGGAG

ATTTTGATATAATACTGGGGATGGATTGGTTGGCTGAGCACGGAGTGGTATTGGATTGTT

ATAAAAAGAAGTTTAGTATTCAGACAGAAGACGGGGACAGAATTGAAGTAAATGGTATCC

GTACTAATGGGCCGACACGTATTATTTCGGCAATAAAGGCTAATAAATTGCTTCAGCGGG

GTTGTACAGCGTATTTAGCCTATGTTATTAATTCTGATTTGGTTGGTAGTCAGTGCAGTA

AGATTAGAACCGTATGTGAGTTTCCAGATGTATTTCCTGAAGAGCTACCGGGTTTACCAC

CTGACAGAGAGGTTGAATTTGCTATAGAAGTGTATCCGGGTACAGCACCAATCTCTATAC

CACCGTATCGAATGTCACCCACTGAGTTGAAAGAGTTGAAAGTGCAGTTACAGGACTTGT

CAGATCGTGGATTTATTAGACCGAGCATCTCACCTTGGGGAGCTCCAGTATTGTTTGTTA

AAAAGAAAGATGGATCGATGCGGCTTTGTATTGATTACCGGCAGTTAAACAAAGTGACGA

TCAAGAACCGGTATCCGTTACCCCGTATAGATGATTTATTTGATCAACTAAAAGGAGCTT

CAGTATTTTCAAAGATTGACTTAAGATCTGGGTATTATCAGCTGAAGGTAAAAGAAAGTG

ATGTTCCGAAGACTGCATTTCGTACTCGATATGGTCATTATGAATTTTTGGTGATGCCGT

TCGGGTTGACTAATGCTCCAGCTGCTTTTATGGATCTGATGAATCGTATTTTTCAGCCGT

ATTTAGATCAGTTTGTGGTGGTTTTTATTGATGACATCTTGGTTTATTCGAAGTCAGAGT

CAGAGCATGATCAGCATCTCAGAACCGTGCTACAAATTCTGCGAGAAAAACAGTTGTACG

GGAAACTAAGTAAATGTGAATTCTGGTTATCAGAGGTAGTATTCTTGGGACATGTTGTAT

CTGCGGATGGGATTAGAGTTGATCCGAAGAAGATCGAGGCAATTGTTCAATGGAAGGCAC

CAAAGAATGTATCAGAGGTACGCAGTTTTCTTGGTTTGGCTGGGTATTACAGAAGATTTG

TAAATGGGTTTTCGAAGATAGCTTTGCCGATGACCAAATTACTACAGAAGAATGTTCCAT

TTATCTGGGATGATCAGTGTCAGAGGAGCTTTGAAACATTGAAACAGATGTTGACAGAGG

CACCAGTTTTAACTTTACCAGAATCAGGGAAAGATTTCATAGTGTACAGTGATGCTTCTT

TGAATGGTTTGGGTTGTGTATTGATGCAAGAAGGAAAAGTAATAGCTTATGCATCTCGAC

AGTTGAAGTCACATGAACGCAACTACCCGACACACGATTTAGAGTTAGCTGCTGTAATCT

TTGCATTGAAGATTTGGATACATTACTTGTATGGTGAGAAATGTTATATTTACACTGATC

ATAAAAGTCTAAAATATCTTCTGTCACAAAAGGAGTTGAATCTGAGACAGAGACGGTGGA

TTGAACTTCTGAAAGATTATGATTGTGTTATAGATTATCATCCAGGGAAGGCAAATGTGG

TAGCAGATGCATTGAGTAGAAAAGCAGCGATTGAATTACGAGCAATGTTCGCTCGACTTA

GTATTAAGGATGATGGAAGTTTGTTAGCTGAGTTAAGAGTCAAGCCGGTGATGTTTGATC

AAATCAGAGCAGCACAGTTAAAAGATGAAAAGTTGATGAGGAAAAGAGAAATGGTACAGT

ATGGTGCGGTAGAAAATTTTAGTATTGACGAGCATGATTGTTTGAGATTTCGAAATCGAA

TTTGTGTTCCATCTACTTCTGAGATTAAAGAATTGATTCTCCGAGAAGCACATAATAGTA

TTTTTGCTTTGCACCCAGGAGGAACGAAGATGTATCGTGATCTACGAGAACTGTATTGGT

GGCCAGGAATGAAGAAAGATATAGTTGAATATGTCAGTAAATGCTTGACTTGTCAGCGGG

TAAAAGCAGAACATCAGGTACCAACAGGCCTGTTACAGCCTATTACTATTCCCGAGTGGA

AATGGGATCGCATTACCATGGATTTTGTTACGGGGTTGCCATTGTCAGTGAGTAAAAAGA

ATGCTATTTGGGTGATTGTTGATCGACTCACAAAATCAGCTCATTTTATAGCAGTTAGAA

CCGACTGGTCATTACAGAAGCTTGCCGAGGTTTATATTCGAGAAATTGTTAGATTACATG

GTATTCCGGTATCAATAATTTCAGACAGAGATCCTCGATTCACTTCGAGATTTTGGAAGC

AGCTGCATGAATCATTGGGTACTCGACTTAGTTTCAGTACAGCTTTTCATCCTCAAACTG

ATGGACAATCTGAACGAGTAATTCAGATATTAGAAGATATGCTTCGAGCTTGTGTCATTG

ATTTTGAATCAGGTTGGGAACGTTATTTACCATTGGCCGAGTTTGTTTATAATAATAGTT

TCCAATCTAGTATTCAAATGGCTCCATATGAAGCACTTTATGGTCGAAGGTGTCGATCAC

CAATATGTTGGACAAAATTAAGAGAAAGAAAAGTGATTGGGCCGGAATTGATTCAAGAGA

CAGAAGAAACAGTTAAAAAGATTAAAGATAGACTGAAAGCCGCTTTCGACAGACAGAAAT

CTTACGCAGACTTGAAACGACGAGACATTGAATATTCCGTTGGTGATAAGGTATTCCTCA

AAGTATCGCCGTGGAAGAAAATTTTGAGATTTGGTCGGAAGGGAAAATTAAGTCCGCGCT

TTATTGGGCCGTATGAGATAGTGGAAAGAATTGGGCCTGTTGCTTATCGATTATCCTTAC

CTCCAGAGTTACAGAAAATTCATGATGTTTTTCATGTTTCGATGCTTCGGAGATATAGAT

CGGATCCTTCTCATGTTATTCCCACTGAAGACATTGAACTTCGATCTGATTTAACTTATG

AAGAAGAACCAGTTCAAATATTAGCACGAGAAGTGAAAGAATTAAGAAATAAACGGGTTC

CTTTAGTACAAGTTTTATGGAGAAGCCATAGTGTGGAAGAAGCAACTTGGGAACCGGAAG

AGACAATGAGAGCACAATATCCTCATCTCTTCTCAGGTAAATTTCGAGGACGAAATTTAT

TAAGAGGGGGAGAAATGTAATGACCTAAAATTCATGGGCATCGGAAAAGTATAATATTGG

GCCTCCGTCCTAGTAAATTGAGTCCGAAAATAATTATTAGAAATATTTACGAGACTAGTA

GTGTGTTTAATTAGGTTTTAATTAAGTAAATTTAGCTTAATTTAGAGTAATTAGTAAAAA

GGATTAAATTGAATAAGAGTAAAAGTTTAATTATAGATTAAAGGAAAATAATAGGGACCA

AATGGGCAATTAAGCCACATTTGGAAGTTGAGGCGGCATAACATTGTAAAAATCTTAGAT

TTTTATATTATTATTTATATAAATATATAAATTAATTATAAAGTATATTATTAAATTAAT

TATATTATAAATATTATATTATTATATATAAAAGAAACAAAACAGAAAAGAAACAGAATA

GAAAGAACAAAGAAACAGAATAGAAGAGACGAAACAGGGGAGAAGCAGGGGAGAAAGAAG

AAAAAGAAGAAAAAAGGGGAAATAGGGTTTTTGAAGCTTGAAATTTAAATTGGTAAGTCA

AATTAGCCATTTTCTCTTAATTCTAATGTTTTAAAAGCTTTAAAACAAAGTTTTGATGGA

ATTAAGTTGATATTTTGTAAGTTCATAGGTTTTCAAGTATAGTTTATGTTGAACAAAAGA

GATGAATTAGGGATTAACTTGAAGGAATTTTAAGTTAGAATTGAAAAAGGGATTAAATTG

TAAAAGAAACTATAAGTTTTTTTTGTTTTAGGGACTAGATTGAGGAAAATTCGGAATTAA

GAAAATATGTTAAAAATTTAATAGTTAAATTTGAGTTTAAATGAAATTTGAATAGGAATA

AGGTGTGAATTGGTGTTATAAATTTGGTTATTAACATTTTTAATCAAAACAGTTTTGGGA

AGTAGCAATGGTCTGACTTTGAAAATTCACTAAAAATTTTATAAATTGAACTAGAGGATG

AACAAAATATGGAATTAAAGCTTATTGAGTCTAGTTTCTTATAGTAGAAACAATGTAAGC

AATTAATTGATGAATCAAGAGATATTTGAAATTTTGTAATACTGGTTCGGGGTGATTTCG

AGATGCCCTGTTTTAACTTTGGAAAATCATTAAAAATTGTACAAAAATTATTATGGAGTG

TAATTTATATATGTAAACTCCTTAATGAATCTAGTTTCAAAATAAATAAACAAGAACCTT

ATTCGAGTTCTGTACAATGAGATAATTTAGTTTTAGTGGAGAGAGGTCAGAACTGTCAAA

TGAAATAACAGGGGAGTATTTAACGAATAAACTGTATTAAATGGCTAGACCAAAAATTCT

GGAAATTTTATGATTAGAAGATATATGAGTCTAGTTTTAAGGAAAATTTACGGATATTAA

TTTGGAGTTTCGTAGCTCAAGATATAAATAATTTAGTAACAATGACCCAAGTAGACAGCT

TAATGGTGAAATTATATAAATACATTAAAAATGGTTAAATTTGCATGTTTAGGCTCATGA

ATTAAATTGAATCATGTTGTATTGATTATTATAAATTATTATTTTCGTAGCCAACAAAGA

ACCTAAAGCATCAGCATCGAAAGGAAAGGAGAAAGTCATCGAGGAGTAAACTCGAGAAAA

TTACGGTTTGTATTACTATAATTCAAGTTATTTATTATTAAATGTTAAATTTTAATTTAT

GTGTCTAGTAAATGAAATGTGAGGTAAGTATTATTATTATTATTATTATTATTATTATTA

TTATTATTATTATTATTATGAGTGGGAATTAAATTGAATAGTTGATATGAAATAATATTT

GAATTGTTTGTTGATTGAAAGCGGGAAATGAATTTAAATCGAATAGTGACCGATATTAAA

TTGAATGGAAATGTATTGAGTTGTGAAAATATGTTAATTGCGGATTAATTATTGATTGAA

AGGTGGAAAAATGATTGAATTGAAAGTGTGAGAAAGTGTGATTGAATTGGGATTATATGT

GATTTAAATACCCTATTAACTAGTCGGGCTGAGTCGGATATAGTTGGCATGCCATAGGAT

TGGAAGAGTTCAGGGATACTTCGACCTCGAGTCGATGAGACACTGGGTGTCACTATATTT

CTTCGGATAGATTCGATGAGGTACTGGGTACCAACTTTCTTCGGCTTTGCCGATGAGACA

CTGGGTGTCAACTATTGCTTCGAACTATCCGATGAGGCACTGGGTGCCATTCTGGTGTGT

TTGGTTGGATCCGTGTATTCGCCAAAGTCCGAGTTTTGTTAATAGGGTAAATGATGAAAT

GATAAACCGAACGAGTTGGTCAAACGAGCTATTGAAATGATATGAAAAAGTTGAATTGTG

AATTGAAATGTGAAATGAGATTGAGAAATGAACCTAAGGTTCGTGAATTATTCAAACTCA

AATTGTGGATATACGATATTGGTTGATGAATTGCTATTGTTGAAATATTTAATTTAAATT

GTATATACGATTTATGCTTTACATGTACATTATTGTTATAATTTGAATTATGGTAATACC

ACTGAGTATGAATTACTCAGCGTACGGTTGTTTCCGTGCGCAGGTCAATAGAAGTCAAAG

GTCTCGGTTCAGCATCCAGATTAATCCCGGCTTCGGCAAAACTTGGTGATGTATTTTTCC

TTTGGTAAAGGTGGCATGTACATAGATTGTGTATAAAGGTTATTATGTTTTATTATATAA

TGGTTAAAAATGTTAGTATTAAAAGTTTATGGATTTTAATGAAAGAAGTCTATCTATTTT

ATCTAATTAGTACATTGTTAAATTTTAAATTGGTATTAGATTGAGTTTGATTAGAAGTAT

TTAGAATAGAAAATGTGAATGTGAAATGAATTGGTTGAATTGATGATATTTGGGAACTAT

ATGGTTTTAATTTGC

>Deltapine20

AGGTAATGACCCAAAATTCATGGGCATCGGAAAAGTATAATATCGGGCCTCCGTCCTAGT

AAATTGAGTCCGAAAATAATTATTAGAAATATTTACGAGACTAGTAGTGTGTTTAATTAG

GTTTTAATTAAGTAAATTTAGCTTAATTTAGAGTAATTAGTAAAAAGGATTAAATTGAAT

AAGAGTAAAAGTTTAATTATAGATTAAAGGAAAATAATAGGGACCAAATGGGCAATTAAG

CCACATTTGGAAGTTGAGGCGGCATAACATTGTAAAAATCTTAGATTTTTATATTATTAT

TTATATAAATATATAAATTAATTATAAAGTATATTATTAAATTAATTATATTATAAATAT

TATATTATTATATATAAAAGAAACAAAACAGAAAAGAAACAGAATAGAAAGAACAAAGAA

ACAGAATAGAAGAGACGAAACAGGGGAGAAGCAGGGGAGAAAGAAGAAAAAGAAGAAAAA

AGGGGAAATAGGGTTTTTGAAGCTTGAAATTTAAATTGGTAAGTCAAATTAGCCATTTTC

TCTTAATTCTAATGTTTTAAAAGCTTTAAAACAAAGTTTTGATGGAATTAAGTTGATATT

TTGTAAGTTCATAGGTTTTCAAGTATAGTTTATGTTGAACAAAAGAGATGAATTAGGGAT

TAACTTGAAGGAATTTTAAGTTAGAATTGAAAAAGGGATTAAATTGTAAAAGAAACTATA

AGTTTTTTTTGTTTTAGGGACTAGATTGAGGAAAATTCGGAATTAAGAAAATATGTTAAA

AATTTAATAGTTAAATTTGAGTTTAAATGAAATTTGAATAGGAATAAGGTGTGAATTGGT

GTTATAAATTTGGTTATTAACATTTTTAATCAAAACAGTTTTGGGAAGTAGCAATGGTCT

GACTTTGAAAATTCACTAAAAATTTTATAAATTGAACTAGAGGATGAACAAAATATGGAA

TTAAAGCTTATTGAGTCTAGTTTCTTATAGTAGAAACAATGTAAGCAATTAATTGATGAA

TCAAGAGATATTTGAAATTTTGTAATACTGGTTCGGGGTGATTTCGAGATGCCCTGTTTT

AACTTTGGAAAATCATTAAAAATTGTACAAAAATTATTATGGAGTGTAATTTATATATGT

GAACTCCTTAATGAATCTAGTTTCAAAATAAATAAACAAGAACCTTATTCGAGTTCTGTA

CAATGAGATAATTTAGTTTTAGTGGAGAGAGGTCAGAACTGTCAAATGAAATAACAGGGG

AGTATTTAACGAATAAACTGTATTAAATGGCTAGACCAAAAATTCTGGAAATTTTATGAT

TAGAAGATATATGAGTCTAGTTTTAAGGAAAATTTACGGATATTAATTTGGAGTTTCGTA

GCTCAAGATATAAATAATTTAGTAACAATGACCCAAGTAGACAGCTTAATGGTGAAATTA

TATAAATACATTAAAAATGGTTAAATTTGCATGTTTAGGCTCATGAATTAAATTGAATCA

TGTTGTATTGATTATTATAAATTATTATTTTCGTAGCCAACAAAGAACCTAAAGCATCAG

CATCGAAAGGAAAGGAGAAAGTCATCGAGGAGTAAACTCGAGAAAATTACGGTTTGTATT

ACTATAATTCAAGTTATTTATTATTAAATGTTAAATTTTAATTTATGTGTCTAGTAAATG

AAATGTGAGGTAAGTATTATTATTATTATTATTATTATTATTATTATTATTATTATTATT

ATGAGTGGGAATTAAATTGAATAGTTGATATGAAATAATATTTGAATTGTTTGTTGATTG

AAAGCGGGAAATGAATTTAAATCGAATAGTGACCGATATTAAATTGAATGGAAATGTATT

GAGTTGTGAAAATATGTTAATTGCGGATTAATTATTGATTGAAAGGTGGAAAAATGATTG

AATTGAAAGTGTGAGAAAGTGTGATTGAATTGGGATTATATGTGATTTAAATACCCTATT

AACTAGTCGGGCTGAGTCGGATATAGTTGGCATGCCATAGGATTGGAAGAGTTCAGGGAT

ACTTCGACCTCGAGTCGATGAGACACTGGGTGATTTCTTCGGATAGATTGGATGAGGTAC

TGGGTACCAACTTTCTTCGGCTTTGCCGATGAGACACTGGGTGTCAACTATTGCTTCGAA

CTATCCGATGAGGCACTGGGTGCCATTCTGGTGTGTTTGGTTGGATCCGTGTATCCGCCA

AAGTCCGAGTTTTGTTAATAGGGTAAATGATGAAATGATAAACCGAACGAGTTGGTCAAA

CGAGCTATTGAAATGATATGAAAAAGTTGAATTGTGAATTGAAATGTGAAATGAGATTGA

GAAATGAACCTAAGGTTCGTGAATTATTCAAACTCAAATTGTGGATATACGATATTGGTT

GATGAATTGCTATTGTTGAAATATTTAATTTAAATTGTATATACGATTTATGCTTTACAT

GTACATTATTGTTATAATTTGAATTATGGTAATACCACTGAGTATGAATTACTCAGCGTA

CGGTTGTTTCCGTGCGCAGGTCAATAGAAGTCAAAGGTCTCGGTTCAGCATCCAGATTAA

TCCCGGCTTCGGCAAAACTTGGTGATGTATTTTTCCTTTGGTAAAGGTGGCATGTACATA

GATTGTGTATAAAGGTTATTATGTTTTATTATATAATGGTTAAAAATGTTAGTATTAAAA

GTTTATGGATTTTAATGAAAGAAGTCTATCTATTTTATCTAATTAGTACATTGTTAAATT

TTAAATTGGTATTGTGTAGATTGAGTTTGATTAGAAGTATTTAGAATAGAAAATGTGAAT

GTGAAATGAATTGGTTGAATTGATGATATTTGGGAACTATATGGTTTTAATTTGCAGGGG

GTTTTATGTAAAAATAAGCAGAAATGCTGCCGAAATTTTTATAAAAAAAAATGAAGTCAT

TTGGTAAACAAATTAATAAATTTTATGAATTATTTTAATATATTGGTTATTTATTTAAGA

ATTGTTGTAAATCGTTCGATACGTCCGGTAGTGCCTCGTAATTCTGTTCCGGCGACGGTT

CGGGGTTAAGGGGTGTTACATTTTATGGTATCAGAGCTATCAGGTTTAGCCGATTCTCGG

CCTAAATCGAGCTCGGAATTGAGTCTAGATGTACATGCCACTGTCGAGTTAAACTGAGTC

GGGATTTTTGGATGCTGACCTATTTGTTTGTTTTGTTTTATAGATTAAAGATGTCTGAAG

AAAGAATAAATGATACTGATGAAAGAATGTATAGTGAAGATAGAGAATTAGATGAAACAG

AATCTGTTGCACCGAGTGTGAATCCGTTAGGCAACCAACCTTCTAATGTAGAACGAGAAA

ATGTCAGAGATAGAGATGAATCCCAATTACTGAGAATTATAGCTGATGCATTACAAAGAG

TAGCAGGAACTACTCCTGTTACGACTTCAGTACCTACTGTTAGACGGGCTCCGATAAAGG

AACTGAGGAAATATGGTGCCACTGAATTTATGGGTCTAAAAGGAGTTGATCCATCCATAG

CTGAAAATTGGATGGAGTCGACTAAAAGAATTTTGCAGCAATTGGATTGTACCCCCCGAG

AGTGTTTAATCTGTGCCGTATCGTTATTACAAGGGGAGGCTTATCTATGGTGGGAATCAG

TGGTTCGACATTTACCAGAGAGTCAGATAACGTGGGATCTATTTCAGAAGGAGTTTCAAA

AGAAATATATCGGAGAGATGTATATTGAAGACAAGAAACAAGAGTTTTTGTTGCTACAAC

AGGGTGATATGTCAGTAATAGATTATGAGAGGGAATTCTCGAGACTCAGTAGATATGCCT

CCGAGTTTATTCCGACAGAAGCCGATAGTTGTAAAAGATTTTTACGGGGTTTACGAGACG

AGATCAAAGTGCAGCTAGTATCCCATCGGATCACTGAGTTAGTAGATTTGATTGAACGAG

CTAAAATGGTGGAACAAGTTCTGGGCCTCGACAAAAAGACTGAAGTTGTTAGACCAACCG

GGAAGCGTACAGGAACTACCAGTTCGAATCCTCAGCCGAAAAGACCAAAGGAATTCCAAA

GTGGTTGGAGATCCAGTTTCAGGTCAGACAGAGGTGGTAGAAATAGGGGAAAACAGACGA

TGACATCTACTGGCAGTGTGAAAGGTCCTTCCCGAGAAATAGATATTCCAGACTGCCAAC

ACTGCGGAAAGAAACACAGAGGGGAATGTTGGAAATTAACTAGAGGCTGTTTTCGATGTG

GTTCTACAGACCATTTCATCAGAGACTGTCCGAAAGTTGATAGTACTGTACCCGTGACAT

CACAGAGATCGGTATCTACAGCTAGAGGCAGAGGGTTAGGAAGAGGTGGTTCGGTTTCAA

GGGGAGGAAGTATTAGGAGAAGCAATGATATTGCTACTCAGCAGTCTGAGGCTAAAGTAC

CTGCCAGAGCTTATGTGGTCAGAACACAGGAAGAAGGTGACGCCCACGATGTAGTAACAG

GTATATTCTTACTATATTCTGAGCCTGTTTATGCTTTAATTGATCCCGGATCTTCACATT

CTTATATAAATTCAAAATTAGTTGAATTGGGAAAATTTAATTCTGAAATATCTAGAGTGA

CTGTAGAAGTGTCGAGTCCGTTGGGGCAAACAGTATTAGTGAATCAGATCTGTCCGAGAT

GCCCGTTAATTATACAAAATAAAACTTTTCCTATTGACCTGTTGATTATGCCATTTGGAG

ATTTTGATATAATACTGGGGATGGATTGGTTGGCTGAGCACGGAGTGGTATTGGATTGTT

ATAAAAAGAAGTTTAGTATTCAGACAGAAGACGGGGACAGAATTGAAGTAAATGGTATCC

GTACTAATGGGCCGACACGTATTATTTCGGCAATAAAGGCTAATAAATTGCTTCAGCGGG

GTTGTACAGCGTATTTAGCCTATGTTATTAATTCTGATTTGGTTGGTAGTCAGTGCAGTA

AGATTAGAACCGTATGTGAGTTTCCAGATGTATTTCCTGAAGAGCTACCGGGTTTACCAC

CTGACAGAGAGGTTGAATTTGCTATAGAAGTGTATCCGGGTACAGCACCAATCTCTATAC

CACCGTATCGAATGTCACCCACTGAGTTGAAAGAGTTGAAAGTGCAGTTACAGGACTTGT

CAGATCGTGGATTTATTAGACCGAGCATCTCACCTTGGGGAGCTCCAGTATTGTTTGTTA

AAAAGAAAGATGGATCGATGCGGCTTTGTATTGATTACCGGCAGTTAAACAAAGTGACGA

TCAAGAACCGGTATCCGTTACCCCGTATAGATGATTTATTTGATCAACTAAAAGGAGCTT

CAGTATTTTCAAAGATTGACTTAAGATCTGGGTATTATCAGCTGAAGGTAAAAGAAAGTG

ATGTTCCGAAGACTGCATTTCGTACTCGATATGGTCATTATGAATTTTTGGTGATGCCGT

TCGGGTTGACTAATGCTCCAGCTGCTTTTATGGATCTGATGAATCGTATTTTTCAGCCGT

ATTTAGATCAGTTTGTGGTGGTTTTTATTGATGACATCTTGGTTTATTCGAAGTCAGAGT

CAGAGCATGATCAGCATCTCAGAACCGTGCTACAAATTCTGCGAGAAAAACAGTTGTACG

GGAAACTAAGTAAATGTGAATTCTGGTTATCAGAGGTAGTATTCTTGGGACATGTTGTAT

CTGCGGATGGGATTAGAGTTGATCCGAAGAAGATCGAGGCAATTGTTCAATGGAAGGCAC

CAAAGAATGTATCAGAGGTACGCAGTTTTCTTGGTTTGGCTGGGTATTACAGAAGATTTG

TAAATGGGTTTTCGAAGATAGCTTTGCCGATGACCAAATTACTACAGAAGAATGTTCCAT

TTATCTGGGATGATCAGTGTCAGAGGAGCTTTGAAACATTGAAACAGATGTTGACAGAGG

CACCAGTTTTAACTTTACCAGAATCAGGGAAAGATTTCATAGTGTACAGTGATGCTTCTT

TGAATGGTTTGGGTTGTGTATTGATGCAAGAAGGAAAAGTAATAGCTTATGCATCTCGAC

AGTTGAAGTCACATGAACGCAACTACCCGACACACGATTTAGAGTTAGCTGCTGTAATCT

TTGCATTGAAGATTTGGATACATTACTTGTATGGTGAGAAATGTTATATTTACACTGATC

ATAAAAGTCTAAAATATCTTCTGTCACAAAAGGAGTTGAATCTGAGACAGAGACGGTGGA

TTGAACTTCTGAAAGATTATGATTGTGTTATAGATTATCATCCAGGGAAGGCAAATGTGG

TAGCAGATGCATTGAGTAGAAAAGCAGCGATTGAATTACGAGCAATGTTCGCTCGACTTA

GTATTAAGGATGATGGAAGTTTGTTAGCTGAGTTAAGAGTCAAGCCGGTGATGTTTGATC

AAATCAGAGCAGCACAGTTAAAAGATGAAAAGTTGATGAGGAAAAGAGAAATGGTACAGT

ATGGTGCGGTAGAAAATTTTAGTATTGACGAGCATGATTGTTTGAGATTTCGAAATCGAA

TTTGTGTTCCATCTACTTCTGAGATTAAAGAATTGATTCTCCGAGAAGCACATAATAGTA

TTTTTGCTTTGCACCCAGGAGGAACGAAGATGTATCGTGATCTACGAGAACTGTATTGGT

GGCCAGGAATGAAGAAAGATATAGTTGAATATGTCAGTAAATGCTTGACTTGTCAGCGGG

TAAAAGCAGAACATCAGGTACCAACAGGCCTGTTACAGCCTATTACTATTCCCGAGTGGA

AATGGGATCGCATTACCATGGATTTTGTTACGGGGTTGCCATTGTCAGTGAGTAAAAAGA

ATGCTATTTGGGTGATTGTTGATCGACTCACAAAATCAGCTCATTTTATAGCAGTTAGAA

CCGACTGGTCATTACAGAAGCTTGCCGAGGTTTATATTCGAGAAATTGTTAGATTACATG

GTATTCCGGTATCAATAATTTCAGACAGAGATCCTCGATTCACTTCGAGATTTTGGAAGC

AGCTGCATGAATCATTGGGTACTCGACTTAGTTTCAGTACAGCTTTTCATCCTCAAACTG

ATGGACAATCTGAACGAGTAATTCAGATATTAGAAGATATGCTTCGAGCTTGTGTCATTG

ATTTTGAATCAGGTTGGGAACGTTATTTACCATTGGCCGAGTTTGTTTATAATAATAGTT

TCCAATCTAGTATTCAAATGGCTCCATATGAAGCACTTTATGGTCGAAGGTGTCGATCAC

CAATATGTTGGACAAAATTAAGAGAAAGAAAAGTGATTGGGCCGGAATTGATTCAAGAGA

CAGAAGAAACAGTTAAAAAGATTAAAGATAGACTGAAAGCCGCTTTCGACAGACAGAAAT

CTTACGCAGACTTGAAACGACGAGACATTGAATATTCCGTTGGTGATAAGGTATTCCTCA

AAGTATCGCCGTGGAAGAAAATTTTGAGATTTGGTCGGAAGGGAAAATTAAGTCCGCGCT

TTATTGGGCCGTATGAGATAGTGGAAAGAATTGGGCCTGTTGCTTATCGATTATCCTTAC

CTCCAGAGTTACAGAAAATTCATGATGTTTTTCATGTTTCGATGCTTCGGAGATATAGAT

CGGATCCTTCTCATGTTATTCCCACTGAAGACATTGAACTTCGATCTGATTTAACTTATG

AAGAAGAACCAGTTCAAATATTAGCACGAGAAGTGAAAGAATTAAGAAATAAACGGGTTC

CTTTAGTACAAGTTTTATGGAGAAGCCATAGTGTGGAAGAAGCAACTTGGGAACCGGAAG

AGACAATGAGAGCACAATATCCTCATCTCTTCTCAGGTAAATTTCGAGGACGAAATTTAT

TAAGAGGGGGAGAAATGTAATGACCTAAAATTCATGGGCATCGGAAAAGTATAATATTGG

GCCTCCGTCCTAGTAAATTGAGTCCGAAAATAATTATTAGAAATATTTACGAGACTAGTA

GTGTGTTTAATTAGGTTTTAATTAAGTAAATTTAGCTTAATTTAGAGTAATTAGTAAAAA

GGATTAAATTGAATAAGAGTAAAAGTTTAATTATAGATTAAAGGAAAATAATAGGGACCA

AATGGGCAATTAAGCCACATTTGGAAGTTGAGGCGGCATAACATTGTAAAAATCTTAGAT

TTTTATATTATTATTTATATAAATATATAAATTAATTATAAAGTATATTATTAAATTAAT

TATATTATAAATATTATATTATTATATATAAAAGAAACAAAACAGAAAAGAAACAGAATA

GAAAGAACAAAGAAACAGAATAGAAGAGACGAAACAGGGGAGAAGCAGGGGAGAAAGAAG

AAAAAGAAGAAAAAAGGGGAAATAGGGTTTTTGAAGCTTGAAATTTAAATTGGTAAGTCA

AATTAGCCATTTTCTCTTAATTCTAATGTTTTAAAAGCTTTAAAACAAAGTTTTGATGGA

ATTAAGTTGATATTTTGTAAGTTCATAGGTTTTCAAGTATAGTTTATGTTGAACAAAAGA

GATGAATTAGGGATTAACTTGAAGGAATTTTAAGTTAGAATTGAAAAAGGGATTAAATTG

TAAAAGAAACTATAAGTTTTTTTTGTTTTAGGGACTAGATTGAGGAAAATTCGGAATTAA

GAAAATATGTTAAAAATTTAATAGTTAAATTTGAGTTTAAATGAAATTTGAATAGGAATA

AGGTGTGAATTGGTGTTATAAATTTGGTTATTAACATTTTTAATCAAAACAGTTTTGGGA

AGTAGCAATGGTCTGACTTTGAAAATTCACTAAAAATTTTATAAATTGAACTAGAGGATG

AACAAAATATGGAATTAAAGCTTATTGAGTCTAGTTTCTTATAGTAGAAACAATGTAAGC

AATTAATTGATGAATCAAGAGATATTTGAAATTTTGTAATACTGGTTCGGGGTGATTTCG

AGATGCCCTGTTTTAACTTTGGAAAATCATTAAAAATTGTACAAAAATTATTATGGAGTG

TAATTTATATATGTAAACTCCTTAATGAATCTAGTTTCAAAATAAATAAACAAGAACCTT

ATTCGAGTTCTGTACAATGAGATAATTTAGTTTTAGTGGAGAGAGGTCAGAACTGTCAAA

TGAAATAACAGGGGAGTATTTAACGAATAAACTGTATTAAATGGCTAGACCAAAAATTCT

GGAAATTTTATGATTAGAAGATATATGAGTCTAGTTTTAAGGAAAATTTACGGATATTAA

TTTGGAGTTTCGTAGCTCAAGATATAAATAATTTAGTAACAATGACCCAAGTAGACAGCT

TAATGGTGAAATTATATAAATACATTAAAAATGGTTAAATTTGCATGTTTAGGCTCATGA

ATTAAATTGAATCATGTTGTATTGATTATTATAAATTATTATTTTCGTAGCCAACAAAGA

ACCTAAAGCATCAGCATCGAAAGGAAAGGAGAAAGTCATCGAGGAGTAAACTCGAGAAAA

TTACGGTTTGTATTACTATAATTCAAGTTATTTATTATTAAATGTTAAATTTTAATTTAT

GTGTCTAGTAAATGAAATGTGAGGTAAGTATTATTATTATTATTATTATTATTATTATTA

TTATTATTATTATTATTATGAGTGGGAATTAAATTGAATAGTTGATATGAAATAATATTT

GAATTGTTTGTTGATTGAAAGCGGGAAATGAATTTAAATCGAATAGTGACCGATATTAAA

TTGAATGGAAATGTATTGAGTTGTGAAAATATGTTAATTGCGGATTAATTATTGATTGAA

AGGTGGAAAAATGATTGAATTGAAAGTGTGAGAAAGTGTGATTGAATTGGGATTATATGT

GATTTAAATACCCTATTAACTAGTCGGGCTGAGTCGGATATAGTTGGCATGCCATAGGAT

TGGAAGAGTTCAGGGATACTTCGACCTCGAGTCGATGAGACACTGGGTGTCACTATATTT

CTTCGGATAGATTCGATGAGGTACTGGGTACCAACTTTCTTCGGCTTTGCCGATGAGACA

CTGGGTGTCAACTATTGCTTCGAACTATCCGATGAGGCACTGGGTGCCATTCTGGTGTGT

TTGGTTGGATCCGTGTATTCGCCAAAGTCCGAGTTTTGTTAATAGGGTAAATGATGAAAT

GATAAACCGAACGAGTTGGTCAAACGAGCTATTGAAATGATATGAAAAAGTTGAATTGTG

AATTGAAATGTGAAATGAGATTGAGAAATGAACCTAAGGTTCGTGAATTATTCAAACTCA

AATTGTGGATATACGATATTGGTTGATGAATTGCTATTGTTGAAATATTTAATTTAAATT

GTATATACGATTTATGCTTTACATGTACATTATTGTTATAATTTGAATTATGGTAATACC

ACTGAGTATGAATTACTCAGCGTACGGTTGTTTCCGTGCGCAGGTCAATAGAAGTCAAAG

GTCTCGGTTCAGCATCCAGATTAATCCCGGCTTCGGCAAAACTTGGTGATGTATTTTTCC

TTTGGTAAAGGTGGCATGTACATAGATTGTGTATAAAGGTTATTATGTTTTATTATATAA

TGGTTAAAAATGTTAGTATTAAAAGTTTATGGATTTTAATGAAAGAAGTCTATCTATTTT

ATCTAATTAGTACATTGTTAAATTTTAAATTGGTATTAGATTGAGTTTGATTAGAAGTAT

TTAGAATAGAAAATGTGAATGTGAAATGAATTGGTTGAATTGATGATATTTGGGAACTAT

ATGGTTTTAATTTGC

>Deltapine25

ATGTAATGACCCAAAATTCATGGGCATCGGAAAAGTATAATATCGGGCCTCCGTCCTAGT

AAATTGAGTCCGAAAATAATTATTAGAAATATTTACGAGACTAGTAGTGTGTTTAATTAG

GTTTTAATTAAGTAAATTTAGCTTAATTTAGAGTAATTAGTAAAAAGGATTAAATTGAAT

AAGAGTAAAAGTTTAATTATAGATTAAAGGAAAATAATAGGGACCAAATGGGCAATTAAG

CCACATTTGGAAGTTGAGGCGGCATAACATTGTAAAAATCTTAGATTTTTATATTATTAT

TTATATAAATATATAAATTAATTATAAAGTATATTATTAAATTAATTATATTATAAATAT

TATATTATTATATATAAAAGAAACAAAACAGAAAAGAAACAGAATAGAAAGAACAAAGAA

ACAGAATAGAAGAGACGAAACAGGGGAGAAGCAGGGGAGAAAGAAGAAAAAGAAGAAAAA

AGGGGAAATAGGGTTTTTGAAGCTTGAAATTTAAATTGGTAAGTCAAATTAGCCATTTTC

TCTTAATTCTAATGTTTTAAAAGCTTTAAAACAAAGTTTTGATGGAATTAAGTTGATATT

TTGTAAGTTCATAGGTTTTCAAGTATAGTTTATGTTGAACAAAAGAGATGAATTAGGGAT

TAACTTGAAGGAATTTTAAGTTAGAATTGAAAAAGGGATTAAATTGTAAAAGAAACTATA

AGTTTTTTTTGTTTTAGGGACTAGATTGAGGAAAATTCGGAATTAAGAAAATATGTTAAA

AATTTAATAGTTAAATTTGAGTTTAAATGAAATTTGAATAGGAATAAGGTGTGAATTGGT

GTTATAAATTTGGTTATTAACATTTTTAATCAAAACAGTTTTGGGAAGTAGCAATGGTCT

GACTTTGAAAATTCACTAAAAATTTTATAAATTGAACTAGAGGATGAACAAAATATGGAA

TTAAAGCTTATTGAGTCTAGTTTCTTATAGTAGAAACAATGTAAGCAATTAATTGATGAA

TCAAGAGATATTTGAAATTTTGTAATACTGGTTCGGGGTGATTTCGAGATGCCCTGTTTT

AACTTTGGAAAATCATTAAAAATTGTACAAAAATTATTATGGAGTGTAATTTATATATGT

GAACTCCTTAATGAATCTAGTTTCAAAATAAATAAACAAGAACCTTATTCGAGTTCTGTA

CAATGAGATAATTTAGTTTTAGTGGAGAGAGGTCAGAACTGTCAAATGAAATAACAGGGG

AGTATTTAACGAATAAACTGTATTAAATGGCTAGACCAAAAATTCTGGAAATTTTATGAT

TAGAAGATATATGAGTCTAGTTTTAAGGAAAATTTACGGATATTAATTTGGAGTTTCGTA

GCTCAAGATATAAATAATTTAGTAACAATGACCCAAGTAGACAGCTTAATGGTGAAATTA

TATAAATACATTAAAAATGGTTAAATTTGCATGTTTAGGCTCATGAATTAAATTGAATCA

TGTTGTATTGATTATTATAAATTATTATTTTCGTAGCCAACAAAGAACCTAAAGCATCAG

CATCGAAAGGAAAGGAGAAAGTCATCGAGGAGTAAACTCGAGAAAATTACGGTTTGTATT

ACTATAATTCAAGTTATTTATTATTAAATGTTAAATTTTAATTTATGTGTCTAGTAAATG

AAATGTGAGGTAAGTATTATTATTATTATTATTATTATTATTATTATTATTATTATTATT

ATGAGTGGGAATTAAATTGAATAGTTGATATGAAATAATATTTGAATTGTTTGTTGATTG

AAAGCGGGAAATGAATTTAAATCGAATAGTGACCGATATTAAATTGAATGGAAATGTATT

GAGTTGTGAAAATATGTTAATTGCGGATTAATTATTGATTGAAAGGTGGAAAAATGATTG

AATTGAAAGTGTGAGAAAGTGTGATTGAATTGGGATTATATGTGATTTAAATACCCTATT

AACTAGTCGGGCTGAGTCGGATATAGTTGGCATGCCATAGGATTGGAAGAGTTCAGGGAT

ACTTCGACCTCGAGTCGATGAGACACTGGGTGATTTCTTCGGATAGATTGGATGAGGTAC

TGGGTACCAACTTTCTTCGGCTTTGCCGATGAGACACTGGGTGTCAACTATTGCTTCGAA

CTATCCGATGAGGCACTGGGTGCCATTCTGGTGTGTTTGGTTGGATCCGTGTATCCGCCA

AAGTCCGAGTTTTGTTAATAGGGTAAATGATGAAATGATAAACCGAACGAGTTGGTCAAA

CGAGCTATTGAAATGATATGAAAAAGTTGAATTGTGAATTGAAATGTGAAATGAGATTGA

GAAATGAACCTAAGGTTCGTGAATTATTCAAACTCAAATTGTGGATATACGATATTGGTT

GATGAATTGCTATTGTTGAAATATTTAATTTAAATTGTATATACGATTTATGCTTTACAT

GTACATTATTGTTATAATTTGAATTATGGTAATACCACTGAGTATGAATTACTCAGCGTA

CGGTTGTTTCCGTGCGCAGGTCAATAGAAGTCAAAGGTCTCGGTTCAGCATCCAGATTAA

TCCCGGCTTCGGCAAAACTTGGTGATGTATTTTTCCTTTGGTAAAGGTGGCATGTACATA

GATTGTGTATAAAGGTTATTATGTTTTATTATATAATGGTTAAAAATGTTAGTATTAAAA

GTTTATGGATTTTAATGAAAGAAGTCTATCTATTTTATCTAATTAGTACATTGTTAAATT

TTAAATTGGTATTGTGTAGATTGAGTTTGATTAGAAGTATTTAGAATAGAAAATGTGAAT

GTGAAATGAATTGGTTGAATTGATGATATTTGGGAACTATATGGTTTTAATTTGCAGGGG

GTTTTATGTAAAAATAAGCAGAAATGCTGCCGAAATTTTTATAAAAAAAAATGAAGTCAT

TTGGTAAACAAATTAATAAATTTTATGAATTATTTTAATATATTGGTTATTTATTTAAGA

ATTGTTGTAAATCGTTCGATACGTCCGGTAGTGCCTCGTAATTCTGTTCCGGCGACGGTT

CGGGGTTAAGGGGTGTTACATTTTATGGTATCAGAGCTATCAGGTTTAGCCGATTCTCGG

CCTAAATCGAGCTCGGAATTGAGTCTAGATGTACATGCCACTGTCGAGTTAAACTGAGTC

GGGATTTTTGGATGCTGACCTATTTGTTTGTTTTGTTTTATAGATTAAAGATGTCTGAAG

AAAGAATAAATGATACTGATGAAAGAATGTATAGTGAAGATAGAGAATTAGATGAAACAG

AATCTGTTGCACCGAGTGTGAATCCGTTAGGCAACCAACCTTCTAATGTAGAACGAGAAA

ATGTCAGAGATAGAGATGAATCCCAATTACTGAGAATTATAGCTGATGCATTACAAAGAG

TAGCAGGAACTACTCCTGTTACGACTTCAGTACCTACTGTTAGACGGGCTCCGATAAAGG

AACTGAGGAAATATGGTGCCACTGAATTTATGGGTCTAAAAGGAGTTGATCCATCCATAG

CTGAAAATTGGATGGAGTCGACTAAAAGAATTTTGCAGCAATTGGATTGTACCCCCCGAG

AGTGTTTAATCTGTGCCGTATCGTTATTACAAGGGGAGGCTTATCTATGGTGGGAATCAG

TGGTTCGACATTTACCAGAGAGTCAGATAACGTGGGATCTATTTCAGAAGGAGTTTCAAA

AGAAATATATCGGAGAGATGTATATTGAAGACAAGAAACAAGAGTTTTTGTTGCTACAAC

AGGGTGATATGTCAGTAATAGATTATGAGAGGGAATTCTCGAGACTCAGTAGATATGCCT

CCGAGTTTATTCCGACAGAAGCCGATAGTTGTAAAAGATTTTTACGGGGTTTACGAGACG

AGATCAAAGTGCAGCTAGTATCCCATCGGATCACTGAGTTAGTAGATTTGATTGAACGAG

CTAAAATGGTGGAACAAGTTCTGGGCCTCGACAAAAAGACTGAAGTTGTTAGACCAACCG

GGAAGCGTACAGGAACTACCAGTTCGAATCCTCAGCCGAAAAGACCAAAGGAATTCCAAA

GTGGTTGGAGATCCAGTTTCAGGTCAGACAGAGGTGGTAGAAATAGGGGAAAACAGACGA

TGACATCTACTGGCAGTGTGAAAGGTCCTTCCCGAGAAATAGATATTCCAGACTGCCAAC

ACTGCGGAAAGAAACACAGAGGGGAATGTTGGAAATTAACTAGAGGCTGTTTTCGATGTG

GTTCTACAGACCATTTCATCAGAGACTGTCCGAAAGTTGATAGTACTGTACCCGTGACAT

CACAGAGATCGGTATCTACAGCTAGAGGCAGAGGGTTAGGAAGAGGTGGTTCGGTTTCAA

GGGGAGGAAGTATTAGGAGAAGCAATGATATTGCTACTCAGCAGTCTGAGGCTAAAGTAC

CTGCCAGAGCTTATGTGGTCAGAACACAGGAAGAAGGTGACGCCCACGATGTAGTAACAG

GTATATTCTTACTATATTCTGAGCCTGTTTATGCTTTAATTGATCCCGGATCTTCACATT

CTTATATAAATTCAAAATTAGTTGAATTGGGAAAATTTAATTCTGAAATATCTAGAGTGA

CTGTAGAAGTGTCGAGTCCGTTGGGGCAAACAGTATTAGTGAATCAGATCTGTCCGAGAT

GCCCGTTAATTATACAAAATAAAACTTTTCCTATTGACCTGTTGATTATGCCATTTGGAG

ATTTTGATATAATACTGGGGATGGATTGGTTGGCTGAGCACGGAGTGGTATTGGATTGTT

ATAAAAAGAAGTTTAGTATTCAGACAGAAGACGGGGACAGAATTGAAGTAAATGGTATCC

GTACTAATGGGCCGACACGTATTATTTCGGCAATAAAGGCTAATAAATTGCTTCAGCGGG

GTTGTACAGCGTATTTAGCCTATGTTATTAATTCTGATTTGGTTGGTAGTCAGTGCAGTA

AGATTAGAACCGTATGTGAGTTTCCAGATGTATTTCCTGAAGAGCTACCGGGTTTACCAC

CTGACAGAGAGGTTGAATTTGCTATAGAAGTGTATCCGGGTACAGCACCAATCTCTATAC

CACCGTATCGAATGTCACCCACTGAGTTGAAAGAGTTGAAAGTGCAGTTACAGGACTTGT

CAGATCGTGGATTTATTAGACCGAGCATCTCACCTTGGGGAGCTCCAGTATTGTTTGTTA

AAAAGAAAGATGGATCGATGCGGCTTTGTATTGATTACCGGCAGTTAAACAAAGTGACGA

TCAAGAACCGGTATCCGTTACCCCGTATAGATGATTTATTTGATCAACTAAAAGGAGCTT

CAGTATTTTCAAAGATTGACTTAAGATCTGGGTATTATCAGCTGAAGGTAAAAGAAAGTG

ATGTTCCGAAGACTGCATTTCGTACTCGATATGGTCATTATGAATTTTTGGTGATGCCGT

TCGGGTTGACTAATGCTCCAGCTGCTTTTATGGATCTGATGAATCGTATTTTTCAGCCGT

ATTTAGATCAGTTTGTGGTGGTTTTTATTGATGACATCTTGGTTTATTCGAAGTCAGAGT

CAGAGCATGATCAGCATCTCAGAACCGTGCTACAAATTCTGCGAGAAAAACAGTTGTACG

GGAAACTAAGTAAATGTGAATTCTGGTTATCAGAGGTAGTATTCTTGGGACATGTTGTAT

CTGCGGATGGGATTAGAGTTGATCCGAAGAAGATCGAGGCAATTGTTCAATGGAAGGCAC

CAAAGAATGTATCAGAGGTACGCAGTTTTCTTGGTTTGGCTGGGTATTACAGAAGATTTG

TAAATGGGTTTTCGAAGATAGCTTTGCCGATGACCAAATTACTACAGAAGAATGTTCCAT

TTATCTGGGATGATCAGTGTCAGAGGAGCTTTGAAACATTGAAACAGATGTTGACAGAGG

CACCAGTTTTAACTTTACCAGAATCAGGGAAAGATTTCATAGTGTACAGTGATGCTTCTT

TGAATGGTTTGGGTTGTGTATTGATGCAAGAAGGAAAAGTAATAGCTTATGCATCTCGAC

AGTTGAAGTCACATGAACGCAACTACCCGACACACGATTTAGAGTTAGCTGCTGTAATCT

TTGCATTGAAGATTTGGATACATTACTTGTATGGTGAGAAATGTTATATTTACACTGATC

ATAAAAGTCTAAAATATCTTCTGTCACAAAAGGAGTTGAATCTGAGACAGAGACGGTGGA

TTGAACTTCTGAAAGATTATGATTGTGTTATAGATTATCATCCAGGGAAGGCAAATGTGG

TAGCAGATGCATTGAGTAGAAAAGCAGCGATTGAATTACGAGCAATGTTCGCTCGACTTA

GTATTAAGGATGATGGAAGTTTGTTAGCTGAGTTAAGAGTCAAGCCGGTGATGTTTGATC

AAATCAGAGCAGCACAGTTAAAAGATGAAAAGTTGATGAGGAAAAGAGAAATGGTACAGT

ATGGTGCGGTAGAAAATTTTAGTATTGACGAGCATGATTGTTTGAGATTTCGAAATCGAA

TTTGTGTTCCATCTACTTCTGAGATTAAAGAATTGATTCTCCGAGAAGCACATAATAGTA

TTTTTGCTTTGCACCCAGGAGGAACGAAGATGTATCGTGATCTACGAGAACTGTATTGGT

GGCCAGGAATGAAGAAAGATATAGTTGAATATGTCAGTAAATGCTTGACTTGTCAGCGGG

TAAAAGCAGAACATCAGGTACCAACAGGCCTGTTACAGCCTATTACTATTCCCGAGTGGA

AATGGGATCGCATTACCATGGATTTTGTTACGGGGTTGCCATTGTCAGTGAGTAAAAAGA

ATGCTATTTGGGTGATTGTTGATCGACTCACAAAATCAGCTCATTTTATAGCAGTTAGAA

CCGACTGGTCATTACAGAAGCTTGCCGAGGTTTATATTCGAGAAATTGTTAGATTACATG

GTATTCCGGTATCAATAATTTCAGACAGAGATCCTCGATTCACTTCGAGATTTTGGAAGC

AGCTGCATGAATCATTGGGTACTCGACTTAGTTTCAGTACAGCTTTTCATCCTCAAACTG

ATGGACAATCTGAACGAGTAATTCAGATATTAGAAGATATGCTTCGAGCTTGTGTCATTG

ATTTTGAATCAGGTTGGGAACGTTATTTACCATTGGCCGAGTTTGTTTATAATAATAGTT

TCCAATCTAGTATTCAAATGGCTCCATATGAAGCACTTTATGGTCGAAGGTGTCGATCAC

CAATATGTTGGACAAAATTAAGAGAAAGAAAAGTGATTGGGCCGGAATTGATTCAAGAGA

CAGAAGAAACAGTTAAAAAGATTAAAGATAGACTGAAAGCCGCTTTCGACAGACAGAAAT

CTTACGCAGACTTGAAACGACGAGACATTGAATATTCCGTTGGTGATAAGGTATTCCTCA

AAGTATCGCCGTGGAAGAAAATTTTGAGATTTGGTCGGAAGGGAAAATTAAGTCCGCGCT

TTATTGGGCCGTATGAGATAGTGGAAAGAATTGGGCCTGTTGCTTATCGATTATCCTTAC

CTCCAGAGTTACAGAAAATTCATGATGTTTTTCATGTTTCGATGCTTCGGAGATATAGAT

CGGATCCTTCTCATGTTATTCCCACTGAAGACATTGAACTTCGATCTGATTTAACTTATG

AAGAAGAACCAGTTCAAATATTAGCACGAGAAGTGAAAGAATTAAGAAATAAACGGGTTC

CTTTAGTACAAGTTTTATGGAGAAGCCATAGTGTGGAAGAAGCAACTTGGGAACCGGAAG

AGACAATGAGAGCACAATATCCTCATCTCTTCTCAGGTAAATTTCGAGGACGAAATTTAT

TAAGAGGGGGAGAAATGTAATGACCTAAAATTCATGGGCATCGGAAAAGTATAATATTGG

GCCTCCGTCCTAGTAAATTGAGTCCGAAAATAATTATTAGAAATATTTACGAGACTAGTA

GTGTGTTTAATTAGGTTTTAATTAAGTAAATTTAGCTTAATTTAGAGTAATTAGTAAAAA

GGATTAAATTGAATAAGAGTAAAAGTTTAATTATAGATTAAAGGAAAATAATAGGGACCA

AATGGGCAATTAAGCCACATTTGGAAGTTGAGGCGGCATAACATTGTAAAAATCTTAGAT

TTTTATATTATTATTTATATAAATATATAAATTAATTATAAAGTATATTATTAAATTAAT

TATATTATAAATATTATATTATTATATATAAAAGAAACAAAACAGAAAAGAAACAGAATA

GAAAGAACAAAGAAACAGAATAGAAGAGACGAAACAGGGGAGAAGCAGGGGAGAAAGAAG

AAAAAGAAGAAAAAAGGGGAAATAGGGTTTTTGAAGCTTGAAATTTAAATTGGTAAGTCA

AATTAGCCATTTTCTCTTAATTCTAATGTTTTAAAAGCTTTAAAACAAAGTTTTGATGGA

ATTAAGTTGATATTTTGTAAGTTCATAGGTTTTCAAGTATAGTTTATGTTGAACAAAAGA

GATGAATTAGGGATTAACTTGAAGGAATTTTAAGTTAGAATTGAAAAAGGGATTAAATTG

TAAAAGAAACTATAAGTTTTTTTTGTTTTAGGGACTAGATTGAGGAAAATTCGGAATTAA

GAAAATATGTTAAAAATTTAATAGTTAAATTTGAGTTTAAATGAAATTTGAATAGGAATA

AGGTGTGAATTGGTGTTATAAATTTGGTTATTAACATTTTTAATCAAAACAGTTTTGGGA

AGTAGCAATGGTCTGACTTTGAAAATTCACTAAAAATTTTATAAATTGAACTAGAGGATG

AACAAAATATGGAATTAAAGCTTATTGAGTCTAGTTTCTTATAGTAGAAACAATGTAAGC

AATTAATTGATGAATCAAGAGATATTTGAAATTTTGTAATACTGGTTCGGGGTGATTTCG

AGATGCCCTGTTTTAACTTTGGAAAATCATTAAAAATTGTACAAAAATTATTATGGAGTG

TAATTTATATATGTAAACTCCTTAATGAATCTAGTTTCAAAATAAATAAACAAGAACCTT

ATTCGAGTTCTGTACAATGAGATAATTTAGTTTTAGTGGAGAGAGGTCAGAACTGTCAAA

TGAAATAACAGGGGAGTATTTAACGAATAAACTGTATTAAATGGCTAGACCAAAAATTCT

GGAAATTTTATGATTAGAAGATATATGAGTCTAGTTTTAAGGAAAATTTACGGATATTAA

TTTGGAGTTTCGTAGCTCAAGATATAAATAATTTAGTAACAATGACCCAAGTAGACAGCT

TAATGGTGAAATTATATAAATACATTAAAAATGGTTAAATTTGCATGTTTAGGCTCATGA

ATTAAATTGAATCATGTTGTATTGATTATTATAAATTATTATTTTCGTAGCCAACAAAGA

ACCTAAAGCATCAGCATCGAAAGGAAAGGAGAAAGTCATCGAGGAGTAAACTCGAGAAAA

TTACGGTTTGTATTACTATAATTCAAGTTATTTATTATTAAATGTTAAATTTTAATTTAT

GTGTCTAGTAAATGAAATGTGAGGTAAGTATTATTATTATTATTATTATTATTATTATTA

TTATTATTATTATTATTATGAGTGGGAATTAAATTGAATAGTTGATATGAAATAATATTT

GAATTGTTTGTTGATTGAAAGCGGGAAATGAATTTAAATCGAATAGTGACCGATATTAAA

TTGAATGGAAATGTATTGAGTTGTGAAAATATGTTAATTGCGGATTAATTATTGATTGAA

AGGTGGAAAAATGATTGAATTGAAAGTGTGAGAAAGTGTGATTGAATTGGGATTATATGT

GATTTAAATACCCTATTAACTAGTCGGGCTGAGTCGGATATAGTTGGCATGCCATAGGAT

TGGAAGAGTTCAGGGATACTTCGACCTCGAGTCGATGAGACACTGGGTGTCACTATATTT

CTTCGGATAGATTCGATGAGGTACTGGGTACCAACTTTCTTCGGCTTTGCCGATGAGACA

CTGGGTGTCAACTATTGCTTCGAACTATCCGATGAGGCACTGGGTGCCATTCTGGTGTGT

TTGGTTGGATCCGTGTATTCGCCAAAGTCCGAGTTTTGTTAATAGGGTAAATGATGAAAT

GATAAACCGAACGAGTTGGTCAAACGAGCTATTGAAATGATATGAAAAAGTTGAATTGTG

AATTGAAATGTGAAATGAGATTGAGAAATGAACCTAAGGTTCGTGAATTATTCAAACTCA

AATTGTGGATATACGATATTGGTTGATGAATTGCTATTGTTGAAATATTTAATTTAAATT

GTATATACGATTTATGCTTTACATGTACATTATTGTTATAATTTGAATTATGGTAATACC

ACTGAGTATGAATTACTCAGCGTACGGTTGTTTCCGTGCGCAGGTCAATAGAAGTCAAAG

GTCTCGGTTCAGCATCCAGATTAATCCCGGCTTCGGCAAAACTTGGTGATGTATTTTTCC

TTTGGTAAAGGTGGCATGTACATAGATTGTGTATAAAGGTTATTATGTTTTATTATATAA

TGGTTAAAAATGTTAGTATTAAAAGTTTATGGATTTTAATGAAAGAAGTCTATCTATTTT

ATCTAATTAGTACATTGTTAAATTTTAAATTGGTATTAGATTGAGTTTGATTAGAAGTAT

TTAGAATAGAAAATGTGAATGTGAAATGAATTGGTTGAATTGATGATATTTGGGAACTAT

ATGGTTTTAATTTGC

>Deltapine41

ATGTAATGACCCAAAATTCATGGGCATCGGAAAAGTATAATATCGGGCCTCCGTCCTAGT

AAATTGAGTCCGAAAATAATTATTAGAAATATTTACGAGACTAGTAGTGTGTTTAATTAG

GTTTTAATTAAGTAAATTTAGCTTAATTTAGAGTAATTAGTAAAAAGGATTAAATTGAAT

AAGAGTAAAAGTTTAATTATAGATTAAAGGAAAATAATAGGGACCAAATGGGCAATTAAG

CCACATTTGGAAGTTGAGGCGGCATAACATTGTAAAAATCTTAGATTTTTATATTATTAT

TTATATAAATATATAAATTAATTATAAAGTATATTATTAAATTAATTATATTATAAATAT

TATATTATTATATATAAAAGAAACAAAACAGAAAAGAAACAGAATAGAAAGAACAAAGAA

ACAGAATAGAAGAGACGAAACAGGGGAGAAGCAGGGGAGAAAGAAGAAAAAGAAGAAAAA

AGGGGAAATAGGGTTTTTGAAGCTTGAAATTTAAATTGGTAAGTCAAATTAGCCATTTTC

TCTTAATTCTAATGTTTTAAAAGCTTTAAAACAAAGTTTTGATGGAATTAAGTTGATATT

TTGTAAGTTCATAGGTTTTCAAGTATAGTTTATGTTGAACAAAAGAGATGAATTAGGGAT

TAACTTGAAGGAATTTTAAGTTAGAATTGAAAAAGGGATTAAATTGTAAAAGAAACTATA

AGTTTTTTTTGTTTTAGGGACTAGATTGAGGAAAATTCGGAATTAAGAAAATATGTTAAA

AATTTAATAGTTAAATTTGAGTTTAAATGAAATTTGAATAGGAATAAGGTGTGAATTGGT

GTTATAAATTTGGTTATTAACATTTTTAATCAAAACAGTTTTGGGAAGTAGCAATGGTCT

GACTTTGAAAATTCACTAAAAATTTTATAAATTGAACTAGAGGATGAACAAAATATGGAA

TTAAAGCTTATTGAGTCTAGTTTCTTATAGTAGAAACAATGTAAGCAATTAATTGATGAA

TCAAGAGATATTTGAAATTTTGTAATACTGGTTCGGGGTGATTTCGAGATGCCCTGTTTT

AACTTTGGAAAATCATTAAAAATTGTACAAAAATTATTATGGAGTGTAATTTATATATGT

GAACTCCTTAATGAATCTAGTTTCAAAATAAATAAACAAGAACCTTATTCGAGTTCTGTA

CAATGAGATAATTTAGTTTTAGTGGAGAGAGGTCAGAACTGTCAAATGAAATAACAGGGG

AGTATTTAACGAATAAACTGTATTAAATGGCTAGACCAAAAATTCTGGAAATTTTATGAT

TAGAAGATATATGAGTCTAGTTTTAAGGAAAATTTACGGATATTAATTTGGAGTTTCGTA

GCTCAAGATATAAATAATTTAGTAACAATGACCCAAGTAGACAGCTTAATGGTGAAATTA

TATAAATACATTAAAAATGGTTAAATTTGCATGTTTAGGCTCATGAATTAAATTGAATCA

TGTTGTATTGATTATTATAAATTATTATTTTCGTAGCCAACAAAGAACCTAAAGCATCAG

CATCGAAAGGAAAGGAGAAAGTCATCGAGGAGTAAACTCGAGAAAATTACGGTTTGTATT

ACTATAATTCAAGTTATTTATTATTAAATGTTAAATTTTAATTTATGTGTCTAGTAAATG

AAATGTGAGGTAAGTATTATTATTATTATTATTATTATTATTATTATTATTATTATTATT

ATGAGTGGGAATTAAATTGAATAGTTGATATGAAATAATATTTGAATTGTTTGTTGATTG

AAAGCGGGAAATGAATTTAAATCGAATAGTGACCGATATTAAATTGAATGGAAATGTATT

GAGTTGTGAAAATATGTTAATTGCGGATTAATTATTGATTGAAAGGTGGAAAAATGATTG

AATTGAAAGTGTGAGAAAGTGTGATTGAATTGGGATTATATGTGATTTAAATACCCTATT

AACTAGTCGGGCTGAGTCGGATATAGTTGGCATGCCATAGGATTGGAAGAGTTCAGGGAT

ACTTCGACCTCGAGTCGATGAGACACTGGGTGATTTCTTCGGATAGATTGGATGAGGTAC

TGGGTACCAACTTTCTTCGGCTTTGCCGATGAGACACTGGGTGTCAACTATTGCTTCGAA

CTATCCGATGAGGCACTGGGTGCCATTCTGGTGTGTTTGGTTGGATCCGTGTATCCGCCA

AAGTCCGAGTTTTGTTAATAGGGTAAATGATGAAATGATAAACCGAACGAGTTGGTCAAA

CGAGCTATTGAAATGATATGAAAAAGTTGAATTGTGAATTGAAATGTGAAATGAGATTGA

GAAATGAACCTAAGGTTCGTGAATTATTCAAACTCAAATTGTGGATATACGATATTGGTT

GATGAATTGCTATTGTTGAAATATTTAATTTAAATTGTATATACGATTTATGCTTTACAT

GTACATTATTGTTATAATTTGAATTATGGTAATACCACTGAGTATGAATTACTCAGCGTA

CGGTTGTTTCCGTGCGCAGGTCAATAGAAGTCAAAGGTCTCGGTTCAGCATCCAGATTAA

TCCCGGCTTCGGCAAAACTTGGTGATGTATTTTTCCTTTGGTAAAGGTGGCATGTACATA

GATTGTGTATAAAGGTTATTATGTTTTATTATATAATGGTTAAAAATGTTAGTATTAAAA

GTTTATGGATTTTAATGAAAGAAGTCTATCTATTTTATCTAATTAGTACATTGTTAAATT

TTAAATTGGTATTGTGTAGATTGAGTTTGATTAGAAGTATTTAGAATAGAAAATGTGAAT

GTGAAATGAATTGGTTGAATTGATGATATTTGGGAACTATATGGTTTTAATTTGCAGGGG

GTTTTATGTAAAAATAAGCAGAAATGCTGCCGAAATTTTTATAAAAAAAAATGAAGTCAT

TTGGTAAACAAATTAATAAATTTTATGAATTATTTTAATATATTGGTTATTTATTTAAGA

ATTGTTGTAAATCGTTCGATACGTCCGGTAGTGCCTCGTAATTCTGTTCCGGCGACGGTT

CGGGGTTAAGGGGTGTTACATTTTATGGTATCAGAGCTATCAGGTTTAGCCGATTCTCGG

CCTAAATCGAGCTCGGAATTGAGTCTAGATGTACATGCCACTGTCGAGTTAAACTGAGTC

GGGATTTTTGGATGCTGACCTATTTGTTTGTTTTGTTTTATAGATTAAAGATGTCTGAAG

AAAGAATAAATGATACTGATGAAAGAATGTATAGTGAAGATAGAGAATTAGATGAAACAG

AATCTGTTGCACCGAGTGTGAATCCGTTAGGCAACCAACCTTCTAATGTAGAACGAGAAA

ATGTCAGAGATAGAGATGAATCCCAATTACTGAGAATTATAGCTGATGCATTACAAAGAG

TAGCAGGAACTACTCCTGTTACGACTTCAGTACCTACTGTTAGACGGGCTCCGATAAAGG

AACTGAGGAAATATGGTGCCACTGAATTTATGGGTCTAAAAGGAGTTGATCCATCCATAG

CTGAAAATTGGATGGAGTCGACTAAAAGAATTTTGCAGCAATTGGATTGTACCCCCCGAG

AGTGTTTAATCTGTGCCGTATCGTTATTACAAGGGGAGGCTTATCTATGGTGGGAATCAG

TGGTTCGACATTTACCAGAGAGTCAGATAACGTGGGATCTATTTCAGAAGGAGTTTCAAA

AGAAATATATCGGAGAGATGTATATTGAAGACAAGAAACAAGAGTTTTTGTTGCTACAAC

AGGGTGATATGTCAGTAATAGATTATGAGAGGGAATTCTCGAGACTCAGTAGATATGCCT

CCGAGTTTATTCCGACAGAAGCCGATAGTTGTAAAAGATTTTTACGGGGTTTACGAGACG

AGATCAAAGTGCAGCTAGTATCCCATCGGATCACTGAGTTAGTAGATTTGATTGAACGAG

CTAAAATGGTGGAACAAGTTCTGGGCCTCGACAAAAAGACTGAAGTTGTTAGACCAACCG

GGAAGCGTACAGGAACTACCAGTTCGAATCCTCAGCCGAAAAGACCAAAGGAATTCCAAA

GTGGTTGGAGATCCAGTTTCAGGTCAGACAGAGGTGGTAGAAATAGGGGAAAACAGACGA

TGACATCTACTGGCAGTGTGAAAGGTCCTTCCCGAGAAATAGATATTCCAGACTGCCAAC

ACTGCGGAAAGAAACACAGAGGGGAATGTTGGAAATTAACTAGAGGCTGTTTTCGATGTG

GTTCTACAGACCATTTCATCAGAGACTGTCCGAAAGTTGATAGTACTGTACCCGTGACAT

CACAGAGATCGGTATCTACAGCTAGAGGCAGAGGGTTAGGAAGAGGTGGTTCGGTTTCAA

GGGGAGGAAGTATTAGGAGAAGCAATGATATTGCTACTCAGCAGTCTGAGGCTAAAGTAC

CTGCCAGAGCTTATGTGGTCAGAACACAGGAAGAAGGTGACGCCCACGATGTAGTAACAG

GTATATTCTTACTATATTCTGAGCCTGTTTATGCTTTAATTGATCCCGGATCTTCACATT

CTTATATAAATTCAAAATTAGTTGAATTGGGAAAATTTAATTCTGAAATATCTAGAGTGA

CTGTAGAAGTGTCGAGTCCGTTGGGGCAAACAGTATTAGTGAATCAGATCTGTCCGAGAT

GCCCGTTAATTATACAAAATAAAACTTTTCCTATTGACCTGTTGATTATGCCATTTGGAG

ATTTTGATATAATACTGGGGATGGATTGGTTGGCTGAGCACGGAGTGGTATTGGATTGTT

ATAAAAAGAAGTTTAGTATTCAGACAGAAGACGGGGACAGAATTGAAGTAAATGGTATCC

GTACTAATGGGCCGACACGTATTATTTCGGCAATAAAGGCTAATAAATTGCTTCAGCGGG

GTTGTACAGCGTATTTAGCCTATGTTATTAATTCTGATTTGGTTGGTAGTCAGTGCAGTA

AGATTAGAACCGTATGTGAGTTTCCAGATGTATTTCCTGAAGAGCTACCGGGTTTACCAC

CTGACAGAGAGGTTGAATTTGCTATAGAAGTGTATCCGGGTACAGCACCAATCTCTATAC

CACCGTATCGAATGTCACCCACTGAGTTGAAAGAGTTGAAAGTGCAGTTACAGGACTTGT

CAGATCGTGGATTTATTAGACCGAGCATCTCACCTTGGGGAGCTCCAGTATTGTTTGTTA

AAAAGAAAGATGGATCGATGCGGCTTTGTATTGATTACCGGCAGTTAAACAAAGTGACGA

TCAAGAACCGGTATCCGTTACCCCGTATAGATGATTTATTTGATCAACTAAAAGGAGCTT

CAGTATTTTCAAAGATTGACTTAAGATCTGGGTATTATCAGCTGAAGGTAAAAGAAAGTG

ATGTTCCGAAGACTGCATTTCGTACTCGATATGGTCATTATGAATTTTTGGTGATGCCGT

TCGGGTTGACTAATGCTCCAGCTGCTTTTATGGATCTGATGAATCGTATTTTTCAGCCGT

ATTTAGATCAGTTTGTGGTGGTTTTTATTGATGACATCTTGGTTTATTCGAAGTCAGAGT

CAGAGCATGATCAGCATCTCAGAACCGTGCTACAAATTCTGCGAGAAAAACAGTTGTACG

GGAAACTAAGTAAATGTGAATTCTGGTTATCAGAGGTAGTATTCTTGGGACATGTTGTAT

CTGCGGATGGGATTAGAGTTGATCCGAAGAAGATCGAGGCAATTGTTCAATGGAAGGCAC

CAAAGAATGTATCAGAGGTACGCAGTTTTCTTGGTTTGGCTGGGTATTACAGAAGATTTG

TAAATGGGTTTTCGAAGATAGCTTTGCCGATGACCAAATTACTACAGAAGAATGTTCCAT

TTATCTGGGATGATCAGTGTCAGAGGAGCTTTGAAACATTGAAACAGATGTTGACAGAGG

CACCAGTTTTAACTTTACCAGAATCAGGGAAAGATTTCATAGTGTACAGTGATGCTTCTT

TGAATGGTTTGGGTTGTGTATTGATGCAAGAAGGAAAAGTAATAGCTTATGCATCTCGAC

AGTTGAAGTCACATGAACGCAACTACCCGACACACGATTTAGAGTTAGCTGCTGTAATCT

TTGCATTGAAGATTTGGATACATTACTTGTATGGTGAGAAATGTTATATTTACACTGATC

ATAAAAGTCTAAAATATCTTCTGTCACAAAAGGAGTTGAATCTGAGACAGAGACGGTGGA

TTGAACTTCTGAAAGATTATGATTGTGTTATAGATTATCATCCAGGGAAGGCAAATGTGG

TAGCAGATGCATTGAGTAGAAAAGCAGCGATTGAATTACGAGCAATGTTCGCTCGACTTA

GTATTAAGGATGATGGAAGTTTGTTAGCTGAGTTAAGAGTCAAGCCGGTGATGTTTGATC

AAATCAGAGCAGCACAGTTAAAAGATGAAAAGTTGATGAGGAAAAGAGAAATGGTACAGT

ATGGTGCGGTAGAAAATTTTAGTATTGACGAGCATGATTGTTTGAGATTTCGAAATCGAA

TTTGTGTTCCATCTACTTCTGAGATTAAAGAATTGATTCTCCGAGAAGCACATAATAGTA

TTTTTGCTTTGCACCCAGGAGGAACGAAGATGTATCGTGATCTACGAGAACTGTATTGGT

GGCCAGGAATGAAGAAAGATATAGTTGAATATGTCAGTAAATGCTTGACTTGTCAGCGGG

TAAAAGCAGAACATCAGGTACCAACAGGCCTGTTACAGCCTATTACTATTCCCGAGTGGA

AATGGGATCGCATTACCATGGATTTTGTTACGGGGTTGCCATTGTCAGTGAGTAAAAAGA

ATGCTATTTGGGTGATTGTTGATCGACTCACAAAATCAGCTCATTTTATAGCAGTTAGAA

CCGACTGGTCATTACAGAAGCTTGCCGAGGTTTATATTCGAGAAATTGTTAGATTACATG

GTATTCCGGTATCAATAATTTCAGACAGAGATCCTCGATTCACTTCGAGATTTTGGAAGC

AGCTGCATGAATCATTGGGTACTCGACTTAGTTTCAGTACAGCTTTTCATCCTCAAACTG

ATGGACAATCTGAACGAGTAATTCAGATATTAGAAGATATGCTTCGAGCTTGTGTCATTG

ATTTTGAATCAGGTTGGGAACGTTATTTACCATTGGCCGAGTTTGTTTATAATAATAGTT

TCCAATCTAGTATTCAAATGGCTCCATATGAAGCACTTTATGGTCGAAGGTGTCGATCAC

CAATATGTTGGACAAAATTAAGAGAAAGAAAAGTGATTGGGCCGGAATTGATTCAAGAGA

CAGAAGAAACAGTTAAAAAGATTAAAGATAGACTGAAAGCCGCTTTCGACAGACAGAAAT

CTTACGCAGACTTGAAACGACGAGACATTGAATATTCCGTTGGTGATAAGGTATTCCTCA

AAGTATCGCCGTGGAAGAAAATTTTGAGATTTGGTCGGAAGGGAAAATTAAGTCCGCGCT

TTATTGGGCCGTATGAGATAGTGGAAAGAATTGGGCCTGTTGCTTATCGATTATCCTTAC

CTCCAGAGTTACAGAAAATTCATGATGTTTTTCATGTTTCGATGCTTCGGAGATATAGAT

CGGATCCTTCTCATGTTATTCCCACTGAAGACATTGAACTTCGATCTGATTTAACTTATG

AAGAAGAACCAGTTCAAATATTAGCACGAGAAGTGAAAGAATTAAGAAATAAACGGGTTC

CTTTAGTACAAGTTTTATGGAGAAGCCATAGTGTGGAAGAAGCAACTTGGGAACCGGAAG

AGACAATGAGAGCACAATATCCTCATCTCTTCTCAGGTAAATTTCGAGGACGAAATTTAT

TAAGAGGGGGAGAAATGTAATGACCTAAAATTCATGGGCATCGGAAAAGTATAATATTGG

GCCTCCGTCCTAGTAAATTGAGTCCGAAAATAATTATTAGAAATATTTACGAGACTAGTA

GTGTGTTTAATTAGGTTTTAATTAAGTAAATTTAGCTTAATTTAGAGTAATTAGTAAAAA

GGATTAAATTGAATAAGAGTAAAAGTTTAATTATAGATTAAAGGAAAATAATAGGGACCA

AATGGGCAATTAAGCCACATTTGGAAGTTGAGGCGGCATAACATTGTAAAAATCTTAGAT

TTTTATATTATTATTTATATAAATATATAAATTAATTATAAAGTATATTATTAAATTAAT

TATATTATAAATATTATATTATTATATATAAAAGAAACAAAACAGAAAAGAAACAGAATA

GAAAGAACAAAGAAACAGAATAGAAGAGACGAAACAGGGGAGAAGCAGGGGAGAAAGAAG

AAAAAGAAGAAAAAAGGGGAAATAGGGTTTTTGAAGCTTGAAATTTAAATTGGTAAGTCA

AATTAGCCATTTTCTCTTAATTCTAATGTTTTAAAAGCTTTAAAACAAAGTTTTGATGGA

ATTAAGTTGATATTTTGTAAGTTCATAGGTTTTCAAGTATAGTTTATGTTGAACAAAAGA

GATGAATTAGGGATTAACTTGAAGGAATTTTAAGTTAGAATTGAAAAAGGGATTAAATTG

TAAAAGAAACTATAAGTTTTTTTTGTTTTAGGGACTAGATTGAGGAAAATTCGGAATTAA

GAAAATATGTTAAAAATTTAATAGTTAAATTTGAGTTTAAATGAAATTTGAATAGGAATA

AGGTGTGAATTGGTGTTATAAATTTGGTTATTAACATTTTTAATCAAAACAGTTTTGGGA

AGTAGCAATGGTCTGACTTTGAAAATTCACTAAAAATTTTATAAATTGAACTAGAGGATG

AACAAAATATGGAATTAAAGCTTATTGAGTCTAGTTTCTTATAGTAGAAACAATGTAAGC

AATTAATTGATGAATCAAGAGATATTTGAAATTTTGTAATACTGGTTCGGGGTGATTTCG

AGATGCCCTGTTTTAACTTTGGAAAATCATTAAAAATTGTACAAAAATTATTATGGAGTG

TAATTTATATATGTAAACTCCTTAATGAATCTAGTTTCAAAATAAATAAACAAGAACCTT

ATTCGAGTTCTGTACAATGAGATAATTTAGTTTTAGTGGAGAGAGGTCAGAACTGTCAAA

TGAAATAACAGGGGAGTATTTAACGAATAAACTGTATTAAATGGCTAGACCAAAAATTCT

GGAAATTTTATGATTAGAAGATATATGAGTCTAGTTTTAAGGAAAATTTACGGATATTAA

TTTGGAGTTTCGTAGCTCAAGATATAAATAATTTAGTAACAATGACCCAAGTAGACAGCT

TAATGGTGAAATTATATAAATACATTAAAAATGGTTAAATTTGCATGTTTAGGCTCATGA

ATTAAATTGAATCATGTTGTATTGATTATTATAAATTATTATTTTCGTAGCCAACAAAGA

ACCTAAAGCATCAGCATCGAAAGGAAAGGAGAAAGTCATCGAGGAGTAAACTCGAGAAAA

TTACGGTTTGTATTACTATAATTCAAGTTATTTATTATTAAATGTTAAATTTTAATTTAT

GTGTCTAGTAAATGAAATGTGAGGTAAGTATTATTATTATTATTATTATTATTATTATTA

TTATTATTATTATTATTATGAGTGGGAATTAAATTGAATAGTTGATATGAAATAATATTT

GAATTGTTTGTTGATTGAAAGCGGGAAATGAATTTAAATCGAATAGTGACCGATATTAAA

TTGAATGGAAATGTATTGAGTTGTGAAAATATGTTAATTGCGGATTAATTATTGATTGAA

AGGTGGAAAAATGATTGAATTGAAAGTGTGAGAAAGTGTGATTGAATTGGGATTATATGT

GATTTAAATACCCTATTAACTAGTCGGGCTGAGTCGGATATAGTTGGCATGCCATAGGAT

TGGAAGAGTTCAGGGATACTTCGACCTCGAGTCGATGAGACACTGGGTGTCACTATATTT

CTTCGGATAGATTCGATGAGGTACTGGGTACCAACTTTCTTCGGCTTTGCCGATGAGACA

CTGGGTGTCAACTATTGCTTCGAACTATCCGATGAGGCACTGGGTGCCATTCTGGTGTGT

TTGGTTGGATCCGTGTATTCGCCAAAGTCCGAGTTTTGTTAATAGGGTAAATGATGAAAT

GATAAACCGAACGAGTTGGTCAAACGAGCTATTGAAATGATATGAAAAAGTTGAATTGTG

AATTGAAATGTGAAATGAGATTGAGAAATGAACCTAAGGTTCGTGAATTATTCAAACTCA

AATTGTGGATATACGATATTGGTTGATGAATTGCTATTGTTGAAATATTTAATTTAAATT

GTATATACGATTTATGCTTTACATGTACATTATTGTTATAATTTGAATTATGGTAATACC

ACTGAGTATGAATTACTCAGCGTACGGTTGTTTCCGTGCGCAGGTCAATAGAAGTCAAAG

GTCTCGGTTCAGCATCCAGATTAATCCCGGCTTCGGCAAAACTTGGTGATGTATTTTTCC

TTTGGTAAAGGTGGCATGTACATAGATTGTGTATAAAGGTTATTATGTTTTATTATATAA

TGGTTAAAAATGTTAGTATTAAAAGTTTATGGATTTTAATGAAAGAAGTCTATCTATTTT

ATCTAATTAGTACATTGTTAAATTTTAAATTGGTATTAGATTGAGTTTGATTAGAAGTAT

TTAGAATAGAAAATGTGAATGTGAAATGAATTGGTTGAATTGATGATATTTGGGAACTAT

ATGGTTTTAATTTGC

>Deltapine45

ATGTAATGACCCAAAATTCATGGGCATCGGAAAAGTATAATATCGGGCCTCCGTCCTAGT

AAATTGAGTCCGAAAATAATTATTAGAAATATTTACGAGACTAGTAGTGTGTTTAATTAG

GTTTTAATTAAGTAAATTTAGCTTAATTTAGAGTAATTAGTAAAAAGGATTAAATTGAAT

AAGAGTAAAAGTTTAATTATAGATTAAAGGAAAATAATAGGGACCAAATGGGCAATTAAG

CCACATTTGGAAGTTGAGGCGGCATAACATTGTAAAAATCTTAGATTTTTATATTATTAT

TTATATAAATATATAAATTAATTATAAAGTATATTATTAAATTAATTATATTATAAATAT

TATATTATTATATATAAAAGAAACAAAACAGAAAAGAAACAGAATAGAAAGAACAAAGAA

ACAGAATAGAAGAGACGAAACAGGGGAGAAGCAGGGGAGAAAGAAGAAAAAGAAGAAAAA

AGGGGAAATAGGGTTTTTGAAGCTTGAAATTTAAATTGGTAAGTCAAATTAGCCATTTTC

TCTTAATTCTAATGTTTTAAAAGCTTTAAAACAAAGTTTTGATGGAATTAAGTTGATATT

TTGTAAGTTCATAGGTTTTCAAGTATAGTTTATGTTGAACAAAAGAGATGAATTAGGGAT

TAACTTGAAGGAATTTTAAGTTAGAATTGAAAAAGGGATTAAATTGTAAAAGAAACTATA

AGTTTTTTTTGTTTTAGGGACTAGATTGAGGAAAATTCGGAATTAAGAAAATATGTTAAA

AATTTAATAGTTAAATTTGAGTTTAAATGAAATTTGAATAGGAATAAGGTGTGAATTGGT

GTTATAAATTTGGTTATTAACATTTTTAATCAAAACAGTTTTGGGAAGTAGCAATGGTCT

GACTTTGAAAATTCACTAAAAATTTTATAAATTGAACTAGAGGATGAACAAAATATGGAA

TTAAAGCTTATTGAGTCTAGTTTCTTATAGTAGAAACAATGTAAGCAATTAATTGATGAA

TCAAGAGATATTTGAAATTTTGTAATACTGGTTCGGGGTGATTTCGAGATGCCCTGTTTT

AACTTTGGAAAATCATTAAAAATTGTACAAAAATTATTATGGAGTGTAATTTATATATGT

GAACTCCTTAATGAATCTAGTTTCAAAATAAATAAACAAGAACCTTATTCGAGTTCTGTA

CAATGAGATAATTTAGTTTTAGTGGAGAGAGGTCAGAACTGTCAAATGAAATAACAGGGG

AGTATTTAACGAATAAACTGTATTAAATGGCTAGACCAAAAATTCTGGAAATTTTATGAT

TAGAAGATATATGAGTCTAGTTTTAAGGAAAATTTACGGATATTAATTTGGAGTTTCGTA

GCTCAAGATATAAATAATTTAGTAACAATGACCCAAGTAGACAGCTTAATGGTGAAATTA

TATAAATACATTAAAAATGGTTAAATTTGCATGTTTAGGCTCATGAATTAAATTGAATCA

TGTTGTATTGATTATTATAAATTATTATTTTCGTAGCCAACAAAGAACCTAAAGCATCAG

CATCGAAAGGAAAGGAGAAAGTCATCGAGGAGTAAACTCGAGAAAATTACGGTTTGTATT

ACTATAATTCAAGTTATTTATTATTAAATGTTAAATTTTAATTTATGTGTCTAGTAAATG

AAATGTGAGGTAAGTATTATTATTATTATTATTATTATTATTATTATTATTATTATTATT

ATGAGTGGGAATTAAATTGAATAGTTGATATGAAATAATATTTGAATTGTTTGTTGATTG

AAAGCGGGAAATGAATTTAAATCGAATAGTGACCGATATTAAATTGAATGGAAATGTATT

GAGTTGTGAAAATATGTTAATTGCGGATTAATTATTGATTGAAAGGTGGAAAAATGATTG

AATTGAAAGTGTGAGAAAGTGTGATTGAATTGGGATTATATGTGATTTAAATACCCTATT

AACTAGTCGGGCTGAGTCGGATATAGTTGGCATGCCATAGGATTGGAAGAGTTCAGGGAT

ACTTCGACCTCGAGTCGATGAGACACTGGGTGATTTCTTCGGATAGATTGGATGAGGTAC

TGGGTACCAACTTTCTTCGGCTTTGCCGATGAGACACTGGGTGTCAACTATTGCTTCGAA

CTATCCGATGAGGCACTGGGTGCCATTCTGGTGTGTTTGGTTGGATCCGTGTATCCGCCA

AAGTCCGAGTTTTGTTAATAGGGTAAATGATGAAATGATAAACCGAACGAGTTGGTCAAA

CGAGCTATTGAAATGATATGAAAAAGTTGAATTGTGAATTGAAATGTGAAATGAGATTGA

GAAATGAACCTAAGGTTCGTGAATTATTCAAACTCAAATTGTGGATATACGATATTGGTT

GATGAATTGCTATTGTTGAAATATTTAATTTAAATTGTATATACGATTTATGCTTTACAT

GTACATTATTGTTATAATTTGAATTATGGTAATACCACTGAGTATGAATTACTCAGCGTA

CGGTTGTTTCCGTGCGCAGGTCAATAGAAGTCAAAGGTCTCGGTTCAGCATCCAGATTAA

TCCCGGCTTCGGCAAAACTTGGTGATGTATTTTTCCTTTGGTAAAGGTGGCATGTACATA

GATTGTGTATAAAGGTTATTATGTTTTATTATATAATGGTTAAAAATGTTAGTATTAAAA

GTTTATGGATTTTAATGAAAGAAGTCTATCTATTTTATCTAATTAGTACATTGTTAAATT

TTAAATTGGTATTGTGTAGATTGAGTTTGATTAGAAGTATTTAGAATAGAAAATGTGAAT

GTGAAATGAATTGGTTGAATTGATGATATTTGGGAACTATATGGTTTTAATTTGCAGGGG

GTTTTATGTAAAAATAAGCAGAAATGCTGCCGAAATTTTTATAAAAAAAAATGAAGTCAT

TTGGTAAACAAATTAATAAATTTTATGAATTATTTTAATATATTGGTTATTTATTTAAGA

ATTGTTGTAAATCGTTCGATACGTCCGGTAGTGCCTCGTAATTCTGTTCCGGCGACGGTT

CGGGGTTAAGGGGTGTTACATTTTATGGTATCAGAGCTATCAGGTTTAGCCGATTCTCGG

CCTAAATCGAGCTCGGAATTGAGTCTAGATGTACATGCCACTGTCGAGTTAAACTGAGTC

GGGATTTTTGGATGCTGACCTATTTGTTTGTTTTGTTTTATAGATTAAAGATGTCTGAAG

AAAGAATAAATGATACTGATGAAAGAATGTATAGTGAAGATAGAGAATTAGATGAAACAG

AATCTGTTGCACCGAGTGTGAATCCGTTAGGCAACCAACCTTCTAATGTAGAACGAGAAA

ATGTCAGAGATAGAGATGAATCCCAATTACTGAGAATTATAGCTGATGCATTACAAAGAG

TAGCAGGAACTACTCCTGTTACGACTTCAGTACCTACTGTTAGACGGGCTCCGATAAAGG

AACTGAGGAAATATGGTGCCACTGAATTTATGGGTCTAAAAGGAGTTGATCCATCCATAG

CTGAAAATTGGATGGAGTCGACTAAAAGAATTTTGCAGCAATTGGATTGTACCCCCCGAG

AGTGTTTAATCTGTGCCGTATCGTTATTACAAGAGGAGGCTTATCTATGGTGGGAATCAG

TGGTTCGACATTTACCAGAGAGTCAGATAACGTGGGATCTATTTCAGAAGGAGTTTCAAA

AGAAATATATCGGAGAGATGTATATTGAAGACAAGAAACAAGAGTTTTTGTTGCTACAAC

AGGGTGATATGTCAGTAATAGATTATGAGAGGGAATTCTCGAGACTCAGTAGATATGCCT

CCGAGTTTATTCCGACAGAAGCCGATAGTTGTAAAAGATTTTTACGGGGTTTACGAGACG

AGATCAAAGTGCAGCTAGTATCCCATCGGATCACTGAGTTAGTAGATTTGATTGAACGAG

CTAAAATGGTGGAACAAGTTCTGGGCCTCGACAAAAAGACTGAAGTTGTTAGACCAACCG

GGAAGCGTACAGGAACTACCAGTTCGAATCCTCAGCCGAAAAGACCAAAGGAATTCCAAA

GTGGTTGGAGATCCAGTTTCAGGTCAGACAGAGGTGGTAGAAATAGGGGAAAACAGACGA

TGACATCTACTGGCAGTGTGAAAGGTCCTTCCCGAGAAATAGATATTCCAGACTGCCAAC

ACTGCGGAAAGAAACACAGAGGGGAATGTTGGAAATTAACTAGAGGCTGTTTTCGATGTG

GTTCTACAGACCATTTCATCAGAGACTGTCCGAAAGTTGATAGTACTGTACCCGTGACAT

CACAGAGATCGGTATCTACAGCTAGAAGCAGAGGGTTAGGAAGAGGTGGTTCGGTTTCAA

GGGGAGGAAGTATTAGGAGAAGCAATGATATTGCTACTCAGCAGTCTGAGGCTAAAGTAC

CTGCCAGAGCTTATGTGGTCAGAACACAGGAAGAAGGTGACGCCCACGATGTAGTAACAG

GTATATTCTTACTATATTCTGAGCCTGTTTATGCTTTAATTGATCCCGGATCTTCACATT

CTTATATAAATTCAAAATTAGTTGAATTGGGAAAATTTAATTCTGAAATATCTAGAGTGA

CTGTAGAAGTGTCGAGTCCGTTGGGGCAAACAGTATTAGTGAATCAGATCTGTCCGAGAT

GCCCGTTAATTATACAAAATAAAACTTTTCCTATTGACCTGTTGATTATGCCATTTGGAG

ATTTTGATATAATACTGGGGATGGATTGGTTGGCTGAGCACGGAGTGGTATTGGATTGTT

ATAAAAAGAAGTTTAGTATTCAGACAGAAGACGGGGACAGAATTGAAGTAAATGGTATCC

GTACTAATGGGCCGACACGTATTATTTCGGCAATAAAGGCTAATAAATTGCTTCAGCGGG

GTTGTACAGCGTATTTAGCCTATGTTATTAATTCTGATTTGGTTGGTAGTCAGTGCAGTA

AGATTAGAACCGTATGTGAGTTTCCAGATGTATTTCCTGAAGAGCTACCGGGTTTACCAC

CTGACAGAGAGGTTGAATTTGCTATAGAAGTGTATCCGGGTACAGCACCAATCTCTATAC

CACCGTATCGAATGTCACCCACTGAGTTGAAAGAGTTGAAAGTGCAGTTACAGGACTTGT

CAGATCGTGGATTTATTAGACCGAGCATCTCACCTTGGGGAGCTCCAGTATTGTTTGTTA

AAAAGAAAGATGGATCGATGCGGCTTTGTATTGATTACCGGCAGTTAAACAAAGTGACGA

TCAAGAACCGGTATCCGTTACCCCGTATAGATGATTTATTTGATCAACTAAAAGGAGCTT

CAGTATTTTCAAAGATTGACTTAAGATCTGGGTATTATCAGCTGAAGGTAAAAGAAAGTG

ATGTTCCGAAGACTGCATTTCGTACTCGATATGGTCATTATGAATTTTTGGTGATGCCGT

TCGGGTTGACTAATGCTCCAGCTGCTTTTATGGATCTGATGAATCGTATTTTTCAGCCGT

ATTTAGATCAGTTTGTGGTGGTTTTTATTGATGACATCTTGGTTTATTCGAAGTCAGAGT

CAGAGCATGATCAGCATCTCAGAACCGTGCTACAAATTCTGCGAGAAAAACAGTTGTACG

GGAAACTAAGTAAATGTGAATTCTGGTTATCAGAGGTAGTATTCTTGGGACATGTTGTAT

CTGCGGATGGGATTAGAGTTGATCCGAAGAAGATCGAGGCAATTGTTCAATGGAAGGCAC

CAAAGAATGTATCAGAGGTACGCAGTTTTCTTGGTTTGGCTGGGTATTACAGAAGATTTG

TAAATGGGTTTTCGAAGATAGCTTTGCCGATGACCAAATTACTACAGAAGAATGTTCCAT

TTATCTGGGATGATCAGTGTCAGAGGAGCTTTGAAACATTGAAACAGATGTTGACAGAGG

CACCAGTTTTAACTTTACCAGAATCAGGGAAAGATTTCATAGTGTACAGTGATGCTTCTT

TGAATGGTTTGGGTTGTGTATTGATGCAAGAAGGAAAAGTAATAGCTTATGCATCTCGAC

AGTTGAAGTCACATGAACGCAACTACCCGACACACGATTTAGAGTTAGCTGCTGTAATCT

TTGCATTGAAGATTTGGATACATTACTTGTATGGTGAGAAATGTTATATTTACACTGATC

ATAAAAGTCTAAAATATCTTCTGTCACAAAAGGAGTTGAATCTGAGACAGAGACGGTGGA

TTGAACTTCTGAAAGATTATGATTGTGTTATAGATTATCATCCAGGGAAGGCAAATGTGG

TAGCAGATGCATTGAGTAGAAAAGCAGCGATTGAATTACGAGCAATGTTCGCTCGACTTA

GTATTAAGGATGATGGAAGTTTGTTAGCTGAGTTAAGAGTCAAGCCGGTGATGTTTGATC

AAATCAGAGCAGCACAGTTAAAAGATGAAAAGTTGATGAGGAAAAGAGAAATGGTACAGT

ATGGTGCGGTAGAAAATTTTAGTATTGACGAGCATGATTGTTTGAGATTTCGAAATCGAA

TTTGTGTTCCATCTACTTCTGAGATTAAAGAATTGATTCTCCGAGAAGCACATAATAGTA

TTTTTGCTTTGCACCCAGGAGGAACGAAGATGTATCGTGATCTACGAGAACTGTATTGGT

GGCCAGGAATGAAGAAAGATATAGTTGAATATGTCAGTAAATGCTTGACTTGTCAGCGGG

TAAAAGCAGAACATCAGGTACCAACAGGCCTGTTACAGCCTATTACTATTCCCGAGTGGA

AATGGGATCGCATTACCATGGATTTTGTTACGGGGTTGCCATTGTCAGTGAGTAAAAAGA

ATGCTATTTGGGTGATTGTTGATCGACTCACAAAATCAGCTCATTTTATAGCAGTTAGAA

CCGACTGGTCATTACAGAAGCTTGCCGAGGTTTATATTCGAGAAATTGTTAGATTACATG

GTATTCCGGTATCAATAATTTCAGACAGAGATCCTCGATTCACTTCGAGATTTTGGAAGC

AGCTGCATGAATCATTGGGTACTCGACTTAGTTTCAGTACAGCTTTTCATCCTCAAACTG

ATGGACAATCTGAACGAGTAATTCAGATATTAGAAGATATGCTTCGAGCTTGTGTCATTG

ATTTTGAATCAGGTTGGGAACGTTATTTACCATTGGCCGAGTTTGTTTATAATAATAGTT

TCCAATCTAGTATTCAAATGGCTCCATATGAAGCACTTTATGGTCGAAGGTGTCGATCAC

CAATATGTTGGACAAAATTAAGAGAAAGAAAAGTGATTGGGCCGGAATTGATTCAAGAGA

CAGAAGAAACAGTTAAAAAGATTAAAGATAGACTGAAAGCCGCTTTCGACAGACAGAAAT

CTTACGCAGACTTGAAACGACGAGACATTGAATATTCCGTTGGTGATAAGGTATTCCTCA

AAGTATCGCCGTGGAAGAAAATTTTGAGATTTGGTCGGAAGGGAAAATTAAGTCCGCGCT

TTATTGGGCCGTATGAGATAGTGGAAAGAATTGGGCCTGTTGCTTATCGATTATCCTTAC

CTCCAGAGTTACAGAAAATTCATGATGTTTTTCATGTTTCGATGCTTCGGAGATATAGAT

CGGATCCTTCTCATGTTATTCCCACTGAAGACATTGAACTTCGATCTGATTTAACTTATG

AAGAAGAACCAGTTCAAATATTAGCACGAGAAGTGAAAGAATTAAGAAATAAACGGGTTC

CTTTAGTACAAGTTTTATGGAGAAGCCATAGTGTGGAAGAAGCAACTTGGGAACCGGAAG

AGACAATGAGAGCACAATATCCTCATCTCTTCTCAGGTAAATTTCGAGGACGAAATTTAT

TAAGAGGGGGAGAAATGTAATGACCTAAAATTCATGGGCATCGGAAAAGTATAATATTGG

GCCTCCGTCCTAGTAAATTGAGTCCGAAAATAATTATTAGAAATATTTACGAGACTAGTA

GTGTGTTTAATTAGGTTTTAATTAAGTAAATTTAGCTTAATTTAGAGTAATTAGTAAAAA

GGATTAAATTGAATAAGAGTAAAAGTTTAATTATAGATTAAAGGAAAATAATAGGGACCA

AATGGGCAATTAAGCCACATTTGGAAGTTGAGGCGGCATAACATTGTAAAAATCTTAGAT

TTTTATATTATTATTTATATAAATATATAAATTAATTATAAAGTATATTATTAAATTAAT

TATATTATAAATATTATATTATTATATATAAAAGAAACAAAACAGAAAAGAAACAGAATA

GAAAGAACAAAGAAACAGAATAGAAGAGACGAAACAGGGGAGAAGCAGGGGAGAAAGAAG

AAAAAGAAGAAAAAAGGGGAAATAGGGTTTTTGAAGCTTGAAATTTAAATTGGTAAGTCA

AATTAGCCATTTTCTCTTAATTCTAATGTTTTAAAAGCTTTAAAACAAAGTTTTGATGGA

ATTAAGTTGATATTTTGTAAGTTCATAGGTTTTCAAGTATAGTTTATGTTGAACAAAAGA

GATGAATTAGGGATTAACTTGAAGGAATTTTAAGTTAGAATTGAAAAAGGGATTAAATTG

TAAAAGAAACTATAAGTTTTTTTTGTTTTAGGGACTAGATTGAGGAAAATTCGGAATTAA

GAAAATATGTTAAAAATTTAATAGTTAAATTTGAGTTTAAATGAAATTTGAATAGGAATA

AGGTGTGAATTGGTGTTATAAATTTGGTTATTAACATTTTTAATCAAAACAGTTTTGGGA

AGTAGCAATGGTCTGACTTTGAAAATTCACTAAAAATTTTATAAATTGAACTAGAGGATG

AACAAAATATGGAATTAAAGCTTATTGAGTCTAGTTTCTTATAGTAGAAACAATGTAAGC

AATTAATTGATGAATCAAGAGATATTTGAAATTTTGTAATACTGGTTCGGGGTGATTTCG

AGATGCCCTGTTTTAACTTTGGAAAATCATTAAAAATTGTACAAAAATTATTATGGAGTG

TAATTTATATATGTAAACTCCTTAATGAATCTAGTTTCAAAATAAATAAACAAGAACCTT

ATTCGAGTTCTGTACAATGAGATAATTTAGTTTTAGTGGAGAGAGGTCAGAACTGTCAAA

TGAAATAACAGGGGAGTATTTAACGAATAAACTGTATTAAATGGCTAGACCAAAAATTCT

GGAAATTTTATGATTAGAAGATATATGAGTCTAGTTTTAAGGAAAATTTACGGATATTAA

TTTGGAGTTTCGTAGCTCAAGATATAAATAATTTAGTAACAATGACCCAAGTAGACAGCT

TAATGGTGAAATTATATAAATACATTAAAAATGGTTAAATTTGCATGTTTAGGCTCATGA

ATTAAATTGAATCATGTTGTATTGATTATTATAAATTATTATTTTCGTAGCCAACAAAGA

ACCTAAAGCATCAGCATCGAAAGGAAAGGAGAAAGTCATCGAGGAGTAAACTCGAGAAAA

TTACGGTTTGTATTACTATAATTCAAGTTATTTATTATTAAATGTTAAATTTTAATTTAT

GTGTCTAGTAAATGAAATGTGAGGTAAGTATTATTATTATTATTATTATTATTATTATTA

TTATTATTATTATTATTATGAGTGGGAATTAAATTGAATAGTTGATATGAAATAATATTT

GAATTGTTTGTTGATTGAAAGCGGGAAATGAATTTAAATCGAATAGTGACCGATATTAAA

TTGAATGGAAATGTATTGAGTTGTGAAAATATGTTAATTGCGGATTAATTATTGATTGAA

AGGTGGAAAAATGATTGAATTGAAAGTGTGAGAAAGTGTGATTGAATTGGGATTATATGT

GATTTAAATACCCTATTAACTAGTCGGGCTGAGTCGGATATAGTTGGCATGCCATAGGAT

TGGAAGAGTTCAGGGATACTTCGACCTCGAGTCGATGAGACACTGGGTGTCACTATATTT

CTTCGGATAGATTCGATGAGGTACTGGGTACCAACTTTCTTCGGCTTTGCCGATGAGACA

CTGGGTGTCAACTATTGCTTCGAACTATCCGATGAGGCACTGGGTGCCATTCTGGTGTGT

TTGGTTGGATCCGTGTATTCGCCAAAGTCCGAGTTTTGTTAATAGGGTAAATGATGAAAT

GATAAACCGAACGAGTTGGTCAAACGAGCTATTGAAATGATATGAAAAAGTTGAATTGTG

AATTGAAATGTGAAATGAGATTGAGAAATGAACCTAAGGTTCGTGAATTATTCAAACTCA

AATTGTGGATATACGATATTGGTTGATGAATTGCTATTGTTGAAATATTTAATTTAAATT

GTATATACGATTTATGCTTTACATGTACATTATTGTTATAATTTGAATTATGGTAATACC

ACTGAGTATGAATTACTCAGCGTACGGTTGTTTCCGTGCGCAGGTCAATAGAAGTCAAAG

GTCTCGGTTCAGCATCCAGATTAATCCCGGCTTCGGCAAAACTTGGTGATGTATTTTTCC

TTTGGTAAAGGTGGCATGTACATAGATTGTGTATAAAGGTTATTATGTTTTATTATATAA

TGGTTAAAAATGTTAGTATTAAAAGTTTATGGATTTTAATGAAAGAAGTCTATCTATTTT

ATCTAATTAGTACATTGTTAAATTTTAAATTGGTATTAGATTGAGTTTGATTAGAAGTAT

TTAGAATAGAAAATGTGAATGTGAAATGAATTGGTTGAATTGATGATATTTGGGAACTAT

ATGGTTTTAATTTGC

>Deltapine491

AGGTAATGACCCAAAATTCATGGGCATCGGAAAAGTATAATATCGGGCCTCCGTCCTAGT

AAATTGAGTCCGAAAATAATTATTAGAAATATTTACGAGACTAGTAGTGTGTTTAATTAG

GTTTTAATTAAGTAAATTTAGCTTAATTTAGAGTAATTAGTAAAAAGGATTAAATTGAAT

AAGAGTAAAAGTTTAATTATAGATTAAAGGAAAATAATAGGGACCAAATGGGCAATTAAG

CCACATTTGGAAGTTGAGGCGGCATAACATTGTAAAAATCTTAGATTTTTATATTATTAT

TTATATAAATATATAAATTAATTATAAAGTATATTATTAAATTAATTATATTATAAATAT

TATATTATTATATATAAAAGAAACAAAACAGAAAAGAAACAGAATAGAAAGAACAAAGAA

ACAGAATAGAAGAGACGAAACAGGGGAGAAGCAGGGGAGAAAGAAGAAAAAGAAGAAAAA

AGGGGAAATAGGGTTTTTGAAGCTTGAAATTTAAATTGGTAAGTCAAATTAGCCATTTTC

TCTTAATTCTAATGTTTTAAAAGCTTTAAAACAAAGTTTTGATGGAATTAAGTTGATATT

TTGTAAGTTCATAGGTTTTCAAGTATAGTTTATGTTGAACAAAAGAGATGAATTAGGGAT

TAACTTGAAGGAATTTTAAGTTAGAATTGAAAAAGGGATTAAATTGTAAAAGAAACTATA

AGTTTTTTTTGTTTTAGGGACTAGATTGAGGAAAATTCGGAATTAAGAAAATATGTTAAA

AATTTAATAGTTAAATTTGAGTTTAAATGAAATTTGAATAGGAATAAGGTGTGAATTGGT

GTTATAAATTTGGTTATTAACATTTTTAATCAAAACAGTTTTGGGAAGTAGCAATGGTCT

GACTTTGAAAATTCACTAAAAATTTTATAAATTGAACTAGAGGATGAACAAAATATGGAA

TTAAAGCTTATTGAGTCTAGTTTCTTATAGTAGAAACAATGTAAGCAATTAATTGATGAA

TCAAGAGATATTTGAAATTTTGTAATACTGGTTCGGGGTGATTTCGAGATGCCCTGTTTT

AACTTTGGAAAATCATTAAAAATTGTACAAAAATTATTATGGAGTGTAATTTATATATGT

GAACTCCTTAATGAATCTAGTTTCAAAATAAATAAACAAGAACCTTATTCGAGTTCTGTA

CAATGAGATAATTTAGTTTTAGTGGAGAGAGGTCAGAACTGTCAAATGAAATAACAGGGG

AGTATTTAACGAATAAACTGTATTAAATGGCTAGACCAAAAATTCTGGAAATTTTATGAT

TAGAAGATATATGAGTCTAGTTTTAAGGAAAATTTACGGATATTAATTTGGAGTTTCGTA

GCTCAAGATATAAATAATTTAGTAACAATGACCCAAGTAGACAGCTTAATGGTGAAATTA

TATAAATACATTAAAAATGGTTAAATTTGCATGTTTAGGCTCATGAATTAAATTGAATCA

TGTTGTATTGATTATTATAAATTATTATTTTCGTAGCCAACAAAGAACCTAAAGCATCAG

CATCGAAAGGAAAGGAGAAAGTCATCGAGGAGTAAACTCGAGAAAATTACGGTTTGTATT

ACTATAATTCAAGTTATTTATTATTAAATGTTAAATTTTAATTTATGTGTCTAGTAAATG

AAATGTGAGGTAAGTATTATTATTATTATTATTATTATTATTATTATTATTATTATTATT

ATGAGTGGGAATTAAATTGAATAGTTGATATGAAATAATATTTGAATTGTTTGTTGATTG

AAAGCGGGAAATGAATTTAAATCGAATAGTGACCGATATTAAATTGAATGGAAATGTATT

GAGTTGTGAAAGTATGTTAATTGCGGATTAATTATTGATTGAAAGGTGGAAAAATGATTG

AATTGAAAGTGTGAGAAAGTGTGATTGAATTGGGATTATATGTGATTTAAATACCCTATT

AACTAGTCGGGCTGAGTCGGATATAGTTGGCATGCCATAGGATTGGAAGAGTTCAGGGAT

ACTTCGACCTCGAGTCGATGAGACACTGGGTGATTTCTTCGGATAGATTGGATGAGGTAC

TGGGTACCAACTTTCTTCGGCTTTGCCGATGAGACACTGGGTGTCAACTATTGCTTCGAA

CTATCCGATGAGGCACTGGGTGCCATTCTGGTGTGTTTGGTTGGATCCGTGTATCCGCCA

AAGTCCGAGTTTTGTTAATAGGGTAAATGATGAAATGATAAACCGAACGAGTTGGTCAAA

CGAGCTATTGAAATGATATGAAAAAGTTGAATTGTGAATTGAAATGTGAAATGAGATTGA

GAAATGAACCTAAGGTTCGTGAATTATTCAAACTCAAATTGTGGATATACGATATTGGTT

GATGAATTGCTATTGTTGAAATATTTAATTTAAATTGTATATACGATTTATGCTTTACAT

GTACATTATTGTTATAATTTGAATTATGGTAATACCACTGAGTATGAATTACTCAGCGTA

CGGTTGTTTCCGTGCGCAGGTCAATAGAAGTCAAAGGTCTCGGTTCAGCATCCAGATTAA

TCCCGGCTTCGGCAAAACTTGGTGATGTATTTTTCCTTTGGTAAAGGTGGCATGTACATA

GATTGTGTATAAAGGTTATTATGTTTTATTATATAATGGTTAAAAATGTTAGTATTAAAA

GTTTATGGATTTTAATGAAAGAAGTCTATCTATTTTATCTAATTAGTACATTGTTAAATT

TTAAATTGGTATTGTGTAGATTGAGTTTGATTAGAAGTATTTAGAATAGAAAATGTGAAT

GTGAAATGAATTGGTTGAATTGATGATATTTGGGAACTATATGGTTTTAATTTGCAGGGG

GTTTTATGTAAAAATAAGCAGAAATGCTGCCGAAATTTTTATAAAAAAAAATGAAGTCAT

TTGGTAAACAAATTAATAAATTTTATGAATTATTTTAATATATTGGTTATTTATTTAAGA

ATTGTTGTAAATCGTTCGATACGTCCGGTAGTGCCTCGTAATTCTGTTCCGGCGACGGTT

CGGGGTTAAGGGGTGTTACATTTTATGGTATCAGAGCTATCAGGTTTAGCCGATTCTCGG

CCTAAATCGAGCTCGGAATTGAGTCTAGATGTACATGCCACTGTCGAGTTAAACTGAGTC

GGGATTTTTGGATGCTGACCTATTTGTTTGTTTTGTTTTATAGATTAAAGATGTCTGAAG

AAAGAATAAATGATACTGATGAAAGAATGTATAGTGAAGATAGAGAATTAGATGAAACAG

AATCTGTTGCACCGAGTGTGAATCCGTTAGGCAACCAACCTTCTAATGTAGAACGAGAAA

ATGTCAGAGATAGAGATGAATCCCAATTACTGAGAATTATAGCTGATGCATTACAAAGAG

TAGCAGGAACTACTCCTGTTACGACTTCAGTACCTACTGTTAGACGGGCTCCGATAAAGG

AACTGAGGAAATATGGTGCCACTGAATTTATGGGTCTAAAAGGAGTTGATCCATCCATAG

CTGAAAATTGGATGGAGTCGACTAAAAGAATTTTGCAGCAATTGGATTGTACCCCCCGAG

AGTGTTTAATCTGTGCCGTATCGTTATTACAAGGGGAGGCTTATCTATGGTGGGAATCAG

TGGTTCGACATTTACCAGAGAGTCAGATAACGTGGGATCTATTTCAGAAGGAGTTTCAAA

AGAAATATATCGGAGAGATGTATATTGAAGACAAGAAACAAGAGTTTTTGTTGCTACAAC

AGGGTGATATGTCAGTAATAGATTATGAGAGGGAATTCTCGAGACTCAGTAGATATGCCT

CCGAGTTTATTCCGACAGAAGCCGATAGTTGTAAAAGATTTTTACGGGGTTTACGAGACG

AGATCAAAGTGCAGCTAGTATCCCATCGGATCACTGAGTTAGTAGATTTGATTGAACGAG

CTAAAATGGTGGAACAAGTTCTGGGCCTCGACAAAAAGACTGAAGTTGTTAGACCAACCG

GGAAGCGTACAGGAACTACCAGTTCGAATCCTCAGCCGAAAAGACCAAAGGAATTCCAAA

GTGGTTGGAGATCCAGTTTCAGGTCAGACAGAGGTGGTAGAAATAGGGGAAAACAGACGA

TGACATCTACTGGCAGTGTGAAAGGTCCTTCCCGAGAAATAGATATTCCAGACTGCCAAC

ACTGCGGAAAGAAACACAGAGGGGAATGTTGGAAATTAACTAGAGGCTGTTTTCGATGTG

GTTCTACAGACCATTTCATCAGAGACTGTCCGAAAGTTGATAGTACTGTACCCGTGACAT

CACAGAGATCGGTATCTACAGCTAGAAGCAGAGGGTTAGGAAGAGGTGGTTCAGTTTCAA

GGGGAGGAAGTATTAGGAGAAGCAATGATATTGCTACTCAGCAGTCTGAGGCTAAAGTAC

CTGCCAGAGCTTATGTGGTCAGAACACAGGAAGAAGGTGACGCCCACGATGTAGTAACAG

GTATATTCTTACTATATTCTGAGCCTGTTTATGCTTTAATTGATCCCGGATCTTCACATT

CTTATATAAATTCAAAATTAGTTGAATTGGGAAAATTTAATTCTGAAATATCTAGAGTGA

CTGTAGAAGTGTCGAGTCCGTTGGGGCAAACAGTATTAGTGAATCAGATCTGTCCGAGAT

GCCCGTTAATTATACAAAATAAAACTTTTCCTATTGACCTGTTGATTATGCCATTTGGAG

ATTTTGATATAATACTGGGGATGGATTGGTTGGCTGAGCACGGAGTGGTATTGGATTGTT

ATAAAAAGAAGTTTAGTATTCAGACAGAAGACGGGGACAGAATTGAAGTAAATGGTATCC

GTACTAATGGGCCGACACGTATTATTTCGGCAATAAAGGCTAATAAATTGCTTCAGCGGG

GTTGTACAGCGTATTTAGCCTATGTTATTAATTCTGATTTGGTTGGTAGTCAGTGCAGTA

AGATTAGAACCGTATGTGAGTTTCCAGATGTATTTCCTGAAGAGCTACCGGGTTTACCAC

CTGACAGAGAGGTTGAATTTGCTATAGAAGTGTATCCGGGTACAGCACCAATCTCTATAC

CACCGTATCGAATGTCACCCACTGAGTTGAAAGAGTTGAAAGTGCAGTTACAGGACTTGT

CAGATCGTGGATTTATTAGACCGAGCATCTCACCTTGGGGAGCTCCAGTATTGTTTGTTA

AAAAGAAAGATGGATCGATGCGGCTTTGTATTGATTACCGGCAGTTAAACAAAGTGACGA

TCAAGAACCGGTATCCGTTACCCCGTATAGATGATTTATTTGATCAACTAAAAGGAGCTT

CAGTATTTTCAAAGATTGACTTAAGATCTGGGTATTATCAGCTGAAGGTAAAAGAAAGTG

ATGTTCCGAAGACTGCATTTCGTACTCGATATGGTCATTATGAATTTTTGGTGATGCCGT

TCGGGTTGACTAATGCTCCAGCTGCTTTTATGGATCTGATGAATCGTATTTTTCAGCCGT

ATTTAGATCAGTTTGTGGTGGTTTTTATTGATGACATCTTGGTTTATTCGAAGTCAGAGT

CAGAGCATGATCAGCATCTCAGAACCGTGCTACAAATTCTGCGAGAAAAACAGTTGTACG

GGAAACTAAGTAAATGTGAATTCTGGTTATCAGAGGTAGTATTCTTGGGACATGTTGTAT

CTGCGGATGGGATTAGAGTTGATCCGAAGAAGATCGAGGCAATTGTTCAATGGAAGGCAC

CAAAGAATGTATCAGAGGTACGCAGTTTTCTTGGTTTGGCTGGGTATTACAGAAGATTTG

TAAATGGGTTTTCGAAGATAGCTTTGCCGATGACCAAATTACTACAGAAGAATGTTCCAT

TTATCTGGGATGATCAGTGTCAGAGGAGCTTTGAAACATTGAAACAGATGTTGACAGAGG

CACCAGTTTTAACTTTACCAGAATCAGGGAAAGATTTCATAGTGTACAGTGATGCTTCTT

TGAATGGTTTGGGTTGTGTATTGATGCAAGAAGGAAAAGTAATAGCTTATGCATCTCGAC

AGTTGAAGTCACATGAACGCAACTACCCGACACACGATTTAGAGTTAGCTGCTGTAATCT

TTGCATTGAAGATTTGGATACATTACTTGTATGGTGAGAAATGTTATATTTACACTGATC

ATAAAAGTCTAAAATATCTTCTGTCACAAAAGGAGTTGAATCTGAGACAGAGACGGTGGA

TTGAACTTCTGAAAGATTATGATTGTGTTATAGATTATCATCCAGGGAAGGCAAATGTGG

TAGCAGATGCATTGAGTAGAAAAGCAGCGATTGAATTACGAGCAATGTTCGCTCGACTTA

GTATTAAGGATGATGGAAGTTTGTTAGCTGAGTTAAGAGTCAAGCCGGTGATGTTTGATC

AAATCAGAGCAGCACAGTTAAAAGATGAAAAGTTGATGAGGAAAAGAGAAATGGTACAGT

ATGGTGCGGTAGAAAATTTTAGTATTGACGAGCATGATTGTTTGAGATTTCGAAATCGAA

TTTGTGTTCCATCTACTTCTGAGATTAAAGAATTGATTCTCCGAGAAGCACATAATAGTA

TTTTTGCTTTGCACCCAGGAGGAACGAAGATGTATCGTGATCTACGAGAACTGTATTGGT

GGCCAGGAATGAAGAAAGATATAGTTGAATATGTCAGTAAATGCTTGACTTGTCAGCGGG

TAAAAGCAGAACATCAGGTACCAACAGGCCTGTTACAGCCTATTACTATTCCCGAGTGGA

AATGGGATCGCATTACCATGGATTTTGTTACGGGGTTGCCATTGTCAGTGAGTAAAAAGA

ATGCTATTTGGGTGATTGTTGATCGACTCACAAAATCAGCTCATTTTATAGCAGTTAGAA

CCGACTGGTCATTACAGAAGCTTGCCGAGGTTTATATTCGAGAAATTGTTAGATTACATG

GTATTCCGGTATCAATAATTTCAGACAGAGATCCTCGATTCACTTCGAGATTTTGGAAGC

AGCTGCATGAATCATTGGGTACTCGACTTAGTTTCAGTACAGCTTTTCATCCTCAAACTG

ATGGACAATCTGAACGAGTAATTCAGATATTAGAAGATATGCTTCGAGCTTGTGTCATTG

ATTTTGAATCAGGTTGGGAACGTTATTTACCATTGGCCGAGTTTGTTTATAATAATAGTT

TCCAATCTAGTATTCAAATGGCTCCATATGAAGCACTTTATGGTCGAAGGTGTCGATCAC

CAATATGTTGGACAAAATTAAGAGAAAGAAAAGTGATTGGGCCGGAATTGATTCAAGAGA

CAGAAGAAACAGTTAAAAAGATTAAAGATAGACTGAAAGCCGCTTTCGACAGACAGAAAT

CTTACGCAGACTTGAAACGACGAGACATTGAATATTCCGTTGGTGATAAGGTATTCCTCA

AAGTATCGCCGTGGAAGAAAATTTTGAGATTTGGTCGGAAGGGAAAATTAAGTCCGCGCT

TTATTGGGCCGTATGAGATAGTGGAAAGAATTGGGCCTGTTGCTTATCGATTATCCTTAC

CTCCAGAGTTACAGAAAATTCATGATGTTTTTCATGTTTCGATGCTTCGGAGATATAGAT

CGGATCCTTCTCATGTTATTCCCACTGAAGACATTGAACTTCGATCTGATTTAACTTATG

AAGAAGAACCAGTTCAAATATTAGCACGAGAAGTGAAAGAATTAAGAAATAAACGGGTTC

CTTTAGTACAAGTTTTATGGAGAAGCCATAGTGTGGAAGAAGCAACTTGGGAACCGGAAG

AGACAATGAGAGCACAATATCCTCATCTCTTCTCAGGTAAATTTCGAGGACGAAATTTAT

TAAGAGGGGGAGAAATGTAATGACCTAAAATTCATGGGCATCGGAAAAGTATAATATTGG

GCCTCCGTCCTAGTAAATTGAGTCCGAAAATAATTATTAGAAATATTTACGAGACTAGTA

GTGTGTTTAATTAGGTTTTAATTAAGTAAATTTAGCTTAATTTAGAGTAATTAGTAAAAA

GGATTAAATTGAATAAGAGTAAAAGTTTAATTATAGATTAAAGGAAAATAATAGGGACCA

AATGGGCAATTAAGCCACATTTGGAAGTTGAGGCGGCATAACATTGTAAAAATCTTAGAT

TTTTATATTATTATTTATATAAATATATAAATTAATTATAAAGTATATTATTAAATTAAT

TATATTATAAATATTATATTATTATATATAAAAGAAACAAAACAGAAAAGAAACAGAATA

GAAAGAACAAAGAAACAGAATAGAAGAGACGAAACAGGGGAGAAGCAGGGGAGAAAGAAG

AAAAAGAAGAAAAAAGGGGAAATAGGGTTTTTGAAGCTTGAAATTTAAATTGGTAAGTCA

AATTAGCCATTTTCTCTTAATTCTAATGTTTTAAAAGCTTTAAAACAAAGTTTTGATGGA

ATTAAGTTGATATTTTGTAAGTTCATAGGTTTTCAAGTATAGTTTATGTTGAACAAAAGA

GATGAATTAGGGATTAACTTGAAGGAATTTTAAGTTAGAATTGAAAAAGGGATTAAATTG

TAAAAGAAACTATAAGTTTTTTTTGTTTTAGGGACTAGATTGAGGAAAATTCGGAATTAA

GAAAATATGTTAAAAATTTAATAGTTAAATTTGAGTTTAAATGAAATTTGAATAGGAATA

AGGTGTGAATTGGTGTTATAAATTTGGTTATTAACATTTTTAATCAAAACAGTTTTGGGA

AGTAGCAATGGTCTGACTTTGAAAATTCACTAAAAATTTTATAAATTGAACTAGAGGATG

AACAAAATATGGAATTAAAGCTTATTGAGTCTAGTTTCTTATAGTAGAAACAATGTAAGC

AATTAATTGATGAATCAAGAGATATTTGAAATTTTGTAATACTGGTTCGGGGTGATTTCG

AGATGCCCTGTTTTAACTTTGGAAAATCATTAAAAATTGTACAAAAATTATTATGGAGTG

TAATTTATATATGTAAACTCCTTAATGAATCTAGTTTCAAAATAAATAAACAAGAACCTT

ATTCGAGTTCTGTACAATGAGATAATTTAGTTTTAGTGGAGAGAGGTCAGAACTGTCAAA

TGAAATAACAGGGGAGTATTTAACGAATAAACTGTATTAAATGGCTAGACCAAAAATTCT

GGAAATTTTATGATTAGAAGATATATGAGTCTAGTTTTAAGGAAAATTTACGGATATTAA

TTTGGAGTTTCGTAGCTCAAGATATAAATAATTTAGTAACAATGACCCAAGTAGACAGCT

TAATGGTGAAATTATATAAATACATTAAAAATGGTTAAATTTGCATGTTTAGGCTCATGA

ATTAAATTGAATCATGTTGTATTGATTATTATAAATTATTATTTTCGTAGCCAACAAAGA

ACCTAAAGCATCAGCATCGAAAGGAAAGGAGAAAGTCATCGAGGAGTAAACTCGAGAAAA

TTACGGTTTGTATTACTATAATTCAAGTTATTTATTATTAAATGTTAAATTTTAATTTAT

GTGTCTAGTAAATGAAATGTGAGGTAAGTATTATTATTATTATTATTATTATTATTATTA

TTATTATTATTATTATTATGAGTGGGAATTAAATTGAATAGTTGATATGAAATAATATTT

GAATTGTTTGTTGATTGAAAGCGGGAAATGAATTTAAATCGAATAGTGACCGATATTAAA

TTGAATGGAAATGTATTGAGTTGTGAAAATATGTTAATTGCGGATTAATTATTGATTGAA

AGGTGGAAAAATGATTGAATTGAAAGTGTGAGAAAGTGTGATTGAATTGGGATTATATGT

GATTTAAATACCCTATTAACTAGTCGGGCTGAGTCGGATATAGTTGGCATGCCATAGGAT

TGGAAGAGTTCAGGGATACTTCGACCTCGAGTCGATGAGACACTGGGTGTCACTATATTT

CTTCGGATAGATTCGATGAGGTACTGGGTACCAACTTTCTTCGGCTTTGCCGATGAGACA

CTGGGTGTCAACTATTGCTTCGAACTATCCGATGAGGCACTGGGTGCCATTCTGGTGTGT

TTGGTTGGATCCGTGTATTCGCCAAAGTCCGAGTTTTGTTAATAGGGTAAATGATGAAAT

GATAAACCGAACGAGTTGGTCAAACGAGCTATTGAAATGATATGAAAAAGTTGAATTGTG

AATTGAAATGTGAAATGAGATTGAGAAATGAACCTAAGGTTCGTGAATTATTCAAACTCA

AATTGTGGATATACGATATTGGTTGATGAATTGCTATTGTTGAAATATTTAATTTAAATT

GTATATACGATTTATGCTTTACATGTACATTATTGTTATAATTTGAATTATGGTAATACC

ACTGAGTATGAATTACTCAGCGTACGGTTGTTTCCGTGCGCAGGTCAATAGAAGTCAAAG

GTCTCGGTTCAGCATCCAGATTAATCCCGGCTTCGGCAAAACTTGGTGATGTATTTTTCC

TTTGGTAAAGGTGGCATGTACATAGATTGTGTATAAAGGTTATTATGTTTTATTATATAA

TGGTTAAAAATGTTAGTATTAAAAGTTTATGGATTTTAATGAAAGAAGTCTATCTATTTT

ATCTAATTAGTACATTGTTAAATTTTAAATTGGTATTAGATTGAGTTTGATTAGAAGTAT

TTAGAATAGAAAATGTGAATGTGAAATGAATTGGTTGAATTGATGATATTTGGGAACTAT

ATGGTTTTAATTTGC

>Deltapine5409

ATGTAATGACCCAAAATTCATGGGCATCGGAAAAGTATAATATCGGGCCTCCGTCCTAGT

AAATTGAGTCCGAAAATAATTATTAGAAATATTTACGAGACTAGTAGTGTGTTTAATTAG

GTTTTAATTAAGTAAATTTAGCTTAATTTAGAGTAATTAGTAAAAAGGATTAAATTGAAT

AAGAGTAAAAGTTTAATTATAGATTAAAGGAAAATAATAGGGACCAAATGGGCAATTAAG

CCACATTTGGAAGTTGAGGCGGCATAACATTGTAAAAATCTTAGATTTTTATATTATTAT

TTATATAAATATATAAATTAATTATAAAGTATATTATTAAATTAATTATATTATAAATAT

TATATTATTATATATAAAAGAAACAAAACAGAAAAGAAACAGAATAGAAAGAACAAAGAA

ACAGAATAGAAGAGACGAAACAGGGGAGAAGCAGGGGAGAAAGAAGAAAAAGAAGAAAAA

AGGGGAAATAGGGTTTTTGAAGCTTGAAATTTAAATTGGTAAGTCAAATTAGCCATTTTC

TCTTAATTCTAATGTTTTAAAAGCTTTAAAACAAAGTTTTGATGGAATTAAGTTGATATT

TTGTAAGTTCATAGGTTTTCAAGTATAGTTTATGTTGAACAAAAGAGATGAATTAGGGAT

TAACTTGAAGGAATTTTAAGTTAGAATTGAAAAAGGGATTAAATTGTAAAAGAAACTATA

AGTTTTTTTTGTTTTAGGGACTAGATTGAGGAAAATTCGGAATTAAGAAAATATGTTAAA

AATTTAATAGTTAAATTTGAGTTTAAATGAAATTTGAATAGGAATAAGGTGTGAATTGGT

GTTATAAATTTGGTTATTAACATTTTTAATCAAAACAGTTTTGGGAAGTAGCAATGGTCT

GACTTTGAAAATTCACTAAAAATTTTATAAATTGAACTAGAGGATGAACAAAATATGGAA

TTAAAGCTTATTGAGTCTAGTTTCTTATAGTAGAAACAATGTAAGCAATTAATTGATGAA

TCAAGAGATATTTGAAATTTTGTAATACTGGTTCGGGGTGATTTCGAGATGCCCTGTTTT

AACTTTGGAAAATCATTAAAAATTGTACAAAAATTATTATGGAGTGTAATTTATATATGT

GAACTCCTTAATGAATCTAGTTTCAAAATAAATAAACAAGAACCTTATTCGAGTTCTGTA

CAATGAGATAATTTAGTTTTAGTGGAGAGAGGTCAGAACTGTCAAATGAAATAACAGGGG

AGTATTTAACGAATAAACTGTATTAAATGGCTAGACCAAAAATTCTGGAAATTTTATGAT

TAGAAGATATATGAGTCTAGTTTTAAGGAAAATTTACGGATATTAATTTGGAGTTTCGTA

GCTCAAGATATAAATAATTTAGTAACAATGACCCAAGTAGACAGCTTAATGGTGAAATTA

TATAAATACATTAAAAATGGTTAAATTTGCATGTTTAGGCTCATGAATTAAATTGAATCA

TGTTGTATTGATTATTATAAATTATTATTTTCGTAGCCAACAAAGAACCTAAAGCATCAG

CATCGAAAGGAAAGGAGAAAGTCATCGAGGAGTAAACTCGAGAAAATTACGGTTTGTATT

ACTATAATTCAAGTTATTTATTATTAAATGTTAAATTTTAATTTATGTGTCTAGTAAATG

AAATGTGAGGTAAGTATTATTATTATTATTATTATTATTATTATTATTATTATTATTATT

ATGAGTGGGAATTAAATTGAATAGTTGATATGAAATAATATTTGAATTGTTTGTTGATTG

AAAGCGGGAAATGAATTTAAATCGAATAGTGACCGATATTAAATTGAATGGAAATGTATT

GAGTTGTGAAAATATGTTAATTGCGGATTAATTATTGATTGAAAGGTGGAAAAATGATTG

AATTGAAAGTGTGAGAAAGTGTGATTGAATTGGGATTATATGTGATTTAAATACCCTATT

AACTAGTCGGGCTGAGTCGGATATAGTTGGCATGCCATAGGATTGGAAGAGTTCAGGGAT

ACTTCGACCTCGAGTCGATGAGACACTGGGTGATTTCTTCGGATAGATTGGATGAGGTAC

TGGGTACCAACTTTCTTCGGCTTTGCCGATGAGACACTGGGTGTCAACTATTGCTTCGAA

CTATCCGATGAGGCACTGGGTGCCATTCTGGTGTGTTTGGTTGGATCCGTGTATCCGCCA

AAGTCCGAGTTTTGTTAATAGGGTAAATGATGAAATGATAAACCGAACGAGTTGGTCAAA

CGAGCTATTGAAATGATATGAAAAAGTTGAATTGTGAATTGAAATGTGAAATGAGATTGA

GAAATGAACCTAAGGTTCGTGAATTATTCAAACTCAAATTGTGGATATACGATATTGGTT

GATGAATTGCTATTGTTGAAATATTTAATTTAAATTGTATATACGATTTATGCTTTACAT

GTACATTATTGTTATAATTTGAATTATGGTAATACCACTGAGTATGAATTACTCAGCGTA

CGGTTGTTTCCGTGCGCAGGTCAATAGAAGTCAAAGGTCTCGGTTCAGCATCCAGATTAA

TCCCGGCTTCGGCAAAACTTGGTGATGTATTTTTCCTTTGGTAAAGGTGGCATGTACATA

GATTGTGTATAAAGGTTATTATGTTTTATTATATAATGGTTAAAAATGTTAGTATTAAAA

GTTTATGGATTTTAATGAAAGAAGTCTATCTATTTTATCTAATTAGTACATTGTTAAATT

TTAAATTGGTATTGTGTAGATTGAGTTTGATTAGAAGTATTTAGAATAGAAAATGTGAAT

GTGAAATGAATTGGTTGAATTGATGATATTTGGGAACTATATGGTTTTAATTTGCAGGGG

GTTTTATGTAAAAATAAGCAGAAATGCTGCCGAAATTTTTATAAAAAAAAATGAAGTCAT

TTGGTAAACAAATTAATAAATTTTATGAATTATTTTAATATATTGGTTATTTATTTAAGA

ATTGTTGTAAATCGTTCGATACGTCCGGTAGTGCCTCGTAATTCTGTTCCGGCGACGGTT

CGGGGTTAAGGGGTGTTACATTTTATGGTATCAGAGCTATCAGGTTTAGCCGATTCTCGG

CCTAAATCGAGCTCGGAATTGAGTCTAGATGTACATGCCACTGTCGAGTTAAACTGAGTC

GGGATTTTTGGATGCTGACCTATTTGTTTGTTTTGTTTTATAGATTAAAGATGTCTGAAG

AAAGAATAAATGATACTGATGAAAGAATGTATAGTGAAGATAGAGAATTAGATGAAACAG

AATCTGTTGCACCGAGTGTGAATCCGTTAGGCAACCAACCTTCTAATGTAGAACGAGAAA

ATGTCAGAGATAGAGATGAATCCCAATTACTGAGAATTATAGCTGATGCATTACAAAGAG

TAGCAGGAACTACTCCTGTTACGACTTCAGTACCTACTGTTAGACGGGCTCCGATAAAGG

AACTGAGGAAATATGGTGCCACTGAATTTATGGGTCTAAAAGGAGTTGATCCATCCATAG

CTGAAAATTGGATGGAGTCGACTAAAAGAATTTTGCAGCAATTGGATTGTACCCCCCGAG

AGTGTTTAATCTGTGCCGTATCGTTATTACAAGGGGAGGCTTATCTATGGTGGGAATCAG

TGGTTCGACATTTACCAGAGAGTCAGATAACGTGGGATCTATTTCAGAAGGAGTTTCAAA

AGAAATATATCGGAGAGATGTATATTGAAGACAAGAAACAAGAGTTTTTGTTGCTACAAC

AGGGTGATATGTCAGTAATAGATTATGAGAGGGAATTCTCGAGACTCAGTAGATATGCCT

CCGAGTTTATTCCGACAGAAGCCGATAGTTGTAAAAGATTTTTACGGGGTTTACGAGACG

AGATCAAAGTGCAGCTAGTATCCCATCGGATCACTGAGTTAGTAGATTTGATTGAACGAG

CTAAAATGGTGGAACAAGTTCTGGGCCTCGACAAAAAGACTGAAGTTGTTAGACCAACCG

GGAAGCGTACAGGAACTACCAGTTCGAATCCTCAGCCGAAAAGACCAAAGGAATTCCAAA

GTGGTTGGAGATCCAGTTTCAGGTCAGACAGAGGTGGTAGAAATAGGGGAAAACAGACGA

TGACATCTACTGGCAGTGTGAAAGGTCCTTCCCGAGAAATAGATATTCCAGACTGCCAAC

ACTGCGGAAAGAAACACAGAGGGGAATGTTGGAAATTAACTAGAGGCTGTTTTCGATGTG

GTTCTACAGACCATTTCATCAGAGACTGTCCGAAAGTTGATAGTACTGTACCCGTGACAT

CACAGAGATCGGTATCTACAGCTAGAGGCAGAGGGTTAGGAAGAGGTGGTTCGGTTTCAA

GGGGAGGAAGTATTAGGAGAAGCAATGATATTGCTACTCAGCAGTCTGAGGCTAAAGTAC

CTGCCAGAGCTTATGTGGTCAGAACACAGGAAGAAGGTGACGCCCACGATGTAGTAACAG

GTATATTCTTACTATATTCTGAGCCTGTTTATGCTTTAATTGATCCCGGATCTTCACATT

CTTATATAAATTCAAAATTAGTTGAATTGGGAAAATTTAATTCTGAAATATCTAGAGTGA

CTGTAGAAGTGTCGAGTCCGTTGGGGCAAACAGTATTAGTGAATCAGATCTGTCCGAGAT

GCCCGTTAATTATACAAAATAAAACTTTTCCTATTGACCTGTTGATTATGCCATTTGGAG

ATTTTGATATAATACTGGGGATGGATTGGTTGGCTGAGCACGGAGTGGTATTGGATTGTT

ATAAAAAGAAGTTTAGTATTCAGACAGAAGACGGGGACAGAATTGAAGTAAATGGTATCC

GTACTAATGGGCCGACACGTATTATTTCGGCAATAAAGGCTAATAAATTGCTTCAGCGGG

GTTGTACAGCGTATTTAGCCTATGTTATTAATTCTGATTTGGTTGGTAGTCAGTGCAGTA

AGATTAGAACCGTATGTGAGTTTCCAGATGTATTTCCTGAAGAGCTACCGGGTTTACCAC

CTGACAGAGAGGTTGAATTTGCTATAGAAGTGTATCCGGGTACAGCACCAATCTCTATAC

CACCGTATCGAATGTCACCCACTGAGTTGAAAGAGTTGAAAGTGCAGTTACAGGACTTGT

CAGATCGTGGATTTATTAGACCGAGCATCTCACCTTGGGGAGCTCCAGTATTGTTTGTTA

AAAAGAAAGATGGATCGATGCGGCTTTGTATTGATTACCGGCAGTTAAACAAAGTGACGA

TCAAGAACCGGTATCCGTTACCCCGTATAGATGATTTATTTGATCAACTAAAAGGAGCTT

CAGTATTTTCAAAGATTGACTTAAGATCTGGGTATTATCAGCTGAAGGTAAAAGAAAGTG

ATGTTCCGAAGACTGCATTTCGTACTCGATATGGTCATTATGAATTTTTGGTGATGCCGT

TCGGGTTGACTAATGCTCCAGCTGCTTTTATGGATCTGATGAATCGTATTTTTCAGCCGT

ATTTAGATCAGTTTGTGGTGGTTTTTATTGATGACATCTTGGTTTATTCGAAGTCAGAGT

CAGAGCATGATCAGCATCTCAGAACCGTGCTACAAATTCTGCGAGAAAAACAGTTGTACG

GGAAACTAAGTAAATGTGAATTCTGGTTATCAGAGGTAGTATTCTTGGGACATGTTGTAT

CTGCGGATGGGATTAGAGTTGATCCGAAGAAGATCGAGGCAATTGTTCAATGGAAGGCAC

CAAAGAATGTATCAGAGGTACGCAGTTTTCTTGGTTTGGCTGGGTATTACAGAAGATTTG

TAAATGGGTTTTCGAAGATAGCTTTGCCGATGACCAAATTACTACAGAAGAATGTTCCAT

TTATCTGGGATGATCAGTGTCAGAGGAGCTTTGAAACATTGAAACAGATGTTGACAGAGG

CACCAGTTTTAACTTTACCAGAATCAGGGAAAGATTTCATAGTGTACAGTGATGCTTCTT

TGAATGGTTTGGGTTGTGTATTGATGCAAGAAGGAAAAGTAATAGCTTATGCATCTCGAC

AGTTGAAGTCACATGAACGCAACTACCCGACACACGATTTAGAGTTAGCTGCTGTAATCT

TTGCATTGAAGATTTGGATACATTACTTGTATGGTGAGAAATGTTATATTTACACTGATC

ATAAAAGTCTAAAATATCTTCTGTCACAAAAGGAGTTGAATCTGAGACAGAGACGGTGGA

TTGAACTTCTGAAAGATTATGATTGTGTTATAGATTATCATCCAGGGAAGGCAAATGTGG

TAGCAGATGCATTGAGTAGAAAAGCAGCGATTGAATTACGAGCAATGTTCGCTCGACTTA

GTATTAAGGATGATGGAAGTTTGTTAGCTGAGTTAAGAGTCAAGCCGGTGATGTTTGATC

AAATCAGAGCAGCACAGTTAAAAGATGAAAAGTTGATGAGGAAAAGAGAAATGGTACAGT

ATGGTGCGGTAGAAAATTTTAGTATTGACGAGCATGATTGTTTGAGATTTCGAAATCGAA

TTTGTGTTCCATCTACTTCTGAGATTAAAGAATTGATTCTCCGAGAAGCACATAATAGTA

TTTTTGCTTTGCACCCAGGAGGAACGAAGATGTATCGTGATCTACGAGAACTGTATTGGT

GGCCAGGAATGAAGAAAGATATAGTTGAATATGTCAGTAAATGCTTGACTTGTCAGCGGG

TAAAAGCAGAACATCAGGTACCAACAGGCCTGTTACAGCCTATTACTATTCCCGAGTGGA

AATGGGATCGCATTACCATGGATTTTGTTACGGGGTTGCCATTGTCAGTGAGTAAAAAGA

ATGCTATTTGGGTGATTGTTGATCGACTCACAAAATCAGCTCATTTTATAGCAGTTAGAA

CCGACTGGTCATTACAGAAGCTTGCCGAGGTTTATATTCGAGAAATTGTTAGATTACATG

GTATTCCGGTATCAATAATTTCAGACAGAGATCCTCGATTCACTTCGAGATTTTGGAAGC

AGCTGCATGAATCATTGGGTACTCGACTTAGTTTCAGTACAGCTTTTCATCCTCAAACTG

ATGGACAATCTGAACGAGTAATTCAGATATTAGAAGATATGCTTCGAGCTTGTGTCATTG

ATTTTGAATCAGGTTGGGAACGTTATTTACCATTGGCCGAGTTTGTTTATAATAATAGTT

TCCAATCTAGTATTCAAATGGCTCCATATGAAGCACTTTATGGTCGAAGGTGTCGATCAC

CAATATGTTGGACAAAATTAAGAGAAAGAAAAGTGATTGGGCCGGAATTGATTCAAGAGA

CAGAAGAAACAGTTAAAAAGATTAAAGATAGACTGAAAGCCGCTTTCGACAGACAGAAAT

CTTACGCAGACTTGAAACGACGAGACATTGAATATTCCGTTGGTGATAAGGTATTCCTCA

AAGTATCGCCGTGGAAGAAAATTTTGAGATTTGGTCGGAAGGGAAAATTAAGTCCGCGCT

TTATTGGGCCGTATGAGATAGTGGAAAGAATTGGGCCTGTTGCTTATCGATTATCCTTAC

CTCCAGAGTTACAGAAAATTCATGATGTTTTTCATGTTTCGATGCTTCGGAGATATAGAT

CGGATCCTTCTCATGTTATTCCCACTGAAGACATTGAACTTCGATCTGATTTAACTTATG

AAGAAGAACCAGTTCAAATATTAGCACGAGAAGTGAAAGAATTAAGAAATAAACGGGTTC

CTTTAGTACAAGTTTTATGGAGAAGCCATAGTGTGGAAGAAGCAACTTGGGAACCGGAAG

AGACAATGAGAGCACAATATCCTCATCTCTTCTCAGGTAAATTTCGAGGACGAAATTTAT

TAAGAGGGGGAGAAATGTAATGACCTAAAATTCATGGGCATCGGAAAAGTATAATATTGG

GCCTCCGTCCTAGTAAATTGAGTCCGAAAATAATTATTAGAAATATTTACGAGACTAGTA

GTGTGTTTAATTAGGTTTTAATTAAGTAAATTTAGCTTAATTTAGAGTAATTAGTAAAAA

GGATTAAATTGAATAAGAGTAAAAGTTTAATTATAGATTAAAGGAAAATAATAGGGACCA

AATGGGCAATTAAGCCACATTTGGAAGTTGAGGCGGCATAACATTGTAAAAATCTTAGAT

TTTTATATTATTATTTATATAAATATATAAATTAATTATAAAGTATATTATTAAATTAAT

TATATTATAAATATTATATTATTATATATAAAAGAAACAAAACAGAAAAGAAACAGAATA

GAAAGAACAAAGAAACAGAATAGAAGAGACGAAACAGGGGAGAAGCAGGGGAGAAAGAAG

AAAAAGAAGAAAAAAGGGGAAATAGGGTTTTTGAAGCTTGAAATTTAAATTGGTAAGTCA

AATTAGCCATTTTCTCTTAATTCTAATGTTTTAAAAGCTTTAAAACAAAGTTTTGATGGA

ATTAAGTTGATATTTTGTAAGTTCATAGGTTTTCAAGTATAGTTTATGTTGAACAAAAGA

GATGAATTAGGGATTAACTTGAAGGAATTTTAAGTTAGAATTGAAAAAGGGATTAAATTG

TAAAAGAAACTATAAGTTTTTTTTGTTTTAGGGACTAGATTGAGGAAAATTCGGAATTAA

GAAAATATGTTAAAAATTTAATAGTTAAATTTGAGTTTAAATGAAATTTGAATAGGAATA

AGGTGTGAATTGGTGTTATAAATTTGGTTATTAACATTTTTAATCAAAACAGTTTTGGGA

AGTAGCAATGGTCTGACTTTGAAAATTCACTAAAAATTTTATAAATTGAACTAGAGGATG

AACAAAATATGGAATTAAAGCTTATTGAGTCTAGTTTCTTATAGTAGAAACAATGTAAGC

AATTAATTGATGAATCAAGAGATATTTGAAATTTTGTAATACTGGTTCGGGGTGATTTCG

AGATGCCCTGTTTTAACTTTGGAAAATCATTAAAAATTGTACAAAAATTATTATGGAGTG

TAATTTATATATGTAAACTCCTTAATGAATCTAGTTTCAAAATAAATAAACAAGAACCTT

ATTCGAGTTCTGTACAATGAGATAATTTAGTTTTAGTGGAGAGAGGTCAGAACTGTCAAA

TGAAATAACAGGGGAGTATTTAACGAATAAACTGTATTAAATGGCTAGACCAAAAATTCT

GGAAATTTTATGATTAGAAGATATATGAGTCTAGTTTTAAGGAAAATTTACGGATATTAA

TTTGGAGTTTCGTAGCTCAAGATATAAATAATTTAGTAACAATGACCCAAGTAGACAGCT

TAATGGTGAAATTATATAAATACATTAAAAATGGTTAAATTTGCATGTTTAGGCTCATGA

ATTAAATTGAATCATGTTGTATTGATTATTATAAATTATTATTTTCGTAGCCAACAAAGA

ACCTAAAGCATCAGCATCGAAAGGAAAGGAGAAAGTCATCGAGGAGTAAACTCGAGAAAA

TTACGGTTTGTATTACTATAATTCAAGTTATTTATTATTAAATGTTAAATTTTAATTTAT

GTGTCTAGTAAATGAAATGTGAGGTAAGTATTATTATTATTATTATTATTATTATTATTA

TTATTATTATTATTATTATGAGTGGGAATTAAATTGAATAGTTGATATGAAATAATATTT

GAATTGTTTGTTGATTGAAAGCGGGAAATGAATTTAAATCGAATAGTGACCGATATTAAA

TTGAATGGAAATGTATTGAGTTGTGAAAATATGTTAATTGCGGATTAATTATTGATTGAA

AGGTGGAAAAATGATTGAATTGAAAGTGTGAGAAAGTGTGATTGAATTGGGATTATATGT

GATTTAAATACCCTATTAACTAGTCGGGCTGAGTCGGATATAGTTGGCATGCCATAGGAT

TGGAAGAGTTCAGGGATACTTCGACCTCGAGTCGATGAGACACTGGGTGTCACTATATTT

CTTCGGATAGATTCGATGAGGTACTGGGTACCAACTTTCTTCGGCTTTGCCGATGAGACA

CTGGGTGTCAACTATTGCTTCGAACTATCCGATGAGGCACTGGGTGCCATTCTGGTGTGT

TTGGTTGGATCCGTGTATTCGCCAAAGTCCGAGTTTTGTTAATAGGGTAAATGATGAAAT

GATAAACCGAACGAGTTGGTCAAACGAGCTATTGAAATGATATGAAAAAGTTGAATTGTG

AATTGAAATGTGAAATGAGATTGAGAAATGAACCTAAGGTTCGTGAATTATTCAAACTCA

AATTGTGGATATACGATATTGGTTGATGAATTGCTATTGTTGAAATATTTAATTTAAATT

GTATATACGATTTATGCTTTACATGTACATTATTGTTATAATTTGAATTATGGTAATACC

ACTGAGTATGAATTACTCAGCGTACGGTTGTTTCCGTGCGCAGGTCAATAGAAGTCAAAG

GTCTCGGTTCAGCATCCAGATTAATCCCGGCTTCGGCAAAACTTGGTGATGTATTTTTCC

TTTGGTAAAGGTGGCATGTACATAGATTGTGTATAAAGGTTATTATGTTTTATTATATAA

TGGTTAAAAATGTTAGTATTAAAAGTTTATGGATTTTAATGAAAGAAGTCTATCTATTTT

ATCTAATTAGTACATTGTTAAATTTTAAATTGGTATTAGATTGAGTTTGATTAGAAGTAT

TTAGAATAGAAAATGTGAATGTGAAATGAATTGGTTGAATTGATGATATTTGGGAACTAT

ATGGTTTTAATTTGC

>Deltapine5415

AGGTAATGACCCAAAATTCATGGGCATCGGAAAAGTATAATATCGGGCCTCCGTCCTAGT

AAATTGAGTCCGAAAATAATTATTAGAAATATTTACGAGACTAGTAGTGTGTTTAATTAG

GTTTTAATTAAGTAAATTTAGCTTAATTTAGAGTAATTAGTAAAAAGGATTAAATTGAAT

AAGAGTAAAAGTTTAATTATAGATTAAAGGAAAATAATAGGGACCAAATGGGCAATTAAG

CCACATTTGGAAGTTGAGGCGGCATAACATTGTAAAAATCTTAGATTTTTATATTATTAT

TTATATAAATATATAAATTAATTATAAAGTATATTATTAAATTAATTATATTATAAATAT

TATATTATTATATATAAAAGAAACAAAACAGAAAAGAAACAGAATAGAAAGAACAAAGAA

ACAGAATAGAAGAGACGAAACAGGGGAGAAGCAGGGGAGAAAGAAGAAAAAGAAGAAAAA

AGGGGAAATAGGGTTTTTGAAGCTTGAAATTTAAATTGGTAAGTCAAATTAGCCATTTTC

TCTTAATTCTAATGTTTTAAAAGCTTTAAAACAAAGTTTTGATGGAATTAAGTTGATATT

TTGTAAGTTCATAGGTTTTCAAGTATAGTTTATGTTGAACAAAAGAGATGAATTAGGGAT

TAACTTGAAGGAATTTTAAGTTAGAATTGAAAAAGGGATTAAATTGTAAAAGAAACTATA

AGTTTTTTTTGTTTTAGGGACTAGATTGAGGAAAATTCGGAATTAAGAAAATATGTTAAA

AATTTAATAGTTAAATTTGAGTTTAAATGAAATTTGAATAGGAATAAGGTGTGAATTGGT

GTTATAAATTTGGTTATTAACATTTTTAATCAAAACAGTTTTGGGAAGTAGCAATGGTCT

GACTTTGAAAATTCACTAAAAATTTTATAAATTGAACTAGAGGATGAACAAAATATGGAA

TTAAAGCTTATTGAGTCTAGTTTCTTATAGTAGAAACAATGTAAGCAATTAATTGATGAA

TCAAGAGATATTTGAAATTTTGTAATACTGGTTCGGGGTGATTTCGAGATGCCCTGTTTT

AACTTTGGAAAATCATTAAAAATTGTACAAAAATTATTATGGAGTGTAATTTATATATGT

GAACTCCTTAATGAATCTAGTTTCAAAATAAATAAACAAGAACCTTATTCGAGTTCTGTA

CAATGAGATAATTTAGTTTTAGTGGAGAGAGGTCAGAACTGTCAAATGAAATAACAGGGG

AGTATTTAACGAATAAACTGTATTAAATGGCTAGACCAAAAATTCTGGAAATTTTATGAT

TAGAAGATATATGAGTCTAGTTTTAAGGAAAATTTACGGATATTAATTTGGAGTTTCGTA

GCTCAAGATATAAATAATTTAGTAACAATGACCCAAGTAGACAGCTTAATGGTGAAATTA

TATAAATACATTAAAAATGGTTAAATTTGCATGTTTAGGCTCATGAATTAAATTGAATCA

TGTTGTATTGATTATTATAAATTATTATTTTCGTAGCCAACAAAGAACCTAAAGCATCAG

CATCGAAAGGAAAGGAGAAAGTCATCGAGGAGTAAACTCGAGAAAATTACGGTTTGTATT

ACTATAATTCAAGTTATTTATTATTAAATGTTAAATTTTAATTTATGTGTCTAGTAAATG

AAATGTGAGGTAAGTATTATTATTATTATTATTATTATTATTATTATTATTATTATTATT

ATGAGTGGGAATTAAATTGAATAGTTGATATGAAATAATATTTGAATTGTTTGTTGATTG

AAAGCGGGAAATGAATTTAAATCGAATAGTGACCGATATTAAATTGAATGGAAATGTATT

GAGTTGTGAAAGTATGTTAATTGCGGATTAATTATTGATTGAAAGGTGGAAAAATGATTG

AATTGAAAGTGTGAGAAAGTGTGATTGAATTGGGATTATATGTGATTTAAATACCCTATT

AACTAGTCGGGCTGAGTCGGATATAGTTGGCATGCCATAGGATTGGAAGAGTTCAGGGAT

ACTTCGACCTCGAGTCGATGAGACACTGGGTGATTTCTTCGGATAGATTGGATGAGGTAC

TGGGTACCAACTTTCTTCGGCTTTGCCGATGAGACACTGGGTGTCAACTATTGCTTCGAA

CTATCCGATGAGGCACTGGGTGCCATTCTGGTGTGTTTGGTTGGATCCGTGTATCCGCCA

AAGTCCGAGTTTTGTTAATAGGGTAAATGATGAAATGATAAACCGAACGAGTTGGTCAAA

CGAGCTATTGAAATGATATGAAAAAGTTGAATTGTGAATTGAAATGTGAAATGAGATTGA

GAAATGAACCTAAGGTTCGTGAATTATTCAAACTCAAATTGTGGATATACGATATTGGTT

GATGAATTGCTATTGTTGAAATATTTAATTTAAATTGTATATACGATTTATGCTTTACAT

GTACATTATTGTTATAATTTGAATTATGGTAATACCACTGAGTATGAATTACTCAGCGTA

CGGTTGTTTCCGTGCGCAGGTCAATAGAAGTCAAAGGTCTCGGTTCAGCATCCAGATTAA

TCCCGGCTTCGGCAAAACTTGGTGATGTATTTTTCCTTTGGTAAAGGTGGCATGTACATA

GATTGTGTATAAAGGTTATTATGTTTTATTATATAATGGTTAAAAATGTTAGTATTAAAA

GTTTATGGATTTTAATGAAAGAAGTCTATCTATTTTATCTAATTAGTACATTGTTAAATT

TTAAATTGGTATTGTGTAGATTGAGTTTGATTAGAAGTATTTAGAATAGAAAATGTGAAT

GTGAAATGAATTGGTTGAATTGATGATATTTGGGAACTATATGGTTTTAATTTGCAGGGG

GTTTTATGTAAAAATAAGCAGAAATGCTGCCGAAATTTTTATAAAAAAAAATGAAGTCAT

TTGGTAAACAAATTAATAAATTTTATGAATTATTTTAATATATTGGTTATTTATTTAAGA

ATTGTTGTAAATCGTTCGATACGTCCGGTAGTGCCTCGTAATTCTGTTCCGGCGACGGTT

CGGGGTTAAGGGGTGTTACATTTTATGGTATCAGAGCTATCAGGTTTAGCCGATTCTCGG

CCTAAATCGAGCTCGGAATTGAGTCTAGATGTACATGCCACTGTCGAGTTAAACTGAGTC

GGGATTTTTGGATGCTGACCTATTTGTTTGTTTTGTTTTATAGATTAAAGATGTCTGAAG

AAAGAATAAATGATACTGATGAAAGAATGTATAGTGAAGATAGAGAATTAGATGAAACAG

AATCTGTTGCACCGAGTGTGAATCCGTTAGGCAACCAACCTTCTAATGTAGAACGAGAAA

ATGTCAGAGATAGAGATGAATCCCAATTACTGAGAATTATAGCTGATGCATTACAAAGAG

TAGCAGGAACTACTCCTGTTACGACTTCAGTACCTACTGTTAGACGGGCTCCGATAAAGG

AACTGAGGAAATATGGTGCCACTGAATTTATGGGTCTAAAAGGAGTTGATCCATCCATAG

CTGAAAATTGGATGGAGTCGACTAAAAGAATTTTGCAGCAATTGGATTGTACCCCCCGAG

AGTGTTTAATCTGTGCCGTATCGTTATTACAAGGGGAGGCTTATCTATGGTGGGAATCAG

TGGTTCGACATTTACCAGAGAGTCAGATAACGTGGGATCTATTTCAGAAGGAGTTTCAAA

AGAAATATATCGGAGAGATGTATATTGAAGACAAGAAACAAGAGTTTTTGTTGCTACAAC

AGGGTGATATGTCAGTAATAGATTATGAGAGGGAATTCTCGAGACTCAGTAGATATGCCT

CCGAGTTTATTCCGACAGAAGCCGATAGTTGTAAAAGATTTTTACGGGGTTTACGAGACG

AGATCAAAGTGCAGCTAGTATCCCATCGGATCACTGAGTTAGTAGATTTGATTGAACGAG

CTAAAATGGTGGAACAAGTTCTGGGCCTCGACAAAAAGACTGAAGTTGTTAGACCAACCG

GGAAGCGTACAGGAACTACCAGTTCGAATCCTCAGCCGAAAAGACCAAAGGAATTCCAAA

GTGGTTGGAGATCCAGTTTCAGGTCAGACAGAGGTGGTAGAAATAGGGGAAAACAGACGA

TGACATCTACTGGCAGTGTGAAAGGTCCTTCCCGAGAAATAGATATTCCAGACTGCCAAC

ACTGCGGAAAGAAACACAGAGGGGAATGTTGGAAATTAACTAGAGGCTGTTTTCGATGTG

GTTCTACAGACCATTTCATCAGAGACTGTCCGAAAGTTGATAGTACTGTACCCGTGACAT

CACAGAGATCGGTATCTACAGCTAGAGGCAGAGGGTTAGGAAGAGGTGGTTCGGTTTCAA

GGGGAGGAAGTATTAGGAGAAGCAATGATATTGCTACTCAGCAGTCTGAGGCTAAAGTAC

CTGCCAGAGCTTATGTGGTCAGAACACAGGAAGAAGGTGACGCCCACGATGTAGTAACAG

GTATATTCTTACTATATTCTGAGCCTGTTTATGCTTTAATTGATCCCGGATCTTCACATT

CTTATATAAATTCAAAATTAGTTGAATTGGGAAAATTTAATTCTGAAATATCTAGAGTGA

CTGTAGAAGTGTCGAGTCCGTTGGGGCAAACAGTATTAGTGAATCAGATCTGTCCGAGAT

GCCCGTTAATTATACAAAATAAAACTTTTCCTATTGACCTGTTGATTATGCCATTTGGAG

ATTTTGATATAATACTGGGGATGGATTGGTTGGCTGAGCACGGAGTGGTATTGGATTGTT

ATAAAAAGAAGTTTAGTATTCAGACAGAAGACGGGGACAGAATTGAAGTAAATGGTATCC

GTACTAATGGGCCGACACGTATTATTTCGGCAATAAAGGCTAATAAATTGCTTCAGCGGG

GTTGTACAGCGTATTTAGCCTATGTTATTAATTCTGATTTGGTTGGTAGTCAGTGCAGTA

AGATTAGAACCGTATGTGAGTTTCCAGATGTATTTCCTGAAGAGCTACCGGGTTTACCAC

CTGACAGAGAGGTTGAATTTGCTATAGAAGTGTATCCGGGTACAGCACCAATCTCTATAC

CACCGTATCGAATGTCACCCACTGAGTTGAAAGAGTTGAAAGTGCAGTTACAGGACTTGT

CAGATCGTGGATTTATTAGACCGAGCATCTCACCTTGGGGAGCTCCAGTATTGTTTGTTA

AAAAGAAAGATGGATCGATGCGGCTTTGTATTGATTACCGGCAGTTAAACAAAGTGACGA

TCAAGAACCGGTATCCGTTACCCCGTATAGATGATTTATTTGATCAACTAAAAGGAGCTT

CAGTATTTTCAAAGATTGACTTAAGATCTGGGTATTATCAGCTGAAGGTAAAAGAAAGTG

ATGTTCCGAAGACTGCATTTCGTACTCGATATGGTCATTATGAATTTTTGGTGATGCCGT

TCGGGTTGACTAATGCTCCAGCTGCTTTTATGGATCTGATGAATCGTATTTTTCAGCCGT

ATTTAGATCAGTTTGTGGTGGTTTTTATTGATGACATCTTGGTTTATTCGAAGTCAGAGT

CAGAGCATGATCAGCATCTCAGAACCGTGCTACAAATTCTGCGAGAAAAACAGTTGTACG

GGAAACTAAGTAAATGTGAATTCTGGTTATCAGAGGTAGTATTCTTGGGACATGTTGTAT

CTGCGGATGGGATTAGAGTTGATCCGAAGAAGATCGAGGCAATTGTTCAATGGAAGGCAC

CAAAGAATGTATCAGAGGTACGCAGTTTTCTTGGTTTGGCTGGGTATTACAGAAGATTTG

TAAATGGGTTTTCGAAGATAGCTTTGCCGATGACCAAATTACTACAGAAGAATGTTCCAT

TTATCTGGGATGATCAGTGTCAGAGGAGCTTTGAAACATTGAAACAGATGTTGACAGAGG

CACCAGTTTTAACTTTACCAGAATCAGGGAAAGATTTCATAGTGTACAGTGATGCTTCTT

TGAATGGTTTGGGTTGTGTATTGATGCAAGAAGGAAAAGTAATAGCTTATGCATCTCGAC

AGTTGAAGTCACATGAACGCAACTACCCGACACACGATTTAGAGTTAGCTGCTGTAATCT

TTGCATTGAAGATTTGGATACATTACTTGTATGGTGAGAAATGTTATATTTACACTGATC

ATAAAAGTCTAAAATATCTTCTGTCACAAAAGGAGTTGAATCTGAGACAGAGACGGTGGA

TTGAACTTCTGAAAGATTATGATTGTGTTATAGATTATCATCCAGGGAAGGCAAATGTGG

TAGCAGATGCATTGAGTAGAAAAGCAGCGATTGAATTACGAGCAATGTTCGCTCGACTTA

GTATTAAGGATGATGGAAGTTTGTTAGCTGAGTTAAGAGTCAAGCCGGTGATGTTTGATC

AAATCAGAGCAGCACAGTTAAAAGATGAAAAGTTGATGAGGAAAAGAGAAATGGTACAGT

ATGGTGCGGTAGAAAATTTTAGTATTGACGAGCATGATTGTTTGAGATTTCGAAATCGAA

TTTGTGTTCCATCTACTTCTGAGATTAAAGAATTGATTCTCCGAGAAGCACATGATAGTA

TTTTTGCTTTGCACCCAGGAGGAACGAAGATGTATCGTGATCTACGAGAACTGTATTGGT

GGCCAGGAATGAAGAAAGATATAGTTGAATATGTCAGTAAATGCTTGACTTGTCAGCGGG

TAAAAGCAGAACATCAGGTACCAACAGGCCTGTTACAGCCTATTACTATTCCCGAGTGGA

AATGGGATCGCATTACCATGGATTTTGTTACGGGGTTGCCATTGTCAGTGAGTAAAAAGA

ATGCTATTTGGGTGATTGTTGATCGACTCACAAAATCAGCTCATTTTATAGCAGTTAGAA

CCGACTGGTCATTACAGAAGCTTGCCGAGGTTTATATTCGAGAAATTGTTAGATTACATG

GTATTCCGGTATCAATAATTTCAGACAGAGATCCTCGATTCACTTCGAGATTTTGGAAGC

AGCTGCATGAATCATTGGGTACTCGACTTAGTTTCAGTACAGCTTTTCATCCTCAAACTG

ATGGACAATCTGAACGAGTAATTCAGATATTAGAAGATATGCTTCGAGCTTGTGTCATTG

ATTTTGAATCAGGTTGGGAACGTTATTTACCATTGGCCGAGTTTGTTTATAATAATAGTT

TCCAATCTAGTATTCAAATGGCTCCATATGAAGCACTTTATGGTCGAAGGTGTCGATCAC

CAATATGTTGGACAAAATTAAGAGAAAGAAAAGTGATTGGGCCGGAATTGATTCAAGAGA

CAGAAGAAACAGTTAAAAAGATTAAAGATAGACTGAAAGCCGCTTTCGACAGACAGAAAT

CTTACGCAGACTTGAAACGACGAGACATTGAATATTCCGTTGGTGATAAGGTATTCCTCA

AAGTATCGCCGTGGAAGAAAATTTTGAGATTTGGTCGGAAGGGAAAATTAAGTCCGCGCT

TTATTGGGCCGTATGAGATAGTGGAAAGAATTGGGCCTGTTGCTTATCGATTATCCTTAC

CTCCAGAGTTACAGAAAATTCATGATGTTTTTCATGTTTCGATGCTTCGGAGATATAGAT

CGGATCCTTCTCATGTTATTCCCACTGAAGACATTGAACTTCGATCTGATTTAACTTATG

AAGAAGAACCAGTTCAAATATTAGCACGAGAAGTGAAAGAATTAAGAAATAAACGGGTTC

CTTTAGTACAAGTTTTATGGAGAAGCCATAGTGTGGAAGAAGCAACTTGGGAACCGGAAG

AGACAATGAGAGCACAATATCCTCATCTCTTCTCAGGTAAATTTCGAGGACGAAATTTAT

TAAGAGGGGGAGAAATGTAATGACCTAAAATTCATGGGCATCGGAAAAGTATAATATTGG

GCCTCCGTCCTAGTAAATTGAGTCCGAAAATAATTATTAGAAATATTTACGAGACTAGTA

GTGTGTTTAATTAGGTTTTAATTAAGTAAATTTAGCTTAATTTAGAGTAATTAGTAAAAA

GGATTAAATTGAATAAGAGTAAAAGTTTAATTATAGATTAAAGGAAAATAATAGGGACCA

AATGGGCAATTAAGCCACATTTGGAAGTTGAGGCGGCATAACATTGTAAAAATCTTAGAT

TTTTATATTATTATTTATATAAATATATAAATTAATTATAAAGTATATTATTAAATTAAT

TATATTATAAATATTATATTATTATATATAAAAGAAACAAAACAGAAAAGAAACAGAATA

GAAAGAACAAAGAAACAGAATAGAAGAGACGAAACAGGGGAGAAGCAGGGGAGAAAGAAG

AAAAAGAAGAAAAAAGGGGAAATAGGGTTTTTGAAGCTTGAAATTTAAATTGGTAAGTCA

AATTAGCCATTTTCTCTTAATTCTAATGTTTTAAAAGCTTTAAAACAAAGTTTTGATGGA

ATTAAGTTGATATTTTGTAAGTTCATAGGTTTTCAAGTATAGTTTATGTTGAACAAAAGA

GATGAATTAGGGATTAACTTGAAGGAATTTTAAGTTAGAATTGAAAAAGGGATTAAATTG

TAAAAGAAACTATAAGTTTTTTTTGTTTTAGGGACTAGATTGAGGAAAATTCGGAATTAA

GAAAATATGTTAAAAATTTAATAGTTAAATTTGAGTTTAAATGAAATTTGAATAGGAATA

AGGTGTGAATTGGTGTTATAAATTTGGTTATTAACATTTTTAATCAAAACAGTTTTGGGA

AGTAGCAATGGTCTGACTTTGAAAATTCACTAAAAATTTTATAAATTGAACTAGAGGATG

AACAAAATATGGAATTAAAGCTTATTGAGTCTAGTTTCTTATAGTAGAAACAATGTAAGC

AATTAATTGATGAATCAAGAGATATTTGAAATTTTGTAATACTGGTTCGGGGTGATTTCG

AGATGCCCTGTTTTAACTTTGGAAAATCATTAAAAATTGTACAAAAATTATTATGGAGTG

TAATTTATATATGTAAACTCCTTAATGAATCTAGTTTCAAAATAAATAAACAAGAACCTT

ATTCGAGTTCTGTACAATGAGATAATTTAGTTTTAGTGGAGAGAGGTCAGAACTGTCAAA

TGAAATAACAGGGGAGTATTTAACGAATAAACTGTATTAAATGGCTAGACCAAAAATTCT

GGAAATTTTATGATTAGAAGATATATGAGTCTAGTTTTAAGGAAAATTTACGGATATTAA

TTTGGAGTTTCGTAGCTCAAGATATAAATAATTTAGTAACAATGACCCAAGTAGACAGCT

TAATGGTGAAATTATATAAATACATTAAAAATGGTTAAATTTGCATGTTTAGGCTCATGA

ATTAAATTGAATCATGTTGTATTGATTATTATAAATTATTATTTTCGTAGCCAACAAAGA

ACCTAAAGCATCAGCATCGAAAGGAAAGGAGAAAGTCATCGAGGAGTAAACTCGAGAAAA

TTACGGTTTGTATTACTATAATTCAAGTTATTTATTATTAAATGTTAAATTTTAATTTAT

GTGTCTAGTAAATGAAATGTGAGGTAAGTATTATTATTATTATTATTATTATTATTATTA

TTATTATTATTATTATTATGAGTGGGAATTAAATTGAATAGTTGATATGAAATAATATTT

GAATTGTTTGTTGATTGAAAGCGGGAAATGAATTTAAATCGAATAGTGACCGATATTAAA

TTGAATGGAAATGTATTGAGTTGTGAAAATATGTTAATTGCGGATTAATTATTGATTGAA

AGGTGGAAAAATGATTGAATTGAAAGTGTGAGAAAGTGTGATTGAATTGGGATTATATGT

GATTTAAATACCCTATTAACTAGTCGGGCTGAGTCGGATATAGTTGGCATGCCATAGGAT

TGGAAGAGTTCAGGGATACTTCGACCTCGAGTCGATGAGACACTGGGTGTCACTATATTT

CTTCGGATAGATTCGATGAGGTACTGGGTACCAACTTTCTTCGGCTTTGCCGATGAGACA

CTGGGTGTCAACTATTGCTTCGAACTATCCGATGAGGCACTGGGTGCCATTCTGGTGTGT

TTGGTTGGATCCGTGTATTCGCCAAAGTCCGAGTTTTGTTAATAGGGTAAATGATGAAAT

GATAAACCGAACGAGTTGGTCAAACGAGCTATTGAAATGATATGAAAAAGTTGAATTGTG

AATTGAAATGTGAAATGAGATTGAGAAATGAACCTAAGGTTCGTGAATTATTCAAACTCA

AATTGTGGATATACGATATTGGTTGATGAATTGCTATTGTTGAAATATTTAATTTAAATT

GTATATACGATTTATGCTTTACATGTACATTATTGTTATAATTTGAATTATGGTAATACC

ACTGAGTATGAATTACTCAGCGTACGGTTGTTTCCGTGCGCAGGTCAATAGAAGTCAAAG

GTCTCGGTTCAGCATCCAGATTAATCCCGGCTTCGGCAAAACTTGGTGATGTATTTTTCC

TTTGGTAAAGGTGGCATGTACATAGATTGTGTATAAAGGTTATTATGTTTTATTATATAA

TGGTTAAAAATGTTAGTATTAAAAGTTTATGGATTTTAATGAAAGAAGTCTATCTATTTT

ATCTAATTAGTACATTGTTAAATTTTAAATTGGTATTAGATTGAGTTTGATTAGAAGTAT

TTAGAATAGAAAATGTGAATGTGAAATGAATTGGTTGAATTGATGATATTTGGGAACTAT

ATGGTTTTAATTTGC

>Deltapine5540

AGGTAATGACCCAAAATTCATGGGCATCGGAAAAGTATAATATCGGGCCTCCGTCCTAGT

AAATTGAGTCCGAAAATAATTATTAGAAATATTTACGAGACTAGTAGTGTGTTTAATTAG

GTTTTAATTAAGTAAATTTAGCTTAATTTAGAGTAATTAGTAAAAAGGATTAAATTGAAT

AAGAGTAAAAGTTTAATTATAGATTAAAGGAAAATAATAGGGACCAAATGGGCAATTAAG

CCACATTTGGAAGTTGAGGCGGCATAACATTGTAAAAATCTTAGATTTTTATATTATTAT

TTATATAAATATATAAATTAATTATAAAGTATATTATTAAATTAATTATATTATAAATAT

TATATTATTATATATAAAAGAAACAAAACAGAAAAGAAACAGAATAGAAAGAACAAAGAA

ACAGAATAGAAGAGACGAAACAGGGGAGAAGCAGGGGAGAAAGAAGAAAAAGAAGAAAAA

AGGGGAAATAGGGTTTTTGAAGCTTGAAATTTAAATTGGTAAGTCAAATTAGCCATTTTC

TCTTAATTCTAATGTTTTAAAAGCTTTAAAACAAAGTTTTGATGGAATTAAGTTGATATT

TTGTAAGTTCATAGGTTTTCAAGTATAGTTTATGTTGAACAAAAGAGATGAATTAGGGAT

TAACTTGAAGGAATTTTAAGTTAGAATTGAAAAAGGGATTAAATTGTAAAAGAAACTATA

AGTTTTTTTTGTTTTAGGGACTAGATTGAGGAAAATTCGGAATTAAGAAAATATGTTAAA

AATTTAATAGTTAAATTTGAGTTTAAATGAAATTTGAATAGGAATAAGGTGTGAATTGGT

GTTATAAATTTGGTTATTAACATTTTTAATCAAAACAGTTTTGGGAAGTAGCAATGGTCT

GACTTTGAAAATTCACTAAAAATTTTATAAATTGAACTAGAGGATGAACAAAATATGGAA

TTAAAGCTTATTGAGTCTAGTTTCTTATAGTAGAAACAATGTAAGCAATTAATTGATGAA

TCAAGAGATATTTGAAATTTTGTAATACTGGTTCGGGGTGATTTCGAGATGCCCTGTTTT

AACTTTGGAAAATCATTAAAAATTGTACAAAAATTATTATGGAGTGTAATTTATATATGT

GAACTCCTTAATGAATCTAGTTTCAAAATAAATAAACAAGAACCTTATTCGAGTTCTGTA

CAATGAGATAATTTAGTTTTAGTGGAGAGAGGTCAGAACTGTCAAATGAAATAACAGGGG

AGTATTTAACGAATAAACTGTATTAAATGGCTAGACCAAAAATTCTGGAAATTTTATGAT

TAGAAGATATATGAGTCTAGTTTTAAGGAAAATTTACGGATATTAATTTGGAGTTTCGTA

GCTCAAGATATAAATAATTTAGTAACAATGACCCAAGTAGACAGCTTAATGGTGAAATTA

TATAAATACATTAAAAATGGTTAAATTTGCATGTTTAGGCTCATGAATTAAATTGAATCA

TGTTGTATTGATTATTATAAATTATTATTTTCGTAGCCAACAAAGAACCTAAAGCATCAG

CATCGAAAGGAAAGGAGAAAGTCATCGAGGAGTAAACTCGAGAAAATTACGGTTTGTATT

ACTATAATTCAAGTTATTTATTATTAAATGTTAAATTTTAATTTATGTGTCTAGTAAATG

AAATGTGAGGTAAGTATTATTATTATTATTATTATTATTATTATTATTATTATTATTATT

ATGAGTGGGAATTAAATTGAATAGTTGATATGAAATAATATTTGAATTGTTTGTTGATTG

AAAGCGGGAAATGAATTTAAATCGAATAGTGACCGATATTAAATTGAATGGAAATGTATT

GAGTTGTGAAAATATGTTAATTGCGGATTAATTATTGATTGAAAGGTGGAAAAATGATTG

AATTGAAAGTGTGAGAAAGTGTGATTGAATTGGGATTATATGTGATTTAAATACCCTATT

AACTAGTCGGGCTGAGTCGGATATAGTTGGCATGCCATAGGATTGGAAGAGTTCAGGGAT

ACTTCGACCTCGAGTCGATGAGACACTGGGTGATTTCTTCGGATAGATTGGATGAGGTAC

TGGGTACCAACTTTCTTCGGCTTTGCCGATGAGACACTGGGTGTCAACTATTGCTTCGAA

CTATCCGATGAGGCACTGGGTGCCATTCTGGTGTGTTTGGTTGGATCCGTGTATCCGCCA

AAGTCCGAGTTTTGTTAATAGGGTAAATGATGAAATGATAAACCGAACGAGTTGGTCAAA

CGAGCTATTGAAATGATATGAAAAAGTTGAATTGTGAATTGAAATGTGAAATGAGATTGA

GAAATGAACCTAAGGTTCGTGAATTATTCAAACTCAAATTGTGGATATACGATATTGGTT

GATGAATTGCTATTGTTGAAATATTTAATTTAAATTGTATATACGATTTATGCTTTACAT

GTACATTATTGTTATAATTTGAATTATGGTAATACCACTGAGTATGAATTACTCAGCGTA

CGGTTGTTTCCGTGCGCAGGTCAATAGAAGTCAAAGGTCTCGGTTCAGCATCCAGATTAA

TCCCGGCTTCGGCAAAACTTGGTGATGTATTTTTCCTTTGGTAAAGGTGGCATGTACATA

GATTGTGTATAAAGGTTATTATGTTTTATTATATAATGGTTAAAAATGTTAGTATTAAAA

GTTTATGGATTTTAATGAAAGAAGTCTATCTATTTTATCTAATTAGTACATTGTTAAATT

TTAAATTGGTATTGTGTAGATTGAGTTTGATTAGAAGTATTTAGAATAGAAAATGTGAAT

GTGAAATGAATTGGTTGAATTGATGATATTTGGGAACTATATGGTTTTAATTTGCAGGGG

GTTTTATGTAAAAATAAGCAGAAATGCTGCCGAAATTTTTATAAAAAAAAATGAAGTCAT

TTGGTAAACAAATTAATAAATTTTATGAATTATTTTAATATATTGGTTATTTATTTAAGA

ATTGTTGTAAATCGTTCGATACGTCCGGTAGTGCCTCGTAATTCTGTTCCGGCGACGGTT

CGGGGTTAAGGGGTGTTACATTTTATGGTATCAGAGCTATCAGGTTTAGCCGATTCTCGG

CCTAAATCGAGCTCGGAATTGAGTCTAGATGTACATGCCACTGTCGAGTTAAACTGAGTC

GGGATTTTTGGATGCTGACCTATTTGTTTGTTTTGTTTTATAGATTAAAGATGTCTGAAG

AAAGAATAAATGATACTGATGAAAGAATGTATAGTGAAGATAGAGAATTAGATGAAACAG

AATCTGTTGCACCGAGTGTGAATCCGTTAGGCAACCAACCTTCTAATGTAGAACGAGAAA

ATGTCAGAGATAGAGATGAATCCCAATTACTGAGAATTATAGCTGATGCATTACAAAGAG

TAGCAGGAACTACTCCTGTTACGACTTCAGTACCTACTGTTAGACGGGCTCCGATAAAGG

AACTGAGGAAATATGGTGCCACTGAATTTATGGGTCTAAAAGGAGTTGATCCATCCATAG

CTGAAAATTGGATGGAGTCGACTAAAAGAATTTTGCAGCAATTGGATTGTACCCCCCGAG

AGTGTTTAATCTGTGCCGTATCGTTATTACAAGGGGAGGCTTATCTATGGTGGGAATCAG

TGGTTCGACATTTACCAGAGAGTCAGATAACGTGGGATCTATTTCAGAAGGAGTTTCAAA

AGAAATATATCGGAGAGATGTATATTGAAGACAAGAAACAAGAGTTTTTGTTGCTACAAC

AGGGTGATATGTCAGTAATAGATTATGAGAGGGAATTCTCGAGACTCAGTAGATATGCCT

CCGAGTTTATTCCGACAGAAGCCGATAGTTGTAAAAGATTTTTACGGGGTTTACGAGACG

AGATCAAAGTGCAGCTAGTATCCCATCGGATCACTGAGTTAGTAGATTTGATTGAACGAG

CTAAAATGGTGGAACAAGTTCTGGGCCTCGACAAAAAGACTGAAGTTGTTAGACCAACCG

GGAAGCGTACAGGAACTACCAGTTCGAATCCTCAGCCGAAAAGACCAAAGGAATTCCAAA

GTGGTTGGAGATCCAGTTTCAGGTCAGACAGAGGTGGTAGAAATAGGGGAAAACAGACGA

TGACATCTACTGGCAGTGTGAAAGGTCCTTCCCGAGAAATAGATATTCCAGACTGCCAAC

ACTGCGGAAAGAAACACAGAGGGGAATGTTGGAAATTAACTAGAGGCTGTTTTCGATGTG

GTTCTACAGACCATTTCATCAGAGACTGTCCGAAAGTTGATAGTACTGTACCCGTGACAT

CACAGAGATCGGTATCTACAGCTAGAGGCAGAGGGTTAGGAAGAGGTGGTTCGGTTTCAA

GGGGAGGAAGTATTAGGAGAAGCAATGATATTGCTACTCAGCAGTCTGAGGCTAAAGTAC

CTGCCAGAGCTTATGTGGTCAGAACACAGGAAGAAGGTGACGCCCACGATGTAGTAACAG

GTATATTCTTACTATATTCTGAGCCTGTTTATGCTTTAATTGATCCCGGATCTTCACATT

CTTATATAAATTCAAAATTAGTTGAATTGGGAAAATTTAATTCTGAAATATCTAGAGTGG

CTGTAGAAGTATCGAGTCCGTTGGGGCAAACAGTATTAGTGAATCAGATCTGTCCGAGAT

GCCCGTTAATTATACAAAATAAAACTTTTCCTATTGACCTGTTGATTATGCCATTTGGAG

ATTTTGATATAATACTGGGGATGGATTGGTTGGCTGAGCACGGAGTGGTATTGGATTGTT

ATAAAAAGAAGTTTAGTATTCAGACAGAAGACGGGGACAGAATTGAAGTAAATGGTATCC

GTACTAATGGGCCGACACGTATTATTTCGGCAATAAAGGCTAATAAATTGCTTCAGCGGG

GTTGTACAGCGTATTTAGCCTATGTTATTAATTCTGATTTGGTTGGTAGTCAGTGCAGTA

AGATTAGAACCGTATGTGAGTTTCCAGATGTATTTCCTGAAGAGCTACCGGGTTTACCAC

CTGACAGAGAGGTTGAATTTGCTATAGAAGTGTATCCGGGTACAGCACCAATCTCTATAC

CACCGTATCGAATGTCACCCACTGAGTTGAAAGAGTTGAAAGTGCAGTTACAGGACTTGT

CAGATCGTGGATTTATTAGACCGAGCATCTCACCTTGGGGAGCTCCAGTATTGTTTGTTA

AAAAGAAAGATGGATCGATGCGGCTTTGTATTGATTACCGGCAGTTAAACAAAGTGACGA

TCAAGAACCGGTATCCGTTACCCCGTATAGATGATTTATTTGATCAACTAAAAGGAGCTT

CAGTATTTTCAAAGATTGACTTAAGATCTGGGTATTATCAGCTGAAGGTAAAAGAAAGTG

ATGTTCCGAAGACTGCATTTCGTACTCGATATGGTCATTATGAATTTTTGGTGATGCCGT

TCGGGTTGACTAATGCTCCAGCTGCTTTTATGGATCTGATGAATCGTATTTTTCAGCCGT

ATTTAGATCAGTTTGTGGTGGTTTTTATTGATGACATCTTGGTTTATTCGAAGTCAGAGT

CAGAGCATGATCAGCATCTCAGAACCGTGCTACAAATTCTGCGAGAAAAACAGTTGTACG

GGAAACTAAGTAAATGTGAATTCTGGTTATCAGAGGTAGTATTCTTGGGACATGTTGTAT

CTGCGGATGGGATTAGAGTTGATCCGAAGAAGATCGAGGCAATTGTTCAATGGAAGGCAC

CAAAGAATGTATCAGAGGTACGCAGTTTTCTTGGTTTGGCTGGGTATTACAGAAGATTTG

TAAATGGGTTTTCGAAGATAGCTTTGCCGATGACCAAATTACTACAGAAGAATGTTCCAT

TTATCTGGGATGATCAGTGTCAGAGGAGCTTTGAAACATTGAAACAGATGTTGACAGAGG

CACCAGTTTTAACTTTACCAGAATCAGGGAAAGATTTCATAGTGTACAGTGATGCTTCTT

TGAATGGTTTGGGTTGTGTATTGATGCAAGAAGGAAAAGTAATAGCTTATGCATCTCGAC

AGTTGAAGTCACATGAACGCAACTACCCGACACACGATTTAGAGTTAGCTGCTGTAATCT

TTGCATTGAAGATTTGGATACATTACTTGTATGGTGAGAAATGTTATATTTACACTGATC

ATAAAAGTCTAAAATATCTTCTGTCACAAAAGGAGTTGAATCTGAGACAGAGACGGTGGA

TTGAACTTCTGAAAGATTATGATTGTGTTATAGATTATCATCCAGGGAAGGCAAATGTGG

TAGCAGATGCATTGAGTAGAAAAGCAGCGATTGAATTACGAGCAATGTTCGCTCGACTTA

GTATTAAGGATGATGGAAGTTTGTTAGCTGAGTTAAGAGTCAAGCCGGTGATGTTTGATC

AAATCAGAGCAGCACAGTTAAAAGATGAAAAGTTGATGAGGAAAAGAGAAATGGTACAGT

ATGGTGCGGTAGAAAATTTTAGTATTGACGAGCATGATTGTTTGAGATTTCGAAATCGAA

TTTGTGTTCCATCTACTTCTGAGATTAAAGAATTGATTCTCCGAGAAGCACATAATAGTA

TTTTTGCTTTGCACCCAGGAGGAACGAAGATGTATCGTGATCTACGAGAACTGTATTGGT

GGCCAGGAATGAAGAAAGATATAGTTGAATATGTCAGTAAATGCTTGACTTGTCAGCGGG

TAAAAGCAGAACATCAGGTACCAACAGGCCTGTTACAGCCTATTACTATTCCCGAGTGGA

AATGGGATCGCATTACCATGGATTTTGTTACGGGGTTGCCATTGTCAGTGAGTAAAAAGA

ATGCTATTTGGGTGATTGTTGATCGACTCACAAAATCAGCTCATTTTATAGCAGTTAGAA

CCGACTGGTCATTACAGAAGCTTGCCGAGGTTTATATTCGAGAAATTGTTAGATTACATG

GTATTCCGGTATCAATAATTTCAGACAGAGATCCTCGATTCACTTCGAGATTTTGGAAGC

AGCTGCATGAATCATTGGGTACTCGACTTAGTTTCAGTACAGCTTTTCATCCTCAAACTG

ATGGACAATCTGAACGAGTAATTCAGATATTAGAAGATATGCTTCGAGCTTGTGTCATTG

ATTTTGAATCAGGTTGGGAACGTTATTTACCATTGGCCGAGTTTGTTTATAATAATAGTT

TCCAATCTAGTATTCAAATGGCTCCATATGAAGCACTTTATGGTCGAAGGTGTCGATCAC

CAATATGTTGGACAAAATTAAGAGAAAGAAAAGTGATTGGGCCGGAATTGATTCAAGAGA

CAGAAGAAACAGTTAAAAAGATTAAAGATAGACTGAAAGCCGCTTTCGACAGACAGAAAT

CTTACGCAGACTTGAAACGACGAGACATTGAATATTCCGTTGGTGATAAGGTATTCCTCA

AAGTATCGCCGTGGAAGAAAATTTTGAGATTTGGTCGGAAGGGAAAATTAAGTCCGCGCT

TTATTGGGCCGTATGAGATAGTGGAAAGAATTGGGCCTGTTGCTTATCGATTATCCTTAC

CTCCAGAGTTACAGAAAATTCATGATGTTTTTCATGTTTCGATGCTTCGGAGATATAGAT

CGGATCCTTCTCATGTTATTCCCACTGAAGACATTGAACTTCGATCTGATTTAACTTATG

AAGAAGAACCAGTTCAAATATTAGCACGAGAAGTGAAAGAATTAAGAAATAAACGGGTTC

CTTTAGTACAAGTTTTATGGAGAAGCCATAGTGTGGAAGAAGCAACTTGGGAACCGGAAG

AGACAATGAGAGCACAATATCCTCATCTCTTCTCAGGTAAATTTCGAGGACGAAATTTAT

TAAGAGGGGGAGAAATGTAATGACCTAAAATTCATGGGCATCGGAAAAGTATAATATTGG

GCCTCCGTCCTAGTAAATTGAGTCCGAAAATAATTATTAGAAATATTTACGAGACTAGTA

GTGTGTTTAATTAGGTTTTAATTAAGTAAATTTAGCTTAATTTAGAGTAATTAGTAAAAA

GGATTAAATTGAATAAGAGTAAAAGTTTAATTATAGATTAAAGGAAAATAATAGGGACCA

AATGGGCAATTAAGCCACATTTGGAAGTTGAGGCGGCATAACATTGTAAAAATCTTAGAT

TTTTATATTATTATTTATATAAATATATAAATTAATTATAAAGTATATTATTAAATTAAT

TATATTATAAATATTATATTATTATATATAAAAGAAACAAAACAGAAAAGAAACAGAATA

GAAAGAACAAAGAAACAGAATAGAAGAGACGAAACAGGGGAGAAGCAGGGGAGAAAGAAG

AAAAAGAAGAAAAAAGGGGAAATAGGGTTTTTGAAGCTTGAAATTTAAATTGGTAAGTCA

AATTAGCCATTTTCTCTTAATTCTAATGTTTTAAAAGCTTTAAAACAAAGTTTTGATGGA

ATTAAGTTGATATTTTGTAAGTTCATAGGTTTTCAAGTATAGTTTATGTTGAACAAAAGA

GATGAATTAGGGATTAACTTGAAGGAATTTTAAGTTAGAATTGAAAAAGGGATTAAATTG

TAAAAGAAACTATAAGTTTTTTTTGTTTTAGGGACTAGATTGAGGAAAATTCGGAATTAA

GAAAATATGTTAAAAATTTAATAGTTAAATTTGAGTTTAAATGAAATTTGAATAGGAATA

AGGTGTGAATTGGTGTTATAAATTTGGTTATTAACATTTTTAATCAAAACAGTTTTGGGA

AGTAGCAATGGTCTGACTTTGAAAATTCACTAAAAATTTTATAAATTGAACTAGAGGATG

AACAAAATATGGAATTAAAGCTTATTGAGTCTAGTTTCTTATAGTAGAAACAATGTAAGC

AATTAATTGATGAATCAAGAGATATTTGAAATTTTGTAATACTGGTTCGGGGTGATTTCG

AGATGCCCTGTTTTAACTTTGGAAAATCATTAAAAATTGTACAAAAATTATTATGGAGTG

TAATTTATATATGTAAACTCCTTAATGAATCTAGTTTCAAAATAAATAAACAAGAACCTT

ATTCGAGTTCTGTACAATGAGATAATTTAGTTTTAGTGGAGAGAGGTCAGAACTGTCAAA

TGAAATAACAGGGGAGTATTTAACGAATAAACTGTATTAAATGGCTAGACCAAAAATTCT

GGAAATTTTATGATTAGAAGATATATGAGTCTAGTTTTAAGGAAAATTTACGGATATTAA

TTTGGAGTTTCGTAGCTCAAGATATAAATAATTTAGTAACAATGACCCAAGTAGACAGCT

TAATGGTGAAATTATATAAATACATTAAAAATGGTTAAATTTGCATGTTTAGGCTCATGA

ATTAAATTGAATCATGTTGTATTGATTATTATAAATTATTATTTTCGTAGCCAACAAAGA

ACCTAAAGCATCAGCATCGAAAGGAAAGGAGAAAGTCATCGAGGAGTAAACTCGAGAAAA

TTACGGTTTGTATTACTATAATTCAAGTTATTTATTATTAAATGTTAAATTTTAATTTAT

GTGTCTAGTAAATGAAATGTGAGGTAAGTATTATTATTATTATTATTATTATTATTATTA

TTATTATTATTATTATTATGAGTGGGAATTAAATTGAATAGTTGATATGAAATAATATTT

GAATTGTTTGTTGATTGAAAGCGGGAAATGAATTTAAATCGAATAGTGACCGATATTAAA

TTGAATGGAAATGTATTGAGTTGTGAAAATATGTTAATTGCGGATTAATTATTGATTGAA

AGGTGGAAAAATGATTGAATTGAAAGTGTGAGAAAGTGTGATTGAATTGGGATTATATGT

GATTTAAATACCCTATTAACTAGTCGGGCTGAGTCGGATATAGTTGGCATGCCATAGGAT

TGGAAGAGTTCAGGGATACTTCGACCTCGAGTCGATGAGACACTGGGTGTCACTATATTT

CTTCGGATAGATTCGATGAGGTACTGGGTACCAACTTTCTTCGGCTTTGCCGATGAGACA

CTGGGTGTCAACTATTGCTTCGAACTATCCGATGAGGCACTGGGTGCCATTCTGGTGTGT

TTGGTTGGATCCGTGTATTCGCCAAAGTCCGAGTTTTGTTAATAGGGTAAATGATGAAAT

GATAAACCGAACGAGTTGGTCAAACGAGCTATTGAAATGATATGAAAAAGTTGAATTGTG

AATTGAAATGTGAAATGAGATTGAGAAATGAACCTAAGGTTCGTGAATTATTCAAACTCA

AATTGTGGATATACGATATTGGTTGATGAATTGCTATTGTTGAAATATTTAATTTAAATT

GTATATACGATTTATGCTTTACATGTACATTATTGTTATAATTTGAATTATGGTAATACC

ACTGAGTATGAATTACTCAGCGTACGGTTGTTTCCGTGCGCAGGTCAATAGAAGTCAAAG

GTCTCGGTTCAGCATCCAGATTAATCCCGGCTTCGGCAAAACTTGGTGATGTATTTTTCC

TTTGGTAAAGGTGGCATGTACATAGATTGTGTATAAAGGTTATTATGTTTTATTATATAA

TGGTTAAAAATGTTAGTATTAAAAGTTTATGGATTTTAATGAAAGAAGTCTATCTATTTT

ATCTAATTAGTACATTGTTAAATTTTAAATTGGTATTAGATTGAGTTTGATTAGAAGTAT

TTAGAATAGAAAATGTGAATGTGAAATGAATTGGTTGAATTGATGATATTTGGGAACTAT

ATGGTTTTAATTTGC

>Deltapine55

ATGTAATGACCCAAAATTCATGGGCATCGGAAAAGTATAATATCGGGCCTCCGTCCTAGT

AAATTGAGTCCGAAAATAATTATTAGAAATATTTACGAGACTAGTAGTGTGTTTAATTAG

GTTTTAATTAAGTAAATTTAGCTTAATTTAGAGTAATTAGTAAAAAGGATTAAATTGAAT

AAGAGTAAAAGTTTAATTATAGATTAAAGGAAAATAATAGGGACCAAATGGGCAATTAAG

CCACATTTGGAAGTTGAGGCGGCATAACATTGTAAAAATCTTAGATTTTTATATTATTAT

TTATATAAATATATAAATTAATTATAAAGTATATTATTAAATTAATTATATTATAAATAT

TATATTATTATATATAAAAGAAACAAAACAGAAAAGAAACAGAATAGAAAGAACAAAGAA

ACAGAATAGAAGAGACGAAACAGGGGAGAAGCAGGGGAGAAAGAAGAAAAAGAAGAAAAA

AGGGGAAATAGGGTTTTTGAAGCTTGAAATTTAAATTGGTAAGTCAAATTAGCCATTTTC

TCTTAATTCTAATGTTTTAAAAGCTTTAAAACAAAGTTTTGATGGAATTAAGTTGATATT

TTGTAAGTTCATAGGTTTTCAAGTATAGTTTATGTTGAACAAAAGAGATGAATTAGGGAT

TAACTTGAAGGAATTTTAAGTTAGAATTGAAAAAGGGATTAAATTGTAAAAGAAACTATA

AGTTTTTTTTGTTTTAGGGACTAGATTGAGGAAAATTCGGAATTAAGAAAATATGTTAAA

AATTTAATAGTTAAATTTGAGTTTAAATGAAATTTGAATAGGAATAAGGTGTGAATTGGT

GTTATAAATTTGGTTATTAACATTTTTAATCAAAACAGTTTTGGGAAGTAGCAATGGTCT

GACTTTGAAAATTCACTAAAAATTTTATAAATTGAACTAGAGGATGAACAAAATATGGAA

TTAAAGCTTATTGAGTCTAGTTTCTTATAGTAGAAACAATGTAAGCAATTAATTGATGAA

TCAAGAGATATTTGAAATTTTGTAATACTGGTTCGGGGTGATTTCGAGATGCCCTGTTTT

AACTTTGGAAAATCATTAAAAATTGTACAAAAATTATTATGGAGTGTAATTTATATATGT

GAACTCCTTAATGAATCTAGTTTCAAAATAAATAAACAAGAACCTTATTCGAGTTCTGTA

CAATGAGATAATTTAGTTTTAGTGGAGAGAGGTCAGAACTGTCAAATGAAATAACAGGGG

AGTATTTAACGAATAAACTGTATTAAATGGCTAGACCAAAAATTCTGGAAATTTTATGAT

TAGAAGATATATGAGTCTAGTTTTAAGGAAAATTTACGGATATTAATTTGGAGTTTCGTA

GCTCAAGATATAAATAATTTAGTAACAATGACCCAAGTAGACAGCTTAATGGTGAAATTA

TATAAATACATTAAAAATGGTTAAATTTGCATGTTTAGGCTCATGAATTAAATTGAATCA

TGTTGTATTGATTATTATAAATTATTATTTTCGTAGCCAACAAAGAACCTAAAGCATCAG

CATCGAAAGGAAAGGAGAAAGTCATCGAGGAGTAAACTCGAGAAAATTACGGTTTGTATT

ACTATAATTCAAGTTATTTATTATTAAATGTTAAATTTTAATTTATGTGTCTAGTAAATG

AAATGTGAGGTAAGTATTATTATTATTATTATTATTATTATTATTATTATTATTATTATT

ATGAGTGGGAATTAAATTGAATAGTTGATATGAAATAATATTTGAATTGTTTGTTGATTG

AAAGCGGGAAATGAATTTAAATCGAATAGTGACCGATATTAAATTGAATGGAAATGTATT

GAGTTGTGAAAATATGTTAATTGCGGATTAATTATTGATTGAAAGGTGGAAAAATGATTG

AATTGAAAGTGTGAGAAAGTGTGATTGAATTGGGATTATATGTGATTTAAATACCCTATT

AACTAGTCGGGCTGAGTCGGATATAGTTGGCATGCCATAGGATTGGAAGAGTTCAGGGAT

ACTTCGACCTCGAGTCGATGAGACACTGGGTGATTTCTTCGGATAGATTGGATGAGGTAC

TGGGTACCAACTTTCTTCGGCTTTGCCGATGAGACACTGGGTGTCAACTATTGCTTCGAA

CTATCCGATGAGGCACTGGGTGCCATTCTGGTGTGTTTGGTTGGATCCGTGTATCCGCCA

AAGTCCGAGTTTTGTTAATAGGGTAAATGATGAAATGATAAACCGAACGAGTTGGTCAAA

CGAGCTATTGAAATGATATGAAAAAGTTGAATTGTGAATTGAAATGTGAAATGAGATTGA

GAAATGAACCTAAGGTTCGTGAATTATTCAAACTCAAATTGTGGATATACGATATTGGTT

GATGAATTGCTATTGTTGAAATATTTAATTTAAATTGTATATACGATTTATGCTTTACAT

GTACATTATTGTTATAATTTGAATTATGGTAATACCACTGAGTATGAATTACTCAGCGTA

CGGTTGTTTCCGTGCGCAGGTCAATAGAAGTCAAAGGTCTCGGTTCAGCATCCAGATTAA

TCCCGGCTTCGGCAAAACTTGGTGATGTATTTTTCCTTTGGTAAAGGTGGCATGTACATA

GATTGTGTATAAAGGTTATTATGTTTTATTATATAATGGTTAAAAATGTTAGTATTAAAA

GTTTATGGATTTTAATGAAAGAAGTCTATCTATTTTATCTAATTAGTACATTGTTAAATT

TTAAATTGGTATTGTGTAGATTGAGTTTGATTAGAAGTATTTAGAATAGAAAATGTGAAT

GTGAAATGAATTGGTTGAATTGATGATATTTGGGAACTATATGGTTTTAATTTGCAGGGG

GTTTTATGTAAAAATAAGCAGAAATGCTGCCGAAATTTTTATAAAAAAAAATGAAGTCAT

TTGGTAAACAAATTAATAAATTTTATGAATTATTTTAATATATTGGTTATTTATTTAAGA

ATTGTTGTAAATCGTTCGATACGTCCGGTAGTGCCTCGTAATTCTGTTCCGGCGACGGTT

CGGGGTTAAGGGGTGTTACATTTTATGGTATCAGAGCTATCAGGTTTAGCCGATTCTCGG

CCTAAATCGAGCTCGGAATTGAGTCTAGATGTACATGCCACTGTCGAGTTAAACTGAGTC

GGGATTTTTGGATGCTGACCTATTTGTTTGTTTTGTTTTATAGATTAAAGATGTCTGAAG

AAAGAATAAATGATACTGATGAAAGAATGTATAGTGAAGATAGAGAATTAGATGAAACAG

AATCTGTTGCACCGAGTGTGAATCCGTTAGGCAACCAACCTTCTAATGTAGAACGAGAAA

ATGTCAGAGATAGAGATGAATCCCAATTACTGAGAATTATAGCTGATGCATTACAAAGAG

TAGCAGGAACTACTCCTGTTACGACTTCAGTACCTACTGTTAGACGGGCTCCGATAAAGG

AACTGAGGAAATATGGTGCCACTGAATTTATGGGTCTAAAAGGAGTTGATCCATCCATAG

CTGAAAATTGGATGGAGTCGACTAAAAGAATTTTGCAGCAATTGGATTGTACCCCCCGAG

AGTGTTTAATCTGTGCCGTATCGTTATTACAAGGGGAGGCTTATCTATGGTGGGAATCAG

TGGTTCGACATTTACCAGAGAGTCAGATAACGTGGGATCTATTTCAGAAGGAGTTTCAAA

AGAAATATATCGGAGAGATGTATATTGAAGACAAGAAACAAGAGTTTTTGTTGCTACAAC

AGGGTGATATGTCAGTAATAGATTATGAGAGGGAATTCTCGAGACTCAGTAGATATGCCT

CCGAGTTTATTCCGACAGAAGCCGATAGTTGTAAAAGATTTTTACGGGGTTTACGAGACG

AGATCAAAGTGCAGCTAGTATCCCATCGGATCACTGAGTTAGTAGATTTGATTGAACGAG

CTAAAATGGTGGAACAAGTTCTGGGCCTCGACAAAAAGACTGAAGTTGTTAGACCAACCG

GGAAGCGTACAGGAACTACCAGTTCGAATCCTCAGCCGAAAAGACCAAAGGAATTCCAAA

GTGGTTGGAGATCCAGTTTCAGGTCAGACAGAGGTGGTAGAAATAGGGGAAAACAGACGA

TGACATCTACTGGCAGTGTGAAAGGTCCTTCCCGAGAAATAGATATTCCAGACTGCCAAC

ACTGCGGAAAGAAACACAGAGGGGAATGTTGGAAATTAACTAGAGGCTGTTTTCGATGTG

GTTCTACAGACCATTTCATCAGAGACTGTCCGAAAGTTGATAGTACTGTACCCGTGACAT

CACAGAGATCGGTATCTACAGCTAGAGGCAGAGGGTTAGGAAGAGGTGGTTCGGTTTCAA

GGGGAGGAAGTATTAGGAGAAGCAATGATATTGCTACTCAGCAGTCTGAGGCTAAAGTAC

CTGCCAGAGCTTATGTGGTCAGAACACAGGAAGAAGGTGACGCCCACGATGTAGTAACAG

GTATATTCTTACTATATTCTGAGCCTGTTTATGCTTTAATTGATCCCGGATCTTCACATT

CTTATATAAATTCAAAATTAGTTGAATTGGGAAAATTTAATTCTGAAATATCTAGAGTGA

CTGTAGAAGTGTCGAGTCCGTTGGGGCAAACAGTATTAGTGAATCAGATCTGTCCGAGAT

GCCCGTTAATTATACAAAATAAAACTTTTCCTATTGACCTGTTGATTATGCCATTTGGAG

ATTTTGATATAATACTGGGGATGGATTGGTTGGCTGAGCACGGAGTGGTATTGGATTGTT

ATAAAAAGAAGTTTAGTATTCAGACAGAAGACGGGGACAGAATTGAAGTAAATGGTATCC

GTACTAATGGGCCGACACGTATTATTTCGGCAATAAAGGCTAATAAATTGCTTCAGCGGG

GTTGTACAGCGTATTTAGCCTATGTTATTAATTCTGATTTGGTTGGTAGTCAGTGCAGTA

AGATTAGAACCGTATGTGAGTTTCCAGATGTATTTCCTGAAGAGCTACCGGGTTTACCAC

CTGACAGAGAGGTTGAATTTGCTATAGAAGTGTATCCGGGTACAGCACCAATCTCTATAC

CACCGTATCGAATGTCACCCACTGAGTTGAAAGAGTTGAAAGTGCAGTTACAGGACTTGT

CAGATCGTGGATTTATTAGACCGAGCATCTCACCTTGGGGAGCTCCAGTATTGTTTGTTA

AAAAGAAAGATGGATCGATGCGGCTTTGTATTGATTACCGGCAGTTAAACAAAGTGACGA

TCAAGAACCGGTATCCGTTACCCCGTATAGATGATTTATTTGATCAACTAAAAGGAGCTT

CAGTATTTTCAAAGATTGACTTAAGATCTGGGTATTATCAGCTGAAGGTAAAAGAAAGTG

ATGTTCCGAAGACTGCATTTCGTACTCGATATGGTCATTATGAATTTTTGGTGATGCCGT

TCGGGTTGACTAATGCTCCAGCTGCTTTTATGGATCTGATGAATCGTATTTTTCAGCCGT

ATTTAGATCAGTTTGTGGTGGTTTTTATTGATGACATCTTGGTTTATTCGAAGTCAGAGT

CAGAGCATGATCAGCATCTCAGAACCGTGCTACAAATTCTGCGAGAAAAACAGTTGTACG

GGAAACTAAGTAAATGTGAATTCTGGTTATCAGAGGTAGTATTCTTGGGACATGTTGTAT

CTGCGGATGGGATTAGAGTTGATCCGAAGAAGATCGAGGCAATTGTTCAATGGAAGGCAC

CAAAGAATGTATCAGAGGTACGCAGTTTTCTTGGTTTGGCTGGGTATTACAGAAGATTTG

TAAATGGGTTTTCGAAGATAGCTTTGCCGATGACCAAATTACTACAGAAGAATGTTCCAT

TTATCTGGGATGATCAGTGTCAGAGGAGCTTTGAAACATTGAAACAGATGTTGACAGAGG

CACCAGTTTTAACTTTACCAGAATCAGGGAAAGATTTCATAGTGTACAGTGATGCTTCTT

TGAATGGTTTGGGTTGTGTATTGATGCAAGAAGGAAAAGTAATAGCTTATGCATCTCGAC

AGTTGAAGTCACATGAACGCAACTACCCGACACACGATTTAGAGTTAGCTGCTGTAATCT

TTGCATTGAAGATTTGGATACATTACTTGTATGGTGAGAAATGTTATATTTACACTGATC

ATAAAAGTCTAAAATATCTTCTGTCACAAAAGGAGTTGAATCTGAGACAGAGACGGTGGA

TTGAACTTCTGAAAGATTATGATTGTGTTATAGATTATCATCCAGGGAAGGCAAATGTGG

TAGCAGATGCATTGAGTAGAAAAGCAGCGATTGAATTACGAGCAATGTTCGCTCGACTTA

GTATTAAGGATGATGGAAGTTTGTTAGCTGAGTTAAGAGTCAAGCCGGTGATGTTTGATC

AAATCAGAGCAGCACAGTTAAAAGATGAAAAGTTGATGAGGAAAAGAGAAATGGTACAGT

ATGGTGCGGTAGAAAATTTTAGTATTGACGAGCATGATTGTTTGAGATTTCGAAATCGAA

TTTGTGTTCCATCTACTTCTGAGATTAAAGAATTGATTCTCCGAGAAGCACATAATAGTA

TTTTTGCTTTGCACCCAGGAGGAACGAAGATGTATCGTGATCTACGAGAACTGTATTGGT

GGCCAGGAATGAAGAAAGATATAGTTGAATATGTCAGTAAATGCTTGACTTGTCAGCGGG

TAAAAGCAGAACATCAGGTACCAACAGGCCTGTTACAGCCTATTACTATTCCCGAGTGGA

AATGGGATCGCATTACCATGGATTTTGTTACGGGGTTGCCATTGTCAGTGAGTAAAAAGA

ATGCTATTTGGGTGATTGTTGATCGACTCACAAAATCAGCTCATTTTATAGCAGTTAGAA

CCGACTGGTCATTACAGAAGCTTGCCGAGGTTTATATTCGAGAAATTGTTAGATTACATG

GTATTCCGGTATCAATAATTTCAGACAGAGATCCTCGATTCACTTCGAGATTTTGGAAGC

AGCTGCATGAATCATTGGGTACTCGACTTAGTTTCAGTACAGCTTTTCATCCTCAAACTG

ATGGACAATCTGAACGAGTAATTCAGATATTAGAAGATATGCTTCGAGCTTGTGTCATTG

ATTTTGAATCAGGTTGGGAACGTTATTTACCATTGGCCGAGTTTGTTTATAATAATAGTT

TCCAATCTAGTATTCAAATGGCTCCATATGAAGCACTTTATGGTCGAAGGTGTCGATCAC

CAATATGTTGGACAAAATTAAGAGAAAGAAAAGTGATTGGGCCGGAATTGATTCAAGAGA

CAGAAGAAACAGTTAAAAAGATTAAAGATAGACTGAAAGCCGCTTTCGACAGACAGAAAT

CTTACGCAGACTTGAAACGACGAGACATTGAATATTCCGTTGGTGATAAGGTATTCCTCA

AAGTATCGCCGTGGAAGAAAATTTTGAGATTTGGTCGGAAGGGAAAATTAAGTCCGCGCT

TTATTGGGCCGTATGAGATAGTGGAAAGAATTGGGCCTGTTGCTTATCGATTATCCTTAC

CTCCAGAGTTACAGAAAATTCATGATGTTTTTCATGTTTCGATGCTTCGGAGATATAGAT

CGGATCCTTCTCATGTTATTCCCACTGAAGACATTGAACTTCGATCTGATTTAACTTATG

AAGAAGAACCAGTTCAAATATTAGCACGAGAAGTGAAAGAATTAAGAAATAAACGGGTTC

CTTTAGTACAAGTTTTATGGAGAAGCCATAGTGTGGAAGAAGCAACTTGGGAACCGGAAG

AGACAATGAGAGCACAATATCCTCATCTCTTCTCAGGTAAATTTCGAGGACGAAATTTAT

TAAGAGGGGGAGAAATGTAATGACCTAAAATTCATGGGCATCGGAAAAGTATAATATTGG

GCCTCCGTCCTAGTAAATTGAGTCCGAAAATAATTATTAGAAATATTTACGAGACTAGTA

GTGTGTTTAATTAGGTTTTAATTAAGTAAATTTAGCTTAATTTAGAGTAATTAGTAAAAA

GGATTAAATTGAATAAGAGTAAAAGTTTAATTATAGATTAAAGGAAAATAATAGGGACCA

AATGGGCAATTAAGCCACATTTGGAAGTTGAGGCGGCATAACATTGTAAAAATCTTAGAT

TTTTATATTATTATTTATATAAATATATAAATTAATTATAAAGTATATTATTAAATTAAT

TATATTATAAATATTATATTATTATATATAAAAGAAACAAAACAGAAAAGAAACAGAATA

GAAAGAACAAAGAAACAGAATAGAAGAGACGAAACAGGGGAGAAGCAGGGGAGAAAGAAG

AAAAAGAAGAAAAAAGGGGAAATAGGGTTTTTGAAGCTTGAAATTTAAATTGGTAAGTCA

AATTAGCCATTTTCTCTTAATTCTAATGTTTTAAAAGCTTTAAAACAAAGTTTTGATGGA

ATTAAGTTGATATTTTGTAAGTTCATAGGTTTTCAAGTATAGTTTATGTTGAACAAAAGA

GATGAATTAGGGATTAACTTGAAGGAATTTTAAGTTAGAATTGAAAAAGGGATTAAATTG

TAAAAGAAACTATAAGTTTTTTTTGTTTTAGGGACTAGATTGAGGAAAATTCGGAATTAA

GAAAATATGTTAAAAATTTAATAGTTAAATTTGAGTTTAAATGAAATTTGAATAGGAATA

AGGTGTGAATTGGTGTTATAAATTTGGTTATTAACATTTTTAATCAAAACAGTTTTGGGA

AGTAGCAATGGTCTGACTTTGAAAATTCACTAAAAATTTTATAAATTGAACTAGAGGATG

AACAAAATATGGAATTAAAGCTTATTGAGTCTAGTTTCTTATAGTAGAAACAATGTAAGC

AATTAATTGATGAATCAAGAGATATTTGAAATTTTGTAATACTGGTTCGGGGTGATTTCG

AGATGCCCTGTTTTAACTTTGGAAAATCATTAAAAATTGTACAAAAATTATTATGGAGTG

TAATTTATATATGTAAACTCCTTAATGAATCTAGTTTCAAAATAAATAAACAAGAACCTT

ATTCGAGTTCTGTACAATGAGATAATTTAGTTTTAGTGGAGAGAGGTCAGAACTGTCAAA

TGAAATAACAGGGGAGTATTTAACGAATAAACTGTATTAAATGGCTAGACCAAAAATTCT

GGAAATTTTATGATTAGAAGATATATGAGTCTAGTTTTAAGGAAAATTTACGGATATTAA

TTTGGAGTTTCGTAGCTCAAGATATAAATAATTTAGTAACAATGACCCAAGTAGACAGCT

TAATGGTGAAATTATATAAATACATTAAAAATGGTTAAATTTGCATGTTTAGGCTCATGA

ATTAAATTGAATCATGTTGTATTGATTATTATAAATTATTATTTTCGTAGCCAACAAAGA

ACCTAAAGCATCAGCATCGAAAGGAAAGGAGAAAGTCATCGAGGAGTAAACTCGAGAAAA

TTACGGTTTGTATTACTATAATTCAAGTTATTTATTATTAAATGTTAAATTTTAATTTAT

GTGTCTAGTAAATGAAATGTGAGGTAAGTATTATTATTATTATTATTATTATTATTATTA

TTATTATTATTATTATTATGAGTGGGAATTAAATTGAATAGTTGATATGAAATAATATTT

GAATTGTTTGTTGATTGAAAGCGGGAAATGAATTTAAATCGAATAGTGACCGATATTAAA

TTGAATGGAAATGTATTGAGTTGTGAAAATATGTTAATTGCGGATTAATTATTGATTGAA

AGGTGGAAAAATGATTGAATTGAAAGTGTGAGAAAGTGTGATTGAATTGGGATTATATGT

GATTTAAATACCCTATTAACTAGTCGGGCTGAGTCGGATATAGTTGGCATGCCATAGGAT

TGGAAGAGTTCAGGGATACTTCGACCTCGAGTCGATGAGACACTGGGTGTCACTATATTT

CTTCGGATAGATTCGATGAGGTACTGGGTACCAACTTTCTTCGGCTTTGCCGATGAGACA

CTGGGTGTCAACTATTGCTTCGAACTATCCGATGAGGCACTGGGTGCCATTCTGGTGTGT

TTGGTTGGATCCGTGTATTCGCCAAAGTCCGAGTTTTGTTAATAGGGTAAATGATGAAAT

GATAAACCGAACGAGTTGGTCAAACGAGCTATTGAAATGATATGAAAAAGTTGAATTGTG

AATTGAAATGTGAAATGAGATTGAGAAATGAACCTAAGGTTCGTGAATTATTCAAACTCA

AATTGTGGATATACGATATTGGTTGATGAATTGCTATTGTTGAAATATTTAATTTAAATT

GTATATACGATTTATGCTTTACATGTACATTATTGTTATAATTTGAATTATGGTAATACC

ACTGAGTATGAATTACTCAGCGTACGGTTGTTTCCGTGCGCAGGTCAATAGAAGTCAAAG

GTCTCGGTTCAGCATCCAGATTAATCCCGGCTTCGGCAAAACTTGGTGATGTATTTTTCC

TTTGGTAAAGGTGGCATGTACATAGATTGTGTATAAAGGTTATTATGTTTTATTATATAA

TGGTTAAAAATGTTAGTATTAAAAGTTTATGGATTTTAATGAAAGAAGTCTATCTATTTT

ATCTAATTAGTACATTGTTAAATTTTAAATTGGTATTAGATTGAGTTTGATTAGAAGTAT

TTAGAATAGAAAATGTGAATGTGAAATGAATTGGTTGAATTGATGATATTTGGGAACTAT

ATGGTTTTAATTTGC

>Deltapine5690

ATGTAATGACCCAAAATTCATGGGCATCGGAAAAGTATAATATCGGGCCTCCGTCCTAGT

AAATTGAGTCCGAAAATAATTATTAGAAATATTTACGAGACTAGTAGTGTGTTTAATTAG

GTTTTAATTAAGTAAATTTAGCTTAATTTAGAGTAATTAGTAAAAAGGATTAAATTGAAT

AAGAGTAAAAGTTTAATTATAGATTAAAGGAAAATAATAGGGACCAAATGGGCAATTAAG

CCACATTTGGAAGTTGAGGCGGCATAACATTGTAAAAATCTTAGATTTTTATATTATTAT

TTATATAAATATATAAATTAATTATAAAGTATATTATTAAATTAATTATATTATAAATAT

TATATTATTATATATAAAAGAAACAAAACAGAAAAGAAACAGAATAGAAAGAACAAAGAA

ACAGAATAGAAGAGACGAAACAGGGGAGAAGCAGGGGAGAAAGAAGAAAAAGAAGAAAAA

AGGGGAAATAGGGTTTTTGAAGCTTGAAATTTAAATTGGTAAGTCAAATTAGCCATTTTC

TCTTAATTCTAATGTTTTAAAAGCTTTAAAACAAAGTTTTGATGGAATTAAGTTGATATT

TTGTAAGTTCATAGGTTTTCAAGTATAGTTTATGTTGAACAAAAGAGATGAATTAGGGAT

TAACTTGAAGGAATTTTAAGTTAGAATTGAAAAAGGGATTAAATTGTAAAAGAAACTATA

AGTTTTTTTTGTTTTAGGGACTAGATTGAGGAAAATTCGGAATTAAGAAAATATGTTAAA

AATTTAATAGTTAAATTTGAGTTTAAATGAAATTTGAATAGGAATAAGGTGTGAATTGGT

GTTATAAATTTGGTTATTAACATTTTTAATCAAAACAGTTTTGGGAAGTAGCAATGGTCT

GACTTTGAAAATTCACTAAAAATTTTATAAATTGAACTAGAGGATGAACAAAATATGGAA

TTAAAGCTTATTGAGTCTAGTTTCTTATAGTAGAAACAATGTAAGCAATTAATTGATGAA

TCAAGAGATATTTGAAATTTTGTAATACTGGTTCGGGGTGATTTCGAGATGCCCTGTTTT

AACTTTGGAAAATCATTAAAAATTGTACAAAAATTATTATGGAGTGTAATTTATATATGT

GAACTCCTTAATGAATCTAGTTTCAAAATAAATAAACAAGAACCTTATTCGAGTTCTGTA

CAATGAGATAATTTAGTTTTAGTGGAGAGAGGTCAGAACTGTCAAATGAAATAACAGGGG

AGTATTTAACGAATAAACTGTATTAAATGGCTAGACCAAAAATTCTGGAAATTTTATGAT

TAGAAGATATATGAGTCTAGTTTTAAGGAAAATTTACGGATATTAATTTGGAGTTTCGTA

GCTCAAGATATAAATAATTTAGTAACAATGACCCAAGTAGACAGCTTAATGGTGAAATTA

TATAAATACATTAAAAATGGTTAAATTTGCATGTTTAGGCTCATGAATTAAATTGAATCA

TGTTGTATTGATTATTATAAATTATTATTTTCGTAGCCAACAAAGAACCTAAAGCATCAG

CATCGAAAGGAAAGGAGAAAGTCATCGAGGAGTAAACTCGAGAAAATTACGGTTTGTATT

ACTATAATTCAAGTTATTTATTATTAAATGTTAAATTTTAATTTATGTGTCTAGTAAATG

AAATGTGAGGTAAGTATTATTATTATTATTATTATTATTATTATTATTATTATTATTATT

ATGAGTGGGAATTAAATTGAATAGTTGATATGAAATAATATTTGAATTGTTTGTTGATTG

AAAGCGGGAAATGAATTTAAATCGAATAGTGACCGATATTAAATTGAATGGAAATGTATT

GAGTTGTGAAAATATGTTAATTGCGGATTAATTATTGATTGAAAGGTGGAAAAATGATTG

AATTGAAAGTGTGAGAAAGTGTGATTGAATTGGGATTATATGTGATTTAAATACCCTATT

AACTAGTCGGGCTGAGTCGGATATAGTTGGCATGCCATAGGATTGGAAGAGTTCAGGGAT

ACTTCGACCTCGAGTCGATGAGACACTGGGTGATTTCTTCGGATAGATTGGATGAGGTAC

TGGGTACCAACTTTCTTCGGCTTTGCCGATGAGACACTGGGTGTCAACTATTGCTTCGAA

CTATCCGATGAGGCACTGGGTGCCATTCTGGTGTGTTTGGTTGGATCCGTGTATCCGCCA

AAGTCCGAGTTTTGTTAATAGGGTAAATGATGAAATGATAAACCGAACGAGTTGGTCAAA

CGAGCTATTGAAATGATATGAAAAAGTTGAATTGTGAATTGAAATGTGAAATGAGATTGA

GAAATGAACCTAAGGTTCGTGAATTATTCAAACTCAAATTGTGGATATACGATATTGGTT

GATGAATTGCTATTGTTGAAATATTTAATTTAAATTGTATATACGATTTATGCTTTACAT

GTACATTATTGTTATAATTTGAATTATGGTAATACCACTGAGTATGAATTACTCAGCGTA

CGGTTGTTTCCGTGCGCAGGTCAATAGAAGTCAAAGGTCTCGGTTCAGCATCCAGATTAA

TCCCGGCTTCGGCAAAACTTGGTGATGTATTTTTCCTTTGGTAAAGGTGGCATGTACATA

GATTGTGTATAAAGGTTATTATGTTTTATTATATAATGGTTAAAAATGTTAGTATTAAAA

GTTTATGGATTTTAATGAAAGAAGTCTATCTATTTTATCTAATTAGTACATTGTTAAATT

TTAAATTGGTATTGTGTAGATTGAGTTTGATTAGAAGTATTTAGAATAGAAAATGTGAAT

GTGAAATGAATTGGTTGAATTGATGATATTTGGGAACTATATGGTTTTAATTTGCAGGGG

GTTTTATGTAAAAATAAGCAGAAATGCTGCCGAAATTTTTATAAAAAAAAATGAAGTCAT

TTGGTAAACAAATTAATAAATTTTATGAATTATTTTAATATATTGGTTATTTATTTAAGA

ATTGTTGTAAATCGTTCGATACGTCCGGTAGTGCCTCGTAATTCTGTTCCGGCGACGGTT

CGGGGTTAAGGGGTGTTACATTTTATGGTATCAGAGCTATCAGGTTTAGCCGATTCTCGG

CCTAAATCGAGCTCGGAATTGAGTCTAGATGTACATGCCACTGTCGAGTTAAACTGAGTC

GGGATTTTTGGATGCTGACCTATTTGTTTGTTTTGTTTTATAGATTAAAGATGTCTGAAG

AAAGAATAAATGATACTGATGAAAGAATGTATAGTGAAGATAGAGAATTAGATGAAACAG

AATCTGTTGCACCGAGTGTGAATCCGTTAGGCAACCAACCTTCTAATGTAGAACGAGAAA

ATGTCAGAGATAGAGATGAATCCCAATTACTGAGAATTATAGCTGATGCATTACAAAGAG

TAGCAGGAACTACTCCTGTTACGACTTCAGTACCTACTGTTAGACGGGCTCCGATAAAGG

AACTGAGGAAATATGGTGCCACTGAATTTATGGGTCTAAAAGGAGTTGATCCATCCATAG

CTGAAAATTGGATGGAGTCGACTAAAAGAATTTTGCAGCAATTGGATTGTACCCCCCGAG

AGTGTTTAATCTGTGCCGTATCGTTATTACAAGGGGAGGCTTATCTATGGTGGGAATCAG

TGGTTCGACATTTACCAGAGAGTCAGATAACGTGGGATCTATTTCAGAAGGAGTTTCAAA

AGAAATATATCGGAGAGATGTATATTGAAGACAAGAAACAAGAGTTTTTGTTGCTACAAC

AGGGTGATATGTCAGTAATAGATTATGAGAGGGAATTCTCGAGACTCAGTAGATATGCCT

CCGAGTTTATTCCGACAGAAGCCGATAGTTGTAAAAGATTTTTACGGGGTTTACGAGACG

AGATCAAAGTGCAGCTAGTATCCCATCGGATCACTGAGTTAGTAGATTTGATTGAACGAG

CTAAAATGGTGGAACAAGTTCTGGGCCTCGACAAAAAGACTGAAGTTGTTAGACCAACCG

GGAAGCGTACAGGAACTACCAGTTCGAATCCTCAGCCGAAAAGACCAAAGGAATTCCAAA

GTGGTTGGAGATCCAGTTTCAGGTCAGACAGAGGTGGTAGAAATAGGGGAAAACAGACGA

TGACATCTACTGGCAGTGTGAAAGGTCCTTCCCGAGAAATAGATATTCCAGACTGCCAAC

ACTGCGGAAAGAAACACAGAGGGGAATGTTGGAAATTAACTAGAGGCTGTTTTCGATGTG

GTTCTACAGACCATTTCATCAGAGACTGTCCGAAAGTTGATAGTACTGTACCCGTGACAT

CACAGAGATCGGTATCTACAGCTAGAGGCAGAGGGTTAGGAAGAGGTGGTTCGGTTTCAA

GGGGAGGAAGTATTAGGAGAAGCAATGATATTGCTACTCAGCAGTCTGAGGCTAAAGTAC

CTGCCAGAGCTTATGTGGTCAGAACACGGGAAGAAGGTGACGCCCACGATGTAGTAACAG

GTATATTCTTACTATATTCTGAGCCTGTTTATGCTTTAATTGATCCCGGATCTTCACATT

CTTATATAAATTCAAAATTAGTTGAATTGGGAAAATTTAATTCTGAAATATCTAGAGTGA

CTGTAGAAGTGTCGAGTCCGTTGGGGCAAACAGTATTAGTGAATCAGATCTGTCCGAGAT

GCCCGTTAATTATACAAAATAAAACTTTTCCTATTGACCTGTTGATTATGCCATTTGGAG

ATTTTGATATAATACTGGGGATGGATTGGTTGGCTGAGCACGGAGTGGTATTGGATTGTT

ATAAAAAGAAGTTTAGTATTCAGACAGAAGACGGGGACAGAATTGAAGTAAATGGTATCC

GTACTAATGGGCCGACACGTATTATTTCGGCAATAAAGGCTAATAAATTGCTTCAGCGGG

GTTGTACAGCGTATTTAGCCTATGTTATTAATTCTGATTTGGTTGGTAGTCAGTGCAGTA

AGATTAGAACCGTATGTGAGTTTCCAGATGTATTTCCTGAAGAGCTACCGGGTTTACCAC

CTGACAGAGAGGTTGAATTTGCTATAGAAGTGTATCCGGGTACAGCACCAATCTCTATAC

CACCGTATCGAATGTCACCCACTGAGTTGAAAGAGTTGAAAGTGCAGTTACAGGACTTGT

CAGATCGTGGATTTATTAGACCGAGCATCTCACCTTGGGGAGCTCCAGTATTGTTTGTTA

AAAAGAAAGATGGATCGATGCGGCTTTGTATTGATTACCGGCAGTTAAACAAAGTGACGA

TCAAGAACCGGTATCCGTTACCCCGTATAGATGATTTATTTGATCAACTAAAAGGAGCTT

CAGTATTTTCAAAGATTGACTTAAGATCTGGGTATTATCAGCTGAAGGTAAAAGAAAGTG

ATGTTCCGAAGACTGCATTTCGTACTCGATATGGTCATTATGAATTTTTGGTGATGCCGT

TCGGGTTGACTAATGCTCCAGCTGCTTTTATGGATCTGATGAATCGTATTTTTCAGCCGT

ATTTAGATCAGTTTGTGGTGGTTTTTATTGATGACATCTTGGTTTATTCGAAGTCAGAGT

CAGAGCATGATCAGCATCTCAGAACCGTGCTACAAATTCTGCGAGAAAAACAGTTGTACG

GGAAACTAAGTAAATGTGAATTCTGGTTATCAGAGGTAGTATTCTTGGGACATGTTGTAT

CTGCGGATGGGATTAGAGTTGATCCGAAGAAGATCGAGGCAATTGTTCAATGGAAGGCAC

CAAAGAATGTATCAGAGGTACGCAGTTTTCTTGGTTTGGCTGGGTATTACAGAAGATTTG

TAAATGGGTTTTCGAAGATAGCTTTGCCGATGACCAAATTACTACAGAAGAATGTTCCAT

TTATCTGGGATGATCAGTGTCAGAGGAGCTTTGAAACATTGAAACAGATGTTGACAGAGG

CACCAGTTTTAACTTTACCAGAATCAGGGAAAGATTTCATAGTGTACAGTGATGCTTCTT

TGAATGGTTTGGGTTGTGTATTGATGCAAGAAGGAAAAGTAATAGCTTATGCATCTCGAC

AGTTGAAGTCACATGAACGCAACTACCCGACACACGATTTAGAGTTAGCTGCTGTAATCT

TTGCATTGAAGATTTGGATACATTACTTGTATGGTGAGAAATGTTATATTTACACTGATC

ATAAAAGTCTAAAATATCTTCTGTCACAAAAGGAGTTGAATCTGAGACAGAGACGGTGGA

TTGAACTTCTGAAAGATTATGATTGTGTTATAGATTATCATCCAGGGAAGGCAAATGTGG

TAGCAGATGCATTGAGTAGAAAAGCAGCGATTGAATTACGAGCAATGTTCGCTCGACTTA

GTATTAAGGATGATGGAAGTTTGTTAGCTGAGTTAAGAGTCAAGCCGGTGATGTTTGATC

AAATCAGAGCAGCACAGTTAAAAGATGAAAAGTTGATGAGGAAAAGAGAAATGGTACAGT

ATGGTGCGGTAGAAAATTTTAGTATTGACGAGCATGATTGTTTGAGATTTCGAAATCGAA

TTTGTGTTCCATCTACTTCTGAGATTAAAGAATTGATTCTCCGAGAAGCACATAATAGTA

TTTTTGCTTTGCACCCAGGAGGAACGAAGATGTATCGTGATCTACGAGAACTGTATTGGT

GGCCAGGAATGAAGAAAGATATAGTTGAATATGTCAGTAAATGCTTGACTTGTCAGCGGG

TAAAAGCAGAACATCAGGTACCAACAGGCCTGTTACAGCCTATTACTATTCCCGAGTGGA

AATGGGATCGCATTACCATGGATTTTGTTACGGGGTTGCCATTGTCAGTGAGTAAAAAGA

ATGCTATTTGGGTGATTGTTGATCGACTCACAAAATCAGCTCATTTTATAGCAGTTAGAA

CCGACTGGTCATTACAGAAGCTTGCCGAGGTTTATATTCGAGAAATTGTTAGATTACATG

GTATTCCGGTATCAATAATTTCAGACAGAGATCCTCGATTCACTTCGAGATTTTGGAAGC

AGCTGCATGAATCATTGGGTACTCGACTTAGTTTCAGTACAGCTTTTCATCCTCAAACTG

ATGGACAATCTGAACGAGTAATTCAGATATTAGAAGATATGCTTCGAGCTTGTGTCATTG

ATTTTGAATCAGGTTGGGAACGTTATTTACCATTGGCCGAGTTTGTTTATAATAATAGTT

TCCAATCTAGTATTCAAATGGCTCCATATGAAGCACTTTATGGTCGAAGGTGTCGATCAC

CAATATGTTGGACAAAATTAAGAGAAAGAAAAGTGATTGGGCCGGAATTGATTCAAGAGA

CAGAAGAAACAGTTAAAAAGATTAAAGATAGACTGAAAGCCGCTTTCGACAGACAGAAAT

CTTACGCAGACTTGAAACGACGAGACATTGAATATTCCGTTGGTGATAAGGTATTCCTCA

AAGTATCGCCGTGGAAGAAAATTTTGAGATTTGGTCGGAAGGGAAAATTAAGTCCGCGCT

TTATTGGGCCGTATGAGATAGTGGAAAGAATTGGGCCTGTTGCTTATCGATTATCCTTAC

CTCCAGAGTTACAGAAAATTCATGATGTTTTTCATGTTTCGATGCTTCGGAGATATAGAT

CGGATCCTTCTCATGTTATTCCCACTGAAGACATTGAACTTCGATCTGATTTAACTTATG

AAGAAGAACCAGTTCAAATATTAGCACGAGAAGTGAAAGAATTAAGAAATAAACGGGTTC

CTTTAGTACAAGTTTTATGGAGAAGCCATAGTGTGGAAGAAGCAACTTGGGAACCGGAAG

AGACAATGAGAGCACAATATCCTCATCTCTTCTCAGGTAAATTTCGAGGACGAAATTTAT

TAAGAGGGGGAGAAATGTAATGACCTAAAATTCATGGGCATCGGAAAAGTATAATATTGG

GCCTCCGTCCTAGTAAATTGAGTCCGAAAATAATTATTAGAAATATTTACGAGACTAGTA

GTGTGTTTAATTAGGTTTTAATTAAGTAAATTTAGCTTAATTTAGAGTAATTAGTAAAAA

GGATTAAATTGAATAAGAGTAAAAGTTTAATTATAGATTAAAGGAAAATAATAGGGACCA

AATGGGCAATTAAGCCACATTTGGAAGTTGAGGCGGCATAACATTGTAAAAATCTTAGAT

TTTTATATTATTATTTATATAAATATATAAATTAATTATAAAGTATATTATTAAATTAAT

TATATTATAAATATTATATTATTATATATAAAAGAAACAAAACAGAAAAGAAACAGAATA

GAAAGAACAAAGAAACAGAATAGAAGAGACGAAACAGGGGAGAAGCAGGGGAGAAAGAAG

AAAAAGAAGAAAAAAGGGGAAATAGGGTTTTTGAAGCTTGAAATTTAAATTGGTAAGTCA

AATTAGCCATTTTCTCTTAATTCTAATGTTTTAAAAGCTTTAAAACAAAGTTTTGATGGA

ATTAAGTTGATATTTTGTAAGTTCATAGGTTTTCAAGTATAGTTTATGTTGAACAAAAGA

GATGAATTAGGGATTAACTTGAAGGAATTTTAAGTTAGAATTGAAAAAGGGATTAAATTG

TAAAAGAAACTATAAGTTTTTTTTGTTTTAGGGACTAGATTGAGGAAAATTCGGAATTAA

GAAAATATGTTAAAAATTTAATAGTTAAATTTGAGTTTAAATGAAATTTGAATAGGAATA

AGGTGTGAATTGGTGTTATAAATTTGGTTATTAACATTTTTAATCAAAACAGTTTTGGGA

AGTAGCAATGGTCTGACTTTGAAAATTCACTAAAAATTTTATAAATTGAACTAGAGGATG

AACAAAATATGGAATTAAAGCTTATTGAGTCTAGTTTCTTATAGTAGAAACAATGTAAGC

AATTAATTGATGAATCAAGAGATATTTGAAATTTTGTAATACTGGTTCGGGGTGATTTCG

AGATGCCCTGTTTTAACTTTGGAAAATCATTAAAAATTGTACAAAAATTATTATGGAGTG

TAATTTATATATGTAAACTCCTTAATGAATCTAGTTTCAAAATAAATAAACAAGAACCTT

ATTCGAGTTCTGTACAATGAGATAATTTAGTTTTAGTGGAGAGAGGTCAGAACTGTCAAA

TGAAATAACAGGGGAGTATTTAACGAATAAACTGTATTAAATGGCTAGACCAAAAATTCT

GGAAATTTTATGATTAGAAGATATATGAGTCTAGTTTTAAGGAAAATTTACGGATATTAA

TTTGGAGTTTCGTAGCTCAAGATATAAATAATTTAGTAACAATGACCCAAGTAGACAGCT

TAATGGTGAAATTATATAAATACATTAAAAATGGTTAAATTTGCATGTTTAGGCTCATGA

ATTAAATTGAATCATGTTGTATTGATTATTATAAATTATTATTTTCGTAGCCAACAAAGA

ACCTAAAGCATCAGCATCGAAAGGAAAGGAGAAAGTCATCGAGGAGTAAACTCGAGAAAA

TTACGGTTTGTATTACTATAATTCAAGTTATTTATTATTAAATGTTAAATTTTAATTTAT

GTGTCTAGTAAATGAAATGTGAGGTAAGTATTATTATTATTATTATTATTATTATTATTA

TTATTATTATTATTATTATGAGTGGGAATTAAATTGAATAGTTGATATGAAATAATATTT

GAATTGTTTGTTGATTGAAAGCGGGAAATGAATTTAAATCGAATAGTGACCGATATTAAA

TTGAATGGAAATGTATTGAGTTGTGAAAATATGTTAATTGCGGATTAATTATTGATTGAA

AGGTGGAAAAATGATTGAATTGAAAGTGTGAGAAAGTGTGATTGAATTGGGATTATATGT

GATTTAAATACCCTATTAACTAGTCGGGCTGAGTCGGATATAGTTGGCATGCCATAGGAT

TGGAAGAGTTCAGGGATACTTCGACCTCGAGTCGATGAGACACTGGGTGTCACTATATTT

CTTCGGATAGATTCGATGAGGTACTGGGTACCAACTTTCTTCGGCTTTGCCGATGAGACA

CTGGGTGTCAACTATTGCTTCGAACTATCCGATGAGGCACTGGGTGCCATTCTGGTGTGT

TTGGTTGGATCCGTGTATTCGCCAAAGTCCGAGTTTTGTTAATAGGGTAAATGATGAAAT

GATAAACCGAACGAGTTGGTCAAACGAGCTATTGAAATGATATGAAAAAGTTGAATTGTG

AATTGAAATGTGAAATGAGATTGAGAAATGAACCTAAGGTTCGTGAATTATTCAAACTCA

AATTGTGGATATACGATATTGGTTGATGAATTGCTATTGTTGAAATATTTAATTTAAATT

GTATATACGATTTATGCTTTACATGTACATTATTGTTATAATTTGAATTATGGTAATACC

ACTGAGTATGAATTACTCAGCGTACGGTTGTTTCCGTGCGCAGGTCAATAGAAGTCAAAG

GTCTCGGTTCAGCATCCAGATTAATCCCGGCTTCGGCAAAACTTGGTGATGTATTTTTCC

TTTGGTAAAGGTGGCATGTACATAGATTGTGTATAAAGGTTATTATGTTTTATTATATAA

TGGTTAAAAATGTTAGTATTAAAAGTTTATGGATTTTAATGAAAGAAGTCTATCTATTTT

ATCTAATTAGTACATTGTTAAATTTTAAATTGGTATTAGATTGAGTTTGATTAGAAGTAT

TTAGAATAGAAAATGTGAATGTGAAATGAATTGGTTGAATTGATGATATTTGGGAACTAT

ATGGTTTTAATTTGC

>Deltapine5816

ATGTAATGACCCAAAATTCATGGGCATCGGAAAAGTATAATATCGGGCCTCCGTCCTAGT

AAATTGAGTCCGAAAATAATTATTAGAAATATTTACGAGACTAGTAGTGTGTTTAATTAG

GTTTTAATTAAGTAAATTTAGCTTAATTTAGAGTAATTAGTAAAAAGGATTAAATTGAAT

AAGAGTAAAAGTTTAATTATAGATTAAAGGAAAATAATAGGGACCAAATGGGCAATTAAG

CCACATTTGGAAGTTGAGGCGGCATAACATTGTAAAAATCTTAGATTTTTATATTATTAT

TTATATAAATATATAAATTAATTATAAAGTATATTATTAAATTAATTATATTATAAATAT

TATATTATTATATATAAAAGAAACAAAACAGAAAAGAAACAGAATAGAAAGAACAAAGAA

ACAGAATAGAAGAGACGAAACAGGGGAGAAGCAGGGGAGAAAGAAGAAAAAGAAGAAAAA

AGGGGAAATAGGGTTTTTGAAGCTTGAAATTTAAATTGGTAAGTCAAATTAGCCATTTTC

TCTTAATTCTAATGTTTTAAAAGCTTTAAAACAAAGTTTTGATGGAATTAAGTTGATATT

TTGTAAGTTCATAGGTTTTCAAGTATAGTTTATGTTGAACAAAAGAGATGAATTAGGGAT

TAACTTGAAGGAATTTTAAGTTAGAATTGAAAAAGGGATTAAATTGTAAAAGAAACTATA

AGTTTTTTTTGTTTTAGGGACTAGATTGAGGAAAATTCGGAATTAAGAAAATATGTTAAA

AATTTAATAGTTAAATTTGAGTTTAAATGAAATTTGAATAGGAATAAGGTGTGAATTGGT

GTTATAAATTTGGTTATTAACATTTTTAATCAAAACAGTTTTGGGAAGTAGCAATGGTCT

GACTTTGAAAATTCACTAAAAATTTTATAAATTGAACTAGAGGATGAACAAAATATGGAA

TTAAAGCTTATTGAGTCTAGTTTCTTATAGTAGAAACAATGTAAGCAATTAATTGATGAA

TCAAGAGATATTTGAAATTTTGTAATACTGGTTCGGGGTGATTTCGAGATGCCCTGTTTT

AACTTTGGAAAATCATTAAAAATTGTACAAAAATTATTATGGAGTGTAATTTATATATGT

GAACTCCTTAATGAATCTAGTTTCAAAATAAATAAACAAGAACCTTATTCGAGTTCTGTA

CAATGAGATAATTTAGTTTTAGTGGAGAGAGGTCAGAACTGTCAAATGAAATAACAGGGG

AGTATTTAACGAATAAACTGTATTAAATGGCTAGACCAAAAATTCTGGAAATTTTATGAT

TAGAAGATATATGAGTCTAGTTTTAAGGAAAATTTACGGATATTAATTTGGAGTTTCGTA

GCTCAAGATATAAATAATTTAGTAACAATGACCCAAGTAGACAGCTTAATGGTGAAATTA

TATAAATACATTAAAAATGGTTAAATTTGCATGTTTAGGCTCATGAATTAAATTGAATCA

TGTTGTATTGATTATTATAAATTATTATTTTCGTAGCCAACAAAGAACCTAAAGCATCAG

CATCGAAAGGAAAGGAGAAAGTCATCGAGGAGTAAACTCGAGAAAATTACGGTTTGTATT

ACTATAATTCAAGTTATTTATTATTAAATGTTAAATTTTAATTTATGTGTCTAGTAAATG

AAATGTGAGGTAAGTATTATTATTATTATTATTATTATTATTATTATTATTATTATTATT

ATGAGTGGGAATTAAATTGAATAGTTGATATGAAATAATATTTGAATTGTTTGTTGATTG

AAAGCGGGAAATGAATTTAAATCGAATAGTGACCGATATTAAATTGAATGGAAATGTATT

GAGTTGTGAAAATATGTTAATTGCGGATTAATTATTGATTGAAAGGTGGAAAAATGATTG

AATTGAAAGTGTGAGAAAGTGTGATTGAATTGGGATTATATGTGATTTAAATACCCTATT

AACTAGTCGGGCTGAGTCGGATATAGTTGGCATGCCATAGGATTGGAAGAGTTCAGGGAT

ACTTCGACCTCGAGTCGATGAGACACTGGGTGATTTCTTCGGATAGATTGGATGAGGTAC

TGGGTACCAACTTTCTTCGGCTTTGCCGATGAGACACTGGGTGTCAACTATTGCTTCGAA

CTATCCGATGAGGCACTGGGTGCCATTCTGGTGTGTTTGGTTGGATCCGTGTATCCGCCA

AAGTCCGAGTTTTGTTAATAGGGTAAATGATGAAATGATAAACCGAACGAGTTGGTCAAA

CGAGCTATTGAAATGATATGAAAAAGTTGAATTGTGAATTGAAATGTGAAATGAGATTGA

GAAATGAACCTAAGGTTCGTGAATTATTCAAACTCAAATTGTGGATATACGATATTGGTT

GATGAATTGCTATTGTTGAAATATTTAATTTAAATTGTATATACGATTTATGCTTTACAT

GTACATTATTGTTATAATTTGAATTATGGTAATACCACTGAGTATGAATTACTCAGCGTA

CGGTTGTTTCCGTGCGCAGGTCAATAGAAGTCAAAGGTCTCGGTTCAGCATCCAGATTAA

TCCCGGCTTCGGCAAAACTTGGTGATGTATTTTTCCTTTGGTAAAGGTGGCATGTACATA

GATTGTGTATAAAGGTTATTATGTTTTATTATATAATGGTTAAAAATGTTAGTATTAAAA

GTTTATGGATTTTAATGAAAGAAGTCTATCTATTTTATCTAATTAGTACATTGTTAAATT

TTAAATTGGTATTGTGTAGATTGAGTTTGATTAGAAGTATTTAGAATAGAAAATGTGAAT

GTGAAATGAATTGGTTGAATTGATGATATTTGGGAACTATATGGTTTTAATTTGCAGGGG

GTTTTATGTAAAAATAAGCAGAAATGCTGCCGAAATTTTTATAAAAAAAAATGAAGTCAT

TTGGTAAACAAATTAATAAATTTTATGAATTATTTTAATATATTGGTTATTTATTTAAGA

ATTGTTGTAAATCGTTCGATACGTCCGGTAGTGCCTCGTAATTCTGTTCCGGCGACGGTT

CGGGGTTAAGGGGTGTTACATTTTATGGTATCAGAGCTATCAGGTTTAGCCGATTCTCGG

CCTAAATCGAGCTCGGAATTGAGTCTAGATGTACATGCCACTGTCGAGTTAAACTGAGTC

GGGATTTTTGGATGCTGACCTATTTGTTTGTTTTGTTTTATAGATTAAAGATGTCTGAAG

AAAGAATAAATGATACTGATGAAAGAATGTATAGTGAAGATAGAGAATTAGATGAAACAG

AATCTGTTGCACCGAGTGTGAATCCGTTAGGCAACCAACCTTCTAATGTAGAACGAGAAA

ATGTCAGAGATAGAGATGAATCCCAATTACTGAGAATTATAGCTGATGCATTACAAAGAG

TAGCAGGAACTACTCCTGTTACGACTTCAGTACCTACTGTTAGACGGGCTCCGATAAAGG

AACTGAGGAAATATGGTGCCACTGAATTTATGGGTCTAAAAGGAGTTGATCCATCCATAG

CTGAAAATTGGATGGAGTCGACTAAAAGAATTTTGCAGCAATTGGATTGTACCCCCCGAG

AGTGTTTAATCTGTGCCGTATCGTTATTACAAGGGGAGGCTTATCTATGGTGGGAATCAG

TGGTTCGACATTTACCAGAGAGTCAGATAACGTGGGATCTATTTCAGAAGGAGTTTCAAA

AGAAATATATCGGAGAGATGTATATTGAAGACAAGAAACAAGAGTTTTTGTTGCTACAAC

AGGGTGATATGTTAGTAATAGATTATGAGAGGGAATTCTCGAGACTCAGTAGATATGCCT

CCGAGTTTATTCCGACAGAAGCCGATAGTTGTAAAAGATTTTTACGGGGTTTACGAGACG

AGATCAAAGTGCAGCTAGTATCCCATCGGATCACTGAGTTAGTAGATTTGATTGAACGAG

CTAAAATGGTGGAACAAGTTCTGGGCCTCGACAAAAAGACTGAAGTTGTTAGACCAACCG

GGAAGCGTACAGGAACTACCAGTTCGAATCCTCAGCCGAAAAGACCAAAGGAATTCCAAA

GTGGTTGGAGATCCAGTTTCAGGTCAGACAGAGGTGGTAGAAATAGGGGAAAACAGACGA

TGACATCTACTGGCAGTGTGAAAGGTCCTTCCCGAGAAATAGATATTCCAGACTGCCAAC

ACTGCGGAAAGAAACACAGAGGGGAATGTTGGAAATTAACTAGAGGCTGTTTTCGATGTG

GTTCTACAGACCATTTCATCAGAGACTGTCCGAAAGTTGATAGTACTGTACCCGTGACAT

CACAGAGATCGGTATCTACAGCTAGAGGCAGAGGGTTAGGAAGAGGTGGTTCGGTTTCAA

GGGGAGGAAGTATTAGGAGAAGCAATGATATTGCTACTCAGCAGTCTGAGGCTAAAGTAC

CTGCCAGAGCTTATGTGGTCAGAACACAGGAAGAAGGTGACGCCCACGATGTAGTAACAG

GTATATTCTTACTATATTCTGAGCCTGTTTATGCTTTAATTGATCCCGGATCTTCACATT

CTTATATAAATTCAAAATTAGTTGAATTGGGAAAATTTAATTCTGAAATATCTAGAGTGA

CTGTAGAAGTGTCGAGTCCGTTGGGGCAAACAGTATTAGTGAATCAGATCTGTCCGAGAT

GCCCGTTAATTATACAAAATAAAACTTTTCCTATTGACCTGTTGATTATGCCATTTGGAG

ATTTTGATATAATACTGGGGATGGATTGGTTGGCTGAGCACGGAGTGGTATTGGATTGTT

ATAAAAAGAAGTTTAGTATTCAGACAGAAGACGGGGACAGAATTGAAGTAAATGGTATCC

GTACTAATGGGCCGACACGTATTATTTCGGCAATAAAGGCTAATAAATTGCTTCAGCGGG

GTTGTACAGCGTATTTAGCCTATGTTATTAATTCTGATTTGGTTGGTAGTCAGTGCAGTA

AGATTAGAACCGTATGTGAGTTTCCAGATGTATTTCCTGAAGAGCTACCGGGTTTACCAC

CTGACAGAGAGGTTGAATTTGCTATAGAAGTGTATCCGGGTACAGCACCAATCTCTATAC

CACCGTATCGAATGTCACCCACTGAGTTGAAAGAGTTGAAAGTGCAGTTACAGGACTTGT

CAGATCGTGGATTTATTAGACCGAGCATCTCACCTTGGGGAGCTCCAGTATTGTTTGTTA

AAAAGAAAGATGGATCGATGCGGCTTTGTATTGATTACCGGCAGTTAAACAAAGTGACGA

TCAAGAACCGGTATCCGTTACCCCGTATAGATGATTTATTTGATCAACTAAAAGGAGCTT

CAGTATTTTCAAAGATTGACTTAAGATCTGGGTATTATCAGCTGAAGGTAAAAGAAAGTG

ATGTTCCGAAGACTGCATTTCGTACTCGATATGGTCATTATGAATTTTTGGTGATGCCGT

TCGGGTTGACTAATGCTCCAGCTGCTTTTATGGATCTGATGAATCGTATTTTTCAGCCGT

ATTTAGATCAGTTTGTGGTGGTTTTTATTGATGACATCTTGGTTTATTCGAAGTCAGAGT

CAGAGCATGATCAGCATCTCAGAACCGTGCTACAAATTCTGCGAGAAAAACAGTTGTACG

GGAAACTAAGTAAATGTGAATTCTGGTTATCAGAGGTAGTATTCTTGGGACATGTTGTAT

CTGCGGATGGGATTAGAGTTGATCCGAAGAAGATCGAGGCAATTGTTCAATGGAAGGCAC

CAAAGAATGTATCAGAGGTACGCAGTTTTCTTGGTTTGGCTGGGTATTACAGAAGATTTG

TAAATGGGTTTTCGAAGATAGCTTTGCCGATGACCAAATTACTACAGAAGAATGTTCCAT

TTATCTGGGATGATCAGTGTCAGAGGAGCTTTGAAACATTGAAACAGATGTTGACAGAGG

CACCAGTTTTAACTTTACCAGAATCAGGGAAAGATTTCATAGTGTACAGTGATGCTTCTT

TGAATGGTTTGGGTTGTGTATTGATGCAAGAAGGAAAAGTAATAGCTTATGCATCTCGAC

AGTTGAAGTCACATGAACGCAACTACCCGACACACGATTTAGAGTTAGCTGCTGTAATCT

TTGCATTGAAGATTTGGATACATTACTTGTATGGTGAGAAATGTTATATTTACACTGATC

ATAAAAGTCTAAAATATCTTCTGTCACAAAAGGAGTTGAATCTGAGACAGAGACGGTGGA

TTGAACTTCTGAAAGATTATGATTGTGTTATAGATTATCATCCAGGGAAGGCAAATGTGG

TAGCAGATGCATTGAGTAGAAAAGCAGCGATTGAATTACGAGCAATGTTCGCTCGACTTA

GTATTAAGGATGATGGAAGTTTGTTAGCTGAGTTAAGAGTCAAGCCGGTGATGTTTGATC

AAATCAGAGCAGCACAGTTAAAAGATGAAAAGTTGATGAGGAAAAGAGAAATGGTACAGT

ATGGTGCGGTAGAAAATTTTAGTATTGACGAGCATGATTGTTTGAGATTTCGAAATCGAA

TTTGTGTTCCATCTACTTCTGAGATTAAAGAATTGATTCTCCGAGAAGCACATAATAGTA

TTTTTGCTTTGCACCCAGGAGGAACGAAGATGTATCGTGATCTACGAGAACTGTATTGGT

GGCCAGGAATGAAGAAAGATATAGTTGAATATGTCAGTAAATGCTTGACTTGTCAGCGGG

TAAAAGCAGAACATCAGGTACCAACAGGCCTGTTACAGCCTATTACTATTCCCGAGTGGA

AATGGGATCGCATTACCATGGATTTTGTTACGGGGTTGCCATTGTCAGTGAGTAAAAAGA

ATGCTATTTGGGTGATTGTTGATCGACTCACAAAATCAGCTCATTTTATAGCAGTTAGAA

CCGACTGGTCATTACAGAAGCTTGCCGAGGTTTATATTCGAGAAATTGTTAGATTACATG

GTATTCCGGTATCAATAATTTCAGACAGAGATCCTCGATTCACTTCGAGATTTTGGAAGC

AGCTGCATGAATCATTGGGTACTCGACTTAGTTTCAGTACAGCTTTTCATCCTCAAACTG

ATGGACAATCTGAACGAGTAATTCAGATATTAGAAGATATGCTTCGAGCTTGTGTCATTG

ATTTTGAATCAGGTTGGGAACGTTATTTACCATTGGCCGAGTTTGTTTATAATAATAGTT

TCCAATCTAGTATTCAAATGGCTCCATATGAAGCACTTTATGGTCGAAGGTGTCGATCAC

CAATATGTTGGACAAAATTAAGAGAAAGAAAAGTGATTGGGCCGGAATTGATTCAAGAGA

CAGAAGAAACAGTTAAAAAGATTAAAGATAGACTGAAAGCCGCTTTCGACAGACAGAAAT

CTTACGCAGACTTGAAACGACGAGACATTGAATATTCCGTTGGTGATAAGGTATTCCTCA

AAGTATCGCCGTGGAAGAAAATTTTGAGATTTGGTCGGAAGGGAAAATTAAGTCCGCGCT

TTATTGGGCCGTATGAGATAGTGGAAAGAATTGGGCCTGTTGCTTATCGATTATCCTTAC

CTCCAGAGTTACAGAAAATTCATGATGTTTTTCATGTTTCGATGCTTCGGAGATATAGAT

CGGATCCTTCTCATGTTATTCCCACTGAAGACATTGAACTTCGATCTGATTTAACTTATG

AAGAAGAACCAGTTCAAATATTAGCACGAGAAGTGAAAGAATTAAGAAATAAACGGGTTC

CTTTAGTACAAGTTTTATGGAGAAGCCATAGTGTGGAAGAAGCAACTTGGGAACCGGAAG

AGACAATGAGAGCACAATATCCTCATCTCTTCTCAGGTAAATTTCGAGGACGAAATTTAT

TAAGAGGGGGAGAAATGTAATGACCTAAAATTCATGGGCATCGGAAAAGTATAATATTGG

GCCTCCGTCCTAGTAAATTGAGTCCGAAAATAATTATTAGAAATATTTACGAGACTAGTA

GTGTGTTTAATTAGGTTTTAATTAAGTAAATTTAGCTTAATTTAGAGTAATTAGTAAAAA

GGATTAAATTGAATAAGAGTAAAAGTTTAATTATAGATTAAAGGAAAATAATAGGGACCA

AATGGGCAATTAAGCCACATTTGGAAGTTGAGGCGGCATAACATTGTAAAAATCTTAGAT

TTTTATATTATTATTTATATAAATATATAAATTAATTATAAAGTATATTATTAAATTAAT

TATATTATAAATATTATATTATTATATATAAAAGAAACAAAACAGAAAAGAAACAGAATA

GAAAGAACAAAGAAACAGAATAGAAGAGACGAAACAGGGGAGAAGCAGGGGAGAAAGAAG

AAAAAGAAGAAAAAAGGGGAAATAGGGTTTTTGAAGCTTGAAATTTAAATTGGTAAGTCA

AATTAGCCATTTTCTCTTAATTCTAATGTTTTAAAAGCTTTAAAACAAAGTTTTGATGGA

ATTAAGTTGATATTTTGTAAGTTCATAGGTTTTCAAGTATAGTTTATGTTGAACAAAAGA

GATGAATTAGGGATTAACTTGAAGGAATTTTAAGTTAGAATTGAAAAAGGGATTAAATTG

TAAAAGAAACTATAAGTTTTTTTTGTTTTAGGGACTAGATTGAGGAAAATTCGGAATTAA

GAAAATATGTTAAAAATTTAATAGTTAAATTTGAGTTTAAATGAAATTTGAATAGGAATA

AGGTGTGAATTGGTGTTATAAATTTGGTTATTAACATTTTTAATCAAAACAGTTTTGGGA

AGTAGCAATGGTCTGACTTTGAAAATTCACTAAAAATTTTATAAATTGAACTAGAGGATG

AACAAAATATGGAATTAAAGCTTATTGAGTCTAGTTTCTTATAGTAGAAACAATGTAAGC

AATTAATTGATGAATCAAGAGATATTTGAAATTTTGTAATACTGGTTCGGGGTGATTTCG

AGATGCCCTGTTTTAACTTTGGAAAATCATTAAAAATTGTACAAAAATTATTATGGAGTG

TAATTTATATATGTAAACTCCTTAATGAATCTAGTTTCAAAATAAATAAACAAGAACCTT

ATTCGAGTTCTGTACAATGAGATAATTTAGTTTTAGTGGAGAGAGGTCAGAACTGTCAAA

TGAAATAACAGGGGAGTATTTAACGAATAAACTGTATTAAATGGCTAGACCAAAAATTCT

GGAAATTTTATGATTAGAAGATATATGAGTCTAGTTTTAAGGAAAATTTACGGATATTAA

TTTGGAGTTTCGTAGCTCAAGATATAAATAATTTAGTAACAATGACCCAAGTAGACAGCT

TAATGGTGAAATTATATAAATACATTAAAAATGGTTAAATTTGCATGTTTAGGCTCATGA

ATTAAATTGAATCATGTTGTATTGATTATTATAAATTATTATTTTCGTAGCCAACAAAGA

ACCTAAAGCATCAGCATCGAAAGGAAAGGAGAAAGTCATCGAGGAGTAAACTCGAGAAAA

TTACGGTTTGTATTACTATAATTCAAGTTATTTATTATTAAATGTTAAATTTTAATTTAT

GTGTCTAGTAAATGAAATGTGAGGTAAGTATTATTATTATTATTATTATTATTATTATTA

TTATTATTATTATTATTATGAGTGGGAATTAAATTGAATAGTTGATATGAAATAATATTT

GAATTGTTTGTTGATTGAAAGCGGGAAATGAATTTAAATCGAATAGTGACCGATATTAAA

TTGAATGGAAATGTATTGAGTTGTGAAAATATGTTAATTGCGGATTAATTATTGATTGAA

AGGTGGAAAAATGATTGAATTGAAAGTGTGAGAAAGTGTGATTGAATTGGGATTATATGT

GATTTAAATACCCTATTAACTAGTCGGGCTGAGTCGGATATAGTTGGCATGCCATAGGAT

TGGAAGAGTTCAGGGATACTTCGACCTCGAGTCGATGAGACACTGGGTGTCACTATATTT

CTTCGGATAGATTCGATGAGGTACTGGGTACCAACTTTCTTCGGCTTTGCCGATGAGACA

CTGGGTGTCAACTATTGCTTCGAACTATCCGATGAGGCACTGGGTGCCATTCTGGTGTGT

TTGGTTGGATCCGTGTATTCGCCAAAGTCCGAGTTTTGTTAATAGGGTAAATGATGAAAT

GATAAACCGAACGAGTTGGTCAAACGAGCTATTGAAATGATATGAAAAAGTTGAATTGTG

AATTGAAATGTGAAATGAGATTGAGAAATGAACCTAAGGTTCGTGAATTATTCAAACTCA

AATTGTGGATATACGATATTGGTTGATGAATTGCTATTGTTGAAATATTTAATTTAAATT

GTATATACGATTTATGCTTTACATGTACATTATTGTTATAATTTGAATTATGGTAATACC

ACTGAGTATGAATTACTCAGCGTACGGTTGTTTCCGTGCGCAGGTCAATAGAAGTCAAAG

GTCTCGGTTCAGCATCCAGATTAATCCCGGCTTCGGCAAAACTTGGTGATGTATTTTTCC

TTTGGTAAAGGTGGCATGTACATAGATTGTGTATAAAGGTTATTATGTTTTATTATATAA

TGGTTAAAAATGTTAGTATTAAAAGTTTATGGATTTTAATGAAAGAAGTCTATCTATTTT

ATCTAATTAGTACATTGTTAAATTTTAAATTGGTATTAGATTGAGTTTGATTAGAAGTAT

TTAGAATAGAAAATGTGAATGTGAAATGAATTGGTTGAATTGATGATATTTGGGAACTAT

ATGGTTTTAATTTGC

>Deltapine607

ATGTAATGACCCAAAATTCATGGGCATCGGAAAAGTATAATATCGGGCCTCCGTCCTAGT

AAATTGAGTCCGAAAATAATTATTAGAAATATTTACGAGACTAGTAGTGTGTTTAATTAG

GTTTTAATTAAGTAAATTTAGCTTAATTTAGAGTAATTAGTAAAAAGGATTAAATTGAAT

AAGAGTAAAAGTTTAATTATAGATTAAAGGAAAATAATAGGGACCAAATGGGCAATTAAG

CCACATTTGGAAGTTGAGGCGGCATAACATTGTAAAAATCTTAGATTTTTATATTATTAT

TTATATAAATATATAAATTAATTATAAAGTATATTATTAAATTAATTATATTATAAATAT

TATATTATTATATATAAAAGAAACAAAACAGAAAAGAAACAGAATAGAAAGAACAAAGAA

ACAGAATAGAAGAGACGAAACAGGGGAGAAGCAGGGGAGAAAGAAGAAAAAGAAGAAAAA

AGGGGAAATAGGGTTTTTGAAGCTTGAAATTTAAATTGGTAAGTCAAATTAGCCATTTTC

TCTTAATTCTAATGTTTTAAAAGCTTTAAAACAAAGTTTTGATGGAATTAAGTTGATATT

TTGTAAGTTCATAGGTTTTCAAGTATAGTTTATGTTGAACAAAAGAGATGAATTAGGGAT

TAACTTGAAGGAATTTTAAGTTAGAATTGAAAAAGGGATTAAATTGTAAAAGAAACTATA

AGTTTTTTTTGTTTTAGGGACTAGATTGAGGAAAATTCGGAATTAAGAAAATATGTTAAA

AATTTAATAGTTAAATTTGAGTTTAAATGAAATTTGAATAGGAATAAGGTGTGAATTGGT

GTTATAAATTTGGTTATTAACATTTTTAATCAAAACAGTTTTGGGAAGTAGCAATGGTCT

GACTTTGAAAATTCACTAAAAATTTTATAAATTGAACTAGAGGATGAACAAAATATGGAA

TTAAAGCTTATTGAGTCTAGTTTCTTATAGTAGAAACAATGTAAGCAATTAATTGATGAA

TCAAGAGATATTTGAAATTTTGTAATACTGGTTCGGGGTGATTTCGAGATGCCCTGTTTT

AACTTTGGAAAATCATTAAAAATTGTACAAAAATTATTATGGAGTGTAATTTATATATGT

GAACTCCTTAATGAATCTAGTTTCAAAATAAATAAACAAGAACCTTATTCGAGTTCTGTA

CAATGAGATAATTTAGTTTTAGTGGAGAGAGGTCAGAACTGTCAAATGAAATAACAGGGG

AGTATTTAACGAATAAACTGTATTAAATGGCTAGACCAAAAATTCTGGAAATTTTATGAT

TAGAAGATATATGAGTCTAGTTTTAAGGAAAATTTACGGATATTAATTTGGAGTTTCGTA

GCTCAAGATATAAATAATTTAGTAACAATGACCCAAGTAGACAGCTTAATGGTGAAATTA

TATAAATACATTAAAAATGGTTAAATTTGCATGTTTAGGCTCATGAATTAAATTGAATCA

TGTTGTATTGATTATTATAAATTATTATTTTCGTAGCCAACAAAGAACCTAAAGCATCAG

CATCGAAAGGAAAGGAGAAAGTCATCGAGGAGTAAACTCGAGAAAATTACGGTTTGTATT

ACTATAATTCAAGTTATTTATTATTAAATGTTAAATTTTAATTTATGTGTCTAGTAAATG

AAATGTGAGGTAAGTATTATTATTATTATTATTATTATTATTATTATTATTATTATTATT

ATGAGTGGGAATTAAATTGAATAGTTGATATGAAATAATATTTGAATTGTTTGTTGATTG

AAAGCGGGAAATGAATTTAAATCGAATAGTGACCGATATTAAATTGAATGGAAATGTATT

GAGTTGTGAAAATATGTTAATTGCGGATTAATTATTGATTGAAAGGTGGAAAAATGATTG

AATTGAAAGTGTGAGAAAGTGTGATTGAATTGGGATTATATGTGATTTAAATACCCTATT

AACTAGTCGGGCTGAGTCGGATATAGTTGGCATGCCATAGGATTGGAAGAGTTCAGGGAT

ACTTCGACCTCGAGTCGATGAGACACTGGGTGATTTCTTCGGATAGATTGGATGAGGTAC

TGGGTACCAACTTTCTTCGGCTTTGCCGATGAGACACTGGGTGTCAACTATTGCTTCGAA

CTATCCGATGAGGCACTGGGTGCCATTCTGGTGTGTTTGGTTGGATCCGTGTATCCGCCA

AAGTCCGAGTTTTGTTAATAGGGTAAATGATGAAATGATAAACCGAACGAGTTGGTCAAA

CGAGCTATTGAAATGATATGAAAAAGTTGAATTGTGAATTGAAATGTGAAATGAGATTGA

GAAATGAACCTAAGGTTCGTGAATTATTCAAACTCAAATTGTGGATATACGATATTGGTT

GATGAATTGCTATTGTTGAAATATTTAATTTAAATTGTATATACGATTTATGCTTTACAT

GTACATTATTGTTATAATTTGAATTATGGTAATACCACTGAGTATGAATTACTCAGCGTA

CGGTTGTTTCCGTGCGCAGGTCAATAGAAGTCAAAGGTCTCGGTTCAGCATCCAGATTAA

TCCCGGCTTCGGCAAAACTTGGTGATGTATTTTTCCTTTGGTAAAGGTGGCATGTACATA

GATTGTGTATAAAGGTTATTATGTTTTATTATATAATGGTTAAAAATGTTAGTATTAAAA

GTTTATGGATTTTAATGAAAGAAGTCTATCTATTTTATCTAATTAGTACATTGTTAAATT

TTAAATTGGTATTGTGTAGATTGAGTTTGATTAGAAGTATTTAGAATAGAAAATGTGAAT

GTGAAATGAATTGGTTGAATTGATGATATTTGGGAACTATATGGTTTTAATTTGCAGGGG

GTTTTATGTAAAAATAAGCAGAAATGCTGCCGAAATTTTTATAAAAAAAAATGAAGTCAT

TTGGTAAACAAATTAATAAATTTTATGAATTATTTTAATATATTGGTTATTTATTTAAGA

ATTGTTGTAAATCGTTCGATACGTCCGGTAGTGCCTCGTAATTCTGTTCCGGCGACGGTT

CGGGGTTAAGGGGTGTTACATTTTATGGTATCAGAGCTATCAGGTTTAGCCGATTCTCGG

CCTAAATCGAGCTCGGAATTGAGTCTAGATGTACATGCCACTGTCGAGTTAAACTGAGTC

GGGATTTTTGGATGCTGACCTATTTGTTTGTTTTGTTTTATAGATTAAAGATGTCTGAAG

AAAGAATAAATGATACTGATGAAAGAATGTATAGTGAAGATAGAGAATTAGATGAAACAG

AATCTGTTGCACCGAGTGTGAATCCGTTAGGCAACCAACCTTCTAATGTAGAACGAGAAA

ATGTCAGAGATAGAGATGAATCCCAATTACTGAGAATTATAGCTGATGCATTACAAAGAG

TAGCAGGAACTACTCCTGTTACGACTTCAGTACCTACTGTTAGACGGGCTCCGATAAAGG

AACTGAGGAAATATGGTGCCACTGAATTTATGGGTCTAAAAGGAGTTGATCCATCCATAG

CTGAAAATTGGATGGAGTCGACTAAAAGAATTTTGCAGCAATTGGATTGTACCCCCCGAG

AGTGTTTAATCTGTGCCGTATCGTTATTACAAGGGGAGGCTTATCTATGGTGGGAATCAG

TGGTTCGACATTTACCAGAGAGTCAGATAACGTGGGATCTATTTCAGAAGGAGTTTCAAA

AGAAATATATCGGAGAGATGTATATTGAAGACAAGAAACAAGAGTTTTTGTTGCTACAAC

AGGGTGATATGTCAGTAATAGATTATGAGAGGGAATTCTCGAGACTCAGTAGATATGCCT

CCGAGTTTATTCCGACAGAAGCCGATAGTTGTAAAAGATTTTTACGGGGTTTACGAGACG

AGATCAAAGTGCAGCTAGTATCCCATCGGATCACTGAGTTAGTAGATTTGATTGAACGAG

CTAAAATGGTGGAACAAGTTCTGGGCCTCGACAAAAAGACTGAAGTTGTTAGACCAACCG

GGAAGCGTACAGGAACTACCAGTTCGAATCCTCAGCCGAAAAGACCAAAGGAATTCCAAA

GTGGTTGGAGATCCAGTTTCAGGTCAGACAGAGGTGGTAGAAATAGGGGAAAACAGACGA

TGACATCTACTGGCAGTGTGAAAGGTCCTTCCCGAGAAATAGATATTCCAGACTGCCAAC

ACTGCGGAAAGAAACACAGAGGGGAATGTTGGAAATTAACTAGAGGCTGTTTTCGATGTG

GTTCTACAGACCATTTCATCAGAGACTGTCCGAAAGTTGATAGTACTGTACCCGTGACAT

CACAGAGATCGGTATCTACAGCTAGAGGCAGAGGGTTAGGAAGAGGTGGTTCGGTTTCAA

GGGGAGGAAGTATTAGGAGAAGCAATGATATTGCTACTCAGCAGTCTGAGGCTAAAGTAC

CTGCCAGAGCTTATGTGGTCAGAACACAGGAAGAAGGTGACGCCCACGATGTAGTAACAG

GTATATTCTTACTATATTCTGAGCCTGTTTATGCTTTAATTGATCCCGGATCTTCACATT

CTTATATAAATTCAAAATTAGTTGAATTGGGAAAATTTAATTCTGAAATATCTAGAGTGA

CTGTAGAAGTGTCGAGTCCGTTGGGGCAAACAGTATTAGTGAATCAGATCTGTCCGAGAT

GCCCGTTAATTATACAAAATAAAACTTTTCCTATTGACCTGTTGATTATGCCATTTGGAG

ATTTTGATATAATACTGGGGATGGATTGGTTGGCTGAGCACGGAGTGGTATTGGATTGTT

ATAAAAAGAAGTTTAGTATTCAGACAGAAGACGGGGACAGAATTGAAGTAAATGGTATCC

GTACTAATGGGCCGACACGTATTATTTCGGCAATAAAGGCTAATAAATTGCTTCAGCGGG

GTTGTACAGCGTATTTAGCCTATGTTATTAATTCTGATTTGGTTGGTAGTCAGTGCAGTA

AGATTAGAACCGTATGTGAGTTTCCAGATGTATTTCCTGAAGAGCTACCGGGTTTACCAC

CTGACAGAGAGGTTGAATTTGCTATAGAAGTGTATCCGGGTACAGCACCAATCTCTATAC

CACCGTATCGAATGTCACCCACTGAGTTGAAAGAGTTGAAAGTGCAGTTACAGGACTTGT

CAGATCGTGGATTTATTAGACCGAGCATCTCACCTTGGGGAGCTCCAGTATTGTTTGTTA

AAAAGAAAGATGGATCGATGCGGCTTTGTATTGATTACCGGCAGTTAAACAAAGTGACGA

TCAAGAACCGGTATCCGTTACCCCGTATAGATGATTTATTTGATCAACTAAAAGGAGCTT

CAGTATTTTCAAAGATTGACTTAAGATCTGGGTATTATCAGCTGAAGGTAAAAGAAAGTG

ATGTTCCGAAGACTGCATTTCGTACTCGATATGGTCATTATGAATTTTTGGTGATGCCGT

TCGGGTTGACTAATGCTCCAGCTGCTTTTATGGATCTGATGAATCGTATTTTTCAGCCGT

ATTTAGATCAGTTTGTGGTGGTTTTTATTGATGACATCTTGGTTTATTCGAAGTCAGAGT

CAGAGCATGATCAGCATCTCAGAACCGTGCTACAAATTCTGCGAGAAAAACAGTTGTACG

GGAAACTAAGTAAATGTGAATTCTGGTTATCAGAGGTAGTATTCTTGGGACATGTTGTAT

CTGCGGATGGGATTAGAGTTGATCCGAAGAAGATCGAGGCAATTGTTCAATGGAAGGCAC

CAAAGAATGTATCAGAGGTACGCAGTTTTCTTGGTTTGGCTGGGTATTACAGAAGATTTG

TAAATGGGTTTTCGAAGATAGCTTTGCCGATGACCAAATTACTACAGAAGAATGTTCCAT

TTATCTGGGATGATCAGTGTCAGAGGAGCTTTGAAACATTGAAACAGATGTTGACAGAGG

CACCAGTTTTAACTTTACCAGAATCAGGGAAAGATTTCATAGTGTACAGTGATGCTTCTT

TGAATGGTTTGGGTTGTGTATTGATGCAAGAAGGAAAAGTAATAGCTTATGCATCTCGAC

AGTTGAAGTCACATGAACGCAACTACCCGACACACGATTTAGAGTTAGCTGCTGTAATCT

TTGCATTGAAGATTTGGATACATTACTTGTATGGTGAGAAATGTTATATTTACACTGATC

ATAAAAGTCTAAAATATCTTCTGTCACAAAAGGAGTTGAATCTGAGACAGAGACGGTGGA

TTGAACTTCTGAAAGATTATGATTGTGTTATAGATTATCATCCAGGGAAGGCAAATGTGG

TAGCAGATGCATTGAGTAGAAAAGCAGCGATTGAATTACGAGCAATGTTCGCTCGACTTA

GTATTAAGGATGATGGAAGTTTGTTAGCTGAGTTAAGAGTCAAGCCGGTGATGTTTGATC

AAATCAGAGCAGCACAGTTAAAAGATGAAAAGTTGATGAGGAAAAGAGAAATGGTACAGT

ATGGTGCGGTAGAAAATTTTAGTATTGACGAGCATGATTGTTTGAGATTTCGAAATCGAA

TTTGTGTTCCATCTACTTCTGAGATTAAAGAATTGATTCTCCGAGAAGCACATAATAGTA

TTTTTGCTTTGCACCCAGGAGGAACGAAGATGTATCGTGATCTACGAGAACTGTATTGGT

GGCCAGGAATGAAGAAAGATATAGTTGAATATGTCAGTAAATGCTTGACTTGTCAGCGGG

TAAAAGCAGAACATCAGGTACCAACAGGCCTGTTACAGCCTATTACTATTCCCGAGTGGA

AATGGGATCGCATTACCATGGATTTTGTTACGGGGTTGCCATTGTCAGTGAGTAAAAAGA

ATGCTATTTGGGTGATTGTTGATCGACTCACAAAATCAGCTCATTTTATAGCAGTTAGAA

CCGACTGGTCATTACAGAAGCTTGCCGAGGTTTATATTCGAGAAATTGTTAGATTACATG

GTATTCCGGTATCAATAATTTCAGACAGAGATCCTCGATTCACTTCGAGATTTTGGAAGC

AGCTGCATGAATCATTGGGTACTCGACTTAGTTTCAGTACAGCTTTTCATCCTCAAACTG

ATGGACAATCTGAACGAGTAATTCAGATATTAGAAGATATGCTTCGAGCTTGTGTCATTG

ATTTTGAATCAGGTTGGGAACGTTATTTACCATTGGCCGAGTTTGTTTATAATAATAGTT

TCCAATCTAGTATTCAAATGGCTCCATATGAAGCACTTTATGGTCGAAGGTGTCGATCAC

CAATATGTTGGACAAAATTAAGAGAAAGAAAAGTGATTGGGCCGGAATTGATTCAAGAGA

CAGAAGAAACAGTTAAAAAGATTAAAGATAGACTGAAAGCCGCTTTCGACAGACAGAAAT

CTTACGCAGACTTGAAACGACGAGACATTGAATATTCCGTTGGTGATAAGGTATTCCTCA

AAGTATCGCCGTGGAAGAAAATTTTGAGATTTGGTCGGAAGGGAAAATTAAGTCCGCGCT

TTATTGGGCCGTATGAGATAGTGGAAAGAATTGGGCCTGTTGCTTATCGATTATCCTTAC

CTCCAGAGTTACAGAAAATTCATGATGTTTTTCATGTTTCGATGCTTCGGAGATATAGAT

CGGATCCTTCTCATGTTATTCCCACTGAAGACATTGAACTTCGATCTGATTTAACTTATG

AAGAAGAACCAGTTCAAATATTAGCACGAGAAGTGAAAGAATTAAGAAATAAACGGGTTC

CTTTAGTACAAGTTTTATGGAGAAGCCATAGTGTGGAAGAAGCAACTTGGGAACCGGAAG

AGACAATGAGAGCACAATATCCTCATCTCTTCTCAGGTAAATTTCGAGGACGAAATTTAT

TAAGAGGGGGAGAAATGTAATGACCTAAAATTCATGGGCATCGGAAAAGTATAATATTGG

GCCTCCGTCCTAGTAAATTGAGTCCGAAAATAATTATTAGAAATATTTACGAGACTAGTA

GTGTGTTTAATTAGGTTTTAATTAAGTAAATTTAGCTTAATTTAGAGTAATTAGTAAAAA

GGATTAAATTGAATAAGAGTAAAAGTTTAATTATAGATTAAAGGAAAATAATAGGGACCA

AATGGGCAATTAAGCCACATTTGGAAGTTGAGGCGGCATAACATTGTAAAAATCTTAGAT

TTTTATATTATTATTTATATAAATATATAAATTAATTATAAAGTATATTATTAAATTAAT

TATATTATAAATATTATATTATTATATATAAAAGAAACAAAACAGAAAAGAAACAGAATA

GAAAGAACAAAGAAACAGAATAGAAGAGACGAAACAGGGGAGAAGCAGGGGAGAAAGAAG

AAAAAGAAGAAAAAAGGGGAAATAGGGTTTTTGAAGCTTGAAATTTAAATTGGTAAGTCA

AATTAGCCATTTTCTCTTAATTCTAATGTTTTAAAAGCTTTAAAACAAAGTTTTGATGGA

ATTAAGTTGATATTTTGTAAGTTCATAGGTTTTCAAGTATAGTTTATGTTGAACAAAAGA

GATGAATTAGGGATTAACTTGAAGGAATTTTAAGTTAGAATTGAAAAAGGGATTAAATTG

TAAAAGAAACTATAAGTTTTTTTTGTTTTAGGGACTAGATTGAGGAAAATTCGGAATTAA

GAAAATATGTTAAAAATTTAATAGTTAAATTTGAGTTTAAATGAAATTTGAATAGGAATA

AGGTGTGAATTGGTGTTATAAATTTGGTTATTAACATTTTTAATCAAAACAGTTTTGGGA

AGTAGCAATGGTCTGACTTTGAAAATTCACTAAAAATTTTATAAATTGAACTAGAGGATG

AACAAAATATGGAATTAAAGCTTATTGAGTCTAGTTTCTTATAGTAGAAACAATGTAAGC

AATTAATTGATGAATCAAGAGATATTTGAAATTTTGTAATACTGGTTCGGGGTGATTTCG

AGATGCCCTGTTTTAACTTTGGAAAATCATTAAAAATTGTACAAAAATTATTATGGAGTG

TAATTTATATATGTAAACTCCTTAATGAATCTAGTTTCAAAATAAATAAACAAGAACCTT

ATTCGAGTTCTGTACAATGAGATAATTTAGTTTTAGTGGAGAGAGGTCAGAACTGTCAAA

TGAAATAACAGGGGAGTATTTAACGAATAAACTGTATTAAATGGCTAGACCAAAAATTCT

GGAAATTTTATGATTAGAAGATATATGAGTCTAGTTTTAAGGAAAATTTACGGATATTAA

TTTGGAGTTTCGTAGCTCAAGATATAAATAATTTAGTAACAATGACCCAAGTAGACAGCT

TAATGGTGAAATTATATAAATACATTAAAAATGGTTAAATTTGCATGTTTAGGCTCATGA

ATTAAATTGAATCATGTTGTATTGATTATTATAAATTATTATTTTCGTAGCCAACAAAGA

ACCTAAAGCATCAGCATCGAAAGGAAAGGAGAAAGTCATCGAGGAGTAAACTCGAGAAAA

TTACGGTTTGTATTACTATAATTCAAGTTATTTATTATTAAATGTTAAATTTTAATTTAT

GTGTCTAGTAAATGAAATGTGAGGTAAGTATTATTATTATTATTATTATTATTATTATTA

TTATTATTATTATTATTATGAGTGGGAATTAAATTGAATAGTTGATATGAAATAATATTT

GAATTGTTTGTTGATTGAAAGCGGGAAATGAATTTAAATCGAATAGTGACCGATATTAAA

TTGAATGGAAATGTATTGAGTTGTGAAAATATGTTAATTGCGGATTAATTATTGATTGAA

AGGTGGAAAAATGATTGAATTGAAAGTGTGAGAAAGTGTGATTGAATTGGGATTATATGT

GATTTAAATACCCTATTAACTAGTCGGGCTGAGTCGGATATAGTTGGCATGCCATAGGAT

TGGAAGAGTTCAGGGATACTTCGACCTCGAGTCGATGAGACACTGGGTGTCACTATATTT

CTTCGGATAGATTCGATGAGGTACTGGGTACCAACTTTCTTCGGCTTTGCCGATGAGACA
[truncated: 1,068,904 more chars]
